# Identification of Antioxidants from Sequence Information Using Naïve Bayes

**DOI:** 10.1155/2013/567529

**Published:** 2013-08-24

**Authors:** Peng-Mian Feng, Hao Lin, Wei Chen

**Affiliations:** ^1^School of Public Health, Hebei United University, Tangshan 063000, China; ^2^Key Laboratory for NeuroInformation of Ministry of Education, Center of Bioinformatics, School of Life Science and Technology, University of Electronic Science and Technology of China, Chengdu 610054, China; ^3^Department of Physics, School of Sciences, Center for Genomics and Computational Biology, Hebei United University, Tangshan 063000, China

## Abstract

Antioxidant proteins are substances that protect cells from the damage caused by free radicals. Accurate identification of new antioxidant proteins is important in understanding their roles in delaying aging. Therefore, it is highly desirable to develop computational methods to identify antioxidant proteins. In this study, a Naïve Bayes-based method was proposed to predict antioxidant proteins using amino acid compositions and dipeptide compositions. In order to remove redundant information, a novel feature selection technique was employed to single out optimized features. In the jackknife test, the proposed method achieved an accuracy of 66.88% for the discrimination between antioxidant and nonantioxidant proteins, which is superior to that of other state-of-the-art classifiers. These results suggest that the proposed method could be an effective and promising high-throughput method for antioxidant protein identification.

## 1. Introduction

Oxidation is a chemical reaction that transfers electrons or hydrogen from a substance to an oxidizing agent. Oxidation reactions can produce free radicals. In turn, these radicals can start chain reactions. When the chain reaction occurs in a cell, it can cause damage or death to the cell. Moreover, oxidative stress is also the cause and the consequence of disease. Antioxidants are protein molecules that terminate these chain reactions by removing free radical intermediates and inhibit other oxidation reactions. They do this by being oxidized themselves, so antioxidants are often reducing agents such as thiols, ascorbic acid, or polyphenols [1].

Antioxidants are widely used in dietary supplements and have been investigated for the prevention of diseases such as cancer, coronary heart disease, and even altitude sickness. Plants and animals maintain complex systems of multiple types of antioxidants, such as glutathione, vitamin A, vitamin C, and vitamin E, as well as enzymes such as catalase, superoxide dismutase, and various peroxidases. Insufficient levels of antioxidants or inhibition of the antioxidant enzymes can cause oxidative stress and may damage or kill the cells.

As oxidative stress appears to be an important part of many human diseases, the use of antioxidants in pharmacology is intensively studied, particularly as treatments for stroke and neurodegenerative diseases. Recently, Fernandez-Blanco et al. reported a computational model to identify antioxidant proteins based on star graph topological indices [2]. However, by analyzing Fernandez-Blanco et al.'s dataset, we found that sequences in their dataset share high-sequence similarities; some sequences in their dataset even share 100% sequences identity. It has been demonstrated that the predictive accuracy is closely related to sequence identity [3, 4], and high-sequence similarity can surely lead to the overestimation of predictive performance. Therefore, their results are not credible. There is an urgent need to develop efficient computational tools for antioxidant proteins identification.

In the current study, we propose a Naïve Bayes-based computational model for predicting antioxidant proteins using amino acid compositions and dipeptide compositions. The correlation-based feature subset selection algorithm [5] was introduced to find the optimal feature set. By using the optimized features, the proposed model was evaluated in a benchmark dataset in the jackknife test.

According to some recent comprehensive reviews [6, 7] a series of recent publications [8–13], to establish a really useful statistical predictor, we need to consider the following procedures: (i) construct or select a valid benchmark dataset to train and test the predictor; (ii) formulate the statistical samples with an effective mathematical expression that can truly reflect their intrinsic correlation with the target to be predicted; (iii) introduce or develop a powerful algorithm (or engine) to operate the prediction; (iv) properly perform cross-validation tests to objectively evaluate the anticipated accuracy of the predictor; (v) and establish a user-friendly webserver for the predictor that is accessible to the public. Below, let us describe how to deal with these steps one by one.

## 2. Materials and Methods

### 2.1. Dataset

Fernandez-Blanco et al. have constructed a dataset containing 324 proteins with antioxidant activity and 1657 proteins without [2]. However, sequences in their dataset share high-sequence identity. The predictive accuracy is closely related to the sequence identity [3, 4], and high-sequence similarity can surely lead to the overestimation of predictive performance.

In order to prepare a reliable benchmark dataset, we first extracted proteins with antioxidant activities from the UniProt [14] according to the following steps: (i) only proteins with the experimentally confirmed antioxidant activities were included; (ii) the proteins which are fragments of other proteins were dislodged; (iii) and proteins containing nonstandard letters, that is, “B”, “X”, or “Z”, were excluded as their meanings are ambiguous. After following the aforementioned strict screening procedures, we obtained 686 proteins with antioxidant activity and obtained a new raw dataset by merging the 686 proteins into Fernandez-Blanco et al.'s dataset [2]. 

For balancing the number of samples and providing a significant statistics, sequences which have >60% sequence similarity were removed from the new raw dataset using CD-HIT program [15]. If the sequence identity cutoff is set to a stringent threshold of 25%, the results will be more objective and reliable. However, in this study we did not use such a stringent criterion because the currently available data do not allow us to do so. Otherwise, the number of antioxidant proteins would be too low to have statistical significance. Finally, a benchmark dataset containing 254 antioxidant and 1567 nonantioxidant proteins was constructed and can be found in the online Supporting Information S1 available online at http://dx.doi.org/10.1155/2013/567529. For further estimating the performance of the method, we also collected 20 antioxidant proteins (supporting information S2) which are independent from the training set.

### 2.2. Feature Vector

One of the most important parts for identifying protein attributes is to generate a set of proper informative parameters to encode protein sequences. The amino acid composition and dipeptide composition are the most important and effective parameters which have been widely applied in the realm of protein prediction [10–13]. Hence, every protein sequence in the benchmark dataset was encoded in a discrete vector as follows:

(1) $$\begin{array}{c} F={\left[f_{1},f_{2},\ldots ,f_{420}\right]}^{T}, \end{array}$$

where *f*_*i*_ are the normalized occurrence frequencies of the 20 amino acids (*i* = 1, 2,…, 20) and the 400 dipeptides (*i* = 21, 22,…, 420) in the protein sequence, respectively. **T**  is the transposing operator.

### 2.3. Feature Selection

Inclusion of redundant and noisy features in the model building process would cause poor predictive performance and increased computation time. Feature selection is the process of removing irrelevant features and is extremely useful in reducing the dimensionality of the data and improving the predictive accuracy. To reduce the dimension of the feature space and improve the predictive accuracy, the filter method Correlation-based Feature Selection [5] combined with best-first search strategy was used in the process of feature selection in the current work. 

The process starts with an empty set of features and generates all possible single-feature expansions. The subset with the highest accuracy is chosen and expanded in the same way by adding single features. If when expanding a subset the accuracy does not maximize, the search drops back to the next best unexpanded subset and continues from there until all features are added. The subset with the highest accuracy will be selected as the final optimized feature set [16].

### 2.4. Naïve Bayes

Naïve Bayes is an effective statistical classification algorithm [17] and has been successfully used in the realm of bioinformatics [18–20]. The theory of Naïve Bayes is to assume the attribute variables to be independent from each other given the outcome. This assumption greatly simplifies the calculation of conditional probabilities.

In the Naïve Bayes framework, a classification problem can be seen as the problem of finding the outcome with maximum probability given a set of observed variables. Given the protein example described by its feature vector **F** = (*f*_1_, *f*_2_,…, *f*_*n*_), we need to look for a class **C** that maximizes the likelihood **P**(**F** | **C**) = **P**(*f*_1_, *f*_2_,…, *f*_*n*_ | **C**). Since the current work is intended to classify antioxidant and nonantioxidant proteins, a binary class **C** ∈ (0,1) was generated, where 1 denotes the sample that was predicted as an antioxidant protein and 0 denotes nonantioxidant protein. For the binary classification, the class for the protein sample could be determined by comparing two posteriors as follows:

(2) P(C=1 ∣ F=f1,f2,…,fn)P(C=0 ∣ F=f1,f2,…,fn) =P(C=1)∏i=1nPi(fi ∣ C=1)P(C=0)∏i=1nPi(fi ∣ C=0).

Taking the logarithm of (2), we have

(3) log⁡P(C=1 ∣ F=f1,f2,…fn)P(C=0 ∣ F=f1,f2,…fn) =log⁡P(C=1)P(C=0)+∑i=1nlog⁡Pi(fi ∣ C=1)Pi(fi ∣ C=0).

Hence, the sample will be predicted as 1 (antioxidant protein) if

(4) $$\begin{array}{c} \log\frac{P\left(C=1\;|\;F=f_{1},f_{2},\ldots ,f_{n}\right)}{P\left(C=0\;|\;F=f_{1},f_{2},\ldots ,f_{n}\right)}\geq \theta , \end{array}$$

and 0 (nonantioxidant protein) for otherwise. *θ* is the threshold determining the tradeoff between sensitivity and specificity and can be trained on the training dataset to maximize the prediction performance.

### 2.5. Performance Evaluation

The performance of the proposed model was evaluated using sensitivity, specificity (Garmer, Sperling, and Forsberg), and accuracy (Acc), which are expressed as follows:

(5) $$\begin{array}{c} \text{Sn}=\frac{\text{TP}}{\text{TP}+\text{FN}},\\ \text{Sp}=\frac{\text{TN}}{\text{TN}+\text{FP}},\\ \text{Acc}=\frac{\text{TP}+\text{TN}}{\text{TP}+\text{FN}+\text{TN}+\text{FP}}. \end{array}$$

TP, TN, FP, and FN represent the number of the correctly recognized antioxidant proteins, the number of the correctly recognized nonantioxidant proteins, the number of nonantioxidant proteins recognized as antioxidant proteins, and the number of antioxidant proteins recognized as nonantioxidant proteins, respectively. 

As the performance of the current classifier depends on the threshold *θ* as given in (4), the receiver operating characteristic (ROC) curve was employed. Therefore, the quality of a classifier can be objectively evaluated by measuring the area under the receiver operating characteristic curve (auROC). The value of auROC score ranges from 0 to 1, with a score of 0.5 corresponding to a random guess and a score of 1.0 indicating a perfect separation.

## 3. Results and Discussion

Three cross-validation methods, namely, subsampling test, independent dataset test, and jackknife test, are often employed to evaluate the predictive capability of a predictor. Among the three methods, the jackknife test is deemed the most objective and rigorous one that can always yield a unique outcome as demonstrated by a penetrating analysis in a recent comprehensive review [21], and hence has been widely and increasingly adopted by investigators to examine the quality of various predictors (see, e.g., [8, 22–28]). Accordingly, the jackknife test was used to examine the performance of the model proposed in the current study. In the jackknife test, each sequence in the training dataset is in turn singled out as an independent test sample and all the rule parameters are calculated without including the one being identified.

### 3.1. Prediction of Antioxidant Proteins

We trained the Naïve Bayes classifier using Waikato Environment for Knowledge Analysis (WEKA) [29] on the benchmark dataset. As shown in Table 1, in the jackknife test, an auROC score of 0.68 and an accuracy of 55.85% with an average sensitivity of 75.59% and an average specificity of 52.65% were obtained for the classification of antioxidant and nonantioxidant proteins by using all the 420 features, that is, 20 amino acid compositions and 400 dipeptide compositions.

For saving computing time, cross-validation methods (fivefold or tenfold) are widely used for feature selection in computational proteomics [16, 30]. In order to identify prominent features that can distinguish between antioxidant and nonantioxidant proteins, feature selection method was also carried out to eliminate the redundant features using WEKA in a ten-fold cross-validation approach on the benchmark dataset. In the ten-fold cross-validation, the benchmark dataset is split into ten pieces, and cross validation is performed using each of these ten pieces as the testing set. Thus, the training process is performed ten times, each of which uses the data obtained by deleting the testing set from the whole dataset. We found that the proposed method achieved a maximum accuracy of 66.89% and auROC of 0.762 when the feature dimension reduced to 44 (i.e., C, G, FP, FW, LK, LS, IE, VL, VH, VC, VW, MS, PD, AP, AY, YQ, YE, YR, HE, HG, QA, KA, KH, DF, DK, DR, EF, EM, EY, ER, CP, CN, CG, WC, RT, RD, RW, SV, SD, GV, GY, GK, GC).

The jackknife test results of the Naïve Bayes classifier based on the 44 optimized features for identifying antioxidant proteins were listed in Table 1. As it can be seen from Table 1, the current method yielded a better auROC score of 0.855 and a predictive accuracy of 66.88% with an average sensitivity of 72.04% and an average specificity of 66.05% (Table 1). Both predictive accuracy and auROC are higher than those of the model based on the 420 features.

Moreover, for the purpose of evaluating the performance of the proposed method, we used the 20 experimentally-confirmed antioxidant proteins (in Supporting Information S2) to examine the method. As a result, 16 antioxidant proteins were correctly predicted by the proposed method; see Table 2. This result demonstrates the excellent performance of our model.

### 3.2. Comparison with Other Methods

In order to further testify its superiority, we compared the capability of the present model with that of other models based on different kinds of algorithms such as BayesNet, J48 tree, and Random forest. All the classifiers were compared on the benchmark dataset based on the optimized features (i.e., C, G, FP, FW, LK, LS, IE, VL, VH, VC, VW, MS, PD, AP, AY, YQ, YE, YR, HE, HG, QA, KA, KH, DF, DK, DR, EF, EM, EY, ER, CP, CN, CG, WC, RT, RD, RW, SV, SD, GV, GY, GK, GC). Their best predictive results from jackknife test were shown in Table 3.

Although the accuracies of BayesNet, J48 tree, and Random forest are higher than those of Naïve Bayes, their auROC scores and sensitivities are all much lower than those of Naïve Bayes. These results indicate that the proposed Naïve Bayes model can be effectively used to classify antioxidant and nonantioxidant proteins.

## 4. Conclusions

In this study, the Naïve Bayes classifier with feature selection method is presented to identity antioxidant proteins based on the primary sequence information. By using Correlation-based Feature Subset Selection algorithm, the feature dimensions were reduced to 44 prominent features that could remarkably improve the predictive accuracies. However, the detailed analyses of the selected features are required to provide more information about their roles in biological activity. It is expected that the presented model will provide novel insights into the research on antioxidants. Since user-friendly and publicly accessible webservers represent the future direction for developing practically more useful predictors [31], we shall make efforts in our future work to provide a webserver for the method presented in this paper.

## Supplementary Material

Supporting Information S1 is the benchmark dataset which consists of a positive dataset containing 254 antioxidant proteins and a negative dataset containing 1567 non-antioxidant proteins. Supporting Information S2 is the independent dataset containing 20 antioxidant proteins which are independent from those in the benchmark dataset.

## Figures and Tables

**Table 1:** Predictive performance of Naïve Bayes based on different features.

Feature dimensions	Sn (%)	Sp (%)	Acc (%)	auROC
420	75.59	52.65	55.85	0.680
44	72.04	66.05	66.88	0.855

**Table 2:** Predictive results based on the independent dataset.

UniProt ID	Predictive result
Q148E0	Antioxidant
Q7RTV5	Antioxidant
Q9D1A0	Antioxidant
P80239	Antioxidant
P0AE08	Antioxidant
Q7BHK8	Nonantioxidant
P0A251	Antioxidant
P0A5N4	Antioxidant
Q8L5E0	Antioxidant
P06728	Antioxidant
Q03247	Antioxidant
P23529	Nonantioxidant
P30041	Antioxidant
O19097	Antioxidant
P23345	Antioxidant
P23346	Antioxidant
O65198	Nonantioxidant
P93407	Antioxidant
P11964	Nonantioxidant
P10792	Antioxidant

**Table 3:** Comparison of Naïve Bayes with other methods by using optimized features.

Classifier	Sn (%)	Sp (%)	Acc (%)	auROC
BayesNet	42.12	92.53	85.50	0.800
J48 tree	26.37	90.81	81.82	0.565
Random Forest	28.35	97.64	87.97	0.797
Naïve Bayes	72.04	66.05	66.88	0.855

## References

[1] Sies H (1997). Oxidative stress: oxidants and antioxidants. *Experimental Physiology*.

[2] Fernandez-Blanco E, Aguiar-Pulido V, Munteanu CR, Dorado J (2013). Random Forest classification based on star graph topological indices for antioxidant proteins. *Journal of Theoretical Biolology*.

[3] Nair R, Rost B (2002). Sequence conserved for subcellular localization. *Protein Science*.

[4] Yu C-S, Chen Y-C, Lu C-H, Hwang J-K (2006). Prediction of protein subcellular localization. *Proteins*.

[5] Hall MA (2008). *Correlation-Based Feature Selection for Machine Learning: Data Mining, Inference and Prediction*.

[6] Chou K-C (2011). Some remarks on protein attribute prediction and pseudo amino acid composition (50th anniversary year review). *Journal of Theoretical Biology*.

[7] Chou KC (2013). Some remarks on predicting multi-label attributes in molecular biosystems. *Molecular Biosystems*.

[8] Chen W, Feng PM, Lin H, Chou KC (2013). iRSpot-PseDNC: identify recombination spots with pseudo dinucleotide composition. *Nucleic Acids Research*.

[9] Chen W, Lin H, Feng PM (2012). iNuc-PhysChem: a sequence-based predictor for identifying nucleosomes via physicochemical properties. *PLoS One*.

[10] Chou K-C, Wu Z-C, Xiao X (2012). ILoc-Hum: using the accumulation-label scale to predict subcellular locations of human proteins with both single and multiple sites. *Molecular BioSystems*.

[11] Lin WZ, Fang JA, Xiao X, Chou K-C (2013). iLoc-Animal: a multi-label learning classifier for predicting subcellular localization of animal proteins. *Molecular BioSystems*.

[12] Xiao X, Wang P, Lin WZ, Jiaa J-H, Choub K-C (2013). iAMP-2L: a two-level multi-label classifier for identifying antimicrobial peptides and their functional types. *Analytical Biochemistry*.

[13] Xu Y, Ding J, Wu LY, Chou K-C (2013). iSNO-PseAAC: predict cysteine S-nitrosylation sites in proteins by incorporating position specific amino acid propensity into pseudo amino acid composition. *PLoS One*.

[14] UniProt Consortium (2012). Reorganizing the protein space at the Universal Protein Resource (UniProt). *Nucleic Acids Research*.

[15] Li W, Godzik A (2006). Cd-hit: a fast program for clustering and comparing large sets of protein or nucleotide sequences. *Bioinformatics*.

[16] Chen W, Lin H (2012). Identification of voltage-gated potassium channel subfamilies from sequence information using support vector machine. *Computers in Biology and Medicine*.

[17] Mitchell TM (1997). *Machine Learning*.

[18] Cao J, Panetta R, Yue S, Steyaert A, Young-Bellido M, Ahmad S (2003). A naïve Bayes model to predict coupling between seven transmembrane domain receptors and G-proteins. *Bioinformatics*.

[19] Yousef M, Jung S, Kossenkov AV, Showe LC, Showe MK (2007). Naïve Bayes for microRNA target predictions—machine learning for microRNA targets. *Bioinformatics*.

[20] Murakami Y, Mizuguchi K (2010). Applying the Naïve Bayes classifier with kernel density estimation to the prediction of protein-protein interaction sites. *Bioinformatics*.

[21] Sambo F, Trifoglio E, di Camillo B, Toffolo GM, Cobelli C (2012). Bag of Naïve Bayes: biomarker selection and classification from genome-wide SNP data. *BMC Bioinformatics*.

[22] Chou KC, Shen HB (2010). Cell-PLoc 2.0: an improved package of web-servers for predicting subcellular localization of proteins in various organisms. *Natural Science*.

[23] Chou K-C, Wu Z-C, Xiao X (2011). iLoc-Euk: a multi-label classifier for predicting the subcellular localization of singleplex and multiplex eukaryotic proteins. *PLoS One*.

[24] Hayat M, Khan A (2012). MemHyb: predicting membrane protein types by hybridizing SAAC and PSSM. *Journal of Theoretical Biology*.

[25] Hayat M, Khan A (2012). Discriminating outer membrane proteins with fuzzy K-nearest neighbor algorithms based on the general form of Chou’s PseAAC. *Protein and Peptide Letters*.

[26] Mei S (2012). Predicting plant protein subcellular multi-localization by Chou's PseAAC formulation based multi-label homolog knowledge transfer learning. *Journal of Theoretical Biology*.

[27] Mohabatkar H (2010). Prediction of cyclin proteins using chou’s pseudo amino acid composition. *Protein and Peptide Letters*.

[28] Xiao X, Wu Z-C, Chou K-C (2011). iLoc-Virus: a multi-label learning classifier for identifying the subcellular localization of virus proteins with both single and multiple sites. *Journal of Theoretical Biology*.

[29] Witten H, Frank E (2005). *Data Mining: Practical Machine Learning Tools and Techniques*.

[30] Ding C, Yuan LF, Guo SH, Lin H, Chen W (2012). Identification of mycobacterial membrane proteins and their types using over-represented tripeptide compositions. *Journal of Proteomics*.

[31] Chou KC, Shen HB (2009). Review: recent advances in developing web-servers for predicting protein attributes. *Natural Science*.

