# Supplementary material for: Identification of Antioxidants from Sequence Information Using Naïve Bayes
Source: Comput Math Methods Med. 2013 Aug 24;2013:567529. doi: 10.1155/2013/567529 (PMC3766563; doi:10.1155/2013/567529)
Supplement: Supplementary file 1 — Supporting Information S1 is the benchmark dataset which consists of a positive dataset containing 254 antioxidant proteins and a negative dataset containing 1567 non-antioxidant proteins. Supporting Information S2 is the independent dataset containing 20 antioxidant proteins which are independent from those in the benchmark dataset. [file 567529.f1.pdf]

Supporting Information S1. The benchmark dataset consists of a positive dataset and a negative dataset. The positive dataset contains 254 antioxidant proteins, while the negative dataset contains 1567 non-antioxidant proteins.

The positive dataset contains 254 antioxidant proteins

>antioxidant_1

MTKGILLGDKFPDFRAETNEGFIPSFYDWIGKDSWAILFSHPRDFTPVCTTELARLVQLAPE
FNKRNVKLIGLSCDSAESHRKWVDDIMAVCKMKCNDGDTCCSGNKLPFPIIADENRFLAT
ELGMMDPDERDENGNALTARCVFIIGPEKTLKLSILYPATTGRNFDEILRVVDSLQLTAVKL
VATPVDWKDGDDCVVLPTIDDTEAKKLFGEKINTIELPSGKHYLRMVAHPK

>antioxidant_2

MLPGLALLLLAAWTARALEVPTDGNAGLLAEPQIAMFCGRLNMHMNVQNGKWDSDPS
GTKTCIDTKEGILQYCQEVYPELQITNVVEANQPVTIQNWCKRGRKQCKTHPHFVIPYRCL
VGEFVSDALLVPDKCKFLHQERMDVCETHLHWHTVAKETCSEKSTNLHDYGMLLPCGID
KFRGVEFVCCPLAEESDNVDSADAEEDDSDVWWGGADTDYADGSEDKVVEVAEEEEVA
EVEEEEADDDEDDEDGDEVEEEAEEPYEEATERTTSIATTTTTTTESVEEVVREVCSEQAE
TGPCRAMISRWYFDVTEGKCAPFFYGGCGGNRNNFDTEEYCMAVCGSAMSQSLLKTTQE
PLARDPVKLPTTAASTPDAVDKYLETPGDENEHAHFQKAKERLEAKHRERMSQVMREW
EEAERQAKNLPKADKKAVIQHFQEKVESLEQEAANERQQLVETHMARVEAMLNDRRRL
ALENYITALQAVPPRPRHVFNMLKKYVRAEQKDRQHTLKHFEHVRMVDPKKAAQIRSQV
MTHLRVIYERMNQSLSLLYNVPAVAEEIQDEVDELLQKEQNYSDDVLANMISEPRISYGND
ALMPSLTETKTTVELLPVNGEFSLDDLQPWHSFGADSVPANTENEVEPVDARPAADRGLT
TRPGSGLTNIKTEEISEVKMDAEFRHDSGYEVHHQKLVFFAEDVGSNKGAIIGLMVGGVVI
ATVIVITLVMLKKKQYTSIHHGVVEVDAAVTPEERHLSKMQQNGYENPTYKFFEQMQN

>antioxidant_3

MAIALSSSSTITSITLQPKLKTIHGLGTVLPGYSVKSHFRSVSLRRSAVVVSAITGASSGAGI
GKGTADSLDTVKVLDLRGNEIPISDLWKDRKAVVAFARHFGCVLCRKRAAYLAEKKDVM
DASGVALVLIGPGSIDQANTFVEQTKFKGEVYADPNHASYEALEFVSGVSVTFTPKAAMKI
LESYMEGYRQDWKLSFMKDTVERGGWQQGGILVAGPGKDNISYIRKDKEAGDDPPVEEI
LKACCA

>antioxidant_4

MITSSKKIVSAMLSTSLWIGVASAAYAETTNVEAEGYSTIGGTYQDGNPQPINIYSVNGVQ
AINFVNRGDFAEYDVSVSTAGEYSIEYLIGTSIASGSAVEISVLVDGNWQSAGSTNVPLGQ
WDNFQALAANNNISLAQGTNRIKITGAGTHDWQWNLDAFSLTLVTPENPDNPDNPDNPD
DGNTGQPGTPFTIEMEAFDATGSDDPRAQGMVIGERGYPEDKHTVVDSNQTTDWVDYNI
NFPVSGNYRIEMLASGQTSHATAILFVDNVQINEVAVDTGNQAVFLDFELTDSTYISAGAH
TIRVQSGSQINEFSWMWFGDALTFTPLDGGSTDGDADNDGVLDSVDTCPNTPAGAQVDA
NGCEIIVDNDTDNDGVDNSIDQCPNTPAGAQVDANGCEIVAVVDADNDGVEDSLDMCPN
TPAGAPVNGQGCADSQLDADNDGVSDDIDQCPSTPAGSVVDGTGCIVVTPPADSDNDGV
VDTLDMCPNTAAGLTVDSQGCALSQLDSDNDGVTDDIDQCANTPSGETANATGCSSSQE
GGGTDPDTPQPGLLYGELAGAMNVSDTNPNWERTTDLLQTEDSVKGNTTEVYTGFIYDA
DGHISFYEHIDDSVRLYIDGVLVLSNDSWEASSQTTDLNLTPGTHEIELRIGNADGGSGAVD
GIGFGIDVDGGTNFVHPSTLSESIFTSVGEETGNPDLEQEGDIIVELESFVFTSTNGRVGSDS
VEGFSPTATGVNWVTNGDYGDYMVTFEEPGTYGAYITISAANDGSYGARVDVDGWPVA
WGYFGGTGSWDVSSENLLYGGTFVVEQAGEKVVRVEAIGGSDWQWSGDRVRFTRLGDV

TAIPSPIYNPDDHFVAEIQGPQTDVTYLKKPVEIPANKKVLKSDVWYTYPQNRELEGYDNF
GATGAFWGHPPEHDFYDDTVIMDWAVDAVYAFQAEGYEYTARGEFDWGYGWFTEYTTN
PQPHYVRTLDDRNVRMTFMGYLSHDGYNNNWLSNHSPAFVPFMKSQVDQILKANPDKL
MFDTQTNSTRSTDMRDFGGDFSPYAMENFRVWLSKKYSTGELAALGINDINSFDYGDFLR
AQGVTHTSWSNAGDTLSGNIPLQEDYIYFNRDVWNQKFAEVLDYIRQQQPDIEIGASTHL
FESRGYVFNENLTFLSGELNLGARTTISELPTNILVHLKGAQAVDKTLVYFPYPWEFDELRL
QDAPRFGRGWVAQAYAYGGLFSIPANVWVGGEVWTWSPGADNYRDIYLFVRAQADLLD
DYTSYSKVGLVHAMYSSMKAGFIDGGNQIQSSTKLLTEGNINFDLLVFGDEGYPVVPRPE
DFDKFDHIFFDGDEQYLTAEQQALLDQQGDKVRHIGQRGTVSGIEITVSISGTESNETVSAV
SRIHETDAAAPYVVHLVNRPFAGGVTPTLNNVEVAIPQSYFPEVVTGATLHLPDGTSTSLTL
STNADGDVVLPVNNLEVWGILELAH
>antioxidant_5
MANSGLWELITIGSAVRNVAKSYLKAEASSITAKQLYDASKITSSKRSTSDLHVQLLEKYR
NGKVKHASQIKELGLSSKDTKRTLLGNSLDVQKDASGVKHLQEQSSKEIKNPISQPILPNK
KDEISPAKPSAIDSSIKDVTKSHPATNANFTADFESSIEESYSTENKSPVILSSSKVPSSQWSR
LWHYGGLATSLSVGAIGEKMKRMWGISKDDGALLLNERNVEILVNKLTQMRGAALKMG
QMLSFQDSKLIDPRVSQILERVRDGAHSMPEKQLEQVMVKNLGKNWMTHYSEFDRKPM
AAASIGQVHRARLASNHMEVVVKVQYPGVMSSIDSDLNNLAYLLKASRILPKGLFLENSL
AAARKELKWECDYEREAAFAERFGSLLKNDSDFKVPMVFREASGPSVITLEYLHGIALGK
QKYSQATRNHIGYLLTKQCLREISEYHFMQTDPNWSNFLYNGKTKKIELLDFGASIEYDEK
FIKKYCRLLLAAAHRNREKCKKLSVELGYLNNHESAQMIDAHINSIFTLAEPFAFDAPDVY
DFGDQTITARVKQQIPVMLDLRLQPPPEETYSLHRRLSGHFLLCAKLGAKVRCKELFSGM
LKHYAD
>antioxidant_6
MAVTNVAELNALVERVKKAQREYASFTQEQVDKIFRAAALAAADARIPLAKMAVAESGM
GIVEDKVIKNHFASEYIYNAYKDEKTCGVLSEDDTFGTITIAEPIGIICGIVPTTNPTSTAIFKS
LISLKTRNAIIFSPHPRAKDATNKAADIVLQAAIAAGAPKDLIGWIDQPSVELSNALMHHP
DINLILATGGPGMVKAAYSSGKPAIGVGAGNTPVVIDETADIKRAVASVLMSKTFDNGVIC
ASEQSVVVVDSVYDAVRERFATHGGYLLQGKELKAVQDVILKNGALNAAIVGQPAYKIAE
LAGFSVPENTKILIGEVTVVDESEPFAHEKLSPTLAMYRAKDFEDAVEKAEKLVAMGGIGH
TSCLYTDQDNQPARVSYFGQKMKTARILINTPASQGGIGDLYNFKLAPSLTLGCGSWGGNS
ISENVGPKHLINKKTVAKRAENMLWHKLPKSIYFRRGSLPIALDEVITDGHKRALIVTDRF
LFNNGYADQITSVLKAAGVETEVFFEVEADPTLSIVRKGAELANSFKPDVIIALGGGSPMD
AAKIMWVMYEHPETHFEELALRFMDIRKRIYKFPKMGVKAKMIAVTTTSGTGSEVTPFAV
VTDDATGQKYPLADYALTPDMAIVDANLVMDMPKSLCAFGGLDAVTHAMEAYVSVLAS
EFSDGQALQALKLLKEYLPASYHEGSKNPVARERVHSAATIAGIAFANAFLGVCHSMAHK
LGSQFHIPHGLANALLICNVIRYNANDNPTKQTAFSQYDRPQARRRYAEIADHLGLSAPGD
RTAAKIEKLLAWLETLKAELGIPKSIREAGVQEADFLANVDKLSEDAFDDQCTGANPRYP
LISELKQILLDTYYGRDYVEGETAAKKEAAPAKAEKKAKKSA
>antioxidant_7
MSDLVNKKFPAGDYKFQYIAISQSDADSESCKMPQTVEWSKLISENKKVIITGAPAAFSPT
CTVSHIPGYINYLDELVKEKEVDQVIVVTVDNPFANQAWAKSLGVKDTTHIKFASDPGCA
FTKSIGFELAVGDGVYWSGRWAMVVENGIVTYAAKETNPGTDVTVSSVESVLAHL
>antioxidant_8

MALLTIGDQFPEYDLTAVVGGDLSKVDAKQPDDYFTRVTSKDYEGKWRIIFFWPKDFTFV
CPTEIAAFGKLNEDFEDRDAKVLGVSVDNEFVHFQWRAQHEDLKTLPFPMVSDLKRELT
AACGVLNADGVADRATFIVDPNNEVQFVSVTAGSVGRNVDEVLRVLDALQSDELCACN
WKKGDPTINAGELLAGAV
>antioxidant_9
MSLINKEILPFTAQAFDPKKDQFKEVTQEDLKGSWSVVCFYPADFSFVCPTELEDLQNQYE
ELQKLGVNVFSVSTDTHFVHKAWHDHSDAISKITYTMIGDPSQTITRNFDVLDEATGLAQ
RGTFIIDPDGVVQASEINADGIGRDASTLAHKIKAAQYVRKNPGEVCPAKWEEGAKTLQP
GLDLVGKI
>antioxidant_10
MPIDTVMETLPGYAKDIKLNYSTLVRQNTELTPQQLWGTVVASAIATRNDTLTAAALEDG
AQHLSEQALEAARIAAALMSMNNIFYRFQHLSSNEKYATMPARLRMNGMRTHGVEPVDF
ELWSLAVSAINGCGKCVDSHEKVLREKGAGEDLVLAAVRVASVIHAIGAVLDAEKVHQPE
AAMA
>antioxidant_11
MAALDAIREALPEPARDIKLNLQAVLQPGTLTPAQRWGVAVATAAAARNERLLAAALADA
RAEVEPAVIEDALAAAAVMAMNNVYYRFRHMVGKASYAEKPARLRMNRLVKPAASKVD
FELFALAVSAVNGCETCVRSHEQVVVAGGLSEDQVHEAVRIAAVLHAAAVSLELAGYAAV
PSAAAAAG
>antioxidant_12
MSIEALKTQLPAFAKDVKLNLSALPREDSLTEQQLYGLLVACGYTTRNGTVAQALEAEAA
PHLSPAALDAAKAAASIMAMNNVYYRFTHLASNKAYETLPAKLRMSVIGNPGVDKVDFE
LWSLAVSAMNGCGRCIDAHEAVLREAGLSEAQIQTAVRVGAIIASAAVALEAAGAGFPEAA
E
>antioxidant_13
MSPIEQIYDKIPDFAKDVRINLTSLMADETLSPRRKYGVLVASAVATRNSALIAAVESAASG
VMTSVAIAAAKAAASVVVMNNVYYRFVHLASNPEYKTMPPRLRMDVIGNPGVDKSDFE
LWSLAVSSINGCGICIDAHERTLRAAGVNSETIQTAVRFAAITQSVAIALEAAGPASPQAGD
>antioxidant_14
MSIEALRARLPDYAHDLGTNLALLVDDPALDPEARWGCFVASACAVGEPQTLRAIDAAAT
AAGLTAEANRAARKAAAMMAMTNVYFRAVHLMEGAAYQALPCRLRLNRLAHAGARGV
TYDLSCVAVSAINGCGACLDSHEADLRGRGVEPTQIQAALRIAAVVSAVARTLAAEAALHP
QNLEI
>antioxidant_15
MSIENLKSALPAYAKDQKLNIGSLTRSTELNEEQLWGSLVAAAAATRNDLVLSEILEEAREH
LSEEAVDAALGSATVMAMNNVAYRAKSWLGDDFAQVKFGLRMNIIAKPGVDKATFELWS
TVVSAINGCEHCLSAHANTLLEEGVTKEQIWEGIKVAGVVEAVAQALQAEAVRSAE
>antioxidant_16
MLQTYKDQLPDYAKDLKLNLTQVLSESPSSELSNQQITGVALAVAYATRNRQLIELIFQKAE
AELDESTLQAIKAAASIMAMNNIYYRFVHLVKDSEYQRLPANLRMNIIANPGIDKKDFELY
SLAVSAINGCGLCIDAHANTLIKAGFSKHSIQHVIRIAAVLNGLAQVSIIENKT
>antioxidant_17
MTVARLRELLPDYARDLRLNLGSVTSQSNLSAQQLWGTVLAAAIASRGRTVLVELEPEAL
DHLSAEAATAARTAAALMAMNNVYYRSLHLLEDEEYSRLRAGLRMNALANPGVDKVD

VELWSLAVSAVNGCGRCLTAHEHELRGRGVAREVIQDAIRVASVVHAVAVTVEALETSSGT
NRVAAAD
>antioxidant_18
MSLETLKTLIPDYAKDIRLNIGSLANETILSEQQKYGCYLASAHAVGEAQTLRAIEAEARG
KLSVEALNAAKAASAIMGMNNVYYRATHLVSNTTYTTMPARLRMNVIGNPGVEKVDFEL
WSLAVSAINGCGMCLDAHEAELRKHGVTSEQIQAAIRIGAVVNAAARVLAAEAALAAEP
A
>antioxidant_19
MNLEALLDTVPAYARDLKLNFSAVVRQNTELTEQQLWGTVVACAAASRNRTLLDAIVEEA
KGKLSAQAVEAAKGAAAILSMNNIFYRFQHLAVNKKYETMRAGLRMNFLRQHGVDPLD
FELWALAVSAVNGCGKCIDAHERVLLQKEFSEEKILAAIRVASTIYGLAVVFDAEEPKGDLV
GAPVNTDAYSTTTI
>antioxidant_20
MSIENLKNSLPEYAKDLKLNLSSIARSTVLNEQQLWGTLLASAAATRSATTLREIAEEAAD
VLSAEAYNAALGAASIMGMNNVFYRGKAFLGGRYDDLRAGLRMQIIGNPGVDKADFEL
WSFAVSSINGCAHCLEAHEHTLREAGVSREVIFESLRVAAIVAGVGQAVQSTEALAAAAV
>antioxidant_21
MSIEALKARLPDYAKDLRLNLGSVLTTSSLKPGQVWGAAIAAAYAARNDDVTRAIVAEAA
QHLDAAGLTAAKAAGAIMGMNNVYYRTVHLSGNADFKKIPARLRMNVIGNPGVEKVDF
ELWSLAASAVNGCGMCIEAHEREVMHKGMSTENIQDAVRIAAVIHAVATVLDAEAALSGQ
DGSTAVAA
>antioxidant_22
MELEQLLNSVPEYAKDLKLNMGGVLRQTELTEQQAWGTAVACAIAARNPQLRESVLGEA
AKHLNEQALFAAKAAASVMGMNNIFYRFRHLSTNPKYGAMPARLRMQVIAKHGSDPIDF
ELWCLAVSAMNGCGVCVDSHENVLREKGVSEEAVLAAIRIASTIHGLAAILDVEADPIA
>antioxidant_23
MSLDSLKSRIPDYAKDLKLNLGSVIGNSGLPAQQLWGTVLSTAIASRSAIVLRELEPEAKA
NLSPEAYTAAKAAAAVMAMNNVFYRTRHLLSDHEYGNLRAGLRMNVIGNPGVDKVDFE
FWSFAVSAINGCGMCLDSTKQVLRKAGVERRSSRRRSDCRGTQAVAATLDAEAFS
>antioxidant_24
MKWVTFISLLFLFSSAYSRGVFRRDAHKSEVAHRFKDLGEENFKALVLIAFAQYLQQCPFE
DHVKLVNEVTEFAKTCVADESAENCDKSLHTLFGDKLCTVATLRETYGEMADCCAKQEP
ERNECFLQHKDDNPNLPRLVRPEVDVMCTAFHDNEETFLKKYLYEIARRHPYFYAPELLFF
AKRYKAAFTECCQAADKAACLLPKLDELRDEGKASSAKQRLKCASLQKFGERAFKAWAV
ARLSQRFPKAEFAEVSKLVTDLTKVHTECCHGDLLECADDRADLAKYICENQDSISSKLKE
CCEKPLLEKSHCIAEVENDEMPADLPSLAADFVESKDVCKNYAEAKDVFLGMFLYEYARR
HPDYSVVLLLRLAKTYETTLEKCCAAADPHECYAKVFDEFKPLVEEPQNLIKQNCELFEQ
LGEYKFQNALLVRYTKKVPQVSTPTLVEVSRNLGKVGSKCCKHPEAKRMPCAEDYLSVV
LNQLCVLHEKTPVSDRVTKCCTESLVNRRPCFSALEVDETYVPKEFNAETFTFHADICTLS
EKERQIKKQTALVELVKHKPKATKEQLKAVMDDFAAFVEKCCKADDKETCFAEEGKKLV
AASQAALGL
>antioxidant_25
MVKAVTVLNSSEGPHGIVYFAQEGDGPTTV
>antioxidant_26

MSTIPFRKNYVFKNWAGIYSAKPERYFQPSSIDEVVELVKSARLAEKSLVTVGSGHSPSNM
CVTDEWLVNLDRLDKVQKFVEYPELHYADVTVDAGMRLYQLNEFLGAKGYSIQNLGSIS
EQSVAGIISTGSHGSSPYHGLISSQYVNLTIVNGKGELKFLDAENDPEVFKAALLSVGKIGII
VSATIRVVPGFNIKSTQEVITFENLLKQWDTLWTSSEFIRVWWYPYTRKCVLWRGNKTTD
AQNGPAKSWWGTKLGRFFYETLLWISTKIYAPLTPFVEKFVFNRQYGKLEKSSTGDVNVT
DSISGFNMDCLFSQFVDEWGCPMDNGLEVLRSLDHSIAQAAINKEFYVHVPMEVRCSNTT
LPSEPLDTSKRTNTSPGPVYGNVCRPFLDNTPSHCRFAPLENVTNSQLTLYINATIYRPFGC
NTPIHKWFTLFENTMMVAGGKPHWAKNFLGSTTLAAGPVKKDTDYDDFEMRGMALKVE
EWYGEDLKKFRKIRKEQDPDNVFLANKQWAIINGIIDPSELSD
>antioxidant_27
MFLKAVVLTLALVAVAGARAEVSADQVATVMWDYFSQLSNNAKEAVEHLQKSELTQQLN
ALFQDKLGEVNTYAGDLQKKLVPFATELHERLAKDSEKLKEEIGKELEELRARLLPHANE
VSQKIGDNLRELQQRLEPYADQLRTQVNTQAEQLRRQLTPYAQRMERVLRENADSLQASL
RPHADELKAKIDQNVEELKGRLTPYADEFKVKIDQTVEELRRSLAPYAQDTQEKLNHQLE
GLTFQMKKNAEELKARISASAEELRQRLAPLAEDVRGNLRGNTEGLQKSLAELGGHLDQ
QVEEFRRRVEPYGENFNKALVQQMEQLRQKLGPHAGDVEGHLSFLEKDLRDKVNSFFST
FKEKESQDKTLSLPELEQQQEQQQEQQQEQVQMLAPLES
>antioxidant_28
MKVLWAALLVTFLAGCQAKVEQAVETEPEPELRQQTEWQSGQRWELALGRFWDYLRWV
QTLSEQVQEELLSSQVTQELRALMDETMKELKAYKSELEEQLTPVAEETRARLSKELQAA
QARLGADMEDVCGRLVQYRGEVQAMLGQSTEELRVRLASHLRKLRKRLLRDADDLQKR
LAVYQAGAREGAERGLSAIRERLGPLVEQGRVRAATVGSLAGQPLQERAQAWGERLRAR
MEEMGSRTRDRLDEVKEQVAEVRAKLEEQAQQIRLQAEAFQARLKSWFEPLVEDMQRQ
WAGLVEKVQAAVGTSAAPVPSDNH
>antioxidant_29
MFHQVWAALLSLYGLLFNSMNQCPEHSQLTALGMDDTETPEPHLGLWYFIAGAASTTEEL
ATFDPVDNIVFNMAAGSAPRQLQLRATIRTKSGVCVPRKWTYRLTEGKGNMELRTEGRPD
MKTDLFSSSCPGGIMLKETGQGYQRFLLYNRSPHPPEKCVEEFQSLTSCLDFKAFLVTPRN
QEACPLSSK
>antioxidant_30
MAAPIVDAEYLKEITKARRELRSLIANKNCAPIMLRLAWHDAGTYDAQSKTGGPNGSIRN
EEEHTHGANSGLKIALDLCEGVKAKHPKITYADLYQLAGVVAVEVTGGPDIVFVPGRKDS
NVCPKEGRLPDAKQGFQHLRDVFYRMGLSDKDIVALSGGHTLGRAHPERSGFDGPWTQE
PLKFDNSYFVELLKGESEGLLKLPTDKTLLEDPEFRRLVELYAKDEDAFFRDYAESHKKLS
ELGFNPNSSAGKAVADSTILAQSAFGVAVAAAVVAFGYFYEIRKRMK
>antioxidant_31
MAERVSLTLNGTLLSPPPTTTTTTMSSSLRSTTAASLLLRSSSSSSRSTLTLSASSSLSFVRSL
VSSPRLSSSSSLSQKKYRIASVNRSFNSTTAATKSSSSDPDQLKNAREDIKELLSTKFCHPIL
VRLGWHDAGTYNKNIKEWPQRGGANGSLRFDIELKHAANAGLVNALNLIKDIKEKYSGI
SYADLFQLASATAIEEAGGPKIPMKYGRVDASGPEDCPEEGRLPDAGPPSPATHLREVFYR
MGLDDKDIVALSGAHTLGRSRPERSGWGKPETKYTKEGPGAPGGQSWTPEWLKFDNSYF
KEIKEKRDEDLLVLPTDAAIFEDSSFKVYAEKYAADQDAFFKDYAVAHAKLSNLGAEFNPP
EGIVI
>antioxidant_32

MGESERSEAFGIPRDSPLSSGDAAELEQLRREAAVLREQLENAVGSHAPTRSARDIHQLEA
RIDSLAARNSKLMETLKEARQQLLALREEVDRLGQPPSGYGVLLATHDDDTVDVFTSGR
KMRLTCSPNIDAASLKKGQTVRLNEALTVVEAGTFEAVGEISTLREILADGHRALVVGHA
DEERVVWLADPLIAEDLPDGLPEALNDDTRPRKLRPGDSLLVDTKAGYAFERIPKAEVEDL
VLEEVPDVSYADIGGLSRQIEQIRDAVELPFLHKELYREYSLRPPKGVLLYGPPGCGKTLIA
KAVANSLAKKMAEVRGDDAHEAKSYFLNIKGPELLNKFVGETERHIRLIFQRAREKASEG
TPVIVFFDEMDSIFRTRGTGVSSDVETTVVPQLLSEIDGVEGLENVIVIGASNREDMIDPAIL
RPGRLDVKIKIERPDAEAAQDIYSKYLTEFLPVHADDLAEFDGDRSACIKAMIEKVVDRM
YAEIDDNRFLEVTYANGDKEVMYFKDFNSGAMIQNVVDRAKKNAIKSVLETGQPGLRIQ
HLLDSIVDEFAENEDLPNTTNPDDWARISGKKGERIVYIRTLVTGKSSSASRAIDTESNLGQ
YL
>antioxidant_33
MSQARPATVLGAMEMGRRMDVTSSSASVRAFLQRGHTEIDTAFVYANGQSETILGDLGL
GLGRSGCKVKIATKAAPMFGKTLKPADVRFQLETSLKRLQCPRVDLFYLHFPDHGTPIEET
LQACHQLHQEGKFVELGLSNYVSWEVAEICTLCKKNGWIMPTVYQGMYNAITRQVETEL
FPCLRHFGLRFYAFNPLAGGLLTGRYKYQDKDGKNPESRFFGNPFSQLYMDRYWKEEHFN
GIALVEKALKTTYGPTAPSMISAAVRWMYHHSQLKGTQGDAVILGMSSLEQLEQNLALVE
EGPLEPAVVDAFDQAWNLVAHECPNYFR
>antioxidant_34
MEDSHKSTTSETAPQPGSAVQGAHISHIAQQVSSLSESEESQDSSDSIGSSQKAHGILARRPS
YRKILKDLSSEDTRGRKGDGENSGVSAAVTSMSVPTPIYQTSSGQYIAIAPNGALQLASPG
TDGVQGLQTLTMTNSGSTQQGTTILQYAQTSDGQQILVPSNQVVVQTASGDMQTYQIRTT
PSATSLPQTVVMTSPVTLTSQTTKTDDPQLKREIRLMKNREAARECRRKKKEYVKCLENR
VAVLENQNKTLIEELKTLKDLYSNKSV
>antioxidant_35
MPKHEFSVDMTCEGCSNAVTRVLNKLGGVQFDIDLPNKKVCINSEHSVDTLLETLGKTGK
AVSYLGPK
>antioxidant_36
MTYSFFVDMTCGGCSKAVNAILSKIDGVSNIQIDLENKKVSCESSKMGADELLKNIQKTG
KKCSIIA
>antioxidant_37
MSMASIASSSSTTLLSSSRVLLPSKSSLLSPTVSFPRIIPSSSASSSSLCSGFSSLGSLTTNRSAS
RRNFAVKAQADDLPLVGNKAPDFEAEAVFDQEFIKVKLSEYIGKKYVILFFYPLDFTFVCP
TEITAFSDRYEEFEKLNTEVLGVSVDSVFSHLAWVQTDRKSGGLGDLNYPLVSDITKSISKS
FGVLIPDQGIALRGLFIIDKEGVIQHSTINNLGIGRSVDETMRTLQALQYVQENPDEVCPAG
WKPGEKSMKPDPKLSKEYFSAI
>antioxidant_38
GLFIIDKEGVIQHSTINNEGVIQHSTINNLAIGRFGVLLADQGLALRSIPNGPSAL
>antioxidant_39
MNPLKAGDIAPKFSLPDQDGEQVNLTDFQGQRVLVYFYPKAMTPGCTVQACGLRDNMD
ELKKAGVDVLGISTDKPEKLSRFAEKELLNFTLLSDEDHQVCEQFGVWGEKSFMGKTYD
GIHRISFLIDADGKIEHVFDDFKTSNHHDVVLNWLKEHA
>antioxidant_40
MEIDQNLFQFPISTRDAVHEKNCTLRVKSTKKRRSSTKDEETRGMHPHIKSSFRNGMNHA

RVIREEDMEVVFEPCFINLSKPVYVVNGIGGINEIHKLPILFSSFVLCFFGNVSGDYGYVDN
VPLHISVISHFYPCLQSYNTDVIGITTDTVENICQWKAHLPRALQFSLPVISDSNNEICREM
GMLHPLGGAKLALDAIVIIDSIGRRRDILPIRTTTCVSTLITAVQETVRFLAIENGRLL
>antioxidant_41
MSATESSSIFTLSHNSNLQDILAANAKWASQMNNIQPTLFPDHNAKGQSPHTLFIGCSDSR
YNENCLGVLPGEVFTWKNVANICHSEDLTLKATLEFAIICLKVNKVIICGHTDCGGIKTCLT
NQREALPKVNCSHLYKYLDDIDTMYHEESQNLIHLKTQREKSHYLSHCNVKRQFNRIIEN
PTVQTAVQNGELQVYGLLYNVEDGLLQTVSTYTKVTPK
>antioxidant_42
MKLLILTCLVAVALARPKHPIKHQGLPQEVLNENLLRFFVAPFPEVFGKEKVNELSKDIGSE
STEDQAMEDIKQMEAESISSSEEIVPNSVEQKHIQKEDVPSERYLGYLEQLLRLKKYKVPQ
LEIVPNSAEERLHSMKEGIHAQQKEPMIGVNQELAYFYPELFRQFYQLDAYPSGAWYYVP
LGTQYTDAPSFSDIPNPIGSENSEKTTMPLW
>antioxidant_43
MKVLILACLVALALARELEELNVPGEIVESLSSSEESITRINKKIEKFQSEEQQQTEDELQDK
IHPFAQTQSLVYPFPGPIPNSLPQNIPPLTQTPVVVPPFLQPEVMGVSKVKEAMAPKHKEMP
FPKYPVEPFTESQSLTLTDVENLHLPLPLLQSWMHQPHQPLPPTVMFPPQSVLSLSQSKVLP
VPQKAVPYPQRDMPIQAFLLYQEPVLGPVRGPFPIIV
>antioxidant_44
MDPYKHRPSSGSNSSFWTTNSGAPVWNNNSALTVGQRGPILLEDYHLIEKLAQFDRERIPE
RVVHARGASAKGFFEVTHDVSHLTCADFLRAPGVQTPVIVRFSTVVHERGSPETLRDPRG
FAVKFYTREGNFDLVGNNMPVFFIRDGMKFPDMVHAFKPNPKTNLQENWRIVDFFSHHPE
SLHMFTFLFDDVGIPLNYRHMEGFGVNTYSLINRDGKPHLVKFHWKPTCGVKCLLDNEA
VTVGGTCHSHATKDLYDSIAAGNYPEWKLYIQTIDLDHEDKFDFDPLDVTKTWPEDIIPLQ
PVGRMVLNKNVDNFFAENEQIAFCPAISVPAIHYSDDKLLQTRIFSYADTQRHRLGPNYLM
LPVNAPKCAHHNNHHDGFMNFMHRDEEVNYFPSRFDPARHAEKVPIPPRVLTRCREKCII
QKENNFKQAGERYRSFDPARQDRFIQRWVDALTHPRVTHEHRTIWISYWSQCDAALGQK
LPSRLNLKPSM
>antioxidant_45
MADSRDPASDQMKHWKEERAAQKPDVLTTAGGNPVGDKLNIMTVGPRGPLLVQDVVFT
DEMAHFDRERIPERVVHAKGAGAFAYFEVTHDITKYCKAKVFEHIGKKTPIAVRFSTVAGE
SGSADTVRDPRGFAVKFYTEEGIWDLVGNNTPIFFIRDALLFPSFIHSQKRNPQTHLKDPDM
MWDFWSLRPESLHQVSFLFSDRGIPDGHRHMNGYGSHTFKLVNGSGEAVYCKFHYKTDQ
GIKNLSVEDAARLSQEDPDYGLRDLFNAIATGNYPSWTLYIQVMTFQQAQSFPFNPFDLTK
IWPHKDYPLIPVGKLVLNRNPVNYFAEVEQIAFDPSNMPPGIEPSPDKMLQGRLFAYPDTH
RHRLGPNYLQIPVNCPYRTRVANYQRDGPMCVTDNQGGAPNYYPNSFSAPVEQRQALEH
TSRCSGDVGRYNSTDDDNVTQVRAFYTQVLNEEQRRRLCENIAGHLKDAQLFIQKKAVK
NFMDVHPDYGNRIQTLLDKYNVEKPKNAIHTFVQDGSHLSAKEKANL
>antioxidant_46
MRLSLWNLLGLSGLVVASCPYMSGEDQEYHISHIQARGTDSEFLDQFKVEDSNSYLTTDA
GGPIQDDASLKAGERGPTLLEDFIFRQKIQHFDHERVPERAVHARGAGAYGTFTSYADWT
NITAASFLNSKGKETPVFVRFSTVAGSRGSADTVRDVHGFATRFYTDEGNFDIVGNNIPVFF
IQDAILFPDLVHAVKPSPDSEIPQAATGHDSAWDFFSQQPSTLHTLFWAMSGHGIPRSYRH
MDGFGVHTMRLVTDDGKSKLVKWHWKTKQGKASLVWEEAQILAGKNPDFHRQDLWD

DINAGNGPEWELGVQIVDEEDVQAFGFDLLDPTKFLPEELVPVTILGKMKLTDNPTNYFAE
TEQVMFQPGHIVRGVDFSDDPLLQGRIYSYLDTQLNRNGGPNFEQLPVNRPRTKVHNNN
RDGAGQMFIHTNKAPYSPNSLSGGNPKQANQTKGRGFFTAPSRKVVGSLHRGTASSFADV
WSQPRMFYNSLIPSEQQFLVNAIRFEISQLKSDLIKKNTLMQLNRVSNDLATRVAAVIGYKP
LDPSPEFYTNATTDYVTIFGKPLPSVVGFTVGILASTSSSTSISQAAQLATSFSSRGIRAVIVG
ESLLSGTDQTYSSADATAFDAVVVTMGAETLFGPVAKPNTLFPSGRPSQILHDAYRWGKP
VGAVSKASVVLEPLPGTKNQGGVYRVESVNELATSIAKGLETFRFVDRFPLDS
>antioxidant_47
MSFRAPNLIRSTVGRRAGQTLNLRSQVIRRRFATEGGPEITKPSAPRSSNTGYIFAGLGVAA
VGAAYYFYGTGRTEHDSTNKADTVVREAVATVEAKTGLRRGKDEYQKVYNRIAETLDKE
GYDDGSLAPVLLRLAWHASGTYSKADGTGGSNFATMRFKPEAEHSANNGLHVAREHME
KIKQEFPWISYGDLWTLGGVCAIQESGGPTIPWRPGRIDGYAAQVTPDGRLPDATQAQDH
LRFIFNRMGFNDQEIVALSGAHAMGRCHPNRSGFDGPWTFSPVTFSNQYFALLRDEPWQ
WKKWTGPAQFEDKKTKTLMMLPTDMALVKDKSFKKYVDIYADNEEKFFSDFAKAFSKLI
ELGVPERQWAGEPWTMATSD
>antioxidant_48
MKILILGIFLFLCSTPAWAKEKHYYIGIIETTWDYASDHGEKKLISVDTEHSNIYLQNGPDRI
GRLYKKALYLQYTDETFRTTIEKPVWLGFLGPIIKAETGDKVYVHLKNLASRPYTFHSHGI
TYYKEHEGAIYPDNTTDFQRADDKVYPGEQYTYMLLATEEQSPGEGDGNCVTRIYHSHID
APKDIASGLIGPLIICKKDSLDKEKEKHIDREFVVMFSVVDENFSWYLEDNIKTYCSEPEKV
DKDNEDFQESNRMYSVNGYTFGSLPGLSMCAEDRVKWYLFGMGNEVDVHAAFFHGQA
LTNKNYRIDTINLFPATLFDAYMVAQNPGEWMLSCQNLNHLKAGLQAFFQVQECNKSSSK
DNIRGKHVRHYYIAAEEIIWNYAPSGIDIFTKENLTAPGSDSAVFFEQGTTRIGGSYKKLVY
REYTDASFTNRKERGPEEEHLGILGPVIWAEVGDTIRVTFHNKGAYPLSIEPIGVRFNKNNE
GTYYSPNYNPQSRSVPPSASHVAPTETFTYEWTVPKEVGPTNADPVCLAKMYYSAVDPTK
DIFTGLIGPMKICKKGSLHANGRQKDVDKEFYLFPTVFDENESLLLEDNIRMFTTAPDQVD
KEDEDFQESNKMHSMNGFMYGNQPGLTMCKGDSVVWYLFSAGNEADVHGIYFSGNTYL
WRGERRDTANLFPQTSLTLHMWPDTEGTFNVECLTTDHYTGGMKQKYTVNQCRRQSED
STFYLGERTYYIAAVEVEWDYSPQREWEKELHHLQEQNVSNAFLDKGEFYIGSKYKKVV
YRQYTDSTFRVPVERKAEEEHLGILGPQLHADVGDKVKIIFKNMATRPYSIHAHGVQTESS
TVTPTLPGETLTYVWKIPERSGAGTEDSACIPWAYYSTVDQVKDLYSGLIGPLIVCRRPYLK
VFNPRRKLEFALLFLVFDENESWYLDDNIKTYSDHPEKVNKDDEEFIESNKMHAINGRMF
GNLQGLTMHVGDEVNWYLMGMGNEIDLHTVHFHGHSFQYKHRGVYSSDVFDIFPGTYQ
TLEMFPRTPGIWLLHCHVTDHIHAGMETTYTVLQNEDTKSG
>antioxidant_49
MEMALLPQPSPARYFLRTPSWSAVAIFQAVKIKPLQLRTNSSNSVTPNLISPSKKSWKDLFS
KRWQYYAEISRAGSPTGTYLLYSPCTWSILMAAYAYDSSLVNVTKMLALFGVGSFLMRSA
GCVINDLWDRELDAKVERSKSRPLASGKLSVRQAISLLSVQLTASLGILLQLNPYTIKLGVA
SLVPVCIYPAMKRITYYPQVVLGLTFGYGAVMGWPALAGEACMNWSVVAPLYLSTISWIV
LYDTIYAHQDKRDDVKANIYSTALRFGDNTKPVLCGLAALQIATLATAGIMNGQGPVFYT
LGVAGAAYRLSSMIYKVDLDDPKDCFRWFKRNSNTGYLVAAAIALDWLAKSFIYDS
>antioxidant_50
MSCNQQKECCKKECQEKECCKECCCPRIKAFKKFINTFEKAQIGKEAPEFKAPAYCPCGSI
KEIDINEYRGKYVVLLFYPLDWTFVCPTEMIGYSELAGQLKEINCEVIGVSVDSVYCHQA

WCEADKSKGGVGKLTFPLVSDIKRCISIKYGMLNVEAGIARRGYVIIDDKGKVRYIQMND
DGIGRSTEETIRIVKAIQFSDEHGAVCPLNWKPGKDTIEPTPDGIKKYLTAH
>antioxidant_51
MGDVEKGKKIFIMKCSQCHTVEKGGKHKTGPNLHGLFGRKTGQAPGYSYTAANKNKGII
WGEDTLMEYLENPKKYIPGTKMIFVGIKKKEERADLIAYLKKATNE
>antioxidant_52
MHSLSERVEQLRLNDCPDWLYLLHEFDALYRQGSDGGSRPIRTHRKRVRDSLALIVEANPA
VNDRPPEVKPVTAHLGRALDLGERGAVQGMSRALARVAGRLTWEYGYEKVPKALARKY
AYCEILGPRGPICAERLILGFVLFAPSTTYPQHSHKDIEESYISVAGAWSENDAAVHAPGSLI
LNRPGLEHRITTGDLSPCLLAYAWTGSEERLNQPGMKLSSPRKARIEKGI
>antioxidant_53
MALEICVKAAVGAPDHLGDCPFSQRALLTLEEKSLTYKIHLINLSDKPQWFLDISPQGKVP
VLKIDDKWVTDSDVIVGILEEKYPDPPLKTPAEFASVGSNIFGTFGTFLKSKDSNDGSEHAL
LVELEALENHLKSHDGPFIAGERVSAVDLSLAPKLYHLQVALGHFKSWSVPESFPHVHNY
MKTLFSLDSFEKTKTEEKYVISGWAPKVNP
>antioxidant_54
MVESRRAAAAASAYASRCGIAPATSQRSLATPPTISVPSGEGRCRCHVARGAGRDPRRRLR
RRRWCGRCGYHSHLTGGEFDVNRLCQQRSRERSCQLVAVPADPRPKRQRITDVLTLALVG
FLGGLITGISPCILPVLPVIFFSGAQSVDAAQVAKPEGAVAVRRKRALSATLRPYRVIGGLVL
SFGMVTLLGSALLSVLHLPQDAIRWAALVALVAIGAGLIFPRFEQLLEKPFSRIPQKQIVTRS
NGFGLGLALGVLYVPCAGPILAAIVVAGATATIGLGTVVLTATFALGAALPLLFFALAGQRI
AERVGAFRRRQREIRIATGSVTILLAVALVFDLPAALQRAIPDYTASLQQQISTGTEIREQLN
LGGIVNAQNAQLSNCSDGAAQLESCGTAPDLKGITGWLNTPGNKPIDLKSLRGKVVLIDF
WAYSCINCQRAIPHVVGWYQAYKDSGLAVIGVHTPEYAFEKVPGNVAKGAANLGISYPIA
LDNNYATWTNYRNRYWPAEYLIDATGTVRHIKFGEGDYNVTETLVRQLLNDAKPGVKLP
QPSSTTTPDLTPRAALTPETYFGVGKVVNYGGGGAYDEGSAVFDYPPSLAANSFALRGRW
ALDYQGATSDGNDAAIKLNYHAKDVYIVVGGTGTLTVVRDGKPATLPISGPPTTHQVVAG
DRLASETLEVRPSKGLQVFSFTYG
>antioxidant_55
MTHYDVVVLGAGPGGYVAAIRAAQLGLSTAIVEPKYWGGVCLNVGCIPSKALLRNAELV
HIFTKDAKAFGISGEVTFDYGIAYDRSRKVAEGRVAGVHFLMKKNKITEIHGYGTFADANT
LLVDLNDGGTESVTFDNAIIATGSSTRLVPGTSLSANVVTYEEQILSRELPKSIIIAGAGAIG
MEFGYVLKNYGVDVTIVEFLPRALPNEDADVSKEIEKQFKKLGVTILTATKVESIADGGSQ
VTVTVTKDGVAQELKAEKVLQAIGFAPNVEGYGLDKAGVALTDRKAIGVDDYMRTNVG
HIYAIGDVNGLLQLAHVAEAQGVVAAETIAGAETLTLGDHRMLPRATFCQPNVASFGLTEQ
QARNEGYDVVVAKFPFTANAKAHGVGDPSGFVKLVADAKHGELLGGHLVGHDVAELLPE
LTLAQRWDLTASELARNVHTHPTMSEALQECFHGLVGHMINF
>antioxidant_56
MSFPFASLLKRPSAISSLLSLKKPGSWSSILLKAVGVLSRDSRWHSDLLKMLTEEMDSLNG
QINTWTDNNPLLDEITKPYRKSSTRFFHPLLVLLMSRASVNGDPPSQQLFQRYKQLARVTE
LIHAANIIHINIGEEQSNEQIKLATLVGDYLLGKASVDLAHLENNAITEIMASVIANLVEGHF
GSRQNGSVGLSNERTILLQSAFMPAKACLCASILNNSSQYINDACFNYGKFLGLSLQLAHK
PVSPDAQVLQKNNDILKTYVENAKSSLSVFPDIEAKQALMEIANSVSK
>antioxidant_57

MKNFSIAKSRRLRSTPYTSRIEKQGVTAYTIYNHMLLPAAFGSIEDSYKHLKEHVQIWDVA
AERQVEISGKDSAELVQLMTCRDLSKSKIGRCYYCPIIDENGNLVNDPVVLKLDENKWWI
SIADSDVIFFAKGLASGHKFDVKIVEPVVDIMAIQGPKSFALMEKVFGKKITELKFFGFDYF
DFEGTKHLIARSGWSKQGGYEVYVENTQSGQKLYDHLFEVGKEFNVGPGCPNLIERIESA
LLSYGNDFDNNDNPFECGFDQYVSLDSDINFLGKEKLKEIKLKGPQKKLRGVKIDIKEISLT
GSKNIYDENNNVIGELRSACYSPHFQKVIGIAMIKKSHWEASQGFKIQINDNTINGNVCDL
PFI
>antioxidant_58
MASIFPSRRVRRTPFSAGVEAAGVKGYTVYNHMLLPTVFDSLQADCAHLKEHVQVWDV
ACERQVSIQGPDALRLMKLISPRDMDRMADDQCYYVPTVDHRGGMLNDPVAVKLAADH
YWLSLADGDLLQFGLGIAIARGFDVEIVEPDVSPLAVQGPRADDLMARVFGEAVRDIRFFR
YKRLAFQGVELVVARSGWSKQGGFEIYVEGSELGMPLWNALFAAGADLNVRAGCPNNIE
RVESGLLSYGNDMTRENTPYECGLGKFCNSPEDYIGKAALAEQAKNGPARQIRALVIGGEI
PPCQDAWPLLADGRQVGQVGSAIHSPEFGVNVAIGMVDRSHWAPGTGMEVETPDGMRP
VTVREGFWR
>antioxidant_59
MGEALRRSTRIAISKRMLEEEESKLAPISTPEVPKKKIKTGPKHNANQAVVQEANRSSDVN
ELEIGDPIPDLSLLNEDNDSISLKKITENNRVVVFFVYPRASTPGCTRQACGFRDNYQELKK
YAAVFGLSADSVTSQKKFQSKQNLPYHLLSDPKREFIGLLGAKKTPLSGSIRSHFIFVDGKL
KFKRVKISPEVSVNDAKKEVLEVAEKFKEE
>antioxidant_60
MIQYVYLKHMRKLWSLGKVRSTVLRFSTTNRNASHLIKNELEQISPGIRQMLNSNSEFLEE
CSKYYTIAQGKQMRPSLVLLMSKATSLCHGIDRSVVGDKYIDDDDLRSFSTGQILPSQLRL
AQITEMIHIASLLHDDVIDHANVRRGSPSSNVAFGNRRSILAGNFILARASTAMARLRNPQ
VTELLATVIADLVRGEFLQLKNTMDPSSLEIKQSNFDYYIEKSFLKTASLISKSCKASTILGQ
CSPTVATAAGEYGRCIGTAFQLMDDVLDYTSKDDTLGKAAGADLKLGLATAPVLFAWKK
YPELGAMIVNRFNHPSDIQRARSLVECTDAIEQTITWAKEYIKKAKDSLLCLPDSPARKALF
ALADKVITRKK
>antioxidant_61
MQEKPQEPKVVGVEILEKSGLDIKKLVDKLVKATAAEFTTYYYYTILRMHLTGMEGEGLK
EIAEDARLEDRLHFELMTQRIYELGGGLPRDIRQLADISACSDAYLPENWKDPKEILKVLL
EAEQCAIRTWKEVCDMTYGKDPRTYDLAQRILQEEIEHEAWFLELLYGRPSGHFRRSSPG
NAPYSKK
>antioxidant_62
MSFLQDPSFFTMGMWSIGAGALGAAALALLLANTDVFLSKPQKAALEYLEDIDLKTLEK
EPRTFKAKELWEKNGAVIMAVRRPGCFLCREEAADLSSLKSMLDQLGVPLYAVVKEHIRTE
VKDFQPYFKGEIFLDEKKKFYGPQRRKMMFMGFIRLGVWYNFFRAWNGGFSGNLEGEGF
ILGGVFVVGSGKQGILLEHREKEFGDKVNLLSVLEAAKMIKPQTLASEKK
>antioxidant_63
MSFSGKYQLQSQENFEAFMKAIGLPEELIQKGKDIKGVSEIVQNGKHFKFTITAGSKVIQN
EFTVGEECELETMTGEKVKTVVQLEGDNKLVTTFKNIKSVTELNGDIITNTMTLGDIVFKR
ISKRI
>antioxidant_64
MAELKLGYKASAEQFAPRELVELAVLAESAGMDSATVSDHFQPWRHEGGHAPFSLAWMT

AVGERTKNLVLGTSVLTPTFRYNPAVIAQAFATMGCLYPGRIFLGVGTGEALNEIATGYAGE
WPEFKERFARLRESVRLMRELWLGDRVDFDGEYYRTKGASIYDVPEGGIPVYIAAGGPVV
AKYAGRAGDGFICTSGKGEELYAEKLIPAVKEGAAAADRDADAIDRMIEIKISYDTDPELA
LENTRFWAPLSLTAEQKHSIDDPIEMEKAADALPIEQVAKRWIVASDPDEAVEKVGQYVK
WGLNHLVFHAPGHDQRRFLELFKRDLEPRLRKLA
>antioxidant_65
MASSLNLGSPAPPIKVQNWLRGDPLSNFQLGKIYVVEFFSIYCGYCAPELSDLAKLHKKFI
DTRVEFIGIAASEEAATADDARAQVDASITKSLPNTNIRMGFDHSGEMDEDWLKASLSFH
VPKTFVVDRDGSIAFIGDLVMLQDVLPKVIDGNWRASGKQRMPKRSGLLKARLMLRRLF
HDRVSAAIEIKNWKAPLSAIEEGINLNPDSIFLRRNLYWHE
>antioxidant_66
MVKHQPLQYYEPQLCLSCLTGIYGCRWKRYQRSHDDTTKWERLWFLILTSSFFLTLVWFY
FWWEVHNDYNEINWFLYNRMGYWSDWSIPILVTTAAGFTYITVLLILALCHIAVGQQMNL
HWLHKIGLMTTLITTVVTMSSIAQLWDDEWEMVFISLQATAPFLHIGALAAVTALSWLIAG
QFARMEKATSQMLMVTAYLAVVVALYLVPLTISSPCIMEKKALGPKPAIIGHRGAPMLAPE
NTLMSFQKAVEQKIYGVQADVILSYDGVPFLMHDKTLRRTTNVEEVFPGRAYEHSSMFN
WTDLEMLNAGEWFLRNDPFWTAGSLSRSDYLEAANQSVCKLADMLEVIKDNTSLILNFQ
DLPPDHPYYTSYINITLKTILASGIQQQAVMWLPDTERQLVRQIAPAFQQTSGLKLDAERLR
EKGIVKLNLRYTKVTNEDVRDYMAANLSVNLYTVNEPWLYSILWCTGVPSVTSDSSHVL
RKVPFPIWLMPPDEYRLIWITSDLISFIIIVGVFIFQNYHNDQWRLGSIRTYNPEQIMLSAAV
RRSSRDVKIMKEKLIFSEINNGVETTDELSLCSENGYANEMVTPTDHRDTRLRMN
>antioxidant_67
MKKKLVVLGLLAVVLVLVIVGLCLWLPSASKEPDNHVYTRAAVAADAKQCSKIGRDALR
DGGSAVDAAIAALLCVGLMNAHSMGIGGGLFLTIYNSTTRKAEVINAREVAPRLAFATMF
NSSEQSQKGGLSVAVPGEIRGYELAHQRHGRLPWARLFQPSIQLARQGFPVGKGLAAALE
NKRTVIEQQPVLCEVFCRDRKVLREGERLTLPQLADTYETLAIEGAQAFYNGSLTAQIVKD
IQAAGGIVTAEDLNNYRAELIEHPLNISLGDVVLYMPSAPLSGPVLALILNILKGYNFSRES
VESPEQKGLTYHRIVEAFRFAYAKRTLLGDPKFVDVTEVVRNMTSEFFAAQLRAQISDDTT
HPISYYKPEFYTPDDGGTAHLSVVAEDGSAVSATSTINLYFGSKVRSPVSGILFNNEMDDFS
SPSITNEFGVPPSPANFIQPGKQPLSSMCPTIMVGQDGQVRMVVGAAGGTQITTATALAIIY
NLWFGYDVKRAVEEPRLHNQLLPNVTTVERNIDQAVTAALETRHHHTQIASTFIAVVQAIV
RTAGGWAAASDSRKGGEPAGY
>antioxidant_68
MSWRRAASVGRRLVASGRILAGRRGAAGAAGSGMGNSTSSFWGKSTTTPVNQIQETISN
NCVVIFSKTSCSYCSMAKKIFHDMNVNYKAVELDMLEYGNQFQDALHKMTGERTVPRIF
VNGRFIGGAADTHRLHKEGKLLPLVHQCYLKKKQEERH
>antioxidant_69
MRSMKALQKALSRAGSHCGRGGWGHPSRSPLLGGGVRHHLSEAAAQGRETPHSHQPQH
QDHDSSESGMLSRLGDLLFYTIAEGQERIPIHKFTTALKATGLQTSDPRLRDCMSEMHRVV
QESSSGGLLDRDLFRKCVSSNIVLLTQAFRKKFVIPDFEEFTGHVDRIFEDVKELTGGKVAA
YIPQLAKSNPDLWGVSLCTVDGQRHSVGHTKIPFCLQSCVKPLTYAISISTLGTDYVHKFV
GKEPSGLRYNKLSLNEEGIPHNPMVNAGAIVVSSLIKMDCNKAEKFDFVLQYLNKMAGN
EYMGFSNATFQSEKETGDRNYAIGYYLKEKKCFPKGVDMMAALDLYFQLCSVEVTCESG
SVMAATLANGGICPITGESVLSAEAVRNTLSLMHSCGMYDFSGQFAFHVGLPAKSAVSGAI

LLVVPNVMGMMCLSPPLDKLGNSHRGTSFCQKLVSLFNFHNYDNLRHCARKLDPRREGA
EIRNKTVVNLLFAAYSGDVSALRRFALSAMDMEQKDYDSRTALHVAAAEGHIEVVKFLIE
ACKVNPFAKDRWGNIPLDDAVQFNHLEVVKLLQDYQDSYTLSETQAEAAAEALSKENLE
SMV
>antioxidant_70
SALYALYDFSPPARKMRAYTVRAYVHGSYSRRGPWYDFEPVPGASMDGL
>antioxidant_71
MKALILVGGFGTRLRPLTLSFPKPLVDFANKPMILHQIEALKAVGVDEVVLAINYQPEVML
NFLKDFETKLEIKITCSQETEPLGTAGPLALARDKLLDGSGEPFFVLNSDVISEYPLKEMLE
FHKSHGGEASIMVTKVDEPSKYGVVVMEESTGRVEKFVEKPKLYVGNKINAGIYLLNPSV
LDKIELRPTSIEKETFPKIAAAQGLYAMVLPGFWMDIGQPRDYITGLRLYLDSLRKKSPAKL
TSGPHIVGNVLVDETATIGEGCLIGPDVAIGPGCIVESGVRLSRCTVMRGVRIKKHACISSSII
GWHSTVGQWARIENMTILGEDVHVSDEIYSNGGVVLPHKEIKSNILKPEIVM
>antioxidant_72
MSEFYKLAPVDKKGQPFPFDQLKGKVVLIVNVASKCGFTPQYKELEALYKRYKDEGFTII
GFPCNQFGHQEPGSDEEIAQFCQLNYGVTFPIMKKIDVNGGNEDPVYKFLKSQKSGMLGL
RGIKWNFEKFLVDKKGKVYERYSSLTKPSSLSETIEELLKEVE
>antioxidant_73
MAASLSSRLIKGIANLKAVRSSRLTSASVYQNGMMRFSSTVPSDSDTHDDFKPTQKVPPDS
TDSLKDIVENDVKDNPVMIYMKGVPESPQCGFSSLAVRVLQQYNVPISSRNILEDQELKNA
VKSFSHWPTFPQIFIKGEFIGGSDIILNMHKEGELEQKLKDVSGNQD
>antioxidant_74
MALLSQAGGSYTVVPSGVCSKAGTKAVVSGGVRNLDVLRMKEAFGSSYSRSLSTKSMLL
HSVKRSKRGHQLIVAASPPTEEAVVATEPLTREDLIAYLASGCKTKDKYRIGTEHEKFGFEV
NTLRPMKYDQIAELLNGIAERFEWEKVMEGDKIIGLKQGKQSISLEPGGQFELSGAPLETL
HQTCAEVNSHLYQVKAVAEEMGIGFLGIGFQPKWRREDIPIMPKGRYDIMRNYMPKVGTL
GLDMMLRTCTVQVNLDFSSEADMIRKFRAGLALQPIATALFANSPFTEGKPNGFLSMRSHI
WTDTDKDRTGMLPFVFDDSFGFEQYVDYALDVPMYFAYRKNKYIDCTGMTFRQFLAGK
LPCLPGELPSYNDWENHLTTIFPEVRLKRYLEMRGADGGPWRRLCALPAFWVGLLYDDD
SLQAILDLTADWTPAEREMLRNKVPVTGLKTPFRDGLLKHVAEDVLKLAKDGLERRGYK
EAGFLNAVDEVVRTGVTPAEKLLEMYNGEWGQSVDPVFEELLY
>antioxidant_75
MGLLSQGSPLSWEETKRHADHVRRHGILQFLHIYHAVKDRHKDVLKWGDEVEYMLVSF
DHENKKVRLVLSGEKVLETLQEKGERTNPNHPTLWRPEYGSYMIEGTPGQPYGGTMSEF
NTVEANMRKRRKEATSILEENQALCTITSFPRLGCPGFTLPEVKPNPVEGGASKSLFFPDEA
INKHPRFSTLTRNIRHRRGEKVVINVPIFKDKNTPSPFIETFTEDDEASRASKPDHIYMDAM
GFGMGNCCLQVTFQACSISEARYLYDQLATICPIVMALSAASPFYRGYVSDIDCRWGVISA
SVDDRTREERGLEPLKNNNYRISKSRYDSIDSYLSKCGEKYNDIDLTIDKEIYEQLLQEGID
HLLAQHVAHLFIRDPLTLFEEKIHLDDANESDHFENIQSTNWQTMRFKPPPPNSDIGWRVE
FRPMEVQLTDFENSAYVVFVVLLTRVILSYKLDFLIPLSKVDENMKVAQKRDAVLQGMFY
FRKDICKGGNAVVDGCGKAQNSTELAAEEYTLMSIDTIINGKEGVFPGLIPILNSYLENME
VDVDTRCSILNYLKLIKKRASGELMTVARWMREFIANHPDYKQDSVITDEMNYSLILKCN
QIANELCECPELLGSAFRKVKYSGSKTDSSN
>antioxidant_76

MPGKPVLHYFDGRGRMEPIRWLLAAAGVEFEENFLKTRDDLARLRSDGSLMFEQVPMVE
IDGMKLVQTKAILNYIATKYNLYGKDMKERALIDMYAEGVADLELMVLYYPYMPPGEKE
ASLAKIKDKARNRYFPAYEKVLKSHGQDYLVGNKLSRADVSLVELLYHVEEMDPGIVDNF
PLLKALRTRVSNLPTVKKFLQPGSQRKPFDDEKCVESAKKIFS
>antioxidant_77
MPMILGYWDIRGLAHAIRLLLEYTDTNYEERQYSVGDAPDYDRSQWLNEKFKLGLDFPN
LPYLIDGTHKLTQSNAILRYIARKHNLCGETEEEMIRVDILENQVMDVRLAMARICYSPDF
EKLKPGFLKEIPEKIKLFSEFLGKRPWFAGDKLTYVDFLVYDVLDMHRIFEPKCLDAFPNL
KDFISRFEGLKKISAYMKSSRFLPGPLFMKLAVWGNK
>antioxidant_78
MAPVYEGMASHVQVFSPHTLQSSAFCSVKKLKIEPSSNWDMTGYGSHSKVYSQSKNIPLS
QPATTTVSTSLPVPNPSLPYEQTIVFPGSTGHIVVTSASSTSVTGQVLGGPHNLMRRSTVSL
LDTYQKCGLKRKSEEIENTSSVQIIEEHPPMIQNNASGATVATATTSTATSKNSGSNSEGDY
QLVQHEVLCSMTNTYEVLEFLGRGTFGQVVKCWKRGTNEIVAIKILKNHPSYARQGQIEV
SILARLSTESADDYNFVRAYECFQHKNHTCLVFEMLEQNLYDFLKQNKFSPLPLKYIRPVL
QQVATALMKLKSLGLIHADLKPENIMLVDPSRQPYRVKVIDFGSASHVSKAVCSTYLQSRY
YRAPEIILGLPFCEAIDMWSLGCVIAELFLGWPLYPGASEYDQIRYISQTQGLPAEYLLSAG
TKTTRFFNRDTDSPYPLWRLKTPDDHEAETGIKSKEARKYIFNCLDDMAQVNMTTDLEGS
DMLVEKADRREFIDLLKKMLTIDADKRITPIETLNHPFVTMTHLLDFPHSTHVKSCFQNME
ICKRRVNMYDTVNQSKTPFITHVAPSTSTNLTMTFNNQLTTVHNQAPSSTSATISLANPEVS
ILNYPSTLYQPSAASMAAVAQRSMPLQTGTAQICARPDPFQQALIVCPPGFQGLQASPSKH
AGYSVRMENAVPIVTQAPGAQPLQIQPGLLAQQAWPSGTQQILLPPAWQQLTGVATHTSV
QHATVIPETMAGTQQLADWRNTHAHGSHYNPIMQQPALLTGHVTLPAAQPLNVGVAHV
MRQQPTSTTSSRKSKQHQSSVRNVSTCEVSSSQAISSPQRSKRVKENTPPRCAMVHSSPAC
STSVTCGWGDVASSTTRERQRQTIVIPDTPSPTVSVITISSDTDEEEEQKHAPTSTVSKQRK
NVISCVTVHDSPYSDSSSNTSPYSVQQRAGHNNANAFDTKGSLENHCTGNPRTIIVPPLKT
QASEVLVECDSLVPVNTSHHSSSYKSKSSSNVTSTSGHSSGSSSGAITYRQQRPGPHFQQQ
QPLNLSQAQQHITTDRTGSHRRQQAYITPTMAQAPYSFPHNSPSHGTVHPHLAAAAAAAH
LPTQPHLYTYTAPAALGSTGTVAHLVASQGSARHTVQHTAYPASIVHQVPVSMGPRVLPSP
TIHPSQYPAQFAHQTYISASPASTVVYTGYPLSPAKVNQYPYI
>antioxidant_79
MESLLSSSSLVSAAGGFCWKKQNLKLHSLSEIRVLRCDSSKVVAKPKFRNNLVRPDGQGS
SLLLYPKHKSRFRVNATAGQPEAFDSNSKQKSFRDSLDAFYRFSRPHTVIGTVLSILSVSFL
AVEKVSDISPLLFTGILEAVVAALMMNIYIVGLNQLSDVEIDKVNKPYLPLASGEYSVNTGI
AIVASFSIMSFWLGWIVGSWPLFWALFVSFMLGTAYSINLPLLRWKRFALVAAMCILAVRAI
IVQIAFYLHIQTHVFGRPILFTRPLIFATAFMSFFSVVIALFKDIPDIEGDKIFGIRSFSVTLGQ
KRVFWTCVTLLQMAYAVAILVGATSPFIWSKVISVVGHVILATTLWARAKSVDLSSKTEITS
CYMFIWKLFYAEYLLLPFLK
>antioxidant_80
MSALGAVIALLLWGQLFAVDSGNDVTDIADDGCPKPPEIAHGYVEHSVRYQCKNYYKLRT
EGDGVYTLNDKKQWINKAVGDKLPECEADDGCPKPPEIAHGYVEHSVRYQCKNYYKLR
TEGDGVYTLNNEKQWINKAVGDKLPECEAVCGKPKNPANPVQRILGGHLDAKGSFPWQA
KMVSHHNLTTGATLINEQWLLTTAKNLFLNHSENATAKDIAPTLTLYVGKKQLVEIEKVVL
HPNYSQVDIGLIKLKQKVSVNERVMPICLPSKDYAEVGRVGYVSGWGRNANFKFTDHLK

YVMLPVADQDQCIRHYEGSTVPEKKTPKSPVGVQPILNEHTFCAGMSKYQEDTCYGDAG
SAFAVHDLEEDTWYATGILSFDKSCAVAEYGVYVKVTSIQDWVQKTIAEN
>antioxidant_81
MQPDPRPSGAGACCRFLPLQSQCPEGAGDAVMYASTECKAEVTPSQHGNRTFSYTLEDHT
KQAFGIMNELRLSQQLCDVTLQVKYQDAPAAQFMAHKVVLASSSPVFKAMFTNGLREQ
GMEVVSIEGIHPKVMERLIEFAYTASISMGEKCVLHVMNGAVMYQIDSVVRACSDFLVQQ
LDPSNAIGIANFAEQIGCVELHQRAREYIYMHFGEVAKQEEFFNLSHCQLVTLISRDDLNV
RCESEVFHACINWVKYDCEQRRFYVQALLRAVRCHSLTPNFLQMQLQKCEILQSDSRCKD
YLVKIFEELTLHKPTQVMPCRAPKVGRLIYTAGGYFRQSLSYLEAYNPSDGTWLRLADLQ
VPRSGLAGCVVGGLLYAVGGRNNSPDGNTDSSALDCYNPMTNQWSPCAPMSVPRNRIGV
GVIDGHIYAVGGSHGCIHHNSVERYEPERDEWHLVAPMLTRRIGVGVAVLNRLLYAVGGFD
GTNRLNSAECYYPERNEWRMITAMNTIRSGAGVCVLHNCIYAAGGYDGQDQLNSVERYD
VETETWTFVAPMKHRRSALGITVHQGRIYVLGGYDGHTFLDSVECYDPDTDTWSEVTRM
TSGRSGVGVAVTMEPCRKQIDQQNCTC
>antioxidant_82
MGNPENIEDAYVAVIRPKNTASLNSREYRAKSYEILLHEVPIEGQKKKRKKVLLETKLQGN
SEITQGILDYVVETTKPISPANQGIRGKRVVLMKKFPLDGEKMGREASLFIVPSVVKDNTK
YTYTPGCPIFYCLQDIMRVCSESSTHFATLTARMLIALDKWLDERHAQSHFIPALFRPSPLER
IKTNVINPAYATESGQTENSLHMGYSALEIKSKMLALEKADTCIYNPLFGSDLQYTNRVDK
VVINPYFGLGAPDYSKIQIPKQEKWQRSMSSVTEDKERQWVDDFPLHRSACEGDSELLSR
LLSERFSVNQLDSDHWAPIHYACWYGKVEATRILLEKGKCNPNLLNGQLSSPLHFAAGGG
HAEIVQILLNHPETDRHITDQQGRSPLNICEENKQNNWEEAAKLLKEAINKPYEKVRIYRM
DGSYRSVELKHGNNTTVQQIMEGMRLSQETQQYFTIWICSENLSLQLKPYHKPLQHVRD
WPEILAELTNLDPQRETPQLFLRRDVRLPLEVEKQIEDPLAILILFDEARYNLLKGFYTAPD
AKLITLASLLLQIVYGNYESKKHKQGFLNEENLKSIVPVTKLKSKAPHWTNRILHEYKNLS
TSEGVSKEMHHLQRMFLQNCWEIPTYGAAFFTGQIFTKASPSNHKVIPVYVGVNIKGLHL
LNMETKALLISLKYGCFMWQLGDTDTCFQIHSMENKMSFIVHTKQAGLVVKLLMKLNG
QLMPTERNS
>antioxidant_83
MANFQEHLSCSSSPHLPFSESKTFNGLQDELTAMGNHPSPKLLEDQQEKGMVRTELIESVH
SPVTTTVLTSVSEDSRDQFENSVLQLREHDESETAVSQGNSNTVDGESTSGTEDIKIQFSRS
GSGSGGFLEGLFGCLRPVWNIIGKAYSTDYKLQQQDTWEVPFEEISELQWLGSGAQGAVF
LGKFRAEEVAIKKVREQNETDIKHLRKLKHPNIIAFKGVCTQAPCYCIIMEYCAHGQLYEV
LRAGRKITPRLLVDWSTGIASGMNYLHLHKIIHRDLKSPNVLVTHTDAVKISDFGTSKELS
DKSTKMSFAGTVAWMAPEVIRNEPVSEKVDIWSFGVVLWELLTGEIPYKDVDSSAIIWGV
GSNSLHLPVPSTCPDGFKILMKQTWQSKPRNRPSFRQTLMHLDIASADVLATPQETYFKSQ
AEWREEVKKHFEKIKSEGTCIHRLDEELIRRRREELRHALDIREHYERKLERANNLYMELS
AIMLQLEMREKELIKREQAVEKKYPGTYKRHPVRPIIHPNAMEKLMKRKGVPHKSGMQT
KRPDLLRSEGIPTTEVAPTASPLSGSPKMSTSSSKSRYRSKPRHRRGNSRGSHSDFAAILKN
QPAQENSPHPTYLHQAQSQYPSLHHHNSLQQQYQQPPPAMSQSHHPRLNMHGQDIATCA
NNLRYFGPAAALRSPLSNHAQRQLPGSSPDLISTAMAADCWRSSEPDKGQAGPWGCCQA
DAYDPCLQCRPEQYGSLDIPSAEPVGRSPDLSKSPAHNPLLENAQSSEKTEENEFSGCRSES
SLGTSHLGTPPALPRKTRPLQKSGDDSSEEEEGEVDSEVEFPRRQRPHRCISSCQSYSTFSSE
NFSVSDGEEGNTSDHSNSPDELADKLEDRLAEKLDDLLSQTPEIPIDISSHSDGLSDKECAV

RRVKTQMSLGKLCVEERGYENPMQFEESDCDSSDGECSDATVRTNKHYSSATW
>antioxidant_84
MASELAMSNSDLPTSPLAMEYVNDFDLMKFEVKKEPVETDRIISQCGRLIAGGSLSSTPMS
TPCSSVPPSPSFSAPSPGSGSEQKAHLEDYYWMTGYPQQLNPEALGFSPEDAVEALISNSH
QLQGGFDGYARGAQQLASAAGAGAGASLGGSGEEMGPAAAVVSAVIAAAAAQSGAAPH
YHHHHHHHHAAGHHHHPTAGAPGAAGSASASAGGAGGSGGGSGGPASAGGGGGGGGG
GGGGAAGAGGALHPHHAAAGGLHFDDRFSDEQLVTMSVRELNRQLRGVSKEEVIRLKQ
KRRTLKNRGYAQSCRFKRVQQRHVLESEKNQLLQQVDHLKQEISRLVRERDAYKEKYEK
LVSSGFRENGSSSDNPSSPEFFM
>antioxidant_85
MTMQFLIASNVLLWLALIGCAVLMLGLLRQVGLLHERSSPMGAMITDHGPDVGDAAPTF
DLPDHSGAMVRIGGPSALKRPTLLMFTAPTCPVCDKLFPLIKSIARAEKFSVVMISDGQPD
EHQRFLAKHELGDIRYVVSAEVGMAFQVGKIPYGVLLDPEGVIRAKGLTNTREHLESLLE
ADKSGFASIQQFMTSRKHSHDAKAA
>antioxidant_86
MTSGILIASNVLLWGAFLALAALMLGVIRQIGLLHERSAPLGAMMIDHGPDVGERSPIFNV
NTFDGEPVLVGRSITPGRPSLLMFTGPSCPICQKLLPIIRSVAAIEETDVILISDGTQAEHRQF
LKDHPLDGELYVVSAEIGMRYQVSKVPYGVLLDQDGKILAKGLCNTREHVESLFETIREG
HSTLQNYLKDENTAPKFKQVTANKVH
>antioxidant_87
MDPETCPCPSGGSCTCADSCKCEGCKCTSCKKSCCSCCPAECEKCAKDCVCKGGEAAEA
EAEKCSCCQ
>antioxidant_88
MILLAVLFLCFISSYSASVKGHTTGLSLNNDRLYKLTYSTEVLLDRGKGKLQDSVGYRISS
NVDVALLWRNPDGDDDQLIQITMKDVNVENVNQQRGEKSIFKGKSPSKIMGKENLEALQ
RPTLLHLIHGKVKEFYSYQNEAVAIENIKRGLASLFQTQLSSGTTNEVDISGNCKVTYQAH
QDKVIKIKALDSCKIARSGFTTPNQVLGVSSKATSVTTYKIEDSFVIAVLAEETHNFGLNFL
QTIKGKIVSKQKLELKTTEAGPRLMSGKQAAAIIKAVDSKYTAIPIVGQVFQSHCKGCPSLS
ELWRSTRKYLQPDNLSKAEAVRNFLAFIQHLRTAKKEEILQILKMENKEVLPQLVDAVTSA
QTSDSLEAILDFLDFKSDSSIILQERFLYACGFASHPNEELLRALISKFKGSIGSSDIRETVMII
TGTLVRKLCQNEGCKLKAVVEAKKLILGGLEKAEKKEDTRMYLLALKNALLPEGIPSLLK
YAEAGEGPISHLATTALQRYDLPFITDEVKKTLNRIYHQNRKVHEKTVRTAAAAIILNNNPS
YMDVKNILLSIGELPQEMNKYMLAIVQDILRFEMPASKIVRRVLKEMVAHNYDRFSRSGS
SSAYTGYIERSPRSASTYSLDILYSGSGILRRSNLNIFQYIGKAGLHGSQVVIEAQGLEALIA
ATPDEGEENLDSYAGMSAILFDVQLRPVTFFNGYSDLMSKMLSASGDPISVVKGLILLIDH
SQELQLQSGLKANIEVQGGLAIDISGAMEFSLWYRESKTRVKNRVTVVITTDITVDSSFVK
AGLETSTETEAGLEFISTVQFSQYPFLVCMQMDKDEAPFRQFEKKYERLSTGRGYVSQKR
KESVLAGCEFPLHQENSEMCKVVFAPQPDSTSSGWF
>antioxidant_89
MAIRKLLLLLKPIDPYPFLQTEGASLIKNPQVLQYLESRCKVHKNAIKFCQEILSKKPVEW
KPISRNDLSHPIRDVDMVITVGGDGTLLHASHFIDDSVPVLGVNSDPTQAHEVEELSDQFD
ASRSTGHLCAATVENFEQVLDDILFGRVVPAKVSRISLKLNSETLLSHALNDILIAQPCPAA
VSRFSFKIKNKDGASSPKTVNCRSSGLRICTAAGSTAAMQSAGGFVMPMLSRDLQFMVRE
PISPGSTASLMHSTFKPDQFMDVNWYSDHGTIYIDGCQVQHSVQLGDTIEISSDAPVLNVF

LSHGISQIRSRY
>antioxidant_90
MALRRVLLFVKPFDVYPPRPLAAAASSPPPPPPPLRVSNPKVLNYLDDRCRVHKETINLCK
SVLQRKSIDWISVQRNDMSNPIHDVDLVISVGGDGTLLRASHFLNSSIPVLGVNSDPTCPD
EVDELTDEFDARRSTGHLCAATAANFEQILDATLDGSRQPSELSRISVKLNGLQLPTYALN
DILVSHPCPASVSRFSFRKRSNTGESSHLINCRSSGLRVATPAGSTAAMLSAGGFVMPISSHE
LQYMIREPISPRDADKPLLHGLVKQGQHILVVWYNEEGAVYFDGSHVMHSIQHGDTLEISS
DAPILKVILPENLLKQGS
>antioxidant_91
MSSTYKLNHTDSFANGDAKSLLPNPENGFTHLTSLAQSEKAVQELLLQQTPMQATDDHLV
EFSEALRTVAKALRGAAEGKALAQAEAAEWKRRYELERSKNVELQHKELSNGVCADESN
GQRMEHLAKSPRLYAQEISSNGMETICSHEVLQDGGFNSFNNKLKRKASFKLSWGCKGM
ANDQHKKEIVSFERGNISTAERSSKQISLTWESDPQTVLIITKPNSTSVRVLSVDMVRWLRT
QKGLNIYVEPRVKEELLSESSSFNFVQTWEDDKEISLLHTKVDLLITLGGDGTVLWAASMF
KGPVPPIVPFSMGSLGFMTPFHSEQYRDCLEAILKGPISITLRHRLQCHIIRDKATHEYEPEE
TMLVLNEVTIDRGISSYLTNLECYCDNSFVTCVQGDGLILSTTSGSTAYSLAAGGSMVHPQ
VPGILFTPICPHSLSFRPLILPEHVTVRVQVPFNSRSSAWVSFDGKDRKQLEAGDALVCSMA
PWPVSTACQVESTNDFLRSIHDGLHWNLRKTQSADGP
>antioxidant_92
MEISMPPPQIYVEKTLAIIKPDIVDKEEEIQDIILRSGFTIVQRRKLRLSPEQCSNFYVEKYGK
MFFPNLTAYMSSGPLVAMILARHKAISYWLELLGPNNSLVAKETHPDSLRAIYGTDDLRNA
LHGSNDFAAAEREIRFMFPEVIVEPIPIGQAAKDYLNLHIMPTLLEGLTELCKQKPADPLIW
LADWLLKNNPNKPKLCHHPIVEEPY
>antioxidant_93
MMDLELPPPGLPSQQDMDLIDILWRQDIDLGVSREVFDFSQRQKEHELEKQKKLEKERQE
QLQKEQEKAFFAQLQLDEETGEFLPIQPAQHIPSETSGSANYSQVAPIPKADDLYFDDCMQ
LLAETFPFVDDNEVSSATFQSLVPDIPSHIESPVFTAPPQAQSPETLIVQVATAVLDDMQDIE
QVWEELLSIPELQCLNIQNDKLAETSTVPSPETKLTEIDNYHFYSSMPSLDKEVGNCSPHFL
NAFEDSFNSILSTEDSSQLTVNSLNSSATVNTDFGDEFYSAFIAEPSTSNGMPSSATLSQSLS
ELLNGPIDLSDLSLCKAFNQNHPESTTAEFNDSDSGISLNTTSPSMASPDHSVESSIYGDTLL
GFSDSEMEEIDSTPGNVKQKGPKTPSVWPPGDPVQPLSSSQGNSAAARDSQCENAPKKEV
PVSPGHRKTPFTKDKHSSRLEAHLTRDELRAKALHIPFPVEKIINLPVEDFNEMMSKEQFN
EAQLALIRDIRRRGKNKVAAQNCRKRKLENIVELEQDLDHLKDEKEKLLKERGENDKSLH
LLKKQLSTLYLEVFSMLRDENGKPYSPSEYSLQQTSDGNVFLVPKSKKPDTKKN
>antioxidant_94
MAFSVQMPALGESVTEGTVTRWLKQEGDTVELDEPLVEVSTDKVDTEIPSPAAGVLTKIIA
QEDDTVEVGGELAVIGDAKDAGEAAAPAPEKVPAAQPESKPAPEPPPVQPTSGAPAGGDA
KPVLMPELGESVTEGTVIRWLKKIGDSVQVDEPLVEVSTDKVDTEIPSPVAGVLVSISADE
DATVPVGGELARIGVAADIGAAPAPKPAPKPVPEPAPTPKAEPAPSPPAAQPAGAAEGAPYV
TPLVRKLASENNIDLAGVTGTGVGGRIRKQDVLAAAEQKKRAKAPAPAAQAAAAPAPKA
PPAPAPALAHLRGTTQKASRIRQITANKTRESLQATAQLTQTHEVDMTKIVGLRARAKAAF
AEREGVNLTFLPFFAKAVIDALKIHPNINASYNEDTKEITYYDAEHLGFAVDTEQGLLSPVI
HDAGDLSLAGLARAIADIAARARSGNLKPDELSGGTFTITNIGSQGALFDTPILVPPQAAM
LGTGAIVKRPRVVVDASGNESIGVRSVCYLPLTYDHRLIDGADAGRFLTTIKHRLEEGAFE

ADLGL
>antioxidant_95
MENKFDHMKLENQLCFLLYASSREMTKQYKPLLDKLNITYPQYLALLLLWEHETLTVKK
MGEQLYLDSGTLTPMLKRMEQQGLITRKRSEEDERSVLISLTEDGALLKEKAVDIPGTILGL
SKQSGEDLKQLKSALYTLLETLHQKN
>antioxidant_96
MTKDKNSPGLKKKSQSVDINAPGFNPLAGAGKQTPQASKPPAPKTPIIEEEQNNAANTQK
HPSRRSELKRFYTIDTGQKKTLDKKDGRRMSFQKPKGTIEYTVESRDSLNSIALKFDTTPN
ELVQLNKLFSRAVVTGQVLYVPDPEYVSSVESSPSLSPVSPLSPTSSEAEFDKTTNPDVHPT
EATPSSTFTGIRPARVVSSTSEEEEAFTEKFLKINCKYITSGKGTVSGVLLVTPNNIMFDPHK
NDPLVQENGCEEYGIMCPMEEVMSAAMYKEILDSKIKESLPIDIDQLSGRDFCHSKKMTG
SNTEEIDSRIRDAGNDSASTAPRSTEESLSEDVFTESELSPIREELVSSDELRQDKSSGASSES
VQTVNQAEVESLTVKSESTGTPGHLRSDTEHSTNEVGTLCHKTDLNNLEMAIKEDQIADN
FQGISGPKEDSTSIKGNSDQDSFLHENSLHQEESQKENMPCGETAEFKQKQSVNKGKQGK
EQNQDSQTEAEELRKLWKTHTMQQTKQQRENIQQVSQKEAKHKITSADGHIESSALLKE
KQRHRLHKFLCLRVGKPMRKTFVSQASATMQQYAQRDKKHEYWFAVPQERTDHLYAFFI
QWSPEIYAEDTGEYTREPGFIVVKKIEESETIEDSSNQAAAREWEVVSVAEYHRRIDALNT
EELRTLCRRLQITTREDINSKQVATVKADLESESFRPNLSDPSELLLPDQIEKLTKHLPPRTIG
YPWTLVYGTGKHGTSLKTLYRTMTGLDTPVLMVIKDSDGQVFGALASEPLKVSDGFYGT
GETFVFTFCPEFEVFKWTGDNMFFIKGDMDSLAFGGGGGEFALWLDGDLYHGRSHSCKT
FGNRTLSKKEDFFIQDIEIWAFE
>antioxidant_97
MTATNKQVILKDYVSGFPTESDFDFTTTTVELRVPEGTNSVLVKNLYLSCDPYMRIRMGKP
DPSTAALAQAYTPGQPIQGYGVSRIIESGHPDYKKGDLLWGIVAWEEYSVITPMTHAHFKI
QHTDVPLSYYTGLLGMPGMTAYAGFYEVCSPKEGETVYVSAASGAVGQLVGQLAKMMG
CYVVGSAGSKEKVDLLKTKFGFDDAFNYKEESDLTAALKRCFPNGIDIYFENVGGKMLDA
VLVNMNMHGRIAVCGMISQYNLENQEGVHNLSNIIYKRIRIQGFVVSDFYDKYSKFLEFV
LPHIREGKITYVEDVADGLEKAPEALVGLFHGKNVGKQVVVVARE
>antioxidant_98
MFTLKKPLLVLLFLGTVSLSLCEQERAADDDEGEVIEEEVKRGFMDTAKNVAKNVAVTLI
DKLRCKVTGGC
>antioxidant_99
MSVEIESIEHELEESIASLRQAGVRITPQRQAILRYLISSHTHPTADEIYQALSPDFPNISVATI
YNNLRVFKDIGIVKELTYGDSSSRFDFNTHNHYHIICEQCGKIVDFQYPQLNEIERLAQHM
TDFDVTHHRMEIYGVCKECQDK
>antioxidant_100
MAKLTALTLLGLGLALFDGQKSSFQTRFNVHREVTPVELPNCNLVKGIDNGSEDLEILPNG
LAFISAGLKYPGIMSFDPDKPGKILLMDLNEKDPVVLELSITGSTFDLSSFNPHGISTFTDED
NIVYLMVVNHPDSKSTVELFKFQEKEKSLLHLKTIRHKLLPSVNDIVAVGPEHFYATNDHY
FIDPYLKSWEMHLGLAWSFVTYYSPNDVRVVAEGFDFANGINISPDGKYVYIAELLAHKIH
VYEKHANWTLTPLKSLDFNTLVDNISVDPVTGDLWVGCHPNGMRIFYYDPKNPPASEVLR
IQDILSKEPKVTVAYAENGTVLQGSTVAAVYKGKMLVGTVFHKALYCELSQAN
>antioxidant_101
MIRAANGFRISVRNTAVCLAPNFRQLKGFSIINLGSLQYFRYNSVYSKSIRLVNTLENRIVPV

YKECASPQSIGGKSNLKQLQWPKPPKNILILKKRMDERVDHCFETLVQHLQQTYPDICIITE
TDVAKKFSYLNLYTWTEISDLEQKVDAIITVGGDGTILHAASLFARSGMPPILSFSLGTLGF
LLPFDFGSFQTAFADFYNSRSFVLMRMRLRVAMKTKLYNESIYAMNEMHIHRGLSPHMAV
LKVFVNDKFLTEAVADGLIISTPTGSTAYSLSSGGPIVHPSINALLLTPICPNSLSFRPVLFPDT
FKISIETSNKSRVRPQLSIDGRPLGLTDIGQRIDITSVKDNAIPCIIRSHKEDDWVSDIVSLLR
WNHPFHRKGW
>antioxidant_102
MFVRVKLNKPVKWYRFYSTLDSHSLKLQSGSKFVKIKPVNNLRSSSSADFVSPPNSKLQS
LIWQNPLQNVYITKKPWTPSTREAMVEFITHLHESYPEVNVIVQPDVAEEISQDFKSPLEN
DPNRPHILYTGPEQDIVNRTDLLVTLGGDGTILHGVSMFGNTQVPPVLAFALGTLGFLSPF
DFKEHKKVFQEVISSRAKCLHRTRLECHLKKKDSNSSIVTHAMNDIFLHRGNSPHLTNLDI
FIDGEFLTRTTADGVALATPTGSTAYSLSAGGSIVSPLVPAILMTPICPRSLSFRPLILPHSSHIR
IKIGSKLNQKPVNSVVKLSVDGIPQQDLDVGDEIYVINEVGTIYIDGTQLPTTRKTENDFNN
SKKPKRSGIYCVAKTENDWIRGINELLGFNSSFRLTKRQTDND
>antioxidant_103
MAAPTAAALSTLSTASVTSGKRFITSSFSLSFSSRPLATGVRAAGARAARRSAASASTVVAT
IAVGDKLPDATLSYFDPADGELKTVTVAELTAGRKAVLFAVPGAFTPTCSQKHLPGFIEKAG
ELHAKGVDAIACVSVNDAFVMRAWKESLGLGDADVLLLSDGNLELTRALGVEMDLSDK
PMGLGVRSRRYALLADDGVVKVLNLEEGGAFTTSSAEEMLKAL
>antioxidant_104
MAATAGRLFRASLIRHVSAIPWGISASAALRPAASRRMCLTNALWSGSDQAKFAFSTSSSY
HAPAVTQHAPYFKGTAVVSGEFKEISLDDFKGKYLVLFFYPLDFTFVCPTEIIAFSDKASEF
HDVNCEVVAVSVDSHFSHLAWINTPRKNGGLGHMNIALLSDLTKQISRDYGVLLEGPGLA
LRGLFIIDPNGVIKHLSVNDLPVGRSVEETLRLVKAFQFVEAHGEVCPANWTPESPTIKPHP
TASREYFEKVNQ
>antioxidant_105
MEAPPPPPPLPATTLAPGRSRKLLLLPLLLFLLRAEAVRGFEAEERPRTREEECHFYAGGQV
YPGEVSRVSVAEHSLHLSKAKISKPAPYWEGTAVINGEFKELKLTDYRGKYLVFFFYPLDFT
FVCPTEIIAFGDRIDEFRSINTEVVACSVDSQFTHLAWINTPRRQGGLGSINIPLLADLNHQIS
KDYGVYLEDSGHTLRGLFIIDDKGILRQITLNDLPVGRSVDETLRLVQAFQYTDKHGEVCP
AGWKPGSETIIPDPAGKLKYFDKLN
>antioxidant_106
MRSATKLFKNLSKSVVQLKTVKPVSSLNFGYIKTRQFSTSTTDSSNLFLNNNNQFQNFTFP
TKQQIRIRKPAPAFKGQAVVNGEFKEISLDDYKGKYLYLFFYPLDFTFVCPTEIIAFSNAAEE
FKKAGCELVGCSIDSPFTHLAWINTPRKEGGLGGINIPLLSDLTHQISKDYGVYIEEDGHTIR
GSILIDKEGLVRVITMNDNPVGRSVDEAIRTLKALKFTDQFGEVCPANWSEGDKSMKADP
KGSKEYFEAVNK
>antioxidant_107
MRLGWLRVLGCRPGSVVSRATIVEGASTTAAGTRGCLEGILEWTFGGVRGFRSAAVAMAP
IKVGDAIPSVEVFEKEPGNKVNLAELFKGKKGVLFGLPGAFTPGCSKTHLPGFVEQADAL
KAKGIQVVACLTVNDVFVTEEWARAHKAEGKVRLLADPSGTFGKETDLLLDDSLLFLFG
NHRLKRFSMVIEDGIVKSLNVEPDGTGLTCSLAPNILSQL
>antioxidant_108
DFTPVCTTELGRLAPEFAKRVVFIFGPDKKLKLSILYPATTGRNFDEILR

>antioxidant_109
MFSRICSAQLKRTAWTLPKQAHLQSQTIKTFATAPILCKQFKQSDQPRLRINSDAPNFDADT
TVGKINFYDYLGDSWGVLFSHPADFTPVCTTEVSAFAKLKPEFDKRNVKLIGLSVEDVES
HEKWIQDIKEIAKVKNVGFPIIGDTFRNVAFLYDMVDAEGFKNINDGSLKTVRSVFVIDPK
KKIRLIFTYPSTVGRNTSEVLRVIDALQLTDKEGVVTPINWQPADDVIIPPSVSNDEAKAKF
GQFNEIKPYLRFTKSK
>antioxidant_110
MAPIDVGDFVPDGSISFFDDDDQLQTVSVHSLAAGKKVILFGVPGAFPPTCSMNHVNGFIE
KAEELKSNGVDEIICLSGDDPFMITACSENKHVKFVEDGSGEYIQLLGLELEVKDKGLGVR
SRGFALLLDNLKVIVVNVGSGGDCSLFQLMKMTTTTMSNLPTDLLEEIISRVPRKYMRAV
RLTCKRWNGMFKSQSFTKMHIGKEEAATRELRQTRMIVMMDYNVYLMGIAVNEIPSIETL
GKLTCLDDSEQVKISQVFCCEGLLLCILKDDDTKIVVWNPYLGQTRWIQTRLICCVSGWK
KYALGYGNNSENRSCRSPKILRVTDNFNIFSENIPLQYEIYDFDSDVWTTLDVSPHWFIMSE
RGLSLKGNTYWGAKERHAYGSIDHIICFDFTRERFGPLLPLPFSAWGAQFASLSSVREDKIT
ALFQNCRAYKLELWITTKIDVNNATWSKFFTMDTPYLHEILSLKTFFIDEENKIVVVSNKE
RDTKGDLTHDSIDIINGEARCLWKLGLGKPADKNCWPLVCPYVPSVVQIKQHKGGKTKEQ
SDYKRH
>antioxidant_111
MATSLSVSRFMSSSATVISVAKPLLSPTVSFTAPLSFTRSLAPNLSLKFRNRRTNSASATTRSF
ATTPVTASISVGDKLPDSTLSYLDPSTGDVKTVTVSSLTAGKKTILFAVPGAFTPTCSQKHV
PGFVSKAGELRSKGIDVIACISVNDAFVMEAWRKDLGINDEVMLLSDGNGEFTGKLGVEL
DLRDKPVGLGVRSRRYAILADDGVVKVLNLEEGGAFTNSSAEDMLKAL
>antioxidant_112
MAMSILKLRNLSALRSAANSARIGVSSRGFSKLAEGTDITSAAPGVSLQKARSWDEGVSS
KFSTTPLSDIFKGKKVVIFGLPGAYTGVCSQQHVPSYKSHIDKFKAKGIDSVICVSVNDPFA
INGWAEKLGAKDAIEFYGDFDGKFHKSLGLDKDLSAALLGPRSERWSAYVEDGKVKAVN
VEEAPSDFKVTGAEVILGQI
>antioxidant_113
MAAICLPVAKHSFPSLLNTQTPKPLFSQNLHTIPLSSQSQICGLKFLISSPSSLPPPPSYSARIS
VFAKVSKGSVPPQFTLKDQDGKNVSLTEFKGKPVVVYFYPADETPGCTKQACAFRDSYE
KFKKAGAEVIGISGDDPSSHKAFAKKYRLPYTLLSDEGNKIRREWGVPADLFGTLPGRQT
YVLDKNGTVQLIYNNQFQPEKHIDETLKFLQSA
>antioxidant_114
MVSVGKKAPDFEMAGFYKGEFKTFRLSEYLGKWVVLCFYPGDFTFVUATEVSAVAEKYP
EFQKLGVEVLSVSVDSVFVHKMWNDNELSKMVEGGIPFPMLSDGGGNVGTLYGVYDPE
AGVENRGRFLIDPDGIIQGYEVLILPVGRNVSETLRQIQAFQLVRETKGAEVAPSGWKPGK
KTLKPGPGLVGNVYKEWSVKEAFED
>antioxidant_115
MDAPRRSSRLAAKIANVLDSKGTIIPEAAPVMLKKPAKDESVDSTIQVGDVIPDITLPDED
GTSIRLRDITANKGLVIFAYPKASTPGCTKQGCGFRDNYPKIQASDYEVLGLSFDTSKAQK
AFKDKQNFPYHLLSDPKGELIKKLGAEKPGGGKLFRSHWIFEKGTGKCIVKEIDISPLVSV
DKAFAVITDSEP
>antioxidant_116
MFRNQYDNDVTVWSPQGRIHQIEYAMEAVKQGSATVGLKSKTHAVLVALKRAQSELAAH

QKKILHVDNHIGISIAGLTADARLLCNFMRQECLDSRFVFDRPLPVSRLVSLIGSKTQIPTQR
YGRRPYGVGLLIAGYDDMGPHIFQTCPSANYFDCRAMSIGARSQSARTYLERHMSEFME
CNLDELVKHGLRALRETLPAEQDLTTKNVSIGIVGKDLEFTIYDDDDVSPFLDGLEERPQR
KAQPSQAAEEPAEKADEPMEH
>antioxidant_117
MSSIGTGYDLSASTFSPDGRVFQVEYAMKAVENSSTAIGIRCKDGVVFGVEKLVLSKLYEE
GSNKRLFNVDRHVGMAVAGLLADARSLADIAREEASNFRSNFGYNIPLKHLADRVAMYV
HAYTLYSAVRPFGCSFMLGSYSVNDGAQLYMIDPSGVSYGYWGCAIGKARQAAKTEIEKL
QMKEMTCRDIVKEVAKIIYIVHDEVKDKAFELELSWVGELTNGRHEIVPKDIREEAEKYA
KESLKEEDESDDDNM

>antioxidant_118
MALASVLQRPMPVNQHGFFGLGGGADLLDLGPGSPGDGLSLAAPSWGVPEEPRIEMLHG
TTTLAFKFLHGVIVAADSRATAGAYIASQTVKKVIEINPYLLGTMAGGAADCSFWERLLAR
QCRIYELRNKERISVAAASKLLANMVYQYKGMGLSMGTMICGWDKRGPGLYYVDSEGN
RISGTAFSVGSGSVYAYGVMDRGYSYDLKVEEAYDLARRAIYQATYRDAYSGGAVNLYH
VREDGWIRVSSDNVADLHDKYSSVSVP
>antioxidant_119
MAAALAVRRAGSAPAFGPEALTPDWENREVSTGTTIMAVQFNGGVVLGADSRTTTGSYIA
NRVTDKLTPIHDHIFCCRSGSAADTQAVADAVTYQLGFHSIELNEPPLVHTAASLFKEMCYR
YREDLMAGIIIAGWDPQEGGQVYSVPMGGMMVRQSFAIGGSGSSYIYGYVDATYREGMT
KDECLQFTANALALAMERDGSSGGVIRLAAIQESGVERQVLLGDQIPKFTIATLPPP
>antioxidant_120
MAAVSVFQPPVGGFSFDNCRRNAVLEADFAKKGFKLPKARKTGTTIAGVVYKDGIVLGA
DTRATEGMVVADKNCSKIHFISPNIYCCGAGTAADTDMTTQLISSNLELHSLTTGRLPRVVT
ANRMLKQMLFRYQGYIGAALVLGGVDVTGPHLYSIYPHGSTDKLPYVTMGSGSLAAMA
VFEDKFRPDMEEEEAKKLVSEAIAAGIFNDLGSGSNIDLCVISKSKLDFLRPFSVPNKKGTR
LGRYRCEKGTTAVLTEKVTPLEIEVLEETVQTMDTS
>antioxidant_121
MPGLTIGDTVPNLELDSTHGKIRIHDYVGDGYAIIFSHPADFTPVCTTEMAAMAGYAKEFE
KRGVKLLGISCDDVESHRQWTKDVEAYGGKQQQQQATTTKVTFPILADPARDAIRQLNM
VDPDEKDAAGRSMPSRALHVVGPDKAVKLSFLYPATTGRNMDEVLRAVDSLLTAAKHGG
KVATPANWKPGECAVIAPGVSDEEARKMFPQGFETADLPSKKGYLRFTKV
>antioxidant_122
MGGGWALGDLVPDIQADSTMGHIKVRDYCKDGWTIIFSHPGDYPPVCTTELGKIAAYNPE
FEKRGVKLLGLSTDTVEDHQGWIKDIESYTPDAPVLYPILADPDRKITVALNMMDPDEKD
ANGKPLASRALHIIGPDCRLKLSLLYPGTTGRNFDEVLRVLDSLQLASKHKIATPANWQKG
EPVVISPSVSDEKAKQMFPQGWETVNLPKALRMTFVD
>antioxidant_123
MKKNRLLFRVIILLILSGAVGFTLYQGFFADKEKMQIGKEAPNFVVTDLEGKKIELKDLKG
KGVFLNFWGTWCKPCEKEMPYMNELYPKYKEKGVEIIALDADETDIAVKNFVNQYGLKF
PVAIDKGQKIIGTYGVGPLPTSFLIDKDGKVVEQIIGEQTKEQLEGYLKKITP
>antioxidant_124
MDKRKRFWMRLSILAVISVALGYTFYSNFFADRSLARAGEQAVNFVLEDLEGESIELRELE

GKGVFLNFWGTYCPPCEREMPHMEKLYGEYKEQGVEIIAVNANEPELTVQRFVDRYGLSF
PIVIDKGLNVIDAYGIRPLPTTILINEHGEIVKVHTGGMTEQMVEEFMELIKPEA
>antioxidant_125
MKKKRRLFIRTGILLVLICALGYTIYNAVFAGKESISEGSDAPNFVLEDTNGKRIELSDLKG
KGVFLNFWGTWCEPCKKEFPYMANQYKHFKSQGVEIVAVNVGESKIAVHNFMKSYGVN
FPVVLDTDRQVLDAYDVSPLPTTFLINPEGKVVKVVTGTMTESMIHDYMNLIKPGETSG
>antioxidant_126
MKKQQRLVMRTAILLVLLAAIGYTIYTNFFTEKTAVAVGSTAPDFVLTDLKGHEHRLSDYR
GKGVFLNFWGTWCKPCEREMPYMNELYPIYKKQGVEILAVNVGEPKLSVEKFAERFGLTF
PIVIDRQDQVLNAYNVGPLPTTFLIDKNGEVKQIITGTMTKEDIERHLESIKP
>antioxidant_127
MDIQQNKTNKQKKKRNRFIFRSSILLILVAAVVFAIVSNMKDDNKIYRVGDAAPDFQLKQI
SEEVDQSTVQLSDLEGKGVMLNFWATWCDPCKAEMPYMQDLYAEYKEKGVEIVAVSLD
GTELVVDQFIDEYDLTFPVPHDKNGEVKDLYKIGPMPTTYFIKPNGEIEEIVQGALTLDRLE
GYLNDIAPQQN
>antioxidant_128
MLTDLECAINSLIDVYHKYSLKKGNYHAVYRDDLKQLLETECPKFMKKKDADTWFKELD
INQDGGINFEEFLVLVIKVGLEAHEEIHKE
>antioxidant_129
MPSELEKALSNLIDVYHNYSNIQGNHHALYKNDFKKMVTTECPQFVQNINIENLFRELDIN
SDNAINFEEFLAMVIKVGVASHKDSHKE
>antioxidant_130
MEDKMSQMESSIETIINIFHQYSVRLGHYDTLIQKEFKQLVQKELPNFLKKQKKNEAAINEI
MEDLDTNVDKQLSFEEFIMLVARLTVASHEEMHNTAPPGQGHRHGPGYGKGGSGSCSGQ
GSPDQGSHDLGSHGHGHGHSHGGHGHSHGGHGHSH
>antioxidant_131
MANKAPSQMERSITTIIDTFHQYSRKEGHPDTLSKKEFRQMVEAQLATFMKKEKRNEALI
NDIMEDLDTNQDNQLSFEECMMLMAKLIFACHEKLHENNPRGHGHSHGKGCGK
>antioxidant_132
MERQEESLSARPALETEGLRFLHTTVGSLLATYGWYIVFSCILLYVVFQKLSARLRALRQR
QLDRAAAAVEPDVVVKRQEALAAARLKMQEELNAQVEKHKEKLKQLEEEKRRQKIEM
WDSMQEGKSYKGNAKKPQEEDSPGPSTSSVLKRKSDRKPLRGGGYNPLSGEGGGACSW
RPGRRGPSSGGUG
>antioxidant_133
MALAVRVVYCGAUGYKPKYLQLKEKLEHEFPGCLDICGEGTPQVTGFFEVTVAGKLVHS
KKRGDGYVDTESKFRKLVTAIKAALAQCQ
>antioxidant_134
MSKTAVCVIKGEKVNGVVKFTQENKDSPVTVNYDITGLEKGEHGFHVHAFGDTTNGCVS
AGPHFNPFGKNHGAPSDEDRHVGDLGNIVADGESNTKGTISDKIISLFGEHTIVGRTMVVH
ADQDDLGKGGKPDSLTTGAAGARLGCGVIGVSQ
>antioxidant_135
MKYTILSLVAGALISCSAMAENTLTVKMNDALSSGTGENIGEITVSETPYGLLFTPHLNGLT
PGIHGFHVHTNPSCMPGMKDGKEVPALMAGGHLDPEKTGKHLGPYNDKGHLGDLPGLV
VNADGTATYPLLAPRLKSLSELKGHSLMIHKGGDNYSDKPAPLGGGGARFACGVIEK

>antioxidant_136
MKKLSGVLAGSLLLISASFSQDLKAHAELINTEGEVIGKAELIETNSGVLIKLNAKGLPPNA
ELAFHIHERGECKPPTFKSAKGHFNPYGKKHGLLNPEGPHAGDMPNIYTDDKGNVRVQV
LNPFVTLKKGEKNSLFKEGGTALVIHSGPDDYKSDPAGNAGKRIACGVIR
>antioxidant_137
KAVAVLRGDSNVSGVVRFEQTHESEPTKIFIGQNSILALTVVVHAGTDDYGK
>antioxidant_138
MNKLIISLLIVLSAISIISADYQYGYCKFGSVGTVNQNITGYVTLTAQDGALNLVYNISSINL
PTGSYATAIMTYGYNPSNNTDLGGVFQVDGKGTEQCQSGGSRAGDLYNLYVNDNRISSNF
DSLTSVSIVDSPNSIIGRSIAIFQESYSCDLLKSSVGTSVGPLTTVVASCIIGIGNSANVPATNG
VNTTTGNANTAGAYSSLSNTQYDAMVLLANTTKSPSAAIGGSVLFRSSSNSVSVNGIVSGV
AKSVHGFHIHAFGDLTTVDGASIGGHWLSGAQVHAFPENTSRHFGDLGNLCIFDNDFKNA
YYYLSTSYFSFSGLVGRGFAVHAARDDGNTYVGGDRVAQGVVALIPKAATTLNQVPSNW
KYEVICSNGTYTGESTIEPSPTPSTTPTPTETSQPGTSSYLAPFFVLILSSLISVILIL
>antioxidant_139
MSPLKAVAVLTGADVKGVVQFT
>antioxidant_140
MEAPRGNLRAVALIAGDNNVRGCLQFVQDISGTTHVTGKISGLSPGFHGFHIHSFGDTTNG
CISTGPHFNPLNRVHGPPNEEERHAGDLGNILAGSNGVAEILIKDKHIPLSGQYSILGRAVV
VHADPDDLGKGGHKLSKSTGNAGSRVGCGIIGLQSSADAKL
>antioxidant_141
MRLLSVLVFLISVISIAKADYQYAFCKFNELSIGGVEGIAHLLSTDGTTLNITFDFTTSYAQN
TQFAAQILTYGYNPSSMTNLGSVFDPTNVKTAGCPSGTPRAGDIGNIQANGGNVEAQTISL
NIPNIKDDANSIIGRSIAIYGGSYDCSDPSKSVIGDMISFCTIGVGNIDYSSFDKSKLTGVNTA
SSYSNLENAIGLAVVYNTTITKGDYIEGRVLFKALNSSFIQVSAKVSGLSYQAHGFHVHQF
GDVSSDNGTSIGGHFLKTGQEHSLPPSGTRHYGDFGNFCAFSQDMMDTGYYYYETDHVT
AALVIGRGMAVHNFTDKGNSDVGGSRCGQGVIALIQDADYSLNLPMDWKWDVICANGS
YYGDMSTSMNSESYNDNEPGSSSTVIPFFALIIFSIIFALL
>antioxidant_142
MSAICVIKGDGVDGIINFKQNDNKSPVIISGVISGLKEGKHGFHVHEFGDTTNGCLSAGAH
FNPFKKEHGSPNDENRHVGDLGNIESNKDKKSIINITDNIITLFGQNSIIGRSIVVHDKEDDL
GRGNSQDSKITGNAGSRLGCGIIALSKI
>antioxidant_143
MVNAIVIIKGLGVEGKVTLSQECEGSPIYINGTVSGLTPGQHGMHVHEFGDTSNGCISAGD
HYNPLHREHGSPLDVERHIGDLGNIKALSNGVATISIRDTIMSLFGDISVMGRTMVIHSDRD
DYGRGNFPDSKTAGHSGKRVGCGIIAKI
>antioxidant_144
MASQTLVSPSPLSSPSLLRTSFSGVSVKLAPQFSTLATSNFKPLTVVAAAKKAVAVLKGQSR
VEGSSTLHQDERQQQLMFVSLAYTQGFMVFTYMSMVIPQMGVSQQDHILIQQVDTWCSE
DEIRHAGDLGNIVANAEGVAECRQSWQIRYHSLRPNSVVGRALVVHELQDDLGKGGHEL
SLSTGNAGGRLACGVVGLTPV
>antioxidant_145
MAAHTILASAPSHTTFSLISPFSSTPTNALSSSLQSSSFNGLSFKLSPTTQSLSLSTSAASKPL
TIVAATKKAVAVLKGTSNVEGVVTLTQEDDGPTTVNVRISGLAPGKHGFHLHEFGDTTNG

CMSTGPHFNPDKKTHGAPEDEVRHAGDLGNIVANTDGVAEATIVDNQIPLTGPNSVVGRA
LVVHELEDDLGKGGHELSPTTGNAGGRLACGVVGLTPV
>antioxidant_146
MMKSLFIASTMVLMAFPAFAESTTVKMYEALPTGPGKEVGTVVISEAPGGLHFKVNMEK
LTPGYHGFHVHENPSCAPGEKDGKIVPALAAGGHYDPGNTHHHLGPEGDGHMGDLPRLS
ANADGKVSETVVAPHLKKLAEIKQRSLMVHVGGDNYSDKPEPLGGGGARFACGVIE
>antioxidant_147
MFMNLLSQVSNAIFPQVEAAQKMSNRAVAVLRGDVVSGTIWITQNSESDPAVIEGEIKGLT
PGLHGFHVHQYGDSTNGCLSAGPHFNPFGKTHGGPNSETRHVGDLGNVEAGADGVAKV
HITDKLITLYGANTVIGRSMVVHAGQDDLGQGVGDKAEESAKTGNAGARAACGVIALAA
PQ
>antioxidant_148
MATVKAVCVLKGEDPVKEPVKGPVKEPVKGIIYFEQQGNGPVTLSGSITGLTEGKHGFHV
HEFGDNTNGCTSAGAHFNPPGKNHGGPQDNERHVGDLGNVIANKEGVAEVCIKDSLISLT
GSQSIIGRTMVVHEKEDDLGKGGNDESLKTGNAGSRLACGVVGIAKL
>antioxidant_149
MIRLSAAAALGLAAALAASPALAQTSATAVVKAGDGKDAGAVTVTEAPHGVLLKLELKG
LTPGWHAAHFHEKGDCGTPDFKSAGAHVHTAATTVHGLLNPDANDSGDLPNIFAAADGA
ATAEIYSPLVSLKGAGGRPALLDADGSSIVVHANPDDHKTQPIGGAGARVACGVIK
>antioxidant_150
MFRTLTVVPLLALGLSLSACADLGQPTVRADLLDQTGKVTGTATFSPSPIGTRVSIEVSGLK
AGPHGLHIHENPNCNPGPDAQGQTIPFGAAGGHFDPGASHNHDGPHARNDQGHGGDLP
MITVGEDGKGRLNFDTNRLKMTGPTGVLGRSIVIHADADDYQTNPAGNSGGRERCGVFQ
AIN
>antioxidant_151
MVVKAVCVINGDAKGTVFFEQESSETPVKVSGEVCGLAKGLHGFHVHEFGDNTNGCMSS
GPHFNPYGKEHGAPVDENRHLGDLGNIEATGDCPTKVSITDSRITLFGADSIIGRTVVVHA
DADDLGKGGHELSKSTGNAGARIGCGVIGIAKV
>antioxidant_152
MKRFSLAILALVVATGAQAASEKVEMNLVTSQGVGQSIGSVTITETDKGLEFSPDLKALPP
GEHGFHIHAKGSCQPATKDGKASAAESAGGHLDPQNTGKHEGPEGAGHLGDLPALVVNN
DGKATDAVIAPRLKSLDEIKDKALMVHVGGDNMSDQPKPLGGGGERYACGVIK
>antioxidant_153
MTAFYKLCGMSMLSLVLADCTFLSANKPYDRDHDGELIVHMKDVNTHKEVGTITISPYIH
DGNQEGMLITPHLYNLPANTTHGMHIHINPSCEDNGIAAGGHWDPDNTQKHLGPYNDNG
HKGDLPVLVVNADGTATEPVVAPKLNSLEELAGHSLMLHAGGDNYSDKPQPLGGGGAR
MWCGVIAD
>antioxidant_154
MKLTKVALFSLGLFGFSSMALAHGDHMHNHDTKMDTMSKDMMSMEKIVVPVQQLDPQ
NGNKDVGTVEITESAYGLVFTPKLHDLAHGLHGFHIHEKPSCEPKEKDGKLVAGLGAGGH
WDPKQTQKHGYPWSDDAHMGDLPALFVMHDGSATTPVLAPRLKKLAEVKGHSLMIHA
GGDNHSDHPAPLGGGGPRMACGVIK
>antioxidant_155
MXKAVAVLTGSEGVXGTIFFTQ

>antioxidant_156
MVNAVAVLKGDSSVSGIVRFEQESEDQQTKISWEITGNDANALRGFHIHTFGDNTNGCTS
AGPHFNPFNKNHGAPEDEERHVGDLGNIPTDAQGISKGSLTDKHIKLLGPLSIVGRTVVVH
AGQDDLGKGGDAESLKTGNAGARHACGVIGISNA
>antioxidant_157
MNKSGIILIGTILFSSMAIADDLTAPIYTTGPKPVAIGKVTFTQTPYGVLITPDLTNLPEGPHG
FHLHKTADCGNHGMHAEGHYDPQNTNSHQGPYGNGHLGDLPVLYVTSNGKAMIPTLAP
RLKLSDMHNLAVMIHANGDTYSDNPPQGGGGDRIACGVIK
>antioxidant_158
MPKPADHRNHAAVSTSVLSALFLGAGAALLSACSSPQHASTVPGTTPSIWTGSPAPSGLSG
HDEESPGAQSLTSTLTAPDGTKVATAKFEFANGYATVTIATTGVGKLTPGFHGLHIHQVGKC
EPNSVAPTGGAPGNFLSAGGHYHVPGHTGTPASGDLASLQVRGDGSAMLVTTTDAFTMD
DLLSGAKTAIIIHAGADNFANIPPERYVQVNGTPGPDETTLTTGDAGKRVACGVIGSG
>antioxidant_159
MKAICIVAGEASGRIYFKQGAPDEPVSITGYLLNLPRGLHGFHVHEFGDTSNGCTSAGEHF
NPTRQRHGAPDAAERHVGDLGNVRSAGCTALTAIHMSDNVITLFGPLSILGRSLVVHTDR
DDLGLGEHPLSKTTGNSGGRLGCGIIGVCAN
>antioxidant_160
MALKAVCVLKGAGETSGTVHFEQEDN
>antioxidant_161
MSLLPTGTLILLVLFILVLITMTKNTSSLNVTKPVSAIAVLEGPVKGTVRFVEESSKVKISVD
ISGLKPNRKHGFHVHEAGDLTDGCTSACAHFNPFGTAHGGPDSKIRHVGDLGNILADKNG
KAKYSFYDSMIKLRGKCNIIGRAIVVHADTDDLGLGGNAESLKTGNAGKRIGCAVIGYAK
ENFC
>antioxidant_162
MNKAKTLLFTALAFGLSHQALAQDLTVKMTDLQTGKPVGTIELSQNKYGVVFTPELADL
TPGMHGFHIHQNGSCASSEKDGKVVLGGAAGGHYDPEHTNKHGFPWTDDNHKGDLPAL
FVSANGLATNPVLAPRLTLKELKGHAIMIHAGGDNHSDMPKALGGGGARVACGVIQ
>antioxidant_163
IVAVLHSSEGVAGTITFIADSQIPLTGPNSI
>antioxidant_164
MMIASFAIFLSHIIFITYATSNQRYFKPNMHNNMTITIRRTITKTATAIAVLHSDNGNINGTIHF
QQDKNSTTISGEIKGLTPGLHGFHVHQYGDTTNGCISAGPHFNPYNKTHGDPTDEMRHVG
DLGNIVAGADGTAHIDISDKHVQLLGPNSIIGRSLVVHADQDDLGKGVGDKKDESLKTGN
AGGRVACGIVAISA
>antioxidant_165
MKTRVVLILALSVCIEAASEVIRARAYIFKAEAGKIPTELIGTIDFDQSGSFLKLNGSVSGLA
AGKHGFHIHEKGDTGNGCLSAGGHYNPHKLSHGAPDDSNRHIGDLGNIESPASGDTLISV
SDSLASLSGQYSIIGRSVVIHEKTDDLGRGTSDQSKTTGNAGSRLACGTIGTVEERILETTT
ASLPPVTQSQPIGSSSYYYSTFYLPIILYFLLSRIL
>antioxidant_166
MMGSFIFLLSIIISINYINSLHTVHRSNIHRNMHNGGMPKKAVAVLKSDTVNGIIYFQQNNR
ASATTIYGTINGLTPGLHGFHIHQYGIKANGCTSAAAHYNPFEKTHGRPTNNIKHIGDLRNI
KAGADGVANVNIISNHIQLSGPLSVIGRSLVVHANPDDLGKGNGDAREESLKTGNAGSRIV

CSIIGIAPST
>antioxidant_167
MTMLQQILLISVIIGTVHVHEVDCANEVLKARAYIFEAVKGGNPAKTVGIIDLVQTGTLVK
MNGSVSGLQPGLHGFHIHEKGDLGNGCLAAGAHFNPHKMMHGAPEDSNRHVGDLGNIE
TPKTGDTPILISDSVISLTGQHNVIGRAIVIHADMDDLGRGTSELSKTTGNAGARVACGVIGI
L
>antioxidant_168
MLAFLFYGLLLAACGSVTMSNPGESSFDLADRLDPVEKIDRLDLVEKIGDTHAKVLEIWM
ELGRRREVDAAEMHAICRVQPSATLPPDQPQITGLVLFRQLGPGSRLEAYFSLEGFPAEQN
ASNRAIHVHEFGDLSQGCDSTGPHYNPMEVPHPQHPGDFGNFVVRNGQLWRHRVGLTAS
LAGPHAILGRSVVVHAGEDDLGKGGNQASLQNGNAGRRLACCVVGTSSSAAWESQTKE
RKKRRRESECKTT
>antioxidant_169
MINSFIVIFLSFLIFINYANLVCVEATHVYGRRSHSNGMHGNGARRAVAVLRGDAGVSGIIY
FQQGSGGSITTISGSVSGLTPGLHGFHVHQYGDQTNGCTSAGDHYNPFGKTHGGPNDRIK
HIGDLGNIVAGANGVAEVYINSYDIKLRGPLSVIGHSLVVHANTDDLGQGTGNMREESLK
TGNAGSRLACGVIGIAAVS
>antioxidant_170
MTVYSYLVILFILLDNYCSAYGYGYSYYHRRHFDPAIASFTKEPYIGAVWFTQHGDYMYV
NGSVAGLPPGKLLGTHVHRYGGLGNMCLEAGPHFNPFNQRHGPRHGYPRHAGDLGNIRV
GRGGVAKFDFYVTIKGLGPFDGFIGRALVIHANRDDLGRNRDEGSRTTGNSGPRLACATIG
FRAP
>antioxidant_171
MLCRAVCSASRRLAPALGILGVRQKHSLPDLPYDYGALQPHINAEIMQLHHSKHHAAYLN
NLNIAEEKYQEALAKGDVTAQVALQPALKFNGGGHINHSIFWTNLSPNGGGEPKGELLEA
IKRDFGSFDKFKEKLTAVSVGVQGSGWGWLGFNKERGCLQIAACSNQDPLQGTTGLIPLL
GIDVWEHAYYLQLKNVRPDYLKAIWKVIKNS
>antioxidant_172
MFVARKISPNCKPGVRGKHTLPKLPYDYAALEPIICREIMELHHQKHHQTYVNNLNAAEE
QLEEAKSKSDTTKLIQLAPALRFNGGGHINHTIFWQNLSPNKTQPSDDLKKAIESQWKSLE
EFKKELTTLTVAVQGSGWGWLGFNKKSGKLQLAALPNQDPLEASTGLIPLFGIDVWEHAY
YLQYKNVRPSYVEAIWDIANWDDISCRFQEAKKLGC
>antioxidant_173
MLSRLFAPKVTVSAHCDLPCGVYDPAQARIEAESVKAVQEKMAGNDDPHFQTRATVIKE
QRAELAKHHVSVLWSDYFKPPHFEKYPELHQLVNDTLKALSAAKGSKDPATGQKALDYI
AQIDKIFWETKKA
>antioxidant_174
MSKVLAIVTGASKGFGKAIAQEIVKCNPNKNQLIDLVLFARSLDGLKSTKESIETISTNAKV
IDLQSLDMSNIPDVELKFKSVLENIKWEEYSKICFFNNHGTLYHLGRIEDFSDFKNIQKDND
TNTTSFVVTTSLLVKKLKELGSQYSSEVTIVNSSSLCAIKAFPTWSLYCSSRAYRDMFFQAL
SLEYKLNDKFKVLNYSLGVLDTDMQSQVREECSEDDKSFYVGLKNDGKLINPNRSASICS
NLVYSHKFETGSHLDYYDLDK
>antioxidant_175
MTSIHTGSNNNIVELDMSELIRPIPPVLDMNKVNSMMETMTGKTPPASCGLTSEDLEAGEL

PPVDVLTFKKSGKPYYFAFGGCHRLRAHDEAGRKKVRCKLVNCSPNTLRLYLGASANKF
LDSD
>antioxidant_176
MSLQSNSVKPTEIPLSEIRRPLAPVLDPQKIDAMVATMKGIPTASKTCSLEQAEAAASAGEL
PPVDVLGVRVKGQTLYYAFGGCHRLQAYDRRARETQNAAFPVRCRVLPATPRQIRMYLGS
SLDIE
>antioxidant_177
MGLRAGGTLGRAGAGRGAPEGPGPSGGAQGGSIHSGRIAAVHNVPLSVLIRPLPSVLDPA
KVQSLVDTIREDPDSVPPIDVLWIKGAQGGDYFYSFGGCHRYAAYQQLQRETIPAKLVQST
LSDLRVYLGASTPDLQ
>antioxidant_178
MANLMMRLPISLRSFSVSASSSNGSPPVIGGSSGGVGPMIVELPLEKIRRPLMRTRSNDQN
KVKELMDSIRQIGLQVPIDVIEVDGTYYGFSGCHRYEAHQKLGLPTIRCKIRKGTKETLRH
HLR
>antioxidant_179
MEFISHFLRATSRRTAALGPILQRNRSEIIQKQSLTNRQAFRRYRSSCSTMDTTVHSAGIDET
HLVPMSVIQRPIPSVLDEQKVQSLMETIKNETSEDEVPPIDLLWISGSEGGDYYFSFGGCHR
FEAYKRLQRPTIKAKLVKSTLGDLYHYMGSSAPKYLA
>antioxidant_180
MLTKRLLTIYIMLLGLIAWFPGAAQAEEKQPAVPAVFLMKTIEGEDISIPNKGQKTILHFWT
SWCPPCKKELPQFQSFYDAHPSDSVKLVTVNLVNSEQNQQVVEDFIKANKLTFPIVLDSKG
ELMKEYHIITIPTSFLLNEKGEIEKTKIGPMTAEQLKEWTEE
>antioxidant_181
MANEKPTEEVKTENNNHINLKVAGQDGSVVQFKIKRQTPLSKLMKAYCEPRGLSMKQIR
FRFGGQPISGTDKPAQLEMEDEDTIDVFQQPTGGVY
>antioxidant_182
MFQRLNKMFVGEVSSSSNQEPEFNEKEDDEWILVDFIDTCTGFSAEEEEEEEDISEESPTEH
PSVFSCLPASLECLADTSDSCFLQFESCPMEESWFITPPPCFTAGGLTTIKVETSPMENLLIEH
PSMSVYAVHNSCPGLSEATRGTDELHSPSSPRVEAQNEMGQHIHCYVAALAAHTTFLEQP
KSFRPSQWIKEHSERQPLNRNSLRRQNLTRDCHPRQVKHNGWVVHQPCPRQYNY
>antioxidant_183
MIRSVLASVSPLRQILASQLCTSSITLAGIRPLGPKNKAPDFSGTAVVNGDFKTISMKDYKG
KWLILFFYPLDFTFVCPTEITAFSDRCAEFQKLNTELIACSCDSHFSHLAWIQTPRSEVGGL
GDMKIPVLADFNKDIANAFGVLDHETGISYRGLFLIDPSGEIRHSLVNDLSVGRSVDEAFR
TLKAFQFVEKHGEVCPANWSDDKPTIKPGIKESKEYFKKVDGHT
>antioxidant_184
MKLYQKFPETQVITTKGPLDFYRDVFEKGKWLFLFAHPADFTPVCTTEFVGFSKVYEEFK
RLNVELVGMSVDSIYSHIEWLKDIQERYGIQVPFPLIADPDKRLARLLDIIDEASGVTIRAVF
LVNPEGIIRFMAYYPIEYGRKIEELLRITKAALVNYKAKVSLPVDWEPGQEVIVPAPSTIDE
AQIRMKLPNAKTWYLTFKKYDELPQDQRVV
>antioxidant_185
MPDENKFPLIGEKFPEMTVETTHGSMKLPDQYKGRWFMLFSHPGDFTPVCTTEFYSFARR
FEDFQKINTDLIGLSVDSTISHIEWVNWIRDNLKIEVPFPIIGDSMGRVATRLGMIQAESSTS
TVRAVFIVDPNSTVRLIMYYPLEIGRNVDELLRAIKALQMSDKYRGAMPANWPYNELIGE

KMLNPAPKNVKDATGRLKEFKGYAWWLTYKDVPDNEVQETKKIVKMREEK
>antioxidant_186
METEAIENIPRVCLPQIGAPAPDFKANSTFGPIKLSDYRGKWVVLFSHPGDFTPVCTTEFIA
FTQVYTSFVERNVQLIGLSVDSNPSHLAWVENIYKTTGVEIPFPIIEDKDMRIAKLYGMISP
AETSTSAVRAVFIIDDKQILRLILYYPLEIGRNIQEIIRIIDALQTVDKYKVLAPANWYPGMPV
IVPPPKTYPELKQRLKNVEGYTCTDWYLCYKKV
>antioxidant_187
MPGSIPLIGERFPEMEVTTDHGVIKLPDHYVSQGKWFVLFSHPADFTPVCTTEFVSFARRY
EDFQRLGVDLIGLSVDSVFSHIKWKEWIERHIGVRIPFPIIADPQGTVARRLGLLHAESATH
TVRGVFIVDARGVIRTMLYYPMELGRLVDEILRIVKALKLGDSLKRAVPADWPNNEIIGEG
LIVPPPTTEDQARARMESGQYRCLDWWFCWDTPASRDDVEEARRYLRRAAEKPAKLLYE
EARTHLH
>antioxidant_188
MEVVSLPRLGEPAPAFEAQTTFGPVKFPDDFKGQWVVLFSHPADFTPVCTTEFVAFAKNY
EEFKKRNVQLIGLSVDSNFSHIAWVMNIKEKFGIEIPFPIIADHNMEVAKKYGMIHPAQSTT
FTVRALFVIDDKGILRAMIYYPLTTGRNIREVIRLVDALQTADREGVATPADWVPEPQTWE
FTEENTKVIVPPPTTYEDAVKRLQEGYECADWYICKKKV
>antioxidant_189
MPLLGDDFPELKVQTTHGPMNIPGDLKGSWFVLFSHPADFTPVCTTEFVAFQQRVEAFEKI
GCKLIGMSVDQVFSHIKWVEWIKENLDVDITFPIVAANDRIANKLGMLHPGKGTNTVRAV
FVGDPNGKVRLVLYYPQEIGRNMDEILRAVKVLQISDSNKVAMPADWPNNLLIKDHVIIPP
ANNVEDAKKRKEQQYDCYDWWFCHKPLDK
>antioxidant_190
MPLIGDDAPSFTAVTTQGIIKFPDDYKGKWVILFSHPADFTPVCTTEFMTFASMQEEFRSM
NTELIGLSIDSVFSHIAWLKRIEEKIEYKGMKNLEIKFPVIEDLKMDVAKKYGMVQPKAST
TQAVRAVFIIDPEAKIRTILYYPQSTGRNMQEIKRIVVALQKNAAEKVATPANWQPGEDVIIP
PPSSMEAVKERMGKEEEGKRCLDWFMCLKKDTQK
>antioxidant_191
MVVLGQKFPEVEVQTTHGRMRLPDHYRGKWFVLFSHPADFTPVCTTEFVEFARNYDKFK
AMNTELIGLSIDQVFSHIKWIEWIKEKFNVEIPFPVIADDQGELARMLGMISPYKGTNTVRA
VFIVDPEGYIRAMLYYPQETGRNIPEILRLVEALQTADKYGVATPANWHVDRYEFKPSSIVG
NDVIIPPASSLEEKKDREERAKKGEIECFDWWFCHKKLE
>antioxidant_192
MPVVLGQKAPDFTVNTSKGPITLSNYRGKWVLLFSHPGDFTPVCTTEFIAFTERYEDFQKL
GVELIGLSIDSVFSHIAWIRDIKEHFGIDIPFPIIADIDKEVAREYNLIDEKSGATVRGVFIIDPN
QIVRWMIYYPAETGRNIEEIIRVIKALQFNWDRKLATPANWQPGQEGIEPAPTTVDSSFKRD
NEQGVKTWYLKFVK
>antioxidant_193
MFPKTLTDSKYKAFVDGEIKEISLQDYIGKYVVLAFYPLDFTFVCPTEINRFSDLKGAFLRR
NAVVLLISCDSVYTHKAWASIPREQNGVLGTAWPMVWDAKRELCNQFGLYDEENGHPM
RSTVILAKDLSVRHISSNYHAIGRSVDEIIRLIDAITFNDENGDICPAEWRSENKDN
>antioxidant_194
MAAGKARIGHLAPGFTAKAVMPDGQFKDISMSDYRGKYVVFFFYPLDFTFVCPTEIIAFSD
AAEEFRKIGCEVIGASVDSHFCHLAWTNTPRKHGGLGAMKIPLVADTMRSISTDYGVFEG

GMRASPTGGLFIIDDKGVLRQITINDLPVGRCVDEILRLVQAFQFTDKHGEVCPAGWKPGS
DTIKPDVQKSKDFFSKQQ
>antioxidant_195
MKIKKLLKNGLSLFLTFIVITSILDFVRRPVVPEEINKITLQDLQGNTFSLESLDQNKPTLLYF
WGTWCGYCRYTSPAINSLAKEGYQVVSVALRSGNEADVNDYLSKNDYHFTTVNDPKGEF
AERWQINVTPTIVLLSKGKMDLVTTGLTSYWGLKVRLFFAEFFG
>antioxidant_196
MASVEEFRNAQRAKGPATILAIGTATPDHCVYQSDYADYYFRVTKSEHMTELKKKFNRIC
DKSMIKKRYIHLTEEMLEEHPNIGAYMAPSLNIRQEIITAEVPRLGRDAALKALKEWGQPK
SKITHLVFCTTSGVEMPGADYKLANLLGLETSVRRVMLYHQGCYAGGTVLRTAKDLAEN
NAGARVLVVCSEITVVTFRGPSEDALDSLVGQALFGDGSSAVIVGSDPDVSIERPLFQLVSA
AQTFIPNSAGAIAGNLREVGLTFHLWPNVPTLISENIEKCLTQAFDPLGISDWNSLFWIAHP
GGPAILDAVEAKLNLEKKKLEATRHVLSEYGNMSSACVLFILDEMRKKSLKGEKATTGEG
LDWGVLFGFGPGLTIETVVLHSVPTVTN
>antioxidant_197
MAEITFKGGPVTLVGQEVKVGDQAPDFTVLTNSLEEKSLADMKGKVTIISVIPSIDTGVCD
AQTRRFNEEAAKLGDVNVYTISADLPFAQARWCGANGIDKVETLSDHRDMSFGEAFGVY
IKELRLLARSVFVLDENGKVVYAEYVSEATNHPNYEKPIEAAKALVK
>antioxidant_198
MSIVNFKGNPVKLKGNSVEVGADAPKVNLKAKDLSVIEIGAAGKTQIILSVPSLDTPVCAT
EAREFNKKVASYNGAEVIVVSMDLPFAMGRFCSTEGIENLSVASDFVAKEFGEKYGVLINE
GALEGLLARAVFVIKEGKVAYKELVNEITEMPDIAKLDAFFGGSSCCGGCGCH
>antioxidant_199
MKVKFKGKEVTLEGTEIKVGDTFPDFVAVNSSLEPVMLKNTNRVRVFLAVPSVDTPVCDL
EVKTFNARASEIDGVSIYTISMDLPFAQSRWCGAEGIKNVTTLSDYRDRAFGKNTGTYIKE
LGLLARAVFVVDSSNKVTYANYLEEVSGYPNYDEVLQAAQAAK
>antioxidant_200
MATTHFDGTDTPTSGELPAVGDTLPDFTVVDTNLGEVSLSDLKGKKLVLNIFPSVDTGLCA
ESVRRFNAEAAGLDNTTVLCVSKDLPFALGRFCGAEGLDNVTAASAFRSTFGEDYGVVLE
GSPLKGLLARSVIVADEDGKVIYTQLVDEIKTEPDYASAIKALG
>antioxidant_201
MSQTVHFQGNPVTVANSIPQAGSKAQTFTLVAKDLSDVTLGQFAGKRKVLNIFPSIDTGVC
AASVRKFNQLATEIDNTVVLCISADLPFAQSRFCGAEGLNNVITLSTFRNAEFLQAYGVAIA
DGPLKGLAARAVVVIDENDNVIFSQLVDEITTEPDYEAALAVLKA
>antioxidant_202
MQKVTFKEETYQLEGKALKVGDKAPDVKLVNGDLQEVNLLKQGVRFQVVSALPSLTGS
VCLLQAKHFNEQAGKLPSVSFSVISMDLPFSQGQICGAEGIKDLRILSDFRYKAFGENYGV
LLGKGSLQGLLARSVFVLDDKGVVIYKEIVQNILEEPNYEALLKVLK
>antioxidant_203
MTKVTLKGNPVQLEGKIPSPGDKAPDFKAIKQDLSEFSLKDYAGKVKILVAVPSLDTSVCA
LETKAFNEKAAGISGVTTLVISGDLPFAMGRFCSTEGINSPNLVTGSQYRDFSFSKAYGTHI
ADGPLKGLSARAVFVLDKSDTVRYVEIVPEITTEPNYTAAIAAANAAL
>antioxidant_204
MSKITLAGTEIEVSGVFPQVGDVVTDFTTVSAKLEDTTLANFAGKRKILNIFPSIDTGICATS

VRKFNEQAAKLANTAVLCLSTDLPFAQARFCGAEGLENVFTVSTFRNKDVHKQLGVDIVE
GPLAGLTARAVIVLDENNRVLHSELVPEIKQEPNYDAALAVL
>antioxidant_205
MTTFLGNSVTFTGKQLQVGDIAKDFLLIATDLSQKSLKDFEGKKKVISVVPSIDTGICSKQT
RTFNEELSELDNTVVITVSMDLPFAQKRWCSAEGLDNVILLSDFYDHSFGQDYALLMNEW
HLLTRAVLILDEHNKVTYTEYVDNVNSDVDYEAAINAAKILP
>antioxidant_206
MTVTFQNNPVSISGSFPKVGDRLPSFTLCGADLNDLNNEDFKGKKIVMSIFPSIDTPVCSKS
VKVLQNALMTRSDTVLLCVSADLPFAMSRFCTEHAVANVTNASFFREPAFTERFGVNLNE
GALRGLAARAVIVADEFGVITHSELVNEITNEPDYDRILMSL
>antioxidant_207
MVKQVSDSSEFKSIVCQDKLVVVDFFATWCGPCKAIAPKFEQFSNTYSDATFIKVDVDQLS
EIAAEAGVHAMPSFFLYKNGEKIEEIVGANPAKLEASIKANL
>antioxidant_208
MVTQLKSASEYDSALASGDKLVVVDFFATWCGPCKMIAPMIEKFAEQYSDAAFYKLDVD
EVSDVAQKAEVSSMPTLIFYKGGKEVTRVVGANPAAIKQAIASNV
>antioxidant_209
MAAEEGVVIACHNKDEFDAQMTKAKEAGKVVIIDFTASWCGPCRFIAPVFAEYAKKFPGA
VFLKVDVDELKEVAEKYNVEAMPTFLFIKDGAEADKVVGARKDDLQNTIVKHVGATAAS
ASA
>antioxidant_210
MNLCNSRFSVTFVRQCSTILTSPSAGIIQNRGSLTTKVPHWISSSLSCAHHTFQRTMNLTGQ
RGSRDSTGATGGNAPAGSGAGAPPPFQHPHCDRAAMYAQPVRKMSTKGGSYDYDLIVIG
GGSAGLACAKEAVLNGARVACLDFVKPTPTLGTKWGVGGTCVNVGCIPKKLMHQASLL
GEAVHEAAAYGWNVDEKIKPDWHKLVQSVQNHIKSVNWVTRVDLRDKKVEYINGLGSF
VDSHTLLAKLKSGERTITAQTFVIAVGGRPRYPDIPGAVEYGITSDDLFSLDREPGKTLVVG
AGYIGLECAGFLKGLGYEPTVMVRSIVLRGFDQQMAELVAASMEERGIPFLRKTVPLSVE
KQDDGKLLVKYKNVETGEEAEDVYDTVLWAIGRKGLVDDLNLPNAGVTVQKDKIPVDS
QEATNVANIYAVGDIIYGKPELTPVAVLAGRLLARRLYGGSTQRMDYKDVATTVFTPLEYA
CVGLSEEDAVKQFGADEIEVFHGYYKPTEFFIPQKSVRYCYLKAVAERHGDQRVYGLHYI
GPVAGEVIQGFAAALKSGLTINTLINTVGIHPTTAEEFTRLAITKRSGLDPTPASCCS
>antioxidant_211
MAPVVQQPAPSFKKTAVVDGVFEEVTLEQYKGKWVLLAFIPLAFTFVCPSEIIAYSEAVKK
FAEKDAQVLFASTDSEYTWLAWTNVARKDGGIGKVDFPVLADTNHSLSRDYGVLIEEEGV
ALRGIFLIDPKGVLRQITINDLPVGRSVEESLRLLEAFQFTEKYGEVCPANWHPGDETIKPS
PEASKEYFNKVNK
>antioxidant_212
MVLVTYPAPDFTASAISCNGDIINNFNFKEFTNNQTSILFFWPMDFTFVCPSEIIAFNQELSK
FKKRNVKLIGVSIDSVYVHHAWRNTLSHNGQIDKINFTMVSDLKREIQRSYGIEHPQLGVA
LRATFLIDKNRIIRHQTINDLPFGRNISETLRMIDALHFYEKYGEVCPANWKKGDTGIKTTQ
EGIHKYLEKISKK
>antioxidant_213
MLVTKLAPDFKAPAVLGNNEVDEHFELSKNLGKNGVILFFWPKDFTFVCPTEIIAFDKRVK
DFHEKGFNVIGVSIDSEQVHFAWKNTPVEKGGIGQVSFPMVADITKSISRDYDVLFEEAIAL

RGAFLIDKNMKVRHAVINDLPLGRNADEMLRMVDALLHFEEHGEVCPAGWRKGDKGM
KATHQGVAEYLKENSIKL
>antioxidant_214
LCPLDVLQLSSELLDIDGNEVEASRILSDITAFGGIRCPLTVVQSRGIGTIISSPYRFIAEGHPL
SLKDMDGWFRVSDDEFNNYK
>antioxidant_215
MSVIEIRSYQHWISTIPKSGYLAVDCYADWCGPCKAISPLFSQLASKYASPKFVFAKVNVD
EQRQIASGLGVKAMPTFVFFENGKQIDMLTGANPQALKEKVALISSKATGTGALASSSSAP
VKGFASLQGCIENPQLECLNQQDDHDLKSAFNSNPSSFLESDVDEQLMIYIPFLEVVKVHSI
AITPVKGETSSAPKTIKLYINQPNNLSFEDAESFTPTQVIEDIVYEQDDQPTIIPLRFVKFQRV
NSLVIFIYSNVGEEETTKISRLELFGEPVGDSSKGKLQKVEA
>antioxidant_216
MQLKPMEINPEMLNKVLSRLGVAGQWRFVDVLGLEEESLGSVPAPACALLLLFPLTAQHE
NFRKKQIEELKGQEVSPKVYFMKQTIGNSCGTIGLIHAVANNQDKLGFEDGSVLKQFLSET
EKMSPEDRAKCFEKNEAIQAAHDAVAQEGQCRVDDKVNFHFILFNNVDGHLYELDGRMP
FPVNHGASSEDTLLKDAAKVCREFTEREQGEVRFSAVALCKAA
>antioxidant_217
MVNVKVEFLGGLDAIFGKQRVHKIKMDKEDPVTVGDLIDHIVSTMINNPNDVSIFIEDDSI
RPGIITLINDTDWELEGEKDYILEDGDIISFTSTLHGG
>antioxidant_218
MSGTRPAARRTNLTAAQNVVRSVDAEERIAWVSKALCRTTDPDELFVRGAAQRKAAVICR
HCPVMQECAADALDNKVEFGVWGGMTERQRRALLKQHPEVVSWSDYLEKRKRRTGTA
G
>antioxidant_219
MSVSTAKRSLDVVSPGSLAEFEGSKSRHDEIENEHRRTGTRDGEDSEQPKKKGSKTSKKQ
DLDPETKQKRTAQNRAAQRAFRERKERKMKELEKKVQSLESIQQQNEVEATFLRDQLITL
VNELKKYRPETRNDSKVLEYLARRDPNLHFSKNNVNHSNSEPIDTPNDDIQENVKQKMN
FTFQYPLDNDNDNDNSKNVGKQLPSPNDPSHSAPMPINQTQKKLSDATDSSSATLDSLSNS
NDVLNNTPNSSTSMDWLDNVIYTNRFVSGDDGSNSKTKNLDSNMFSNDFNFENQFDEQV
SEFCSKMNQVCGTRQCPIPKKPISALDKEVFASSSILSSNSPALTNTWESHSNITDNTPANVI
ATDATKYENSFSGFGRLGFDMSANHYVVNDNSTGSTDSTGSTGNKNKKNNNNSDDVLPF
ISESPFDMNQVTNFFSPGSTGIGNNAASNTNPSLLQSSKEDIPFINANLAFPDDNSTNIQLQP
FSESQSQNKFDYDMFFRDSSKEGNNLFGEFLEDDDDDKKAANMSDDESSLIKNQLINEEP
ELPKQYLQSVPGNESEISQKNGSSLQNADKINNGNDNDNDNDVVPSKEGSLLRCSEIWDR
ITTHPKYSDIDVDGLCSELMAKAKCSERGVVINAEDVQLALNKHMN
>antioxidant_220
MTNFPKIGKTPPNFLTIGVYKKRLGKIRLSDYRGKKYVILFFYPANFTAISPTELMLLSDRIS
EFRKLSTQILAISVDSPFSHLQYLLCNREEGGLEDLNYPLVSDLTQTITRDYQVLTDEGLAF
PGLFIIDKEGIIQYYTVNNLLCGRNINELLRILESIQYVKENPGYACPVNWNFGDQVFYSHP
LKSKIYFKDLYSPKKSS
>antioxidant_221
MIASLDELYHSELFFLPVMDENARLVGLEIIATFAAEDGAVRMPTELVAPRLSVEEQYCLFV
EKLALLETCQHFFIQHKLIAWLNLPPAISDLLLLDSELFSRAARFPFLELAINENYPGLNQG
KNNETLANLAMHFPLMLANFGAGEASTKAIFDGLFKRVMLDKNFIQQRAEMISFEPFMH

AIVAQISSSCESLMIAGIDTEAMFARAAPLGFSAFQGGLWPPVPVSQLIKLVQR
>antioxidant_222
MAERMVGKQAPRFEMEAVLASKEFGKVSLEENMKNDKWTVLFFYPMDFTFVCPTEITA
MSDRYDEFEDLDAEVIGVSTDTIHTHLAWINTDRKENGLGQLKYPLAADTNHEVSREYG
VLIEEEGVALRGLFIINPEGELQYQTVFHNNIGRDVDETLRVLQALQTGGLCPANWKPGQK
TL
>antioxidant_223
MKLRQPMPELTGEKAWLNGEVTREQLIGEKPTLIHFWSISCHLCKEAMPQVNEFRDKYQD
QLNVVAVHMPRSEDDLDPGKIKETAAEHDITQPIFVDSDHALTDAFENEYVPAYYVFDKT
GQLRHFQAGGSGMKMLEKRVNRVLAETE
>antioxidant_224
MLKKWLAGILLIMLVGYTGWNLYQTYSKKEVGIQEGQQAPDFSLKTLSGEKSSLQDAKG
KKVLLNFWATWCKPCRQEMPAMEKLQKEYADKLAVVAVNFTSAEKSEKQVRAFADTYD
LTFPILIDKKGINADYNVMSYPTTYILDEKGVIQDIHVGTMTKKEMEQKLDLD
>antioxidant_225
SYTLPSLPYAYDALEPHFDKQTMEIHHTKHHQTYVNNANAALESLPEFANLPVEELITKLD
QLPADKKTVLRNNAGGHANHSLFWKGLKKGTTLQGDLKAAIERDFGSVDNFKAEFEKA
AASRFGSGWAWLVLKGDKLAVVSTANQDSPLMGEAISGASGFPIMGLDVWEHAYYLKFQ
NRRPDYIKEFWNVVNWDEAAARFAAKK
>antioxidant_226
HHHHDYDIPTTENLYFQGHMTIHKKGQAHWEGDIKRGKGTVSTESGVLNQQPYGFNTRF
EGEKGTNPEELIGAAHAACFSMALSLMLGEAGFTPTSIDTTADVSLDKVDAGFAITKIALK
SEVAVPGIDASTFDGIIQKAKAGCPVSQVLKAEITLDYQLKS
>antioxidant_227
MSGLAKYLPGATNLLSKSGEVSLGSLVGKTVFLYFSASWCPPCRGFTPVLAEFYEKHHVA
KNFEVVLISWDENESDFHDYYGKMPWLALPFDQRSTVSELGKTFGVESIPTLITINADTGA
IIGTQARTRVIEDPDGANFPWPN
>antioxidant_228
PHMSGLKKFFPYSTNVLKGAAADIALPSLAGKTVFFYFSASWCPPCRAFTPQLIDFYKAH
AEKKNFEVMLISWDESAEDFKDYYAKMPWLALPFEDRKGMEFLTTGFDVKSIPTLVGVE
ADSGNIITTQARTMVVKDPEAKDFPWPNVEAKK
>antioxidant_229
MSCGEAEDLHPAPDFNETALMPNGTFKKVALTSYKGKWLVLFFYPMDFTFVCPTEICQFS
DRVKEFSDIGCEVLACSMDSEYSHLAWTSIERKRGGLGQMNIPILADKTKCIMKSYGVLK
EEDGVAYRGLFIIDPKQNLRQITVNDLPVGRDVDEALRLVKAFQFVEKHGEVCPANWKPG
DKTMKPDPEKSKEYFGAVAKLP
>antioxidant_230
GSSGSSGTEERLKEIEAKYDEIAKDWPKKVKHVLHEEHELELTRVQVYTCDKCEEEGTIW
SYHCDECDFDLHAKCALNEDTKESGPSSG
>antioxidant_231
MGYHLGATFPNFTAKASGIDGDFELYKYIENSWAILFSHPNDFTPVCTTELAELGKMHEDF
LKLNCKLIGFSCNSKESHDKWIEDIKYYGKLNKWEIPIVCDESRELANKLKIMDEQEKDIT
GLPLTCRCLFFISPEKKIKATVLYPATTGRNAHEILRVLKSLQLTYTTPVATPVNWNEGDKC
CVIPTLQDDEISKHFKNEITKVEMPSKKKYLRFVNL

>antioxidant_232
MAQITLRGNAINTVGELPAVGSPAPAFTLTGGDLGVISSDQFRGKSVLLNIFPSVDTPVCAT
SVRTFDERAAASGATVLCVSKDLPFAQKRFCGAEGTENVMPASAFRDSFGEDYGVTIADG
PMAGLLARAIVVIGADGNVAYTELVPEIAQEPNYEAALAALGATSGSHHHHHH
>antioxidant_233
KVPRGSHMLNVGATAPDFTLRDQNQQLVTLRGYRGAKNVLLVFFPLAFTGICQGELDQLR
DHLPEFENDDSAALAISVGPPPTHKIWATQSGFTFPLLSDFWPHGAVSQAYGVFNEQAGIA
NRGTFVVDRSGIIRFAEMKQPGEVRDQRLWTDALAALTA
>antioxidant_234
MGHHHHHHHHHHSSGHIEGRHMKLSLVTKKAYNFTAQGLNKNNEIINVDLSSFIGQKYC
CLLFYPLNYTFVCPTEIIEFNKHIKDFENKNVELLGISVDSVYSHLAWKNMPIEKGGIGNVE
FTLVSDINKDISKNYNVLYDNSFALRGLFIIDKNGCVRHQTVNDLPIGRNVQEVLRTIDSIIH
VDTSGEVCPINWKKGQKAFKPTTESLIDYMNNANKNV
>antioxidant_235
MLQYPELPLESPLIDAELPDPRGGRYRLSQFHEPLLAVVFMCNHCPYVKGSIGELVALAER
YRGKVAFVGINANDYEKYPEDAPEKMAAFAEEHGIFFPYLLDETQEVAKAYRALRTPEVF
LFDERRLLRYHGRVNDNPKDPSKVQSHDLEAAIEALLRGEEPPLKEAPAIGCTIKWRPGNE
PEVRIG
>antioxidant_236
MKGLVELGEKAPDFTLPNQDFEPVNLYEVLKRGRPAVLIFFPAAFSPVCTKELCTFRDKMA
QLEKANAEVLAISVDSPWCLKKFKDENRLAFNLLSDYNREVIKLYNVYHEDLKGLKMVA
KRAVFIVKPDGTVAYKWVTDNPLNEPDYDEVVREANKIAGELVA
>antioxidant_237
QVKLLEQSGAEVKKPGASVKVSCKASGYSFTSYGLHWVRQAPGQRLEWMGWISAGTGN
TKYSQKFRGRVTFTRDTSATTAYMGLSSLRPEDTAVYYCARDPYGGGKSEFDYWGQGTL
VTVSSASPTSPKVFPLSLCSTQPDGNVVIACLVQGFFPQEPLSVTWSESGQGVTARNFPPSQ
DASGDLYTTSSQLTLPATQCLAGKSVTCHVKHYTNPSQDVTVPCPVPSTPPTPSPSTPPTPS
PSCCHPRLSLHRPALEDLLLGSEANLTCTLTGLRDASGVTFTWTPSSGKSAVQGPPERDLC
GCYSVSSVLPGCAEPWNHGKTFTCTAAYPESKTPLTATLSKSGNTFRPEVHLLPPPSEELAL
NELVTLTCLARGFSPKDVLVRWLQGSQELPREKYLTWASRQEPSQGTTTFAVTSILRVAAE
DWKKGDTFSCMVGHEALPLAFTQKTIDRLAGKPTHVNVSVVMAEVDGTCY
>antioxidant_238
MGSSHHHHHHSSGLVPRGSPTYVGKEAPFFKAEAVFGDNSFGEVNLTQFIGKKYVLLYFY
PLDFTFVCPSEIIALDKALDAFHERNVELLGCSVDSKYTHLAWKKTPLAKGGIGNIKHTLL
SDITKSISKDYNVLFDDSVSLRAFVLIDMNGIVQHLLVNNLAIGRSVDEILRIIDAIQHHEKY
GDVCPANWQKGKVSMKPSEEGVAQYLSTL
>antioxidant_239
MGITLGEVFPNFEADSTIGKLKFHDWLGNSWGVLFSHPRDFTPVSTTELGRVIQLEGDFKK
RGVKLIALSCDNVADHKEWSEDVKCLSGVKGDMPYPIIADETRELAVKLGMVDPDERTST
GMPLTCRAVFIIGPDKKLKLSILYPATTGRNFSEILRVIDSLQLTAQKKVATPADWQPGDRC
MVVPGVSAEEAKTLFPNMEVKAVPSGKGYLRYTPQPKSMGGSRSHHHHHH
>antioxidant_240
GHMEERVLGMPAVESNMFPLGKQAPPFALTNVIDGNVVRLEDVKSDAATVIMFICNHCPF
VKHVQHELVRLANDYMPKGVSFVAINSNDAEQYPEDSPENMKKVAEELGYPFPYLYDET

QEVAKAYDAACTPDFYIFDRDLKCVYRGQLDDSRPNNGIPVTGESIRAALDALLEGRPVP
EKQKPSIGCSIKWKPSA
>antioxidant_241
GHMVEIGELAPDFELPDTELKKVKLSALKGKVVVLAFYPAAFTQVCTKEMCTFRDSMAK
FNQVNAVVLGISVDPPFSNKAFKEHNKLNFTILSDYNREVVKKYNVAWEFPALPGYVLAK
RAVFVIDKEGKVRYKWVSDDPTKEPPYDEIEKVVKSLS
>antioxidant_242
GHMARTVNLKGNPVTLVGPELKVGDRAPEAVVVTKDLQEKIVGGAKDVVQVIITVPSLD
TPVCETETKKFNEIMAGMEGVDVTVVSMDLPFAQKRFCESFNIQNVTVASDFRYRDMEK
YGVLIGEGALKGILARAVFIIDKEGKVAYVQLVPEITEEPNYDEVVNKVKELI
>antioxidant_243
MVKVGDKAPLFEGIADNGEKISLSDYIGKHNIVLYFYPKDDTPGSTREASAFRDNWDLLK
DYDVVVIGVSSDDINSHKRFKEKYKLPFILVSDPDKKIRELYGAKGFILPARITFVIDKKGII
RHIYNSQMNPANHVNEALKALKQIKEEEISLEHHHHHH
>antioxidant_244
SNAGMKAADFTYVTVHGDNSRMSRLKAQYTMLFFYDPDCSNCRKFEKLFAEIPAFVEMV
ENGTLRVLAIYPDENREEWATKAVYMPQGWIVGWNKAGDIRTRQLYDIRATPTIYLLDGR
KRVILKDTSMEQLIDYLATQAGK
>antioxidant_245
SNAMKAPELQIQQWFNSATDLTLADLRGKVIVIEAFQMLCPGCVMHGIPLAQKVRAAFPE
DKVAVLGLHTVFEHHEAMTPISLKAFLHEYRIKFPVGVDQPGDGAMPRTMAAYQMRGTP
SLLLIDKAGDLRAHHFGDVSELLLGAEIATLLGEAAPSV
>antioxidant_246
SNAKVTVGKSAPYFSLPNEKGEKLSRSAERFRNRYLLLNFWASWCDPQPEANAELKRLN
KEYKKNKNFAMLGISLDIDREAWETAIKKDTLSWDQVCDFTGLSSETAKQYAILTLPTNIL
LSPTGKILARDIQGEALTGKLKELLKTE
>antioxidant_247
SNAKSEIGKYAPFFSLPNAKGEKITRSSDAFKQKSLLINFWASWNDSISQKQSNSELREIYK
KYKKNKYIGMLGISLDVDKQQWKDAIKRDTLDWEQVCDFGGLNSEVAKQYSIYKIPANI
LLSSDGKILAKNLRGEELKKKIENIVEEA
>antioxidant_248
SNAMTDAVLELPAATFDLPLSLSGGTQTTLRAHAGHWLVIYFYPKDSTPGCTTEGLDFNA
LLPEFDKAGAKILGVSRDSVKSHDNFCAKQGFAFPLVSDGDEALCRAFDVIKEKNMYGK
QVLGIERSTFLLSPEGQVVQAWRKVKVAGHADAVLAALKAHAKQ
>antioxidant_249
SNAPRAAGHSEEVLEREASFSLTTIDGEVISLNNVGGDVVILWFMAAWCPSCVYMADLLD
RLTEKYREISVIAIDFWTAEALKALGLNKPGYPPPDTPEMFRKFIANYGDPSWIMVMDDGS
LVEKFNVRSIDYIVIMDKSSNVLYAGTTPSLGELESVIKSVQGG
>antioxidant_250
SNAPLLLGKKAPNLYMTDTTGTYRYLYDVQAKYTILFFWDSQCGHCQQETPKLYDWWL
KNRAKGIQVYAANIERKDEEWLKFIRSKKIGGWLNVRDSKNHTDFKITYDIYATPVLYVL
DKNKVIIAKRIGYENLDDFLVQYEKSLKTK
>antioxidant_251
MSLAPGKAESDAPLVRTGALAPNFKLPTLSGENKSLAQYRGKIVLVNFWASWCPYCRDE

MPSMDRLVKSFPKGDLVVLAVNVEKRFPEKYRRAPVSFNFLSDATGQVQQRYGANRLPD
TFIVDRKGIIRQRVTGGIEWDAPKVVSYLKSLEGHHHHHH
>antioxidant_252
MSLAVKPGEPLPDFLLLDPKGQPVTPATVSKPAVIVFWASWCTVCKAEFPGLHRVAEETGV
PFYVISREPRDTREVVLEYMKTYPRFIPLLASDRDRPHEVAARFKVLGQPWTFVVDREGK
VVALFAGRAGREALLDALLLAGADLEGHHHHHH
>antioxidant_253
MSLEENPAPDFTLNTLNGEVVKLSDLKGQVVIVNFWATWCPPCREEIPSMMRLNAAMAG
KPFRMLCVSIDEGGKVAVEEFFRKTGFTLPVLLDADKRVGKLYGTTGVPETFVIDRHGVIL
KKVVGAMEWDHPEVIAFLNNELSKAREGHHHHHH
>antioxidant_254
EKKSINECDLKGKKVLIRVDFNVPVKNGKITNDYRIRSALPTLKKVLTEGGSCVLMSHLGR
PKGIPMAQAGKIRSTGGVPGFQQKATLKPVAKRLSELLLRPVTFAPDCLNAADVVSKMSP
GDVVLLENVRFYKEEGSKKAKDREAMAKILASYGDVYISDAFGTAHRDSATMTGIPKILG
NGAAGYLMEKEISYFAKVLGNPPRPLVAIVGGAKVSDKIQLLDNMLQRIDYLLIGGAMAY
TFLKAQGYSIGKSKCEESKLEFARSLLKKAEDRKVQVILPIDHVCHTEFKAVDSPLITEDQN
IPEGHMALDIGPKTIEKYVQTIGKCKSAIWNGPMGVFEMVPYSKGTFAIAKAMGRGTHEH
GLMSIIGGGDSASAAELSGEAKRMSHVSTGGGASLELLEGKTLPGVTVLDDK

The negative dataset contains 1567 non-antioxidant proteins
>non-antioxidant_1
MERPYACPVESCDRRFSRSADLTRHIRIHTGQKPFQCRICMRNFSRSDHLTTHIRTHTGEKP
FACDICGRKFARSDERKRHTKIHLRQKD
>non-antioxidant_2
MAPAEILNGKEISAQIRARLKNQVTQLKEQVPGFTPRLAILQVGNRDDSNLYINVKLKAAE
EIGIKATHIKLPRTTTESEVMKYITSLNEDSTVHGFLVQLPLDSENSINTEEVINAIAPEKDV
DGLTSINAGRLARGDLNDCFIPCTPKGCLELIKETGVPIAGRHAVVVGRSKIVGAPMHDLL
LWNNATVTTCHSKTAHLDEEVNKGDILVVATGQPEMVKGEWIKPGAIVIDCGINYVPDDK
KPNGRKVVGDVAYDEAKERASFITPVPGGVGPMTVAMLMQSTVESAKRFLEKFKPG
>non-antioxidant_3
MNLTELKNTPVSELITLGENMGLENLARMRKQDIIFAILKQHAKSGEDIFGDGVLEILQDG
FGFLRSADSSYLAGPDDIYVSPSQIRRFNLRTGDTISGKIRPPKEGERYFALLKVNEVNFDK
PENARNK
>non-antioxidant_4
MKNLDCWVDNEEDIDVILKKSTILNLDINNDIISDISGFNSSVITYPDAQLVPGINGKAIHLV
NNESSEVIVHKAMDIEYNDMFNNFTVSFWLRVPKVSASHLEQYGTNEYSIISSMKKHSLSI
GSGWSVSLKGNNLIWTLKDSAGEVRQITFRDLPDKFNAYLANKWVFITITNDRLSSANLYI
NGVLMGSAEITGLGAIREDNNITLKLDRCNNNNQYVSIDKFRIFCKALNPKEIEKLYTSYLS
ITFLRDFWGNPLRYDTEYYLIPVASSSKDVQLKNITDYMYLTNAPSYTNGKLNIYYRRLYN
GLKFIIKRYTPNNEIDSFVKSGDFIKLYVSYNNNEHIVGYPKDGNAFNNLDRILRVGYNAP
GIPLYKKMEAVKLRDLKTYSVQLKLYDDKNASLGLVGTHNGQIGNDPNRDILIASNWYFN
HLKDKILGCDWYFVPTDEGWTND
>non-antioxidant_5
MFKVYGYDSNIHKCGPCDNAKRLLTVKKQPFEFINIMPEKGVFDDEKIAELLTKLGRDTQI

GLTMPQVFAPDGSHIGGFDQLREYFK
>non-antioxidant_6
WSAEDKHKEGVNSHLWIVNRAIDIMSRNTTLVKQDRVAQLNEWRTELENGIYAADYENP
YYDNSTFASHFYDPDNGKTYIPFAKQAKETGAKYFKLAGESYKNKDMKQAFFYLGLSLH
YLGDVNQPMHAANFTNLSYPQGFHSKYENFVDTIKDNYKVTDGNGYWNWKGTNPEEW
IHGAAVVAKQDYSGIVNDNTKDWFVKAAVSQEYADKWRAEVTPMTGKRLMDAQRVTA
GYIQLWFDTYGDR
>non-antioxidant_7
VKDGYIVDDVNCTYFCGRNAYCNEECTKLKGESGYCQWASPYGNACYCYKLPDHVRTK
GPGRCH
>non-antioxidant_8
EYFTLQIRGRERFEMFRELNEALELKDAQAG
>non-antioxidant_9
LEPRHAMLLRCRLPGGVITTKQWQAIDKFAGENTIYGSIRLTNRQTFQFHGILKKNVKPVH
QMLHSVGLDALATANDMNRNVLCTSNPYESQLHAEAYEWAKKISEHLLPRTRAYAEIWL
DQEKVATTDEEPILGQTYLPRKFKTTVVIPPQNDIDLHANDMNFVAIAENGKLVGFNLLVG
GGLSIEHGNKKTYARTASEFGYLPLEHTLAVAEAVVTTQRDWGNRTDRKNAKTKYTLERV
GVETFKAEVERRAGIKFEPIRPYEFTGRGDRIGWVKGIDDNWHLTLFIENGRILDYPARPLK
TGLLEIAKIHKGDFRITANQNLIIAGVPESEKAKIEKIAKESGLMNAVTPQRENSMACVSFP
TCPLAMAEAERFLPSFIDNIDNLMAKHGVSDEHIVMRVTGCPNGCGRAMLAEVGLVGKA
PGRYNLHLGGNRIGTRIPRMYKENITEPEILASLDELIGRWAKEREAGEGFGDFTVRAGIIR
PVLDPARDLWD
>non-antioxidant_10
GVSGSCNIDVVCPEGDGRRDIIRAVGAYSKSGTLACTGSLVNNTANDRKMYFLTAHHCGM
GTASTAASIVVYWNYQNSTCRAPNTPASGANGDGSMSQTQSGSTVKATYATSDFTLLELN
NAANPAFNLFWAGWDRRDQNYPGAIAIHHPNVAEKRISNSTSPTSFVAWGGGAGTTHLNV
QWQPSGGVTEPGSSGSPIYSPEKRVLGQLHGGPSSCSATGTNRSDQYGRVFTSWTGGGAA
ASRLSDWLDPASTGAQFIDGLDSGGGTP
>non-antioxidant_11
ELKVVTATNFLGTLEQLAGQFAKQTGHAVVISSGSSGPVYAQIVNGAPYNVFFSADEKSPE
KLDNQGFALPGSRFTYAIGKLVLWSAKPGLVDNQGKVLAGNGWRHIAISNPQIAPYGLAG
TQVLTHLGLLDKLTAQERIVEANSVGQAHSQTASGAADLGFVALAQIIQAAAKIPGSHWFP
PANYYEPIVQQAVITKSTAEKANAEQFMSWMKGPKAVAIIKAAGYVLPQ
>non-antioxidant_12
PYSSDTTPCCFAYIARPLPRAHIKEYFYTSGKCSNPAVVFVTRKNRQVCANPEKKWVREYI
NSLEMS
>non-antioxidant_13
MISDSMTVEEIRLHLGLALKEKDFVVDKTGVKTIEIIGASFVADEPFIFGALNDEYIQRELE
WYKSKSLFVKDIPGETPKIWQQVASSKGEINSNYGWAIWSEDNYAQYDMCLAELGQNPD
SRRGIMIYTRPSMQFDYNKDGMSDFMCTNTVQYLIRDKKINAVVNMRSNDVVFGFRNDY
AWQKYVLDKLVSDLNAGDSTRQYKAGSIIWNVGSLHVYSRHFYLVDHWWKTGETHISK
KDYVGKYA
>non-antioxidant_14
MNKKELIDRVAKKAGAKKKDVKLILDTILETITEALAKGEKVQIVGFGSFEVRKAAARKG

VNPQTRKPITIPERKVPKFKPGKALKEKVK
>non-antioxidant_15
GGSQWNQVQQLEIKFLEQVDQFYDDNFPMEIRHLLAQWIETQDWEVASNNETMATILLQ
NLLIQLDEQLGRVSKEKNLLLIHNLKRIRKVLQGKFHGNPMHVAVVISNCLREERRILAAA
NMPI
>non-antioxidant_16
KKSAKDALLLWCQMKTAGYPNVNIHNFTTSWRDGMAFNALIHKHRPDLIDFDKLKKSN
AHYNLQNAFNLAEQHLGLTKLLDPEDISVDHPDEKSIITYVVTYYHYFSKM
>non-antioxidant_17
TQGNTCGGETCSAAQVCLKGKCVCNEVHCRIRCKYGLKKDENGCEYPCSCAKASQ
>non-antioxidant_18
SKRYFVTGTDTEVGKTVASCALLQAAKAAGYRTAGYKPVASGSEKTPEGLRNSDALALQ
RNSSLQLDYATVNPYTFAEPTSPHIISAQEGRPIESLVMSAGLRALEQQADWVLVEGAGGW
FTPLSDTFTFADWVTQEQLPVILVVGVKLGCINHAMLTAQVIQHAGLTLAGWVANDVTPP
GKRHAEYMTTLTRMIPAPLLGEIPWLAENPENAATGKYINLALL
>non-antioxidant_19
SIDSALNWDGEMTVTRFDAMTGAHFVIRLDSTQLGPAAGGTRAAQYSNLADALTDAGKL
AGAMTLKMAVSNLPMGGGKSVIALPAPRHSIDPSTWARILRIHAENIDKLSGNYWTGPDV
NTNSADMDTLNDTTEFVFGRSLERGGAGSSAFTTAVGVFEAMKATVAHRGLGSLDGLTVL
VQGLGAVGGSLASLAAEAGAQLLVADTDTERVAHAVALGHTAVALEDVLSTPCDVFAPCA
MGGVITTEVARTLDCSVVAGAANNVIADEAASDILHARGILYAPDFVANAGGAIHLVGREV
LGWSESVVHERAVAIGDTLNQVFEISDNDGVTPDEAARTLAGRRAREASTTTATA
>non-antioxidant_20
MIKLRMPAGGERYIDGKSVYKLYLMIKQHMNGKYDVIKYNWCMRVSDAAYQKRRDKYF
FQKLSEKYKLKELALIFISNLVANQDAWIGDISDADALVFYREYIGRLKQIKFKFEEDIRNIY
YFSKKVEVSAFKEIFEYNPKVQSSYIFKLLQSNIISFETFILLDSFLNIIDKHDEQTDNLVWN
NYSIKLKAYRKILNIDSQKAKNVFIETVKSCKY
>non-antioxidant_21
TPDCVTGKVEYTKYNDDDTFTVKVGDKELATNRANLQSLLLSAQITGMTVTIKTNACHN
GGGFSEVIFR
>non-antioxidant_22
SDPAHTATAPGGLSAKAPAMTPLMLDTSSRKLVAWDGTTDGAAVGILAVAADQTSTTLTFY
KSGTFRYEDVLWPEAASDETKKRTAFAGTAISIV
>non-antioxidant_23
VDAEAVVQQKCISCHGGDLTGASAPAIDKAGANYSEEEILDIILNGQGGMPGGIAKGAEAE
AVAAWLAEKK
>non-antioxidant_24
TVTVTYDPSNAPSFQQEIANAAQIWNSSVRNVQLRAGGNADFSYYEGNDSRGSYAQTDG
HGRGYIFLDYQQNQQYDSTRVTAHETGHVLGLPDHYQGPCSELMSGGGPGPSCTNPYPN
AQERSRVNALWANG
>non-antioxidant_25
MAEIKHYQFNVVMTCSGCSGAVNKVLTKLEPDVSKIDISLEKQLVDVYTTLPYDFILEKIK
KTGKEVRSGKQL
>non-antioxidant_26

MEKKTIVLGVIGSDCHAVGNKILDHAFTNAGFNVVNIGVLSPQELFIKAAIETKADAILVSS
LYGQGEIDCKGLRQKCDEAGLEGILLYVGGNIVVGKQHWPDVEKRFKDMGYDRVYAPGT
PPEVGIADLKKDLNIE
>non-antioxidant_27
MELKNKKWTDEEFHKQREEVLQQWPTGKEVDLQEAVDYLKKIPAEKNFAEKLVLAKKK
GITMAQPRAGVALLDEHIELLRYLQDEGGADFLPSTIDAYTRQNRYDECENGIKESEKAGR
SLLNGFPGVNFGVKGCRKVLEAVNLPLQARHGTPDSRLLAEIIHAGGWTSNEGGGISYNV
PYAKNVTIEKSLLDWQYCDRLVGFYEEQGVHINREPFGPLTGTLVPPSMSNAVGITEALLA
AEQGVKNITVGYGECGNMIQDIAALRCLEEQTNEYLKAYGYNDVFVTTVFHQWMGGFP
QDESKAFGVIVTATTIAALAGATKVIVKTPHEAIGIPTKEANAAGIKATKMALNMLEGQRM
PMSKELETEMAVIKAETKCILDKMFELGKGDLAIGTVKAFETGVMDIPFGPSKYNAGKM
MPVRDNLGCVRYLEFGNVPFTEEIKNYNRERLQERAKFEGRDVSFQMVIDDIFAVGKGRL
IGRPE
>non-antioxidant_28
VLSSQNKKAIEELGNLIKANAEAWGADALARLFELHPQTKTYFSKFSGFEACNEQVKKHG
KRVMNALADATHHLDNLHLHLEDLARKHGENLLVDPHNFHLFADCIVVTLAVNLQAFTP
VTHCAVDKFLELVAYELSSCYR
>non-antioxidant_29
PLREGRGLGPLQIWQTDFTLEPRMAPRSWLAVTVDTASSAIVVTQHGRVTSVAAQHHWAT
AIAVLGRPKAIKTDNGSCFTSKSTREWLARWGIAHTTGIPGNSQGQAMVERANRLLKDKI
RVLAEGDGFMKRIPTSKQGELLAKAMYALNHFERGENTKTNL
>non-antioxidant_30
MDAKARNCLLQHREALEKDIKTSYIMDHMISDGFLTISEEEKVRNEPTQQQRAAMLIKMI
LKKDNDSYVSFYNALLHEGYKDLAALLHDGIPVVSSS
>non-antioxidant_31
ATFKVTLINEAEGTKHEIEVPDDEYILDAAEEQGYDLPFSCRAGACSTCAGKLVSGTVDQS
DQSFLDDDQIEAGYVLTCVAYPTSDVVIQTHKEEDLY
>non-antioxidant_32
MEISGTHTEINLDNAIDMIREANSIIITPGYGLCAAKAQYPIADLVKMLSEQGKKVRFGIHP
VAGRMPGQLNVLLAEAGVPYDIVLEMDEINHDFPDTDLVLVIGANDTVNSAAQEDPNSII
AGMPVLEVWKSKQVIVMKRSLGVGYAAVDNPIFYKPNTAMLLGDAKKTCDALQAKVRE
SYQK
>non-antioxidant_33
HHMDEEYDVIVLGTGLTECILSGIMSVNGKKVLHMDRNPYYGGESSSITPLEELYKRFQLL
EGPPETMGRGRDWNVDLIPKFLMANGQLVKMLLYTEVTRYLDFKVVEGSFVYKGGKIYK
VPSTETEALASNLMGMFEKRRFRKFLVFVANFDENDPKTFEGVDPQNTSMRDVYRKFDL
GQDVIDFTGHALALYRTDDYLDQPCLETINRIKLYSESLARYGKSPYLYPLYGLGELPQGFA
RLSAIYGGTYMLNKPVDDIIMENGKVVGVKSEGEVARCKQLICDPSYVPDRVRKAGQVIR
IICILSHPIKNTNDANSCQIIIPQNQVNRKSDIYVCMISYAHNVAAQGKYIAIASTTVETTDPE
KEVEPALGLLEPIDQKFVAISDLYEPIDDGSESQVFCSCSYDATTHFETTCNDIKDIYKRMA
GSAFDF
>non-antioxidant_34
LNSCSMPLGMESKAISDAQITASSYFTNMFATWSPSKARLHLQGRSNAWRPQVNNPKEWL
QVDFQKTMKVTGVTTQGVKSLLTSMYVKEFLISSSQDGHQWTLFFQNGKVKVFQGNQD

SFTPVVNCLDPPLLTRYLRIHPQSWVHQIALRMEVLGCEAQ
>non-antioxidant_35
GHHHHHHMTTQLEQAWELAKQRFAAVGIDVEEALRQLDRLPVSMHCWQGDDVSGFENP
EGSLTGGIQATGNYPGKARNASELRADLEQAMRLIPGPKRLNLHAIYLESDTPVSRDQIKP
EHFKNWVEWAKANQLGLDFNPSCFSHPLSADGFTLSHADDSIRQFWIDHCKASRRVSAYF
GEQLGTPSVMNIWIPDGMKDITVDRLAPRQRLLAALDEVISEKLNPAHHIDAVESKLFGIG
AESYTVGSNEFYMGYATSRQTALCLDAGHFHPTEVISDKISAAMLYVPQLLLHVSRPVRW
DSDHVVLLDDETQAIASEIVRHDLFDRVHIGLDFFDASINRIAAWVIGTRNMKKALLRALL
EPTAELRKLEAPGDYTARLALLEEQKSLPWQAVWEMYCQRHDTPAGSEWLESVRAYEKE
ILSRRG
>non-antioxidant_36
MRGSHHHHHHGSGSMHQRQTLYQLMDGLNTFYQQSLQQPVATSARQYLEKRGLSHEVI
ARFAIGFAPPGWDNVLKRFGGNPENRQSLIDAGMLVTNDQGRSYDRFRERVMFPIRDKRG
RVIGFGGRVLGNDTPKYLNSPETDIFHKGRQLYGLYEAQQDNAEPNRLLVVEGYMDVVAL
AQYGINYAVASLGTSTTADHIQLLFRATNNVICCYDGDRAGRDAAWRALETALPYMTDGR
QLRFMFLPDGEDPDTLVRKEGKEAFEARMEQAMPLSAFLFNSLMPQVDLSTPDGRARLST
LALPLISQVPGETLRIYLRQELGNKLGILDDSQLERLMPK
>non-antioxidant_37
SGIVQQQNNLLRAIEAQQHLLQLTVWGIKQLQARSGGRGGWMEWDREINNYTSLIHSLIE
ESQNQQEK
>non-antioxidant_38
MKIDITDYNHADEILNPQLWKEIEETLLKMPLHVKASDQASKVGSLIFDPVGTNQYIKDEL
VPKHWKNNIPIPKRFDFLGTDIDFGKRDTLVEVQFSNYPFLLNNTVRSELFHKSNMDIDEE
GMKVAIIITKGHMFPASNSSLYYEQAQNQLNSLAEYNVFDVPIRLVGLIEDFETDIDIVSTTY
ADKRYSRTITKRDTVKGKVIDTNTPNTRRRKRGTIVTY
>non-antioxidant_39
MILRTSEETISTVQEKQQNISPLVRERGPQRVAAHITGTRGRSNTLSSPNSKNEKALGRKINS
WESSRSGHSFLSNLHLRNGELVIHEKGFYYIYSQTYFRFQEEIKENTKNDKQMVQYIYKYT
SYPAPILLMKSARNSCWSKDAEYGLYSIYQGGIFELKENDRIFVSVTNEHLIDMDHEASFF
GAFLVG
>non-antioxidant_40
PPVYKIALGIEYDGSKYYGWQRQNEVRSVQEKLEKALSQVANEPITVFCAGRTDAGVHG
TGQVVHFETTALRKDAAWTLGVNANLPGDIAVRWVKTVPDDFHARFSATARRYRYIIYNH
RLRPAVLSKGVTHFYEPLDAERMHRAAQCLLGENDFTSFRAVQCQSRTPWRNVMHINVT
RHGPYVVVDIKANAFVHHMVRNIVGSLMEVGAHNQPESWIAELLAAKDRTLAAATAKA
EGLYLVAVDYPDRYDLPKPPMGPLFLAD
>non-antioxidant_41
GSASPTPPYLKWAESLHSLLDDQDGISLFRTFLKQEGCADLLDFWFACTGFRKLEPCDSNE
EKRLKLARAIYRKYILDNNGIVSRQTKPATKSFIKGCIMKQLIDPAMFDQAQTEIQATMEEN
TYPSFLKSDIYLEYTRTGSESPKV
>non-antioxidant_42
CGUTACCAUGGTAACG
>non-antioxidant_43
MTSVDCTAYGPELRALAARLPRTPRADLYAFLDAAHTAAASLPGALATALDTFNAEGSED

GHLLLRGLPVEADADLPTTPSSTPAPEDRSLLTMEAMLGLVGRRLGLHTGYRELRSGTVY
HDVYPSPGAHHLSSETSETLLEFHTEMAYHRLQPNYVMLACSRADHERTAATLVASVRKA
LPLLDERTRARLLDRRMPCCVDVAFRGGVDDPGAIAQVKPLYGDADDPFLGYDRELLAPE
DPADKEAVAALSKALDEVTEAVYLEPGDLLIVDNFRTTHARTPFSPRWDGKDRWLHRVYI
RTDRNGQLSGGERAGDVVAFTPRG
>non-antioxidant_44
KETAAAKFERQHMDSSTSAASSSNYCNQMMKSRNLTKDRCKPVNTFVHESLADVQAVCS
QKNVACKDGQTNCYQSYSTMSITDCRETGSSKYPNCAYKTTQANKHIIVACEGNPYVPVH
FDASV
>non-antioxidant_45
QEPQPEQDPFELSGKWITSYIGSSDLEKIGENAPFQVFMRSIEFDDKESKVYLNFFSKENGI
CEEFSLIGTKQEGNTYDVNYAGNNKFVVSYASETALIISNINVDEEGDKTIMTGLLGKGTDI
EDQDLEKFKEVTRENGIPEENIVNIIERDDCPA
>non-antioxidant_46
MGKRVVIALGGNALQQRGQKGSYEEMMDNVRKTARQIAEIIARGYEVVITHGNGPQVGS
LLLHMDAGQATYGIPAQPMDVAGAMSQGWIGYMIQQALKNELRKRGMEKKVVTIITQTI
VDKNDPAFQNPTKPVGPFYDEETAKRLAREKGWIVKEDSGRGWRRVVPSPDPKGHVEAE
TIKKLVERGVIVIASGGGGVPVILEDGEIKGVEAVIDKDLAGEKLAEEVNADIFMILTDVNG
AALYYGTEKEQWLREVKVEELRKYYEEGHFKAGSMGPKVLAAIRFIEWGGERAIIAHLE
KAVEALEGKTGTQVLP
>non-antioxidant_47
VELTESTRTIPLDEAGGTTTLTARQFTNGQKIFVDTCTQCHLQGKTKTNNNVSLGLADLAG
AEPRRDNVLALVEFLKNPKSYDGEDDYSELHPNISRPDIYPEMRNYTEDDIFDVAGYTLIA
PKLDERWGGTIYF
>non-antioxidant_48
YPVFAQQNYANPREANGRIVCANCHLAQKAVEIEVPQAVLPDTVFEAVIELPYDKQVKQV
LANGKKGDLNVGMVLILPEGFELAPPDRVPAEIKEKVGNLYYQPYSPEQKNILVVGPVPG
KKYSEMVVPILSPDPAKNKNVSYLKYPIYFGGNRGRGQVYPDGKKSNFTIYNASAAGKIV
AITALSEKKGGFEVSIEKANGEVVVDKIPAGPDLIVKEGQTVQADQPLTNNPNVGGFGQA
ETEIVLQNPAR
>non-antioxidant_49
AVTKLVLVRHGESQWNKENRFTGWYDVDLSEKGVSEAKAAGKLLKEEGYSFDFAYTSVL
KRAIHTLWNVLDELDQAWLPVEKSWKLNERHYGALQGLNKAETAEKYGDEQVKQWRR
GFAVTPPELTKDDERYPGHDPRYAKLSEKELPLTESLALTIDRVIPYWNETILPRMKSGERVI
IAAHGNSLRALVKYLDNMSEEEILELNIPTGVPLVYEFDENFKPLKRYYLGNADEIAAKAA
AVANQGKAK
>non-antioxidant_50
MNLMTTITGVVLAGGKARRMGGVDKGLLELNGKPLWQHVADALMTQLSHVVVNANRH
QEIYQASGLKVIEDSLADYPGPLAGMLSVMQQEAGEWFLFCPCDTPYIPPDLAARLNHQR
KDAPVVWVHDGERDHPTIALVNRAIEPLLLEYLQAGERRVMVFMRLAGGHAVDFSDHK
DAFVNVNTPEELARWQEKRSHHHHHH
>non-antioxidant_51
MLLTGKLYKEEKQKFYDAQNGKCLICQRELNPDVQANHLDHDHELNGPKAGKVRGLLC
NLCDAAEGQMKHKFNRSGLKGQGVDYLEWLENLLTYLKSDYTQNNIHPNFVGDKSKEF

SRLGKEEMMAEMLQRGFEYNESDTKTQLIASFKKQLRKSLK
>non-antioxidant_52
SGDRSMVEVLADHPGELVRTDSPNFLSSVLPTHWRSNKTLPIAFKVVALGDVPDGTLVTV
MAGNDENYSAELRNATAAMKNQVARFNDLRFVGRSGRGKSFTLTITVFTNPPQVATYHR
AIKITVDGPREPRRHRQKLDD
>non-antioxidant_53
TEVTDCKGDAESSLTTALSNAAKLANQAAEAAESGDESKFEEYFKTTDQQTRTTVAERLR
AVAKEAGSTSGGSTTYHCNDPYGYCEPNVLAYTLPSKNEIANCDIYYSELPPLAQKCHAQ
DQATTTLHEFTHAPGVYQPGTEDLGYGYDAATQLSAQDALNNADSYALYANAIELKC
>non-antioxidant_54
LSADQISTVQASFDKVKGDPVGILYAVFKADPSIMAKFTQFAGKDLESIKGTAPFETHANRI
VGFFSKIIGELPNIEADVNTFVASHKPRGVTHDQLNNFRAGFVSYMKAHTDFAGAEAAWG
ATLDTFFGMIFSKM
>non-antioxidant_55
MYDASLIPNLQIPQKNIPNNDGMNFVKGLRLGWNLGNTFDAFNGTNITNELDYETSWSGI
KTTKQMIDAIKQKGFNTVRIPVSWHPHVSGSDYKISDVWMNRVQEVVNYCIDNKMYVIL
NTHHDVDKVKGYFPSSQYMASSKKYITSVWAQIAARFANYDEHLIFEGMNEPRLVGHAN
EWWPELTNSDVVDSINCINQLNQDFVNTVRATGGKNASRYLMCPGYVASPDGATNDYFR
MPNDISGNNNKIIVSVHAYCPWNFAGLAMADGGTNAWNINDSKDQSEVTWFMDNIYNK
YTSRGIPVIIGECGAVDKNNLKTRVEYMSYYVAQAKARGILCILWDNNNFSGTGELFGFFD
RRSCQFKFPEIIDGMVKYAFGLIN
>non-antioxidant_56
VDGDQCESNPCLNGGSCKDDINSYECWCPFGFEGKNCEL
>non-antioxidant_57
GRKKIQITRIMDERNRQVTFTKRKFGLMKKAYELSVLCDCEIALIIFNSSNKLFQYASTDM
DKVLLKYTEYNEPHES
>non-antioxidant_58
TTCCPSIVARSNFNVCRLPGTPEALCATYTGCIIIPGATCPGDYAN
>non-antioxidant_59
MDAQSAAKCLTAVRRHSPLVHSITNNVVTNFTANGLLALGASPVMAYAKEEVADMAKIA
GALVLNIGTLSKESVEAMIIAGKSANEHGVPVILDPVGAGATPFRTESARDIIREVRLAAIR
GNAAEIAHTVGVTDWLIKGVDAGEGGGDIIRLAQQAAQKLNTVIAITGEVDVIADTSHVY
TLHNGHKLLTKVTGAGCLLTSVVGAFCAVEENPLFAAIAAISSYGVAAQLAAQQTADKGP
GSFQIELLNKLSTVTEQDVQEWATIERVTVS
>non-antioxidant_60
GAMGSDFLLGNPFSSPVGQRIEKATDGSLQSEDWALNMEICDIINETEEGPKDALRAVKKR
IVGNKNFHEVMLALTVLETCVKNCGHRFHVLVASQDFVESVLVRTILPKNNPPTIVHDKVL
NLIQSWADAFRSSPDLTGVVTIYEDLRRKGLEFPM
>non-antioxidant_61
MTMITPSLHQFPGLANKTYFNFGGQGILPTVALEAITAMYGYLQENGPFSIAANQHIQQLI
AQLRQALAETFNVDPNTITITDNVTTGCDIVLWGLDWHQGDEILLTDCEHPGIIAIVQAIAA
RFGITYRFFPVAATLNQGDAAAVLANHLGPKTRLVILSHLLWNTGQVLPLAEIMAVCRRH
QGNYPVRVLVDGAQSAGSLPLDFSRLEVDYYAFTGHKWFAGPAGVGGLYIHGDCLGEINP
TYVGWRSITYGAKGEPTGWAEGGKRFEVATSAYPQYAGLLAALQLHQRQGTAEERYQAI

CQRSEFLWRGLNQLPHVHCLATSAPQAGLVSFTVDSPLGHRAIVQKLEEQRIYLRTIADPD
CIRACCHYITDEEEINHLLARLADFGP
>non-antioxidant_62
MSGDENPASKPTPVQDVQGDGKWMSLHHRFVADSKDKEPEVVFIGDSLVQLMHQCEIW
RELFSPLHALNFGIGGDSTQHVLWRLENGELEHIRPKIVVVWVGTNNHGHTAEQVTGGIK
AIVQLVNERQPQARVVVLGLLPRGQHPNPLREKNRRVNELVRAALAGHPRAHFLDADPG
FVHSDGTISHHDMYDYLHLSRLGYTPVCRALHSLLLRLLTQDQGQGGAPLPEPSP
>non-antioxidant_63
SVSEIQLMHNLGKHLNSMERVEWLRKKLQDVHNF
>non-antioxidant_64
GSLVPELNEKDDDQVQKALASRENTQLMNRDNIEITVRDFKTLAPRRWLNDTIIEFFMKYI
EKSTPNTVAFNSFFYTNLSERGYQGVRRWMKRKKTQIDKLDKIFTPINLNQSHWALGIIDL
KKKTIGYVDSLSNGPNAMSFAILTDLQKYVMEESKHTIGEDFDLIHLDCPQQPNGYDCGIY
VCMNTLYGSADAPLDFDYKDAIRMRRFIAHLILTDALK
>non-antioxidant_65
MMKKIDVKILDPRVGKEFPLPTYATSGSAGLDLRACLNDAVELAPGDTTLVPTGLAIHIAD
PSLAAMMLPRSGLGHKHGIVLGNLVGLIDSDYQGQLMISVWNRGQDSFTIQPGERIAQMI
FVPVVQAEFNLVEDFDATDRGEGGFGHSGRQ
>non-antioxidant_66
RDHRKIGKQLDLYHMQEEAPGMVFWHNDGWTIFRELEVFVRSKLKEYQYQEVKGPFMM
DRVLWEKTGHWDNYKDAMFTTSSENREYCIKPMNCPGHVQIFNQGLKSYRDLPLRMAE
FGSCHRNEPSGSLHGLMRVRGFTQDDAHIFCTEEQIRDEVNGCIRLVYDMYSTFGFEKIVV
KLSTRPEKRIGSDEMWDRAEADLAVALEENNIPFEYQLGEGAFYGPKIEFTLYDCLDRAW
QCGTVQLDFSLPSRLSASYVGEDNERKVPVMIHRAILGSMERFIGILTEEFAGFFPTWLAPV
QVVIMNITDSQSEYVNELTQKLSNAGIRVKADLRNEKIGFKIREHTLRRVPYMLVCGDKEV
ESGKVAVRTRRGKDLGSMDVNEVIEKLQQEIRSRSLKQLEE
>non-antioxidant_67
MNDSEFHRLADQLWLTIEERLDDWDGDSDIDCEINGGVLTITFENGSKIIINRQEPLHQVW
LATKQGGYHFDLKGDEWICDRSGETFWDLLEQAATQQAGETVSFR
>non-antioxidant_68
HNYSEAEIKVREATSNDPWGPSSSLMSEIADLTYNVVAFSEIMSMIWKRLNDHGKNWRHV
YKAMTLMEYLIKTGSERVSQQCKENMYAVQTLKDFQYVDRDGKDQGVNVREKAKQLVA
LLRDEDRLREERAHALKTKEKLAQTA
>non-antioxidant_69
QCTGGADCTSCTGACTGCGNCPNAVTCTNSQHCVKANTCTGSTDCNTAQTCTNSKDCFE
ANTCTDSTNCYKATACTNSSGCPGH
>non-antioxidant_70
MAVELPKAAIERIFRQGIGERRLSQDAKDTIYDFVPTMAEYVANAAKSVLDASGKKTLME
EHLKALADVLMVEGVEDYDGELFGRATVRRILKRAGIERASSDAVDLYNKLICRATEELG
EKAAEYADEDGRKTVQGEDVEKAITYSMPKGGEL
>non-antioxidant_71
KRKEAVIIMNVAAHHGSELNGELLLNSIQQAGFIFGDMNIYHRHLSPDGSGPALFSLANMV
KPGTFDPEMKDFTTPGVTIFMQVPSYGDELQLFKLMLQSAQHIADEVGGVVLDDQRRM
MTPQKLREYQDIIREVKDANA

>non-antioxidant_72
GIYGIGLDITELKRIASMAGRQKRFAERILTRSELDQYYELSEKRKNEFLAGRFAAKEAFSK
AFGTGIGRQLSFQDIEIRKDQNGKPYIICTKLSPAAVHVSITHTKEYAAAQVVIERLSS
>non-antioxidant_73
CPLMVKVLDAVRGSPAINVAVHVFRKAADDTWEPFASGKTSESGELHGLTTEEEFVEGIYK
VEIDTKSYWKALGISPFHEHAEVVFTANDSGPRRYTIAALLSPYSYSTMAVVTN
>non-antioxidant_74
RDFNNLTKGLCTINSWHIYGKDNAVRIGEDSDVLVTREPYVSCDPDECRFYALSQGTTIRG
KHSNGTIHDRSQYRALISWPLSSPPTVYNSRVECIGWSSTSCHDGKTRMSICISGPNNNASA
VIWYNRRPVTEINTWARNILRTQESECVCHNGVCPVVFTDGSATGPAETRIYYFKEGKILK
WEPLAGTAKHIEECSCYGERAEITCTCRDNWQGSNRPVIRIDPVAMTHTSQYICSPVLTDN
PRPNDPTVGKCNDPYPGNNNNGVKGFSYLDGVNTWLGRTISIASRSGYEMLKVPNALTD
DKSKPTQGQTIVLNTDWSGYSGSFMDYWAEGECYRACFYVELIRGRPKEDKVWWTSNSI
VSMCSSTEFLGQWDWPDGAKIEYFL
>non-antioxidant_75
MECYRCGVSGCHLKITCSAEETFCYKWLNKISNERWLGCAKTCTEIDTWNVYNKCCTTN
LCNT
>non-antioxidant_76
MRGNIRVYCRIRPALKNLENSDTSLINVNEFDDNSGVQSMEVTKIQNTAQVHEFKFDKIFD
QQDTNVDVFKEVGQLVQSSLDGYNVCIFAYGQTGSGKTFTMLNPGDGIIPSTISHIFNWIN
KLKTKGWDYKVNCEFIEIYNENIVDLLRSDNNNKEDTSIGLKHEIRHDQETKTTTITNVTS
CKLESEEMVEIILKKANKLRSTASTASNEHSSASHSIFIIHLSGSNAKTGAHSYGTLNLVDL
AGSERINVSQVVGDRLRETQNINKSLSCLGDVIHALGQPDSTKRHIPFRNSKLTYLLQYSLT
GDSKTLMFVNISPSSSHINETLNSLRFASKVNSTRLVSRK
>non-antioxidant_77
TPDNNKTVREFNVYWNVPTFMCHKYGLRFEEVSEKYGILQNWMDKFRGEEIAILYDPGM
FPALLKDPNGNVVARNGGVPQLGNLTKHLQVFRDHLINQIPDKSFPGVGVIDFESWRPIFR
QNWASLQPYKKLSVEVVRREHPFWDDQRVEQEAKRRFEKYGQLFMEETLKAAKRMRPA
ANWGYYAYPYCYNLTPNQPSAQCEATTMQENDKMSWLFESEDVLLPSVYLRWNLTSGER
VGLVGGRVKEALRIARQMTTSRKKVLPYYWYKYQDRRDTDLSRADLEATLRKITDLGAD
GFIIWGSSDDINTKAKCLQFREYLNNELGPAVKRIALNNNANDRLTVDVSVDQV
>non-antioxidant_78
GIGDPVTCLKSGAICHPVFCPRRYKQIGTCGLPGTKCCKKP
>non-antioxidant_79
MQPLFTQERRIFHKKLLDGNILATNNRGVVSNADGSNTRSFNIAKGIADLLHSETVSERLP
GQTSGNAFEAICSEFVQSAFEKLQHIRPGDWNVKQVGSRNRLEIARYQQYAHLTALAKAA
EENPELAAALGSDYTITPDIIVTRNLIADAEINRNEFLVDENIATYASLRAGNGNMPLLHASI
SCKWTIRSDRAQNARSEGLNLVRNRKGRLPHIVVVTAEPTPSRISSIALGTGEIDCVYHFAL
YELEQILQSLNYEDALDLFYIMVNGKRLKDISDLPLDLAV
>non-antioxidant_80
AISCGQVASAIAPCISYARGQGSGPSAGCCSGVRSLNNAARTTADRRAACNCLKNAAAGV
SGLNAGNAASIPSKCGVSIPYTISTSTDCSRVN
>non-antioxidant_81
MLPGTFFEVLKNEGVVAIATQGEDGPHLVNTWNSYLKVLDGNRIVVPVGGMHKTEANVA

RDERVLMTLGSRKVAGRNGPGTGFLIRGSAAFRTDGPEFEAIARFKWARAALVITVVSAEQ
TL
>non-antioxidant_82
MIKVLFFAQVRELVGTDATEVAADFPTVEALRQHMAAQSDRWALALEDGKLLAAVNQTL
VSFDHPLTDGDEVAFFPPVTGG
>non-antioxidant_83
MAETKIVVGPQPFSVGEEYPWLAERDEDGAVVTFTGKVRNHNLGDSVNALTLEHYPGMT
EKALAEIVDEARNRWPLGRVTVIHRIGELWPGDEIVFVGVTSAHRSSAFEAGQFIMDYLKT
RAPFWKREATPEGDRWVEARESDQQAAKRW
>non-antioxidant_84
LAVIPILVIACDRSTVRRCLDKLLHYRPSAELFPIIVSQDCGHEETAQVIASYGSAVTHIRQP
DLSNIAVQPDHRKFQGYYKIARHYRWALGQIFHNFNYPAAVVVEDDLEVAPDFFEYFQAT
YPLLKADPSLWCVSAWNDNGKEQMVDSSKPELLYRTDFFPGLGWLLLAELWAELEPKWP
KAFWDDWMRRPEQRKGRACVRPEISRTMTFGRKGVSHGQFFDQHLKFIKLNQQFVPFTQ
LDLSYLQQEAYDRDFLARVYGAPQLQVEKVRTNDRKELGEVRVQYTGRDSFKAFAKALG
VMDDLKSGVPRAGYRGIVTFLFRGRRVHLAPPQTWDGYDPSWT
>non-antioxidant_85
GSHMASKPIEDYGKGKGRIEPMYIPDNTFYNADDFLVPPHCKPYIDKILLPGGLVKDRVEK
LAYDIHRTYFGEELHIICILKGSRGFFNLLIDYLATIQKYSGRESSVPPFFEHYVRLKSYQND
NSTGQLTVLSDDLSIFRDKHVLIVEDIVDTGFTLTEFGERLKAVGPKSMRIATLVEKRTDRS
NSLKGDFVGFSIEDVWIVGCCYDFNEMFRDFDHVAVLSDAARKKFEK
>non-antioxidant_86
ADAPFEGRKKCSSCHKAQAQSWKDTAHAKAMESLKPNVKKEAKQKAKLDPAKDYTQD
KDCVGCHVDGFGQKGGYTIESPKPMLTGVGCESCHGPGRNFRGDHRKSGQAFEKSGKKT
PRKDLAKKGQDFHFEERCSACHLNYEGSPWKGAKAPYTPFTPEVDAKYTFKFDEMVKE
VKAMHEHYKLEGVFEGEPKFKFHDEFQASAKPAKKGK
>non-antioxidant_87
NNNRAPKEPTDPVTLIFTDIESSTALWAAHPDLMPDAVAAHHRMVRSLIGRYKCYEVKTV
GDSFMIASKSPFAAVQLAQELQLCFLHHDWGTNALDDSYREFEEQRAEGECEYTPPTAHM
DPEVYSRLWNGLRVRVGIHTGLCDIRHDEVTKGYDYYGRTPNMAARTESVANGGQVLMT
HAAYMSLSAEDRKQIDVTALGDVALRGVSDPVKMYQLNTVPSRNFAALRLDREYFD
>non-antioxidant_88
MELLLLSNSTLPGKAWLEHALPLIANQLNGRRSAVFIPFAGVTQTWDEYTDKTAEVLAPL
GVNVTGIHRVADPLAAIEKAEIIIVGGGNTFQLLKESRERGLLAPMADRVKRGALYIGWSA
GANLACPTIRTTNDMPIVDPNGFDALDLFPLQINPHFTNALPEGHKGETREQRIRELLVVAP
ELTVIGLPEGNWIQVSNGQAVLGGPNTTWVFKAGEEAVALEAGHRF
>non-antioxidant_89
GSHMKTRKIPLRKSVVSNEVIDKRDLLRIVKNKEGQVFIDPTGKANGRGAYIKLDNAEAL
EAKKKKVFNRSFSMEVEESFYDELIAYVDHKVKRRELGLE
>non-antioxidant_90
MVSKLSQLQTEMLAALLESGLSKEALIQALGE
>non-antioxidant_91
AETVESCLAKSHTENSFTNVWKDDKTLDRYANYEGCLWNATGVVVCTGDETQCYGTWV
PIGLAIPENEGGGSEGGGSEGGGSEGGGTKPPEYGDTPIPGYTYINPLDGTYPPGTEQNPAN

PNPSLEESQPLNTFMFQNNRFRNRQGALTVYTGTVTQGTDPVKTYYQYTPVSSKAMYDA
YWNGKFRDCAFHSGFNEDIFVCEYQGQSSDLPQPPVNA
>non-antioxidant_92
MGRKLELTKAEKHVHNFMMDTQLTKRVKNAAANVLRETWLIYKNTKLVKKIDHAKVRK
HQRKFLQAIHQLRSVKMEQRKLNDQANTLVDLAKTQLEHHHHH
>non-antioxidant_93
MTMIIRKYFSGIPTIGVLALTTEEITLLPIFLDKDDVNEVSEVLETKCLQTNIGGSSLVGSLSV
ANKYGLLLPKIVEDEELDRIKNFLKENNLDLNVEIIKSKNTALGNLILTNDKGALISPELKD
FKKDIEDSLNVEVEIGTIAELPTVGSNAVVTNKGCLTHPLVEDDELEFLKSLFKVEYIGKGT
ANKGTTSVGACIIANSKGAVVGGDTTGPELLIIEDALGLI
>non-antioxidant_94
SCPAIHVFGARETTASPGYGSSSTVVNGVLSAYPGSTAEAINYPACGGQSSCGGASYSSSVA
QGIAAVASAVNSFNSQCPSTKIVLVGYSQGGEIMDVALCGGGDPNQGYTNTAVQLSSSAVN
MVKAAIFMGDPMFRAGLSYEVGTCAAGGFDQRPAGFSCPSAAKIKSYCDASDPYCCNGS
NAATHQGYGSEYGSQALAFVKSKLG
>non-antioxidant_95
SLAALKSELQALKKEGFSPEELAALESELQALEKKLAALKSKLQALKG
>non-antioxidant_96
RPDFCLEPPYAGACRARIIRYFYNAKAGLCQTFVYGGCRAKRNNFKSAEDCLRTCGGA
>non-antioxidant_97
MVEVKKHKFPGVYTVIDDDGSERIATKNLVPGQRVYGERVIKWEGEEYRIWNPNRSKLG
AAIMNGLKNFPIKPGKSVLYLGIASGTTASHVSDIVGWEGKIFGIEFSPRVLRELVPIVEERR
NIVPILGDATKPEEYRALVPKVDVIFEDVAQPTQAKILIDNAEVYLKRGGYGMIAVKSRSID
VTKEPEQVFREVERELSEYFEVIERLNLEPYEKDHALFVVRKT
>non-antioxidant_98
FVNKDQIAKDVKQFYDQALQQAVVDDDANNAKAVVKTFHETLDCCGSSTLTALTTSVLK
NNLCPSGSNIISNLFKEDCHQKIDDLFSGKH
>non-antioxidant_99
RTDCYGNVNRIDTTGASCKTAKPEGLSYCGVPASKTIAERDLKAMDRYKTIIKKVGEKLC
VEPAVIAGIISRESHAGKVLKNGWGDRGNGFGLMQVDKRSHKPQGTWNGEVHITQGTTIL
TDFIKRIQKKFPSWTKDQQLKGGISAYNAGAGNVRSYARMDIGTTHDDYANDVVARAQY
YKQHGY
>non-antioxidant_100
KPSSPPEELKFQCGQKTLRPRFK
>non-antioxidant_101
GSNEKVELQELNDRFANYIDKVRFLEQQNKILLAELEQL
>non-antioxidant_102
EQSSSEIKIVRDEYGMPHIYANDTWHLFYGYGYVVAQDRLFQMEMARRSTQGTVAEVLG
KDFVKFDKDIRRNYWPDAIRAQIAALSPEDMSILQGYADGMNAWIDKVNTNPETLLPKQF
NTFGFTPKRWEPFDVAMIFVGTMANRFSDSTSEIDNLALLTALKDKYGVSQGMAVFNQLK
WLVNPSAPTTIAVQESNYPLKFNQQNSQTAALLPRYDLPAPMLDRPAKGADGALLALAAG
KNRETIAAQFAQGGANGLAGY
>non-antioxidant_103
SNMWVIGKSKAQDAKAIMVNGPQFGWYAPAYTYGIGLHGAGYDVTGNTPFAYPGLVFG

HNGVISWGSTAGFGDDVDIFAERLSAEKPGYYLHNGKWVKMLSREETITVKNGQAETFT
VWRTVHGNILQTDQTTQTAYAKSRAWDGKEVASLLAWTHQMKAKNWQEWTQQAAKQ
ALTINWYYADVNGNIGYVHTGAYPDRQSGHDPRLPVPGTGKWDWKGLLPFEMNPKVYN
PQSGYIANWNNSPQKDYPASDLFAFLWGGADRVTEIDRLLEQKPRLTADQAWDVIRQTSR
QDLNLRLFLPTLQAATSGLTQSDPRRQLVETLTRWDGINLLNDDGKTWQQPGSAILNVWL
TSMLKRTVVAAVPMPFDKWYSASGYETTQDGPTGSLNISVGAKILYEAVQGDKSPIPQAV
DLFAGKPQQEVVLAALEDTWETLSKRYGNNVSNWKTPAMALTFRANNFFGVPQAAAEE
TRHQAEYQNRGTENDMIVFSPTTSDRPVLAWDVVAPGQSGFIAPDGTVDKHYEDQLKMY
ENFGRKSLWLTKQDVEAHKESQEVLHVQR
>non-antioxidant_104
TELTLKPGTLTLAQLRAIHAAPVRLQLDASAAPAIDASVACVEQIIAEDRTAYGINTGFGLL
ASTRIASHDLENLQRSLVLSHAAGIGAPLDDDLVRLIMVLKINSLSRGFSGIRRKVIDALIAL
VNAEVYPHIPLKGSVGASGDLAPLAHMSLVLLGEGKARYKGQWLSATEALAVAGLEPLTL
AAKEGLALLNGTQASTAYALRGLFYAEDLYAAAIACGGLSVEAVLGSRSPFDARIHEARGQ
RGQIDTAACFRDLLGDSSEVSLSHKNADKVQDPYSLRCQPQVMGACLTQLRQAAEVLGIE
ANAVSDNPLVFAAEGDVISGGNFHAEPVAMAADNLALAIAEIGSLSERRISLMMDKHMSQ
LPPFLVENGGVNSGFMIAQVTAAALASENKALSHPHSVDSLPTSANQEDHVSMAPAAGKR
LWEMAENTRGVLAIEWLGACQGLDLRKGLKTSAKLEKARQALRSEVAHYDRDRFFAPDI
EKAVELLAKGSLTGLLPAGVLPSL
>non-antioxidant_105
PLLIKNGEIITADSRYKADIYAEGETITRIGQNLEAPPGTEVIDATGKYVFPGFIDPHVHIYLP
FMATFAKDTHETGSKAALMGGTTTYIEMCCPSRNDDALEGYQLWKSKAEGNSYCDYTF
HMAVSKFDEKTEGQLREIVADGISSFKIFLSYKNFFGVDDGEMYQTLRLAKELGVIVTAHC
ENAELVGRLQQKLLSEGKTGPEWHEPSRPEAVEAEGTARFATFLETTGATGYVVHLSCKPA
LDAAMAAKARGVPIYIESVIPHFLLDKTYAERGGVEAMKYIMSPPLRDKRNQKVLWDAL
AQGFIDTVGTDHCPFDTEQKLLGKEAFTAIPNGIPAIEDRVNLLYTYGVSRGRLDIHRFVDA
ASTKAAKLFGLFPRKGTIAVGSDADLVVYDPQYRGTISVKTQHVNNDYNGFEGFEIDGRP
SVVTVRGKVAVRDGQFVGEKGWGKLLRREPMYF
>non-antioxidant_106
MLYLTQRLEIPAAATASVTLPIDVRVKSRVKVTLNDGRDAGLLLPRGLLLRGGDVLSNEEG
TEFVQVIAADEEVSVVRCDDPFMLAKACYALGNRHVPLQIMPGELRYHHDHVLDDMLR
QFGLTVTFGQLPFEPEAGAYASES
>non-antioxidant_107
MDQFECINVADAHQKLQEKEAVLVDIRDPQSFAMGHAVQAFHLTNDTLGAFMRDNDFDT
PVMVMCYHGNSSKGAAQYLLQQGYDVVYSIDGGFEAWQRQFPAEVAYGA
>non-antioxidant_108
MKSNEHDDCQVTNPSTGHLFDLSSLSGRAGFTAAYSEKGLVYMSICGENENCPPGVGACF
GQTRISVGKANKRLRYVDQVLQLVYKDGSPCPSKSGLSYKSVISFVCRPEAGPTNRPMLIS
LDKQTCTLFFSWHTPLACEQAT
>non-antioxidant_109
MHHHHHHGSACFAKGTNVLMADGSIECIENIEVGNKVMGKDGRPREVIKLPRGSETMYS
VVQKSQHRAHKSDSSREMPELLKFTCNATHELVVRTPRSVRRLSRTIKGVEYFEVITFEMG
QKKAPDGRIVELVKEVSKSYPVSEGPERANELVESYRKASNKAYFEWTIEARDLSLLGSH
VRKATYQTYAPIGAAFARECRGFYFELQELKEDDYYGITLSDDSDHQFLLANQVVVH

>non-antioxidant_110
QDDLTISSLAKGETTKAAFNQMVQGHKLPAWVMKGGTYTPAQTVTLGDETYQVMSACK
PHDCGSQRIAVMWSEKSNQMTGLFSTIDEKTSQEKLTWLNVNDALSIDGKTVLFAALTGS
LENHPDGFNFRHHHHHH
>non-antioxidant_111
EDGYDMWLRYQPIADQTLLKTYQKQIRHLHVAGDSPTINAAAAELQRGLSGLLNKPIVAR
DEKLKDYSLVIGTPDNSPLIASLNLGERLQALGAEGYLLEQTRINKRHVVIVAANSDVGVL
YGSFHLLRLIQTQHALEKLSLSSAPRLQHRVVNHWDNLNRVVERGYAGLSLWDWGSLPN
YLAPRYTDYARINASLGINGTVINNVNADPRVLSDQFLQKIAALADAFRPYGIKMYLSINF
NSPRAFGDVDTADPLDPRVQQWWKTRAQKIYSYIPDFGGFLVKADSEGQPGPQGYGRDH
AEGANMLAAALKPFGGVVFWRAFVYHPDIEDRFRGAYDEFMPLDGKFADNVILQIKNGP
IDFQPREPFSALFAGMSRTNMMMEFQITQEYFGFATHLAYQGPLFEESLKTETHARGEGSTI
GNILEGKVFKTRHTGMAGVINPGTDRNWTGHPFVQSSWYAFGRMAWDHQISAATAADE
WLRMTFSNQPAFIEPVKQMMLVSREAGVNYRSPLGLTHLYSQGDHYGPAPWTDDLPRAD
WTAVYYHRASKTGIGFNRTKTGSNALAQYPEPIAKAWGDLNSVPEDLILWFHHLSWDHR
MQSGRNLWQELVHKYYQGVEQVRAMQRTWDQQEAYVDAARFAQVKALLQVQEREAV
RWRNSCVLYFQSVAGRPIPANYEQPEHDLEYYKMLARTTYVPEPWHPASSSRVLK
>non-antioxidant_112
EFMKVLVIGNGGREHALAWKAAQSPLVETVFVAPGNAGTALEPALQNVAIGVTDIPALLDF
AQNEKIDLTIVGPEAPLVKGVVDTFRAAGLKIFGPTAGAAQLEGSKAFTKDFLARHKIPTA
EYQNFTEVEPALAYLREKGAPIVIKADGLAAGKGVIVAMTLEEAEAAVHDMLAGNAFGD
AGHRIVIEEFLDGEEASFIVMVDGEHVLPMATSQDHKRVGDKDTGPNTGGMGAYSPAPV
VTDDVHQRTMERIIWPTVKGMAAEGNTYTGFLYAGLMIDKQGNPKVIEFNCRFGDLETQ
PIMLRMKSDLVELCLAACESKLDEKTSEWDERASLGVVMAAGGYPGDYRTGDVIHGLPL
EEVAGGKVFHAGTKLADDEQVVTNGGRVLCVTALGHTVAEAQKRAYALMTDIHWDDCF
CRKDIGWRAIEREQN
>non-antioxidant_113
DVTNAEKLVYKYTNIAHSANPMYEAPSITDGKIFFNRKFKTPSGKEAACASCHTNNPANV
GKNIVTGKEIPPLAPRVNTKRFTDIDKVEDEFTKHCNDILGADCSPSEKANFIAYLLTETKP
TK
>non-antioxidant_114
MSISARNQLKGKVVGLKKGVVTAEVVLEIAGGNKITSIISLDSVEELGVKEGAELTAVVKS
TDVMILA
>non-antioxidant_115
LIKGPWTKEEDQRLIKLVQKYGPKRWSVIAKHLKGRIGKQCRERWHNHLNPE
>non-antioxidant_116
MNHKPKGTFKDYVRDRADLNKDKPVIPAAALAGYTGSGPIQLWQFLLELLTDKSCQSFIS
WTGDGWEFKLSDPDEVARRWGKRKNKPKMNYEKLSRGLRYYYDKNIIHKTAGKRYVYR
FVCDLQSLLGYTPEELHAMLDVKPDADE
>non-antioxidant_117
MIKVEIKPSQAQFTTRSGVSRQGKPYSLNEQLCYVDLGNEYPVLVKITLDEGQPAYAPGLY
TVHLSSFKVGQFGSLMIDRLRLVPAK
>non-antioxidant_118
MEHQKTTPHATGSTRQNGAPAVSDRQSLTVGSEGPIVLHDTHLLETHQHFNRMNIPERRP

HAKGSGAFGEFEVTEDVSKYTKALVFQPGTKTETLLRFSTVAGELGSPDTWRDVRGFALR
FYTEEGNYDLVGNNTPIFFLRDPMKFTHFIRSQKRLPDSGLRDATMQWDFWTNNPESAHQ
VTYLMGPRGLPRTWREMNGYGSHTYLWVNAQGEKHWVKYHFISQQGVHNLSNDEATKI
AGENADFHRQDLFESIAKGDHPKWDLYIQAIPYEEGKTYRFNPFDLTKTISQKDYPRIKVG
TLTLNRNPENHFAQIESAAFSPSNTVPGIGLSPDRMLLGRAFAYHDAQLYRVGAHVNQLPV
NRPKNAVHNYAFEGQMWYDHTGDRSTYVPNSNGDSWSDETGPVDDGWEADGTLTREA
QALRADDDDFGQAGTLVREVFSDQERDDFVETVAGALKGVRQDVQARAFEYWKNVDA
TIGQRIEDEVKRHEGDGIPGVEAGGEARI
>non-antioxidant_119
MNVRATYTVIFKNASGLPNGYDNWGWGCTLSYYGGAMIINPQEGKYGAVSLKRNSGSFR
GGSLRFDMKNEGKVKILVENSEADEKFEVETISPSDEYVTYILDVDFDLPFDRIDFQDAPG
NGDRIWIKNLVHSTGSADDFVDPINLEHHHHHH
>non-antioxidant_120
GLVPRGSHMTGRMLTLDGNPAANWLNNARTKWSASRADVVLSYQQNNGGWPKNLDYN
SVGNGGGGNESGTIDNGATITEMVFLAEVYKSGGNTKYRDAVRKAANFLVNSQYSTGAL
PQFYPLKGGYSDHATFNDNGMAYALTVLDFAANKRAPFDTDVFSDNDRTRFKTAVTKGT
DYILKAQWKQNGVLTVWCAQHGALDYQPKKARAYELESLSGSESVGVLAFLMTQPQTA
EIEQAVRAGVAWFNSPRTYLEGYTYDSSLAATNPIVPRAGSKMWYRFYDLNTNRGFFSDR
DGSKFYDITQMSLERRTGYSWGGNYGTSIINFAQKVGYL
>non-antioxidant_121
MAKNTSCGVQLRIRGKVQGVGFRPFVWQLAQQLNLHGDVCNDGDGVEVRLREDPEVFL
VQLYQHCPPLARIDSVEREPFIWSALPTEFTIR
>non-antioxidant_122
MSLDFNTLAQNFTQFYYNQFDTDRSQLGNLYRNESMLTFETSQLQGAKDIVEKLVSLPFQ
KVQHRITTLDAQPASPYGDVLVMITGDLLIDEEQNPQRFSQVFHLIPDGNSYYVFNDIFRL
NYSA
>non-antioxidant_123
PHIDIKCFPRELDEQQKAALAADITDVIIRHLNSKDSSISIALQQIQPESWQAIWDAEIAPQM
EALIKKPGYSMNA
>non-antioxidant_124
SELIVNVINGPNLGRLGRREPAVYGGTTHDELVALIEREAAELGLKAVVRQSDSEAQLLDW
IHQAADAAEPVILNAGGLTHTSVALRDACAELSAPLIEVHISNVHAREEFRRHSYLSPIATG
VIVGLGIQGYLLALRYLAEHVGT
>non-antioxidant_125
SELNEKLATAWEGFTKGDWQNEVNVRDFIQKNYTPYEGDESFLAGATEATTTLWDKVME
GVKLENRTHAPVDFDTAVASTITSHDAGYINKQLEKIVGLQTEAPLKRALIPFGGIKMIEGS
CKAYNRELDPMIKKIFTEYRKTHNQGVFDVYTPDILRCRKSGVLTGLPDAYGRGRIIGDYR
RVALYGIDYLMKDKLAQFTSLQADLENGVNLEQTIRLREEIAEQHRALGQMKEMAAKYG
YDISGPATNAQEAIQWTYFGYLAAVKSQNGAAMSFGRTSTFLDVYIERDLKAGKITEQEA
QEMVDHLVMKLRMVRFLRTPEYDELFSGDPIWATESIGGMGLDGRTLVTKNSFRFLNTLY
TMGPSPEPNMTILWSEKLPLNFKKFAAKVSIDTSSLQYENDDLMRPDFNNDDYAIACCVSP
MIVGKQMQFFGARANLAKTMLYAINGGVDEKLKMQVGPKSEPIKGDVLNYDEVMERM
DHFMDWLAKQYITALNIIHYMHDKYSYEASLMALHDRDVIRTMACGIAGLSVAADSLSAI
KYAKVKPIRDEDGLAIDFEIEGEYPQFGNNDPRVDDLAVDLVERFMKKIQKLHTYRDAIPT

QSVLTITSNVVYGKKTGNTPDGRRAGAPFGPGANPMHGRDQKGAVASLTSVAKLPFAYAK
DGISYTFSIVPNALGKDDEVRKTNLAGLMDGYFHHEASIEGGQHLNVNVMNREMLLDA
MENPEKYPQLTIRVSGYAVRFNSLTKEQQQDVITRTFTQSM
>non-antioxidant_126
ADDTIDHASHTPGSVSSAFILEAMVNVISGPKVLMKQIPIWLPLGVADQKTYSFDSTTAAI
MLASYTITHFGKATNPLVRVNRLGPGIPDHPLRLLRIGNQAFLQEFVLPPVQLPQYFTFDLT
ALKLITQPLPAATWTDD
>non-antioxidant_127
MLSFQYPDVYRDETAIQDYHGHKVCDPYAWLEDPDSEQTKAFVEAQNKITVPFLEQCPIR
GLYKERMTELYDYPKYSCHFKKGKRYFYFYNTGLQNQRVLYVQDSLEGEARVFLDPNILS
DDGTVALRGYAFSEDGEYFAYGLSASGSDWVTIKFMKVDGAKELPDVLERVKFSCMAWT
HDGKGMFYNAYPQQDGKSDGTETSTNLHQKLYYHVLGTDQSEDILCAEFPDEPKWMGG
AELSDDGRYVLLSIREGCDPVNRLWYCDLQQESNGITGILKWVKLIDNFEGEYDYVTNEG
TVFTFKTNRHSPNYRLINIDFTDPEESKWKVLVPEHEKDVLEWVACVRSNFLVLCYLHDV
KNTLQLHDLATGALLKIFPLEVGSVVGYSGQKKDTEIFYQFTSFLSPGIIYHCDLTKEELEP
RVFREVTVKGIDASDYQTVQIFYPSKDGTKIPMFIVHKKGIKLDGSHPAFLYGYGGFNISIT
PNYSVSRLIFVRHMGGVLAVANIRGGGEYGETWHKGGILANKQNCFDDFQCAAEYLIKE
GYTSPKRLTINGGSNGGLLVATCANQRPDLFGCVIAQVGVMDMLKFHKYTIGHAWTTDY
GCSDSKQHFEWLIKYSPLHNVKLPEADDIQYPSMLLLTADHDDRVVPLHSLKFIATLQYIV
GRSRKQNNPLLIHVDTKAGHGAGKPTAKVIEEVSDMFAFIARCLNIDWIP
>non-antioxidant_128
GPDDPLVINGEIEIVTRAPTPAHLADRFDEIRSGWTFRTDDTQALEMDDFENSGMVFVEEA
RAVWDRPEGTEGKACADCHGAVDDGMYGLRAVYPKYVESAGKVRTVEQMINACRTSR
MGAPEWDYIGPDMTAMVALIASVSRGMPVSVAIDGPAQSTWEKGREIYYTRYGQLDLSC
ASCHEQYFDHYIRADHLSQGQINGFPSYRLKNARLNAVHDRFRGCIRDTRGVPFAVGSPEF
VALELYVASRGNGLSVEGPSVRN
>non-antioxidant_129
GKITLYEDRGFQGRHYECSSDHPNLQPYLSRCNSARVDSGCWMLYEQPNYSGLQYFLHR
GDYADHQQWMGLSDSVRSCRLIPHSGSHRIRLYEREDYRGQMIEFTEDCSCLQDRFRFNEI
HSLNVLEGSWVLYELSNYRGRQYLLMPGDYRRYQDWGATNARVGSLRRVIDFS
>non-antioxidant_130
EIVTDNSIGNHDGYDYEFWKDSGGSGTMILNHGGTFSAQWNNVNNILFRKGKKFNETQT
HQQVGNMSINYGANFQPNGNAYLCVYGWTVDPLVEYYIVDSWGNWRPPGATPKGTITV
DGGTYDIYETLRVNQPSIKGIATFKQYWSVRRSKRTSGTISVSNHFRAWENLGMNMGKM
YEVALTVEGYQSSGSANVYSNTLRINGNPLS
>non-antioxidant_131
MAFQLEMVTRETVVIRLFGELDHHAVEQIRAKISTAIFQGAVTTIIWNFERLSFMDSSGVGL
VLGRMRELEAVAGRTILLNPSPTMRKVFQFSGLGPWMMDATEEEAIDRVRGIVNG
>non-antioxidant_132
TLTKHEQDILLKELGPHVDTPAHIVETGLGAYHALFTAHPQYISHFSRLEGHTIENVMQSEG
IKHYARTLTEAIVHMLKEISNDAEVKKIAAQYGKDHTSRKVTKDEFMSGEPIFTKYFQNLV
KDAEGKAAVEKFLKHVFPMMAAEI
>non-antioxidant_133
GAMEKFKTLLYDIPIECMEVSEEIISYAKLQLGKKLNDSIYVSLTDHINFAIQRNQKGLDIKN

ALLWETKRLYKDEFAIGKEALVMVKNKTGVSLPEDEAGFIALHIVNAELNEEMPNIINITK
VMEEILSIVKYHFKIEFNEESLHYYRFVTDLKFFAQRLFNGTHMESEDDFLLDTVKEKYHR
AYECTKKIQTYIEREYEHKLTSDELLYLTIDIERVVKQA
>non-antioxidant_134
IVGGYTCAANSIPYQVSLNSGSHFCGGSLINSQWVVSAAHCYKSRIQVRLGEHNIDVLEG
NEQFINAAKIITHPNFNGNTLDNDIMLIKLSSPATLNSRVATVSLPRSCAAAGTECLISGWGN
TKSSGSSYPSLLQCLKAPVLSDSSCKSSYPGQITGNMICVGFLEGGKDSCQGDSGGPVVCN
GQLQGIVSWGYGCAQKNKPGVYTKVCNYVNWIQQTIAAN
>non-antioxidant_135
ADKLFINALKKKFEESPEEKKTTFYTLGGWKQSERKTEFVNAGKEVAAKRGIPQYNPDIG
TPLGQRVLMPYQVSTTDTYVEGDDLHFVNNAAMQQMWDDIRRTVIVGLNHAHAVIEKR
LGKEVTPETITHYLETVNHAMPGAAVVQEHMVETHPALVADSYVKVFTGNDEIADEIDPA
FVIDINKQFPEDQAETLKAEVGDGIWQVVRIPTIVSRTCDGATTSRWSAMQIGMSMISAYK
QAAGEAATGDFAYAAKHAEVIHMGTYLPVRRARGENEPGGVPFGYLADICQSSRVNYED
PVRVSLDVVATGAMLYDQIWLGSYMSGGVGFTQYATAAYTDNILDDFTYFGKEYVEDKY
GLCEAPNNMDTVLDVATEVTFYGLEQYEEYPALLEDQFGGSQRAAVVAAAAGCSTAFATG
NAQTGLSGWYLSMYLHKEQHSRLGFYGYDLQDQCGASNVFSIRGDEGLPLELRGPNYPN
YAMNVGHQGEYAGISQAPHAARGDAFVFNPLVKIAFADDNLVFDFTNVRGEFAKGALRE
FEPAGERALITPAK
>non-antioxidant_136
AKFEDKVDLYDDRGNLVEEQVPLEALSPLRNPAIKSIVQGIKRTVAVNLEGIENALKTAKV
GGPACKIMGRELDLDIVGNAESIAAAAKEMIQVTEDDDTNVELLGGGKRALVQVPSARFD
VAAEYSAAPLVTATAFVQAIINEFDVSMYDANMVKAAVLGRYPQSVEYMGANIATMLDIP
QKLEGPGYALRNIMVNHVVAATLKNTLQAAALSTILEQTAMFEMGDAVGAFERMHLLGL
AYQGMNADNLVFDLVKANGKEGTVGSVIADLVERALEDGVIKVEKELTDYKVYGTDDL
AMWNAYAAAGLMAATMVNQGAARAAQGVSSTLLYYNDLIEFETGLPSVDFGKVEGTAV
GFSFFSHSIYGGGGPGIFNGNHIVTRHSKGFAIPCVAAAMALDAGTQMFSPEATSGLIKEVF
SQVDEFREPLKYVVEAAAEIKNEI
>non-antioxidant_137
AQYYPGTTKVAQNRRNFCNPEYELEKLREISDEDVVKILGHRAPGEEYPSVHPPLEEMDE
PEDAIREMVEPIDGAKAGDRVRYIQFTDSMYFAPAQPYVRSRAYLCRYRGADAGTLSGRQ
IIETRERDLEKISKELLETEFFDPARSGVRGKSVHGHSLRLDEDGMMFDMLRRQIYNKDTG
RVEMVKNQIGDELDEPVDLGEPLDEETLMEKTTIYRVDGEAYRDDVEAVEIMQRIHVLRS
QGGFNLE
>non-antioxidant_138
MAVKKIAIFGATGQTGLTTLAQAVQAGYEVTVLVRDSSRLPSEGPRPAHVVVGDVLQAAD
VDKTVAGQDAVIVLLGTRNDLSPTTVMSEGARNIVAAMKAHGVDKVVACTSAFLLWDPT
KVPPRLQAVTDDHIRMHKVLRESGLKYVAVMPPHIGDQPLTGAYTVTLDGRGPSRVISKH
DLGHFMLRCLTTDEYDGHSTYPSHQYQ
>non-antioxidant_139
MSRTVMERIEYEMHTPDPKADPDKLHFVQIDEAKCIGCDTCSQYCPTAAIFGEMGEPHSIP
HIEACINCGQCLTHCPENAIYEAQSWVPEVEKKLKDGKVKCIAMPAPAVRYALGDAFGMP
VGSVTTGKMLAALQKLGFAHCWDTEFTADVTIWEEGSEFVERLTKKSDMPLPQFTSCCPG
WQKYAETYYPELLPHFSTCKSPIGMNGALAKTYGAERMKYDPKQVYTVSIMPCIAKKYE

GLRPELKSSGMRDIDATLTTRELAYMIKKAGIDFAKLPDGKRDSLMGESTGGATIFGVTGG
VMEAALRFAYEAVTGKKPDSWDFKAVRGLDGIKEATVNVGGTDVKVAVVHGAKRFKQV
CDDVKAGKSPYHFIEYMACPGGCVCGGGQPVMPGVLEAMDRTTTRLYAGLKKRLAMAS
ANKA
>non-antioxidant_140
GFDLNDFLEQLRQMKNMGGMASLMGKLPGMGQIPDNVKSQMDDKVLVRMEAIINSMT
MKERAKPEIIKGSRKRRIAAGSGMQVQDVNRLLKQFDDMQRMMKKMKK
>non-antioxidant_141
MAQGEKITVSNGVLNVPNNPIIPFIEGDGTGPDIWNAASKVLEAAVEKAYKGEKKITWKE
VYAGEKAYNKTGEWLPAETLDVIREYFIAIKGPLTTPVGGGIRSLNVALRQELDLFVCLRP
VRYFTGVPSPVKRPEDTDMVIFRENTEDIYAGIEYAKGSEEVQKLISFLQNELNVNKIRFPE
TSGIGIKPVSEEGTSRLVRAAIDYAIEHGRKSVTLVHKGNIMKFTEGAFKNWGYELAEKEY
GDKVFTWAQYDRIAEEQGKDAANKAQSEAEAAGKIIIKDSIADIFLQQILTRPNEFDVVAT
MNLNGDYISDALAAQVGGIGIAPGANINYETGHAIFEATHGTAPKYAGLDKVNPSSVILSG
VLLLEHLGWNEAADLVIKSMEKTIASKVVTYDFARLMDGATEVKCSEFGEELIKNMD
>non-antioxidant_142
QNNTDYPFEANNPYMYHENPMEEGLSMLKLANLAEAALAFEAVCQKEPEREEAWRSLGL
TQAENEKDGLAIIALNHARMLDPKDIAVHAALAVSHTNEHNANAALASLRAWLLSQPQY
EQL
>non-antioxidant_143
GSMSFIPVAEDSDFPIQNLPYGVFSTQSNPKPRIGVAIGDQILDLSVIKHLFTGPALSKHQHV
FDETTLNNFMGLGQAAWKEARASLQNLLSASQARLRDDKELRQRAFTSQASATMHLPATI
GDYTDFYSSRQHATNVGIMFRGKENALLPNWLHLPVGYHGRASSIVVSGTPIRRPMGQM
RPDNSKPPVYGACRLLDMELEMAFFVGPGNRFGEPIPISKAHEHIFGMVLMNDWSARDIQ
QWEYVPLGPFLGKSFGTTISPWVVPMDALMPFVVPNPKQDPKPLPYLCHSQPYTFDINLS
VSLKGEGMSQAATICRSNFKHMYWTMLQQLTHHSVNGCNLRPGDLLASGTISGSDPESFG
SMLELSWKGTKAIDVGQGQTRTFLLDGDEVIITGHCQGDGYRVGFGQCAGKVLPALSPA
>non-antioxidant_144
GHEIKDIREDTMHAEFNALRAQVAINDGNPDEAERLAKLALEELPPGWFYSRIVATSVLGE
VLHCKGELTRSLALMQQTEQMARQHDVWHYALWSLIQQSEILFAQGFLQTAWETQEKAF
QLINEQHLEQLPMHEFLVRIRAQLLWAWARLDEAEASARSGIEVLSSYQPQQQLQCLAMLI
QCSLARGDLDNARSQLNRLENLLGNGKYHSDWISNANKVRVIYWQMTGDKAAAANWL
RHTAKPEFANNHFLQGQWRNIARAQILLGEFEPAEIVLEELNENARSLRLMSDLNRNLLLL
NQLYWQAGRKSDAQRVLLDALKLANRTGFISHFVIEGEAMAQQLRQLIQLNTLPELEQHR
AQRILREINQHHGA
>non-antioxidant_145
ACDYTCGSNCYSSSDVSTAQAAGYKLHEDGETVGSNSYPHKYNNYEGFDFSVSSPYYEW
PILSSGDVYSGGSPGADRVVFNENNQLAGVITHTGASGNNFVECT
>non-antioxidant_146
EAAQSVDQLIKARGKVYFGVATDQNRLTTGKNAAIIQANFGQVTPENSMKWDATEPSQG
NFNFAGADYLVNWAQQNGKLIRGHTLVWHSQLPSWVSSITDKNTLTNVMKNHITTLMTR
YKGKIRAWDVVNEAFNEDGSLRQTVFLNVIGEDYIPIAFQTARAADPNAKLYINDYNLDS
ASYPKTQAIVNRVKKWRAAGVPIDGIGSQTHLSAGQGASVLQALPLLASAGTPEVAITEL
DVAGASSTDYVNVVNACLNVSSCVGITVWGVADPDSWRASTTPLLFDGNFNPKPAYNAI

VQNLQQ
>non-antioxidant_147
MRGSHHHHHHGSRVMVIGGDGYCGWATALHLSKKNYEVCIVDNLVRRLFDHQLGLESLT
PIASIHDRISRWKALTGKSIELYVGDICDFEFLAESFKSFEPDSVVHFGEQRSAPYSMIDRSR
AVYTQHNNVIGTLNVLFAIKEFGEECHLVKLGTMGEYGTPNIDIEEGYITITHNGRTDTLPY
PKQASSFYHLSKVHDSHNIAFTCKAWGIRATDLNQGVVYGVKTDETEMHEELRNRLDYD
AVFGTALNRFCVQAAVGHPLTVYGKGGQTRGYLDIRDTVQCVEIAIANPAKAGEFRVFNQ
FTEQFSVNELASLVTKAGSKLGLDVKKMTVPNPRVEAEEHYYNAKHTKLMELGLEPHYL
SDSLLDSLLNFAVQFKDRVDTKQIMPSVSWKKIGVKTKSMTT
>non-antioxidant_148
GPLGSGDVQVTEDAVRRYLTRKPMTTKDLLKKFQTKKTGLSSEQTVNVLAQILKRLNPER
KMINDKMHFSLKE
>non-antioxidant_149
HRQALGERLYPRVQAMQPAFASKITGMLLELSPAQLLLLLASEDSLRARVDEAMELIIAHG
>non-antioxidant_150
DKIPDFVVPGKCASVDRNKLWAEQTPNRNSYAGVWYQFALTNNPYQLIEKCVRNEYSFD
GKQFVIESTGIAYDGNLLKRNGKLYPNPFGEPHLSIDYENSFAAPLVILETDYSNYACLYSCI
DYNFGYHSDFSFIFSRSANLADQYVKKCEAAFKNINVDTTRFVKTVQGSSCPYDTQKTL
>non-antioxidant_151
MKLCFNEATTLENSNLKLDLELCEKHGYDYIEIRTMDKLPEYLKDHSLDDLAEYFQTHHI
KPLALNALVFFNNRDEKGHNEIITEFKGMMETCKTLGVKYVVAVPLVTEQKIVKEEIKKSS
VDVLTELSDIAEPYGVKIALEFVGHPQCTVNTFEQAYEIVNTVNRDNVGLVLDSFHFHAM
GSNIESLKQADGKKIFIYHIDDTEDFPIGFLTDEDRVWPGQGAIDLDAHLSALKEIGFSDVV
SVELFRPEYYKLTAEEAIQTAKKTTVDVVSKYFSM
>non-antioxidant_152
DCYHGDGQSYRGSFSTTVTGRTCQSWSSMTPHWHQRTTEYYPNGGLTRNYCRNPDAEIR
PWCYTMDPSVRWEYCNLTQCPVME
>non-antioxidant_153
EDAKAGEAVFKQCMTCHRADKNMVGPALAGVVGRKAGTAAGFTYSPLNHNSGEAGLV
WTADNIVPYLADPNAFLKKFLTEKGKADQAVGVTKMTFKLANEQQRKDVVAYLATLK
>non-antioxidant_154
MGSQVQKSDEITFSDYLGLMTCVYEWADSYDSKDWDRLRKVIAPTLRIDYRSFLDKLWE
AMPAEEFVGMVSSKQVLGDPTLRTQHFIGGTRWEKVSEDEVIGYHQLRVPHQRYKDTTM
KEVTMKGHAHSANLHWYKKIDGVWKFAGLKPDIRWGEFDFDRIAEDGRETFGDK
>non-antioxidant_155
DSLRPKLSEEQQRIIAILLDAHHKTYDPTYSDFCQFRPPVRVNDGGGSVTLELSQLSMLPH
LADLVSYSIQKVIGFAKMIPGFRDLTSEDQIVLLKSSAIEVIMLRSNESFTMDDMSWTCGN
QDYKYRVSDVTKAGHSLELIEPLIKFQVGLKKLNLHEEEHVLLMAICIVSPDRPGVQDAA
LIEAIQDRLSNTLQTYIRCRHPPPGSHLLYAKMIQKLADLRSLNEEHSKQYRCLSFQPECSM
KLTPLVLEVFGNEIS
>non-antioxidant_156
GSHRTSGRVAVEEVDEEGKFVRLRNKSNEDQSMGNWQIKRQNGDDPLLTYRFPPKFTLKA
GQVVTIWAAGAGATHSPPTDLVWKAQNTWGCGNSLRTALINSTGEEVAMRKLVRSVTVV
ED

>non-antioxidant_157
GSASAKELACQEITVPLCKGIGYEYTYMPNQFNHDTQDEAGLEVHQFWPLVEIQCSPDLK
FFLCSMYTPICLEDYKKPLPPCRSVCERAKAGCAPLMRQYGFAWPDRMRCDRLPEQGNP
DTLCMDYERTD
>non-antioxidant_158
MSEFLTPERTVYDSGVQFLRPKSLDEFIGQENVKKKLSLALEAAKMRGEVLDHVLLAGPP
GLGKTTLAHIIASELQTNIHVTSGPVLVKQGDMAAILTSLERGDVLFIDEIHRLNKAVEELL
YSAIEDFQIDIMIGKGPSAKSIRIDIQPFTLVGATTRSGLLSSPLRSRFGIILELDFYTVKELKEI
IKRAASLMDVEIEDAAAEMIAKRSRGTPRIAIRLTKRVRDMLTVVKADRINTDIVLKTMEV
LNIDDEGLDEFDRKILKTIIEIYRGGPVGLNALAASLGVEADTLSEVYEPYLLQAGFLARTP
RGRIVTEKAYKHLKYEVPENRLF
>non-antioxidant_159
GLNSVIKPTKYKPVPDEEPNSTDVEETLKRIQNNDPDLEEVNLNNIMNIPVPTLKACAEAL
KTNTYVKKFSIVGTRSNDPVAFALAEMLKVNNTLKSLNVESNFISGSGILALVEALQSNTS
LIELRIDNQSQPLGNNVEMEIANMLEKNTTLLKFGYHFTQQGPRLRASNAMMNNNDLVR
KRRL
>non-antioxidant_160
MEVARGLEGVLFTESRMCYIDGQQGKLYYYGIPIQELAEKSSFEETTFLLLHGRLPRRQEL
EEFSAALARRRALPAHLLESFKRYPVSAHPMSFLRTAVSEFGMLDPTEGDISREALYEKGL
DLIAKFATIVAANKRLKEGKEPIPPREDLSHAANFLYMANGVEPSPEQARLMDAALILHAE
HGFNASTFTAIAAFSTETDLYSAITAAVASLKGPRHGGANEAVMRMIQEIGTPERAREWVR
EKLAKKERIMGMGHRVYKAFDPRAGVLEKLARLVAEKHGHSKEYQILKIVEEEAGKVLN
PRGIYPNVDFYSGVVYSDLGFSLEFFTPIFAVARISGWVGHILEYQELDNRLLRPGAKYVGE
LDVPYVPLEARE
>non-antioxidant_161
PKYTIVDKETCIACGACGAAAPDIYDYDEDGIAYVTLDDNQGIVEVPDILIDDMMDAFEG
CPTDSIKVADEPFDGDPNKFE
>non-antioxidant_162
MIVGNLGAQKAKRNDTPISAKKDIMGDKTVRVRADLHHIIKIETAKNGGNVKEVMDQAL
EEYIRKYLPDKL
>non-antioxidant_163
LQPATAEAADSYKIVGYYPSWAAYGRNYNVADIDPTKVTHINYAFADICWNGIHGNPDPS
GPNPVTWTCQNEKSQTINVPNGTIVLGDPWIDTGKTFAGDTWDQPIAGNINQLNKLKQTN
PNLKTIISVGGWTWSNRFSDVAATAATREVFANSAVDFLRKYNFDGVDLDWEYPVSGGLD
GNSKRPEDKQNYTLLLSKIREKLDAAGAVDGKKYLLTIASGASATYAANTELAKIAAIVD
WINIMTYDFNGAWQKISAHNAPLNYDPAASAAGVPDANTFNVAAGAQGHLDAGVPAAK
LVLGVPFYGRGWDGCAQAGNGQYQTCTGGSSVGTWEAGSFDFYDLEANYINKNGYTRY
WNDTAKVPYLYNASNKRFISYDDAESVGYKTAYIKSKGLGGAMFWELSGDRNKTLQNKL
KADL
>non-antioxidant_164
ASHSRKFLDVRSEEELLSCIKKETEAGKLPPNVAAGMEELYQNYRNAVIESGNPKADEIVL
SNMTVALDRILLDVEDPFVFSSHHKAIREPFDYYIFGQNYIRPLIDFGNSFVGNLSLFKDIEE
KLQQGHNVVLISNHQTEADPAIISLLLEKTNPYIAENTIFVAGDRVLADPLCKPFSIGRNLIC
VYSKKHMFDIPELTETKRKANTRSLKEMALLLRGGSQLIWIAPSGGRDRPDPSTGEWYPA

PFDASSVDNMRRLIQHSDVPGHLFPLALLCHDIMPPPSQVEIEIGEKRVIAFNGAGLSVAPEI
SFEEIAATHKNPEEVREAYSKALFDSVAMQYNVLKTAISGKQGLGASTADVSLSQPW
>non-antioxidant_165
AAPKAPADGLKMDKTKQPVVFNHSTHKAVKCGDCHHPVNGKEDLQKCATAGCHDNMD
KKDKSAKGYYHAMHDKGTKFKSCVGCHLETAGADAAKKKELTGCKGSKCHS
>non-antioxidant_166
GSMRILMVGLDAAGKTTILYKLKLGEIVTTIPTIGFNVETVEYKNISFTVWDVGGLDKIRPL
WRHYFQNTQGLIFVVDSNDRERVNEAREELMRMLAEDELRDAVLLVFANKQDLPNAMN
AAEITDKLGLHSLRHRNWYIQATCATSGDGLYEGLDWLSNQLRNQK
>non-antioxidant_167
MRHDFVFSGVIMLELNAKTTALVVIDLQEGILPFAGGPHTADEVVNRAGKLAAKFRASGQ
PVFLVRVGWSADYAEALKQPVDAPSPAKVLPENWWQHPAALGTTDSDIEIIKRQWGAFY
GTDLELQLRRRGIDTIVLCGISTNIGVESTARNAWELGFNLVIAEDACSAASAEQHNNSINH
IYPRIARVRSVEEILNAL
>non-antioxidant_168
DCPSGWSSYEGHCYKPFKLYKTWDDAERFCTEQAKGGHLVSIESAGEADFVAQLVTENIQ
NTKSYVWIGLRVQGKEKQCSSEWSDGSSVSYENWIEAESKTCLGLEKETGFRKWVNIYC
GQQNPFVCEA
>non-antioxidant_169
MRIINADGLILGRLASRVAKMLLEGEEVVIVNAEKAVITGNREVIFSKYKQRTGLRTLTNPR
RGPFYPKRSDEIVRRTIRGMLPWKTDRGRKAFRRLKVYVGIPKEFQDKQLETIVEAHVSR
LSRPKYVTVGEVAKFLGGKF
>non-antioxidant_170
MVEPSLVLYGAPYERAVEVLEETLRETGARYALLIDRKGFVLAHKEALWAPKPPPLDTLAT
LVAGNAAATQALAKLLGEARFQEEVHQGERMGLYVDEAGEHALLVLVFDETAPLGKVKL
HGKRASEALARIAEEALANPPRLALDTEYREGAEALLDDLLRN
>non-antioxidant_171
MSETENQALTFAKRLKADTTAVHDSVDNLVMSVQPFVSKENYIKFLKLQSVFHKAVDHIY
KDAELNKAIPELEYMARYDAVTQDLKDLGEEPYKFDKELPYEAGNKAIGWLYCAEGSNL
GAAFLFKHAQKLDYNGEHGARHLAPHPDGRGKHWRAFVEHLNALNLTPEAEAEAIQGA
REAFAFYKVVLRETFGLAADAEAPEGMMPHRH
>non-antioxidant_172
MPSVESFELDHNAVVAPYVRHCGVHKVGTDGVVNKFDIRFCQPNKQAMKPDTIHTLEHL
LAFTIRSHAEKYDHFDIIDISPMGCQTGYYLVVSGETTSAEIVDLLEDTMKEAVEITEIPAAN
EKQCGQAKLHDLEGAKRLMRFWLSQDKEELLKVFG
>non-antioxidant_173
SSNAKADQASSDAQTANAKADQASNDANAARSDAQAAKDDAARANQRADNAA
>non-antioxidant_174
MQSLANATAAQQEVLEASYAMVQHIAKGIRILEARVARVEAM
>non-antioxidant_175
MWQQWEEEIEQHEGNLSLLLREAALQVHIAQRDARM
>non-antioxidant_176
ADVPAGVQLADKQTLVRNNGSEVQSLDPHKIEGVPESNVSRDLFEGLLISDVEGHPSPGVA
EKWENKDFKVWTFHLRENAKWSDGTPVTAHDFVYSWQRLADPNTASPYASYLQYGHIA

NIDDIIAGKKPATDLGVKALDDHTFEVTLSEPVPYFYKLLVHPSVSPVPKSAVEKFGDKWT
QPANIVTNGAYKLKNWVVNERIVLERNPQYWDNAKTVINQVTYLPISSEVTDVNRYRSGE
IDMTYNNMPIELFQKLKKEIPNEVRVDPYLCTYYYEINNQKAPFNDVRVRTALKLALDRDI
IVNKVKNQGDLPAYSYTPPYTDGAKLVEPEWFKWSQQKRNEEAKKLLAEAGFTADKPLT
FDLLYNTSDLHKKLAIAVASIWKKNLGVNVNLENQEWKTFLDTRHQGTFDVARAGWCA
DYNEPTSFLNTMLSDSSNNTAHYKSPAFDKLIADTLKVADDTQRSELYAKAEQQLDKDSAI
VPVYYYVNARLVKPWVGGYTGKDPLDNIYVKNLYIIKH
>non-antioxidant_177
GLSAAQRQVVASTWKDIAGSDNGAGVGKECFTKFLSAHHDMAAVFGFSGASDPGVADL
GAKVLAQIGVAVSHLGDEGKMVAEMKAVGVRHKGYGNKHIKAEYFEPLGASLLSAMEH
RIGGKMNAAAKDAWAAAYADISGALISGLQS
>non-antioxidant_178
MDKKTIYFISTGNSARSQMAEGWGKEILGEGWNVYSAGIETHGVNPKAIEAMKEVDIDIS
NHTSDLIDNDILKQSDLVVTLCSDADNNCPILPPNVKKEHWGFDDPAGKEWSEFQRVRDE
IKLAIEKFKLR
>non-antioxidant_179
TMASGAPSFPFSRASGPEPPAEFAKLRATNPVSQVKLFDGSLAWLVTKHKDVCFVATSEKL
SKVRTRQGFPELSASGKQAAKAKPTFVDMDPPEHMHQRSMVEPTFTPEAVKNLQPYIQRT
VDDLLEQMKQKGCANGPVDLVKEFALPVPSYIIYTLLGVPFNDLEYLTQQNAIRTNGSSTA
REASAANQELLDYLAILVEQRLVEPKDDIISKLCTEQVKPGNIDKSDAVQIAFLLLVAGNAT
MVNMIALGVATLAQHPDQLAQLKANPSLAPQFVEELCRYHTASALAIKRTAKEDVMIGDK
LVRANEGIIASNQSANRDEEVFENPDEFNMNRKWPPQDPLGFGFGDHRCIAEHLAKAELT
TVFSTLYQKFPDLKVAVPLGKINYTPLNRDVGIVDLPVIF
>non-antioxidant_180
SAAMAEQRHQEWLRFVDLLKNAYQNDLHLPLLNLMLTPDEREALGTRVRIIEELLRGEMS
QRELKNELGAGIATITRGSNSLKAAPVELRQWLEEVLLKSD
>non-antioxidant_181
ATPNKTPPGADPKQLERTGTVREIGSQAVWSLSSCKPGFGVDQLRDDNLETYWQSDGSQP
HLVNIQFRRKTTVKTLCIYADYKSDESYTPSKISVRVGNNFHNLQEIRQLELVEPSGWIHVP
LTDNHKKPTRTFMIQIAVLANHQNGRDTHMRQIKIYTPVEESSIGKFPR
>non-antioxidant_182
AANDNNVEWNGLFHDQGPLFDNAPEPTSTQSVTLKLRTFKGDITSANIKYWDTADNAFH
WVPMVWDSNDPTGTFDYWKGTIPASPSIKYYRFQINDGTSTAWYNGNGPSSTEPNADDF
YIIPNFKTPDWLKNGVMYQIFPDRFYNGDSSNDVQTGSYTYNGTPTEKKAWGSSVYADP
GYDNSLVFFGGDLAGIDQKLGYIKKTLGANILYLNPIFKAPTNHKYDTQDYMAVDPAFGD
NSTLQTLINDIHSTANGPKGYLILDGVFNHTGDSHPWFDKYNNFSSQGAYESQSSPWYNY
YTFYTWPDSYASFLGFNSLPKLNYGNSGSAVRGVIYNNSNSVAKTYLNPPYSVDGWRLDA
AQYVDANGNNGSDVTNHQIWSEFRNAVKGVNSNAAIIGEYWGNANPWTAQGNQWDAA
TNFDGFTQPVSEWITGKDYQNNSASISTTQFDSWLRGTRANYPTNVQQSMMNFLSNHDIT
RFATRSGGDLWKTYLALIFQMTYVGTPTIYYGDEYGMQGGADPDNRRSFDWSQATPSNS
AVALTQKLITIRNQYPALRTGSFMTLITDDTNKIYSYGRFDNVNRIAVVLNNDSVSHTVNVP
VWQLSMPNGSTVTDKITGHSYTVQNGMVTVAVDGHYGAVLAQ
>non-antioxidant_183
SIRLPAHLRLQPIYWSRDDVAQWLKWAENEFSLRPIDSNTFEMNGKALLLLTKEDFRYRSP

HSGDELYELLQHILKQRDHHHHHHHRHD
>non-antioxidant_184
GPVWRKHYITYRINNYTPDMNREDVDYAIRKAFQVWSNVTPLKFSKINTGMADILVVFAR
GAHGDFHAFDGKGGILAHAFGPGSGIGGDAHFDEDEFWTTHSGGTNLFLTAVHAIGHSLG
LGHSSDPKAVMFPTYKYVDINTFRLSADDIRGIQSLYG
>non-antioxidant_185
MIALIQRVTRASVTVEGEVTGEIGAGLLVLLGVEKDDDEQKANRLCERVLGYRIFSDAEG
KMNLNVQQAGGSVLVVSQFTLAADTERGMRPSFSKGASPDRAEALYDYFVERCRQQEM
NTQTGRFAADMQVSLVNDGPVTFWLQV
>non-antioxidant_186
MEAAHFFEGTEKLLEVWFSRQQPDANQGSGDLRTIPRSEWDILLKDVQCSIISVTKTDKQE
AYVLSESSMFVSKRRFILKTCGTTLLLKALVPLLKLARDYSGFDSIQSFFYSRKNFMKPSH
QGYPHRNFQEEIEFLNAIFPNGAGYCMGRMNSDCWYLYTLDFPESRVISQPDQTLEILMSE
LDPAVMDQFYMKDGVTAKDVTRESGIRDLIPGSVIDATMFNPCGYSMNGMKSDGTYWTI
AITPEPEFSYVSFETNLSQTSYDDLIRKVVEVFKPGKFVTTLFVNQSSKCRTVLASPQKIEG
FKRLDCQSAMFNDYNFVFTSFAKKQQQQQS
>non-antioxidant_187
MLKQVEIFTAGSALGNPGPGGYGAILRYRGREKTFSAGYTRTTNNRMELMAAIVALEALK
EHAEVILSTDSQYVRQGITQWIHNWKKRGWKTADKKPVKNVDLWQRLDAALGQHQIK
WEWVKGHAGHPENERADELARAAAMNPTLEDTGYQVEV
>non-antioxidant_188
DAPAVGKDLTQAAENIPPAFHNAPRQGELPALNYVNQPPMVPHSVANYQVTKNVNQCLN
CHSPENSRLSGATRISPTHFMDRDGKVGSSSSPRRYFCLQCHVSQANVDPIVPNDFKPMKG
YGN
>non-antioxidant_189
TTLSTKQKQFLKGLAHHLNPVVMLGGNGLTEGVLAEIENALNHHELIKVKVAGADRETK
QLIINAIVRETKAAQVQTIGHILVLYRPSEEAKIQLPRK
>non-antioxidant_190
MKTTLLKTLTPELHLVQHNDIPVLHLKHAVGTAKISLQGAQLISWKPQNAKQDVLWLSEV
EPFKNGNAIRGGVPICYPWFGGVKQPAHGTARIRLWQLSHYYISVHKVRLEFELFSDLNIIE
AKVSMVFTDKCHLTFTHYGEESAQAALHTYFNIGDINQVEVQGLPETCFNSLNQQQENVP
SPRHISENVDCIYSAENMQNQILDKSFNRTIALHHHNASQFVLWNPWHKKTSGMSETGYQ
KMLCLETARIHHLLEFGESLSVEISLKG
>non-antioxidant_191
LMGDDKVKKEVGRASWKYFHTLLARFPDEPTPEEREKLHTFIGLYAELYPCGECSYHFVK
LIEKYPVQTSSRTAAAMWGCHIHNKVNEYLKKDIYDCATILEDYDCGCSDSDGKRVS
>non-antioxidant_192
DTSSLIVEDAPDHVRPYVIRHYSHARAVTVDTQLYRFYVTGPSSGYAFTLMGTNAPHSDA
LGVLPHIHQKHYENFYCNKGSFQLWAQSGNETQQTRVLSSGDYGSVPRNVTHTFQIQDPD
TEMTGVIVPGGFEDLFYYLGTNATDTTHTPYIPSSSDSSSTTGPDSSTISTLQSFDVYAELSF
TPRTDTVNGTAPANTVWHTGANALASTAGDPYFIANGWGPKYLNSQYGYQIVAPFVTAT
QAQDTNYTLSTISMSTTPSTVTVPTWSFPGACAFQVQEGRVVVQIGDYAATELGSGDVAFI
PGGVEFKYYSEAYFSKVLFVSSGSDGLDQNLVNGGEEWSSVSFPADW
>non-antioxidant_193

VLNGYWGYQEFLDEFPEQRNLTNALSEAVRAQPVPLSKPTQRPIKISVVYPGQQVSDYWV
RNIASFEKRLYKLNINYQLNQVFTRPNADIKQQSLSLMEALKSKSDYLIFTLDTTRHRKFV
EHVLDSTNTKLILQNITTPVREWDKHQPFLYVGFDHAEGSRELATEFGKFFPKHTYYSVLY
FSEGYISDVRGDTFIHQVNRDNNFELQSAYYTKATKQSGYDAAKASLAKHPDVDFIYACS
TDVALGAVDALAELGREDIMINGWGGGSAELDAIQKGDLDITVMRMNDDTGIAMAEAIK
WDLEDKPVPTVYSGDFEIVTKADSPERIEALKKRAFRYSDN
>non-antioxidant_194
SACKETGWPFCSDEDWNTKCPSGCRMKGLIDEVDQDFTSRINKLRDSLFNYQK
>non-antioxidant_195
KVERKPPDADGCLHADPDLGVLCPTGCKLQDTLVRQERPIRKSIEDLRNTVDSVSR
>non-antioxidant_196
YVATRDNCCILDERFGSYCPTTCGIADFLNNYQTSVDKDLRTLEGILY
>non-antioxidant_197
MGSSHHHHHHSSGLVPRGSHMKEIRVKAIILAAGLGTRLRPLTENTPKALVQVNQKPLIEY
QIEFLKEKGINDIIIIVGYLKEQFDYLKEKYGVRLVFNDKYADYNNFYSLYLVKEELANSYV
IDADNYLFKNMFRNDLTRSTYFSVYREDCTNEWFLVYGDDYKVQDIIVDSKAGRILSGVS
FWDAPTAEKIVSFIDKAYVSGEFVDLYWDNMVKDNIKELDVYVEELEGNSIYEIDSVQDY
RKLEEILKNEN
>non-antioxidant_198
GSHMLEDPVVLQRRDWENPGVTQLNRLAAHPPFASWRNSEEARTDRPSQQLRSLNGEW
RFAWFPAPEAVPESWLECDLPEADTVVVPSNWQMHGYDAPIYTNVTYPITVNPPFVPTEN
PTGCYSLTFNVDESWLQEGQTRIIFDGVNSAFHLWCNGRWVGYGQDSRLPSEFDLSAFLR
AGENRLAVMVLRWSDGSYLEDQDMWRMSGIFRDVSLLHKPTTQISDFHVATRFNDDFSR
AVLEAEVQMCGELRDYLRVTVSLWQGETQVASGTAPFGGEIIDERGGYADRVTLRLNVEN
PKLWSAEIPNLYRAVVELHTADGTLIEAEACDVGFREVRIENGLLLLNGKPLLIRGVNRHE
HHPLHGQVMDEQTMVQDILLMKQNNFNAVRCSHYPNHPLWYTLCDRYGLYVVDEANIE
THGMVPMNRLTDDPRWLPAMSERVTRMVQRDRNHPSVIIWSLGNESGHGANHDALYRW
IKSVDPSRPVQYEGGGADTTATDIICPMYARVDEDQPFPAVPKWSIKKWLSLPGETRPLILC
QYAHAMGNSLGGFAKYWQAFRQYPRLQGGFVWDWVDQSLIKYDENGNPWSAYGGDFG
DTPNDRQFCMNGLVFADRTPHPALTEAKHQQQFFQFRLSGQTIEVTSEYLFRHSDNELLH
WMVALDGKPLASGEVPLDVAPQGKQLIELPELPQPESAGQLWLTVRVVQPNATAWSEAG
HISAWQQWRLAENLSVTLPAASHAIPHLTTSEMDFCIELGNKRWQFNRQSGFLSQMWIGD
KKQLLTPLRDQFTRAPLDNDIGVSEATRIDPNAWVERWKAAGHYQAEAALLQCTADTLA
DAVLITTAHAWQHQGKTLFISRKTYRIDGSGQMAITVDVEVASDTPHPARIGLNCQLAQVA
ERVNWLGLGPQENYPDRLTAACFDRWDLPLSDMYTPYVFPSENGLRCGTRELNYGPHQW
RGDFQFNISRYSQQQLMETSHRHLLHAEEGTWLNIDGFHMGIGGDDSWSPSVSAEFQLSA
GRYHYQLVWCQK

>non-antioxidant_199
PEGPEIRRAADNLEAAIKGKPLTDVWFAFPQLKTYQSQLIGQHVTHVETRGKALLTHFSND
LTLYSHNQLYGVWRVVDTGEEPQTTRVLRVKLQTADKTILLYSASDIEMLRPEQLTTHPFL
QRVGPDVLDPNLTPEVVKERLLSPRFRNRQFAGLLLDQAFLAGLGNYLRVEILWQVGLTG
NHKAKDLNAAQLDALAHALLEIPRFSYATRGQVDENKHHGALFRFKVFHRDGEPCERCG
SIIEKTTLSSRPFYWCPGCQH

>non-antioxidant_200
MPSTDSIPKSNFDAIPDVIQAFKNGEFVVVLDDPSRENEADLIIAAESVTTEQMAFMVRHS
SGLICAPLTPERTTALDLPQMVTHNADPRGTAYTVSVDAEHPSTTTGISAHDRALACRMLA
APDAQPSHFRRPGHVFPLRAVAGGVRARRGHTEAGVELCRLAGKRPVAVISEIVDDGQEV
EGRAVRAAPGMLRGDECVAFARRWGLKVCTIEDMIAHVEKTEGKLETNGSG
>non-antioxidant_201
GHMIMANWQSIDELQDIASDLPRFIHALDELSRRLGLNITPLTADHISLRCHQNATAERWR
RGFEQCGELLSENMINGRPICLFKLHEPVQVAHWQFSIVELPWPGEKRYPHEGWEHIEIVL
PGDPETLNARALALLSDEGLSLPGISVKTSSPKGEHERLPNPTLAVTDGKTTIKFHPWSIEEI
VASEQSA
>non-antioxidant_202
SITENTSWNKEFSAEAVNGVFVLCKSSSKSCATNDLARASKEYLPASTFKIPNAIIGLETGVI
KNEHQVFKWDGKPRAMKQWERDLTLRGAIQVSAVPVFQQIAREVGEVRMQKYLKKFSY
GNQNISGGIDKFWLEGQLRISAVNQVEFLESLYLNKLSASKENQLIVKEALVTEAAPEYLV
HSKTGFSGVGTESNPGVAWWVGWVEKETEVYFFAFNMDIDNESKLPLRKSIPTKIMESEGI
IGG
>non-antioxidant_203
ATCTVKSVDDAKDIAGCSAVTLNGFTVPAGNTLVLNPDKGATVTMAGDITFAKTTLDGPL
FTIDGTGINFVGADHIFDGNGALYWDGKGTNNGTHKPHPFLKIKGSGTYKKFEVLNSPAQ
AISVGPTDAHLTLDGITVDDFAGDTKNLGHNTDGFDVSANNVTIQNCIVKNQDDCIAIND
GNNIRFENNQCSGGHGISIGSIATGKHVSNVVIKGNTVTRSMYGVRIKAQRTATSASVSGV
TYDANTISGIAKYGVLISQSYPDDVGNPGTGAPFSDVNFTGGATTIKVNNAATRVTVECGN
CSGNWNWSQLTVTGGKAGTIKSDKAKITGGQYL
>non-antioxidant_204
GSHSMRYFHTSVSRPGRGEPRFITVGYVDDTLFVRFDSDAASPREEPRAPWIEQEGPEYW
DRETQICKAKAQTDREDLRTLLRYYNQSEAGSHTLQNMYGCDVGPDGRLLRGYHQHAY
DGKDYIALNEDLSSWTAADTAAQITQRKWEAARVAEQLRAYLEGECVEWLRRYLENGKE
TLQRADPPKTHVTHHPISDHEATLRCWALGFYPAEITLTWQRDGEDQTQDTELVETRPAG
DRTFQKWAAVVVPSGEEQRYTCHVQHEGLPKPLTLRWEP
>non-antioxidant_205
TTVYLAGDSTMAKNGGGSGTNGWGEYLASYLSATVVNDAVAGRSARSYTREGRFENIAD
VVTAGDYVIVEFGHNDGGSLSTDNGRTDCSGTGAEVCYSVYDGVNETILTFPAYLENAAK
LFTAKGAKVILSSQTPNNPWETGTFVNSPTRFVEYAELAAEVAGVEYVDHWSYVDSIYET
LGNATVNSYFPIDHTHTSPAGAEVVAEAFLKAVVCTGTSLKSVLTTTSFEGTCL
>non-antioxidant_206
MSQFFYIHPDNPQQRLINQAVEIVRKGGVIVYPTDSGYALGCKIEDKNAMERICRIRQLPD
GHNFTLMCRDLSELSTYSFVDNVAFRLMKNNTPGNYTFILKGTKEVPRRLLQEKRKTIGM
RVPSNPIAQALLEALGEPMLSTSLMLPGSEFTESDPEEIKDRLEKQVDLIIHGGYLGQKPTT
VIDLTDDTPVVVREGVGDVKPFL
>non-antioxidant_207
TTILKHLPVGQRIGIAFSGGLDTSAALLWMRQKGAVPYAYTANLGQPDEEDYDAIPRRAM
EYGAENARLIDCRKQLVAEGIAAIQCGAFHNTTGGLTYFNTTPLGRAVTGTMLVAAMKED
GVNIWGDGSTYKGNDIERFYRYGLLTNAELQIYKPWLDTDFIDELGGRHEMSEFMIACGF
DYKMSVEKAYSTDSNMLGATHEAKDLEYLNSSVKIVNPIMGVKFWDESVKIPAEEVTVRF

EQGHPVALNGKTFSDDVEMMLEANRIGGRHGLGMSDQIENRIIEAKSRGIYEAPGMALLH
IAYERLLTGIHNEDTIEQYHAHGRQLGRLLYQGRWFDSQALMLRDSLQRWVASQITGEVT
LELRRGNDYSILNTVSENLTYKPERLTMEKGDSVFSPDDRIGQLTMRNLDITDTREKLFGY
AKTGLLSSSAASGVPQVENLENKGQSVEHHHHHH
>non-antioxidant_208
MEITSDMEEDKDLMLKLLDKNGFVLKKVEIYRSNYLAILEKRTNGIRNFEINNNGNMRIF
GYKMMEHHIQKFTDIGMSCKIAKNGNVYLDIKRSAENIEAVITVASEL
>non-antioxidant_209
AAGTAKGHNPTEFPTIYDASSAPTAANTTVGIITIGGVSQTLQDLQQFTSANGLASVNTQTI
QTGSSNGDYSDDQQGQGEWDLDSQSIVGSAGGAVQQLLFYMADQSASGNTGLTQAFNQ
AVSDNVAKVINVSLGWCEADANADGTLQAEDRIFATAAAQGQTFSVSSGDEGVYECNNR
GYPDGSTYSVSWPASSPNVIAVGGTTLYTTSAGAYSNETVWNEGLDSNGKLWATGGGYS
VYESKPSWQSVVSGTPGRRLLPDISFDAAQGTGALIYNYGQLQQIGGTSLASPIFVGLWAR
LQSANSNSLGFPAASFYSAISSTPSLVHDVKSGNNGYGGYGYNAGTGWDYPTGWGSLDIA
KLSAYIRSNGF
>non-antioxidant_210
GSHMRKTLVLLGAHGVGRRHIKNTLITKHPDRFAYPIPHTTRPPKKDEENGKNYYFVSHD
QMMQDISNNEYLEYGSHEDAMYGTKLETIRKIHEQGLIAILDVEPQALKVLRTAEFAPFVV
FIAAPTITPGLNEDESLQRLQKESDILQRTYAHYFDLTIINNEIDETIRHLEEAVELVC
>non-antioxidant_211
TLLGTALRPAATRVMLLGSGELGKEVAIECQRLGVEVIAVDRYADAPAMHVAHRSHVINM
LDGDALRRVVELEKPHYIVPEIEAIATDMLIQLEEEGLNVVPCARATKLTMNREGIRRLAA
EELQLPTSTYRFADSESLFREAVADIGYPCIVKPVMSSSGKGQTFIRSAEQLAQAWKYAQQ
GGRAGAGRVIVEGVVKFDFEITLLTVSAVDGVHFCAPVGHRQEDGDYRESWQPQQMSPL
ALERAQEIARKVVLALGGYGLFGVELFVCGDEVIFSEVSPRPHDTGMVTLISQDLSEFALH
VRAFLGLPVGGIRQYGPAASAVILPQLTSQNVTFDNVQNAVGADLQIRLFGKPEIDGSRRL
GVALATAESVVDAIERAKHAAGQVKVQG
>non-antioxidant_212
MKIKQALFTAGYSSFYFDDQQAIKNGAGHDGFIYTGDPVTPGFTSVRQAGECVSVQLILE
NGAVAVGDCAAVQYSGAGGRDPLFLAEHFIPFLNDHIKPLLEGRDVDAFLPNARFFDKLRI
DGNLLHTAVRYGLSQALLDATALASGRLKTEVVCDEWQLPCVPEAIPLFGQSGDDRYIAV
DKMILKGVDVLPHALINNVEEKLGFKGEKLREYVRWLSDRILSLRSSPRYHPTLHIDVYGT
IGLIFDMDPVRCAEYIASLEKEAQGLPLYIEGPVDAGNKPDQIRMLTAITKELTRLGSGVKI
VADEWCNTYQDIVDFTDAGSCHMVQIKTPDLGGIHNIVDAVLYCNKHGMEAYQGGTCNE
TEISARTCVHVALAARPMRMLIKPGMGFDEGLNIVFNEMNRTIALLQTKD
>non-antioxidant_213
GAMVPNVVVTGLTLVCSSAPGPLELDLTGDLESFKKQSFVLKEGVEYRIKISFRVNREIVSG
MKYIQHTYRKGVKIDKTDYMVGSYGPRAAAYEFLTPVEEAPKGMLARGSYSIKSRFTDD
DKTDHLSWEWNLTIKKDWKD
>non-antioxidant_214
VGSLNCIVAVSQNMGIGKNGDLPWPPLRNEFRYFQRMTTTSSVEGKQNLVIMGKKTWFSI
PEKNRPLKGRINLVLSRELKEPPQGAHFLSRSLDDALKLTEQPELANKVDMVWIVGGSSV
YKEAMNHPGHLKLFVTRIMQDFESDTFFPEIDLEKYKLLPEYPGVLSDVQEEKGIKYKFE
VYEKND

>non-antioxidant_215
DPSRIPSALIGRPAPQTALPPLEGLQADNVQVPGLDPAAFKGKVSLVNVWASWCVPCHDE
APLLTELGKDKRFQLVGINYKDAADNARRFLGRYGNPFGRVGVDANGRASIEWGVYGVP
ETFVVGREGTIVYKLVGPITPDNLRSVLLPQMEKALK
>non-antioxidant_216
QPVLHLVALNTPLSGGMRGIRGADFQCFQQARAVGLSGTFRAFLSSRLQDLYSIVRRADR
GSVPIVNLKDEVLSPSWDSLFSGSQGQLQPGARIFSFDGRDVLRHPAWPQKSVWHGSDPS
GRRLMESYCETWRTETTGATGQASSLLSGRLLEQKAASCHNSYIVLCIENSF
>non-antioxidant_217
MADEIAKAQVARPGGDTIFGKIIRKEIPAKIIFEDDRCLAFHDISPQAPTHFLVIPKKHISQISV
AEDDDESLLGHLMIVGKKCAADLGLNKGYRMVVNEGSDGGQSVYHVHLHVLGGRQMH
WPPG
>non-antioxidant_218
MIANENIQDKALENFKANQTEVTVFFLNGFQMKGVIEEYDKYVVSLNSQGKQHLIYKHAI
STYTVETEGQASTESEE
>non-antioxidant_219
MGDTFIRHIALLGFEKRFVPSQHYVYMFLVKWQDLSEKVVYRRFTEIYEFHKTLKEMFPIE
AGAINPENRIIPHLPAPKWFDGQRAAENRQGTLTEYCSTLMSLPTKISRCPHLLDFFKVRPD
DLKLPTDNQTKKPETYLM
>non-antioxidant_220
MDVSRRQFFKICAGGMAGTTVAALGFAPKQALAQARNYKLLRAKEIRNTCTYCSVGCGL
LMYSLGDGAKNAREAIYHIEGDPDHPVSRGALCPKGAGLLDYVNSENRLRYPEYRAPGS
DKWQRISWEEAFSRIAKLMKADRDANFIEKNEQGVTVNRWLSTGMLCASGASNETGML
TQKFARSLGMLAVDNQARVCHGPTVASLAPTFGRGAMTNHWVDIKNANVVMVMGGNA
AEAHPVGFRWAMEAKNNNDATLIVVDPRFTRTASVADIYAPIRSGTDITFLSGVLRYLIENN
KINAEYVKHYTNASLLVRDDFAFEDGLFSGYDAEKRQYDKSSWNYQLDENGYAKRDETL
THPRCVWNLLKEHVSRYTPDVVENICGTPKADFLKVCEVLASTSAPDRTTTFLYALGWTQ
HTVGAQNIRTMAMIQLLLGNMGMAGGGVNALRGHSNIQGLTDLGLLSTSLPGYLTLPSE
KQVDLQSYLEANTPKATLADQVNYWSNYPKFFVSLMKSFYGDAAQKENNWGYDWLPK
WDQTYDVIKYFNMMDEGKVTGYFCQGFNPVASFPDKNKVVSCLSKLKYMVVIDPLVTE
TSTFWQNHGESNDVDPASIQTEVFRLPSTCFAEEDGSIANSGRWLQWHWKGQDAPGEAR
NDGEILAGIYHHLRELYQSEGGKGVEPLMKMSWNYKQPHEPQSDEVAKENNGYALEDLY
DANGVLIAKKGQLLSSFAHLRDDGTTASSCWIYTGSWTEQGNQMANRDNSDPSGLGNTL
GWAWAWPLNRRVLYNRASADINGKPWDPKRMLIQWNGSKWTGNDIPDFGNAAPGTPTG
PFIMQPEGMGRLFAINKMAEGPFPEHYEPIETPLGTNPLHPNVVSNPVVRLYEQDALRMG
KKEQFPYVGTTYRLTEHFHTWTKHALLNAIAQPEQFVEISETLAAAKGINNGDRVTVSSK
RGFIRAVAVVTRRLKPLNVNGQQVETVGIPIHWGFEGVARKGYIANTLTPNVGDANSQTPE
YKAFLVNIEKA
>non-antioxidant_221
SMQEKIMRELHVKPSIDPKQEIEDRVNFLKQYVKKTGAKGFVLGISGGQDSTLAGRLAQL
AVESIREEGGDAQFIAVRLPHGTQQDEDDAQLALKFIKPDKSWKFDIKSTVSAFSDQYQQE
TGDQLTDFNKGNVKARTRMIAQYAIGGQEGLLVLGTDHAAEAVTGFFTKYGDGGADLLP
LTGLTKRQGRTLLKELGAPERLYLKEPTADLLDEKPQQSDETELGISYDEIDDYLEGKEVS
AKVSEALEKRYSMTEHKRQVPASMFDDWWK

>non-antioxidant_222
GSPEFPGRENLYFQGREPVLDGPYQPTTFNPPVDYWMLLAPTAAGVVVEGTNNTDRWLA
TILVEPNVTSETRSYTLFGTQEQITIANASQTQWKFIDVVKTTQNGSYSQYGPLQSTPKLYA
VMKHNGKIYTYNGETPNVTTKYYSTTNYDSVNMTAFCDFYIIPREEESTCTEYINNGL
>non-antioxidant_223
MAYDYKQVLRDSLLFYEAQRSGRLPADQKVTWRKDSALNDQGDQGQDLTGGYFDAGD
FVKFGFPMAYTATVLAWGLIDFEAGYSSAGALDDGRKAVKWATDYFIKAHTSQNEFYGQ
VGQGDADHAFWGRPEDMTMARPAYKIDTSRPGSDLAGETAAALAAASIVFRNVDGTYSN
NLLTHARQLFDFANNYRGKYSDSITDARNFYASADYRDELVWAAAWLYRATNDNTYLNT
AESLYDEFGLQNWGGGLNWDSKVSGVQVLLAKLTNKQAYKDTVQSYVNYLINNQQKTP
KGLLYIDMWGTLRHAANAAFIMLEAAELGLSASSYRQFAQTQIDYALGDGGRSFVCGFGS
NPPTRPHHRSSSCPPAPATCDWNTFNSPDPNYHVLSGALVGGPDQNDNYVDDRSDYVHN
EVATDYNAGFQSALAALVALGY
>non-antioxidant_224
ERDCRVSSFRVKENFDKARFAGTWYAMAKKDPEGLFLQDNIVAEFSVDENGHMSATAKG
RVRLLNNWDVCADMVGTFTDTEDPAKFKMKYWGVASFLQKGNDDHWIIDTDYETFAVQ
YSCRLLNLDGTCADSYSFVFARDPSGFSPEVQKIVRQRQEELCLARQYRLIPHNGYCDGKS
ERNIL
>non-antioxidant_225
AGVPFNTKYPYGPTSIADNQSEVTAMLKAEWEDWKSKRITSNGAGGYKRVQRDASTNY
DTVSQGMGYGLLLAVCFNEQALFDDLYRYVKSHFNGNGLMHWHIDANNNVTSHDGGD
GAATDADEDIALALIFADKLWGSSGAINYGQEARTLINNLYNHCVEHGSYVLKPGDRWGG
SSVTNPSYFAPAWYKVYAQYTGDTRWNQVADKCYQIVEEVKKYNNGTGLVPDWCTASG
TPASGQSYDYKYDATRYGWRTAVDYSWFGDQRAKANCDMLTKFFARDGAKGIVDGYTI
QGSKISNNHNASFIGPVAAASMTGYDLNFAKELYRETVAVKDSEYYGYYGNSLRLLTLLYI
TGNFPNPLSDL
>non-antioxidant_226
GSPGIRLGSSEDNFARFVCKNNGVLFENQLLQIGLKSEFRQNLGRMFIFYGNKTSTQFLNF
TPTLICADDLQTNLNLQTKPVDPTVDGGAQVQQVVNIECISDFTEAPVLNIQFRYGGTFQN
VSVKLPITLNKFFQPTEMASQDFFQRWKQLSNPQQEVQNIFKAKHPMDTEITKAKIIGFGS
ALLEEVDPNPANFVGAGIIHTKTTQIGCLLRLEPNLQAQMYRLTLRTSKDTVSQRLCELLS
EQF
>non-antioxidant_227
MSKSESPKEPEQLRKLFIGGLSFETTDESLRSHFEQWGTLTDCVVMRDPNTKRSRGFGFVT
YATVEEVDAAMNARPHKVDGRVVEPKRAVSREDSQRPGAHLTVKKIFVGGIKEDTEEHH
LRDYFEQYGKIEVIEIMTDRGSGKKRGFAFVTFDDHDSVDKIVIQKYHTVNGHNCEVRKA
LSKQEMASASSSQRGRVGAGIIHTKTTQIGCLLRLEPNLQAQMYRLTLRTSKDTVSQRLCE
LLSEQF
>non-antioxidant_228
MQTLHALLRDIPAPDAEAMARTQQHIDGLLKPPGSLGRLETLAVQLAGMPGLNGTPQVGE
KAVLVMCADHGVWDEGVAVSPKIVTAIQAANMTRGTTGVCVLAAQAGAKVHVIDVGID
AEPIPGVVNMRVARGCGNIAVGPAMSRLQAEALLLEVSRYTCDLAQRGVTLFGVGELGM
ANTTPAAAMVSVFTGSDAKEVVGIGANLPPSRIDNKVDVVRRAIAINQPNPRDGIDVLSK
VGGFDLVGMTGVMLGAARCGLPVLLDGFLSYSAALAACQIAPAVRPYLIPSHFSAEKGAR

IALAHLSMEPYLHMAMRLGEGSGAALAMPIVEAACAMFHNMGELAASNIVLPEGNANA
T
>non-antioxidant_229
AWKGEVLANNEAGQVTSIIYNPGDVITIVAAGWASYGPTQKWGPQGDREHPDQGLICHD
AFCGALVMKIGNSGTIPVNTGLFRWVAPNNVQGAITLIYNDVPGTYGNNSGSFSVNIGKD
QS
>non-antioxidant_230
GRDYRTCLTIVQKLKKMVDKPTQRSVSNAATRVCRTGRSRWRDVCRNFMRRYQSRVIQG
LVAGETAQQICEDLR
>non-antioxidant_231
TKGLVLGIYSKEKEEDEPQFTSAGENFNKLVSGKLREILNISGPPLKAGKTRTFYGLHEDFP
SVVVVGLGKKTAGIDEQENWHEGKENIRAAVAAGCRQIQDLEIPSVEVDPCGDAQAAAE
GAVLGLYEYDDLKQKRKVVVSAKLHGSEDQEAWQRGVLFASGQNLARRLMETPANEMT
PTKFAEIVEENLKSASIKTDVFIRPKSWIEEQEMGSFLSVAKGSEEPPVFLEIHYKGSPNASE
PPLVFVGKGITFDSGGISIKAAANMDLMRADMGGAATICSAIVSAAKLDLPINIVGLAPLCE
NMPSGKANKPGDVVRARNGKTIQVDNTDAEGRLILADALCYAHTFNPKVIINAATLTGA
MDIALGSGATGVFTNSSWLWNKLFEASIETGDRVWRMPLFEHYTRQVIDCQLADVNNIG
KYRSAGACTAAAFLKEFVTHPKWAHLDIAGVMTNKDEVPYLRKGMAGRPTRTLIEFLFR
FSQ
>non-antioxidant_232
MITNSGKFGVVVVGVGRAGSVRLRDLKDPRSAAFLNLIGFVSRRELGSLDEVRQISLEDA
LRSQEIDVAYICSESSSHEDYIRQFLQAGKHVLVEYPMTLSFAAAQELWELAAQKGRVLHE
EHVELLMEEFEFLRREVLGKELLKGSLRFTASPLEEERFGFPAFSGISRLTWLVSLFGELSLI
SATLEERKEDQYMKMTVQLETQNKGLLSWIEEKGPGLKRNRYVNFQFTSGSLEEVPSVG
VNKNIFLKDQDIFVQKLLDQVSAEDLAAEKKRIMHCLGLASDIQKLCHQKKNIVGLAPLC
ENMPSGKANKPGDVVRARNGKTIQVDNTDAEGRLILADALCYAHTFNPKVIINAATLTGA
MDIALGSGATGVFTNSSWLWNKLFEASIETGDRVWRMPLFEHYTRQVIDCQLADVNNIG
KYRSAGACTAAAFLKEFVTHPKWAHLDIAGVMTNKDEVPYLRKGMAGRPTRTLIEFLFR
FSQ
>non-antioxidant_233
LEPEPWFFKNLSRKDAERQLLAPGNTHGSFLIRESESTAGSFSLSVRDFDQNQGEVVKHYK
IRNLDNGGFYISPRITFPGLHELVRHYTNASDGLCTRLSRPCQT
>non-antioxidant_234
APTAKLANGDTITGLNAIINEAFLGIPFAEPPVGNLRFKDPVPYSGSLNGQKFTSYGPSCMQ
QNPEGTFEENLGKTALDLVMQSKVFQAVLPQSEDCLTINVVRPPGTKAGANLPVMLWIFG
GGFEIGSPTIFPPAQMVTKSVLMGKPIIHVAVNYRVASWGFLAGDDIKAEGSGNAGLKDQR
LGMQWVADNIAGFGGDPSKVTIFGESAGSMSVLCHLIWNDGDNTYKGKPLFRAGIMQSG
AMVPSDPVDGTYGNEIYDLFVSSAGCGSASDKLACLRSASSDTLLDATNNTPGFLAYSSL
RLSYLPRPDGKNITDDMYKLVRDGKYASVPVIIGDQNDEGTIFGLSSLNVTTNAQARAYFK
QSFIHASDAEIDTLMAAYPQDITQGSPFDTGIFNAITPQFKRISAVLGDLAFIHARRYFLNHF
QGGTKYSFLSKQLSGLPIMGTFHANDIVWQDYLLGSGSVIYNNAFIAFATDLDPNTAGLLV
NWPKYTSSSQSGNNLMMINALGLYTGKDNFRTAGYDALMTNPSSFFV
>non-antioxidant_235
SAYPITGKLGSELTMTDTVGQVVLGWKVSDLKSSTAVIPGYPVAGQVWEATATVNAIRGS

VTPAVSQFNARTADGINYRVLWQAAGPDTISGATIPQGEQSTGKIYFDVTGPSPTIVAMNNG
MEDLLIWEP
>non-antioxidant_236
DVSGTVCLSALPPEATDTLNLIASDGPFPYSQDGVVFQNRESVLPTQSYGYYHEYTVITPG
ARTRGTRRIITGEATQEDYYTGDHYATFSLIDQTC
>non-antioxidant_237
MRPYYIAIVGSGPSAFFAAASLLKAADTTEDLDMAVDMLEMLPTPWGLVRSGVAPDHPKI
KSISKQFEKTAEDPRFRFFGNVVVGEHVQPGELSERYDAVIYAVGAQSDRMLNIPGEDLPG
SIAAVDFVGWYNAHPHFEQVSPDLSGARAVVIGNGNVALDVARILLTDPDVLARTDIADH
ALESLRPRGIQEVVIVGRRGPLQAAFTTLELRELADLDGVDVVIDPAELDGITDEDAAAVG
KVCKQNIKVLRGYADREPRPGHRRMVFRFLTSPIEIKGKRKVERIVLGRNELVSDGSGRVA
AKDTGEREELPAQLVVRSVGYRGVPTPGLPFDDQSGTIPNVGGRINGSPNEYVVGWIKRG
PTGVIGTNKKDAQDTVDTLIKNLGNAKEGAECKSFPEDHADQVADWLAARQPKLVTSAH
WQVIDAFERAAGEPHGRPRVKLASLAELLRIGLG
>non-antioxidant_238
SQDASDGLQRLHMLQISYFRDPYHVWYQGNASLGGHLTHVLEGPDTNTTIIQLQPLQEPE
SWARTQSGLQSYLLQFHGLVRLVHQERTLAFPLTIRCFLGCELPPEGSRAHVFFEVAVNGSS
FVSFRPERALWQADTQVTSGVVTFTLQQLNAYNRTRYELREFLEDTCVQYVQKHISAENT
KGSQTSRSYTS
>non-antioxidant_239
MFQQLSARLQEAIGRLRGRGRITEEDLKATLREIRRALMDADVNLEVARDFVERVREEAL
GKQVLESLTPAEVILATVYEALKEALGGEARLPVLKDRNLWFLVGLQGSGKTTTAAKLAL
YYKGKGRRPLLVAADTQRPAAREQLRLLGEKVGVPVLEVMDGESPESIRRRVEEKARLEA
RDLILVDTAGRLQIDEPLMGELARLKEVLGPDEVLLVLDAMTGQEALSVARAFDEKVGVT
GLVLTKLDGDARGGAALSARHVTGKPIYFAGVSEKPEGLEPFYPERLAGRILGMG
>non-antioxidant_240
MNDRADFVVPDITTRKNVGLSHDANDFTLPQPLDRYSAEDHATWATLYQRQCKLLPGRA
CDEFLEGLERLEVDADRVPDFNKLNEKLMAATGWKIVAVPGLIPDDVFFEHLANRRFPVT
WWLREPHQLDYLQEPDVFHDLFGHVPLLINPVFADYLEAYGKGGVKAKALGALPMLAR
LYWYTVEFGLINTPAGMRIYGAGILSSKSESIYCLDSASPNRVGFDLMRIMNTRYRIDTFQK
TYFVIDSFKQLFDATAPDFAPLYLQLADAQPWGAGDIAPDDLVLNAGDHQGWADTEDV
>non-antioxidant_241
RVCPRILLECKKDSDCLAECVCLEHGYCG
>non-antioxidant_242
MKFGNFLLTYQPPELSQTEVMKRLVNLGKASEGCGFDTVWLLEHHFTEFGLLGNPYVAA
AHLLGATETLNVGTAAIVLPTAHPVRQAEDVNLLDQMSKGRFRFGICRGLYDKDFRVFGT
DMDNSRALMDCWYDLMKEGFNEGYIAADNEHIKFPKIQLNPSAYTQGGAPVYVVAESAS
TTEWAAERGLPMILSWIINTHEKKAQLDLYNEVATEHGYDVTKIDHCLSYITSVDHDSNRA
KDICRNFLGHWYDSYVNATKIFDDSDQTKGYDFNKGQWRDFVLKGHKDTNRRIDYSYEI
NPVGTPEECIAIIQQDIDATGIDNICCGFEANGSEEEIIASMKLFQSDVMPYLKEKQ
>non-antioxidant_243
SHHWGYGKHNGPEHWHKDFPIAKGERQSPVDIDTHTAKYDPSLKPLSVSYDQATSLRILN
NGHAFNVEFDDSQDKAVLKGGPLDGTYRLIQFHFHWGSLDGQGSEHTVDKKKYAAELHL
VHWNTKYGDFGKAVQQPDGLAVLGIFLKVGSAKPGLQKVVDVLDSIKTKGKSADFTNFD

PRGLLPESLDYWTYPGSLTTPPLLECVTWIVLKEPISVSSEQVLKFRKLNFNGEGEPEELM
VDNWRPAQPLKNRQIKASFK
>non-antioxidant_244
APADKPQVLASFTQTSASSQNAWLAANRNQSAWAAYEFDWSTDLCTQAPDNPFGFPFNT
ACARHDFGYRNYKAAGSFDANKSRIDSAFYEDMKRVCTGYTGEKNTACNSTAWTYYQA
VKIFG
>non-antioxidant_245
AHPELKSSVPQADSAVAAPEKIQLNFSENLTVKFSGAKLTMTGMKGMSSHSPMPVAAKVA
PGADPKSMVIIPREPLPAGTYRVDWRAVSSDTHPITGNYTFTVK
>non-antioxidant_246
MLELLPTAVEGVSQAQITGRPEWIWLALGTALMGLGTLYFLVKGMGVSDPDAKKFYAITT
LVPAIAFTMYLSMLLGYGLTMVPFGGEQNPIYWARYADWLFTTPLLLLDLALLVDADQGT
ILALVGADGIMIGTGLVGALTKVYSYRFVWWAISTAAMLYILYVLFFGFTSKAESMRPEVA
STFKVLRNVTVVLWSAYPVVWLIGSEGAGIVPLNIETLLFMVLDVSAKVGFGLILLRSRAI
FGEAEAPEPSAGDGAAATSD
>non-antioxidant_247
MVDQATLDKLEAGFKKLQEASDCKSLLKKHLTKDVFDSIKNKKTGMGATLLDVIQSGVE
NLDSGVGIYAPDAESYRTFGPLFDPIIDDYHGGFKLTDKHPPKQWGDINTLVGLDPAGQFII
STRVRCGRSLQGYPFNPCLTAEQYKEMEEKVSSTLSSMEDELKGTYYPLTGMSKATQQQL
IDDHFLFKEGDRFLQTANACRYWPTGRGIFHNDAKTFLVWVNEEDHLRIISMQKGGDLKT
VYKRLVTAVDNIESKLPFSHDDRFGFLTFCPTNLGTTMRASVHIQLPKLAKDRKVLEDIAS
KFNLQVRGTRGEHTESEGGVYDISNKRRLGLTEYQAVREMQDGILEMIKMEKAAA
>non-antioxidant_248
MERGEIWLVSLDPTAGHEQQGTRPVLIVTPAAFNRVTRLPVVVPVTSGGNFARTAGFAVSL
DGVGIRTTGVVRCDQPRTIDMKARGGKRLERVPETIMNEVLGRLSTILT
>non-antioxidant_249
TGMSREEVESLIQEVLEVYPEKARKDRNKHLAVNDPAVTQSKKCIISNKKSQPGLMTIRGC
AYAGSKGVVWGPIKDMIHISHGPVGCGQYSRAGRRNYYIGTTGVNAFVTMNFTSDFQEK
DIVFGGDKKLAKLIDEVETLFPLNKGISVQSECPIGLIGDDIESVSKVKGAELSKTIVPVRCE
GFRGVSQSLGHHIANDAVRDWVLGKRDEDTTFASTPYDVAIIGDYNIGGDAWSSRILLEE
MGLRCVAQWSGDGSISEIELTPKVKLNLVHCYRSMNYISRHMEEKYGIPWMEYNFFGPTK
TIESLRAIAAKFDESIQKKCEEVIAKYKPEWEAVVAKYRPRLEGKRVMLYIGGLRPRHVIG
AYEDLGMEVVGTGYEFAHNDDYDRTMKEMGDSTLLYDDVTGYEFEEFVKRIKPDLIGSG
IKEKFIFQKMGIPFREMHSWDYSGPYHGFDGFAIFARDMDMTLNNPCWKKLQAPWEASE
GAEKVAASA
>non-antioxidant_250
SQQVDKIKASYPLFLDQDYKDMLAKKRDGFEEKYPQDKIDEVFQWTTTKEYQELNFQRE
ALTVNPAKACQPLGAVLCALGFEKTMPYVHGSQGCVAYFRSYFNRHFREPVSCVSDSMTE
DAAVFGGQQNMKDGLQNCKATYKPDMIAVSTTCMAEVIGDDLNAFINNSKKEGFIPDEFP
VPFAHTPSFVGSHVTGWDNMFEGIARYFTLKSMDDKVVGSNKKINIVPGFETYLGNFRVI
KRMLSEMGVGYSLLSDPEEVLDTPADGQFRMYAGGTTQEEMKDAPNALNTVLLQPWHL
EKTKKFVEGTWKHEVPKLNIPMGLDWTDEFLMKVSEISGQPIPASLTKERGRLVDMMTDS
HTWLHGKRFALWGDPDFVMGLVKFLLELGCEPVHILCHNGNKRWKKAVDAILAASPYG
KNATVYIGKDLWHLRSLVFTDKPDFMIGNSYGKFIQRDTLHKGKEFEVPLIRIGFPIFDRHH

LHRSTTLGYEGAMQILTTLVNSILERLDEETRGMQATDYNHDLVR
>non-antioxidant_251
ADQKLSDFHAESGGCESCHKDGTPSADGAFEFAQCQSCHGKLSEMDAVHKPHDGNLVCA
DCHAVHDMNVGQKPTCESCHDDGRTSASVLKK
>non-antioxidant_252
SRNVPFPYAETDVADLQARMTAGELDSTTLTQAYLQRIAALDRTGPRLRAVIELNPDALKE
AAERDRERRDGRLRGPLHGIPLLLKDNINAAPMATSAGSLALQGFRPDDAYLVRRLRDAG
AVVLGKTNLSEWANFRGNDSISGWSARGGQTRNPYRISHSPCGSSSGSAVAVAANLASVAI
GTETDGSIVCPAAINGVVGLKPTVGLVSRDGIIPISFSQDTAGPMARSVADAAAVLTAIAGR
DDADPATATMPGRAVYDYTARLDPQGLRGKRIGLLQTPLLKYRGMPPLIEQAATELRRAG
AVVVPVELPNQGAWAEAERTLLLYEFKAGLERYFNTHRAPLRSLADLIAFNQAHSKQELG
LFGQELLVEADATAGLADPAYIRARSDARRLAGPEGIDAALAAHQLDALVAPTTGVAWPIR
SEGDDFPGESYSAAAVAGYPSLTVPMGQIDGLPVGLLFMGTAWSEPKLIEMAYAYEQRTR
ARRPPHFDTDALIDAGEP
>non-antioxidant_253
AEFKHVFVCVQDRPPGHPQGSCAQRGSREVFQAFMEKIQTDPQLFMTTVITPTGCMNAS
MMGPVVVVYPDGVWYGQVKPEDVDEIVEKHLKGGEPVERLVISKGKPPGMF
>non-antioxidant_254
HPETLVKVKDAEDQLGARVGYIELDLNSGKILESFRPEERFPMMSTFKVLLCGAVLSRVDA
GQEQLGRRIHYSQNDLVEYSPVTEKHLTDGMTVRELCSAAITMSDNTAANLLLTTIGGPKE
LTAFLHNMGDHVTRLDRWEPELNEAIPNDERDTTTPAAMATTLRKLLTGELLTLASRQQLI
DWMEADKVAGPLLRSALPAGWFIADKSGAGERGSRGIIAALGPDGKPSRIVVIYTTGSQAT
MDERNRQIAEIGASLIKHW
>non-antioxidant_255
MATFYEVIVRVPFDVEEHLPGISDSFVDWVTGQIWELPPESDLNLTLVEQPQLTVADRIRRV
FLYEWNKFSKQESKFFVQFEKGSEYFHLHTLVETSGISSMVLGRYVSQIRAQLVKVVFQGI
EPQINDWVAITKVKKGGANKVVDSGYIPAYLLPKVQPELQWAWTNLDEYKLAALNLEER
KRLVAQFLAESSQRS
>non-antioxidant_256
MYPVDLHMHTVASTHAYSTLSDYIAQAKQKGIKLFAITDHGPDMEDAPHHWHFINMRIW
PRVVDGVGILRGIEANIKNVDGEIDCSGKMFDSLDLIIAGFHEPVFAPHDKATNTQAMIATI
ASGNVHIISHPGNPKYEIDVKAVAEAAAKHQVALEINNSSFLHSRKGSEDNCREVAAAVRD
AGGWVALGSDSHTAFTMGEFEECLKILDAVDFPPERILNVSPRRLLNFLESRGMAPIAEFA
DL
>non-antioxidant_257
ALCKFCDVRFSTCDNQKSCMSNCSITSICEKPQEVCVAVWRKNDENITLETVCHDPKLPY
HDFILEDAASPTCIMKEKKKPGETFFMCSCSSDECNDNIIFSEEYNTSNPD
>non-antioxidant_258
SLFELGKMIWQETGKNPVKNYGLYGCNCGVGGRGEPLDATDRCCFVHKCCYKKLTDCD
SKKDRYSYKWKNKAIVCGKNQPCMQEMCECDKAFAICLRENLDTYNKSFRYHLKPSCK
KTSEQC
>non-antioxidant_259
CPQEDSDIAFLIDGSGSIIPHDFRRMKEFVSTVMEQLKKSKTLFSLMQYSEEFRIHFTFKEF
QNNPNPRSLVKPITQLLGRTHTATGIRKVVRELFNITNGARKNAFKILVVITDGEKFGDPLG

YEDVIPEADREGVIRYVIGVGDAFRSEKSRQELNTIASKPPRDHVFQVNNFEALKTIQNQL
REKIFCIGS
>non-antioxidant_260
GTLSSEKKAKKVRFYRNGDRYFKGIVYAISPDRFRSFEALLADLTRTLSDNVNLPQGVRTI
YTIDGLKKISSMDQLVEGESYVCGSIEPFKKLEYTKNVNPNWSVNV
>non-antioxidant_261
MQVEANSERRVKILGIDRSENSPVLTYMETEDDPNFRNSKLAAAPHTVHMMDSGFLAINR
QCLVKGKAILAREPKSSNEHMIDDLPKHAHDQHTLSILRDFIDQLKLHNVYEINFYDPLDS
SGKLAVIPMLIALWKCMLASETDICDQEVLKSIMNSVIAKFELQIPCKNAVIDATLSGSREE
VHIIAEDGSLENSNGTTEHFNKKHDLVFVKTDLHPEDFTPQMFPSQAKAKLLRDAFNNEE
DEDTFPDILVPAYMTAHSKNRVRQEDYTCLEVEFDSQVALEKLMNEHEQVEGFEVQQGGI
LVALKKDSFFDDELIEKIAIAIATESRQSVSSVSFDLLKLGPGASLVTLANSRRFEPECRVVL
QIEVKPVSPGETSSEGISDEHHYEEYDEDDIMEEEEAPSARQDDTYDEDEE
>non-antioxidant_262
QESTHIYTKEEVSSHTSPETGIWVTLGSEVFDVTEFVDLHPGGPSKLMLAAGGPLEPFWAL
YAVHNQSHVRELLAQYKIGEL
>non-antioxidant_263
MSLGAKPFGEKKFIEIKGRRMAYIDEGTGDPILFQHGNPTSSYLWRNIMPHCAGLGRLIAC
DLIGMGDSDKLDPSGPERYAYAEHRDYLDALWEALDLGDRVVLVVHDWGSALGFDWAR
RHRERVQGIAYMEAIAMPIEWADFPEQDRDLFQAFRSQAGEELVLQDNVFVEQVLPGLIL
RPLSEAEMAAYREPFLAAGEARRPTLSWPRQIPIAGTPADVVAIARDYAGWLSESPIPKLFI
NAEPGALTTGRMRDFCRTWPNQTEITVAGAHFIQEDSPDEIGAAIAAFVRRLRPAHHHHH
H
>non-antioxidant_264
MKSGIYQIKNTLNNKVYVGSAKDFEKRWKRHFKDLEKGCHSSIKLQRSFNKHGNVFECSI
LEEIPYEKDLIIERANFWIKELNSKINGYNIADATFG
>non-antioxidant_265
MVVKFMDVYQRSYCHPIETLVDIFQEYPDEIEYIFKPSCVPLMRCGGCANDEGLECVPTEE
SNITMQIMRIKPHQGQHIGEMSFLQHNKCEARPKK
>non-antioxidant_266
MTQFAFVFPGQGSQTVGMLADMAASYPIVEETFAEASAALGYDLWALTQQGPAEELNKT
WQTQPALLTASVALYRVWQQQGGKAPAMMAGHSLGEYSALVCAGVIDFADAVRLVEMR
GKFMQEAVPEGTGAMAAIIGLDDASIAKACEEAAEGQVVSPVNFNSPGQVVIAGHKEAVE
RAGAACKAAGAKRALPLPVSVPSHCALMKPAADKLAVELAKITFNAPTVPVVNNVDVKC
ETNGDAIRDALVRQLYNPVQWTKSVEYMAAQGVEHLYEVGPGKVLTGLTKRIVDTLTAS
ALNEPSAMAAALEL
>non-antioxidant_267
AMGQTVTTPLSLTLGHWKDVERIAHNQSVDVKKRRWVTFCSAEWPTFNVGWPRDGTFN
RDLITQVKIKVFSPGPHGHPDQVPYIVTWEALAFDPPPWVKP
>non-antioxidant_268
MNEMENTVPVLQDDLVSKYERELSTEQEEDTPVILTQLNEDGTTSNYFDKRKLKIAPRSTL
QFKVGPPFELVRDYCPVVESHTGRTLDLRIIPRIDRGFDHIDEEWVGYKRNYFTLVSTFETA
NCDLDTFLKSSFDLLVEDSSVEGRLRVQYFAIKIKAKNDDDDTEINLVQHTAKRDKGPQFC
PSVCPLVPSPLPKHQTIREASNVRNITKMKKYDSTFYLHRDHVNYEEYGVDSLLFSYPEDS

IQKVARYERVQFASSISVKKPSQQNKHFSLHVILGAVVDPDTFHGENPGIPYDELALKNGS
KGMFVYLQEMKTPPLIIRGRSPSNYASSQRITVR
>non-antioxidant_269
SQKVEKTVIKNETGTISISQLNKNVWVHTELGSFNGEAVPSNGLVLNTSKGLVLVDSSWD
DKLTKELIEMVEKKFQKRVTDVIITHAHADRIGGIKTLKERGIKAHSTALTAELAKKNGYE
EPLGDLQTVTNLKFGNMKVETFYPGKGHTEDNIVVWLPQYNILVGGCLVKSTSAKDLGN
VADAYVNEWSTSIENVLKRYRNINAVVPGHGEVGDKGLLLHTLDLLK
>non-antioxidant_270
GIVEQCCTSICSLYQLENYCN
>non-antioxidant_271
FVNQHLCGSHLVEALYLVCGERGFFYTPKT
>non-antioxidant_272
MSGGFLRDDHLEFALHLHRRLAEAVPDGEVIWSPYSVACALGVLAAGARATTRTELTTLL
GTDPAPLLAALDRAVTDSPDLASRTVLWVSADVPVRSSFRATMHDRPDSDVRTADFRTNP
EGVRATVNADIADATRGMIRELLPQGAVTPDLRAILTNALWAKARWTTPFEAHLTREGTFR
TPRGPKRVPFMHRTKTMPYATARGWRMVTLHAHDELAVDVLLPPGTNAAAVPTAPLLTAL
HRRSASTSVELALPRFELTQPHQLVEVLAEAGVRTLFTASADLSGISTVPLYVDTVIHQARL
RVDERGAEGAAATAAMMLLA
>non-antioxidant_273
MQASQFSAQVLDWYDKYGRKTLPWQIDKTPYKVWLSEVMLQQTQVATVIPYFERFMAR
FPTVTDLANAPLDEVLHLWTGLGYYARARNLHKAAQQVATLHGGKFPETFEEVAALPGV
GRSTAGAILSLSLGKHFPILNGNVKRVLARCYAVSGWPGKKEVENKLWSLSEQVTPAVGV
ERFNQAMMDLGAMICTRSKPKCSLCPLQNGCIAAANNSWALYPGKKPK
>non-antioxidant_274
SYQPTPEDRFTFGLWTVGWQGRDPFGDATRPALDPVETVQRLAELGAHGVTFHDDDLIPF
GSSDTERESHIKRFRQALDATGMTVPMATTNLFTHPVFKDGGFTANDRDVRRYALRKTIR
NIDLAVELGAKTYVAWGGREGAESGAAKDVRVALDRMKEAFDLLGEYVTSQGYDIRFAI
EPKPNEPRGDILLPTVGHALAFIERLERPELYGVNPEVGHEQMAGLNFPHGIAQALWAGKL
FHIDLNGQSGIKYDQDLRFGAGDLRAAFWLVDLLESAGYEGPRHFDFKPPRTEDIDGVWA
SAAGCMRNYLILKERAAAFRADPEVQEALRASRLDELAQPTAADGVQELLADRTAFEDF
DVDAAAARGMAFERLDQLAMDHLLGAR
>non-antioxidant_275
GSHMYYVIFAQDIPNTLEKRLAVREQHLARLKQLQAENRLLTAGPNPAIDDENPSEAGFTG
STVIAQFENLQAAKDWAAQDPYVEAGVYADVIVKPFKKVF
>non-antioxidant_276
AYTTFSQTKNDQLKEPMFFGQPVNVARYDQQKYDIFEKLIEKQLSFFWRPEEVDVSRDRI
DYQALPEHEKHIFISNLKYQTLLDSIQGRSPNVALLPLISIPELETWVETWAFSETIHSRSYT
HIIRNIVNDPSVVFDDIVTNEQIQKRAEGISSYYDELIEMTSYWHLLGEGTHTVNGKTVTV
SLRELKKKLYLCLMSVNALEAIRFYVSFACSFAFAERELMEGNAKIIRLIARDEALHLTGTQ
HMLNLLRSGADDPEMAEIAEECKQECYDLFVQAAQQEKDWADYLFRDGSMIGLNKDILC
QYVEYITNIRMQAVGLDLPFQTRSNPIPWINTWLVSDNVQVAPQEVEVSSYLVGQIDSEVD
TDDLSNFQL
>non-antioxidant_277
TAELKICRVNRRSGSCLGGDEIFLLCDKVQKEDIEVYFTGPGWEARGSFSQADVHRQVAIV

FRTPPYADPSLQAPVRVSMQLRRPSDRELSEPMEFQYLPDTDDRHRIEEKRKR
>non-antioxidant_278
MTVNLEEKRPEIVGPEKVQSPYPIRFEGKVVHGFGRGSKELGIPTANISEDAIQELLRYRDS
GVYFGYAMVQKRVFPMVMSVGWNPYYKNKLRSAEVHLIERQGEDFYEEIMRVIVLGYIR
PELNYAGLDKLIEDIHTDIRVALNSMDRPSYSSYKKDPFFKV
>non-antioxidant_279
NGRTPLHLAARNGHLEVVKLLLEAGADVNAKDKNGRTPLHLAARNGHLEVVKLLLEAG
ADVNAKDKNGRTPLHLAARNGHLEVVKLLLEAGAY
>non-antioxidant_280
MNAEINPLHAYFKLPNTVSLVAGSSEGETPLNAFDGALLNAGIGNVNLIRIS
>non-antioxidant_281
MIMPPEAEIVPLPKLPMGALVPTAYGYIISDVPGETISAAISVAIPKDKSLCGLIMEYEGKCS
KKEAEKTVREMAKIGFEMRGWELDRIESIAVEHTVEKLGCAFAAAALWYK
>non-antioxidant_282
MGDAPSPEEKLHLITRNLQEVLGEEKLKEILKERELKIYWGTATTGKPHVAYFVPMSKIAD
FLKAGCEVTILFADLHAYLDNMKAPWELLELRVSYYENVIKAMLESIGVPLEKLKFIKGTD
YQLSKEYTLDVYRLSSVVTQHDSKKAGAEVVKQVEHPLLSGLLYPGLQALDEEYLKVDA
QFGGIDQRKIFTFAEKYLPALGYSKRVHLMNPMVPGLTGSKMSSSEEESKIDLLDRKEDVK
KKLKKAFCEPGNVENNGVLSFIKHVLFPLKSEFVILRDEKWGGNKTYTAYVDLEKDFAAE
VVHPGDLKNSVEVALNKLLDPIREKFNTPALKKLASAAYPDPSKQKPMAKGPAKNSEPEE
VILEHHHHHH
>non-antioxidant_283
DNGGYVPAVVIGTGYGAAVSALRLGEAGVQTLMLEMGQLWNQPGPDGNIFCGMLNPDK
RSSWFKNRTEAPLGSFLWLDVVNRNIDPYAGVLDRVNYDQMSVYVGRGVGGGSLVNGG
MAVEPKRSYFEEILPRVDSSEMYDRYFPRANSMLRVNHIDTKWFEDTEWYKFARVSREQA
GKAGLGTVFVPNVYDFGYMQREAAGEVPKSALATEVIYGNNHGKQSLDKTYLAAALGT
GKVTIQTLHQVKTIRQTKDGGYALTVEQKDTDGKLLATKEISCRYLFLGAGSLGSTELLVR
ARDTGTLPNLNSEVGAGWGPNGNIMTARANHMWNPTGAHQSSIPALGIDAWDNSDSSVF
AEIAPMPAGLETWVSLYLAITKNPQRGTFVYDAATDRAKLNWTRDQNAPAVNAAKALFD
RINKANGTIYRYDLFGTQLKAFADDFCYHPLGGCVLGKATDDYGRVAGYKNLYVTDGSLI
PGSVGVNPFVTITALAERNVERIIKQDVTAS
>non-antioxidant_284
MAKAHIELTINGHPVEALVEPRTLLIHFIREQQNLTGAHIGCDTSHCGACTVDLDGMSVKS
CTMFAVQANGASITTIEGMAAPDGTLSALQEGFRMMHGLQCGYCTPGMIMRSHRLLQEN
PSPTEAEIRFGIGGNLCRCTGYQNIVKAIQYAAAKINGVPFEEAAE
>non-antioxidant_285
MNIQTTVEPTSAERAEKLQGMGCKRKRVEDIRFTQGKGNYVDDVKLPGMLFGDFVRSSH
AHARIKSIDTSKAKALPGVFAVLTAADLKPLNLHYMPTLAGDVQAVLADEKVLFQNQEVA
FVVAKDRYVAADAIELVEVDYEPLPVLVDPFKAMEPDAPLLREDIKDKMTGAHGARKHH
NHIFRWEIGDKEGTDATFAKAEVVSKDMFTYHRVHPSPLETCQCVASMDKIKGELTLWGT
FQAPHVIRTVVSLISGLPEHKIHVIAPDIGGGFGNKVGAYSGYVCAVVASIVLGVPVKWVE
DRMENLSTTSFARDYHMTTELAATKDGKILAMRCHVLADHGAFDACADPSKWPAGFMN
ICTGSYDMPVAHLAVDGVYTNKASGGVAYRCSFRVTEAVYAIERAIETLAQRLEMDSADL
RIKNFIQPEQFPYMAPLGWEYDSGNYPLAMKKAMDTVGYHQLRAEQKAKQEAFKRGET

REIMGIGISFFTEIVGAGPSKNCDILGVSMFDSAEIRIHPTGSVIARMGTKSQGQGHETTYA
QIIATELGIPADDIMIEEGNTDTAPYGLGTYGSRSTPTAGAATAVAARKIKAKAQMIAAHML
EVHEGDLEWDVDRFRVKGLPEKFKTMKELAWASYNSPPPNLEPGLEAVNYYDPPNMTYP
FGAYFCIMDIDVDTGVAKTRRFYALDDCGTRINPMIIEGQVHGGLTEAFAVAMGQEIRYDE
QGNVLGASFMDFFLPTAVETPKWETDYTVTPSPHHPIGAKGVGESPHVGGVPCFSNAVND
AYAFLNAGHIQMPHDAWRLWKVGEQLGLHV
>non-antioxidant_286
MIPGSFDYHRPKSIADAVALLTKLGEDARPLAGGHSLIPIMKTRLATPEHLVDLRDIGDLVG
IREEGTDVVIGAMTTQHALIGSDFLAAKLPIIRETSLLIADPQIRYMGTIGGNAANGDPGND
MPALMQCLGAAYELTGPEGARIVAARDYYQGAYFTAIEPGELLTAIRIPVPPTGHGYAYEKL
KRKIGDYATAAAAVVLTMSGGKCVTASIGLTNVANTPLWAEEAGKVLVGTALDKPALDKA
VALAEAITAPASDGRGPAEYRTKMAGVMLRRAVERAKARAKN
>non-antioxidant_287
GSNRRLQQTQAQVDEVVDIMRVNVDKVLERDQKLSELDDRADALQAGASQFETSAAKL
KRKYW
>non-antioxidant_288
GSALSEIETRHSEIIKLENSIRELHDMFMDMAMLVESQGEMIDRIEYNVEHAVDYVERAVS
DTKKAVK
>non-antioxidant_289
GSMRNELEEMQRRADQLADESLESTRRMLQLVEESKDAGIRTLVMLDEQGEQLDRVEEG
MNHINQDMKEAEKNLKDLGK
>non-antioxidant_290
GSARENEMDENLEQVSGIIGNLRHMALDMGNEIDTQNRQIDRIMEKADSNKTRIDEANQR
ATKMLG
>non-antioxidant_291
EDKYTDKYDNINLDEILANKRLLVAYVNCVMERGKCSPEGKELKEHLQDAIENGCKKCT
ENQEKGAYRVIEHLIKNEIEIWRELTAKYDPTGNWRKKYEDRAKAAGIVIPEE
>non-antioxidant_292
MGSMDQSIAVKSPLTYAEALANTIMNTYTVEELPPANRWHYHQGVFLCGVLRLWEATGE
KRYFEYAKAYADLLIDDNGNLLFRRDELDAIQAGLILFPLYEQTKDERYVKAAKRLRSLYG
TLNRTSEGGFWHKDGYPYQMWLDGLYMGGPFALKYANLKQETELFDQVVLQESLMRKH
TKDAKTGLFYHAWDEAKKMPWANEETGCSPEFWARSIGWYVMSLADMIEELPKKHPNR
HVWKNTLQDMIKSICRYQDKETGLWYQIVDKGDRSDNWLESSGSCLYMYAIAKGINKGY
LDRAYETTLLKAYQGLIQHKTETSEDGAFLVKDICVGTSAGFYDYYVSRERSTNDLHGAG
AFILAMTELEPLFRSAGK
>non-antioxidant_293
MSLLERLNQDMKLYMKNREKDKLTVVRMVKASLQNEAIKLKKDSLTEDEELTVLSRELK
QRKDSLQEFSNANRLDLVDKVQKELDILEVYLPEQLSEEELRTIVNETIAEVGASSKADMG
KVMGAIMPKVKGKADGSLINKLVSSQLS
>non-antioxidant_294
MTKQEKTALNMARFIRSQTLTLLEKLNELADAADEQADICESLHDHADELYRSCLARFGD
DGENL
>non-antioxidant_295
AFGITTSSSAYVIDTNAPNQLKFTVSRSSCDITSIIHYGTELQYSSQGSHIGSGLGSATVTATQ

SGDYIKVTCVTDTLTQYMVVHNGDPIIHMATYITAEPSIGELRFIARLNSDLLPNEEPFGDV
STTADGTAIEGSDVFLVGSETRSKFYSSERFIDDQRHCIAGDAHRVCMILNQYESSSGGPFH
RDINSNNGGSYNALYWYMNSGHVQTESYRMGLHGPYSMYFSRSGTPSTSIDTSFFADLDI
KGYVAASGRGKVAGTASGADSSMDWVVHWYNDAAQYWTYTSSSGSFTSPAMKPGTYT
MVYYQGEYAVATSSVTVSAGSTTTKNISGSVKTGTTIFKIGEWDGQPTGFRNAANQLRMH
PSDSRMSSWGPLTYTVGSSALTDFPMAVFKSVNNPVTIKFTATSAQTGAATLRIGTTLSFAG
GRPQATINSYTGSAPAAPTNLDSRGVTRGAYRGLGEVYDVSIPSGTIVAGTNTITINVISGSS
GDTYLSPNFIFDCVELFQ
>non-antioxidant_296
MLTGLNHLTLAVADLPASIAFYRDLLGFRLEARWDQGAYLELGSLWLCLSREPQYGGPAA
DYTHYAFGIAAADFARFAAQLRAHGVREWKQNRSEGDSFYFLDPDGHRLEAHVGDLRSR
LAACRQAPYAGMRFAD
>non-antioxidant_297
MGSSEQELKAIVKDLGCGPYFLGTYDKRFPGFVSPHKLACAIVNTAGRETGGVHWMAFA
WNPRSKTCYLFEPFGFSDQRLKQVYQFEYESLLRRSAIASSPDRCITLEKSTQSVQGPNSA
ACGLFCCMFLHAFANWPQTPMDHNPTMNLITGVPNSMLNSPQVQPTLRRNQEQLYSFLE
RHSPYFRSHSAQIRSATSFCHLKNM
>non-antioxidant_298
MAEESFYGVTLTAESDSVTWDVDEDYARGQKLVIKQILLGAEAKENEFNVVEVNTPKDS
VQIPIAVLKAGETRAVNPDVEFYESKVTFKLIKGSGPVYIHGHNIKDDV
>non-antioxidant_299
ADTIVAVELDTYPNTDIGDPSYPHIGIDIKSVRSKKTAKWNMQNGKVGTAHIIYNSVDKRL
SAVVSYPNADSATVSYDVDLDNVLPEWVRVGLSASTGLYKETNTILSWSFTSKLKSNSTH
ETNALHFMFNQFSKDQKDLILQGDATTGTDGNLELTRVSSNGSPQGSSVGRALFYAPVHI
WESSAVVASFEATFTFLIKSPDSHPADGIAFFISNIDSSIPSGSTGRLLGLFPDAN
>non-antioxidant_300
GSHMAARRGALIVLEGVDRAGKSTQSRKLVEALCAAGHRAELLRFPERSTEIGKLLSSYL
QKKSDVEDHSVHLLFSANRWEQVPLIKEKLSQGVTLVVDRYAFSGVAFTGAKENFSLDW
CKQPDVGLPKPDLVLFLQLQLADAAKRGAFGHERYENGAFQERALRCFHQLMKDTTLN
WKMVDASKSIEAVHEDIRVLSEDAIATATEKPLGELWK
>non-antioxidant_301
DITVYNGQQKEAATAVAKAFEQETGIKVTLNSGKSEQLAGQLKEEGDKTPADVFYTEQTA
TFADLSEAGLLAPISEQTIQQTAQKGVPLAPKKDWIALSGRSRVVVYDHTKLSEKDMEKS
VLDYATPKWKGKIGYVSTSGAFLEQVVALSKMKGDKVALNWLKGLKENGKLYAKNSVA
LQAVENGEVPAALINNYYWYNLAKEKGVENLKSRLYFVRHQDPGALVSYSGAAVLKASK
NQAEAQKFVDFLASKKGQEALVAARAEYPLRADVVSPFNLEPYEKLEAPVVSATTAQDK
EHAIKLIEEAGLK
>non-antioxidant_302
EQGGFSGPSATQSQAGGFQGPNGSVTTVESAKSLRDDTWVTLRGNIVERISDDLYVFKDA
SGTINVDIDHKRWNGVTVTPKDTVEIQGEVDKDWNSVEIDVKQIRKVNP
>non-antioxidant_303
DTVKIDANVNYQIIQGFGGMSGVGWINDLTTEQINTAYGSGVGQIGLSIMRVRIDPDSSKW
NIQLPSARQAVSLGAKIMATPWSPPAYMKSNNSLINGGRLLPANYSAYTSHLLDFSKYMQT
NGAPLYAISIQNEPDWKPDYESCEWSGDEFKSYLKSQGSKFGSLKVIVAESLGFNPALTDP

VLKDSDASKYVSIIGGHLYGTTPKPYPLAQNAGKQLWMTEHYVDSKQSANNWTSAIEVG
TELNASMVSNYSAYVWWYIRRSYGLLTEDGKVSKRGYVMSQYARFVRPGALRIQATENP
QSNVHLTAYKNTDGKMVIVAVNTNDSDQMLSLNISNANVTKFEKYSTSASLNVEYGGSSQ
VDSSGKATVWLNPLSVTTFVSK
>non-antioxidant_304
GGPGNEKLKEKENNDSSDKATVIPNFNTTMQGSLLGDDSRDYYSFEVKEEGEVNIELDKK
DEFGVTWTLHPESNINDRITYGQVDGNKVSNKVKLRPGKYYLLVYKYSGSGNYELRVNK
>non-antioxidant_305
MTFRKSFDCYDFYDRAKVGEKCTQDDWDLMKIPMKAMELKQKYGLDFKGEFIPTDKD
MMEKLFKAGFEMLLECGIYCTDTHRIVKYTEDEIWDAINNVQKEFVLGTGRDAVNVRKR
SVGDKAKPIVQGGPTGSPISEDVFMPVHMSYALEKEVDTIVNGVMTSVRGKSPIPKSPYEV
LAAKTETRLIKNACAMAGRPGMGVKGPETSLSAQGNISADCTGGMTCTDSHEVSQLNEL
KIDLDAISVIAHYKGNSDIIMDEQMPIFGGYAGGIEETTIVDVATHINAVLMSSASWHLDGP
VHIRWGSTNTRETLMIAGWACATISEFTDILSGNQYYPCAGPCTEMCLLEASAQSITDTAS
GREILSGVASAKGVVTDKTTGMEARMMGEVARATAGVEISEVNVILDKLVSLYEKNYASA
PAGKTFQECYDVKTVTPTEEYMQVYDGARKKLEDLGLVF
>non-antioxidant_306
GQDRSEATLIKRFKGEGVRYKAKLIGIDEVSAARGDKLCQDSMMKLKGVVAGARSKGEH
KQKIFLTISFGGIKIFDEKTGALQHHHAVHEISYIAKDITDHRAFGYVCGKEGNHRFVAIKT
AQAAEPVILDLRDLFQLIYELKQREELEKKA
>non-antioxidant_307
MSGTLMAFDFGTKSIGVAVGQRITGTARPLPAIKAQDGTPDWNIIERLLKEWQPDEIIVGLP
LNMDGTEQPLTARARKFANRIHGRFGVEVKLHDERLSTVEARSGLFEQGGYRALNKGKV
DSASAVIILESYMEQGY
>non-antioxidant_308
TDQAAFDTNIVTLTRFVMEEGRKARGTGEMTQLLNSLCTAVKAISTAVRKAGIAHLYGIAG
STNVTGDQVKKLDVLSNDLVINVLKSSFATCVLVSEEDKNAIIVEPEKRGKYVVCFDPLDG
SSNIDCLVSIGTIFGIYRKNSTDEPSEKDALQPGRNLVAAGYALYGSATMLVLAMVNGVNC
FMLDPAIGEFILVDRDVKIKKKGSIYSINEGYAKEFDPAITEYIQRKKFPPDNSAPYGARYV
GSMVADVHRTLVYGGIFMYPANKKSPKGKLRLLYECNPMAYVMEKAGGLATTGKEAVL
DIVPTDIHQRAPIILGSPEDVTELLEIYQKHAAK
>non-antioxidant_309
MGHHHHHHHHHHSSGHIEGRHMTSNQKAILAGGCFWGLQDLIRNQPGVVSTRVGYSGG
NIPNATYRNHGTHAEAVEIIFDPTVTDYRTLLEFFFQIHDPTTKDRQGNDRGTSYRSAIFYF
DEQQKRIALDTIADVEASGLWPGKVVTEVSPAGDFWEAEPEHQDYLQRYPNGYTCHFVR
PGWRLPRRTAESALRASLSPELGT
>non-antioxidant_310
MTSKIEQPRWASKDSAAGAASTPDEKIVLEFMDALTSNDAAKLIEYFAEDTMYQNMPLPP
AYGRDAVEQTLAGLFTVMSIDAVETFHIGSSNGLVYTERVDVLRALPTGKSYNLSILGVFQ
LTEGKITGWRDYFDLREFEEAVDLPLRG
>non-antioxidant_311
MEHVAFGSEDIENTLAKMDDGQLDGLAFGAIQLDGDGNILQYNAAEGDITGRDPKQVIG
KNFFKDVAPCTDSPEFYGKFKEGVASGNLNTMFEYTFDYQMTPTKVKVHMKKALSGDSY
WVFVKRV

>non-antioxidant_312
MTENFFGKTLAARPVEAIPGMLEFDIPVHGDNRGWFKENFQKEKMLPLGFPESFFAEGKL
QNNVSFSRKNVLRGLHAEPWDKYISVADGGKVLGTWVDLREGETFGNTYQTVIDASKSI
FVPRGVANGFQVLSDFVAYSYLVNDYWALELKPKYAFVNYADPSLDIKWENLEEAEVSEA
DENHPFLKDVKPLRKEDL
>non-antioxidant_313
GSMYQLQFINLVYDTTKLTHLEQTNINLFIGNWSNHQLQKSICIRHGDDTSHNQYHILFIDT
AHQRIKFSSFDNEEIIYILDYDDTQHILMQTSSKQGIGTSRPIVYERLV
>non-antioxidant_314
METYAVFGNPIAHSKSPFIHQQFAQQLNIEHPYGRVLAPINDFINTLNAFFSAGGKGANVTV
PFKEEAFARADELTERAALAGAVNTLMRLEDGRLLGDNTDGVGLLSDLERLSFIRPGLRIL
LIGAGGASRGVLLPLLSLDCAVTITNRTVSRAEELAKLFAHTGSIQALSMDELEGHEFDLII
NATSSGISGDIPAIPSSLIHPGIYCYDMFYQKGKTPFLAWCEQRGSKRNADGLGMLVAQAA
HAFLLWHGVLPDVEPVIKQLQEELS
>non-antioxidant_315
GSTESFTRRERLRLRRDFLLIFKEGKSLQNEYFVVLFRKNGMDYSRLGIVVKRKFGKATRR
NKLKRWVREIFRRNKGVIPKGFDIVVIPRKKLSEEFERVDFWTVREKLLNLLKRIEG
>non-antioxidant_316
MTDTQYIGRFAPSPSGELHFGSLIAALGSYLQARARQGRWLVRIEDIDPPREVPGAAETILR
QLEHYGLHWDGDVLWQSQRHDAYREALAWLHEQGLSYYCTCTRARIQSIGGIYDGHCR
VLHHGPDNAAVRIRQQHPVTQFTDQLRGIIHADEKLAREDFIIHRRDGLFAYNLAVVVDDH
FQGVTEIVRGADLIEPTVRQISLYQLFGWKVPDYIHLPLALNPQGAKLSKQNHAPALPKGD
PRPVLIAALQFLGQQAEAHWQDFSVEQILQSAVKNWRLTAVPESAIVNSTFSNASC
>non-antioxidant_317
EEDPDLKAAIQESLREAEEA
>non-antioxidant_318
MFKAVLFDLDGVITDTAEYHFRAWKALAEEIGINGVDRQFNEQLKGVSREDSLQKILDLA
DKKVSAEEFKELAKRKNDNYVKMIQDVSPADVYPGILQLLKDLRSNKIKIALASASKNGP
FLLERMNLTGYFDAIADPAEVAASKPAPDIFIAAAHAVGVAPSESIGLEDSQAGIQAIKDSG
ALPIGVGRPEDLGDDIVIVPDTSHYTLEFLKEVWLQKQK
>non-antioxidant_319
MGSDKIHHHHHHMKIDILDKGFVELVDVMGNDLSAVRAARVSFDMGLKDEERDRHLIEY
LMKHGHETPFEHIVFTFHVKAPIFVARQWFRHRIASYNELSGRYSKLSYEFYIPSPERLEGY
KTTIPPERVTEKISEIVDKAYRTYLELIESGVPREVARIVLPLNLYTRFFWTVNARSLMNFLN
LRADSHAQWEIQQYALAIARIFKEKCPWTFEAFLKYAYKGDILKEVQV
>non-antioxidant_320
MGSDKIHHHHHHVWEFYMPTDVFFGEKILEKRGNIIDLLGKRALVVTGKSSSKKNGSLDD
LKKLLDETEISYEIFDEVEENPSFDNVMKAVERYRNDSFDFVVGLGGGSPMDFAKAVAVLL
KEKDLSVEDLYDREKVKHWLPVVEIPTTAGTGSEVTPYSILTDPEGNKRGCTLMFPVYAFL
DPRYTYSMSDELTLSTGVDALSHAVEGYLSRKSTPPSDALAIEAMKIIHRNLPKAIEGNRE
ARKKMFVASCLAGMVIAQTGTTLAHALGYPLTTEKGIKHGKATGMVLPFVMEVMKEEIP
EKVDTVNHIFGGSLLKFLKELGLYEKVAVSSEELEKWVEKGSRAKHLKNTPGTFTPEKIRN
IYREALGV
>non-antioxidant_321

MDIAQDWNGIPVPANPGNGMTWQLQDNVSDSFNYTSSEGNRPTAFTSKWKPSYINGWTG
PGSTIFNAPQAWTNGSQLAIQAQPAGNGKSYNGIITSKNKIQYPVYMEIKAKIMDQVLANA
FWTLTDDETQEIDIMEGYGSDRGGTWFAQRMHLSHHTFIRNPFTDYQPMGDATWYYNGG
TPWRSAYHRYGCYWKDPFTLEYYIDGVKVRTVTRAEIDPNNHLGGTGLNQATNIIIDCEN
QTDWRPAATQEELADDSKNIFWVDWIRVYKPVAVSGGGNNSLEHHHHHH
>non-antioxidant_322
MEEKVGNLKPNMESVNVTVRVLEASEARQIQTKNGVRTISEAIVGDETGRVKLTLWGKH
AGSIKEGQVVKIENAWTTAFKGQVQLNAGSKTKIAEASEDGFPESSQIPENTPTAPQQMR
>non-antioxidant_323
ADKLPNIVILATGGTIAGSAATGTQTTGYKAGALGVDTLINAVPEVKKLANVKGEQFSNM
ASENMTGDVVLKLSQRVNELLARDDVDGVVITHGTDTVEESAYFLHLTVKSDKPVVFVA
AMRPATAISADGPMNLLEAVRVAGDKQSRGRGVMVVINDRIGSARYITKTNASTLDTFRA
NEEGYLGVIIGNRIYYQNRIDKLHTTRSVFDVRGLTSLPKVDILYGYQDDPEYLYDAAIQH
GVKGIVYAGMGAGSVSVRGIAGMRKALEKGVVVMRSTRTGNGIVPPDEELPGLVSDSLN
PAHARILLMLALTRTSDPKVIQEYFHTY
>non-antioxidant_324
MNYNNKILVSESGLSQKHLIHGDEELFQHELKTIFARNWLFLTHDSLIPAPGDYVTAKMGI
DEVIVSRQNDGSIRAFLNVCRHRGKTLVSVEAGNAKGFVCSYHGWGFGSNGELQSVPFE
KDLYGESLNKKCLGLKEVARVESFHGFIYGCFDQEAPPLMDYLGDAAWYLEPMFKHSGG
LELVGPPGKVVIKANWKAPAENFVGDAYHVGWTHASSLRSGESIFSSLAGNAALPPEGAG
LQMTSKYGSGMGVLWDGYSGVHSADLVPELMAFGGAKQERLNKEIGDVRARIYRSHLN
CTVFPNNSMLTCSGVFKVWNPIDANTTEVWTYAIVEKDMPEDLKRRLADSVQRTFGPAGF
WESDDNDNMETASQNGKKYQSRDSDLLSNLGFGEDVYGDAVYPGVVGKSAIGETSYRG
FYRAYQAHVSSSNWAEFEHASSTWHTELTKTTDR
>non-antioxidant_325
ESKLKLSDWFNPFKRPEVVTMTKWKAPVVWEGTYNRAVLDNYYAKQKITVGLTVFAVG
RYIEHYLEEFLTSANKHFMVGHPVIFYIMVDDVSRMPLIELGPLRSFKVFKIKPEKRWQDIS
MMRMKTIGEHIVAHIQHEVDFLFCMDVDQVFQDKFGVETLGESVAQLQAWWYKADPND
FTYERRKESAAYIPFGEGDFYYHAAIFGGTPTQVLNITQECFKGILKDKKNDIEAQWHDES
HLNKYFLLNKPTKILSPEYCWDYHIGLPADIKLVKMSWQTKEYNVVRNNV
>non-antioxidant_326
MAKEFGIPAAVAGTVLNVVEAGGWVTTIVSILTAVGSGGLSLLAAAGRESIKAYLKKEIKK
KGKRAVIAW
>non-antioxidant_327
MTQDELKKAVGWAALQYVQPGTIVGVGTGSTAAHFIDALGTMKGQIEGAVSSSDASTEK
LKSLGIHVFDLNEVDSLGIYVDGADEINGHMQMIKGGGAALTREKIIASVAEKFICIADAS
KQVDILGKFPLPVEVIPMARSAVARQLVKLGGRPEYRQGVVTDNGNVILDVHGMEILDPI
AMENAINAIPGVVTVGLFANRGADVALIGTPDGVKTIVK
>non-antioxidant_328
MKILVAVKQTAALEEDFEIREDGMDVDEDFMMYDLNEWDDFSLEEAMKIKESSDTDVEV
VVVSVGPDRVDESLRKCLAKGADRAVRVWDDAAEGSDAIVVGRILTEVIKKEAPDMVFA
GVQSSDQAYASTGISVASYLNWPHAAVVADLQYKPGDNKAVIRRELEGGMLQEVEINCPA
VLTIQLGINKPRYASLRGIKQAATKPIEEVSLADIGLSANDVGAAQSMSRVRRMYIPEKGRA
TMIEGTISEQAAKIIQIINEFKGA

>non-antioxidant_329
PTLSPEQQEMLQAFSTQSGMNLEWSQKCLQDNNWDYTRSAQAFTHLKAKGEIPEVAFMK
>non-antioxidant_330
RVVTQNEIDRTIPVGAIMMWAADSLPSDAWRFCHGGTVSASDCPLYASRIGTRYGGSSSNP
GLPDMRGLFVRGSGRGSHLTNPNVNGNDQFGKPRLGVGCTGGYVGEVQKQQMSYHKH
AGGFGEYDDSGAFGNTRRSNFVGTRKGLDWDNRSYFTNDGYEIDPASQRNSRYTLNRPE
LIGNETRPWNISLNYIIKVKE
>non-antioxidant_331
MHVVYPGSFDPLTNGHLDVIQRASRLFEKVTVAVLENPSKRGQYLFSAEERLAIIREATAH
LANVEAATFSGLLVDFVRRVGAQAIVKGLRAVSDYEYELQMAHLNRQLYPGLETLFILAA
TRYSFVSSTMVKEIARYGGDVSKLVPPATLRALKAKLGQ
>non-antioxidant_332
MGSVSKANVPKIDVSPLFGDDQAAKMRVAQQIDAASRDTGFFYAVNHGINVQRLSQKTK
EFHMSITPEEKWDLAIRAYNKEHQDQVRAGYYLSIPGKKAVESFCYLNPNFTPDHPRIQAK
TPTHEVNVWPDETKHPGFQDFAEQYYWDVFGLSSALLKGYALALGKEENFFARHFKPDD
TLASVVLIRYPYLDPYPEAAIKTAADGTKLSFEWHEDVSLITVLYQSNVQNLQVETAAGY
QDIEADDTGYLINCGSYMAHLTNNYYKAPIHRVKWVNAERQSLPFFVNLGYDSVIDPFDP
REPNGKSDREPLSYGDYLQNGLVSLINKNGQT
>non-antioxidant_333
STGSATTTPIDSLDDAYITPVQIGTPAQTLNLDFDTGSSDLWVFSSETTASEVDGQTIYTPSK
STTAKLLSGATWSISYGDGSSSSGDVYTDTVSVGGLTVTGQAVESAKKVSSSFTEDSTIDG
LLGLAFSTLNTVSPTQQKTFFDNAKASLDSPVFTADLGYHAPGTYNFGFIDTTAYTGSITYT
AVSTKQGFWEWTSTGYAVGSGTFKSTSIDGIADTGTTLLYLPATVVSAYWAQVSGAKSSSS
VGGYVFPCSATLPSFTFGVGSARIVIPGDYIDFGPISTGSSSCFGGIQSSAGIGINIFGDVALK
AAFVVFNGATTPTLGFASK
>non-antioxidant_334
MSESPMFAANGMPKVNQGAEEDVRILGYDPLASPALLQVQIPATPTSLETAKRGRREAIDII
TGKDDRVLVIVGPCSIHDLEAAQEYALRLKKLSDELKGDLSIIMRAYLEKPRTTVGWKGLI
NDPDVNNTFNINKGLQSARQLFVNLTNIGLPIGSEMLDTISPQYLADLVSFGAIGARTTESQ
LHRELASGLSFPVGFKNGTDGTLNVAVDACQAAAHSHHFMGVTKHGVAAITTTKGNEHC
FVILRGGKKGTNYDAKSVAEAKAQLPAGSNGLMIDYSHGNSNKDFRNQPKVNDVVCEQI
ANGENAITGVMIESNINEGNQGIPAEGKAGLKYGVSITDACIGWETTEDVLRKLAAAVRQ
RREVNKK
>non-antioxidant_335
MNLPTAQEVQGLMARYIELVDVGDIEAIVQMYADDATVEDPFGQPPIHGREQIAAFYRQG
LGGGKVRACLTGPVRASHNGCGAMPFRVEMVWNGQPCALDVIDVMRFDEHGRIQTMQA
YWSEVNLSVREPQ
>non-antioxidant_336
GSSMKISRGLLKTILEAAKSAHPDEFIALLSGSKDVMDELIFLPFVSGSVSAVIHLDMLPIG
MKVFGTVHSHPSPSCRPSEEDLSLFTRFGKYHIIVCYPYDENSWKCYNRKGEEVELEVVE
KD
>non-antioxidant_337
MILVNRETRVLVQGITGREGQFHTKQMLTYGTKIVAGVTPGKGGMEVLGVPVYDTVKEA
VAHHEVDASIIFVPAPAAADAALEAAHAGIPLIVLITEGIPTLDMVRAVEEIKALGSRLIGGN

CPGIISAEETKIGIMPGHVFKRGRVGIISRSGTLTYEAAAALSQAGLGTTTTVGIGGDPVIGT
TFKDLLPLFNEDPETEAVVLIGEIGGSDEEEAAAWVKDHMKKPVVGFIGGRSAPKGKRMG
HAGAIIMGNVGTPESKLRAFAEAGIPVADTIDEIVELVKKALG
>non-antioxidant_338
DTTVSEPAPSCVTLYQSWRYSQADNGCAETVTVKVVYEDDTEGLCYAVAPGQITTVGDG
YIGSHGHARYLARCL
>non-antioxidant_339
SHMTMEQFLTSLDMIRSGCAPKFKLKTEDLDRLRVGDFNFPPSQDLMCYTKCVSLMAGT
VNKKGEFNAPKALAQLPHLVPPEMMEMSRKSVEACRDTHKQFKESCERVYQTAKCFSEN
ADGQFMWP
>non-antioxidant_340
GAMEDMEIAYPITCGESKAILLWKKFVCPGINVKCVKFNDQLISPKHFVHLAGKSTLKDW
KRAIRLGGIMLRKMMDSGQIDFYQHDKVCSNTCRSTK
>non-antioxidant_341
MGSSHHHHHHSSGLVPRGSHMDSQNMTKAAQSLNSIQVALTQTYRGLGNYPATADATAA
SKLTSGLVSLGKISSDEAKNPFIGTNMNIFSFPRNAAANKAFAISVDGLTQAQCKTLITSVG
DMFPYIAIKAGGAVALADLGDFENSAAAAETGVGVIKSIAPASKNLDLTNITHVEKLCKGT
APFGVAFGNS
>non-antioxidant_342
MEYKSSPKRPYLLRAYYDWLVDNSFTPYLVVDATYLGVNVPVEYVKDGQIVLNLSASAT
GNLQLTNDFIQFNARFKGVSRELYIPMGAALAIYARENGDGVMFEPEEIYDE
>non-antioxidant_343
GSSHHHHHHSSGLVPRGSHMKLNRVVVTGYGVTSPIGNTPEEFWNSLATGKIGIGGITKFD
HSDFDVHNAAEIQDFPFDKYFVKKDTNRFDNYSLYALYAAQEAVNHANLDVEALNRDRF
GVIVASGIGGIKEIEDQVLRLHEKGPKRVKPMTLPKALPNMASGNVAMRFGANGVCKSIN
TACSSSNDAIGDAFRSIKFGFQDVMLVGGTEASITPFAIAGFQALTALSTTEDPTRASIPFDK
DRNGFVMGEGSGMLVLESLEHAEKRGATILAEVVGYGNTCDAYHMTSPHPEGQGAIKAI
KLALEEAEISPEQVAYVNAHGTSTPANEKGESGAIVAVLGKEVPVSSTKSFTGHLLGAAGA
VEAIVTIEAMRHNFVPMTAGTSEVSDYIEANVVYGQGLEKEIPYAISNTFGFGGHNAVLAF
KRWENR
>non-antioxidant_344
ASMKETNQKPYKETYGISHITRHDMLQIPEQQKNEKYQVPEFDSSTIKNISSAKGLDVWD
SWPLQNADGTVANYHGYHIVFALAGDPKNADDTSIYMFYQKVGETSIDSWKNAGRVFKD
SDKFDANDSILKDQTQEWSGSATFTSDGKIRLFYTDFSGKHYGKQTLTTAQVNVSASDSSL
NINGVEDYKSIFDGDGKTYQNVQQFIDEGNYSSGDNHTLRDPHYVEDKGHKYLVFEANT
GTEDGYQGEESLFNKAYYGKSTSFFRQESQKLLQSDKKRTAELANGALGMIELNDDYTLK
KVMKPLIASNTVTDEIERANVFKMNGKWYLFTDSRGSKMTIDGITSNDIYMLGYVSNSLT
GPYKPLNKTGLVLKMDLDPNDVTFTYSHFAVPQAKGNNVVITSYMTNRGFYADKQSTFA
PSFLLNIKGKKTSVVKDSILEQGQLTVNK
>non-antioxidant_345
PATGEKKECWSWESYLEEQKAITAPVSLFQDSQAVTHNKNGFKLGMKLEGIDPQHPSMYF
ILTVAEVCGYRLRLHFDGYSECHDFWVNANSPDIHPAGWFEKTGHKLQPPKGYKEEEFSW
SQYLRSTRAQAAPKHLFVSQSHSPPPLGFQVGMKLEAVDRMNPSLVCVASVTDVVDSRFL
VHFDNWDDTYDYWCDPSSPYIHPVGWCQKQGKPLTPPQDYPDPDNFCWEKYLEETGAS

AVPTWAFKVRPPHSFLVNMKLEAVDRRNPALIRVASVEDVEDHRIKIHFDGWSHGYDFWI
DADHPDIHPAGWCSKTGHPLQPPLGPREPSSAS
>non-antioxidant_346
MIIGNCLILKDFSSEPFWGAVEIENGTIKRVLQGEVKVDLDLSGKLVMPALFNTHTHAPMT
LLRGVAEDLSFEEWLFSKVLPIEDRLTEKMAYYGTILAQMEMARHGIAGFVDMYFHEEWI
AKAVRDFGMRALLTRGLVDSNGDDGGRLEENLKLYNEWNGFEGRIFVGFGPHSPYLCSEE
YLKRVFDTAKSLNAPVTIHLYETSKEEYDLEDILNIGLKEVKTIAAHCVHLPERYFGVLKDI
PFFVSHNPASNLKLGNGIAPVQRMIEHGMKVTLGTDGAASNNSLNLFFEMRLASLLQKAQ
NPRNLDVNTCLKMVTYDGAQAMGFKSGKIEEGWNADLVVIDLDLPEMFPVQNIKNHLV
HAFSGEVFATMVAGKWIYFDGEYPTIDSEEVKRELARIEKELYSS
>non-antioxidant_347
HMTDLKASSLRALKLMDLTTLNDDDTDEKVIALCHQAKTPVGNTAAICIYPRFIPIARKTL
KEQGTPEIRIATVTNFPHGNDDIDIALAETRAAIAYGADEVDVVFPYRALMAGNEQVGFDL
VKACKEACAAANVLLKVIIETGELKDEALIRKASEISIKAGADFIKTSTGKVAVNATPESAR
IMMEVIRDMGVEKTVGFKPAGGVRTAEDAQKYLAIADELFGADWADARHYRFGASSLLA
SLLKALGHGDGKSASSY
>non-antioxidant_348
GSHMATPPKRSCPSFSASSEGTRIKKISIEGNIAAGKSTFVNILKQLCEDWEVVPEPVARWC
NVQSTQDEFEELTMSQKNGGNVLQMMYEKPERWSFTFQTYACLSRIRAQLASLNGKLKD
AEKPVLFFERSVYSDRYIFASNLYESECMNETEWTIYQDWHDWMNNQFGQSLELDGIIYL
QATPETCLHRIYLRGRNEEQGIPLEYLEKLHYKHESWLLHRTLKTNFDYLQEVPILTLDVN
EDFKDKYESLVEKVKEFLSTL
>non-antioxidant_349
GSSMVTGGMASKWDQKGMDIAYEEAALGYKEGGVPIGGCLINNKDGSVLGRGHNMRF
QKGSATLHGEISTLENCGRLEGKVYKDTTLYTTLSPCDMCTGAIIMYGIPRCVVGENVNFK
SKGEKYLQTRGHEVVVVDDERCKKIMKQFIDERPQDWFEDIGE
>non-antioxidant_350
ETCASRCPRPCNAGLCCSIYGYCGSGAAYCGAGNCRCQCRG
>non-antioxidant_351
MDQLNALLASLEAENKQLKAKVEELLAKVGE
>non-antioxidant_352
AELVSDKALESAPTVGWASQNGFTTGGAAATSDNIYIVTNISEFTSALSAGAEAKIIQIKGTI
DISGGTPYTDFADQKARSQINIPANTTVIGLGTDAKFINGSLIIDGTDGTNNVIIRNVYIQTPI
DVEPHYEKGDGWNAEWDAMNITNGAHHVWIDHVTISDGNFTDDMYTTKDGETYVQHD
GALDIKRGSDYVTISNSLIDQHDKTMLIGHSDSNGSQDKGKLHVTLFNNVFNRVTERAPR
VRYGSIHSFNNVFKGDAKDPVYRYQYSFGIGTSGSVLSEGNSFTIANLSASKACKVVKKF
NGSIFSDNGSVLNGSAVDLSGCGFSAYTSKIPYIYDVQPMTTELAQSITDNAGSGKL
>non-antioxidant_353
DLVYAAEKIIQKRVKKGVVEYRVKWKGWNQRYNTWEPEVNILDRRLIDIYEQTNK
>non-antioxidant_354
MEIPVIEPLFTKVTEDIPGAEGPVFDKNGDFYIVAPEVEVNGKPAGEILRIDLKTGKKTVIC
KPEVNGYGGIPAGCQCDRDANQLFVADMRLGLLVVQTDGTFEEIAKKDSEGRRMQGCND
CAFDYEGNLWITAPAGEVAPADYTRSMQEKFGSIYCFTTDGQMIQVDTAFQFPNGIAVRH
MNDGRPYQLIVAETPTKKLWSYDIKGPAKIENKKVWGHIPGTHEGGADGMDFDEDNNLL

VANWGSSHIEVFGPDGGQPKMRIRCPFEKPSNLHFKPQTKTIFVTEHENNAVWKFEWQRN
GKKQYCETLKFGIF
>non-antioxidant_355
ESSVNPVVLDFEDGTVMSFGEAWGDSLKCIKKVSVSQDLQRPGNKYALRLDVEFNPNNG
WDQGDLGTWIGGVVEGQFDFTGYKSVEFEMFIPYDEFSKSQGGFAYKVVINDGWKELGS
EFNITANAGKKVKINGKDYTVIHKAFAIPEDFRTKKRAQLVFQFAGQNSNYKGPIYLDNVR
IRPEDA
>non-antioxidant_356
MAKKTSSKGKLPPGPSPLPVLGNLLQMDRKGLLRSFLRLREKYGDVFTVYLGSRPVVVL
CGTDAIREALVDQAEAFSGRGKIAVVDPIFQGYGVIFANGERWRALRRFSLATMRDFGMG
KRSVEERIQEEARCLVEELRKSKGALLDNTLLFHSITSNIICSIVFGKRFDYKDPVFLRLLDL
FFQSFSLISSFSSQVFELFSGFLKHFPGTHRQIYRNLQEINTFIGQSVEKHRATLDPSNPRDFI
DVYLLRMEKDKSDPSSEFHHQNLILTVLSLFFAGTETTSTTLRYGFLLMLKYPHVTERVQK
EIEQVIGSHRPPALDDRAKMPYTDAVIHEIQRLGDLIPFGVPHTVTKDTQFRGYVIPKNTEV
FPVLSSALHDPRYFETPNTFNPGHFLDANGALKRNEGFMPFSLGKRICLGEGIARTELFLFF
TTILQNFSIASPVPPEDIDLTPRESGVGNVPPSYQIRFLARHHHH
>non-antioxidant_357
ASDDNVFQPVDQLPEDLIPSSIQVLKFSGKYLKLEQDKAYFDWPGFKTAIDNYTGEDLSFD
KYDQSTINQQSQEVGAMVDKIAKFLHDAFAAVVDLSKLAAIILNTFTNLEEESSSGFLQFN
TNNVKKNSSWEYRVLFSVPFGDNAPSYFYSLVTTILITADIEEKTGWWGLTSSTKKNFAVQ
IDALELVVKKGFKAPN
>non-antioxidant_358
IVGGTSASAGDFPFIVSISRNGGPWCGGSLLNANTVLTAAHCVSGYAQSGFQIRAGSLSRTS
GGITSSLSSVRVHPSYSGNNNDLAILKLSTSIPSGGNIGYARLAASGSDPVAGSSATVAGWG
ATSEGGSSTPVNLLKVTVPIVSRATCRAQYGTSAITNQMFCAGVSSGGKDSCQGDSGGPIV
DSSNTLIGAVSWGNGCARPNYSGVYASVGALRSFIDTYA
>non-antioxidant_359
MRGSHHHHHHGLVPRGSMIRTMLQGKLHRVKVTHADLHYEGTCAIDQDFLDAAGILENE
AIDIWNVTNGKRFSTYAIAAERGSRIISVNGAAAHCASVGDIVIIASFVTMPDEEARTWRPN
VAYFEGDNEMKRTAKAIPVQVA
>non-antioxidant_360
SNTQAERSIIGMIDMFHKYTRRDDKIDKPSLLTMMKENFPNFLSACDKKGTNYLADVFEK
KDKNEDKKIDFSEFLSLLGDIATDYHKQSHGAAPCSGGSQ
>non-antioxidant_361
GSPGIRMSLKSDEVFAKIAKRLESIDPANRQVEHVYKFRITQGGKVVKNWVMDLKNVKL
VESDDAAEATLTMEDDIMFAIGTGALPAKEAMAQDKMEVDGQVELIFLLEPFIASLK
>non-antioxidant_362
EKVIIEKAAGDAEAIAFDGRTYMEYHNAVTKSPDALDYPAEPSEKALQSNHFELSIKTEAT
QGLILWSGKGLERSDYIALAIVDGFVQMMYDLGSKPVVLRSTVPINTNHWTHIKAYRVQR
EGSLQVGNEAPITGSSPLGATQLDTDGALWLGGMERLSVAHKLPKAYSTGFIGCIRDVIVD
RQELHLVEDALNNPTILHCSAK
>non-antioxidant_363
MSERIVPSGDVELWSDDFGDPADPALLLVMGGNLSALGWPDEFARRLADGGLHVIRYDH
RDTGRSTTRDFAAHPYGFGELAADAVAVLDGWGVDRAHVVGLSMGATITQVIALDHHDR

LSSLTMLLGGGLDIDFDANIERVMRGEPTLDGLPGPQQPFLDALALMNQPAEGRAAEVAK
RVSKWRILSGTGVPFDDAEYARWEERAIDHAGGVLAEPYAHYSLTLPPPSRAAELREVTV
PTLVIQAEHDPIAPAPHGKHLAGLIPTARLAEIPGMGHALPSSVHGPLAEVILAHTRSAA
>non-antioxidant_364
GTQGFAVLSYVYEHEKRDLASRIVSTQHHHHDLSVATLHVHINHDDCLEIAVLKGDMGDV
QHFADDVIAQRGVRHGHLQCLPKED
>non-antioxidant_365
MSLPMLQVALDNQTMDSAYETTRLIAEEVDIIEVGTILCVGEGVRAVRDLKALYPHKIVLA
DAKIADAGKILSRMCFEANADWVTVICCADINTAKGALDVAKEFNGDVQIELTGYWTWE
QAQQWRDAGIGQVVYHRSRDAQAAGVAWGEADITAIKRLSDMGFKVTVTGGLALEDLP
LFKGIPIHVFIAGRSIRDAASPVEAARQFKRSIAELWG
>non-antioxidant_366
MASVHGTTYELLRRQGIDTVFGNPGSNALPFLKDFPEDFRYILALQEACVVGIADGYAQA
SRKPAFINLHSAAGTGNAMGALSNAWNSHSPLIVTAGQQTRAMIGVEALLTNVDAANLPR
PLVKWSYEPASAAEVPHAMSRAIHMASMAPQGPVYLSVPYDDWDKDADPQSHHLFDRH
VSSSVRLNDQDLDILVKALNSASNPAIVLGPDVDAANANADCVMLAERLKAPVWVAPSA
PRCPFPTRHPCFRGLMPAGIAAISQLLEGHDVVLVIGAPVFRYHQYDPGQYLKPGTRLISVT
CDPLEAARAPMGDAIVADIGAMASALANLVEESSRQLPTAAPEPAKVDQDAGRLHPETVF
DTLNDMAPENAIYLNESTSTTAQMWQRLNMRNPGSYYFCAAGGLGFALPAAIGVQLAEP
ERQVIAVIGDGSANYSISALWTAAQYNIPTIFVIMNNGTYGALRWFAGVLEAENVPGLDVP
GIDFRALAKGYGVQALKADNLEQLKGSLQEALSAKGPVLIEVSTVSPVK
>non-antioxidant_367
MSLSTPLQGIKVLDFTGVQSGPSCTQMLAWFGADVIKIERPGVGDVTRHQLRDIPDIDALY
FTMLNSNKRSIELNTKTAEGKEVMEKLIREADILVENFHPGAIDHMGFTWEHIQEINPRLIF
GSIKGFDECSPYVNVKAYENVAQAAGGAASTTGFWDGPPLVSAAALGDSNTGMHLLIGL
LAALLHREKTGRGQRVTMSMQDAVLNLCRVKLRDQQRLDKLGYLEEYPQYPNGTFGDA
VPRGGNAGGGGQPGWILKCKGWETDPNAYIYFTIQEQNWENTCKAIGKPEWITDPAYSTA
HARQPHIFDIFAEIEKYTVTIDKHEAVAYLTQFDIPCAPVLSMKEISLDPSLRQSGSVVEVEQ
PLRGKYLTVGCPMKFSAFTPDIKAAPLLGEHTAAVLQELGYSDDEIAAMKQNHAIEGGSH
HHHHH
>non-antioxidant_368
MTSKGPEEEHPSVTLFRQYLRIRTVQPKPDYGAAVAFFEETARQLGLGCQKVEVAPGYVV
TVLTWPGTNPTLSSILLNSHTDVVPVFKEHWSHDPFEAFKDSEGYIYARGAQDMKCVSIQ
YLEAVRRLKVEGHRFPRTIHMTFVPDEEVGGHQGMELFVQRPEFHALRAGFALDEGIANP
TDAFTVFYSERSPWWVRV
>non-antioxidant_369
NPWWAAFSRVCKDMNLTLEPEIMPAAGDNRYIRAVGVPALGFSPMNRTPVLLHDHDERL
HEAVFLRGVDIYTRLLPALASVPALPSDS
>non-antioxidant_370
QEAQTELPQARISCPEGTNAYRSYCYYFNEDRETWVDADLYCQNMNSGNLVSVLTQAEG
AFVASLIKESGTDDFNVWIGLHDPKKNRAWHWSSGSLVSYKSWGIGAPSSVNPGYCVSLT
SSTGFQKWKDVPCEDKFSFVCKFKN
>non-antioxidant_371
PKVSVIMTSYNKSDYVAKSISSILSQTFSDFELFIMDDNSNEETLNVIRPFLNDNRVRFYQS

DISGVKERTEKTRYAALINQAIEMAEGEYITYATDDNIYMPDRLLKMVRELDTHPEKAVIY
SASKTYHLNENRDIVKETVRPAAQVTWNAPCAIDHCSVMHRYSVLEKVKEKFGSYWDES
PAFYRIGDARFFWRVNHFYPFYPLDEELDLNYITDQSIHFQLFELEKNEFVRNLPPQRNCRE
LRESLKKLGMG

>non-antioxidant_372
ASPDDNFSPETLQFLRNNTGLDGEQWNNIMKLINKPEQDDLNWIKYYGYCEDIEDERGYT
IGLFGATTGGSRDTHPDGPDLFKAYDAAKGASNPSADGALKRLGINGKMKGSILEIKDSE
KVFCGKIKKLQNDAAWRKAMWETFYNVYIRYSVEQARQRGFTSAVTIGSFVDTALNQGA
TGGSDTLQGLLARSGSSSNEKTFMKNFHAKRTLVVDTNKYNKPPNGKNRVKQWDTLVD
MGKMNLKNVDSEIAQVTDWEMK
>non-antioxidant_373
MSYMLPHLHNGWQVDQAILSEEDRVVVIRFGHDWDPTCMKMDEVLYSIAEKVKNFAVIY
LVDITEVPDFNKMYELYDPCTVMFFFRNKHIMIDLGTGNNNKINWAMEDKQEMVDIIETV
YRGARKGRGLVVSPKDYSTKYRY
>non-antioxidant_374
SIDNCAVGCPTGGSSKVSIVRHAYTLNNNSTTKFANWVAYHITKDTPASGKTRNWKTDPA
LNPADTLAPADYTGANAALKVDRGHQAPLASLAGVSDWESLNYLSNITPQKSDLNQGAW
ARLEDQERKLIDRADISSVYTVTGPLYERDMGKLPGTQKAHTIPSAYWKVIFINNSPAVNH
YAAFLFDQNTPKGADFCQFRVTVDEIEKRTGLIIWAGLPDDVQASLKSKPGVLPELMGCK
N
>non-antioxidant_375
APPPVPKTPAGPLTLSGQGSFFVGGRDVTSETLSLSPKYDAHGTVTVDQMYVRYQIPQRA
KRYPITLIHGCCLTGMTWETTPDGRMGWDEYFLRKGYSTYVIDQSGRGRSATDISAINAV
KLGKAPASSLPDLFAAGHEAAWAIFRFGPRYPDAFKDTQFPVQAQAELWQQMVPDWLGS
MPTPNPTVANLSKLAIKLDGTVLLSHSQSGIYPFQTAAMNPKGITAIVSVEPGECPKPEDVK
PLTSIPVLVVFGDHIEEFPRWAPRLKACHAFIDALNAAGGKGQLMSLPALGVHGNSHMMM
QDRNNLQVADLILDWIGRNTAKPAHGR
>non-antioxidant_376
ASSFVTISGTQFNIDGKVGYFAGTNCYWCSFLTNHADVDSTFSHISSSGLKVVRVWGFND
VNTQPSPGQIWFQKLSATGSTINTGADGLQTLDYVVQSAEQHNLKLIIPFVNNWSDYGGI
NAYVNAFGGNATTWYTNTAAQTQYRKYVQAVVSRYANSTAIFAWELGNEPRCNGCSTDV
IVQWATSVSQYVKSLDSNHLVTLGDEGLGLSTGDGAYPYTYGEGTDFAKNVQIKSLDFGT
FHLYPDSWGTNYTWGNGWIQTHAAACLAAGKPCVFEEYGAQQNPCTNEAPWQTTSLTT
RGMGGDMFWQWGDTFANGAQSNSDPYTVWYNSSNWQCLVKNHVDAIN
>non-antioxidant_377
CGEQNMIGMTPTVIAVHYLDQTEQWEKFGLEKRQEALELIKKGYTQQLAFKQPISAYAAF
NNRPPSTWLTAYVSRVFSLAANLIAIDSQVLCGAVKWLILEKQKPDGVFQEDGPVIHQEMI
GGFRNTKEADVSLTAFVLIALQEARDICEGQVNSLPGSINKAGEYLEASYLNLQRPYTVAI
AGYALALMNKLEEPYLTKFLNTAKDRNRWEEPGQQLYNVEATSYALLALLLLKDFDSVPP
VVRWLNDERYYGGGYGSTQATFMVFQALAQYRADV
>non-antioxidant_378
MMFNKQIFTILILSLSLALAGSGCISEGAEDNVAQEITVDEFSNIRENPVTPWNPEPSAPVID
PTAYIDPQASVIGEVTIGANVMVSPMASIRSDEGMPIFVGDRSNVQDGVVLHALETINEEG

EPIEDNIVEVDGKEYAVYIGNNVSLAHQSQVHGPAAVGDDTFIGMQAFVFKSKVGNNCVL
EPRSAAIGVTIPDGRYIPAGMVVTSQAEADKLPEVTDDYAYSHTNEAVVYVNVHLAEGYK
ETS
>non-antioxidant_379
MKYIGAHVSAAGGLANAAIRAAEIDATAFALFTKNQRQWRAAPLTTQTIDEFKAACEKYH
YTSAQILPHDSYLINLGHPVTEALEKSRDAFIDEMQRCEQLGLSLLNFHPGSHLMQISEED
CLARIAESINIALDKTQGVTAVIENTAGQGSNLGFKFEHLAAIIDGVEDKSRVGVCIDTCHA
FAAGYDLRTPAECEKTFADFARTVGFKYLRGMHLNDAKSTFGSRVDRHHSLGEGNIGHDA
FRWIMQDDRFDGIPLILETINPDIWAEEIAWLKAQQTEKAVA
>non-antioxidant_380
MSKTIATENAPAAIGPYVQGVDLGNMIITSGQIPVNPKTGEVPADVAAQARQSLDNVKAIV
EAAGLKVGDIVKTTVFVKDLNDFATVNATYEAFFTEHNATFPARSCVEVARLPKDVKIEIE
AIAVRR
>non-antioxidant_381
MTVAKAIFIKCGNLGTSMMMDMLLDERADREDVEFRVVGTSVKMDPECVEAAVEMALD
IAEDFEPDFIVYGGPNPAAPGPSKAREMLADSEYPAVIIGDAPGLKVKDEMEEQGLGYILV
KPDAMLGARREFLDPVEMAIYNADLMKVLAATGVFRVVQEAFDELIEKAKEDEISENDLP
KLVIDRNTLLEREEFENPYAMVKAMAALEIAENVADVSVEGCFVEQDKERYVPIVASAHE
MMRKAAELADEARELEKSNDAVLRTPHAPDGKVLSKRKFMEDPE
>non-antioxidant_382
NYFQGHMMQIDSIEIGGKVYQFFKSDLGNAPLLFIKGSKGYAMCGYLNMETSNKVGDIAV
RVMGVKTLDDMLSAKVVEASQEAQKVGINPGDVLRNVIDKLG
>non-antioxidant_383
MATKKATMIIEKDFKIAEIDKRIYGSFIEHLGRAVYGGIYEPGHPQADENGFRQDVIELVKE
LQVPIIRYPGGNFVSGYNWEDGVGPKEQRPRRLDLAWKSVETNEIGLNEFMDWAKMVGA
EVNMAVNLGTRGIDAARNLVEYCNHPSGSYYSDLRIAHGYKEPHKIKTWCLGNAMDGP
WQIGHKTAVEYGRIACEAAKVMKWVDPTIELVVCGSSNRNMPTFAEWEATVLDHTYDH
VDYISLHQYYGNRDNDTANYLALSLEMDDFIRSVVAIADYVKAKKRSKKTIHLSFDEWN
VWYHSNEADKLIEPWTVAPPLLEDIYNFEDALLVGCMLITLMKHADRVKIACLAQLVNVI
APIMTEKNGPAWKQTIYYPFMHASVYGRGVALHPVISSPKYDSKDFTDVPYLESIAVYNEE
KEEVTIFAVNRDMEDALLLECDVRSFEDYRVIEHIVLEHDNVKQTNSAQSSPVVPHRNGD
AQLSDRKVSATLPKLSWNVIRLGKR
>non-antioxidant_384
MKAFEFLYEDFQRGLTVVLDKGLPPKFVEDYLKVCGDYIDFVKFGWGTSAVIDRDVVKE
KINYYKDWGIKVYPGGTLFEYAYSKGKFDEFLNECEKLGFEAVEISDGSSDISLEERNNAIK
RAKDNGFMVLTEVGKKMPDKDKQLTIDDRIKLINFDLDAGADYVIIEGRESGKGKGLFDK
EGKVKENELDVLAKNVDINKVIFEAPQKSQQVAFILKFGSSVNLANIAFDEVISLETLRRGL
RGDTFGKV
>non-antioxidant_385
SAGSKSCDTVDLGYQCSPATSHLWGQYSPFFSLEDELSVSSKLPKDCRITLVQVLSRHGAR
YPTSSKSKKYKKLVTAIQANATDFKGKFAFLKTYNYTLGADDLTPFGEQQLVNSGIKFYQR
YKALARSVVPFIRASGSDRVIASGEKFIEGFQQAKLADPGATNRAAPAISVIIPESETFNNTL
DHGVCTKFEASQLGDEVAANFTALFAPDIRARAEKHLPGVTLTDEDVVSLMDMCSFDTVA
RTSDASQLSPFCQLFTHNEWKKYNYLQSLGKYYGYGAGNPLGPAQGIGFTNELIARLTRS

PVQDHTSTNSTLVSNPATFPLNATMYVDFSHDNSMVSIFFALGLYNGTEPLSRTSVESAKEL
DGYSASWVVPFGARAYFETMQCKSEKEPLVRALINDRVVPLHGCDVDKLGRCKLNDFVK
GLSWARSGGNWGECFS
>non-antioxidant_386
AETTNTQQAHTQMSTQSQDVSYGTYYTIDSNGDYHHTPDGNWNQAMFDNKEYSYTFVD
AQGHTHYFYNCYPKNANANGSGQTYVNPATAGDNNDYTASQSQQHINQYGYQSNVGPD
ASYYSHSNNNQAYNSHDGNGKVNYPNGTSNQNGGSASKATASGHAKDASWLTSRKQLQ
PYGQYHGGGAHYGVDYAMPENSPVYSLTDGTVVQAGWSNYGGGNQVTIKEANSNNYQ
WYMHNNRLTVSAGDKVKAGDQIAYSGSTGNSTAPHVHFQRMSGGIGNQYAVDPTSYLQS
R
>non-antioxidant_387
GSADSQIQFTRHASDVLLNLNRLRSRDILTDVVIVVSREQFRAHKTVLMACSGLFYSIFTD
QLKRNLSVINLDPEINPEGFNILLDFMYTSRLNLREGNIMAVMATAMYLQMEHVVDTCRK
FIKASE
>non-antioxidant_388
GSHMSPLLQPGLAALRRRAREAGVPLAPLPLTDSFLLRFLRARDFDLDLAWRLLKNYYK
WRAECPEISADLHPRSIIGLLKAGYHGVLRSRDPTGSKVLIYRIAHWDPKVFTAYDVFRVS
LITSELIVQEVETQRNGIKAIFDLEGWQFSHAFQITPSVAKKIAAVLTDSFPLKVRGIHLINEP
VIFHAVFSMIKPFLTEKIKERIHMHGNNYKQSLLQHFPDILPLEYGGEEFSMEDICQEWTNF
IMKSEDYLSSISESIQ
>non-antioxidant_389
SESNKAGEDGASTVERETQEDDTNSIDSSDDLDGFVKILKEIVKLDNIVSSTWNPLDESILA
YGEKNSVARLARIVETDQEGKKYWKLTIIAELRHPFALSASSGKTTNQVTCLAWSHDGNSI
VTGVENGELRLWNKTGALLNVLNFHRAPIVSVKWNKDGTHIISMDVENVTILWNVISGT
VMQHFELKETGGSSINAENHSGDGSLGVDVEWVDDDKFVIPGPKGAIFVYQITEKTPTGK
LIGHHGPISVLEFNDTNKLLLSASDDGTLRIWHGGNGNSQNCFYGHSQSIVSASWVGDDK
VISCSMDGSVRLWSLKQNTLLALSIVDGVPIFAGRISQDGQKYAVAFMDGQVNVYDLKKL
NSKSRSLYGNRDGILNPLPIPLYASYQSSQDNDYIFDLSWNCAGNKISVAYSLQEGSVVAIP
G
>non-antioxidant_390
GAMDPRTITMHKDSTGHVGFIFKNGKITSIVKDSSAARNGLLTEHNICEINGQNVIGLKDS
QIADILSTSGTVVTITIMPAF
>non-antioxidant_391
PAPHGGILQDLIARDALKKNELLSEAQSSDILVWNLTPRQLCDIELILNGGFSPLTGFLNEN
DYSSVVTDSRLADGTLWTIPITLDVDEAFANQIKPDTRIALFQDDEIPIAILTVQDVYKPNK
TIEAEKVFRGDPEHPAISYLFNVAGDYYVGGSLEAIQLPQHYDYPGLRKTPAQLRLEFQSR
QWDRVVAFQTRNPMHRAHRELTVRAAREANAKVLIHPVVGLTKPGDIDHHTRVRVYQEII
KRYPNGIAFLSLLPLAMRMSGDREAVWHAIIRKNYGASHFIVGRDHAGPGKNSKGVDFY
GPYDAQELVESYKHELDIEVVPFRMVTYLPDEDRYAPIDQIDTTKTRTLNISGTELRRRLRV
GGEIPEWFSYPEVVKILRESNPPRPKGSS
>non-antioxidant_392
AKKKSKLEIIQAILEACKSGSPKTRIMYGANLSYALTGRYIKMLMDLEIIRQEGKQYMLTK
KGEELLEDIRKFNEMRKNMDQLKEKINSVLSIRQ
>non-antioxidant_393

ADLPGKGITVNPVQSTITEETFQTLLVSRALEKLGYTVNKPSEVDYNVGYTSLASGDATFT
AVNWTPLHDNMYEAAGGDKKFYREGVFVNGAAQGYLIDKKTADQYKITNIAQLKDPKI
AKLFDTNGDGKADLTGCNPGWGCEGAINHQLAAYELTNTVTHNQGNYAAMMADTISRY
KEGKPVFYYTWTPYWVSNELKPGKDVVWLQVPFSALPGDKNADTKLPNGANYGFPVST
MHIVANKAWAEKNPAAAKLFAIMQLPVADINAQNAIMHDGKASEGDIQGHVDGWIKAHQ
QQFDGWVNEALAAQK
>non-antioxidant_394
GSSMANNALQTIINARLPGEEGLWQIHLQDGKISAIDAQSGVMPITENSLDAEQGLVIPPFV
EPHIHLDTTQTAGQPNWNQSGTLFEGIERWAERKALLTHDDVKQRAWQTLKWQIANGIQ
HVRTHVDVSDATLTALKAMLEVKQEVAPWIDLQIVAFPQEGILSYPNGEALLEEALRLGA
DVVGAIPHFEFTREYGVESLHKTFALAQKYDRLIDVHCDEIDDEQSRFVETVAALAHHEG
MGARVTASHTTAMHSYNGAYTSRLFRLLKMSGINFVANPLVNIHLQGRFDTYPKRRGITR
VKEMLESGINVCFGHDGVFDPWYPLGTANMLQVLHMGLHVCQLMGYGQINDGLNLITH
HSARTLNLQDYGIAAGNSANLIILPAENGFDALRRQVPVRYSVRGGKVIASTQPAQTTVYL
EQPEAIDYKR
>non-antioxidant_395
MRPARALIDLQALRHNYRLAREATGARALAVIKADAYGHGAVRCAEALAAEADGFAVACI
EEGLELREAGIRQPILLLEGFFEASELELIVAHDFWCVVHCAWQLEAIERASLARPLNVWL
KMDSGMHRVGFFPEDFRAAHERLRASGKVAKIVMMSHFSRADELDCPRTEEQLAAFSAA
SQGLEGEISLRNSPAVLGWPKVPSDWVRPGILLYGATPFERAHPLADRLRPVMTLESKVIS
VRDLPAGEPVGYGARYSTERRQRIGVVAMGYADGYPRHAADGTLVFIDGKPGRLVGRVS
MDMLTVDLTDHPQAGLGSRVELWGPNVPVGALAAQFGSIPYQLLCNLKRVPRVYSGA
>non-antioxidant_396
GNAAAAKKGSEQESVKEFLAKAKEDFLKKWETPSQNTAQLDQFDRIKTLGTGSFGRVML
VKHKESGNHYAMKILDKQKVVKLKQIEHTLNEKRILQAVNFPFLVKLEFSFKDNSNLYMV
MEYVAGGEMFSHLRRIGRFSEPHARFYAAQIVLTFEYLHSLDLIYRDLKPENLLIDQQGYIQ
VTDFGFAKRVKGRTWTLCGTPEALAPEIILSKGYNKAVDWWALGVLIYEMAAGYPPFFAD
QPIQIYEKIVSGKVRFPSHFSSDLKDLLRNLLQVDLTKRFGNLKNGVNDIKNHKWFATTD
WIAIYQRKVEAPFIPKFKGPGDTSNFDDYEEEEIRVSINEKCGKEFTEF
>non-antioxidant_397
MELEDANVTKKVELRPLIGLTRGLPPTDLETITIDAIRTHRRLVEKADELFQALPETYKTGQ
ACGGPQHIRYIEASIEMHAQMSALNTLISILGFIPKVVVN
>non-antioxidant_398
HHHHHHFNLPPGNYKKPKLLYCSNGGHFLRILPDGTVDGTRDRSDQHIQLQLSAESVGEV
YIKSTETGQYLAMDTDGLLYGSQTPNEECLFLERLEENHYNTYISKKHAEKNWFVGLKK
NGSCKRGPRTHYGQKAILFLPLPVSSD
>non-antioxidant_399
HECQCQCGSCKNNEQCQKSCSCPTGCNSDDKCPCGN
>non-antioxidant_400
MKKHIIIKTIPKKEEIISRDLCDCIYYYDNSVICKPIGPSKVYVSTSLENLEKCLQLHYFKKL
VKNIEIFDEVHNSKPNCDKCLIVEIGGVYFVRRVNGVPR
>non-antioxidant_401
GASIVSLLGIKVLNNPAKFTDPYEFEITFECLESLKHDLEWKLTYVGSSRSLDHDQELDSIL
VGPVPVGVNKFVFSADPPSAELIPASELVSVTVILLSCSYDGREFVRVGYYVNNEYDEEEL

RENPPAKVQVDHIVRNILAEKPRVTRFNIVWD
>non-antioxidant_402
GRGSAGYDLNAFTFDPIKESIVSREMTRRYMTDMITYAETDVVVVGAGSAGLSAAYEISK
NPNVQVAIIEQSVSPGGGAWLGGQLFSAMIVRKPAHLFLDEIGVAYDEQDTYVVVKHAAL
FTSTIMSKLLARPNVKLFNAVAAEDLIVKGNRVGGVVTNWALVAQNHHTQSCMDPNVME
AKIVVSSCGHDGPFGATGVKRLKSIGMIDHVPGMKALDMNTAEDAIVRLTREVVPGMIVT
GMEVAEIDGAPRMGPTFGAMMISGQKAGQLALKALGLPNAIDGTL
>non-antioxidant_403
MPPITQQATVTAWLPQVDASQITGTISSLESFTNRFYTTTSGAQASDWIASEWQALSASLP
NASVKQVSHSGYNQKSVVMTITGSEAPDEWIVIGGHLDSTIGSHTNEQSVAPGADDDASG
IAAVTEVIRVLSENNFQPKRSIAFMAYAAEEVGLRGSQDLANQYKSEGKNVVSALQLDMT
NYKGSAQDVVFITDYTDSNFTQYLTQLMDEYLPSLTYGFDTCGYACSDHASWHNAGYPA
AMPFESKFNDYNPRIHTTQDTLANSDPTGSHAKKFTQLGLAYAIEMGSATGDTPTPGNQ
>non-antioxidant_404
MSLSDDIKVLGISGSLRSGSYNSAALQEAIGLVPPGMSIELADISGIPLYNEDVYALGFPPAV
ERFREQIRAADALLFATPEYNYSMAGVLKNAIDWASRPPEQPFSGKPAAILGASAGRFGTA
RAQYHLRQTLVFLDVHPLNKPEVMISSAQNAFDAQGRLLDDKARELIQQQLQALQLWVR
EGGSHHHHHH
>non-antioxidant_405
ADCSSDLTSGISTKRIYYVAPNGNSSNNGSSFNAPMSFSAAMAAVNPGELILLKPGTYTIPY
TQGKGNTITFNKSGKDGAPIYVAAANCGRAVFDFSFPDSQWVQASYGFYVTGDYWYFKG
VEVTRAGYQGAYVIGSHNTFENTAFHHNRNTGLEINNGGSYNTVINSDAYRNYDPKKNG
SMADGFGPKQKQGPGNRFVGCRAWENSDDGFDLFDSPQKVVIENSWAFRNGINYWNDS
AFAGNGNGFKLGGNQAVGNHRITRSVAFGNVSKGFDQNNNAGGVTVINNTSYKNGINYG
FGSNVQSGQKHYFRNNVSLSASVTVSNADAKSNSWDTGPAASASDFVSLDTSLATVSRD
NDGTLPETSLFRLSANSKLINAGTKESNISYSGSAPDLGAFERN
>non-antioxidant_406
GSLSWKRCAGCGGKIADRFLLYAMDSYWHSRCLKCSSCQAQLGDIGTSSYTKSGMILCR
NDYIRLFGNSGACSACGQSIPASELVMRAQGNVYHLKCFTCSTCRNRLVPGDRFHYINGSL
FCEHDRPTALINGHLNSGGSGGSGGSGGDVMVVGEPTLMGGEFGDEDERLITRLENTQFD
AANGIDDE
>non-antioxidant_407
MKTITETLNLAPKGKNFLTADWPAPANVKTLITTRNGGVSQGAYQSLNLGTHVGDNPEAV
RRNREIVQQQVGLPVAYLNQIHSTVVVNAAEALGGTPDADASVDDTGKVACAVMTADCL
PVLFCDRAGTAVAAAHAGWRGLAGGVLQNTIAAMKVPPVEMMAYLGPAISADAFEVGQ
DVFDAFCTPMPEAATAFEGIGSGKFLADLYALARLILKREGVGGVYGGTHCTVLERDTFFS
YRRDGATGRMASLIWLDGNAV
>non-antioxidant_408
TYVLYGIKACDTMKKARTWLDEHKVAYDFHDYKAVGIDREHLRRWCAEHGWQTVLNR
AGTTFRKLDEAQKADLDEAKAIELMLAQPSMIKRPVLELGGRTLVGFKPDAYAAALA
>non-antioxidant_409
EAEPGAAEFAALRNRWVDQITGRNVIQAGDPDFAKAITALNNKAADSLAKLDAAAGRTS
VFTDLSLAKDAEMVTTYTRLSQLATAWATPTAAVFGDAAVLAAIKAGLADANTLCYNDR
KEEVGNWWSWEIGVPRALADAMVLLHAELSAAERTAYCAAIDHFVPDPWLQFPPKRGKI

TSVGANRVDLCQGIIIRSLAGEDPTKLNHAVAGLSQVWQYVTSGDGIFRDGSFIQHSTTPY
TGSYGVVLLTGLSKLFSLLGGTAFEVSDPTRSIFFDAVEGSFAPVMINGAMADAVRGRSISR
EANTGYDLGASAIEAILLLARAMDPATAARWRGLCAGWIARDTYRPILNSASVPRTALVK
QLEATGVAPVAEATGHKLFPAMDRTMHRGPGWALSLALSSNRIAWYECGNGENNRGYHT
GSGMTYFYTSDLGQYDDAFWATANYNRLPGITVDTTPLPDKVEGQWGAAVPADEWSGAT
ALGEVAAVGQHLVGPGRTGLTARKSWFVSGDVTVCLGADISTASGAKVETIVDHRNLHQ
GSNTLTTAAGTIAGTAGTVEVLGDGRWVHLEGFGGYAMLDDSPLHVLRETRSGSWSGVN
INGSATVQQRNFATLYVNHGVGPVAGSYAYMVAPGASVDLTRKLLEGNKYSVIRNDATAQ
SVEFKTAKTTAATFWKPGMAGDLGASGPACVVFSRHGNELSLAVSEPTQKAAGLTLTLPE
GTWSSVLEGAGTLGTDADGRSTLTLDTTGLSGKTKLIKLKR
>non-antioxidant_410
MGMIGYFAEIDSEKINQLLESTEKPLMDNIHDTLSGLRRLDIDKRWDFLHFGLTGTSAFDP
AKNDPLSRAVLGEHSLEDGIDGFLGLTWNQELAATIDRLESLDRNELRKQFSIKRLNEMEI
YPGVTFSEELEGQLFASIMLDMEKLISAYRRMLRQGNHALTVIVG
>non-antioxidant_411
VPDKTVRWCAVSEHEATKCQSFRDHMKSVIPSDGPSVACVKKASYLDCIRAIAANEADAV
TLDAGLVYDAYLAPNNLKPVVAEFYGSKEDPQTFYYAVAVVKKDSGFQMNQLRGKKSCH
TGLGRSAGWNIPIGLLYCDLPEPRKPLEKAVANFFSGSCAPCADGTDFPQLCQLCPGCGCS
TLNQYFGYSGAFKCLKDGAGDVAFVKHSTIFENLANKADRDQYELLCLDNTRKPVDEYK
DCHLAQVPSHTVVARSMGGKEDLIWELLNQAQEHFGKDKSKEFQLFSSPHGKDLLFKDS
AHGFLKVPPRMDAKMYLGYEYVTAIRNLR
>non-antioxidant_412
AHHHHHHGSSEKACRHCHYITSEDRCPVCGSRDLSEEWFDLVIIVDVENSEIAKKIGAKVP
GKYAIRVR
>non-antioxidant_413
THNAHNWRLGQAPANWYNDTYPLSPPQRTPAGIRYRIAVIADLDTESRAQEENTWFSYLK
KGYLTLSDSGDKVAVEWDKDHGVLESHLAEKGRGMELSDLIVFNGKLYSVDDRTGVVYQ
IEGSKAVPWVILSDGDGTVEKGFKAEWLAVKDERLYVGGLGKEWTTTTGDVVNENPEW
VKVVGYKGSVDHENWVSNYNALRAAAGIQPPGYLIHESACWSDTLQRWFFLPRRASQER
YSEKDDERKGANLLLSASPDFGDIAVSHVGAVVPTHGFSSFKFIPNTDDQIIVALKSEEDSG
RVASYIMAFTLDGRFLLPETKIGSVKYEGIEFI
>non-antioxidant_414
GSHMPLSSENKQKLQKQVEFYFSDVNVQRDIFLKGKMAENAEGFVSLETLLTFKRVNSVT
TDVKEVVEAIRPSEKLVLSEDGLMVRRRDPLP
>non-antioxidant_415
MSNITIYHNPASGTSRNTLEMIRNSGTEPTIILYLENPPSRDELVKLIADMGISVRALLRKNV
EPYEQLGLAEDKFTDDQLIDFMLQHPILINRPIVVTPLGTRLCRPSEVVLDILQDAQKGAFT
KEDGEKVVDEAGKRLK
>non-antioxidant_416
MSVFDSKFKGIHVYSEIGELESVLVHEPGREIDYITPARLDELLFSAILESHDARKEHKQFV
AELKANDINVVELIDLVAETYDLASQEAKDKLIEEFLEDSEPVLSEEHKVVVRNFLKAKKT
SRELVEIMMAGITKYDLGIEADHELIVDPMPNLYFTRDPFASVGNGVTIHYMRYKVRQRE
TLFSRFVFSNHPKLINTPWYYDPSLKLSIEGGDVFIYNNDTLVVGVSERTDLQTVTLLAKNI
VANKECEFKRIVAINVPKWTNLMHLDTWLTMLDKDKFLYSPIANDVFKFWDYDLVNGGA

EPQPVENGLPLEGLLQSIINKKPVLIPIAGEGASQMEIERETHFDGTNYLAIRPGVVIGYSRN
EKTNAALEAAGIKVLPFHGNQLSLGMGNARCMSMPLSRKDVKW
>non-antioxidant_417
SDAMTTFLQRDEFAVTARVLGALFYYSPESHETAPLVQALLNDDWQAQWPLDAEALAPV
AAMFKTHSEESLPQAWQRLFIGPYALPSPPWGSVWLDRESVLFGDSTLALRQWMRENGI
QFEMQQNEPEDHFGSLLLLAAWLAENDRHHECEQLLAWHLFPWSSRFLDVFIDHAGHPF
YQALGQLARLTLAQWQAQLIIPVAVKPLFR
>non-antioxidant_418
MPELEVKGKKLRLDEDGFLQDWEEWDEEVAEALAKDTRFSPQPIELTEEHWKIIRYLRDY
FIKYGVAPPVRMLVKHCKKEVRPDCNLQYIYKLFPQGPAKDACRIAGLPKPTGCV
>non-antioxidant_419
MHHHHHHSSGVDLGTENLYFQSNAMARGIWGVDSAQVVTDQLFQCVRTELGYPKFWGR
YLSEVPNVSEGLTRDEIVRIRNYGVKVLPIYNAFREAVGYANGQVAARNAVFHARRLGIPK
NKLLFANIEDFFAVDAAWIAAWVETLYPTGYRPGLYADPTKGDFAAAYCEAVSRNNQVAV
QAVIWSAAPRPGTTKEQKAPRYQPAAPPCSANVWVWQYGRDAEVCPVDTNLADRRLLD
FLY
>non-antioxidant_420
AGKKVLIVYAHQEPKSFNGSLKNVAVDELSRQGCTVTVSDLYAMNFEPRATDKDITGTLS
NPEVFNYGVETHEAYKQRSLASDITDEQKKVREADLVIFQFPLYWFSVPAILKGWMDRVL
CQGFAFDIPGFYDSGLLQGKLALLSVTTGGTAEMYTKTGVNGDSRYFLWPLQHGTLHFCG
FKVLAPQISFAPEIASEEERKGMVAAWSQRLQTIWKEEPIPCTAHWHFGQ
>non-antioxidant_421
GSQRVLVEPDAGAGVAVMKFKNPPVNSLSLEFLTELVISLEKLENDKSFRGVILTSDRPGVF
SAGLDLTEMCGRSPAHYAGYWKAVQELWLRLYQSNLVLVSAINGACPAGGCLVALTCDYR
ILADNPRYCIGLNETQLGIIAPFWLKDTLENTIGHRAAERALQLGLLFPPAEALQVGIVDQV
VPEEQVQSTALSAIAQWMAIPDHARQLTKAMMRKATASRLVTQRDADVQNFVSFISKDSI
QKSLQMYLERLKEEKG
>non-antioxidant_422
MSRQTEIVRRMVSAFNTGRTDDVDEYIHPDYLNPATLEHGIHTGPKAFAQLVGWVRATFS
EEARLEEVRIEERGPWVKAYLVLYGRHVGRLVGMPPTDRRFSGEQVHLMRIVDGKIRDHR
DWPDFQGTLRQLGDPWPDDEGWRP
>non-antioxidant_423
MGKSASKQFHNEVLKAHNEYRQKHGVPPLKLCKNLNREAQQYSEALASTRILKHSPESS
RGQCGENLAWASYDQTGKEVADRWYSEIKNYNFQQPGFTSGTGHFTAMVWKNTKKMG
VGKASASDGSSFVVARYFPAGNVVNEGFFEENVLPPKK
>non-antioxidant_424
MYRIPSYDFADELAKLLRQAAGM
>non-antioxidant_425
MNKIFIYAGVRNHNSKTLEYTKRLSSIISSRNNVDISFRTPFNSELEISNSDSEELFKKGIDRQ
SNADDGGVIKKELLESDIIIISSPVYLQNVSVDTKNFIERIGGWSHLFRLAGKFVVTLDVAE
SNGSDNVSEYLRDIFSYMGGQILHQVSITNSLKDIAEAQLMEATYKIEDVLEGKIKYKTTD
YQERAYQTLKLILENYDSEHFEKMYWEKKRLFEANSLEEWYYVENIKLEHHHHHH
>non-antioxidant_426
MAKQNLKSTDRAVQQMLDKAKREGIQTVWDRYEAMKPQCGFGETGLCCRHCLQGPCRI

NPFGDEPKVGICGATAEVIVARGLDRSIAAGAAGHSGHAKHLAHTLKKAVQGKAASYMIK
DRTKLHSIAKRLGIPTEGQKDEDIALEVAKAALADFHEKDTPVLWVTTVLPPSRVKVLSAH
GLIPAGIDHEIAEIMHRTSMGCDADAQNLLLGGLRCSLADLAGCYMGTDLADILFGTPAPV
VTESNLGVLKADAVNVAVHGHNPVLSDIIVSVSKEMENEARAAGATGINVVGICCTGNEV
LMRHGIPACTHSVSQEMAMITGALDAMILDYQCIQPSVATIAECTGTTVITTMEMSKITGA
THVNFAEEAAVENAKQILRLAIDTFKRRKGKPVEIPNIKTKVVAGFSTEAIINALSKLNAND
PLKPLIDNVVNGNIRGVCLFAGCNNVKVPQDQNFTTIARKLLKQNVLVVATGCGAGALM
RHGFMDPANVDELCGDGLKAVLTAIGEANGLGGPLPPVLHMGSCVDNSRAVALVAALAN
RLGVDLDRLPVVASAAEAMHEKAVAIGTWAVTIGLPTHIGVLPPITGSLPVTQILTSSVKDIT
GGYFIVELDPETAADKLLAAINERRAGLGLPW
>non-antioxidant_427
TAAVALVKANENAAAILNLKNAIQKTNAAVADVVQATQSLGTAVQAVQDHINSVVSPAITA
ANY
>non-antioxidant_428
QILSIDPLDISQNLAAVNKSLSDALQHLAQSDTYLSAI
>non-antioxidant_429
SLRSDLINALYDENQKYDVCGIISAEGKIYPLGSDTAVLSTIFELFSRPIINKIAEKHGYIVEE
PKQQNHYPDFTLYKPSEPNKKIAIDIKTTYTNKENEKIKFTLGGYTSFIRNNTKNIVYPFDQ
YIAHWIIGYVYTRVATRKSSLKTYNINELNEIPKPYKGVKVFLQDKWVIAGDLAGSGNTTN
IGSIHAHYKDFVEGKGIFDSEDEFLDYWRNYERTSQLRNDKYNNISEYRNWIYRGRK
>non-antioxidant_430
MSHHHHHHGSSRQANRGTESKKMSSELFTLTYGALVTQLCKDYENDEDVNKQLDKMGF
NIGVRLIEDFLARSNVGRCHDFRETADVIAKVAFKMYLGITPSITNWSPAGDEFSLILENNP
LVDFVELPDNHSSLIYSNLLCGVLRGALEMVQMAVEAKFVQDTLKGDGVTEIRMRFIRRI
EDNLPAGEEAAAWSHPQFEK
>non-antioxidant_431
GSSSTLTKELIKDAAEKCCTRNRQECCIEIMKFGTPIRCGYDRDPKLPGYVYKCLQNVLFA
KEPKKKINLDDSVCCSVFGNDQEDSGRRCENRCKNLMTSPSIDAATRLDSIKSCSLLDNVL
YKCFEKCRSLRKDGIKIEVLQFEEYCEATFIQKRTFRGV
>non-antioxidant_432
ERVVINVSGLRFETQLKTLNQFPDTLLGNPQKRNRYYDPLRNEYFFDRNRPSFDAILYFYQ
SGGRLRRPVNVPLDVFSEEIKFYELGENAFERYREDEGF
>non-antioxidant_433
PILEKAPQKMPVKASSWEELDLPKLPVPPLQQTLATYLQCMQHLVPEEQFRKSQAIVKRF
GAPGGLGETLQEKLLERQEKTANWVSEYWLNDMYLNNRLALPVNSSPAVIFARQHFQDT
NDQLRFAACLISGVLSYKTLLDSHSLPTDWAKGQLSGQPLCMKQYYRLFSSYRLPGHTQD
TLVAQKSSIMPEPEHVIVACCNQFFVLDVVINFRRLSEGDLFTQLRKIVKMASNEDERLPPI
GLLTSDGRSEWAKARTVLLKDSTNRDSLDMIERCICLVCLDGPGTGELSDTHRALQLLHG
GGCSLNGANRWYDKSLQFVVGRDGTCGVVCEHSPFDGIVLVQCTEHLLKHMMTSNKKL
VRADSVSELPAPRRLRWKCSPETQGHLASSAEKLQRIVKNLDFIVYKFDNYGKTFIKKQK
YSPDGFIQVALQLAYYRLYQRLVPTYESASIRRFQEGRVDNIRSATPEALAFVQAMTDHKA
AMPASEKLQLLQTAMQAQTEYTVMAITGMAIDNHLLALRELARDLCKEPPEMFMDETYL
MSNRFVLSTSQVPTTMEMFCCYGPVVPNGYGACYNPQPEAITFCISSFHSCKETSSVEFAE
AVGASLVDMRDLCSSRQPADSKPPAPKEKARGPSQAKQS

>non-antioxidant_434
MSGLRVYSTSVTGSREIKSQQSEVTRILDGKRIQYQLVDISQDNALRDEMRTLAGNPKATP
PQIVNGNHYCGDYELFVEAVEQDTLQEFLKLA
>non-antioxidant_435
MAPKAKIVLVGSGMIGGVMATLIVQKNLGDVVLFDIVKNMPHGKALDTSHTNVMAYSN
CKVSGSNTYDDLAGADVVIVTAGFTKAPGKSDKEWNRDDLLPLNNKIMIEIGGHIKKNCP
NAFIIVVTNPVDVMVQLLHQHSGVPKNKIIGLGGVLDTSRLKYYISQKLNVCPRDVNAHI
VGAHGNKMVLLKRYITVGGIPLQEFINNKLISDAELEAIFDRTVNTALEIVNLHASPYVAPA
AAIIEMAESYLKDLKKVLICSTLLEGQYGHSDIFGGTPVVLGANGVEQVIELQLNSEEKAK
FDEAIAETKRMKALAHHHHHH
>non-antioxidant_436
ATKIDKEACRAAYNLVRDDGSAVIWVTFKYDGSTIVPGEQGAEYQHFIQQCTDDVRLFAF
VRFTTGDAMSKRSKFALITWIGENVSGLQRAKTGTDKTLVKEVVQNFAKEFVISDRKELE
EDFIKSELKKAGGANYDAQTE
>non-antioxidant_437
MQNVGFIGWRGMVGSVLMQRMVEERDFDAIRPVFFSTSQLGQAAPSFGGTTGTLQDAFD
LEALKALDIIVTCQGGDYTNEIYPKLRESGWQGYWIDAASSLRMKDDAIIILDPVNQDVIT
DGLNNGIRTFVGGNCTVSLMLMSLGGLFANDLVDWVSVATYQAASGGGARHMRELLTQ
MGHLYGHVADELATPSSAILDIERKVTTLTRSGELPVDNFGVPLAGSLIPWIDKQLDNGQS
REEWKGQAETNKILNTSSVIPVDGLCVRVGALRCHSQAFTIKLKKDVSIPTVEELLAAHNP
WAKVVPNDREITMRELTPAAVTGTLTTPVGRLRKLNMGPEFLSAFTVGDQLLWGAAEPLR
RMLRQLA
>non-antioxidant_438
SVDHGFLVTRHSQTTDDPQCPPGTKILYHGYSLLYVQGNERAHGQDLGTAGSCLRKFSTM
PFLFCNINNVCNFASRNDYSYWLSTPEPMPMSMAPITGENIRPFISRCAVCEAPAMVMAVH
SQTIQIPQCPTGWSSLWIGYSFVMHTSAGAEGSGQALASPGSCLEEFRSAPFIECHGRGTCN
YYANAYSFWLATIERSEMFKKPTPSTLKAGELRTHVSRCQVCMRRT
>non-antioxidant_439
GSHMSLDNKPIMRVASIDIGSYSVRLTIAQIKDGKLSIILERGRITSLGTKVKETGRLQEDRIE
ETIQVLKEYKKLIDEFKVERVKAVATEAIRRAKNAEEFLERVKREVGLVVEVITPEQEGRYA
YLAVAYSLKPEGEVCVVDQGGGSTEYVFGKGYKVREVISLPIGIVNLTETFFKQDPPTEEE
VKRFFEFLEKELSKVKKPVDTIVGLGGTITTLAALEYNVYPYDPQKVHGKVLTYGQIKKW
FDTFKEIPSEERSKRFRQVEDRRAKVILAGIGIFLKTLEIFEKDCLIVSDWGLREGVLVSEVL
KENHS
>non-antioxidant_440
TLYEALKENEKLHKEIEQKDNEIARLKKENKELAEVA
>non-antioxidant_441
GSPGISGGGGGSHIEGYECQPIFLNVLEAIEPGVVCAGHDNNQPDSFAALLSSLNELGERQL
VHVVKWAKALPGFRNLHVDDQMAVIQYSWMGLMVFAMGWRSFTNVNSRMLYFAPDLV
FNEYRMHKSRMYSQCVRMRHLSQEFGWLQITPQEFLCMKALLLFSIIPVDGLKNQKFFDE
LRMNYIKELDRIIACKRKNPTSCSRRFYQLTKLLDSVQPIARELHQFTFDLLIKSHMVSVDF
PEMMAEIISVQVPKILSGKVKPIYFHTQ
>non-antioxidant_442
STIEERVKKIIGEQLGVKQEEVTNNASFVEDLGADSLDTVELVMALEEEFDTEIPDEEAEKI

TTVQAAIDYINGHQA
>non-antioxidant_443
MGNKEKADRQKVVSDLVALEGALDMYKLDNSRYPTTEQGLQALVSAPSAEPHARNYPE
GGYIRRLPQDPWGSDYQLLSPGQHGQVDIFSLGPDGVPESNDDIGNWTIGFHHHHHHK
>non-antioxidant_444
MNTKYNKEFLLYLAGFVDGNGSIIAQIKPNQSYKFKHQLSLTFQVTQKTQRRWFLDKLVD
EIGVGYVRDRGSVSDYILSEIKPLHNFLTQLQPFLKLKQKQANLVLKIIEQLPSAKESPDKF
LEVCTWVDQIAALNDSKTRKTTSETVRAVLDSLSEKKKSSP
>non-antioxidant_445
MGSSHHHHHHSSGLVPRGSHMEEETRELQSLAAAVVPSAQTLKITDFSFSDFELSDLETAL
CTIRMFTDLNLVQNFQMKHEVLCRWILSVKKNYRKNVAYHNWRHAFNTAQCMFAALKA
GKIQNKLTDLEILALLIAALSHDLDHPGVSNQFLINTNSELALMYNDESVLEHHHFDQCLM
ILNSPGNQILSGLSIEEYKTTLKIIKQAILATDLALYIKRRGEFFELIRKNQFNLEDPHQKELF
LAMLMTACDLSAITKPWPIQQRIAELVATEFFDQGDRERKELNIEPTDLMNREKKNKIPSM
QVGFIDAICLQLYEALTHVSEDCFPLLDGCRKNRQKWQALAELSEKKKSSP
>non-antioxidant_446
LPSGSDPAFSQPKSVLDAGLTCQGASPSSVSKPILLVPGTGTTGPQSFDSNWIPLSTQLGYTP
CWISPPPFMLNDTQVNTEYMVNAITALYAGSGNNKLPVLTWSQGGLVAQWGLTFFPSIRS
KVDRLMAFAPDYKGTVLAGPLDALAVSAPSVWQQTTGSALTTALRNAGGLTQIVPTTNLY
SATDEIVQPQVSNSPLDSSYLFNGKNVQAQAVCGPLFVIDHAGSLTSQFSYVVGRSALRST
TGQARSADYGITDCNPLPANDLTPEQKVAAAALLAPAAAAIVAGPKQNCEPDLMPYARPF
AVGKRTCSGIVTP
>non-antioxidant_447
SGGGGGILEKLGDICFSLRYVPTAGKLTVVILEAKNLKKMDVGGLSDPYVKIHLMQNGKR
LKKKKTTIKKNTLNPYYNESFSFEVPFEQIQKVQVVVTVLDYDKIGKNDAIGKVFVGYNS
TGAELRHWSDMLANPRRPIAQWHTLQVEEEVDAMLAVKK
>non-antioxidant_448
MTCAYRREIHHAHVAIRDWLAGDSRADALDALMARFAEDFSMVTPHGVVLDKTALGELF
RSKGGTRPGLRIEIDGESLLASGVDGATLAYREIQSDAAGRSERLSTVVLHRDDEGRLYWR
HLQETFCG
>non-antioxidant_449
GSHMEYLGVFVDETKEYLQNLNDTLLELEKNPEDMELINEAFRALHTLKGMAGTMGFSS
MAKLCHTLENILDKARNSEIKITSDLLDKIFAGVDMITRMVDKIVS
>non-antioxidant_450
SHPALTQLRALRYSKEIPALDPQLLDWLLLEDSMTKRFEQQGKTVSVTMIREGFVEQNEIP
EELPLLPKESRYWLREILLSADGEPWLAGRTVVPVSTLSGPELALQKLGKTPLGRYLFTSS
TLTRDFIEIGRDAGLWGRRSRLRLSGKPLLLTELFLPASPLY
>non-antioxidant_451
GHMNAADRVMQSYGRCCASTGFFDDFYRHFLASSPQIRAKFATTDMTAQKHLLRAGIMN
LVMYARGMSDSKLRALGASHSRAALDIRPELYDLWLDALLMAVAEHDRDCDAETRDAW
RDVMGRGIAVIKSYYGS
>non-antioxidant_452
AMKPRIYVKVKPERLGAVIGPRGEVKAEIMRRTGTVITVDTENSMVIVEPEAEGIPPVNLM
KAAEVVKAISLGFPPEKAFRLLEEDQILVVVDLKQVVGDSQNHLKRIKGRIIGEGGRARRT

IEEMTDTYINVGEYEVAIIGDYERAMAAKQAIEMLAEGRMHSTVYRHLERIMREIKRRER
LKMWAREEL
>non-antioxidant_453
ACQASQLAVCASAILSGAKPSGECCGNLRAQQGCFCQYAKDPTYGQYIRSPHARDTLTSC
GLAVPHC
>non-antioxidant_454
ATPWQKITQPVPGSAQSIGSFSNGCIVGADTLPIQSEHYQVMRTDQRRYFGHPDLVMFIQR
LSSQVSNLGMGTVLIGDMGMPAGGRFNGGHASHQTGLDVDIFLQLPKTRWTSAQLLRPQ
ALDLVSRDGKHVVSTLWKPEIFSLIKLAAQDKDVTRIFVNPAIKQQLCLDAGTDRDWLRK
VRPWFQHRAHMHVRLRCPADSLECEDQPLPPSGDGCGAELQSWFEPPKPGTTKPEKKTPP
PLPPSCQALLDEHVI

>non-antioxidant_455
ASGPRALSRNQPQYPARAQALRIEGQVKVKFDVTPDGRVDNVQILSAKPANMFEREVKN
AMRRWRYEPGKPGSGIVVNILFKINGTTEIQ
>non-antioxidant_456
GSKAPPTFKVSLMDQSVREGQDVIMSIRVQGEPKPVVSWLRNRQPVRPDQRRFAEEAEGG
LCRLRILAAERGDAGFYTCKAVNEYGARQCEARLEVRGE
>non-antioxidant_457
GHMSARVRPFLMFQGVQAEAAMNFYLSLFDDAEILQIQRYGAEGPGPEGSVLKALFRLG
DQSVHCIDSHVRHAFDFTPAFSFFVDCESNAQIERLAEALSDGGKALMPLGDYGFSQRFA
WLADRFGVSWQLNLAGS
>non-antioxidant_458
SNAMDGQQLNRLLLEWIGAWDPFGLGKDAYDVEAASVLQAVYETEDARTLAARIQSIYE
FAFDEPIPFPHCLKLARRLLELKQAASCPLP
>non-antioxidant_459
MLMTAEQYIESLRKLNTRVYMFGEKIENWVDHPMIRPSINCVRMTYELAQDPQYADLMT
TKSNLIGKTINRFANLHQSTDDLRKKVKMQRLLGQKTASCFQRCVGMDAFNAVFSTTYEI
DQKYGTNYHKNFTEYLKYIQENDLIVDGAMTDPKGDRGLAPSAQKDPDLFLRIVEKRED
GIVVRGAKAHQTGSINSHEHIIMPTIAMTEADKDYAVSFACPSDADGLFMIYGRQSCDTRK
MEEGADIDLGNKQFGGQEALVVFDNVFIPNDRIFLCQEYDFAGMMVERFAGYHRQSYGG
CKVGVGDVVIGAAALAADYNGAQKASHVKDKLIEMTHLNETLYCCGIACSAEGYPTAA
GNYQIDLLLANVCKQNITRFPYEIVRLAEDIAGGLMVTMPSEADFKSETVVGRDGETIGDF
CNKFFAAAPTCTTEERMRVLRFLENICLGASAVGYRTESMHGAGSPQAQRIMIARQGNIN
AKKELAKAIAGIK
>non-antioxidant_460
FENGLGRTPQMGWNSWNHFYCGINEQIIRETADALVNTGLAKLGYQYVNIDDCWAEYSR
DSQGNFVPNRQTFPSGIKALADYVHAKGLKLGIYSDAGSQTCSNKMPGSLDHEEQDVKTF
ASWGVDYLKYDNCNDAGRSVMERYTRMSNAMKTYGKNIFFSLCEWGKENPATWAGRM
GNSWRTTGDIADNWGSMTSRADENDQWAAYAGPGGWNDPDMLEVGNGGMSEAEYRS
HFSIWALAKAPLLIGCDVRSMSQQTKNILSNSEVIAVNQDSLGVQGKKVQSDNGLEVWAG
PLSNNRKAVVLWNRQSYQATITAHWSNIGLAGSVAVTARDLWAHSSFAAQGQISASVAPH
DCKMYVLTPN
>non-antioxidant_461

FDSFWFVQQWPPAVCSFQKSGSCPGSGLRTFTIHGLWPQQSGTSLTNCPGSPFDITKISHLQ
SQLNTLWPNVLRANNQQFWSHEWTKHGTCSESTFNQAAYFKLAVDMRNNYDIIGALRPH
AAGPNGRTKSRQAIKGFLKAKFGKFPGLRCRTDPQTKVSYLVQVVACFAQDGSTLIDCTR
DTCGANFIF
>non-antioxidant_462
MEEAKQKVVDFLNSKSGSKSKFYFNDFTDLFPDMKQREVKKILTALVNDEVLEYWSSGS
TTMYGLKGAGKQAAAEHED
>non-antioxidant_463
NKASVVANQLIPINTALTLIMMKAEVVTPMGIPAEEIPKLVGMQVNRAVPLGTTLMPDMV
KNYE
>non-antioxidant_464
MVRGIRGAITVEEDTPEAIHQATRELLLKMLEANGIQSYEELAAVIFTVTEDLTSAFPAEAA
RQIGMHRVPLLSAREVPVPGSLPRVIRVLALWNTDTPQDRVRHVYLREAVRLRPDLESAQ
>non-antioxidant_465
MTENAEKFLWGVATSAYQIEGATQEDGRGPSIWDAFAQRPGAIRDGSTGEPACDHYRRYE
EDIALMQSLGVRAYRFSVAWPRILPEGRGRINPKGLAFYDRLVDRLLASGITPFLTLYHWD
LPLALEERGGWRSRETAFAFAEYAEAVARALADRVPFFATLNEPWCSAFLGHWTGEHAPG
LRNLEAALRAAHHLLLGHGLAVEALRAAGARRVGIVLNFAPAYGEDPEAVDVADRYHNR
FFLDPILGKGYPESPFRDPPPVPILSRDLELVARPLDFLGVNYYAPVRVAPGTGTLPVRYLPP
EGPATAMGWEVYPEGLYHLLKRLGREVPWPLYVTENGAAYPDLWTGEAVVEDPERVAYL
EAHVEAALRAREEGVDLRGYFVWSLMDNFEWAFGYTRRFGLYYVDFPSQRRIPKRSALW
YRERIARAQT
>non-antioxidant_466
MTNLSDIIEKETGKQLVIQESILMLPEEVEEVIGNKPESDILVHTAYDESTDENVMLLTSDAP
EYKPWALVIQDSNGENKIKML
>non-antioxidant_467
GKAFDDGAFTGIREINLSYNKETAIGDFQVVYDLNGSPYVGQNHVSFITGFTPVKISLDFPS
EYIMEVSGYTGNVSGYVVVRSLTFKTNKKTYGPYGVTSGTPFNLPIENGLIVGFKGSIGY
WLDYFSMYLSL
>non-antioxidant_468
MTLELLQAQAQNCTACRLMEGRTRVVFGEGNPDAKLMIVGEGPGEEEDKTGRPFVGKA
GQLLNRILEAAGIPREEVYITNIVKCRPPQNRAPLPDEAKICTDKWLLKQIELIAPQIIVPLG
AVAAEFFLGEKVSITKVRGKWYEWHGIKVFPMFHPAYLLRNPSRAPGSPKHLTWLDIQEV
KRALDALPPKERRPVKAVSQEPLF
>non-antioxidant_469
MTTLEAFAKARSEGRAALIPYLTAGFPSREGFLQAVEEVLPYADLLEIGLPYSDPLGDGPVI
QRASELALRKGMSVQGALELVREVRALTEKPLFLMTYLNPVLAWGPERFFGLFKQAGAT
GVILPDLPPDEDPGLVRLAQEIGLETVFLLAPTSTDARIATVVRHATGFVYAVSVTGVTGM
RERLPEEVKDLVRRIKARTALPVAVGFGVSGKATAAQAAVADGVVVGSALVRALEEGRSL
APLLQEIRQGLQRLEANPGLKESSKKPLP
>non-antioxidant_470
MANLEIGKSILAAGVLTNYHDVGEGQPVILIHGSGPGVSAYANWRLTIPALSKFYRVIAPD
MVGFGFTDRPENYNYSKDSWVDHIIGIMDALEIEKAHIVGNAFGGGLAIATALRYSERVD
RMVLMGAAGTRFDVTEGLNAVWGYTPSIENMRNLLDIFAYDRSLVTDELARLRYEASIQP

GFQESFSSMFPEPRQRWIDALASSDEDIKTLPNETLIIHGREDQVVPLSSSLRLGELIDRAQL
HVFGRCGHWTQIEQTDRFNRLVVEFFNEANTPKLVGRP
>non-antioxidant_471
SLSDFSVASRDVNHNNICAGLSTEWLVMSSDGDAESRMDHLDYNGEGQSRGSERHQVYN
DALRAALSNDDEAPFFTASTAVIEDAGFSLRREPKTVHASGGSAQLGQTVAHDVAQSGRK
HLLSLRFANVQGHAIACSCEGSQFKLFDPNLGEFQSSRSAAPQLIKGLIDHYNSLNYDVAC
VNEFRVSV
>non-antioxidant_472
MSMSDVAIVKEGWLHKRGEYIKTWRPRYFLLKNDGTFIGYKERPQDVDQREAPLNNFSV
AQCQLMKTERPRPNTFIIRCLQWTTVIERTFHVETPEEREEWTTAIQTVADGLKKQEEEEM
DFRSG
>non-antioxidant_473
DTCGSGYNVDQRRTNSGCKAGNGDRHFCGCDRTGVVECKGGKWTEVQDCGSSSCKGTS
NGGATC
>non-antioxidant_474
MASRILLNNGAKMPILGLGTWKSPPGQVTEAVKVAIDVGYRHIDCAHVYQNENEVGVAI
QEKLREQVVKREELFIVSKLWCTYHEKGLVKGACQKTLSDLKLDYLDLYLIHWPTGFKPG
KEFFPLDESGNVVPSDTNILDTWAAMEELVDEGLVKAIGISNFNHLQVEMILNKPGLKYKP
AVNQIECHPYLTQEKLIQYCQSKGIVVTAYSPLGSPDRPWAKPEDPSLLEDPRIKAIAAKHN
KTTAQVLIRFPMQRNLVVIPKSVTPERIAENFKVFDFELSSQDMTTLLSYNRNWRVCALLS
CTSHKDYPFHEEF
>non-antioxidant_475
MRKPILAALTLAGLGLAQEFITIGSGSTTGVYFPVATGIAKLVNDANVGIRANARSTGGSVA
NINAINAGEFEMALAQNDIAYYAYQGCCIPAFEGKPVKTIRALAALYPEVVHVVARKDAGI
RTVADLKGKRVVVGDVGSGTEQNARQILEAYGLTFDDLGQAIRVSASQGIQLMQDKRAD
ALFYTVGLGASAIQQLALTTPIALVAVDLNRIQAIAKKYPFYVGFNIPGGTYKGVDVTTPTV
AVQAMLIASERLSEETVYKFMKAVFGNLEAFKKIHPNLERFFGLEKAVKGLPIPLHPGAER
FYKEAGVLK
>non-antioxidant_476
PSSSDYSDLQRVKQELLEEVKKELQKVKEEIIEAFVQELRKRGSP
>non-antioxidant_477
MDWEERENLKRLVKTFAFPNFREALDFANRVGALAERENHHPRLTVEWGRVTVEWWTH
SAGGVTEKDREMARLTDALLQR
>non-antioxidant_478
TAPTPILRFVAVGDWGGVPNAPFHTAREMANAKAIATTVKTLGADFILSLGDNFYFTGVH
DAKDKRFQETFEDVFSDPSLRNVPWHVLAGNHDHLGNVSAQIAYSKISKRWNFPSPYYRL
RFKIPRSNVSVAIFMLDTVTLCGNSDDFVSQQPERPRNLALARTQLAWIKKQLAAAKEDY
VLVAGHYPVWSIAEHGPTHCLVKQLLPLLTTHKVTAYLCGHDHNLQYLQDENGLGFVLS
GAGNFMDPSKKHLRKVPNGYLRFHFGAENSLGGFAYVEITPKEMSVTYIEASGKSLFKTK
LPRRARSEHQHRRA
>non-antioxidant_479
GICPRFAHVIENLLLGTPSSYETSLKEFEPDDTMKDAGMQMKKVLDSLPQTTRENIMKLTE
KIVKSPLCM
>non-antioxidant_480

VPGPEYKVAILTVSDTVSAGAGPDRSGPRAVSVVDSSSEKLGGAKVVATAVVPDEVERIKD
ILQKWSDVDEMDLILTLGGTGFTPRDVTPEATKKVIERETPGLLFVMMQESLKITPFAMLA
RSAAGIRGSTLIINMPGNPNAVAECMEALLPALKHALKQIKGDKR
>non-antioxidant_481
ASTQGISEDLYNRLVEMATISQAAYADLCNIPSTIIKGEKIYNAQTDINGWILRDDTSKEIITV
FRGTGSDTNLQLDTNYTLTPFDTLPQCNDCEVHGGYYIGWISVQDQVESLVKQQASQYPD
YALTVTGHSLGASMAALTAAQLSATYDNVRLYTFGEPRSGNQAFASYMNDAFQVSSPETT
QYFRVTHSNDGIPNLPPAEQGYAHGGVEYWSVDPYSAQNTFVCTGDEVQCCEAQGGQGV
NDAHTTYFGMTSGACTWV
>non-antioxidant_482
MTDNNKYRDVEIRAPRGNKLTAKSWLTEAPLRMLMNNLDPQVAENPKELVVYGGIGRAA
RNWECYDKIVETLTRLEDDETLLVQSGKPVGVFKTHSNAPRVLIANSNLVPHWANWEHFN
ELDAKGLAMYGQMTAGSWIYIGSQGIVQGTYETFVEAGRQHYGGSLKGKWVLTAGLGG
MGGAQPLAATLAGACSLNIESQQSRIDFRLETRYVDEQATDLDDALVRIAKYTAEGKAISI
ALHGNAAEILPELVKRGVRPDMVTDQTSAHDPLNGYLPAGWTWEQYRDRAQTEPAAVV
KAAKQSMAVHVQAMLDFQKQGVPTFDYGNNIRQMAKEEGVADAFDFPGFVPAYIRPLFC
RGVGPFRWAALSGEAEDIYKTDAKVKELIPDDAHLHRWLDMARERISFQGLPARICWVGL
GLRAKLGLAFNEMVRSGELSAPVVIGRDHLDSGSVSSPNAETEAMRDGSDAVSDWPLLN
ALLNTAGGATWVSLHHGGGVGMGFSQHSGMVIVCDGTDEAAERIARVLTNDPGTGVMR
HADAGYDIAIDCAKEQGLDLPMITG
>non-antioxidant_483
MPEETQTQDQPMEEEEVETFAFQAEIAQLMSLIINTFYSNKEIFLRELISNSSDALDKIRYES
LTDPSKLDSGKELHINLIPNKQDRTLTIVDTGIGMTKADLINNLGTIAKSGTKAFMEALQA
GADISMIGQFGVGFYSAYLVAEKVTVITKHNDDEQYAWESSAGGSFTVRTDTGEPMGRGT
KVILHLKEDQTEYLEERRIKEIVKKHSQFIGYPITLFVEKERDKEVSDDEAE
>non-antioxidant_484
GSMPVVWPTLLDLSRDECKRILRKLELEAYAGVISALRAQGDLTKEKKDLLGELSKVLSIS
TERHRAEVRRAVNDERLTTIAHNMSGPNSSSEWSIEGRRLV
>non-antioxidant_485
AMGSDASKVVTRGPGLSQAFVGQKNSFTVDCSKAGTNMMMVGVHGPKTPCEEVYVKH
MGNRVYNVTYTVKEKGDYILIVKWGDESVPGSPFKVKVP
>non-antioxidant_486
ADSATPHLDAVEQTLRQVSPGLEGDVWERTSGNKLDGSAADPSDWLLQTPGCWGDDKC
ADRVGTKRLLAKMTENIGNATRTVDISTLAPFPNGAFQDAIVAGLKESAAKGNKLKVRILV
GAAPVYHMNVIPSKYRDELTAKLGKAAENITLNVASMTTSKTAFSWNHSKILVVDGQSAL
TGGINSWKDDYLDTTHPVSDVDLALTGPAAGSAGRYLDTLWTWTCQNKSNIASVWFAAS
GNAGCMPTMHKDTNPKASPATGNVPVIAVGGLGVGIKDVDPKSTFRPDLPTASDTKCVV
GLHDNTNADRDYDTVNPEESALRALVASAKGHIEISQQDLNATCPPLPRYDIRLYDALAAK
MAAGVKVRIVVSDPANRGAVGSGGYSQIKSLSEISDTLRNRLANITGGQQAAKTAMCSNL
QLATFRSSPNGKWADGHPYAQHHKLVSVDSSTFYIGSKNLYPSWLQDFGYIVESPEAAKQ
LDAKLLDPQWKYSQETATVDYARGICNA
>non-antioxidant_487
GKPKTDTDFQTYNGDGFKLQIPSKWNPNKEVEYPGQVLRFEDNFDATSNVIVAITPTDKK
SITDFGSPEQFLSQVDYLLGRQAYSGKTDSEGGFESDAVAIANVLETSTAEVGGKQYYYLS

ILTRTADGNEGGKHQLVTATVNDGKLYICKAQAGDKRWFKGAKKFVENTATSFSLA
>non-antioxidant_488
MRERTEARRRRIEEVLRRRQPDLTVLLENVHKPHNLSAILRTCDAVGVLEAHAVNPTGGV
PTFNETSGGSHKWVYLRVHPDLHEAFRFLKERGFTVYATALREDARDFREVDYTKPTAVL
FGAEKWGVSEEALALADGAIKIPMLGMVQSLNVSVAAAVILFEAQRQRLKAGLYDRPRL
DPELYQKVLADWLRK
>non-antioxidant_489
MYSIEVRTHSALHVVKGAVVKVLGSEAKWTYSTYVKGNKGVLIVKFDRKPSDEEIREIER
LANEKVKENAPIKIYELPREEAEKMFGEDMYDLFPVPEDVRILKVVVIEDWNVNACNKE
HTKTTGEIGPIKIRKVRFRKSKGLLEIHFELLELENPS
>non-antioxidant_490
AQSVPYGVSQIKAPALHSQGYTGSNVKVAVIDSGIDSSHPDLKVAGGASMVPSETNPFQDN
NSHGTHVAGTVAALNNSIGVLGVAPSASLYAVKVLGADGSGQYSWIINGIEWAIANNMDVI
NMSLGGPSGSAALKAAVDKAVASGVVVVAAAGNEGTSGSSSTVGYPGKYPSVIAVGAVD
SSNQRASFSSVGPELDVMAPGVSIQSTLPGNKYGAYNGTCMASPHVAGAAALILSKHPNW
TNTQVRSSLENTTTKLGDSFYYGKGLINVQAAAQ
>non-antioxidant_491
MIQMVKRVHIFDWHKEHARKIEEFAGWEMPIWYSSIKEEHLAVRNAVGIFDVSHMGEIVF
RGKDALKFLQYVTTNDISKPPAISGTYTLVLNERGAIKDETLVFNMGNNEYLMICDSDAFE
KLYAWFTYLKRTIEQFTKLDLEIELKTYDIAMFAVQGPKARDLAKDLFGIDINEMWWFQA
RWVELDGIKMLLSRSGYTGENGFEVYIEDANPYHPDESKRGEPEKALHVWERILEEGKK
YGIKPCGLGARDTLRLEAGYTLYGNETKELQLLSTDIDEVTPLQANLEFAIYWDKDFIGKD
ALLKQKERGVGRKLVHFKMIDKGIPREGYKVYANGEMIGEVTSGTLSPLLNVGIGIAFVK
EEYAKPGIEIEVEIRGQRKKAVTVTPPFYDPKKYGLFRET
>non-antioxidant_492
LECHNQQSSQTPTTTGCSGGETNCYKKRWRDHRGYRTERGCGCPSVKNGIEINCCTTDRC
NN
>non-antioxidant_493
MKYGYFDNDNREYVITRPDVPAPWTNYLGTEKFCTVISHNAGGYSFYNSPEYNRVTKFRP
NATFDRPGHYVYLRDDDSGDYWSISWQPVAKSLDEAQYQIRHGLSYSKFQCDYNGIHAR
KTLFVPKGEDAEIWDVVIKNTSDQVRTISAFSFVEFSFSHIQSDNQNHQMSLYSAGTAYRP
GLIEYDLYYNTDDFEGFYYLASTFDPDSYDGQRDRFLGLYRDEANPLAVEQGRCSNSAQT
CYNHCGSLHKQFTLQPGEEIRFAYILGIGKGNGERLREHYQDVANIDAAFAAIKAHWDER
CAKFQVKSPNQGLDTMINAWTLYQAETCVVWSRFASFIEVGGRTGLGYRDTAQDAISVPH
ANPEMTRKRIVDLLRGQVKAGYGLHLFDPDWFDPEKEDVAPSKSPTVVPTPSDEDKIHGI
KDTCSDDHLWLIPTICKYVMETGETSFFDQMIPYADGGEASVYEHMKAALDFSAEYVGQ
TGICKGLRADWNDCLNLGGGESSMVSFLHFWALQEFIDLAKFLGKDQDVNTYTEMAAN
VREACETHLWDDEGGWYIRGLTKNGDKIGTAQQQEGRVHLESNTLAVLSGLASQERGEQ
AMDAVDEHLFSPYGLHLNAPSFSTPNDDIGFVTRVYQGVKENGAIFSHPNPWAWVAETKL
GRGDRAMKFYDALNPYNQNDIIEKRIAEPYSYVQFIMGRDHQDHGRANHPWLTGTSGWA
YFAVTNYILGVQSGFTGLSVDPCIPSDWPGFEVTRQWRGATYHIQVENPDHVSKGVKSITL
NGAPIQGRIPPQAQGSDNQVVVVLGHHHHHH
>non-antioxidant_494
PFRTIARLNPAKPKAGEEFRLQVVAQHPNEPGTRRDAEGKLIPAKYINLVEVYFEGEKVAE

ARPGPSTSANPLYAFKFKAEKAGTFTIKLKDTDGDTGEASVKLELV
>non-antioxidant_495
SRCQGKSSWPQLVGSTGAAAKAVIERENPRVRAVIIKVGSGATKDFRCDRVRVWVTERGIV
ARPPTIG
>non-antioxidant_496
MRALFYKDGKLFTDNNFLNPVSDDNPAYEVLQHVKIPTHLTDVVVYEQTWEEALTRLIFV
GSDSKGRRQYFYGKMHV
>non-antioxidant_497
MTAFRQRPLRLGHRGAPLKAKENTLESFRLALEAGLDGVELDVWPTRDGVFAVRHDPDT
PLGPVFQVDYADLKAQEPDLPRLEEVLALKEAFPQAVFNVELKSFPGLGEEAARRLAALL
RGREGVWVSSFDPLALLALRKAAPGLPLGFLMAEDHSALLPCLGVEAVHPHHALVTEEAV
AGWRKRGLFVVAWTVNEEGEARRLLALGLDGLIGDRPEVLLPLGG
>non-antioxidant_498
MRRFALTVALPKGRMFREAYEVLKRAGLDLPEVEGERTLLHGKEGGVALLELRNKDVPIY
VDLGIAEIGVVGKDVLLDSGRDLFEPVDLGFGACRLSLIRRPGDTGPIRRVATKYPNFTARL
LKERGWAADVVELSGNIELAAVTGLADAVVDVVQTGATLRAAGLVEVEVLAHSTARLVV
NRQALKLKRAVLKPLIQRLRELSGS
>non-antioxidant_499
METRTLEDWRALLEAEKTLDSGVYNKHDLLIVRGQGARVWDAEGNEYIDCVGGYGVAN
LGHGNPEVVEAVKRQAETLMAMPQTLPTPMRGEFYRTLTAILPPELNRVFPVNSGTEANE
AALKFARAHTGRKKFVAAMRGFSGRTMGSLSVTWEPKYREPFLPLVEPVEFIPYNDVEAL
KRAVDEETAAVILEPVQGEGGVRPATPEFLRAAREITQEKGALLILDEIQTGMGRTGKRFAF
EHFGIVPDILTLAKALGGGVPLGVAVMREEVARSMPKGGHGTTFGGNPLAMAAGVAAIRY
LERTRLWERAAELGPWFMEKLRAIPSPKIREVRGMGLMVGLELKEKAAPYIARLEKEHRV
LALQAGPTVIRFLPPLVIEKEDLERVVEAVRAVLA
>non-antioxidant_500
DSKTPADWIDSDACMICSKKFSLLNRKHHCRSCGGVFCQEHSSNSIPLPDLGIYEPVRVCD
SCFEDYEFIVTD
>non-antioxidant_501
MSLIWKRKITLEALNAMGEGNMVGFLDIRFEHIGDDTLEATMPVDSRTKQPFGLLHGGAS
VVLAESIGSVAGYLCTEGEQKVVGLEINANHVRSAREGRVRGVCKPLHLGSRHQVWQIEI
FDEKGRLCCSSRLTTAILEGGSHHHHHH
>non-antioxidant_502
MAHHHHHHMSLAVEAVKDFLLKLQDDICEALEAEDGQATFVEDKWTREGGGGGRTRVM
VDGAVIEKGGVNFSHVYGKGLPMSSTERHPDIAGCNFEAMGVSLVIHPKNPHVPTSHANV
RLFVAEREGKEPVWWFGGGFDLTPYYAVEEDCRDFHQVAQDLCKPFGADVYARFKGWC
DEYFFIPYRNEARGIGGLFFDDLNEWPFEKCFEFVQAVGKGYMDAYIPIVNRRKNTPYTEQ
QVEFQEFRRGRYAEFNLVIDRGTKFGLQSGGRTESILISLPPRARWGYNWQPEPGTPEARLT
EYFLTKRQWV
>non-antioxidant_503
MGVEKVPKYDIPVKKVEYVFIELDKMKPHEQLVQRELEDFIESVTGSGIFWKPMLLAKIP
GTDEYLIVDGHHRWAGLQKLGAKRAPSVILDYFDEGVKVYTWYPAFKGDVNKVIERLKA
EGLEVIEDEKAEEKAEKGEIAFALIGEKSFAIPGGLEEQKKVSKVLDEMDQAKEIELVYYG
LKEDAKADMEKGEIDYVFIRKAPTKEEVMELVKRGEVFSPKTTRHVLPFIPDKIDVKLEDL

F
>non-antioxidant_504
MGSDKIHHHHHHMEYKKFVEARRELNEKVLSRGTLNTKRFFNLDSAVYRPGKLDVKTKE
LMGLVASTVLRCDDCIRYHLVRCVQEGASDEEIFEALDIALVVGGSIVIPHLRRAVGFLEEL
REMEKNGETISL
>non-antioxidant_505
MGSDKIHHHHHHMSDSLVVCEVDPELKETLRKFRFRKETNNAAIIMKVDKDRQMVVLED
ELQNISPEELKLELPERQPRFVVYSYKYVHDDGRVSYPLCFIFSSPVGCKPEQQMMYAGSK
NRLVQTAELTKVFEIRTTDDLTETWLKEKLAFFR
>non-antioxidant_506
MGSDKIHHHHHHMSQLEKAQAEYAGFIQEFQSAIISTISEQGIPNGSYAPFVIDDAKNIYIY
VSGLAVHTKNIEANPLVNVLFVDDEAKTNQIFARRRLSFDCTATLIERESQKWNQVVDQF
QERFGQIIEVLRGLADFRIFQLTPKEGRFVIGFGA
>non-antioxidant_507
MGSDKIHHHHHHMAFTPFPPRQPTASARLPLTLMTLDDWALATITGADSEKYMQGQVTA
DVSQMAEDQHLLAAHCDAKGKMWSNLRLFRDGDGFAWIERRSVREPQLTELKKYAVFS
KVTIAPDDERVLLGVAGFQARAALANLFSELPSKEKQVVKEGATTLLWFEHPAERFLIVTD
EATANMLTDKLRGEAELNNSQQWLALNIEAGFPVIDAANSGQFIPQATNLQALGGISFKKG
CYTGQEMVARAKFRGANKRALWLLAGSASRLPEAGEDLELKMGENWRRTGTVLAAVKL
EDGQVVVQVVMNNDMEPDSIFRVRDDANTLHIEPLPYSLEE
>non-antioxidant_508
MGSDKIHHHHHHMDLELKELQSKMKEMYFEKDSQRGIYATFTWLVEEVGELAEALLSNN
LDSIQEELADVIAWTVSIANLEGIDIEEALKKKYKL
>non-antioxidant_509
MGSDKIHHHHHHMKGVIEYSLKTSNDDQFIDITNLVKKAVDESGVSDGMAVVFCPHTTA
GITINENADPDVTRDILVNLDKVFPKVGDYKHVEGNSHAHIKASLMGSSQQIIIENGKLKL
GTWQGIYFTEFDGPRDRKVFVKII
>non-antioxidant_510
MGSDKIHHHHHHMEKKIVYFNKPGRENTEETLRLAVERAKELGIKHLVVASSYGDTAMK
ALEMAEGLEVVVVTYHTGFVREGENTMPPEVEEELRKRGAKIVRQSHILSGLERSISRKL
GGVSRTEAIAEALRSLFGHGLKVCVEITIMAADSGAIPIEEVVAVGGRSRGADTAVVIRPAH
MNNFFDAEIKEIICMPRNKR
>non-antioxidant_511
MGSDKIHHHHHHMFYEIRTYRLKNGAIPAYLKVVEDEGIEIQKSHLGELVGYFFSEIGPINE
IVHIWAFSSLDDRAERRARLMADPRWLSFLPKIRDLIEVAENKIMKPARFSPLM
>non-antioxidant_512
MGSDKIHHHHHHMKSLGRHLVAEFYECDREVLDNVQLIEQEMKQAAYESGATIVTSTFH
RFLPYGVSGVVVISESHLTIHTWPEYGYAAIDLFTCGEDVDPWKAFEHLKKALKAKRVHV
VEHERGRYDEIGIPEDSPHKAAV
>non-antioxidant_513
MGSDKIHHHHHHRTKDQYYELRDFALGTSVRIVVSSQKINPRTIAEAILEDMKRITYKFSF
TDERSVVKKINDHPNEWVEVDEETYSLIKAACAFAELTDGAFDPTVGRLLELWGFTGNYE
NLRVPSREEIEEALKHTGYKNVLFDDKNMRVMVKNGVKIDLGGIAKGYALDRARQIALSF
DENATGFVEAGGDVRIIGPKFGKYPWVIGVKDPRGDDVIDYIYLKSGAVATSGDYERYFV

VDGVRYHHILDPSTGYPARGVWSVTIIAEDATTADALSTAGFVMAGKDWRKVVLDFPNM
GAHLLIVLEGGAIERSETFKLFERE
>non-antioxidant_514
WSATNEEDDLSVEAEIAHQIAESFSKKYKFPSRSSGIFLYNFEQLKMNLDDIVKEAKNVPG
VTRLAHDGSKIPLRCVLGWVALANSKKFQLLVEADKLSKIMQDDLNRYTSC
>non-antioxidant_515
EARPIVVGPPPPLSGGLPGTENSDQARDGTLPYTKDRFYLQPLPPTEAAQRAKVSASEILN
VKQFIDRKAWPSLQNDLRLRASYLRYDLKTVISAKPKDEKKSLQELTSKLFSSIDNLDHAA
KIKSPTEAEKYYGQTVSNINEVLAKLG
>non-antioxidant_516
SAEKLFTPLKVGAVTAPNRVFMAPLTRLRSIEPGDIPTPLMGEYYRQRASAGLIISEATQISA
QAKGYAGAPGLHSPEQIAAWKKITAGVHAEDGRIAVQLWHTGRISHSSIQPGGQAPVSAS
ALNANTRTSLRDENGNAIRVDTTTPRALELDEIPGIVNDFRQAVANAREAGFDLVELHSAH
GYLLHQFLSPSSNQRTDQYGGSVENRARLVLEVVDAVCNEWSADRIGIRVSPIGTFQNVD
NGPNEEADALYLIEELAKRGIAYLHMSETDLAGGKPYSEAFRQKVRERFHGVIIGAGAYTA
EKAEDLIGKGLIDAVAFGRDYIANPDLVARLQKKAELNPQRPESFYGGGAEGYTDYPSL
>non-antioxidant_517
MPERLQVYKCEVCGNIVEVLNGGIGELVCCNQDMKLMSENTVDAAKAKHVPVIEKIDGG
YKVKVGAVAHPMEEKHYIQWIELLADDKCYTQFLKPGQAPEAVFLIEAAKVVAREYCNIH
GHWKAEN
>non-antioxidant_518
AAKELTLAQTESLREVCETNMACDEMADAQGIVAAYQAFYGPIPF
>non-antioxidant_519
GSHMADSYYDYICIIDFEATCEEGNPPEFVHEIIEFPVVLLNTHTLEIEDTFQQYVRPEINTQ
LSDFCISLTGITQDQVDRADTFPQVLKKVIDWMKLKELGTKYKYSLLTDGSWDMSKFLNI
QCQLSRLKYPPFAKKWINIRKSYGNFYKVPRSQTKLTIMLEKLGMDYDGRPHCGLDDSK
NIARIAVRMLQDGCELRINEKM
>non-antioxidant_520
WQFTPNTGGNTITKVEAENMKIGGTYAGKISAPFDGVALYANADYVSYSQYFANSTHNIS
VRGASSNAGTAKVDLVIGGVTVGSFNFTGKTPTVQTLSNITHATGDQEIKLALTSDDGTW
DAYVDFIEFSL
>non-antioxidant_521
VKQVFNFNAGPSALPKPALERAQKELLNFNDTQMSVMELSHRSQSYEEVHEQAQNLLRE
LLQIPNDYQILFLQGGASLQFTMLPMNLLTKGTIGNYVLTGSWSEKALKEAKLLGETHIAA
STKANSYQSIPDFSEFQLNENDAYLHITSNNTIYGTQYQNFPEINHAPLIADMSSDILSRPLK
VNQFGMIYAGAQKNLGPSGVTVVIVKKDLLNTKVEQVPTMLQYATHIKSDSLYNTPPTFSI
YMLRNVLDWIKDLGGAEAIAKQNEEKAKIIYDTIDESNGFYVGHAEKGSRSLMNVTFNL
RNEELNQQFLAKAKEQGFVGLNGHRSVGGCRASIYNAVPIDACIALRELMIQFKENA
>non-antioxidant_522
HMSSGSAGLSSLHRTYSQDCSFKNSMYHVGDYVYVEPAEANLQPHIVCIERLWEDSAGE
KWLYGCWFYRPNETFHLATRKFLEKEVFKSDYYNKVPVSKILGKCVVMFVKEYFKLCPE
NFRDEDVYVCESRYSAKTKSFKKIKLWTMPVSSVRFVPRDVPLPVVRVASVFANTD
>non-antioxidant_523
MDFREVIEQRYHQLLSRYIAELTETSLYQAQKFSRKTIEHQIPPEEIISIHRKVLKELYPSLPE

DVFHSLDFLIEVMIGYGMAY
>non-antioxidant_524
MSFTPANRAYPYTRLRRNRRDDFSRRLVRENVLTVDDLILPVFVLDGVNQRESIPSMPGVE
RLSIDQLLIEAEEWVALGIPALALFPVTPVEKKSLDAAEAYNPEGIAQRATRALRERFPELGI
ITDVCLCEFTTHGQCGILDDDGYVLNDVSIDVLVRQALSHAEAGAQVVAPSDMMDGRIG
AIREALESAGHTNVRVMAYSAKYASAYYGPFRDAVGSASNLGKGNRATYQMDPANSDEA
LHEVAADLAEGADMVMVKPGMPYLDIVRRVKDEFRAPTFVYQVSGEYAMHMGAIQNG
WLAESVILESLTAFKRAGADGILTYFAKQAAEQLRRGR
>non-antioxidant_525
ESHMAMDLGGYLTRIGLDGRPRPDLGTLHAIVAAHNRSIPFENLDPLLGIPVADLSAEALF
AKLVDRRRGGYQYEHNGLLGYVLEELGFEVERLSGRVVWMRADDAPLPAQTHNVLSVA
VPGADGRYLVDVGFGGQTLTSPIRLEAGPVQQTRHEPYRLTRHGDDHTLAAQVRGEWQP
LYTFTTEPRPRIDLEVGSWYVSTHPGSHFVTGLTVAVVTDDARYNLRGRNLAVHRSGATEH
IRFDSAAQVLDAIVNRFGIDLGDLAGRDVQARVAEVLDT
>non-antioxidant_526
GAMAGSIRSKLSAIDVRQLGTVDYRTAWQLQRELADARVAGGADTLLLLEHPAVYTAGRR
TETHERPIDGTPVVDTDRGGKITWHGPGQLVGYPIIGLAEPLDVVNYVRRLEESLIQVCAD
LGLHAGRVDGRSGVWLPGRPARKVAAIGVRVSRATTLHGFALNCDCDLAAFTAIVPCGIS
DAAVTSLSAELGRTVTVDEVRATVAAAVCAALDGVLPVGDRVPSHAVPSPL
>non-antioxidant_527
NDKLVELSKSDDNWVMPGKNYDSNNFSDLKQINKGNVKQLRPAWTFSTGLLNGHEGAPL
VVDGKMYIHTSFPNNTFALGLDDPGTILWQDKPKQNPAARAVACCDLVNRGLAYWPGDG
KTPALILKTQLDGNVAALNAETGETVWKVENSDIKVGSTLTIAPYVVKDKVIIGSSGAELG
VRGYLTAYDVKTGEQVWRAYATGPDKDLLLASDFNIKNPHYGQKGLGTGTWEGDAWKI
GGGTNWGWYAYDPGTNLIYFGTGNPAPWNETMRPGDNKWTMTIFGRDADTGEAKFGY
QKTPHDEWDYAGVNVMMLSEQKDKDGKARKLLTHPDRNGIVYTLDRTDGALVSANKLD
DTVNVFKSVDLKTGQPVRDPEYGTRMDHLAKDICPSAMGYHNQGHDSYDPKRELFFMG
INHICMDWEPFMLPYKAGQFFVGATLNMYPGPKGDRQNYEGLGQIKAYNAITGDYKWE
KMERFAVWGGTMATAGDLVFYGTLDGYLKARDSDTGDLLWKFKIPSGAIGYPMTYTHK
GTQYVAIYYGVGGWPGVGLVFDLADPTAGLGAVGAFKKLANYTQMGGGVVVFSLDGK
GPYDDPNVGEWKSAAK
>non-antioxidant_528
YDGTKCKAAGNCWEPKPGFPEKIAGSKYDPKHDPKELNKQADSIKQMEERNKKRVENFK
KTGKFEYDVAKISAN
>non-antioxidant_529
ASAECVSNENVEIEAPKTNIWTSLAKEEVQEVLDLLHSTYNITEVTKADFFSNYVLWIETL
KPNKTEALTYLDEDGDLPPRNARTVVYFGEGEEGYFEELKVGPLPVSDETTIEPLSFYNTN
GKSKLPFEVGHLDRIKSAAKSSFLNKNLNTTIMRDVLEGLIGVPYEDMGCHSAAPQLHDP
ATGATVDYGTCNINTENDAENLVPTGFFFKFDMTGRDVSQWKMLEYIYNNKVYTSAEEL
YEAMQKDDFVTLPKIDVDNLDWTVIQRNDSAPVRHLDDRKSPRLVEPEGRRWAYDGDEE
YFSWMDWGFYTSWSRDTGISFYDITFKGERIVYELSLQELIAEYGSDDPFNQHTFYSDISY
GVGNRFSLVPGYDCPSTAGYFTTDTFEYDEFYNRTLSYCVFENQEDYSLLRHTGASYSAIT
QNPTLNVRFISTIGNYDYNFLYKFFLDGTLEVSVRAAGYIQAGYWNPETSAPYGLKIHDVL
SGSFHDHVLNYKVDLDVGGTKNRASQYVMKDVDVEYPWAPGTVYNTKQIAREVFENED

FNGINWPENGQGILLIESAEETNSFGNPRAYNIMPGGGGVHRIVKNSRSGPETQNWARSNL
FLTKHKDTELRSSTALNTNALYDPPVNFNAFLDDESLDGEDIVAWVNLGLHHLPNSNDLP
NTIFSTAHASFMLTPFNYFDSENSRDTTQQVFYTYDDETEESNWEFYGNDWSSCGVEVAE
PNFEDYTYGRGTRINKKMTNSDEVY
>non-antioxidant_530
MASDKFPVAENPSSSFKYESAVQYRPAPDSYLNPCPQAGRIVKETYTGINGTKSLNVYLPY
GYDPNKKYNIFYLMHGGGENENTIFSNDVKLQNILDHAIMNGELEPLIVVTPTFNGGNCT
AQNFYQEFRQNVIPFVESKYSTYAESTTPQGIAASRMHRGFGGFAMGGLTTWYVMVNCL
DYVAYFMPLSGDYWYGNSPQDKANSIAEAINRSGLSKREYFVFAATGSEDIAYANMNPQI
EAMKALPHFDYTSDFSKGNFYFLVAPGATHWWGYVRHYIYDALPYFFHELEHHHHHH
>non-antioxidant_531
MALLAEHLLRPLPADKQIETGPFLEAVSHLPPFFDCLGSPVFTPIKADISGNITKIKAVYDTN
PTKFRTLQNILEVEKEMYGAEWPKVGATLALMWLKRGLRFIQVFLQSICDGERDENHPNL
IRVNATKAYEMALKKYHGWIVQKIFQAALYAAPYKSDFLKALSKGQNVTEEECLEKVRLF
LVNYTATIDVIYEMYTRMNAELNYKV
>non-antioxidant_532
AMKNWKTSAESILTTGPVVPVIVVKKLEHAVPMAKALVAGGVRVLNVTLRTECAVDAIRA
IAKEVPEAIVGAGTVLNPQQLAEVTEAGAQFAISPGLTEPLLKAATEGTIPLIPGISTVSELM
LGMDYGLKEFKFFPAEANGGVKALQAIAGPFSQVRFCPTGGISPANYRDYLALKSVLCIG
GSWLVPADALEAGDYDRITKLAREAVEGAKL
>non-antioxidant_533
NQKCSGNPRRYNGKSCASTTNYHDSHKGACGCGPASGDAQFGWNAGSFVAAASQMYFD
SGNKGWCGQHCGQCIKLTTTGGYVPGQGGPVREGLSKTFMITNLCPNIYPNQDWCNQGS
QYGGHNKYGYELHLDLENGRSQVTGMGWNNPETTWEVVNCDSEHNHDHRTPSNSMYG
QCQCAHQ
>non-antioxidant_534
MRRLEEDAVRVAYAGLRRKEAFKALAEKLGFTPLLFPVQATEKVPVPEYRDQVRALAQG
VDLFLATTGVGVRDLLEAGKALGLDLEGPLAKAFRLARGAKAARALKEAGLPPHAVGDG
TSKSLLPLLPQGRGVAALQLYGKPLPLLENALAERGYRVLPLMPYRHLPDPEGILRLEEAL
LRGEVDALAFVAAIQVEFLFEGAKDPKALREALNTRVKALAVGRVTADALREWGVKPFY
VDETERLGSLLQGFKRALQKEVA
>non-antioxidant_535
MSPQTETKASVGFKAGVKDYKLTYYTPEYETKDTDILAAFRVTPQPGVPPEEAGAAVAAE
SSTGTWTTVWTDGLTSLDRYKGRCYHIEPVVGEDNQYIAYVAYPLDLFEEGSVTNMFTSI
VGNVFGFKALRALRLEDLRIPPTYSKTFQGPPHGIQVERDKLNKYGRPLLGCTIKPKLGLS
AKNYGRACYECLRGGLDFTKDDENVNSQPFMRWRDRFVFCAEAIYKSQAETGEIKGHYL
NATAGTCEEMIKRAVFARELGVPIVMHDYLTGGFTANTSLAHYCRDNGLLLHIHRAMHAV
IDRQKNHGMHFRVLAKALRMSGGDHIHAGTVVGKLEGEREMTLGFVDLLRDDFIEKDRA
RGIFFTQDWVSMPGVIPVASGGIHVWHMPALTEIFGDDSVLQFGGGTLGHPWGNAPGAA
ANRVALEACVQARNEGRDLAREGNEIIRSACKWSPELAAACEIWKAIKFEFEPVDKLDS
>non-antioxidant_536
ATSDSNMLLNYVPVYVMLPLGVVNVDNVFEDPDGLKEQLLQLRAAGVDGVMVDVWW
GIIELKGPKQYDWRAYRSLLQLVQECGLTLQAIMSFHQCGGNVGDIVNIPIPQWVLDIGES
NHDIFYTNRSGTRNKEYLTVGVDNEPIFHGRTAIEIYSDYMKSFRENMSDFLESGLIIDIEVG

LGPAGELRYPSYPQSQGWEFPGIGEFQCYDKYLKADFKAAVARAGHPEWELPDDAGKYN
DVPESTGFFKSNGTYVTEKGKFFLTWYSNKLLNHGDQILDEANKAFLGCKVKLAIKVSGI
HWWYKVENHAAELTAGYYNLNDRDGYRPIARMLSRHHAILNFTCLEMRDSEQPSDAKS
GPQELVQQVLSGGWREDIRVAGENALPRYDATAYNQIILNARPQGVNNNGPPKLSMFGVT
YLRLSDDLLQKSNFNIFKKFVLKMHADQDYCANPQKYNHAITPLKPSAPKIPIEVLLEATK
PTLPFPWLPETDMKVDG
>non-antioxidant_537
MEKIMPEEEYSEFKELILQKELHVVYALSHVCGQDRTLLASILLRIFLHEKLESLLLCTLND
REISMEDEATTLFRATTLASTLMEQYMKATATQFVHHALKDSILKIMESKQSCELSPSKLE
KNEDVNTNLTHLLNILSELVEKIFMASEILPPTLRYIYGCLQKSVQHKWPTNTTMRTRVVS
GFVFLRLICPAILNPRMFNIISDSPSPIAARTLILVAKSVQNLANLVEFGAKEPYMEGVNPFIK
SNKHRMIMFLDELGNVPELPDTTEHSRTDLSRDLAALHEICVAHSDELRTLSNERGAQQH
VLKKLLAITELLQQKQNQYTKTNDVR
>non-antioxidant_538
DTASDAAAAAALTAANAKAAAELTAANAAAAAAATARM
>non-antioxidant_539
MIQQESRLKVADNSGAREVLVIKVLGGSGRRYANIGDVVVATVKDATPGGVVKKGQVVK
AVVVRTKRGVRRPDGSYIRFDENACVIIRDDKSPRGTRIFGPVARELRDKDFMKIISLAPEV
I
>non-antioxidant_540
MWMPPRPEEVARKLRRLGFVERMAKGGHRLYTHPDGRIVVVPFHSGELPKGTFKRILRD
AGLTEEEFHNL
>non-antioxidant_541
DNDSVVEEHGQLSISNGELVNERGEQVQLKGMSSHGLQWYGQFVNYESMKWLRDDWGI
NVFRAAMYTSSGGYIDDPSVKEKVKEAVEAAIDLDIYVIIDWHILSDNDPNIYKEEAKDFF
DEMSELYGDYPNVIYEIANEPNGSDVTWGNQIKPYAEEVIPIIRNNDPNNIIIVGTGTWSQD
VHHAADNQLADPNVMYAFHFYAGTHGQNLRDQVDYALDQGAAIFVSEWGTSAATGDG
GVFLDEAQVWIDFMDERNLSWANWSLTHKDESSAALMPGANPTGGWTEAELSPSGTFV
REKIRES
>non-antioxidant_542
GPHMATADRDILARLHKAVTSHYHAITQEFENFDTMKTNTISREEFRAICNRRVQILTDEQF
DRLWNEMPVNAKGRLKYPDFLSRFSSETAATPMATGDSAVAQR
>non-antioxidant_543
GPLGSQVRVKAYYRGDIMITHFEPSISFEGLCNEVRDMCSFDNEQLFTMKWIDEEGDPCT
VSSQLELEEAFRLYELNKDSELLIHVFPC
>non-antioxidant_544
GPHMSIVEVKSKFDAEFRRFALPRASVSGFQEFSRLLRAVHQIPGLDVLLGYTDAHGDLLP
LTNDDSLHRALASGPPPLRLLVQKR
>non-antioxidant_545
MVPAPEAIRQALQERLLARLDHPDPLYRDLLQDYPRRGGKMLRGLLTVYSALAHGAPLE
AGLEAATALELFQNWVLVHDDIEDGSEERRGRPALHRLHPMPLALNAGDAMHAEMWGL
LAEGLARGLFPPEVLLEFHEVVRRTAYGQHLDLLWTLGGTFDLRPEDYFRMVAHKAAYY
TAVAPLRLGALLAGKTPPAAYEEGGLRLGTAFQIVDDVLNLEGGEAYGKERAGDLYEGKR
TLILLRFLEEAPPEERARALALLALPREAKPEAEVGWLLERLLASRALAWAKAEAKRLQA

EGLALLEAAFQDLPGKEALDHLRGLLAALVERRA
>non-antioxidant_546
MIKMFKYKQVIVARADLKLSKGKLAAQVAHGAVTAAFEAYKKKREWFEAWFREGQKKV
VVKVESEEELFKLKAEAEKLGLPNALIRDAGLTEIPPGTVTVLAVGPAPEEIVDKVTGNLK
LL
>non-antioxidant_547
MVRVGMRAAPRVSLEALKAALGGLKLSEAKVYLITDWQDKRDQARYALLLHTGKKDLL
VPDAFGPAFPGGEEALSELVGLLLAQGARRFYEAVVSPGEMTALLDLPPEELLKRVMAIAN
PTDPGIYLKRAA
>non-antioxidant_548
MQDPSVYVRFPLKEPKKLGLEKASLLIWTTTPWTLPGNVAAAVHPEYTYAAFQVGDEALI
LEEGLGRKLLGEGTPVLKTFPGKALEGLPYTPPYPQALEKGYFVVLADYVSQEDGTGIVH
QAPAFGAEDLETARVYGLPLLKTVDEEGKLLVEPFKGLYFREANRAILRDLRGRGLLFKEE
SYLHS
>non-antioxidant_549
GSHMEEDWIREYPPITSDQQRQLYKRNFDTGLQEYKSLQSELDEINKELSRLDKELDDYR
EESEEYMAAADEYNRLKQVKGSADYKSKKNHCKQLKSKLSHIKKMVGDYDRQKT
>non-antioxidant_550
MEKILIFGHQNPDTDTICSAIAYADLKNKLGFNAEPVRLGQVNGETQYALDYFKQESPRLV
ETAANEVNGVILVDHNERQQSIKDIEEVQVLEVIDHHRIANFETAEPLYYRAEPVGCTATIL
NKMYKENNVKIEKEIAGLMLSAIISDSLLFKSPTCTDQDVAAAKELAEIAGVDAEEYGLN
MLKAG
>non-antioxidant_551
TLHKERRIGRLSVLLLLNEAEESTQVEELERDGWKVCLGKVGSMDAHKVIAAIETASKKS
GVIQSEGYRESHALYHATMEALHGVTRGEMLLGSLLRTVGLRFAVLRGNPYESEAEGDWI
AVSLYGTIGAPIKGLEHETFGVGINHI
>non-antioxidant_552
AIHCPPCSEEKLARCRPPVGCEELVREPGCGCCATCALGLGMPCGVYTPRCGSGLRCYPPR
GVEKPLHTLMHGQGVCMEL
>non-antioxidant_553
MATVHKVGDSTGWTTLVPYDYAKWASSNKFHVGDSLLFNYNNKFHNVLQVDQEQFKSC
NSSSPAASYTSGADSIPLKRPGTFYFLCGIPGHCQLGQKVEIKVDPGSSSA
>non-antioxidant_554
GAMAEAEAEVTLRELQEALEEEVLTRQSLSREMEAIRTDNQNFASQLREAEARNRDLEAH
VRQLQERMELLQAEGATAVTG
>non-antioxidant_555
MSASHADQPTQTVSYPQLIDLLRRIFVVHGTSPEVADVLAENCASAQRDGSHSHGIFRIPG
YLSSLASGWVDGKAVPVVEDVGAAFVRVDACNGFAQPALAAARSLLIDKARSAGVAILAI
RGSHHFAALWPDVEPFAEQGLVALSMVNSMTCVVPHGARQPLFGTNPIAFGAPRAGGEPI
VFDLATSAIAHGDVQIAAREGRLLPAGMGVDRDGLPTQEPRAILDGGALLPFGGHKGSAL
SMMVELLAAGLTGGNFSFEFDWSKHPGAQTPWTGQLLIVIDPDKGAGQHFAQRSEELVR
QLHGVGQERLPGDRRYLERARSMAHGIVIAQADLERLQELAGH
>non-antioxidant_556
GSDEAAELMQQVNVLKLTVEDLEKERDFYFGKLRNIELICQENEGENDPVLQRIVDILYAT

DEGFVIPDEGGPQEEQEEY
>non-antioxidant_557
SSYSGPIVVDPVTRIEGHLRIEVEVENGKVKNAYSSSTLFRGLEIILKGRDPRDAQHFTQRT
CGVCTYTHALASTRCVDNAVGVHIPKNATYIRNLVLGAQYLHDHIVHFYHLHALDFVDV
TAALKADPAKAAKVASSISPRKTTAADLKAVQDKLKTFVETGQLGPFTNAYFLGGHPAYY
LDPETNLIATAHYLEALRLQVKAARAMAVFGAKNPHTQFTVVGGVTCYDALTPQRIAEFE
ALWKETKAFVDEVYIPDLLVVAAAYKDWTQYGGTDNFITFGEFPKDEYDLNSRFFKPGV
VFKRDFKNIKPFDKMQIEEHVRHSWYEGAEARHPWKGQTQPKYTDLHGDDRYSWMKA
PRYMGEPMETGPLAQVLIAYSQGHPKVKAVTDAVLAKLGVGPEALFSTLGRTAARGIETA
VIAEYVGVMLQEYKDNIAKGDNVICAPWEMPKQAEGVGFVNAPRGGLSHWIRIEDGKIG
NFQLVVPSTWTLGPRCDKNKLSPVEASLIGTPVADAKRPVEILRTVHSFDPCIACGVH
>non-antioxidant_558
LMGPRRPSVVYLHNAECTGCSESVLRAFEPYIDTLILDTLSLDYHETIMAAAGDAAEAAL
EQAVNSPHGFIAVVEGGIPTAANGIYGKVANHTMLDICSRILPKAQAVIAYGTCATFGGVQ
AAKPNPTGAKGVNDALKHLGVKAINIAGCPPNPYNLVGTIVYYLKNKAAPELDSLNRPT
MFFGQTVHEQCPRLPHFDAGEFAPSFESEEARKGWCLYELGCKGPVTMNNCPKIKFNQTN
WPVDAGHPCIGCSEPDFWDAMTPFYQN
>non-antioxidant_559
SEQNNAVLPKGVTQGEFNKAVQKFRALLGDDNVLVESDQLVPYNKIMMPVENAAHAPSA
AVTATTVEQVQGVVKICNEHKIPIWTISTGRNFGYGSAAPVQRGQVILDLKKMNKIIKIDPE
MCYALVEPGVTFGQMYDYIQENNLPVMLSFSAPSAIAGPVGNTMDRGVGYTPYGEHFM
MQCGMEVVLANGDVYRTGMGGVPGSNTWQIFKWGYGPTLDGMFTQANYGICTKMGF
WLMPKPPVFKPFEVIFEDEADIVEIVDALRPLRMSNTIPNSVVIASTLWEAGSAHLTRAQY
TTEPGHTPDSVIKQMQKDTGMGAWNLYAALYGTQEQVDVNWKIVTDVFKKLGKGRIVT
QEEAGDTQPFKYRAQLMSGVPNLQEFGLYNWRGGGGSMWFAPVSEARGSECKKQAAM
AKRVLHKYGLDYVAEFIVAPRDMHHVIDVLYDRTNPEETKRADACFNELLDEFEKEGYAV
YRVNTRFQDRVAQSYGPVKRKLEHAIKRAVDPNNILAPGRSGIDLNNDF
>non-antioxidant_560
GAACNVLFINSVEMESLTGPQAISKAVAETLVADPTPTATIVHFKVSAQGITLTDNQRKLFF
RRHYPLNTVTFCDLDPQERKWTKTDGSGPAKLFGFVARKQGSTTDNVCHLFAELDPDQP
AAAIVNFVSRVML
>non-antioxidant_561
MTDMSIKFELIDVPIPQGTNVIIGQAHFIKTVEDLYEALVTSVPGVKFGIAFCEASGKRLVR
HEANDEELRNLAIDLCKKIAAGHVFVIYIRNAWPINVLNAIKNVPEVVRIFAATANPLKVIV
AEVEPERRGVVGVVDGHSPLGVETEKDREERKKFLREVVKYKL
>non-antioxidant_562
MLMKVAEFERLFRQAAGLDVDKNDLKRVSDFLRNKLYDLLAVAERNAKYNGRDLIFEPD
LPIAKGLQETLQEFRRMDTALELKPVLDALAALPPLDLEVAEDVRNLLPELAGALVVAYAR
VLKELDPALKNPQTEHHERAERVFNLLL
>non-antioxidant_563
MTVRKNQATLTADEKRRFVAAVLELKRSGRYDEFVRTHNEFIMSDTDSGERTGHRSPSFLP
WHRRFLLDFEQALQSVDSSVTLPYWDWSADRTVRASLWAPDFLGGTGRSTDGRVMDGPF
AASTGNWPINVRVDSRTYLRRSLGGSVAELPTRAEVESVLAISAYDLPPYNSASEGFRNHL
EGWRGVNLHNRVHVWVGGQMATGVSPNDPVFWLHHAYVDKLWAEWQRRHPDSAYVP

TGGTPDVVDLNETMKPWNTVRPADLLDHTAYYTFDALEHHHHHH
>non-antioxidant_564
MPEITRRRALTAAAAVAATASAAVTLAAPAASAAGHHEPAAPESFDEVYKGRRIQGRPARG
AAHHHEHGGGYEVFVDGVQLHVMRNADGSWISVVSHYDPVPTPRAAARAAVDELQGA
PLLPFPANLEHHHHHH
>non-antioxidant_565
LSDEDFKAVFGMTRSAFANLPLWLQQHLKKEKGLF
>non-antioxidant_566
MTTATAGLAVELKQSTAQAHEKAEHSTFMSDLLKGRLGVAEFTRLQEQAWLFYTALEQAV
DAVRASGFAESLLDPALNRAEVLARDLDKLNGSSEWRSRITASPAVIDYVNRLEEIRDNVD
GPALVAHHYVRYLGDLSGGQVIARMMQRHYGVDPEALGFYHFEGIAKLKVYKDEYREK
LNNLELSDEQREHLLKEATDAFVFNHQVFADLGKGL
>non-antioxidant_567
MVDIVKRRDWEKKEKKKIAIERIDTLFTLAERVARYSPDLAKRYVELALEIQKKAKVKIPR
KWKRRYCKRCHTFLIPGVNARVRLRTKRMPHVVITCLECGYIMRYPYLREVKQKRKKAT
>non-antioxidant_568
MEKKALTLAERQRLIVEGLPHVSATLARRLLKHFGSVERVFTASVAELMKVEGIGEKIAKE
IRRVITAPYIEDEE
>non-antioxidant_569
MIEKVYCQEVKPELDGKKVRLAGWVYTNMRVGKKIFLWIRDSTGIVQAVVAKNVVGEET
FEKAKKLGRESSVIVEGIVKADERAPGGAEVHVEKLEVIQAVSEFPIPENPEQASPELLLDY
RHLHIRTPKASAIMKVKETLIMAAREWLLKDGWHEVFPPILVTGAVEGGATLFKLKYFDK
YAYLSQSAQLYLEAAIFGLEKVWSLTPSFRAEKSRTRRHLTEFWHLELEAAWMDLWDIMK
VEEELVSYMVQRTLELRKKEIEMFRDDLTTLKNTEPPFPRISYDEAIDILQSKGVNVEWGD
DLGADEERVLTEEFDRPFFVYGYPKHIKAFYMKEDPNDPRKVLASDMLAPEGYGEIIGGS
QREDDYDKLLNRILEEGMDPKDYEWYLDLRRYGSVPHSGFGLGVERLVAWVLKLDHIRW
AALFPRTPARLYP
>non-antioxidant_570
GSHMDINNKARIHWACRRGMRELDISIMPFFEHEYDSLSDDEKRIFIRLLECDDPDLFNWL
MNHGKPADAELEMMVRLIQTRNRERGPVAI
>non-antioxidant_571
MAHHHHHHMSDEDHDFSHQGGGDNASKTYPLAAGALKKGGYVCINGRPCKVIDLSVSK
TGKHGHAKVSIVATDIFTGNRLEDQAPSTHNVEVPFVKTYTYSVLDIQANEDPSLPAHLSL
MDDEGESREDLDMPPDPALATQIKEQFDSGKDVLVVVVSAMGTEQVLQTKNAAEK
>non-antioxidant_572
ALEGTEFARSEGASALASVNPLKTTVEEALSRGWSVKSGTGTEDATKKEVPLGVAADAN
KLGTIALKPDPADGTADITLTFTMGGAGPKNKGKIITLTRTAADGLWKCTSDQDEQFIPKG
CSR
>non-antioxidant_573
ACTKNAIAQTGFNKDKYFNGDVWYVTDYLDLEPDDVPKRYCAALAAGTASGKLKEALY
HYDPKTQDTFYDVSELQVESLGKYTANFKKVDKNGNVKVAVTAGNYYTFTVMYADDSS
ALIHTCLHKGNKDLGDLYAVLNRNKDAAAGDKVKSAVSAATLEFSKFISTKENNCAYDND
SLKSLLTK
>non-antioxidant_574

GAMDSSEMSTICDKTLNPSFCLKFLNTKFASANLQALAKTTLDSTQARATQTLKKLQSIID
GGVDPRSKLAYRSCVDEYESAIGNLEEAFEHLASGDGMGMNMKVSAALDGADTCLDDV
KRLRSVDSSVVNNSKTIKNLCGIALVISNMLPRN
>non-antioxidant_575
EFALLTCRLDPPSQDLKDGTQEEATKRQEAPVDPRPEGDPQRTVISWRGAVIEPEQGTELPS
RRAEVPTKPPLPPARTQGTPVHLNYRQKGVIDVFLHAWKGYRKFAWGHDELKPVSRSFSE
WFGLGLTLIDALDTMWILGLRKEFEEARKWVSKKLHFEKDVDVNLFESTIRILGGLLSAY
HLSGDSLFLRKAEDFGNRLMPAFRTPSKIPYSDVNIGTGVAHPPRWTSDSTVAEVTSIQLEF
RELSRLTGDKKFQEAVEKVTQHIHGLSGKKDGLVPMFINTHSGLFTHLGVFTLGARADSY
YEYLLKQWIQGGKQETQLLEDYVEAIEGVRTHLLRHSEPSKLTFVGELAHGRFSAKMDHL
VCFLPGTLALGVYHGLPASHMELAQELMETCYQMNRQMETGLSPEIVHFNLYPQPGRRD
VEVKPADRHNLLRPETVESLFYLYRVTGDRKYQDWGWEILQSFSRFTRVPSGGYSSINNV
QDPQKPEPRDKMESFFLGETLKYLFLLFSDDPNLLSLDAYVFNTEAHPLPIWTPA
>non-antioxidant_576
MSQLLQDYLNWENYILRRVDFPTSYVVEGEVVRIEAMPRLYISGMGGSGVVADLIRDFSL
TWNWEVEVIAVKDYFLKARDGLLIAVSYSGNTIETLYTVEYAKRRRIPAVAITTGGRLAQM
GVPTVIVPKASAPRAALPQLLTAALHVVAKVYGIDVKIPEGLEPPNEALIHKLVEEFQKRPT
IIAAESMRGVAYRVKNEFNENAKIEPSVEILPEAHHNWIEGSERAVVALTSPHIPKEHQERV
KATVEIVGGSIYAVEMHPKGVLSFLRDVGIASVKLAEIRGVNPLATPRIDALKRRLQ
>non-antioxidant_577
GSHMAVYVKFKVPEEIQKELLDAVAKAQKIKKGANEVTKAVERGIAKLVIIAEDVKPEEVV
AHLPYLCEEKGIPYAYVASKQDLGKAAGLEVAASSVAIINEGDAEELKVLIEKVNVLKQ
>non-antioxidant_578
MEGQRWLPLEANPEVTNQFLKQLGLHPNWQFVDVYGMDPELLSMVPRPVCAVLLLFPIT
EKYEVFRTEEEEKIKSQGQDVTSSVYFMKQTISNACGTIGLIHAIANNKDKMHFESGSTLK
KFLEESVSMSPEERARYLENYDAIRVTHETSAHEGQTEAPSIDEKVDLHFIALVHVDGHLY
ELDGRKPFPINHGETSDETLLEDAIEVCKKFMERDPDELRFNAIALSAA
>non-antioxidant_579
GHMDQSDFSPYIEIDLPSESRIQSLHKSGLAAQEWVACEKVHGTNFGIYLINQGDHEVVRF
AKRSGIMDPNENFFGYHILIDEFTAQIRILNDLLKQKYGLSRVGRLVLNGELFGAKYKHPL
VPKSEKWCTLPNGKKFPIAGVQIQREPFPQYSPELHFFAFDIKYSVSGAEEDFVLLGYDEF
VEFSSKVPNLLYARALVRGTLDECLAFDVENFMTPLPALLGLGNYPLEGNLAEGVVIRHV
RRGDPAVEKHNVSTIIKLRCSSFMELKHPGKQKE
>non-antioxidant_580
NMNIEEFTSGLAEKGISLSPRQLEQFELYYDMLVEWNEKINLTSITEKKEVYLKHFYDSITA
AFYVDFNQVNTICDVGAGAGFPSLPIKICFPHLHVTIVDSLNKRITFLEKLSEALQLENTTF
CHDRAETFGQRKDVRESYDIVTARAVARLSVLSELCLPLVKKNGLFVALKAASAEEELNA
GKKAITTLGGELENIHSFKLPIEESDRNIMVIRKIKNTPKKYPRKPGTPNKSPIEG
>non-antioxidant_581
MLDAFSRVVTNADSKAAYVGGADLQALKKFISEGNKRLDSVNSIVSNASCIVSDAVSGMI
CENPSLISPSGNCYTNRRMAACLRDGEIILRYVSYALLSGDASVLEDRCLNGLKETYSSLG
VPANSNARAVSIMKACAVAFVNNTASQKKLSTPQGDCSGLASEVGGYFDKVTAAIS
>non-antioxidant_582
SLHSPGKAFRAALTKENPLQIVGTINANHALLAQRAGYQAIYLSGGGVAAGSLGLPDLGIS

TLDDVLTDIRRITDVCSLPLLVDADIGFGSSAFNVARTVKSMIKAGAAGLHIEDQVGAKRS
GHRPNKAIVSKEEMVDRIRAAVDAKTDPDFVIMARTDALAVEGLDAAIERAQAYVEAGA
EMLFPEAITELAMYRQFADAVQVPILANITEFGATPLFTTDELRSAHVAMALYPLSAFRAM
NRAAEHVYNVLRQEGTQKSVIDTMQTRNELYESINYYQYEEKLDNLFARSQVK
>non-antioxidant_583
MAQQKKTIAVVGATGRQGASLIRVAAAVGHHVRAQVHSLKGLIAEELQAIPNVTLFQGPL
LNNVPLMDTLFEGAHLAFINTTSQAGDEIAIGKDLADAAKRAGTIQHYIYSSMPDHSLYGP
WPAVPMWAPKFTVENYVRQLGLPSTFVYAGIYNNNFTSLPYPLFQMELMPDGTFEWHAP
FDPDIPLPWLDAEHDVGPALLQIFKDGPQKWNGHRIALTFETLSPVQVCAAFSRALNRRVT
YVQVPKVEIKVNIPVGYREQLEAIEVVFGEHKAPYFPLPEFSRPAAGSPKGLGPANGKGAG
AGMMQGPGGVISQRVTDEARKLWSGWRDMEEYAREVFPIEEEANGLDWML
>non-antioxidant_584
RTNQAGLELIGNAEGCRRDPYMCPAGVWTDGIGNTHGVTPGVRKTDQQIAADWEKNILI
AERCINQHFRGKDMPDNAFSAMTSAAFNMGCNSLRTYYSKARGMRVETSIHKWAQKGE
WVNMCNHLPDFVNSNGVPLRGLKIRREKERQLCLTGLVNEHHHHHH
>non-antioxidant_585
GSHMASLDMAEIKEKICDYLFNVSDSSALNLAKNIGLTKARDINAVLIDMERQGDVYRQG
TTPPIWHLTDKKRERMQIK
>non-antioxidant_586
SATEPPKIVWNEGKRRFETEDHEAFIEYKMRNNGKVMDLVHTYVPSFKRGLGLASHLCV
AAFEHASSHSISIIPSCSYVSDTFLPRNPSWKPLIHSEVFKSSI
>non-antioxidant_587
GSDDSYARVRAVVMTRDDSSGGWLQLGGGGLSSVTVSKTLQPGDSGGTEFLVHGERLRD
KTVVLECVLRRDLVYNKVTPTFHHWRIGDKKFGLTFQSPADARAFDRGIRRAIEDLSQG
>non-antioxidant_588
GHMQRERTAESSLRVISKEKNSITVEMINYDNTLLRTLVEEILKDDQVDEARYYIKHPVIDN
PQIYVRVKSGKPQSAIKRAVRKLSKLYEDLGTQFQKEFQRYESDHMIKAVEGS
>non-antioxidant_589
MAAESQLKRVIETLRRLGIEEVLKLERRDPQYRAVCNVVKRHGETVGSRLAMLNALISYR
LTGKGEEHWEYFGKYFSQLEVIDLCRDFLKYIETSPFLKIGVEARKKRALKACDYVPNLE
DLGLTLRQLSHIVGARREQKTLVFTIKILNYAYMCSRGVNRVLPFDIPIPVDYRVARLTWCA
GLIDFPPEEALRRYEAVQKIWDAVARETGIPPLHLDTLLWLAGRAVLYGENLHGVPKEVIA
LFQWRGGCRPPSE
>non-antioxidant_590
MAKLTSAVPVLTARDVAEAVEFWTDRLGFSRVFVEDDFAGVVRDDVTLFISAVQDQVVPD
NTQAWVWVRGLDELYAEWSEVVSTNFRDASGPAMTEIVEQPWGREFALRDPAGNCVHFV
AEEQD
>non-antioxidant_591
GASHPEIEKAQREIIEAFNAKPKNGINKIKEICEQYKISPNEEIAEFFHQQRKNLDLEAVGDY
LSSPEAENQQVLKAFTSQMNFNGQSFVEGLRTFLKTFKLPGEAQKIDRLVQSFSGAYFQQN
PDVVSNADAAYLLAFQTIMLNTDLHNPSIPEKNKMTVDGLKRNLRGGNNGGDFDAKFLE
ELYSEIKAKPFELNFVKTSPGYELTSTTLNKDSTFKKLDSFLHSTDVNINTVFPGIGDNVKT
TVDQPKSWLSFFTGYKGTITLTDNKTSAQATIQVYTPNIFSKWLFGEQPRVIIQPGQTKESID
LAAKAAADFSSPVKNFKATYDYEVGDLIKAYDNQKKLITIERNLALKA

>non-antioxidant_592
MGSSHHHHHHSSGLVPRGSHMHNYVIIDAFASVPLEGNPVAVFFDADDLPPAQMQRIARE
MNLSESTFVLKPRNGGDALIRIFTPVNELPFAGAPLLGTAIALGAHTDNHRLYLETQMGTI
AFELERQNGSVIAASMDQPIPTWTALGRDAELLKALGISDSTFPIEIYHNGPRHVFVGLPSI
DALSALHPDHRALSNFHDMAINCFAGAGRRWRSRMFSPAYGVVEDAATGSAAGPLAIHL
ARHGQIEFGQPVEILQGVEIGRPSLMFAKAEGRAEQLTRVEVSGNGVTFGRGTIVL
>non-antioxidant_593
MVKVKFKYKGEEKEVDTSKIKKVWRAGKAVSFTYDDNGKTGRGAVSEKDAPKELLDML
ARAEREKK
>non-antioxidant_594
EEYSSNWAGAVLIGDGYTKVTGEFTVPSVSAGSSGSSGY
>non-antioxidant_595
QSEEYCASAWVGIDGDTCETAILQTGVDFCYEDGQTSYDAWYEWYPDYAYDFSDITISEG
DSIKVTVEATSKSSGSATVENLTTGQSVTHTFSGNVEGDLCETNAEWIVEDFESGDSLVAF
ADFGSVTFTNAEATSGGSTVGPSDATVMDIEQDGSVLTETSVSGDSVTVTYV
>non-antioxidant_596
FNYDQPYRGQYHFSPQKNWMNDPNGLLYHNGTYHLFFQYNPGGIEWGNISWGHAISEDL
THWEEKPVALLARGFGSDVTEMYFSGSAVADVNNTSGFGKDGKTPLVAMYTSYYPVAQT
LPSGQTVQEDQQSQSIAYSLDDGLTWTTYDAANPVIPNPPSPYEAEYQNFRDPFVFWHDE
SQKWVVVVTSIAELHKLAIYTSDNLKDWKLVSEFGPYNAQGGVWECPGLVKLPLDSGNST
KWVITSGLNPGGPPGTVGSGTQYFVGEFDGTTFTPDADTVYPGNSTANWMDWGPDFYA
AAGYNGLSLNDHVHIGWMNNWQYGANIPTYPWRSAMAIPRHMALKTIGSKATLVQQPQ
EAWSSISNKRPIYSRTFKTLSEGSTNTTTTGETFKVDLSFSAKSKASTFAIALRASANFTEQT
LVGYDFAKQQIFLDRTHSGDVSFDETFASVYHGPLTPDSTGVVKLSIFVDRSSVEVFGGQG
ETTLTAQIFPSSDAVHARLASTGGTTEDVRADIYKIASTWN
>non-antioxidant_597
MQQSLAVKTFEDLFAELGDRARTRPADSTTVAALDGGVHALGKKLLEEAGEVWLAAEHE
SNDALAEEISQLLYWTQVLMISRGLSLDDVYRKL
>non-antioxidant_598
MGSSHHHHHHSSGRENLYFQGHMFFTDWEGPWILTDFALELCMAVFNNARFFSNLSEYD
DYLAYEVRREGYEAGYTLKLLTPFLAAAGVKNRDVERIAELSAKFVPDAEKAMATLQER
WTPVVISTSYTQYLRRTASMIGVRGELHGTEVDFDSIAVPEGLREELLSIIDVIASLSGEELF
RKLDELFSRSEVRKIVESVKAVGAGEKAKIMRGYCESKGIDFPVVVGDSISDYKMFEAAR
GLGGVAIAFNGNEYALKHADVVIISPTAMSEAKVIELFMERKERAFEVLSAVSIPETEIYIME
NSDFGEVLEKSKRMRVRLRGLAGELGGS
>non-antioxidant_599
SSSNSEKEWHIVPVSKDYFSIPNDLLWSFNTTNKSINVYSKCISGKAVYSFNAGKFMGNFN
VKEVDGCFMDAQKIAIDKLFSMLKDGVVLKGNKINDTILIEKDGEVKLKLIRGI
>non-antioxidant_600
AHHHHHHGSMLKEVHELLNRIWGDIFELREELKEELKGFTVEEVSEVFNAYLYIDGKWEE
MKYPHPAFAVKPGGEVGATPQGFYFVFAFPKEELSKEFIEDVIRAFEKLFIYGAENFLEDFY
NFEHPISGDEVWDRIVNSDEEMINFEVDLGFDKEEVKREIKRFIELARRYNLL
>non-antioxidant_601
GSIINETADDIVYRLTVIIDDRYESLKNLITLRADRLEMIINDNVSTILASI

>non-antioxidant_602
MKMKEFLDLLNESRLTVTLTGAGISTPSGIPDFRGPNGIYKKYSQNVFDIDFFYSHPEEFYR
FAKEGIFPMLQAKPNLAHVLLAKLEEKGLIEAVITQNIDRLHQRAGSKKVIELHGNVEEYY
CVRCEKKYTVEDVIKKLESSDVPLCDDCNSLIRPNIVFFGENLPQDALREAIGLSSRASLMI
VLGSSLVVYPAAELPLITVRSGGKLVIVNLGETPFDDIATLKYNMDVVEFARRVMEEGGIS
>non-antioxidant_603
MKEKIRKKILLAPEEPGVYIFKNKGVPIYIGKAKRLSNRLRSYLNPQTEKVFRIGEEADELE
TIVVMNEREAFILEANLIKKYRPKYNVRLKDTDF
>non-antioxidant_604
MKIIITGEPGVGKTTLVKKIVERLGKRAIGFWTEEVRDPETKKRTGFRIITTEGKKKIFSSKF
FTSKKLVGSYGVNVQYFEELAIPILERAYREAKKDRRKVIIIDEIGKMELFSKKFRDLVRQI
MHDPNVNVVATIPIRDVHPLVKEIRRLPGAVLIELTPENRDVILEDILSLLER
>non-antioxidant_605
MQIVQIEQAPKDYISDIKIIPSKSLLLITSWDGSLTVYKFDIQAKNVDLLQSLRYKHPLLCCN
FIDNTDLQIYVGTVQGEILKVDLIGSPSFQALTNNEANLGICRICKYGDDKLIAASWDGLIE
VIDPRNYGDGVIAVKNLNSNNTKVKNKIFTMDTNSSRLIVGMNNSQVQWFRLPLCEDDN
GTIEESGLKYQIRDVALLPKEQEGYACSSIDGRVAVEFFDDQGDDYNSSKRFAFRCHRLNL
KDTNLAYPVNSIEFSPRHKFLYTAGSDGIISCWNLQTRKKIKNFAKFNEDSVVKIACSDNIL
CLATSDDTFKTNAAIDQTIELNASSIYIIFDYENP
>non-antioxidant_606
MEEIKCLLCRYLKERQEKFISDWKKKVIIRERDPYKEEIIKNGEHLLSAFIMYLKEEISLQEI
EITSKKIARERIDAKVNIAEFIHNTNVAKIEIMNILTLLNPDLQQYQALVKKINQFFDHLIYY
TVHSYYEQKA
>non-antioxidant_607
SNAMEFAPRSVVIEEFIDTLEPMMEAYGLDQVGIFEEHGEGNRYYVGYTINKDDEMITIHM
PFVKNERGELALEKQEWTVRKDGREKKGFHSLQEAMEEVIHS
>non-antioxidant_608
GSSGGPNVPELILQLLQLEPEEDQVRARIVGCLQEPAKSRSDQPAPFSLLCRMADQTFISIV
DWARRCMVFKELEVADQMTLLQNSWSELLVLDHIYRQVQYGKEDSILLVTGQEVELSTV
AVQAGSLLHSLVLRAQELVLQLHALQLDRQEFVCLKFLILFSLDVKFLNNHSLVKDAQEK
ANAALLDYTLSHYPHSGDKFQQLLLSLVEVRALSMQAKEYLYHKHLGNEMPRNNLLIEM
LQAKQT
>non-antioxidant_609
GSTVPYTITVNGTSQNILSNLTFNKNQNISYKDLEGKVKSVLESNRGITDVDLRLSKQAKY
TVNFKNGTKKVIDLKSGIYTANLINSSDIKSININID
>non-antioxidant_610
RVANCSAPCPQDWIWHGENCYLFSSGSFNWEKSQEKCLSLDAKLLKINSTADLDFIQQAIS
YSSFPFWMGLSRRNPSYPWLWEDGSPLMPHLFRVRGAVSQTYPSGTCAYIQRGAVYAENC
ILAAFSICQKKANL
>non-antioxidant_611
GAAASIQTTVNTLSERISSKLEQEANASAQTKCDIEIGNFYIRQNHGCNLTVKNMCSADAD
AQLDAVLSAATETYSGLTPEQKAYVPAMFTAALNIQTSVNTVVRDFENYVKQTCNSSAVV
DNKLKIQNVIIDECYGAPGSPTNLEFINTGSSKGNCAIKALMQLTTKATTQIAPKQVAGTG
VQ

>non-antioxidant_612
ADLPAPDDTGLQAVLHTALSQGAPGAMVRVDDNGTIHQLSEGVADRATGRAITTTDRFRV
GSVTKSFSAVVLLQLVDEGKLDLDASVNTYLPGLLPDDRITVRQVMSHRSGLYDYTNDMF
AQTVPGFESVRNKVFSYQDLITLSLKHGVTNAPGAAYSYSNTNFVVAGMLIEKLTGHSVA
TEYQNRIFTPLNLTDTFYVHPDTVIPGTHANGYLTPDEAGGALVDSTEQTVSWAQSAGAVI
SSTQDLDTFFSALMSGQLMSAAQLAQMQQWTTVNSTQGYGLGLRRRDLSCGISVYGHT
GTVQGYYTYAFASKDGKRSVTALANTSNNVNVLNTMARTLESAFCGKPTT
>non-antioxidant_613
MSTAVQFRGGTTAQHATFTGAAREITVDTDKNTVVVHDGATAGGFPLARHDLVKTAFIKA
DKSAVAFTRTGNATASIKAGTIVEVNGKLVQFTADTAITMPALTAGTDYAIYVCDDGTVRA
DSNFSAPTGYTSTTARKVGGFHYAPGSNAAAQAGGNTTAQINEYSLWDIKFRPAALDPRG
MTLVAGAFWADIYLLGVNHLTDGTSKYNVTIADGSASPKKSTKFGGDGSAAYSDGAWYN
FAEVMTHHGKRLPNYNEFQALAFGTTEATSSGGTDVPTTGVNGTGATSAWNIFTSKWGV
VQASGCLWTWGNEFGGVNGASEYTANTGGRGSVYAQPAAALFGGAWNGTSLSGSRAAL
WYSGPSFSFAFFGARGVCDHLILE
>non-antioxidant_614
GSALRELKVCLLGDTGVGKSSIMWRFVEDSFDPNINPTIGASFMTKTVQYQNELHKFLIW
DTAGLERFRALAPMYYRGSAAAIIVYDITKEETFSTLKNWVRELRQHGPPSIVVAIAGNKC
DLTDVREVMERDAKDYADSIHAIFVETSAKNAININELFIEISRRIPST
>non-antioxidant_615
DYKLFITEGYEVGRVNGLAVIGESAGIVLPIIAEVTPSMSKSEGRVIATGRLQEIAREAVMN
VSAIIKKYTGRDISNMDVHIQFVGTYEGVEGDSASISIATAVISAIEGIPVDQSVAMTGSLSV
KGEVLPVGGVTQKIEAAIQAGLKKVIIPKDNIDDVLLDAEHEGKIEVIPVSRINEVLEHVLE
DGKKKNRLMSKFKELELAAV
>non-antioxidant_616
GPLGSMTTKQTVSLFIWLPESKQKTLFISTKNHTQFELNNIIFDVTLSTELPDKEPNAIITKR
THPVGKMADEMRKYEKDHPKVLFLESSAIHDMMSSREEINALLIKNNIPIPNSFSVKSKEE
VIQLLQSKQLILPFIVKPENAQGTFNAHQMKIVLEQEGIDDIHFPCLCQHYINHNNKIVKVF
CIGNTLKWQTRTSLPNVHRCGIKSVDFNNQHLEDILSWPEGVIDKQDIIENSANRFGSKILE
DPILLNLTSEAEMRDLAYKVRCALGVQLCGIDFIKENEQGNPLVVDVNVFPSYGGKVDFD
WFVEKVALCYTEVAKI
>non-antioxidant_617
GSHMALKRIQKELQDLGRDPPAQCSAGPVGDDLFHWQATIMGPPESPYQGGVFFLTIHFPT
DYPFKPPKVAFTTRIYHPNINSNGSICLDILRSQWSPALTISKVLLSICSLLCDPNPDDPLVPEI
ARIYKTDRERYNQLAREWTQKYAM
>non-antioxidant_618
HMVTLYTSPSCTSCRKARAWLEEHEIPFVERNIFSEPLSIDEIKQILRMTEDGTDEIISTRSKV
FQKLNVNVESMPLQDLYRLINEHPGLLRRPIIIDEKRLQVGYNEDEIRRFLPRKVRSFQLRE
AQRLAN
>non-antioxidant_619
PEFMVTTEPALADLQEQLYNGNEKSQLAAMSTLSTAGTEGYHLLQEFLKDSATFSPPPAP
WIRGQAYRLLFHSPEASVQAFLQQHYPQGVIPLRSDRGVDYQELAKLLVAEKFEAADRLT
TQKLCELAGPLAQKRRWLYFTEVEQLPIPDLQTIDQLWLAFSLGRFGYSVQRQLWLGCGQ
NWDRLWEKIGWRQGKRWPRYPNEFIWDLSAPRGHLPLTNQLRGVQVLNALLNHPAWTA

>non-antioxidant_620
SNAMGLFDEVVGAFLKGDAGKYQAILSWVEEQGGIQVLLEKLQSGGLGAILSTWLSNQQ
RNQSVSGEQLESALGTNAVSDLGQKLGVDTSTASSLLAEQLPKIIDALSPQGEVSAQANND
LLSAGMELLKGKLFR
>non-antioxidant_621
SNAMDISVIDATKVNTETGLHIGESNAPVKMIEFINVRCPYCRKWFEESEELLAQSVKSGK
VERIIKLFDKEKESLQRGNVMHHYIDYSAPEQALSALHKMFATQDEWGNLTLEEVATYAE
KNLGLKEQKDATLVSAVIAEANAAHIQFVPTIIIGEYIFDESVTEEELRGYIEK
>non-antioxidant_622
EANAPGPVPGERQLAHSKMVPIPAGVFTMGTDDPQIKQDGEAPARRVTIDAFYMDAYEVS
NTEFEKFVNSTGYLTEAEKFGDSFVFEGMLSEQVKTNIQQAVAAAPWWLPVKGANWRHP
EGPDSTILHRPDHPVLHVSWNDAVAYCTWAGKRLPTEAEWEYSCRGGLHNRLFPWGNKL
QPKGQHYANIWQGEFPVTNTGEDGFQGTAPVDAFPPNGYGLYNIVGNAWEWTSDWWTV
HHSVEETLNPKGPPSGKDRVKKGGSYMCHRSYCYRYRCAARSQNTPDSSASNLGFRCAA
DRLPTMDRGSHHHHHH
>non-antioxidant_623
SNAMETQDYAFQPGLTVGELLKSSQKDWQAAINHRFVKELFAGTIENKVLKDYLIQDYHF
FDAFLSMLGACVAHADKLESKLRFAKQLGFLEADEDGYFQKAFKELKVAENDYLEVTLH
PVTKAFQDLMYSAVASSDYAHLLVMLVIAEGLYLDWGSKDLALPEVYIHSEWINLHRGPF
FAEWVQFLVDELNRVGKNREDLTELQQRWNQAVALELAFFDIGYDV
>non-antioxidant_624
MANYWLYKSEPFKWSWEMQKAKGETGEEWTGVRNYQARNNMRAMKIGDKGFFYHSN
EGLDVVGIVEVCALSHPDSTAEGDLKWDCVDIRAVCDMPQPVSLKDVKANPKLEKMSLV
TSMRLSVQPVTEEEYLEVCRMGGLANPPKSPDLEHHHHHH
>non-antioxidant_625
MSEAADVERVYAAMEEAAGLLGVACARDKIYPLLSTFQDTLVEGGSVVVFSMASGRHST
ELDFSISVPTSHGDPYATVVEKGLFPATGHPVDDLLADTQKHLPVSMFAIDGEVTGGFKKT
YAFFPTDNMPGVAELSAIPSMPPAVAENAELFARYGLDKVQMTSMDYKKRQVNLYFSELS
AQTLEAESVLALVRELGLHVPNELGLKFCKRSFSVYPTLNWETGKIDRLCFAVISNDPTLV
PSSDEGDIEKFHNYATKAPYAYVGEKRTLVYGLTLSPKEEYYKLGAYYHITDVQRGLLKAF
DSLED
>non-antioxidant_626
APRIKLKILNGSYGIARLSASEAIPAWADGGGFVSITRTDDELSIVCLIDRIPQDVRVDPGWS
CFKFQGPFAFDETGIVLSVISPLSTNGIGIFVVSTFDGDHLLVRSNDLEKTADLLANAGHSL
LLEHHHHHH
>non-antioxidant_627
GAMDPSQYASSSSWTSFLKSIASFNGDLSSLSAPPFILSPISLTEFSQYWAEHPELFLEPSFIN
DDNYKEHCLIDPEVESPELARMLAVTKWFISTLKSQYCSRNESLGSEKKPLNPFLGELFVG
KWENKEHPEFGETVLLSEQVSHHPPVTAFSIFNDKNKVKLQGYNQIKASFTKSLMLTVKQ
FGHTMLDIKDESYLVTPPPLHIEGILVASPFVELEGKSYIQSSTGLLCVIEFSGRGYFSGKKN
SFKARIYKDSKDSKDKEKALYTISGQWSGSSKIIKANKKEESRLFYDAARIPAEHLNVKPL
EEQHPLESRKAWYDVAGAIKLGDFNLIAKTKTELEETQRELRKEEEAKGISWQRRWFKDF
DYSVTPEEGALVPEKDDTFLKLASALNLSTKNAPSGTLVGDKEDRKEDLSSIHWRFQREL
WDEEKEIVL

>non-antioxidant_628
MLTEGISIQSYDGHTFGALVGSPAKAPAPVIVIAQDIFGVNAFMRETVSWLVDQGYAAVCP
DLYARQAPGTALDPQDERQREQAYKLWQAFDMEAGVGDLEAAIRYARHQPYSNGKVGL
VGYSLGGALAFLVASKGYVDRAVGYYGVGLEKQLNKVPEVKHPALFHMGGQDHFVPAP
SRQLITEGFGANPLLQVHWYEEAGHSFARTGSSGYVASAAALANERTLDFLVPLQSRKP
>non-antioxidant_629
AVSFIGSTENDVGPSQGSYSSTHAMDNLPFVYNTGHNIGYQNANVWRISGGFCVGLDGK
VDLPVVGSLDGQSIYGLTEEVGLLIWMGDTNYSRGTAMSGNSWENVFSGWCVGNYVST
QGLSVHVRPVILKRNSSAQYSVQKTSIGSIRMRPYNGSSAGSVQTTVNFSLNPFTLNDT
>non-antioxidant_630
GHMFEKIRKILADIEDSQNEIEMLLKLANLSLGDFIEIKRGSMDMPKGVNEAFFTQLSEEV
ERLKELINALNKIKKGLLVFGS
>non-antioxidant_631
GHMTSRPSSDQTWQPIDGRVALIAPASAIATDVLEATLRQLEVHGVDYHLGRHVEARYRY
LAGTVEQRLEDLHNAFDMPDITAVWCLRGGYGCGQLLPGLDWGRLQAASPRPLIGFSDIS
VLLSAFHRHGLPAIHGPVATGLGLSPLSAPREQQERLASLASVSRLLAGIDHELPVQHLGG
HKQRVEGALIGGNLTALACMAGTLGGLHAPAGSILVLEDVGEPYYRLERSLWQLLESIDA
RQLGAICLGSFTDCPRKEVAHSLERIFGEYAAAIEVPLYHHLPSGHGAQNRAWPYGKTAVL
EGNRLRWGS
>non-antioxidant_632
CYCRIPACIAGERRYATCIYQGRLWAFCC
>non-antioxidant_633
VCSCRLVFCRRTELRVGNCLIGGVSFTYCCTRV
>non-antioxidant_634
EAEAEFNSINACLAAADVEFHEEDSEGWDMDGTAFNLRVDYDPAAIAIPRSTEDIAAAVQ
CGLDAGVQISAKGGGHSYGSYGFGGEDGHLMLELDRMYRVSVDDNNVATIQGGARLGY
TALELLDQGNRALSHGTCPAVGVGGHVLGGGYGFATHTHGLTLDWLIGATVVLADASIVH
VSETENADLFWALRGGGGGFAIVSEFEFNTFEAPEIITTYQVTTTWNRKQHVAGLKALQD
WAQNTMPRELSMRLEINANALNWEGNFFGNAKDLKKILQPIMKKAGGKSTISKLVETDW
YGQINTYLYGADLNITYNYDVHEYFYANSLTAPRLSDEAIQAFVDYKFDNSSVRPGRGW
WIQWDFHGGKNSALAAVSNDETAYAHRDQLWLWQFYDSIYDYENNTSPYPESGFEFMQG
FVATIEDTLPEDRKGKYFNYADTTLTKEEAQKLYWRGNLEKLQAIKAKYDPEDVFGNVVS
VEPIAYLEQKLISEEDLNSAVDHHHHHH
>non-antioxidant_635
GSPLGQDVFLDYCQKLLEKFRYPWELMPLMYVILKDADANIEEASRRIEEGQYVVNEYSR
QHNLN
>non-antioxidant_636
ALGKLQDVVNQNAQALNTLVKQLSSNFGAISSVLNDISGGRGGDISGINASVVNIQKEIDR
LNEVAKNLNESLIDLQ
>non-antioxidant_637
MAIGYVWNTLYGWVDTGTGSLAAANLTARMQPISHHLAHPDTKRRFHELVCASGQIEHL
TPIAAVAATDADILRAHSAAHLENMKRVSNLPTGGDTGDGITMMGNGGLEIARLSAGGAV
ELTRRVATGELSAGYALVNPPGHHAPHNAAMGFCIFNNTSVAAGYARAVLGMERVAILDW
DVHHGNGTQDIWWNDPSVLTISLHQHLCFPPDSGYSTERGAGNGHGYNINVPLPPGSGNA

AYLHAMDQVVLPALRAYRPQLIIVGSGFDASMLDPLARMMVTADGFRQMARRTIDCAAD
ICDGRIVFVQEGGYSPHYLPFCGLAVIEELTGVRSLPDPYHEFLAGMGGNTLLDAERAAIE
EIVPLLADIR
>non-antioxidant_638
GAMGPIITTQVTIPKDLAGSIIGKGGQRIKQIRHESGASIKIDEPLEGSEDRIITITGTQDQIQN
AQYLLQNSVKQYSGKFF
>non-antioxidant_639
ADLEDNMETLNDNLKVIEKADNAAQVKDALTKMRAAALDAQKATPPKLEDKSPDSPEM
KDFRHGFDILVGQIDDALKLANEGKVKEAQAAAEQLKTTRNAYHQKYR
>non-antioxidant_640
GSHMASEELQKDLEEVKVLLEKATRKRVRDALTAEKSKIETEIKNKMQQK
>non-antioxidant_641
MGSDKIHHHHHHMNNQEKNMLGFNQDEYLTSAREIIAARQKAEQVADEIYQAGFSSLFFA
SVGGSLAPMMAINEFAKELTTLPVYVEQAAELIHKGNKRLNKDSVVITLSKSGDTKESVAI
AEWCKAQGIRVVAITKNADSPLAQAATWHIPMRHKNGVEYEYMLLYWLFFRVLSRNNEF
ASYDRFASQLEILPANLLKAKQKFDPQADAIASRYHNSDYMMWVGGAEMWGEVYLFSM
CILEEMQWKRTRPVSSAEFFHGALELLEKDVPLILVKGEGKCRALDERVERFASKITDNLV
VIDPKAYALDGIDDEFRWIMAPCVVSTLLVDRLAAHFEKYTGHSLDIRRYYRQFDY
>non-antioxidant_642
GSDASKLSSDYSLPDLINTRKVPNNWQTGEQASLEEGRIVLTSNQNSKGSLWLKQGFDLK
DSFTMEWTFRSVGYSGQTDGGISFWFVQDSNIPRDKQLYNGPVNYDGLQLLVDNNGPLG
PTLRGQLNDGQKPVDKTKIYDQSFASCLMGYQDSSVPSTIRVTYDLEDDNLLKVQVDNK
VCFQTRKVRFPSGSYRIGVTAQNGAVNNNAESFEIFKMQFFNGV
>non-antioxidant_643
MGSSHHHHHHSSGLVPRGSHMAVDSSNSATGPMRVFAIGNPILDLVAEVPSSFLDEFFLKR
GDATLATPEQMRIYSTLDQFNPTSLPGGSALNSVRVVQKLLRKPGSAGYMGAIGDDPRGQ
VLKELCDKEGLATRFMVAPGQSTGVCAVLINEKERTLCTHLGACGSFRLPEDWTTFASGA
LIFYATAYTLTATPKNALEVAGYAHGIPNAIFTLNLSAPFCVELYKDAMQSLLLHTNILFGNE
EEFAHLAKVHNLVAAEKTALSTANKEHAVEVCTGALRLLTAGQNTSATKLVVMTRGHNP
VIAAEQTADGTVVVHEVGVPVVAAEKIVDTNGAGDAFVGGFLYALSQGKTVKQCIMCGN
ACAQDVIQHVGFSLSFTFTSG
>non-antioxidant_644
HHHHHHMPVNQFTGYLKLTDNVAIKCVDIVKEAQSANPMVIVNAANIHLKHGGGVAGAL
NKATNGAMQKESDDYIKLNGPLTVGGSCLLSGHNLAKKCLHVVGPNLNAGEDIQLLKAA
YENFNSQDILLAPLLSAGIFGAKPLQSLQVCVQTVRTQVYIAVNDKALYEQVVMDYLDNL
KPRV
>non-antioxidant_645
MSAKSRTIGIIGAPFSKGQPRGGVEEGPTVLRKAGLLEKLKEQECDVKDYGDLPFADIPND
SPFQIVKNPRSVGKASEQLAGKVAEVKKNGRISLVLGGDHSLAIGSISGHARVHPDLGVIW
VDAHTDINTPLTTTSGNLHGQPVSFLLKELKGKIPDVPGFSWVTPCISAKDIVYIGLRDVDP
GEHYILKTLGIKYFSMTEVDRLGIGKVMEETLSYLLGRKKRPIHLSFDVDGLDPSFTPATGT
PVVGGLTYREGLYITEEIYKTGLLSGLDIMEVNPSLGKTPEEVTRTVNTAVAITLACFGLAR
EGNHKPIDYLNPPK
>non-antioxidant_646

TKFIGCIDLHNGEVKQIVGGTLTSKKEDVPKTNFVSQHPSSYYAKLYKDRDVQGCHVIKL
GPNNDDAAREALQESPQFLQVGGGINDTNCLEWLKWASKVIVTSWLFTKEGHFQLKRLE
RLTELCGKDRIVVDLSCRKTQDGRWIVAMNKWQTLTDLELNADTFRELRKYTNEFLIHAA
DVEGLCGGIDELLVSKLFEWTKDYDDLKIVYAGGAKSVDDLKLVDELSHGKVDLTFGSSL
DIFGGNLVKFEDCCRWNEKQG
>non-antioxidant_647
MWQQAIGDALGITARNLKKFGDRFPHVSDGSNKYVLNDNTDWTDGFWSGILWLCYEYT
GDEQYREGAVRTVASFRERLDRFENLDHHNIGFLYSLSAKAQWIVEKDESARKLALDAAD
VLMRRWRADAGIIQAWGPKGDPENGGRIIIDCLLNLPLLLWAGEQTGDPEYRRVAEAHAL
KSRRFLVRGDDSSYHTFYFDPENGNAIRGGTHQGNTDGSTWTRGQAWGIYGFALNSRYL
GNADLLETAKRMARHFLARVPEDGVVYWDFEVPQEPSSYRDSSASAITACGLLEIASQLD
ESDPERQRFIDAAKTTVTALRDGYAERDDGEAEGFIRRGSYHVRGGISPDDYTIWGDYYY
LEALLRLERGVTGYWYERGR
>non-antioxidant_648
SNAMFAPQGLAQFIKVNVTLENGEPVFIYTDANGQVCQGDITVTQAGTITYLLNDQTLKG
LKFVGVGFVTPFDGIIDAVTISSDGMLVQLVDLDKTPGTTKFQFVLSNTANTLLVLSPD
>non-antioxidant_649
TACTATQQTAAYKTLVSILSESSFSQCSKDSGYSMLTATALPTNAQYKLMCASTACNTMIK
KIVALNPPDCDLTVPTSGLVLDVYTYANGFSSKCASL
>non-antioxidant_650
VSRLEEDVRNLNAIVQKLQERLDRLEETVQAK
>non-antioxidant_651
SIEKIWAREILDSRGNPTVEVDLYTAKGLFRAAVPSGASTGIYEALELRDGDKQRYLGKGV
LKAVDHINSTIAPALISSGLSVVEQEKLDNLMLELDGTENKSKFGANAILGVSLAVCKAGA
AERELPLYRHIAQLAGNSDLILPVPAFNVINGGSHAGNKLAMQEFMILPVGAESFRDAMR
LGAEVYHTLKGVIKDKYGKDATNVGDEGGFAPNILENSEALELVKEAIDKAGYTEKIVIG
MDVAASEFYRDGKYDLDFKSPTDPSRYITGDQLGALYQDFVRDYPVVSIEDPFDQDDWAA
WSKFTANVGIQIVGDDLTVTNPKRIERAVEEKACNCLLLKVNQIGSVTEAIQACKLAQENG
WGVMVSHRSGETEDTFIADLVVGLCTGQIKTGAPCRSERLAKYNQLMRIEEELGDEARFA
GHNFRNPSVLHHHHHH
>non-antioxidant_652
AHMGIQRPTSTTTDKKEIKAYLKQVDKIKDDEEPIKTVGKKIAELDEKKKKLTEDVNSKDT
AVRGKAVKDLIKNADDRLKEFEKEEDAIKKSEQDFKKAKSHVDNIDNDVKRKEVKQLDD
VLKEKYKLHSDYAKAYKKAVNSEKTLFKYLNQNDATQQGVNEKSKAIEQNYKKLKEVS
DKYTKVLNKVQKEKQDVDQFK
>non-antioxidant_653
MEGGSITPGEDKPEIQSPIPPNQIFILSGQSNMAGRGGVFKDHHNNRWVWDKILPPECAPN
SSILRLSADLRWEEAHEPLHVDIDTGKVCGVGPGMAFANAVKNRLETDSAVIGLVPCASG
GTAIKEWERGSHLYERMVKRTEESRKCGGEIKAVLWYQGESDVLDIHDAESYGNNMDRL
IKNLRHDLNLPSLPIIQVAIASGGGYIDKVREAQLGLKLSNVVCVDAKGLPLKSDNLHLTT
EAQVQLGLSLAQAYLSNFC
>non-antioxidant_654
DPLLPGYSFNAHLVAGLTPIEANGYLDFFIDRPLGMKGYILNLTIRGQGVVKNQGREFVCR
PGDILLFPPGEIHHYGRHPEAREWYHQWVYFRPRAYWHEWLNWPSIFANTGFFRPDEAH

QPHFSDLFGQIINAGQGEGRYSELLAINLLEQLLLRRMEAINES
>non-antioxidant_655
GEFSTREGEIVAGVIQRDSRANARGLVVVRIGTETKASEGVIPAAEQVPGESYEHGNRLRC
YVVGVTRGAREPLITLSRTHPNLVRKLFSLEVPEIADGSVEIVAVAREAGHRSKIAVRSNVA
GLNAKGACIGPMGQRVRNVMSELSGEKIDIIDYDDDPARFVANALSPAKVVSVSVIDQTAR
AARVVVPDFQLSLAIGKEGQNARLAARLTGWRIDIRGDAPPPPPGQPEPGVSRGMAHDRL
EHHHHHH
>non-antioxidant_656
AGGPGSRARAAGARGCRLRSQLVPVRALGLGHRSDELVRFRFCSGSCRRARSPHDLSLAS
LLGAGALRPPPGSRPVSQPCCRPTRYEAVSFMDVNSTWRTVDRLSATACGCLG
>non-antioxidant_657
SLLNVPAGKDLPEDIYVVIEIPANADPIKYEIDKESGALFVDQFMSTAMFYPCNYGYINHTL
SLDGDPVDVLVPTPYPLQPGSVTRCRPVGVLKMTDEAGEDAKLVAVPHSKLSKEYDHIKD
VNDLPELLKAQIAHFFEHYKDLEKGKWVKVEGWENAEAAKAEIVASFERAKNK
>non-antioxidant_658
AELHLESRGGSGTQLRDGAKVATGRIICREAHTGFHVWMNERQVDGRAERYVVQSKDGR
HELRVRTGGDGWSPVKGEGGKGVSRPGQEEQVFFDVMADGNQDIAPGEYRFSVGGACV
VPQEKLAAALEHHHHHH
>non-antioxidant_659
SRIVVHTQTLFDIVNDGYRWRKYGQKSVKGSPYPRSYYRCSSPGCPVKKHVERSSHDTKL
LITTYEGKHDHDMPPG
>non-antioxidant_660
EILDLTQTLINFPRPGDPELRIIEKKIDGFIVSEIIMGSHLCTHIDYPKHVGLENRIPFKDGIIK
GKGYCISLDDFPGNKLPACDILLIYTGFSKYWGRDEYFEKIPEIPFLDDIIKSNIKCVGIDAC
TIGGFEEHKRLLSNNILIIENLNENLKNLVGKSFYFLGLPLKIFDIDASPIRCIAILE
>non-antioxidant_661
GHMTWKPNVTVAAVIEQDDKYLLVEEIPRGTAIKLNQPAGHLEPGESIIQACSREVLEETG
HSFLPEVLTGIYHWTCASNGTTYLRFTFSGQVVSFDPDRKLDTGIVRAAWFSIDEIRAKQA
MHRTPLVMQCIEDYHAGKRYPLDILQYYDGS
>non-antioxidant_662
GSHMVGQLSEGAIAAIMQKGDTNIKPILQVINIRPITTGNSPPRYRLLMSDGLNTLSSFMLA
TQLNPLVEEEQLSSNCVCQIHRFIVNTLKDGRRVVILMELEVLKSAEAVGVKIGNPVPYNE
>non-antioxidant_663
SPLPSQAMDDLMLSPDDIEQWFTEDPGP
>non-antioxidant_664
MGSSHHHHHHSSGLVPRGSHMRAQANEDIVVSKTSLWKEMQYNRDITIRFKFASSIVKSG
GLGYKWSEISFKPANYQYTYTRDGEEVTAHTTCSVNGMNDFNFNGGSLPTDFVISRYEVI
KENSYVYVDYWDDSQAFRNMVYVRSLAANLNSVICTGGDYSFALPVGQWPVMTGGAV
SLHSAGVTLSTQFTDFVSLNSLRFRFRLTVEEPSFSITRTRVSRLYGLPAANPNNGKEYYEV
AGRFSLISLVPSNDD
>non-antioxidant_665
MINEIEIKRKFGRTLKKIRTQKGVSQEELADLAGLHRTYISEVERGDRNISLINIHKICAALD
IPASTFFRKMEEEN
>non-antioxidant_666

SPSPLNPGTNVARLAEQAPIHWVSVAQIENSLAGRPPMAVGFDIDDTVLFSSPGFWRGKKT
FSPESEDYLKNPVFWEKMNNGWDEFSIPKEVARQLIDMHVRRGDAIFFVTGRSPTKTETV
SKTLADNFHIPATNMNPVIFAGDKPGQNTKSQWLQDKNIRIFYGDSDNDITAARDVGARGI
RILRASNSTYKPLPQAGAFGEEVIVNSEY
>non-antioxidant_667
AVCPTGLFSNPLCCATNVLDLIGVDCKTPTIAVDTGAIFQAHCASKGSKPLCCVAPVADQA
LLCQKAIGTF
>non-antioxidant_668
MKQPYAVVASCAYCEKLVRLTVLADHSAIRQLEEMLLRSLNIVCPLCTLQRQ
>non-antioxidant_669
GSHMLCAISGKVPRRPVLSPKSRTIFEKSLLEQYVKDTGNDPITNEPLSIEEIVEIVPSAQ
>non-antioxidant_670
MSDSKEVPSLPFLRHLLEELDSHEDSLLLFLCHDAAPGCTTVTQALCSLSQQRKLTLAALV
EMLYVLQRMDLLKSRFGLSKEGAEQLLGTSFLTRYRKLMVCVGEELDSSELRALRLFACN
LNPSLSTALSESSRFVELVLALENVGLVSPSSVSVLADMLRTLRRLDLCQQLVEYEQQEQA
RYRYCLEHHHHHH
>non-antioxidant_671
MTFQASGTTGITTLTVTEECRVQVGNVTATLARSKLKDDTAIGVIGVTALGCNGLQAALQ
ADPDNYDATNLYMTSRNHDKLNVKLKATDGSSWTYGNGVFYKTEGGNWGGHVGISVD
GNQTDKPTGEYTLNLTGGYWTNDNKQTFQASGTTGITTLTLEHHHHHH
>non-antioxidant_672
MRTLMIEPLTKEAFAQFGDVIETDGSDHFMINNGSTMRFHKLATVETAEPEDKAIISIFRAD
AQDMPLTVRMLERHPLGSQAFIPLLGNPFLIVVAPVGDAPVSGLVRAFRSNGRQGVNYHR
GVWHHPVLTIEKRDDFLVVDRSGSGNNCDEHYFTEEQMLILNPHQLEHHHHHH
>non-antioxidant_673
HGYVESPASRAYQCKLQLNTQCGSVQYEPQSVEGLKGFPQAGPADGHIASADKSTFFELD
QQTPTRWNKLNLKTGPNSFTWKLTARHSTTSWRYFITKPNWDASQPLTRASFDLTPFCQF
NDGGAIPAAQVTHQCNIPADRSGSHVILAVWDIADTANAFYQAIDVNLSK
>non-antioxidant_674
VEGAVKTEPVDLFHPGFLNSSNYRIPALFKTKEGTLIASIDARRHGGADAPNNDIDTAVRRS
EDGGKTWDEGQIIMDYPDKSSVIDTTLIQDDETGRIFLLVTHFPSKYGFWNAGLGSGFKNI
DGKEYLCLYDSSGKEFTVRENVVYDKDSNKTEYTTNALGDLFKNGTKIDNINSSTAPLKA
KGTSYINLVYSDDDGKTWSEPQNINFQVKKDWMKFLGIAPGRGIQIKNGEHKGRIVVPVY
YTNEKGKQSSAVIYSDDSGKNWTIGESPNDNRKLENGKIINSKTLSDDAPQLTECQVVEM
PNGQLKLFMRNLSGYLNIATSFDGGATWDETVEKDTNVLEPYCQLSVINYSQKVDGKDA
VIFSNPNARSRSNGTVRIGLINQVGTYENGEPKYEFDWKYNKLVKPGYYAYSCLTELSNG
NIGLLYEGTPSEEMSYIEMNLKYLESG
>non-antioxidant_675
GPSQPTYPGDDAPVEDLIRFYNDLQQYLNVVTRHRM
>non-antioxidant_676
SSLDDKPQFPGASAEFIDKLEFIQPNVISGIPIYRVMDRQGQIINPSEDPHLPKEKVLKLYKS
MTLLNTMDRILYESQRQGRISFYMTNYGEEGTHVGSAAALDNTDLVFGQAREAGVLMYR
DYPLELFMAQCYGNISDLGKGRQMPVHYGCKERHFVTISSPLATQIPQAVGAAYAAKRAN
ANRVVICYFGEGAASEGDAHAGFNFAATLECPIIFFCRNNGYAISTPTSEQYRGDGIAARGP

GYGIMSIRVDGNDVFAVYNATKEARRRAVAENQPFLIEAMTYRIGHASTSDDSSAFRSVDE
VNYWDKQDHPISRLRHYLLSQGWWDEEQEKAWRKQSRRKVMEAFEQAERKPKPNPNL
LFSDVYQEMPAQLRKQQESLARHLQTYGEHYPLDHFDK
>non-antioxidant_677
MTQTQPVTPTPPASFQTQHDPRTRLGATPLPGGAGTRFRLWTSTARTVAVRVNGTEHVMT
SLGGGIYELELPVGPGARYLFVLDGVPTPDPYARFLPDGVHGEAEVVDFGTFDWTDADW
HGIKLADCVFYEVHVGTFTPEGTYRAAAEKLPYLKELGVTAIQVMPLAAFDGQRGWGYD
GAAFYAPYAPYGRPEDLMALVDAAHRLGLGVFLDVVYNHFGPSGNYLSSYAPSYFTDRFS
SAWGMGLDYAEPHMRRYVTGNARMWLRDYHFDGLRLDATPYMTDDSETHILTELAQEI
HELGGTHLLLAEDHRNLPDLVTVNHLDGIWTDDFHHETRVTLTGEQEGYYAGYRGGAEA
LAYTIRRGWRYEGQFWAVKGEEHERGHPSDALEAPNFVYCIQNHDQIGNRPLGERLHQSD
GVTLHEYRGAAALLLTLPMTPLLFQGQEWAASTPFQFFSDHAGELGQAVSEGRKKEFGGF
SGFSGEDVPDPQAEQTFLNSKLNWAEREGGEHARTLRLYRDLLRLRREDPVLHNRQRENL
TTGHDGDVLWVRTVTGAGERVLLWNLGQDTRAVAEVKLPFTVPRRLLLHTEGREDLTLG
AGEAVLVG
>non-antioxidant_678
MADPWQECMDYAVTLAGQAGEVVREALKNEMNIMVKSSPADLVTATDQKVEKMLITSIK
EKYPSHSFIGEESVAAGEKSILTDNPTWIIDPIDGTTNFVHGFPFVAVSIGFVVNKKMEFGIV
YSCLEDKMYTGRKGKGAFCNGQKLQVSHQEDITKSLLVTELGSSRTPETVRIILSNIERLLC
LPIHGIRGVGTAALNMCLVAAGAADAYYEMGIHCWDVAGAGIIVTEAGGVLLDVTGGPF
DLMSRRVIASSNKTLAERIAKEIQIIPLQRDDED
>non-antioxidant_679
EQWQTLYEAIGGEETVAKLVEAFYRRVAAHPDLRPIFPDDLTETAHKQKQFLTQYLGGPPL
YTAEHGHPMLRARHLRFEITPKRAEAWLACMRAAMDEIGLSGPAREQFYHRLVLTAHHM
VNTPDHLD
>non-antioxidant_680
MKVMECQTYEELSQIAARITADTIKEKPDAVLGLATGGTPEGTYRQLIRLHQTENLSFQNI
TTVNLDEYAGLSSDDPNSYHFYMNDRFFQHIDSKPSRHFIPNGNADDLEAECRRYEQLVD
SLGDTDIQLLGIGRNGHIGFNEPGTSFKSRTHVVTLNEQTRQANARYFPSIDSVPKKALTM
GIQTILSSKRILLLISGKSKAEAVRKLLEGNISEDFPASALHLHSDVTVLIDREAASLRP
>non-antioxidant_681
MKKNAKQIVHELYNDISISKDPKYSDILEVLQKVYLKLEKQKYELDPSPLINRLVNYLYFT
AYTNKIRFTEYQEELIRNLSEIGRTAGINGLYRADYGDKSQF
>non-antioxidant_682
MKTVVFAYHDMGCLGIEALLAAGYEISAIFTHTDNPGEKAFYGSVARLAAERGIPVYAPD
NVNHPLWVERIAQLSPDVIFSFYYRHLIYDEILQLAPAGAFNLHGSLLPKYRGRAPLNWVL
VNGETETGVTLHRMVKRADAGAIVAQLRIAIAPDDIAITLHHKLCHAARQLLEQTLPAIKH
GNILEIAQRENEATCFGRRTPDDSFLEWHKPASVLHNMVRAVADPWPGAFSYVGNQKFTV
WSSRVHPHASKAQPGSVISVAPLLIACGDGALEIVTGQAGDGITMQGSQLAQTLGLVQGS
RLN
>non-antioxidant_683
MTQAKAKHADVPVNLYRPNAPFIGKVISNEPLVKEGGIGIVQHIKFDLTGGNLKYIEGQSIG
IIPPGVDKNGKPEKLRLYSIASTRHGDDVDDKTISLCVRQLEYKHPESGETVYGVCSTYLT
HIEPGSEVKITGPVGKEMLLPDDPEANVIMLAGGTGITPMRTYLWRMFKDAERAANPEYQ

FKGFSWLVFGVPTTPNILYKEELEEIQQKYPDNFRLTYAISREQKNPQGGRMYIQDRVAEH
ADQLWQLIKNQKTHTYICGPPPMEEGIDAALSAAAAKEGVTWSDYQKDLKKAGRWHVE
TY
>non-antioxidant_684
SSNNFNYGAYHSLEAIYHEMDNIAADFPDLARRVKIGHSFENRPMYVLKFSTGKGVRRPA
VWLNAGIHSREWISQATAIWTARKIVSDYQRDPAITSILEKMDIFLLPVANPDGYVYTQTQ
NRLWRKTRSRNPGSSCIGADPNRNWNASFAGKGASDNPCSEVYHGPHANSEVEVKSVVD
FIQKHGNFKGFIDLHSYSQLLMYPYGYSVKKAPDAEELDKVARLAAKALASVSGTEYQV
GPTCTTVYPASGSSIDWAYDNGIKFAFTFELRDTGTYGFLLPANQIIPTAEETWLGLKTIME
HVRDNLY
>non-antioxidant_685
NDSPFYVNPNMSSAEWVRNNPNDPRTPVIRDRIASVPQGTWFAHHNPGQITGQVDALMS
AAQAAGKIPILVVSNAPGRDCGNHSSGGAPSHSAYRSWIDEFAAGLKNRPAYIIVEPDLISL
MSSCMQHVQQEVLETMAYAGKALKAGSSQARIYFDAGHSAWHSPAQMASWLQQADISN
SAHGIATNTSNYRWTADEVAYAKAVLSAIGNPSLRAVIDTSRNGNGPAGNEWCDPSGRAIG
TPSTTNTGDPMIDAFLWIKLPGEADGCIAGAGQFVPQAAYEMAIAA
>non-antioxidant_686
MGEVEAPGRLWLESPPGEAPPIFLPSDGQALVLGRGPLTQVTDRKCSRTQVELVADPETRT
VAVKQLGVNPSTTGTQELKPGLEGSLGVGDTLYLVNGLHPLTLRWEETR
>non-antioxidant_687
MRGSHHHHHHRSPSKVQELSVYEINELDRHSPKILKNAFSLMFGLGDLVPFTNKLYTGDL
KKRVGITAGLCVVIEHVPEKKGERFEATYSFYFGDYGHLSVQGPYLTYEDSFLAITGGAGI
FEGAYGQVKLQQLVYPTKLFYTFYLKGLANDLPLELTGTPVPPSKDIEPAPEAKALEPSGVI
SNYTN
>non-antioxidant_688
MAHHHHHHMASPASTNPAHDHFETFVQAQLCQDVLSSFQGLCRALGVESGGGLSQYHKI
KAQLNYWSAKSLWAKLDKRASQPVYQQGQACTNTKCLVVGAGPCGLRAAVELALLGAR
VVLVEKRIKFSRHNVLHLWPFTIHDLRALGAKKFYGRFCTGTLDHISIRQLQLLLLKVALL
LGVEIHWGVKFTGLQPPPRKGSGWRAQLQPNPPAQLASYEFDVLISAAGGKFVPEGFTIRE
MRGKLAIGITANFVNGRTVEETQVPEISGVARIYNQKFFQSLLKATGIDLENIVYYKDETHY
FVMTAKKQCLLRLGVLRQDLSETDQLLGKANVVPEALQRFARAAADFATHGKLGKLEFA
QDARGRPDVAAFDFTSMMRAESSARVQEKHGARLLLGLVGDCLVEPFWPLGTGVARGFL
AAFDAAWMVKRWAEGAGPLEVLAERESLYQLLSQTSPENMHRNVAQYGLDPATRYPNLN
LRAVTPNQVQDLYDMMDKE
>non-antioxidant_689
SSVQTAATSWGTVPSIRVYTANNGKITERCWDGKGWYTGAFNEPGDNVSVTSWLVGSAI
HIRVYASTGTTTTEWCWDGNGWTKGAYTSTN
>non-antioxidant_690
FCLTLRRRYTMGHHHHHHHHHHSSGHIEGRHMKLEQTLTLSPNLIGFNSNEGEKLLLTSRS
REDFFPLSMQFVTQVNQAYCGVASIIMVLNSLGINAPETAQYSPYRVFTQDNFFSNEKTKA
VIAPEVVARQGMTLDELGRLIASYGVKVKVNHASDTNIEDFRKQVAENLKQDGNFVIVN
YLRKEIGQERGGHISPLAAYNEQTDRFLIMDVSRYKYPPVWVKTTDLWKAMNTVDSVSQ
KTRGFVFVSKTQDD
>non-antioxidant_691

IVEGSDAEIGMSPWQVMLFRKSPQELLCGASLISDRWVLTAAHCLLYPPWDKNFTENDLL
VRIGKHSRTRYERNIEKISMLEKIYIHPRYNWRENLDRDIALMKLKKPVAFSDYIHPVCLPD
RETAASLLQAGYKGRVTGWGNLKEKGQPSVLQVVNLPIVERPVCKDSTRIRITDNMFCAG
YKPDEGKRGDACEGDSGGPFVMKSPFNNRWYQMGIVSWGEGCDRDGKYGFYTHVFRL
KKWIQKVIDQFGE
>non-antioxidant_692
LDMSLNIHIKSGQDKWEVNVAPESTVLQFKEAINKANGIPVANQRLIYSGKILKDDQTVES
YHIQDGHSVHLVKSQP
>non-antioxidant_693
MQLKRVAEAKLPTPWGDFLMVGFEELATGHDHVALVYGDISGHTPVLARVHSECLTGDA
LFSLRCDCGFQLEAALTQIAEEGRGILLYHRQEGRNIGLLNKIRAYALQDQGYDTVEANHQ
LGFAADERDFTLCADMFKLLGVNEVRLLTNNPKKVEILTEAGINIVERVPLIVGRNPNNEH
YLDTKAEKMGHLLNK
>non-antioxidant_694
RIAISNSNRTRSVPSLTTIWSISPTPNCSIYETQDANLFLCLTKNGAHVLGTITIKGLKGALRE
MHDNALSLKLPFDNQGNLLNCALESSTWRYQETNAVASNALTFMPNSTVYPRNKTAHPG
NMLIQISPNITFSVVYNEINSGYAFTFKWSAEPGKPFHPPTAVFCYITEQGSHHHHHH
>non-antioxidant_695
ETEAIDNGALREEAKGVFEAIPEKMTAIKQTEDNPEGVPLTAEKIELGKVLFFDPRMSSSGL
ISCQTCHNVGLGGVDGLPTSIGHGWQKGPRNAPTMLNAIFNAAQFWDGRAADLAEQAK
GPVQAGVEMSNTPDQVVKTINSMPEYVEAFKAAFPEEADPVTFDNFAAAIEQFEATLITPN
SAFDRFLAGDDAAMTDQEKRGLQAFMETGCTACHYGVNFGGQDYHPFGLIAKPGAEVL
PAGDTGRFEVTRTTDDEYVFRAAPLRNVALTAPYFHSGVVWELAEAVKIMSSAQIGTELTD
QQAEDITAFLGTLTGEQPVIDHPILPVRTGTTPLPTPM
>non-antioxidant_696
MTKKSTKSEAASKTKKSGVPETGAQGVRAGGADHADAAHLGTVNNALVNHHYLEEKEF
QTVAETLQRNLATTISLYLKFKKYHWDIRGRFFRDLHLAYDEFIAEIFPSIDEQAERLVALG
GSPLAAPADLARYSTVQVPQETVRDARTQVADLVQDLSRVGKGYRDDSQACDEANDPVT
ADMYNGYAATIDKIRWMLQAIMDDERLD
>non-antioxidant_697
GSHMASGDATDITIYYKTGWTHPHIHYSLNQGAWTTLPGVPLTKSEYEGYVKVTIEAEEG
SQLRAAFNNGSGQWDNNQGRDYDFSSGVHTLADGRILSGTPK
>non-antioxidant_698
MKHHHHHHPMVKEYKTITQIAGPLIFVEKTEPVGYNEIVNIKMGDGTVRRGQVLDSSADI
VVVQVFEGTGGLDKDCGVIFTGETLKLPASVDLLGRILSGSGEPRDGGPRIVPDQLLDING
AAMNPYARLPPKDFIQTGISTIDGTNTLVRGQKLPIFSASGLPHNEIALQIARQASVPGSESA
FAVVFAAMGITNEEAQYFMSDFEKTGALERAVVFLNLADDPAVERIVTPRMALTAAEYLA
YEHGMHVLVILTDITNYAEALRQMGAARNEVPGRRGYPGYMYTDLATLYERAGIVKGAK
GSVTQIPILSMPGDDITHPIPDLSGYITEGQIVVARELHRKGIYPPINVLPSLSRLMNSGIGAG
KTREDHKAVSDQMYAGYAEGRDLRGLVAIVGKEALSERDTKFLEFADLFEDKFVRQGRN
ENRTIEDTLEIGWQILTHLPENQLGRIDNKYIQKYHPAHRKAK
>non-antioxidant_699
MPANKLVALTFDDGPDNVLTARVLDKLDKYNVKATFMVVGQRVNDSTAAIIRRMVNSGH
EIGNHSWSYSGMANMSPDQIRKSIADTNAVIQKYAGTTPKFFRPPNLETSPTLFNNVDLVF

VGGLTANDWIPSTTAEQRAAAVINGVRDGTIILLHDVQPEPHPTPEALDIIIPTLKSRGYEFV
TLTELFTLKGVPIDPSVKRMYNSVPLEHHHHHH
>non-antioxidant_700
AKGEFVRTKPHVNVGTIGHVDHGKTTLTAALTYVAAAENPNVEVKDYGDIDKAPEERAR
GITINTAHVEYETAKRHYSHVDCPGHADYIKNMITGAAQMDGAILVVSAADGPMPQTRE
HILLARQVGVPYIVVFMNKVDMVDDPELLDLVEMEVRDLLNQYEFPGDEVPVIRGSALL
ALEQMHRNPKTRRGENEWVDKIWELLDAIDEYIPTPVRDVDKPFLMPVEDVFTITGRGTV
ATGRIERGKVKVGDEVEIVGLAPETRKTVVTGVEMHRKTLQEGIAGDNVGVLLRGVSRE
EVERGQVLAKPGSITPHTKFEASVYVLKKEEGGRHTGFFSGYRPQFYFRTTDVTGVVQLP
PGVEMVMPGDNVTFTVELIKPVALEEGLRFAIREGGRTVGAGVVTKILE
>non-antioxidant_701
MKGGAGVPDLPSLDASGVRLAIVASSWHGKICDALLDGARKVAAGCGLDDPTVVRVLGA
IEIPVVAQELARNHDAVVALGVVIRGQTPHFDYVCDAVTQGLTRVSLDSSTPIANGVLTTNT
EEQALDRAGLPTSAEDKGAQATVAALATALTLRELRAHS
>non-antioxidant_702
GSMAASLVGKKIVFVTGNAKKLEEVVQILGDKFPCTLVAQKIDLPEYQGEPDEISIQKCQE
AVRQVQGPVLVEDTCLCFNALGGLPGPYIKWFLEKLKPEGLHQLLAGFEDKSAYALCTFA
LSTGDPSQPVRLFRGRTSGRIVAPRGCQDFGWDPCFQPDGYEQTYAEMPKAEKNAVSHRF
RALLELQEYFGSLAA
>non-antioxidant_703
MSQAEFEKAAEEVRHLKTKPSDEEMLFIYGHYKQATVGDINTERPGMLDFTGKAKWDA
WNELKGTSKEDAMKAYINKVEELKKKYGI
>non-antioxidant_704
MNSITVRNATFTWARSDPPTLNGITFSIPEGALVAVVGQVGCGKSSLLSALLAEMDKVEGH
VAIKGSVAYVPQQAWIQNDSLRENILFGCQLEEPYYRSVIQACALLPDLEILPSGDRTEIGE
KGVNLSGGQKQRVSLARAVYSNADIYLFDDPLSAVDAHVGKHIFENVIGPKGMLKNKTRI
LVTHSMSYLPQVDVIIVMSGGKISEMGSYQELLARDGAFAEFLRTYASHHHHHH
>non-antioxidant_705
MVFKKVLLTGTSEESFTAAADDAIDRAEDTLDNVVWAEVVDQGVEIGAVEERTYQTEVQ
VAFELDGSQ
>non-antioxidant_706
GSASPAVAELCQNTPETFLEASKLLLTYADNILRNPNDEKYRSIRIGNTAFSTRLLPVRGAVE
CLFEMGFEEGETHLIFPKKASVEQLQKIRDLIAIER
>non-antioxidant_707
RVQSGKIDCGDDAGWAKVPSDDPGRDNTRELAKNITFASPYCRPPVVLLSITQLDVEQSQ
NLRVIARLYSVSPSGFKASCYTWHNTKVYSMSISWISIENY
>non-antioxidant_708
AQCEATVESNDAMQYNVKEIVVDKSCKQFTMHLKHVGKMAKVAMGHNLVLTKDADKQ
AVATDGMGAGLAQDYVKAGDTRVIAHTKVIGGGESDSVTFDVSKIAAGENYAYFCSFPGH
WAMMKGTLKLGS
>non-antioxidant_709
MTEYKLVVVGAGGVGKSALTIQLIQNHFVDECDPTIEDSYRKQVVIDGETCLLDILDTAGQ
EEYSAMRDQYMRTGEGFLCVFAINNTKSFEDIHQYREQIKRVKDSDDVPMVLVGNKSDL
AARTVESRQAQDLARSYGIPYIETSAKTRQGVEDAFYTLVREIRQH

>non-antioxidant_710
AEETCFDKYTGNTYRVGDTYERPKDSMIWDCTCIGAGRGRISCTIANRCHEGGQSYKIGD
TWRRPHETGGYMLECVCLGNGKGEWTCKPI
>non-antioxidant_711
MAQQGVFTLPANTSFGVTAFANAANTQTIQVLVDNVVKATFTGSGTSDKLLGSQVLNSGS
GAIKIQVSVNGKPSDLVSNQTILANKLNFAMVGSEDGTDNDYNDGIAVLNWPLG
>non-antioxidant_712
ATFGRATHVVVRALPESLAQQALRRTKGDEVDFARAERQHQLYVGVLGSKLGLQVVQLP
ADESLPDCVFVEDVAVVCEETALITRPGAPSRRKEADMMKEALEKLQLNIVEMKDENATL
DGGDVLFTGREFFVGLSKRTNQRGAEILADTFKDYAVSTVPVVDALHLKSFCSMAGPNLI
AIGSSESAQKALKIMQQMSDHRYDKLTVPDDTAANCIYLNIPSKGHVLLHRTPEEYPESAK
VYEKLKDHMLIPVSNSELEKVDGLLTMSSVLINKK
>non-antioxidant_713
IPEYVDWRQKGAVTPVKNQGSCGSCWAFSAVVTIEGIIKIRTGNLNQYSEQELLDCDRRSY
GCNGGYPWSALQLVAQYGIHYRNTYPYEGVQRYCRSREKGPYAAKTDGVRQVQPYNEG
ALLYSIANQPVSVVLEAAGKDFQLYRGGIFVGPCGNKVDHAVAAVGYGPNYILIKNSWGT
GWGENGYIRIKRGTGNSYGVCGLYTSSFYPVKN
>non-antioxidant_714
GAMGSGDTQLFNRAVSMVEKNKDIRSLLQCDDGITGKERLKAYGELITNDKWTRNRPIVS
TKKLDKEGRTHHYMRFHVESKKKIALVHLEAKESKQNYQPDFINMYVDVPGEKRYYLIK
PKLHPVSN
>non-antioxidant_715
EEPGSGIGYPYDNNTLPYVAPGPTDSRAPCPALNALANHGYIPHDGRAISRETLQNAFLNH
MGIANSVIELALTNAFVVCEYVTGSDCGDSLVNLTLLAEPHAFEHDHSFSRKDYKQGVAN
SNDFIDNRNFDAETFQTSLDVVAGKTHFDYADMNEIRLQRESLSNELDFPGWFTESKPIQN
VESGFIFALVSDFNLPDNDENPLVRIDWWKYWFTNESFPYHLGWHPPSPAREIEFVTSASSA
VLAASVTSTPSSLPSGAIGPGAEAVPLSFASTMTPFLLATNAPYYAQDPTLGPND
>non-antioxidant_716
GEEEKKRTARTDYWLQPEIIVKIITKKLGEKYHKKKAIVKEVIDKYTAVVKMIDSGDKLKL
DQTHLETVIPAPGKRILVLNGGYRGNEGTLESINEKTFSATIVIETGPLKGRRVEGIQYEDIS
KLA
>non-antioxidant_717
GSHMKEPKLSPKQERFIEEYFINDMNATKAAIAAGYSKNSASAIGAENLQKPAIRARIDAR
LK
>non-antioxidant_718
MSITKTELDGILPLVARGKVRDIYEVDAGTLLFVATDRISAYDVIMENSIPEKGILLTKLSEF
WFKFLSNDVRNHLVDIAPGKTIFDYLPAKLSEPKYKTQLEDRSLLVHKHKLIPLEVIVRGYI
TGSAWKEYVKTGTVHGLKQPQGLKESQEFPEPIFTPSTKAEQGEHDENISPAQAAELVGED
LSRRVAELAVKLYSKCKDYAKEKGIIIADTKFEFGIDEKTNEIILVDEVLTPDSSRFWNGASY
KVGESQDSYDKQFLRDWLTANKLNGVNGVKMPQDIVDRTRAKYIEAYETLTGSKWSH
>non-antioxidant_719
MGTEPPENCQDDFNFNYVSDQEIEVYHVDKGWSAGWNYVCLNDYCLPGNKSNGAFRKT
FNAVLGQDYKLTFKVEDRYGQGQQILDRNITFTTQVCNLEHHHHHH
>non-antioxidant_720

MKKRLTITLSESVLENLEKMAREMGLSKSAMISVALENYKKGQER
>non-antioxidant_721
QGRYTTDDGYIFNASDIIEDTGDAYIVPHGDHYHYIPKNELSASELAAAEAFLSG
>non-antioxidant_722
MSLTLADKVVYEEEIQKSRFIAKAAPVASEEEALAFLAENREPEATHNGHAYKIGLLYRFS
DDGEPSGTAGRPILHAIEAQGLDRVAVLVVRYFGGVKLGAGGLVRAYGGVAAEALRRAPK
VPLVERVGLAFLVPFAEVGRVYALLEARALKAEETYTPEGVRFALLLPKPEREGFLRALLD
ATRGQVALE
>non-antioxidant_723
MPAAAPGKNFDLSHWKLQLPDANTTEISSANLGLGYTSQYFYTDTDGAMTFWAPTTGGT
TANSSYPRSELREMLDPSNSKVNWGWQGTHTMKLSGKTVQLPSSGKIIVAQIHGIMDDGT
NAPPLVKAVFQDGQLDMQVKQNSDGTGSDVHNYFTGIKLGDLYNMEIRVTDGVAYVTM
NGDTRSVDFVGKDAGWKNLKYYFKAGNYVQDNTSTGGSAIAKLYSLSVSHSNLEHHHH
HH
>non-antioxidant_724
MRGSHHHHHHTDPALRAMRLVQLSRHSIAFPSPEGALREPNGLLALGGDLSPARLLMAYQ
RGIFPWFSPGDPILWWSPDPRAVLWPESLHISRSMKRFHKRSPYRVTMNYAFGQVIEGCAS
DREEGTWITRGVVEAYHRLHELGHAHSIEVWREDELVGGMYGVAQGTLFCGESMFSRME
NASKTALLVFCEEFIGHGGKLIDCQVLNDHTASLGACEIPRRDYLNYLNQMRLGRLPNNF
WVPRCLFSPQEGLCGR
>non-antioxidant_725
MAALTRNPQFQKLLEWHRANSANLKLRELFEADPERFNNFSLNLNTNHGHILVDYSKNL
VSKEVMQMLVELAKSRGVEAARDNMFSGSKINYTEDRAVLHVALRNRSNTPIKVDGKDV
MPEVNRVLDKMKSFCQRVRSGDWKGYTGKSITDIINIGIGGSDLGPLMVTEALKPYSKGG
PRVWFVSNIDGTHIAKTLASLSPETSLFIIASKTFTTQETITNAETAKEWFLEAAKDPSAVAK
HFVALSTNTAKVKEFGIDPQNMFEFWDWVGGRYSLWSAIGLSIALHVGFDHFEQLLSGAH
WMDQHFLKTPLEKNAPVLLALLGIWYINCYGCETHALLPYDQYMHRFAAYFQQGDMES
NGKYITKSGARVDHQTGPIVWGEPGTNGQHAFYQLIHQGTKMIPCDFLIPVQTQHPIRKGL
HHKILLANFLAQTEALMKGKLPEEARKELQAAGKSPEDLEKLLPHKVFEGNRPTNSIVFT
KLTPFILGALIAMYEHKIFVQGIMWDINSFDQWGVELGKQLAKKIEPELEGSSAVTSHDSS
TNGLISFIKQQRDTKL
>non-antioxidant_726
GSSGSSGEKITKVYELGNEPERKLWVDRYLTFMEERGSPVSSLPAVGKKPLDLFRLYVCVK
EIGGLAQVNKNKKWRELATNLNVGTSSSAASSLKKQYIQYLFAFECKIERGEEPPPEVFST
GDT
>non-antioxidant_727
IGVCYGMLGNNLPPPSEVVSLYKSNNIARMRLYDPNQAALQALRNSNIQVLLDVPRSDVQ
SLASNPSAAGDWIRRNVVAYWPSVSFRYIAVGNELIPGSDLAQYILPAMRNIYNALSSAGL
QNQIKVSTAVDTGVLGTSYPPSAGAFSSAAQAYLSPIVQFLASNGAPLLVNVYPYFSYTGN
PGQISLPYALFTASGVVVQDGRFSYQNLFDAIVDAVFAALERVGGANVAVVVSESGWPSA
GGGAEASTSNAQTYNQNLIRHVGGGTPRRPGKEIEAYIFEMFNENQKAGGIEQNFGLFYP
NKQPVYQISF
>non-antioxidant_728
MKIEEVRFGLVKIDGKEFDHDIVIYPSGRIERRMKEISKKKHGTSHKLDPEELEKYLVEDFD

VLLVGTGIYGMLSLLPESKKLVEDKEVIEKPTKEALKLLEELWGKKRILAIIHVTC
>non-antioxidant_729
MEALRLGFSPCPNDTFIFYALVHGRVESPVPLEPVLEDVETLNRWALEGRLPLTKLSYAAYA
QVRDRYVALRSGGALGRGVGPLVVARGPLQALEGLRVAVPGRHTTAYFLLSLYAQGFVPV
EVRYDRILPMVAQGEVEAGLIIHESRFTYPRYGLVQVVDLGAWWEERTGLPLPLGAILARR
DLGEGLIRALDEAVRRSVAYALAHPEEALDYMRAHAQELSDEVIWAHVHTYVNAFSLDV
GEEGERAVARLFAEAEARGLAAPSPRPLFV
>non-antioxidant_730
MVSGEVRTKKVPLDTNHKRFYDAFAQGAGKLDLDRQCVECHHEKPGGIPFPKNHPVKPA
DGPMRCLFCHKFKLEHHHHHH
>non-antioxidant_731
MENMENDENIVVGPKPFYPIEEGSAGTQLRKYMERYAKLGAIAFTNAVTGVDYSYAEYLE
KSCCLGKALQNYGLVVDGRIALCSENCEEFFIPVIAGLFIGVGVAPTNEIYTLRELVHSLGIS
KPTIVFSSKKGLDKVITVQKTVTTIKTIVILDSKVDYRGYQCLDTFIKRNTPPGFQASSFKT
VEVDRKEQVALIMNSSGSTGLPKGVQLTHENIVTRFSHARDPIYGNQVSPGTAVLTVVPFH
HGFGMFTTLGYLICGFRVVMLTKFDEETFLKTLQDYKCTSVILVPTLFAILNKSELLNKYD
LSNLVEIASGGAPLSKEVGEAVARRFNLPGVRQGYGLTETTSAIIITPEGDDKPGASGKVVP
LFKAKVIDLDTKKSLGPNRRGEVCVKGPMLMKGYVNNPEATKELIDEEGWLHTGDIGYY
DEEKHFFIVDRLKSLIKYKGYQVPPAELESVLLQHPSIFDAGVAGVPDPVAGELPGAVVVL
ESGKNMTEKEVMDYVASQVSNAKRLRGGVRFVDEVPKGLTGKIDGRAIREILKKPVAKM
>non-antioxidant_732
LSSNSSMNPKSLTDPKLLKNIPMWLKSLRLHKYSDALSGTPWIELIYLDDETLEKKGVLAL
GARRKLLKAFGIVIDYKERDLIDRSAY
>non-antioxidant_733
MKKSLGARTLAYPTPLFLVGTYDRDSRPNIMAAAWAGICCSQPPSIAVSLRKATYTYRSITE
RGAFTISIPSRAYVRHADYAGIYSGENEDKFASLGLTPVPGEHVDAPYVGEFPMAIELKLIH
QIEIGLHTQFIGEIMDVKVDESCLRDDGLPDINKVDPVIFAPVSREYYAVGEFLAKAFSAGK
GLRS
>non-antioxidant_734
MGPQDNSLVIGASQEPRVLAGDFLRVISNQAIKSEIEQYLFAPFIGFNADSQNFPVLATEVPT
LENGRLRVTDIGGGKKRLEMDITIRPDAKWSDGRPITTEDVAFYFEVGKAKGMPVLNPDF
WERVNVRIKDARNFTLIFEPAYYYDTYGPINTYAPKHIMGPEWERVKAAARGLDPDKDAE
KLNELYRNFFLKFATPQALNRGAMVYSGPFKLKRWVPGNSIEMERNPNFPIKPEGGESKY
VQKVVYRFIQNTNSLLVAVIGGSIDATSSVSLTFDQGRSPQLVRRAPGRFDIWFVPGAIWEH
IDINKFENCQVVKDLGLNDKRTRQAILHALNREGLVKAFFDGLQPVAHTWIAPVNPLFNP
NVKKYEFDLKKAEALLAEMGWRKGPDGILQRTVNGRTVRFEIEYVTTAGNVVRERTQQF
FAEDLKKIGIAVKINNAPSAVVFADEFIQRASECKWTGMFEFAWVSNLQEDGSLFQYKNLN
TGAIMVPTKENNYQGQNIGGWRNDEFDRLTSQAVLEFDPERRKQLFWRAQEIWAEELPAL
PLYFRANPYVVRKGLVNYVASAYSGGYGYPGWNAWEIGWESRGAVKKWDQAKYALSTR
>non-antioxidant_735
KTPLVNESLKKFLNTKDGRLVASLVAEFLQFFNLDFTLAVFQPETSTLQGLEGRENLARDL
GIIEAEGTVGGPLLLEVIRRW
>non-antioxidant_736
MDERIQALRKEVDRVNREILRLLSERGRLVQEIGRLQTELGLPHYDPKREEEMLAYLTAEN

PGPFPDETIRKLFKEIFKASLDLEERQDQ
>non-antioxidant_737
MNARSTGQHPARYPGAAAGEPTLDSWQEPPHNRWAFAHLGEMVPSAAVSRRPVNAPGH
ALARLGAIAAQLPDLEQRLEQTYTDAFLVLRGTEVVAEYYRAGFAPDDRHLLMAVSKSLC
GTVVGALVDEGRIDPAQPVTEYVPELAGSVYDGPSVLQVLDMQISIDYNEDYVDPASEVQ
THDRSAGWRTRRHGDPADTYEFLTTLRGDGSTGEFQYCSANTDVLAWIVERVTGLRYVE
ALSTYLWAKLDADRDATITVDTTGFGFANGGVSCTARDLARVGRMMLDGGVAPGGRVVS
EDWVRRVLAGGSHEAMTDKGFTNTFPDGSYTRQWWCTGNERGNVSGIGIHGQNLWLDP
LTDSVIVKLSSWPDPDTEHWHRLQNGILLDVSRALDAV
>non-antioxidant_738
EVSTTQNDTLKVMTHNVYMLSTNLYPNWGQTERADLIGAADYIKNQDVVILNEVFDNSA
SDRLLGNLKKEYPNQTAVLGRSSGSEWDKTLGNYSSSTPEDGGVAIVSKWPIAEKIQYVFA
KGCGPDNLSNKGFVYTKIKKNDRFVHVIGTHLQAEDSMCGKTSPASVRTNQLKEIQDFIK
NKNIPNNEYVLIGGDMNVNKINAENNNDSEYASMFKTLNASVPSYTGHTATWDATTNSIA
KYNFPDSPAEYLDYIIASKDHANPSYIENKVLQPKSPQWTVTSWFQKYTYNDYSDHYPVE
ATISMK
>non-antioxidant_739
LDGVLVPESGILVSVGQDVDSVNDYASALGTIPAGVTNYVGIVNLDGLNSDADAGAGRN
NIAELANAYPTSALVVGVSMNGEVDAVASGRYNANIDTLLNTLAGYDRPVYLRWAYEVD
GPWNGHSPSGIVTSFQYVHDRIIALGHQAKISLVWQVASYCPTPGGQLDQWWPGSEYVD
WVGLSYFAPQDCNWDRVNEAAQFARSKGKPLFLNESTPQRYQVADLTYSADPAKGTNRQ
SKTSQQLWDEWFAPYFQFMSDNSDIVKGFTYINADWDSQWRWAAPYNEGYWGDSRVQA
NALIKSNWQQEIAKGQYINHSETLFETLGYGSTHHHHHH
>non-antioxidant_740
MTSRVDPANPGSELDSAIRDTLTYSNSPVPNALLTASESGFLDAAGIELDVLSGQQGTVHFT
YDQPAYTRFGGEIPPLLSEGLRAPGRTRLLGITPLLGRQGFFVRDDSPITAAADLAGRRIGV
SASAIRILRGQLGDYLELDPWRQTLVALGSWEARALLHTLEHGELGVDDVELVPISSPGVD
VPAEQLEESATVKGADLFPDVARGQAAVLASGDVDALYSWLPWAGELQATGARPVVDLG
LDERNAYASVWTVSSGLVRQRPGLVQRLVDAAVDAGLWARDHSDAVTSLHAANLGVSTG
AVGQGFGADFQQRLVPRLDHDALALLERTQQFLLTNNLLQEPVALDQWAAPEFLNNSLNR
HR
>non-antioxidant_741
MIKVKSPGRVNLIGEHTDYTYGYVMPMAINLYTKIEAEKHGEVILYSEHFGEERKFSLNDL
RKENSWIDYVKGIFWVLKESDYEVGGIKGRVSGNLPLGAGLSSSASFEVGILETLDKLYNL
KLDSLSKVLLAKKAENEFVGVPCGILDQFAVVFGREGNVIFLDTHTLDYEYIPFPKDVSILV
FYTGVRRELASSEYAERKHIAEESLKILGKGSSKEVREGELSKLPPLHRKFFGYIVRENARV
LEVRDALKEGNVEEVGKILTTAHWDLAKNYEVSCKELDFFVERALKLGAYGARLTGAGF
GGSAIALVDKEDAETIGEEILREYLKRFPWKARHFIVEPSDGVGI
>non-antioxidant_742
AAPPAPPVSYGVEEDVFHPVRAKQGMVASVDATATQVGVDILKEGGNAVDAAVAVGYAL
AVTHPQAGNLGGGGFMLIRSKNGNTTAIDFREMAPAKATRDMFLDDQGNPDSKKSLTSHL
ASGTPGTVAGFSLALDKYGTMPLNKVVQPAFKLARDGFIVNDALADDLKTYGSEVLPNH
ENSKAIFWKEGEPLKKGDTLVQANLAKSLEMIAENGPDEFYKGTIAEQIAQEMQKNGGLI
TKEDLAAYKAVERTPISGDYRGYQVYSMPPPSSGGIHIVQILNILENFDMKKYGFGSADAM

QIMAEAEKYAYADRSEYLGDPDFVKVPWQALTNKAYAKSIADQIDINKAKPSSEIRPGKLA
PYESNQ
>non-antioxidant_743
MGHHHHHHMPEINTNHLDKQQVQLLAEMCILIDENDNKIGAETKKNCHLNENIEKGLLH
RAFSVFLFNTENKLLLQQRSDAKITFPGCFTNTCCSHPLSNPAELEESDALGVRRAAQRRL
KAELGIPLEEVPPEEINYLTRIHYKAQSDGIWGEHEIDYILLVRMNVTLNPDPNEIKSYCYV
SKEELKELLKKAASGEIKITPWFKIIAATFLFKWWDNLNHLNQFVDHEKIYRM
>non-antioxidant_744
MVSTLKRDEALFELIALEEKRQREGLELIASENFVSKQVREAVGSVLTNKYAEGYPGARY
YGGCEVIDRVESLAIERAKALFGAAWANVQPHSGSQANMAVYMALMEPGDTLMGMDLA
AGGHLTHGSRVNFSGKLYKVVSYGVRPDTELIDLEEVRRLALEHRPKVIVAGASAYPRFW
DFKAFREIADEVGAYLVVDMAHFAGLVAAGLHPNPLPYAHVVTSTTHKTLRGPRGGLILS
NDPELGKRIDKLIFPGIQGGPLEHVIAGKAVAFFEALQPEFKEYSRLVVENAKRLAEELARR
GYRIVTGGTDNHLFLVDLRPKGLTGKEAEERLDAVGITVNKNAIPFDPKPPRVTSGIRIGTP
AITTRGFTPEEMPLVAELIDRALLEGPSEALREEVRRLALAHPMP
>non-antioxidant_745
SGISLDNSYKMDYPEMGLCIIINNKNFHKSTGMTSRSGTDVDAANLRETFRNLKYEVRNK
NDLTREEIVELMRDVSKEDHSKRSSFVCVLLSHGEEGIIFGTNGPVDLKKITNFFRGDRCRS
LTGKPKLFIIQACRGTELDCGIET
>non-antioxidant_746
ASGVDDDMACHKIPVEADFLYAYSTAPGYYSWRNSKDGSWFIQSLCAMLKQYADKLEFM
HILTRVNRKVATEFESFSFDATFHAKKQIPCIVSMLTKELYFYH
>non-antioxidant_747
MAGYLNNIALNLEIVLKNKADSPEVSETLVTRICENLLLSKEVSFLKADGSVENFKLSDME
YEITNTEELPELEHHHHHH
>non-antioxidant_748
MDNVLLSGQTLHADHSLQAGAYTLTIQNKCNLVKYQNGRQIWASNTDRRGSGCRLTLLS
DGNLVIYDHNNNDVWGSACWGDNGKYALVLQKDGRFVIYGPVLWSLGPNGCRRVNG
>non-antioxidant_749
SFAPGMVSQKCLLCMCKLESGGCKPIGCRMDVGSLSCGYFQIKQPYWIDCGKPGKDWKS
CSNDINCSSKCVQQYMKRYATHYRCPLNCEGFAREHNGGPNGCHSSRTLKYWELLQKIPG
CKGVK
>non-antioxidant_750
PPGPPGPPGPPGLPGPPGPPGPPGPPGPPG
>non-antioxidant_751
TDKRKDGSGKLLYCSFCGKSQHEVRKLIAGPSVYICDECVDLCNDIIREEI
>non-antioxidant_752
MDIYVCTVCGYEYDPAKGDPDSGIKPGTKFEDLPDDWACPVCGASKDAFEKQ
>non-antioxidant_753
MRITLVTDSTSDLPQDLRGRLGVRVVPLYVNLSGAIYRDWEEITPTEIFQKVREGAAFPTTS
QPSPEDFARVYREALEEADHVLSLHISGKLSGTVQSAELAAQEFPGRVTVVDTQAASLGV
GMMVLRAKELLEEGQSLEAVLAELERLRRDHFVRFSVATLEFLKRGGRIGGAQAFLGTLL
NLKPVLTLKEGRVEAAGRARGEKKAREEILKAFRAWAEGRKRIRAYFLYSGDEDAVAALR
QEVLASGLPVEEALVNELGAVIASHTGPGTYGFYAYSL

>non-antioxidant_754
MEEAVLTGVATDKSEAKVTVLGISDKPGEAAKVFRALADAEINIDMVLQNVSSVEDGTTD
ITFTCPRSDGRRAMEILKKLQVQGNWTNVLYDDQVGKVSLVGAGMKSHPGVTAEFMEA
LRDVNVNIELISTSEIRISVLIREDDLDAAARALHEQFQLGGEDEAVVYAGTGRHHHHHH
>non-antioxidant_755
MSVCHKHSVIGVLDSGVGGLTVASEIIRQLPKESICYIGDNERCPYGPRSVEEVQSFVFEMV
EFLKQFPLKALVVACNTAAAATLAALQEALSIPVIGVIHPGARAAIKVTKKGKIGVIGTVGT
IQSNMYEKALHELDTYLKVHSHACPTLATVVENRLEDTAYVTQQVKQALLPLTKEDIDTL
ILGCTHYPLLESYIKKELGEDVTIISSAEETAIELSTILQHKGILADNLNPKHRFFTTGSVSSF
EHIAERWLGYQISVDCVDLPVKNARICN
>non-antioxidant_756
GSSGSSGMTPAVKLLEKNKISFQIHTYEHDPAETNFGDEVVKKLGLNPDQVYKTLLVAVN
GDMKHLAVAVTPVAGQLDLKKVAKALGAKKVEMADPMVAQRSTGYLVGGISPLGQKKR
LPTIIDAPAQEFATIYVSGGKRGLDIELAAGDLAKILDAKFADIARRD
>non-antioxidant_757
MLGLKTSIIGRRVIYFQEITSTNEFAKTSYLEEGTVIVADKQTMGHGALNRKWESPEGGLW
LSIVLSPKVPQKDLPKIVFLGAVGVVETLKEFSIDGRIKWPNDVLVNYKKIAGVLVEGKGD
KIVLGIGLNVNNKVPNGATSMKLELGSEVPLLSVFRSLITNLDRLYLNFLKNPMDILNLVR
DNMILGVRVKILGDGSFEGIAEDIDDFGRLIIRLDSGEVKKVIYGDVSLRFL
>non-antioxidant_758
GSSGSSGMTATAQQLEYLKNSIKSIQDYPKPGILFRDVTSLLEDPKAYALSIDLLVERYKNA
GITKVVGTEARGFLFGAPVALGLGVGFVPVRKPGKLPRETISETYDLEYGTDQLEIHVDAI
KPGDKVLVVDDLLATGGTIEATVKLIRRLGGEVADAAFIINLFDLGGEQRLEKQGITSYSLV
PFPGH
>non-antioxidant_759
ELKIGDRVLVGGTKAGVVRFLGETDFAKGEWCGVELDEPLGKNDGAVAGTRYFQCQPKY
GLFAPVHKVTKIGFPSTTPAKAKANAVRRVM
>non-antioxidant_760
SNSLHWPTSLPSGDAFSSVGTHRFVQKVEEMVQNHMTYSLQDVGGDANWQLVVEEGEM
KVYRREVEENGIVLDPLKATHAVKGVTGHEVCNYFWNVDVRNDWETTIENFHVVETLA
DNAIIIYQTHKRVWPASQRDVLYLSVIRKIPALTENDPETWIVCNFSVDHDSAPLNNRCVRA
KINVAMICQTLVSPPEGNQEISRDNILCKITYVANVNPGGWAPASVLRAVAKREYPKFLKRF
TSYVQEKTAGKPILF

>non-antioxidant_761
GSRSEPAKVVDIRIDTSAERKPISPYIYGSNQELDATVTAKRFGGNRTTGYNWENNFSNAG
SDWLHYSDTYLLEDGGVPKGEWSTPASVVTTFHDKALSKNVPYTLITLQAAGYVSADGN
GPVSQEETAPSSRWKEVKFEKGAPFSLTPDTEDDYVYMDEFVNYLVNKYGNASTPTGIKG
YSIDNEPALWSHTHPRIHPDNVTAKELIEKSVALSKAVKKVDPYAEIFGPALYGFAAYETLQ
SAPDWGTEGEGYRWFIDYYLDKMKKASDEEGKRLLDVLDVHWYPEARGGGERICFGAD
PRNIETNKARLQAPRTLWDPTYIEDSWIGQWKKDFLPILPNLLDSIEKYYPGTKLAITEYDY
GGGNHITGGIAQADVLGIFGKYGVYLATFWGDASNNYTEAGINLYTNYDGKGGKFGDTS
VKCETSDIEVSSAYASIVGEDDSKLHIILLNKNYDQPTTFNFSIDSSKNYTIGNVWAFDRGSS
NITQRTPIVNIKDNTFTYTVPALTACHIVLEAAEP

>non-antioxidant_762
MMCLKLNLLDHVFANPFMNAAGVLCSTEEDLRCMTASSSGALVSKSCTSAPRDGNPEPR
YMAFPLGSINSMGLPNLGFDFYLKYASDLHDYSKKPLFLSISGLSVEENVAMVRRLAPVA
QEKGVLLELNLSCPNVPGKPQVAYDFEAMRTYLQQVSLAYGLPFGVKMPPYFDIAHFDTA
AAVLNEFPLVKFVTCVNSVGNGLVIDAESESVVIKPKQGFGGLGGKYILPTALANVNAFYR
RCPDKLVFGCGGVYSGEDAFLHILAGASMVQVGTALQEEGPGIFTRLEDELLEIMARKGY
RTLEEFRGRVKTIE
>non-antioxidant_763
KKHVVCQSCDINCVVEAEVKADGKIQTKSISEPHPTTPPNSICMKSVNADTIRTHKDRVLY
PLKNVGSKRGEQRWERISWDQALDEIAEKLKKIIAKYGPESLGVSQTEINQQSEYGTLRRF
MNLLGSPNWTSAMYMCIGNTAGVHRVTHGSYSFASFADSNCLLFIGKNLSNHNWVSQFN
DLKAALKRGCKLIVLDPRRTKVAEMADIWLPLRYGTDAALFLGMINVIINEQLYDKEFVE
NWCVGFEELKERVQEYPLDKVAEITGCDAGEIRKAAVMFATESPASIPWAVSTDMQKNSCS
AIRAQCILRAIVGSFVNGAEILGAPHSDLVPISKIQMHEALPEEKKKLQLGTETYPFLTYTG
MSALEEPSERVYGVKYFHNMGAFMANPTALFTAMATEKPYPVKAFFALASNALMGYAN
QQNALKGLMNQDLVVCYDQFMTPTAQLADYVLPGDHWLERPVVQPNWEGIPFGNTSQQ
VVEPAGEAKDEYYFIRELAVRMGLEEHFPWKDRLELINYRISPTGMEWEEYQKQYTYMS
KLPDYFGPEGVGVATPSGKVELYSSVFEKLGYDPLPYYHEPLQTEISDPELAKEYPLILFAG
LREDSNFQSCYHQPGILRDAEPDPVALLHPKTAQSLGLPSGEWIWVETTHGRLKLLLKHD
GAQPEGTIRIPHGRWCPEQEGGPETGFSGAMLHNDAMVLSDDDWNLDPEQGLPNLRGGI
LAKAYKC
>non-antioxidant_764
MVIASVEDGGDGDTSKDDWLWYKQPASQTDATATAGGNYGNPDNNRWQQTTLPFGNGK
IGGTVWGEVSRERVTFNEETLWTGGPGSSTSYNGGNNETKGQNGATLRALNKQLANGAE
TVNPGNLTGGENAAEQGNYLNWGDIYLDYGFNDTTVTEYRRDLNLSKGKADVTFKHDG
VTYTREYFASNPDNVMVARLTASKAGKLNFNVSMPTNTNYSKTGETTTVKGDTLTVKGA
LGNNGLLYNSQIKVVLDNGEGTLSEGSDGASLKVSDAKAVTLYIAAATDYKQKYPSYRTG
ETAAEVNTRVAKVVQDAANKGYTAVKKAHIDDHSAIYDRVKIDLGQSGHSSDGAVATDAL
LKAYQRGSATTAQKRELETLVYKYGRYLTIGSSRENSQLPSNLQGIWSVTAGDNAHGNTP
WGSDFHMNVNLQMNYWPTYSANMGELAEPLIEYVEGLVKPGRVTAKVYAGAETTNPET
TPIGEGEGYMAHTENTAYGWTAPGQSFSWGWSPAAVPWILQNVYEAYEYSGDPALLDRV
YALLKEESHFYVNYMLHKAGSSSGDRLTTGVAYSPEQGPLGTDGNTYESSLVWQMLNDA
IEAAKAKGDPDGLVGNTTDCSADNWAKNDSGNFTDANANRSWSCAKSLLKPIEVGDSGQ
IKEWYFEGALGKKKDGSTISGYQADNQHRHMSHLLGLFPGDLITIDNSEYMDAAKTSLRY
RCFKGNVLQSNTGWAIGQRINSWARTGDGNTTYQLVELQLKNAMYANLFDYHAPFQIDG
NFGNTSGVDEMLLQSNSTFTDTAGKKYVNYTNILPALPDAWAGGSVSGLVARGNFTVGTT
WKNGKATEVRLTSNKGKQAAVKITAGGAQNYEVKNGDTAVNAKVVTNADGASLLVFDT
TAGTTYTITKKAS
>non-antioxidant_765
MFDKHTHTLIAQRLDQAEKQREQIRAISLDYPEITIEDAYAVQREWVRLKIAEGRTLKGHKI
GLTSKAMQASSQISEPDYGALLDDMFFHDGSDIPTDRFIVPRIEVELAFVLAKPLRGPNCTL
FDVYNATDYVIPALELIDARCHNIDPETQRPRKVFDTISDNAANAGVILGGRPIKPDELDLR
WISALMYRNGVIEETGVAAGVLNHPANGVAWLANKLAPYDVQLEAGQIILGGSFTRPVPA
RKGDTFHVDYGNMGSISCRFV

>non-antioxidant_766
MHVFYSEERRGNLLILREGEVKHFRVRRIEKDEEFGVIHEGKIYVCKVRREDKREISCEIVE
ELETKLPPKDITLYQSVTVDLKTMDTIVRQATELGVLTFVPIISERSFQKEEAILKKTEKWK
RIVIEAMKQSRRPIPMEIKKPVRLSDLIPESEENIILDNFYEGVKPKDVNLEAKTYSVVVGP
EGGFSKRESQILREKGFKSVLLEPYTLRTETAVVSIVSILMNF
>non-antioxidant_767
MPAIFTHEGKVEGVPGNYPLTAENLFRIGLALCTLWILDKEIEEPTLSIPETNFVTLALSVGF
MNAGGSVNVGKGGDIKLFLQKGEIYVLEFQPLSETDIKKLESILFGRAPIPKKTGEDIGSFK
C
>non-antioxidant_768
MSKQAAVIELGYMGISVKDPDAWKSFATDMLGLQVLDEGEKDRFYLRMDYWHHRIVVH
HNGQDDLEYLGWRVAGKPEFEALGQKLIDAGYKIRICDKVEAQERMVLGLMKTEDPGG
NPTEIFWGPRIDMSNPFHPGRPLHGKFVTGDQGLGHCIVRQTDVAEAHKFYSLLGFRGDV
EYRIPLPNGMTAELSFMHCNARDHSIAFGAMPAAKRLNHLMLEYTHMEDLGYTHQQFVK
NEIDIALQLGIHANDKALTFYGATPSGWLIEPGWRGATAIDEAEYYVGDIFGHGVEATGYG
LDVKLS
>non-antioxidant_769
MQIKAGLIWMNGAFVPQEEAKTSVLSHALHYGTSVFEGIRAYETAKGPAIFRLKEHVKRF
YNSAKVLRMEIPFAPEELEEAIKEVVRRNGYRSCYIRPLAWMGAKALGVNPLPNNPAEVM
VAAWEWGAYLGEEAVRKGARLITSSWARFPANVMPGKAKVGGNYVNSALAKMEAVAA
GADEALLLDEEGYVAEGSGENLFFVRDGVIYALEHSVNLEGITRDSVIRIAKDLGYEVQVV
RATRDQLYMADEVFMTGTAAEVTPVSMIDWRPIGKGTAGPVALRLREVYLEAVTGRRPE
YEGWLTYVNGQ
>non-antioxidant_770
MDAVKKAILGEVLEEEEAYEVMRALMAGEVSPVRAAGLLVALSLRGERPHEIAAMARAM
REAARPLRVHRRPLLDIVGTGGDGKGLMNLSTLAALVAAAGGVAVAKHGNRAASSRAGS
ADLLEALGVDLEAPPERVGEAIEELGFGFLFARVFHPAMRHVAPVRAELGVRTVFNLLGPL
TNPAGADAYVLGVFSPEWLAPMAEALERLGARGLVVHGEGADELVLGENRVVEVGKGA
YALTPEEVGLKRAPLEALKGGGPEENAALARRLLKGEEKGPLADAVALAAGAGFYAAGK
TPSLKEGVALAREVLASGEAYLLLERYVAFLRA
>non-antioxidant_771
MTRINLTLVSELADQHLMAEYRELPRVFGAVRKHVANGKRVRDFKISPTFILGAGHVTFFY
DKLEFLRKRQIELIAECLKRGFNIKDTTVQDISDIPQEFRGDYIPHEASIAISQARLDEKIAQ
RPTWYKYYGKAIYA
>non-antioxidant_772
ADGRSTRYWDCCKPSCGWAKKAPVNQPVFSCNANFQRITDFDAKSGCEPGGVAYSCADQ
TPWAVNDDFALGFAATSIAGSNEAGWCCACYELTFTSGPVAGKKMVVQSTSTGGDLGSN
HFDLNIPGGGVGIFDGCTPQFGGLPGQRYGGISSRNECDRFPDALKPGCYWRFDWFKNAD
NPSFSFRQVQCPAELVARTGCRRNDDGNFPAV
>non-antioxidant_773
MATFLGLSSKQEKALVRLDKYLNLGEIAVSLVTDSATSIKVEGRQGYYQVSYKQPHQLYR
ALALLSAALRSGQDEVQIEEEAAYEDLAYMADCSRNAVLNLSSAKKMIEVLALMGYSTFE
LYMEDTYEIENQPYFGYFRGRYTVAELQEIEDYAADFDMSFVPCIQTLAHLSAFVKWGIKE
VQELRDVEDILLIGEEKVYDLIEGMFQTMAHLHTRKINIGMDEAHLVGLGRYLIKHGFQN

RSLLMCQHLERVLDIADKYGFNCQMWSDMFFKLMSADGQYDRDVEIPEETRVYLDRLK
ERVTLVYWDYYQDSEEKYNRNFQNHHKISQDIAFAGGAWKWIGFTPHNHFSRLVAIEANK
ACRKNQVKEVIVTGWGDNGGETSQFSVLPALQIWAELAYRNDLKKVSEHFLVSTGLDFD
DFMKIDLANLLPDLPDNLSGINPNRYVLYQDVLCPLLEQHIRPEKDKQHFASSAQQLGEIS
KRAGEYAYIFETQAQLNALLALKISITSGIQKAYRNGDKEHLSALAEKDFPQLYQMVEDFS
DQFSRQWQQENKIFGLDTIDIRFGGLLKRIKRAQERLEQFISGQIDCVEELEQEILPFNDFY
KDQGLTATTANQWHLIATASTIYTT
>non-antioxidant_774
GGDNSVFDIFELTGAARKGSGRRLVKGPDPSSPAFRIEDANLIPPVPDDKFQDLVDAVRTEK
GFLLLASLRQMKKTRGTLLALERKDHSGQVFSVVSNGKAGTLDLSLTVQGKQHVVSVEE
ALLATGQWKSITLFVQEDRAQLYIDCEKMENAELDVPIQSVFTRDLASIARLRIAKGGVND
NFQGVLQNVRFVFGTTPEDILRNKGCS
>non-antioxidant_775
DACEQAAIQCVESACESLCTEGEDRTGCYMYIYSNCPPYV
>non-antioxidant_776
TDPDPLQDFCVADLDGKAVSVNGHTCKPMSEAGDDFLFSSKLTKAGNTSTPNGSAVTELD
VAEWPGTNTLGVSMNRVDFAPGGTNPPHIHPRATEIGMVMKGELLVGILGSLDSGNKLYS
RVVRAGETFVIPRGLMHFQFNVGKTEAYMVVSFNSQNPGIVFVPLTLFGSDPPIPTPVLTKA
LRVEAGVVELLKSKFAGGS
>non-antioxidant_777
GHMTKLNQESTAPKVLFTGVVDARGERAVLALGGSLAGSAAEASHLVTDRIRRTVKFLC
ALGRGIPILSLDWLHQSRKAGFFLPPDEYVVTDPEQEKNFGFSLQDALSRARERRLLEGYE
IYVTPGVQPPPPQMGEIISCCGGTYLPSMPRSYKPQRVVITCPQDFPHCSIPLRVGLPLLSPE
FLLTGVLKQEAKPEAFVLSPLEMSST
>non-antioxidant_778
VEDDKEGHLVCRIGDWLQERYEIVGNLGEGTFGKVVECLDHARGKSQVALKIIRNVGKY
REAARLEINVLKKIKEKDKENKFLCVLMSDWFNFHGHMCIAFELLGKNTFEFLKENNFQP
YPLPHVRHMAYQLCHALRFLHENQLTHTDLKPENILFVNSEFETLYNEHKSCEEKSVKNTS
IRVADFGSATFDHEHHTTIVATRHYRPPEVILELGWAQPCDVWSIGCILFEYYRGFTLFQTH
ENREHLVMMEKILGPIPSHMIHRTRKQKYFYKGGLVWDENSSDGRYVKENCKPLKSYML
QDSLEHVQLFDLMRRMLEFDPAQRITLAEALLHPFFAGLTPEERSFHTSRNPSR
>non-antioxidant_779
MKTLVHVASVEKGRSYEDFQKVYNAIALKLREDDEYDNYIGYGPVLVRLAWHISGTWDK
HDNTGGSYGGTYRFKKEFNDPSNAGLQNGFKFLEPIHKEFPWISSGDLFSLGGVTAVQEM
QGPKIPWRCGRVDTPEDTTPDNGRLPDADKDAGYVRTFFQRLNMNDREVVALMGAHAL
GKTHLKNSGYEGPGGAANNVFTNEFYLNLLNEDWKLEKNDANNEQWDSKSGYMMLPT
DYSLIQDPKYLSIVKEYANDQDKFFKDFSKAFEKLLENGITFPKDAPSPFIFKTLEEQGL
>non-antioxidant_780
MRGSHHHHHHGSMTDHVREADDANIDDLLGDLGGTARAERAKLVEWLLEQGITPDEIRA
TNPPLLLATRHLVGDDGTYVSAREISENYGVDLELLQRVQRAVGLARVDDPDAVVHMRA
DGEAAARAQRFVELGLNPDQVVLVVRVLAEGLSHAAEAMRYTALEAIMRPGATELDIAK
GSQALVSQIVPLLGPMIQDMLFMQLRHMMETEAVNAGERAAGSSP
>non-antioxidant_781
MVVSENVVSAPMPGKVLRVLVRVGDRVRVGQGLLVLEAMKMENEIPSPRDGVVKRILVK

EGEAVDTGQPLIELG
>non-antioxidant_782
MTKQYLTHRCLIAPPEMADDFFANTVIYLARHDEEGAQGIIINRPAGIQIKELLNDLDIDAD
NVNPHEVLQGGPLRPEAGFVLHTGQPTWHSSIAVGENVCITTSKDILDAIAHNEGVGRYQI
ALGYASWGKNQLEDEIARGDWLICDADMDLIFNLPYDDRWDAAYKKIGVDRTWLASEIG
HALEHHHHHH
>non-antioxidant_783
MANVDEYITQLPAGANLALMVQKVGASAPAIDYHSQQMALPASTQKVITALAALIQLGPD
FRFTTTLETKGNVENGVLKGDLVARFGADPTLKRQDIRNMVATLKKSGVNQIDGNVLIDT
SIFASHDKAPGWPWNDMTQCFSAPPAAAIVDRNCFSVSLYSAPKPGDMAFIRVASYYPVT
MFSQVRTLPRGSAEAQYCELDVVPGDLNRFTLTGCLPQRSEPLPLAFAVQDGASYAGAILK
YELKQAGITWSGTLLRQTQVNEPGTVVASKQSAPLHDLLKIMLKKSDNMIADTVFRMIGH
ARFNVPGTWRAGSDAVRQILRQQAGVDIGNTIIADGSGLSRHNLIAPATMMQVLQYIAQH
DNELNFISMLPLAGYDGSLQYRAGLHQAGVDGKVSAKTGSLQGVYNLAGFITTASGQRM
AFVQYLSGYAVEPADQRNRRIPLVRFESRLYKDIYQNN
>non-antioxidant_784
AEAGITGTWYNQLGSTFIVTAGADGALTGTYESAVGNAESRYVLTGRYDSAPATDGSGTA
LGWTVAWKNNYRNAHSATTWSGQYVGGAEARINTQWLLTSGTTEANAWKSTLVGHDTF
TKVKPSAAS
>non-antioxidant_785
GMDTNGVLYAANMTNALAKEIPESKWDIQLIPELGTLRKLFIHIVRVRDVYRDGLKTGSIK
FPGRLASDEHRLLDELERSMEELVFEFKQTTFNSIKMGENYLSIMELLGTVIQHEGIHQGQ
YYVALKQSGINLPKQWVQDWHM
>non-antioxidant_786
MAREVKLTKAGYERLMQQLERERERLQEATKILQELMESSDDYDDSGLEAAKQEKARIE
ARIDSLEDILSRAVILEEGSGEVIGLGSVVELEDPLSGERLSVQVVSPAEANVLDTPMKISD
ASPMGKALLGHRVGDVLSLDTPKGKREFRVVAIHG
>non-antioxidant_787
ADQLTEEQIAEFKEAFSLFDKDGDGTITTKELGTVMRSLGQNPTEAELQDMINEVDADGN
GTIDFPEFLTMMARKMKDTDSEEEIREAFRVFDKDGNGYISAAELRHVMTNLGEKLTDEE
VDEMIREADIDGDGQVNYEEFVQMMTAK
>non-antioxidant_788
KFYATFLAAEYFRKFKKRKEQ
>non-antioxidant_789
GMPSEKQPQSKGNKMAILKLDEHLYISPQLTKADAEQIAQLGIKTIICNRPDREEESQPDFA
QIKQWLEQAGVTGFHHQPVTARDIQKHDVETFRQLIGQAEYPVLAYCRTGTRCSLLWGFR
RAAEGMPVDEIIRRAQAAGVNLENFRERLDNARV
>non-antioxidant_790
AIWRSRSFDEAIEMFRESLYSAKNEVIVVTPSEFFETIREDLIKTLERGVTVSLYIDKIPDLSE
FKGKGNFFVRQFYKLNHLIGMTDGKEVVTIQNATFDSIGPPSFKSTYPEIIFSQYSLIIEIFKE
STLEKEIIGNPKDIRFFAMFHAVDFVKNHLKNRNIYAEITGKNLESGRLETLTGRVVGYTLS
LREAVNNIHLETENGVVKVGGMFAVIEDYESTEIKFIMGGSRS
>non-antioxidant_791
GEHIPGTLRFRLSPAARNILEKHSLDASQGTATGPRGIFTKEDALKLVQLKQTGKILEHHHH

HH
>non-antioxidant_792
MAHHHHHHMRKIYIAGPAVFNPDMGASYYNKVRELLKKENVMPLIPTDNEATEALDIRQ
KNIQMIKDCDAVIADLSPFRGHEPDCGTAFEVGCAAALNKMVLTFTSDRRNMREKYGSG
VDKDNLRVEGFGLPFNLMLYDGVEVFDSFESAFKYFLANFPSK
>non-antioxidant_793
GSHMRWRKWRHITKLDPDRTNTDEIIKAVADSGTDAVMISGTQNVTYEKARTLIEKVSQY
GLPIVVEPSDPSNVVYDVDYLFVPTVLNSADGDWITGKHAQWVRMHYENLQKFTEIIESE
FIQIEGYIVLNPDSAVARVTKALCNIDKELAASYALVGEKLFNLPIIYIEYSGTYGNPELVAE
VKKVLDKARLFYGGGIDSREKAREMLRYADTIIVGNVIYEKGIDAFLETLP
>non-antioxidant_794
IVGGTDATLGEFPYQLSFQETFIGFSFHFCGASIYNENYAITAGHCVYGDDYENPSGLQIVA
GELDMSVNEGSEQIITVSKIILHENFDYNLLDNDISLLKLSGSLTFNDNVAPIALPEQGHTAT
GDVIVTGWGTTSEGGNTPDVLQKVTVPLVSDEDCRADYGADEILDSMICAGVPEGGKDS
CQGDSGGPLAASDTGSTYLAGIVSWGYGCARPGYPGVYTEVSYHVDWIKANAV
>non-antioxidant_795
SNAMGWKMQATVTEIGKHAIDDSEKMIILFGETATDTLKQHAVIQSFPEKDQVTLAEGDH
LKIGDTNYTITKVGSFANSNLQSIAHSTLIFADAPTDEDDVIRNGVYLTPHQLPKITIGTTID
YLVNGA
>non-antioxidant_796
MGARKASAGASRAATAGVRISHPQRLIDPSIQASKLELAEFHARYADLLLRDLRERPVSLV
RGPDGIGGELFFQKHAARLKIPGIVQLDPALDPGHPPLLQIRSAEALVGAVQMGSIEFHTW
NASLANLERPDRFVLDLDPDPALPWKRMLEATQLSLTLLDELGLRAFLKTSGGKGMHLLV
PLERRHGWDEVKDFAQAISQHLARLMPERFSAVSGPRNRVGKIFVDYLRNSRGASTVAAY
SVRAREGLPVSVPVFREELDSLQGANQWNLRSLPQRLDELAGDDPWADYAGTRQRISAA
MRRQLGRG
>non-antioxidant_797
SNAMSANDKLTILWTTDNKDTVFNMLAMYALNSKNRGWWKHINIILWGASVKLVANDT
QVQTEILEMLQSGITIEACQDCCENFGVASIITNLGITVRYMGIPLTEYLKNGEKILSI
>non-antioxidant_798
AYPSFEAYSNYKVDRTDLETFLDKQKEVSLYYLLQNIAYPEGQFNNGVPGTVIASPSTSNP
DYYYQWTRDSAITFLTVLSELEDNNFNTTLAKAVEYYINTSYNLQRTSNPSGSFDDENHK
GLGEPKFNTDGSAYTGAWGRPQNDGPALRAYAISRYLNDVNSLNEGKLVLTDSGDINFSST
EDIYKNIIKPDLEYVIGYWDSTGFDLWEENQGRHFFTSLVQQKALAYAVDIAKSFDDGDFA
NTLSSTASTLESYLSGSDGGFVNTDVNHIVENPDLLQQNSRQGLDSATYIGPLLTHDIGESS
STPFDVDNEYVLQSYYLLLEDNKDRYSVNSAYSAGAAIGRYPEDVYNGDGSSEGNPWFL
ATAYAAQVPYKLAYDAKSASNDITINKINYDFFNKYIVDLSTINSAYQSSDSVTIKSGSDEF
NTVADNLVTFGDSFLQVILDHINDDGSLNEQLNRYTGYSTGAYSLTWSSGALLEAIRLRNK
VKALA
>non-antioxidant_799
MRRVEKVIIVEGRSDKQKVAAVLNEPVVIVCTNGTISDARLEELADELEGYDVYLLADAD
EAGEKLRRQFRRMFPEAEHLYIDRAYREVAAAPIWHLAQVLLRARFDVRIESLMRGRGE
>non-antioxidant_800
MGSDKIHHHHHHMIRPEYLRVLRKIYDRLKNEKVNWVVTGSLSFALQGVPVEVHDIDIQT

DEEGAYEIERIFSEFVSKKVRFSSTEKICSHFGELIIDGIKVEIMGDIRKRLEDGTWEDPVDL
NKYKRFVETHGMKIPVLSLEYEYQAYLKLGRVEKAETLRKWLNERK
>non-antioxidant_801
MALKYPSGKEYRGNKPNAARRPAADYANRGMTLEDDLNATNEYYRERGIAVIHKKPTPV
QIVRVDYPKRSAAVITEAYFRQASTTDYNGVYRGKYIDFEAKETKNKTAFPLKNFHAHQIR
HMEQVVAHGGICFAILRFSLLNETYLLDASHLIAWWNKQEAGGRKSIPKQEIERHGHSIPL
GYQPRIDYISVVDNVYFTR
>non-antioxidant_802
GSHMSKKFALTAEQRASFEKNGFIGPFDAYSPEEMKETWKRTRLRLLDRSAAAYQDLDAI
SGGTNIANYDRHLDDDFLASHICRPEICDRVESILGPNVLCWRTEFFPKYPGDEGTDWHQA
DTFANASGKPQIIWPENEEFGGTITVWTAFTDANIANGCLQFIPGTQNSMNYDETKRMTY
EPDANNSVVKDGVRRGFFGYDYRQLQIDENWKPDEASAVPMQMKAGQFIIFWSTLMHA
SYPHSGESQEMRMGFASRYVPSFVHVYPDSDHIEEYGGRISLEKYGAVQVIGDETPEYNRL
VTHTTRGKKFEAV
>non-antioxidant_803
GAEFEEPRVIDLWDLAQSANLTDKELEAFREELKHFEAKIEKHNHYQKQLEIAHEKLRHA
ESVGDGERVSRSREKHALLEGRTKELGYTVKKHLQDLSGRISRARHNEL
>non-antioxidant_804
KTCSQAEFRCHDGKCISRQFVCDSDRDCLDGSDEASCPVLTCGPASFQCNSSTCIPQLWAC
DNDPDCEDGSDEWPQRCRG
>non-antioxidant_805
AYVINEACISCGACEPECPVNAISSGDDRYVIDADTCIDCGACAGVCPVDAPVQA
>non-antioxidant_806
SNAMEILYIKGDATAPIGSGVKVITHICNDIGGWGKGFVLALSKKWKMPEEAYRQWYKSQ
EEFTLGAVQFVNVENKLYVANMIGQHGIYKDSKGLPPIRYDAVRQCLKEVALFTIAHKASV
HMPRIGCGLAGGKWELMEQIIKEELITKEIAVTVYDL
>non-antioxidant_807
QSSVTLFGIVDTNVAYVNKDAAGDSRYGLGTSGASTSRLGLRGTEDLGGGLKAGFWLEG
EIFGDDGNASGFNFKRRSTVSLSGNFGEVRLGRDLVPTSQKLTSYDLFSATGIGPFMGFRN
WAAGQGADDNGIRANNLISYYTPNFGGFNAGFGYAFDEKQTIGTADSVGRYIGGYVAYD
NGPLSASLGLAQQKTAVGGLATDRDEITLGASYNFGVAKLSGLLQQTKFKRDIGGDIKTNS
YMLGASAPVGGVGEVKLQYALYDQKAIDSKAHQITLGYVHNLSKRTALYGNLAFLKNKD
ASTLGLQAKGVYAGGVQAGESQTGVQVGIRHAF
>non-antioxidant_808
MDDDGTGQKQIWRIEGSNKVPVDPATYGQFYGGDSYIILYNYRHGGRQGQIIYNWQGAQ
STQDEVAASAILTAQLDEELGGTPVQSRVVQGKEPAHLMSLFGGKPMIIYKGGTSREGGQT
APASTRLFQVRANSAGATRAVEVLPKAGALNSNDAFVLKTPSAAYLWVGTGASEAEKTG
AQELLRVLRAQPVQVAEGSEPDGFWEALGGKAAYRTSPRLKDKKMDAHPPRLFACSNKI
GRFVIEEVPGELMQEDLATDDVMLLDTWDQVFVWVGKDSQEEEKTEALTSAKRYIETDP
ANRDRRTPITVVKQGFEPPSFVGWFLGWDDDYWSVDPLDRAMAELAA
>non-antioxidant_809
MTNKLFEHTVLYDSGDAFFELKGNASMKLSPKAAIEVCNEAAKKGLWILGIDGGHWLNP
GFRIDSSASWTYDMPEEYKSKIPENNRLAIENIKDDIENGYTAFIITLKM
>non-antioxidant_810

MLAKNKGKIPGLKIDQKIRGQMPERGWTEDDIKNTVSNGATGTSFDKRSPKKTPPDYLGR
NDPATVYGSPGKYVVVNDRTGEVTQISDKTDPGWVDDSRIQWGNKNDQ
>non-antioxidant_811
AGKKRPWKCCDEAVCTRSIPPICTCMDEVFECPKTCKSCGPSMGDPSRRICQDQYVGDPG
PICRPWECCDKAICTRSNPPTCRCVDEVKKCAPTCKTCLPSRSRPSRRVCIDSYFGPVPPRC
TPR
>non-antioxidant_812
DEIIDETDNLYVSQGPGVDISGDVNLTDFDKIGWPNVEAVQSYQREFNAVSNIFDTIYPIGTI
YENAVNPNNPVTYMGFGSWKLFGQGKVLVGWNEDISDPNFALNNNDLDSGGNPSHTAG
GTGGSTSVTLENTNLPATETDEEVLIVDENGSVIVGGCQYDPDESGPIYTKYREAKASTNS
THTPPTSITNIQPYITVYRWIRIA
>non-antioxidant_813
MVKEFNTQTELSVRLEALWAVLSKDFITVVPKVLPHIVKDVQLIEGDGGVGTILIFNFLPEV
SPSYQREEITEFDESSHEIGLQVIEGGYLSQGLSYYKTTFKLSEIEEDKTLVNVKISYDHDSD
IEEKVTPTKTSQSTLMYLRRLERYLSNGSA
>non-antioxidant_814
MKEFAYSEPCLDKEDKKAVLEVLNSKQLTQGKRSLLFEEALCEFLGVKHALVFNSATSALL
TLYRNFSEFSADRNEIITTPISFVATANMLLESGYTPVFAGIKNDGNIDELALEKLINERTKAI
VSVDYAGKSVEVESVQKLCKKHSLSFLSDSSHALGSEYQNKKVGGFALASVFSFHAIKPIT
TAEGGAVVTNDSELHEKMKLFRSHGMLKKDFFEGEVKSIGHNFRLNEIQSALGLSQLKKA
PFLMQKREEAALTYDRIFKDNPYFTPLHPLLKDKSSNHLYPILMHQKFFTCKKLILESLHK
RGILAQVHYKPIYQYQLYQQLFNTAPLKSAEDFYHAEISLPCHANLNLESVQNIAHSVLKT
FESFKIE
>non-antioxidant_815
GSHMLEADLELERAADVRWEEQAEISGSSPILSITISEDGSMSIKNEEEEQTLGGGGSGGG
G
>non-antioxidant_816
AGVLWDVPSPPPVGKAELEDGAYRIKQKGILGYSQIGAGVYKEGTFHTMWHVTRGAVLM
HKGKRIEPSWADVKKDLISYGGGWKLEGEWKEGEEVQVLALEPGKNPRAVQTKPGLFKT
NTGTIGAVSLDFSPGTSGSPIVDKKGKVVGLYGNGVVTRSGAYVSAIANTEKSIEDNPEIED
DIFRK
>non-antioxidant_817
MASLSRFRGCLAGALLGDCVGSFYEAHDTVDLTSVLRHVQSLEPDPGTPGSERTEALYYT
DDTAMARALVQSLLAKEAFDEVDMAHRFAQEYKKDPDRGYGAGVVTVFKKLLNPKCR
DVFEPARAQFNGKGSYGNGGAMRVAGISLAYSSVQDVQKFARLSAQLTHASSLGYNGAIL
QALAVHLALQGESSSEHFLKQLLGHMEDLEGDAQSVLDARELGMEERPYSSRLKKIGELL
DQASVTREEVVSELGNGIAAFESVPTAIYCFLRCMEPDPEIPSAFNSLQRTLIYSISLGGDTD
TIATMAGAIAGAYYGMDQVPESWQQSCEGYEETDILAQSLHRVFQKS
>non-antioxidant_818
MRGSHHHHHHGMASSYSKWFNLEKIHSIEVQSLPEFFTNRIPSKTPEVYMRYRNFMVNSY
RLNPNEYFSVTTARRNVSGDAAALFRLHKFLTKWGLINYQVDSK
>non-antioxidant_819
MAQERPSCAVEPEHVQRLLLSSREAKKSAYCPYSRFPVGAALLTGDGRIFSGCNIENACYP
LGVCAERTAIQKAISEGYKDFRAIAISSDLQEEFISPCGACRQVMREFGTDWAVYMTKPDG

TFVVRTVQELLPASFGPEDLQKIQ
>non-antioxidant_820
GHMHAPLVSKDLDYISTANHDQPPRHLGSRFSAEGEFLPEPGNTVVCHLVEGSQTESAIVS
TRQRFLDMPEASQLAFTPVSSLHMTVFQGVIESRRALPYWPQTLPLDTPIDAVTDYYRDRL
STFPTLPAFNMRVTGLRPVGMVMKGATAEDDSIVALWRDTFADFFGYRHPDHDTYEFHIT
LSYIVSWFEPECLPRWQAMLDEELEKLRVAAPVIQMRPPAFCEFKDMNHFKELVVFDKRG
S
>non-antioxidant_821
MGSSHHHHHHSSGRENLYFQGHMNHSIPTLRTERLTLRPLAMADFPAYRDFMASPRSTGV
GGPYDLPSTWGVFCHDLANWHFFGHGALMIDLGETGECIGQIGINHGPLFPEKELGWLLY
EGHEGRGYAAEAAVALRDWAFETLNLPTLVSYVSPQNRKSAAVAERIGGTLDPLAPRSDPE
DLVYRYHQVKTAGS
>non-antioxidant_822
GSKVEDPKDFPSELLSFLSHAVFSNRTLACFAIYTTKEKAALLYKKIMEKYSVTFISRHNSY
NHNILFFLTPHRHRVSAINNYAQKLCTFSFLICKGVNKEYLMYSALTRDPFSVIEESLPGGL
KEHDFNPESS
>non-antioxidant_823
GSPEFVNSELTQLDEYGEWILEQAGEDKENLPSDVELYKKAAELDVLNDPKIGCVLAQCL
FDEDIVNEIAEHNAFFTKILVTPEYEKNFMGGIERFLGLEHKDLIPLLPKILVQLYNNDIISEE
EIMRFGTKSSKKFVPKEVSKKVRRAAKPFITWLETAESDDDEEDDELERPHRD
>non-antioxidant_824
GMPDSPTLLDLFAEDIGHANQLLQLVDEEFQALERRELPVLQQLLGAKQPLMQQLERNGR
ARAEILREAGVSLDREGLARYARERADGAELLARGDELGELLERCQQANLRNGRIIRANQ
ASTGSLLNILRGQDAPSLYDSRGGTASSSRQRPLSQA
>non-antioxidant_825
ADTRIGVTIYKYDDNFMSVVRKAIEQDAKAAPDVQLLMNDSQNDQSKQNDQIDVLLAK
GVKALAINLVDPAAAGTVIEKARGQNVPVVFFNKEPSRKALDSYDKAYYVGTDSKESGII
QGDLIAKHWAANQGWDLNKDGQIQFVLLKGEPGHPDAEARTTYVIKELNDKGIKTEQLQ
LDTAMWDTAQAKDKMDAWLSGPNANKIEVVIANNDAMAMGAVEALKAHNKSSIPVFG
VDALPEALALVKSGALAGTVLNDANNQAKATFDLAKNLADGKGAADGTNWKIDNKVV
RVPYVGVDKDNLAEFSKK
>non-antioxidant_826
ATHTAQTQTHLNFTQIKTVDELNQALVEAKGKPVMLDLYADWCVACKEFEKYTFSDPQV
QKALADTVLLQANVTANDAQDVALLKHLNVLGLPTILFFDGQGQEHPQARVTGFMDAET
FSAHLRDRQPHHHHHH
>non-antioxidant_827
MAHHHHHHDDDDKAPPTLWSRVTKFGSGWGFWVSPTVFITTTHVVPTGVKEFFGEPLSSI
AIHQAGEFTQFRFSKKMRPDLTGMVLEEGCPEGTVCSVLIKRDSGELLPLAVRMGAIASM
RIQGRLVHGQSGMLLTGANAKGMDLGTIPGDCGAPYVHKRGNDWVVCGVHAAATKSG
NTVVCAVQAGEGETALE
>non-antioxidant_828
MGSSHHHHHHSSGLVPRGSHMGMAPGGYVAPKAVWLPAVKAKGLEISGTFTHRQGHIYM
EMNFTNKALQHMTDFAIQFNKNSFGVIPSTPLAIHTPLMPNQSIDVSLPLNTLGPVMKMEP
LNNLQVAVKNNIDVFYFSCLIPLNVLFVEDGKMERQVFLATWKDIPNENELQFQIKECHLN

ADTVSSKLQNNNVYTIAKRNVEGQDMLYQSLKLTNGIWILAELRIQPGNPNYTLSLKCRA
PEVSQYIYQVYDSILKN
>non-antioxidant_829
GHMNSFVGLRVVAKWSSNGYFYSGKITRDVGAGKYKLLFDDGYECDVLGKDILLCDPIP
LDTEVTALSEDEYFSAGVVKGHRKESGELYYSIEKEGQRKWYKRMAVILSLEQGNRLREQ
YGLG
>non-antioxidant_830
PNFSGNWKIIRSENFEELLKVLGVNVMLRKIAVAAASKPAVEIKQEGDTFYIKTSTTVRTTE
INFKVGEEFEEQTVDGRPCKSLVKWESENKMVCEQKLLKGEGPKTSWTLELTNDGELIET
MTADDVVCTKVYVRE
>non-antioxidant_831
ESAFNQTEFNKLLLECVVKTQSSVAKILGIESLSPHVSGNSKFEYANMVEDIREKVSSEME
RFFPKNDDE
>non-antioxidant_832
GAMAPATVNVEVLQDKLDHPWALAFLPDNHGMLITLRGGELRHWQAGKGLSAPLSGVP
DVWAHGQGGLLDVVLAPDFAQSRRIWLSYSEVGDDGKAGTAVGYGRLSDDLSKVTDFRT
VFRQMPKLSTGNHFGGRLVFDGKGYLFIALGENNQRPTAQDLDKLQGKLVRLTDQGEIPD
DNPFIKESGVRAEIWSYGIRNPQGMAMNPWSNALWLNEHGPRGGDEINIPQKGKNYGWP
LATWGINYSGFKIPEAKGEIVAGTEQPVFYWKDSPAVSGMAFYNSDKFPQWQQKLFIGAL
KDKDVIVMSVNGDKVTEDGRILTDRGQRIRDVRTGPDGYLYVLTDESSGELLKVSPR
>non-antioxidant_833
MGSDKIHHHHHHMSLDMKEHPDAEVQKNQVLTLEDWKEKWVTRHISFHQEQGHQLLK
KHLDTFLKGQSGLRVFFPLCGKAIEMKWFADRGHTVVGVEISEIGIREFFAEQNLSYTEEPL
AEIAGAKVFKSSSGSISLYCCSIFDLPRANIGKFDRIWDRGALVAINPGDHDRYADIILSLLR
KEFQYLVAVLSYDPTKHAGPPFYVPSAELKRLFGTKCSMQCLEEVDALEERHKAWGLDY
LFEKLYLLTEK
>non-antioxidant_834
AISIKTPEDIEKMRVAGRLAAEVLEMIEPYVKPGVSTGELDRICNDYIVNEQHAVSACLGY
HGYPKSVCISINEVVCHGIPDDAKLLKDGDIVNIDVTVIKDGFHGDTSKMFIVGKPTIMGE
RLCRITQESLYLALRMVKPGINLREIGAAIQKFVEAEGFSVVREYCGHGIGRGFHEEPQVL
HYDSRETNVVLKPGMTFTIEPMVNAGKKEIRTMKDGWTVKTKDRSLSAQYEHTIVVTDN
GCEILTLRKDDTIPAIISHDE
>non-antioxidant_835
ELLPEIFRQTVEHAPIAISITDLKANILYANRAFRTITGYGSEEVLGKNESILSNGTTPRLVYQ
ALWGRLAQKKPWSGVLVNRRKDKTLYLAELTVAPVLNEAGETIYYLGMHRDTSELH
>non-antioxidant_836
QISVRGLAGVENVTELKKNFNRHLHFTLVKDRNVATPRDYYFALAHTVRDHLVGRWIRTQ
QHYYEKDPKRIYYLSLEFYMGRTLQNTMVNLALENACDEATYQLGLDMEELEEIEEDAG
LGNGGLGRLAACFLDSMATLGLAAYGYGIRYEFGIFNQKICGGWQMEEADDWLRYGNP
WEKARPEFTLPVHFYGRVEHTSQGAKWVDTQVVLAMPYDTPVPGYRNNVVNTMRLWS
AKAPNDFNLKDFNVGGYIQAVLDRNLAENISRVLYPNDNFFEGKELRLKQEYFVVAATLQ
DIIRRFKSSKFGCRDPVRTNFDAFPDKVAIQLNDTHPSLAIPELMRVLVDLERLDWDKAWE
VTVKTCAYTNHTVLPEALERWPVHLLETLLPRHLQIIYEINQRFLNRVAAAFPGDVDRLRR
MSLVEEGAVKRINMAHLCIAGSHAVNGVARIHSEILKKTIFKDFYELEPHKFQNKTNGITPR

RWLVLCNPGLAEIIAERIGEEYISDLDQLRKLLSYVDDEAFIRDVAKVKQENKLKFAAYLE
REYKVHINPNSLFDVQVKRIHEYKRQLLNCLHVITLYNRIKKEPNKFVVPRTVMIGGKAAP
GYHMAKMIIKLITAIGDVVNHDPVVGDRLRVIFLENYRVSLAEKVIPAADLSEQISTAGTEA
SGTGNMKFMLNGALTIGTMDGANVEMAEEAGEENFFIFGMRVEDVDRLDQRGYNAQEY
YDRIPELRQIIEQLSSGFFSPKQPDLFKDIVNMLMHHDRFKVFADYEEYVKCQERVSALYK
NPREWTRMVIRNIATSGKFSSDRTIAQYAREIWGVEPSRQRLP
>non-antioxidant_837
MQFSKMHGLGNDFVVVDGVTQNVFFTPETIRRLANRHCGIGFDQLLIVEAPYDPELDFHY
RIFNADGSEVSQCGNGARCFARFVTLKGLTNKKDISVSTQKGNMVLTVKDDNQIRVNMG
EPIWEPAKIPFTANKFEKNYILRTDIQTVLCGAVSMGNPHCVVQVDDIQTANVEQLGPLLE
SHERFPERVNAGFMQIINKEHIKLRVYERGAGETQACGSGACAAVAVGIMQGLLNNNVQV
DLPGGSLMIEWNGVGHPLYMTGEATHIYDGFITL
>non-antioxidant_838
GSHMSKKILIVESDTALSATLRSALEGRGFTVDETTDGKGSVEQIRRDRPDLVVLAVDLSA
GQNGYLICGKLKKDDDLKNVPIVIIGNPDGFAQHRKLKAHADEYVAKPVDADQLVERAG
ALIGFPE
>non-antioxidant_839
SNAMTFNQEQDYWAGYKANERALIIQTWSGFGRYAPDHLYPPHILPLDTDNETLGTTVLQ
ALANSRTFVYDSPEDQDFFDTEKIRQRYEDWVAKLCGNLGYKTRRALFKNMMSVDIWLH
NGCLKISPSRHVKLEAWDAIDADDVILSLDNSPEEIGAGLKLALSRCR
>non-antioxidant_840
GMCVEKTPWELVIDFHGHTCPDIALGYRIAQLAQREMGIRPAPDSECLVKAYTQSCALDAI
QVLNKATIGRHALIIEETHRYMYQFHFTGTQDIHQFTVSPAVLDHLETLRHPDLSPRERQN
KVLEGVQYVLTLEESAFCHYDKIPGQLSKIV
>non-antioxidant_841
IKKEQKLIQAQNLVREFEKTHTVSAHRKAQKAVNLVSFEYKVKKMVLQERIDNVLKQGL
VR
>non-antioxidant_842
GMNQSIIFTEQLTWDVQLSAIHFTAQQQGMVIDCYIGQKVLEHLAAEKINNSEQALSLFEQ
FRFDIEEQAEKLIEQEAFDVQGHIQVERVD
>non-antioxidant_843
MEFMRVERVLLKDYTTLGVGGPAELWTVETREELKRATEAPYRVLGNGSNLLVLDEGVP
ERVIRLAGEFQTYDLKGWVGAGTLLPLLVQEAARAGLSGLEGLLGIPAQVGGAVKMNAG
TRFGEMADALEAVEVFHDGAFHVYCPEELGFGYRKSHLPPGGIVTRVRLKLKERPKEEIL
RRMAEVDRARKGQPKRKSAGCAFKNPPGQSAGRLIDERGLKGLRVGDAMISLEHGNFIV
NLGQARAKDVLELVRRVQEELPLELEWEVWP
>non-antioxidant_844
GSHMSGIALSRLAQERKAWRKDHPFGFVAVPTKNPDGTMNLMNWECAIPGKKGTPWEG
GLFKLRMLFKDDYPSSPPKCKFEPPLFHPNVYPSGTVCLSILEEDKDWRPAITIKQILLGIQE
LLNEPNIQSPAQAEAYTIYCQNRVEYEKRVRAQAKKFAPS
>non-antioxidant_845
MFADRLFNAMERNEPAPGMVLVAAPSMESEDFARSVILIIEHSEYATFGVNLASRSDVAVF
NVIPEWVPCVTKPQALYIGGPLNQQSVVGVGVTAQGVDAARVDNLTRLANRLVMVNLG
ADPEEIKPLVSGMRLFAGHAEWAPGQLAQEIENGDWFVAPALPSDVTAPGSVDVWGDVM

RRQPMPLPLYSTFPVNVGEN
>non-antioxidant_846
SNAMDPNVITVTSYANIAIIKYWGKENQAKMIPSTSSISLTLENMFTTTSVSFLPDTATSDQF
YINGILQNDEEHTKISAIIDQFRQPGQAFVKMETQNNMPTAAGLSSSSSGLSALVKACDQL
FDTQLDQKALAQKAKFASGSSSRSFFGPVAAWDKDSGAIYKVETDLKMAMIMLVLNAAK
KPISSREGMKLCRDTSTTFDQWVEQSAIDYQHMLTYLKTNNFEKVGQLTEANALAMHAT
TKTANPPFSYLTKESYQAMEAVKELRQEGFACYFTMDAGPNVKVLCLEKDLAQLAERLG
KNYRIIVSKTKDLPDV
>non-antioxidant_847
ASASTPHALLLISIDGLRADMLDRGITPNLSHLAREGVRARWMAPSYPSLTFPNHYTLVTG
LRPDHHGIVHNSMRDPTLGGFWLSKSEAVGDARWWGGEPVWVGVENTGQHAATWSWP
GSEAAIKGVRPSQWRHYQKGVRLDTRVDAVRGWLATDGAQRNRLVTLYFEHVDEAGHD
HGPESRQYADAVRAVDAAIGRLLAGMQRDGTRARTNIIVVSDHGMAEVAPGHAISVEDIA
PPQIATAITDGQVIGFEPLPGQQAAAEASVLGAHDHYDCWRKAELPARWQYGSHPRIPSLV
CQMHEGWDALFPDKLAKRAQRGTRGSHGYDPALPSMRAVFLAQGPDLAQGKTLPGFDN
VDVYALMSRLLGIPAAPNDGNPATLLPALRMPPAPDAR
>non-antioxidant_848
MSLTHRKFGGSGGSPFSGLSSIAVRSGSYLDAIIIDGVHHGGSGGNLSPTFTFGSGEYISNM
TIRSGDYIDNISFETNMGRRFGPYGGSGGSANTLSNVKVIQINGSAGDYLDSLDIYYEQY
>non-antioxidant_849
SIEFSTAITRQIVLDTETTGMNQIGAHYEGHKIIEIGAVEVVNRRLTGNNFHVYLKPDRLVD
PEAFGVHGIADEFLLDKPTFAEVADEFMDYIRGAELVIHNAAFDIGFMDYEFSLLKRDIPKT
NTFCKVTDSLAVARKMFPGKRNSLDALCARYEIDNSKRTLHGALLDAQILAEVYLAMTG
GQTSMATRESG
>non-antioxidant_850
GMNPFQNSFGGHIPQDVAGKQGENVIFIVYNLTDSPDTVDKVKDVCANFSAMIRSMRNRF
PDMQFSCTMGFGADAWTRLFPDKGKPKELSTFSEIKGEKYTAVSTPGDLLFHIRAKQMGL
CFEFASILDEKLKGAVVSVDETHGFRYMDGKAIIGFVDGTENPAVDENPYHFAVIGEEDAD
FAGGSYVFVQKYIHDMVAWNALPVEQQEKVIGRHKFNDVELSDEEKPGNAHNAVTNIGD
DLKIVRANMPFANTSKGEYGTYFIGYASTFSTTRRMLENMFIGSPAGNTDRLLDFSTAITGT
LFFVPSYDLLGELGE
>non-antioxidant_851
ESKQIQALRYYSAQGYSVINKYLRGDDYPETQAKETLLSRDYLSTNEPSDEEFKNAMSVY
INDIAEGLSSLPETDHRVVYRGLKLDKPALSDVLKEYTTIGNIIIDKAFMSTSPDKAWINDTI
LNIYLEKGHKGRILGDVAHFKGEAEMLFPPNTKLKIESIVNCGSQDFASQLSKLRLSDDAT
ADTNRIKRIINMRVLN
>non-antioxidant_852
MEFKDFPLKPEILEALHGRGLTTPTPIQAAALPLALEGKDLIGQARTGTGKTLAFALPIAER
LAPSQERGRKPRALVLTPTRELALQVASELTAVAPHLKVVAVYGGTGYGKQKEALLRGAD
AVVATPGRALDYLRQGVLDLSRVEVAVLDEADEMLSMGFEEEVEALLSATPPSRQTLLFSA
TLPSWAKRLAERYMKNPVLINVIK
>non-antioxidant_853
GHMGFFSRDIQTMEDLLLHGLRDIYYAEQQITKALPKMIEQATNRDLSQGLTSHLEETQKQ
IERLDQVFKKLGQKPSGVNCPAIDGLIKEADETAGEIADKTVLDAAIVANAQAVEHYEIAR

YGTLIAWAEELGHDDIVRFLTTNLNEEKAANTKLNTVALRKGVNRKAASGS
>non-antioxidant_854
MAHHHHHHMGGPPTIEELKREKIIPHVFPDENVDLTVDMYISFKSGKEVNHGNILDLAGT
GSVPRNIKFSEEPPEDYCYILFMIDPDFPSRRRPDGRDYVHWAVSGIKSKELVKGTDKNCIT
LLPYVGPSIKKGTGLHRISFILSLVKEENKGNVTGVPLYRGEHYITRVKFNNCQSAYNVIQ
MNDMKIVGFNWCQMRRK
>non-antioxidant_855
MSRKKMGLLVMAYGTPYKEEDIERYYTHIRRGRKPEPEMLQDLKDRYEAIGGISPLAQITE
QQAHNLEQHLNEIQDEITFKAYIGLAHIEPFIEDAVAEMHKDGITEAVSIVLAPHFSTFSVQS
YNKRAKEEAEKLGGLTITSVESWYDEPKFVTYWVDRVKETYASMPEDERENAMLIVSAH
SLPEKIKEFGDPYPDQLHESAKLIAEGAGVSEYAVGWQSEGNTPDPWLGPDVQDLTRDLF
EQKGYQAFVYVPVGFVADHLEVLYDNDYECKVVTDDIGASYYRPEMPNAKPEFIDALAT
VVLKKLGR
>non-antioxidant_856
SGFRKMAFPSGKVEGCMVQVTCGTTTLNGLWLDDTVYCPRHVICTAEDMLNPNYEDLLI
RKSNHSFLVQAGNVQLRVIGHSMQNCLLRLKVDTSNPKTPKYKFVRIQPGQTFSVLACYN
GSPSGVYQCAMRPNHTIKGSFLNGSCGSVGFNIDYDCVSFCYMHHMELPTGVHAGTDLE
GKFYGPFVDRQTAQAAGTDTTITLNVLAWLYAAVINGDRWFLNRFTTTLNDFNLVAMKY
NYEPLTQDHVDILGPLSAQTGIAVLDMCAALKELLQNGMNGRTILGSTILEDEFTPFDVVR
QCSGVTFQ
>non-antioxidant_857
ANIVGGIEYSINNASLCSVGFSVTRGATKGFVTAGHCGTVNATARIGGAVVGTFAARVFPG
NDRAWVSLTSAQTLLPRVANGSSFVTVRGSTEAAVGAAVCRSGRTTGYQCGTITAKNVTA
NYAEGAVRGLTQGNACMGRGDSGGSWITSAGQAQGVMSGGNVQSNGNNCGIPASQRSS
LFERLQPILSQYGLSLVTG
>non-antioxidant_858
MVSKGEENNMAIIKEFMRFKVRMEGSVNGHEFEIEGEGEGRPYEGFQTAKLKVTKGGPLP
FAWDILSPQFGYGSKAYVKHPADIPDYFKLSFPEGFKWERVMNFEDGGVVTVTQDSSLQD
GEFIYKVKLRGTNFPSDGPVMQKKTMGWEASSERMYPEDGALKGEIKMRLKLKDGGHY
TSEVKTTYKAKKPVQLPGAYIVGIKLDITSHNEDYTIVEQYERAEGRHSTGGMDELYK
>non-antioxidant_859
MAATEGVGEAAQGGEPGQPAQPPPQPHPPPPQQQHKEEMAAEAGEAVASPMDDGFVSLD
SPSYVLYRDRAEWADIDPVPQNDGPNPVVQIIYSDKFRDVYDYFRAVLQRDERSERAFKLT
RDAIELNAANYTVWHFRRVLLKSLQKDLHEEMNYITAIIEEQPKNYQVWHHRRVLVEWL
RDPSQELEFIADILNQDAKNYHAWQHRQWVIQEFKLWDNELQYVDQLLKEDVRNNSVW
NQRYFVISNTTGYNDRAVLEREVQYTLEMIKLVPHNESAWNYLKGILQDRGLSKYPNLLN
QLLDLQPSHSSPYLIAFLVDIYEDMLENQCDNKEDILNKALELCEILAKEKDTIRKEYWRYI
GRSLQSKHSTENDSPTNVQQEEF
>non-antioxidant_860
MASPSSFTYYCPPSSSPVWSEPLYSLRPEHARERLQDDSVETVTSIEQAKVEEKIQEVFSSY
KFNHLVPRLVLQREKHFHYLKRGLRQLTDAYECLDASRPWLCYWILHSLELLDEPIPQIVA
TDVCQFLELCQSPEGGFGGGPGQYPHLAPTYAAVNALCIIGTEEAYDIINREKLLQYLYSLK
QPDGSFLMHVGGEVDVRSAYCAASVASLTNIITPDLFEGTAEWIARCQNWEGGIGGVPGM
EAHGGYTFCGLAALVILKRERSLNLKSLLQWVTSRQMRFEGGFQGRCNKLVDGCYSFWQ

AGLLPLLHRALHAQGDPALSMSHWMFHQQALQEYILMCCQCPAGGLLDKPGKSRDFYH
TCYCLSGLSIAQHFGSGAMLHDVVLGVPENALQPTHPVYNIGPDKVIQATTYFLQKPVPG
FEELKDETSAEPATD
>non-antioxidant_861
DDPTASACVLS
>non-antioxidant_862
QAVGPPYTLCFECNRMTSSDCSTALRCYRGSCYTLYRPDENCELKWAVKGCAETCPTAGP
NERVKCCRSPRCNDD
>non-antioxidant_863
MNTYSITLPWPPSNNRYYRHNRGRTHVSAEGQAYRDNVARIIKNAMLDIGLAMPVKIRIE
CHMPDRRRRNLDNLQKAAFDALTKAGFWLDDAQVVDYRVVKMPVTKGGRLELTITEM
GNE
>non-antioxidant_864
MAPHGDGLSDIEEPEVDAQSEILRPISSVVFVIAMQAEALPLVNKFGLSETTDSPLGKGLP
WVLYHGVHKDLRINVVCPGRDAALGIDSVGTVPASLITFASIQALKPDIIINAGTCGGFKV
KGANIGDVFLVSDVVFHDRRIPIPMFDLYGVGLRQAFSTPNLLKELNLKIGRLSTGDSLDM
STQDETLIIANDATLKDMEGAAVAYVADLLKIPVVFLKAVTDLVDGDKPTAEEFLQNLTVV
TAALEGTATKVINFINGRNLSDL
>non-antioxidant_865
SMSYTWTGALITPCAAEESKLPINALSNSLLRHHNMVYATTSRSAGQRQKKVTFDRLQVL
DDHYRDVLKEMKAKASTVKAKLLSVEEACKLTPPHSAKSKYGYGAKDVRNLSSRAVNHI
HSVWKDLLEDTVTPIDTTIMAKNEVFCVQPEKGGRKPARLIVFPDLGVRVCEKMALYDV
VSTLPQVVMGSSYGFQYSPGQRVEFLVNTWKSKKNPMGFSYDTRCFDSTVTENDIRVEES
IYQCCDLAPEARQAIKSLTERLYIGGPLTNSKGQNCGYRRCRASGVLTTSCGNTLTCYLKA
SAACRAAKLQDCTMLVNGDDLVVICESAGTQEDAASLRVFTEAMTRYSAPPGDPPQPEY
DLELITSCSSNVSVAHDASGKRVYYLTRDPTTPLARAAWETARHTPVNSWLGNIIMYAPTL
WARMILMTHFFSILLAQEQLEKALDCQIYGACYSIEPLDLPQIIERLHGLSAFSLHSYSPGEI
NRVASCLRKLGVPPLRVWRHRARSVRARLLSQGGRAATCGKYLFNWAVKTKLKLTPIPAA
SQLDLSGWFVAGYSGGDIYHSLSRARPRHHHHHH
>non-antioxidant_866
STLEIAGLVRKNLVQFGVGEKNGSVRWVMNALGVKDDWLLVPSHAYKFEKDYEMMEFY
FNRGGTYYSISAGNVVIQSLDVGFQDVVLMKVPTIPKFRDITQHFIKKGDVPRALNRLATL
VTTVNGTPMLISEGPLKMEEKATYVHKKNDGTTVDLTVDQAWRGKGEGLPGMCGGALV
SSNQSIQNAILGIHVAGGNSILVAKLVTQEMFQNI
>non-antioxidant_867
MKVIFLKDVKGMGKKGEIKNVADGYANNFLFKQGLAIEATPANLKALEAQKQ
>non-antioxidant_868
MAHHHHHHLPALKLALEYIVPCMNKHGICVVDDFLGKETGQQIGDEVRALHDTGKFTDG
QLVSQKSDSSKDIRGDKITWIEGKEPGCETIGLLMSSMDDLIRHCNGKLGSYKINGRTKAM
VACYPGNGTGYVRHVDNPNGDGRCVTCIYYLNKDWDAKVSGGILRIFPEGKAQFADIEP
KFDRLLFFWSDRRNPHEVQPAYATRYAITVWYFDADERARAKVKYLTGEKGVRVELNKPS
DSVGKDVF
>non-antioxidant_869
GMLSNLESSIQSPKSGEYQCLAALNLYDSPECTSLATQAAVGRHLQVTSNQQGAAVEVCL

CEDDYPGWLSLGDLGLLKPATVLYQAKSFSESEIKKLLPGAIAFTQKAMQQSNYYLWGGT
VGPNYDCSGLMQAAFVSVGIWLPRDAYQQEAFTQAITIDELAPGDLVFFGTPVKATHVGL
YLGDGCYIHSSGKAQGRDGIGIDILSEQGDVVSRSYYQQLRGAGRVVKSYKPQRH
>non-antioxidant_870
SNRKTSCPIKINQFEGHFMKLQADSNYLLSKEYEELKDVGRNQSCDIALLPENRGKNRYN
NILPYDATRVKLSGGSDYINASYIPGNNFRREYIVTQGPLPGTKDDFWKMVWEQNVHNIV
MVTQCVEKGRVKCDHYWPADQDSLYYGDLILQMLSESVLPEWTIREFKICGEEQLDAHR
LIRHFHYTVWPDHGVPETTQSLIQFVRTVRDYINRSPGAGPTVVHCSAGVGRTGTFIALDR
ILQQLDSKDSVDIYGAVHDLRLHRVHMVQTECQYVYLHQCVRDVLRARKLR
>non-antioxidant_871
MHHHHHHLEVLFQGPSSTVTIEYFNQKKEMTKTLEEITRDFEKENPKIKVKVVNVPNAGE
VLKTRVLAGDVPDVVNIYPQSIELQEWAKAGVFEDLSNKDYLKRVKNGYAEKYAVNEKV
YNVPFTANAYGIYYNKDKFEELGLKVPETWDEFEQLVKDIVAKGQTPFGIAGADAWTLNG
YNQLAFATATGGGKEANQYLRYSQPNAIKLSDPIMKDDIKVMDILRINGSKQKNWEGAGY
TDVIGAFARGDVLMTPNGSWAITAINEQKPNFKIGTFMIPGKEKGQSLTVGAGDLAWSISA
TTKHPKEANAFVEYMTRPEVMQKYYDVDGSPTAIEGVKQAGEDSPLAGMTEYAFTDRHL
VWLQQYWTSEADFHTLTMNYVLTGDKQGMVNDLNAFFNPMKM
>non-antioxidant_872
GSHMSSSPAKDPPIQRLRGAVTRCEDGQLFISSYKNEYQTMEVQNNSVVIKCDGLYIIYLK
GSFFQEVKIDLHFREDHNPISIPMLNDGRRIVFTVVASLAFKDKVYLTVNAPDTLCEHLQIN
DGELIVVQLTPGYCAPEGSYHSTVNQVPL
>non-antioxidant_873
MTKERFVISRRRTGFGDCLWSLASAWSYAQRTGRTLVIDWRGSCYVEQPFSNAFPAFFEPV
EDIAGVPVICDDRVNQLSFPGPFFPRWWNRPSIDCINRPDEQIFRERDELTELFQAREDSEA
NTIVCDACLMWRCSEEAERLIFRNIKLRSEIRARIDALYEEHFSGHSIIGVHVRHGNGEDIM
EHAPYWADSELALHQVCMAIRKAKALSYPKPVKVFLCTDSAQVLDQVSGLFPDVFAVPK
RFQADRAGPLHSAEMGIEGGASALIDMYLLARCATVIRFPPTSAFTRYARLLVPRIIEFDLS
NPGHLTMIDNPYEHFAASHHHHHH
>non-antioxidant_874
KKMAFTLADRVTEEMLADKAALVVEVVEENYHDAPIVGIAVVNEHGRFFLRPETALADP
QFVAWLGDETKKKSMFDSKRAAVALKWKGIELCGVSFDLLLAAYLLDPAQGVDDVAAA
AKMKQYEAVRPDEAVYGKGAKRAVPDEPVLAEHLVRKAAAIWELERPFLDELRRNEQDR
LLVELEQPLSSILAEMEFAGVKVDTKRLEQMGKELAEQLGTVEQRIYELAGQEFNINSPKQ
LGVILFEKLQLPVLKKTKTGYSTSADVLEKLAPYHEIVENILHYRQLGKLQSTYIEGLLKV
VRPDTKKVHTIFNQALTQTGRLSSTEPNLQNIPIRLEEGRKIRQAFVPSESDWLIFAADYSQI
ELRVLAHIAEDDNLMEAFRRDLDIHTKTAMDIFQVSEDEVTPNMRRQAKAVNFGIVYGIS
DYGLAQNLNISRKEAAEFIERYFESFPGVKRYMENIVQEAKQKGYVTTLLHRRRYLPDITS
RNFNVRSFAERMAMNTPIQGSAADIIKKAMIDLNARLKEERLQAHLLLQVHDELILEAPK
EEMERLCRLVPEVMEQAVTLRVPLKVDYHYGSTWYDAK
>non-antioxidant_875
MKPEELVRHFGDVEKAAVGVGVTPGAVYQWLQAGEIPPLRQSDIEVRTAYKLKSDFTSQR
MGKEGHNSGTK
>non-antioxidant_876
SNAMTRYALLVRGINVGGKNKVVMAELRQELTNLGLEKVESYINSGNIFFTSIDSKAQLVE

KLETFFAVHYPFIQSFSLLSLEDFEAELENLPAWWSRDLARKDFLFYTEGLDVDQVIATVES
LELKDEVLYFGKLGIFWGKFSEESYSKTAYHKYLLKVPFYRHITIRNAKTFDKIGQMLKK
>non-antioxidant_877
ALCAFKDPYQQDLGIGESRISHENGTILCSKGSTCYGLWEKSKGDINLVKQGCWSHIGDPQ
ECHYEECVVTTTPPSIQNGTYRFCCCSTDLCNVNFTENF
>non-antioxidant_878
GYFEGMLIKQTDYFRIYRVINSLLISQNADPASASMYFSTFGAFILQQHYKVKAVPKGGLA
AYNLGGTVLLFADHREDGYVTGAGENFHCWVEADGWAIDFMAPAFSEGTDALAVPAKM
FQRPLSAMAASINDLGQSGDFFYRSEPEATARRFADWHKQAMIGDMASVAANWFRKSPK
QMAASLSVTDRDGKARTVPLTGEMLTGAW
>non-antioxidant_879
GSDLPAGWMRVQDTSGTYYWHIPTGTTQWEPPGRASPS
>non-antioxidant_880
KMTWTMKAAEEAEAVANINCSEHGRAFLDGIISEGSPKCECNTCYTGPDCSEKIQGCSAD
VASGDGLFLEEYWKQHKEASAVLVSPWHRMSYFFNPVSNFISFELEKTIKELHEVVGNAA
AKDRYIVFGVGVTQLIHGLVISLSPNMTATPDAPESKVVAHAPFYPVFREQTKYFDKKGYV
WAGNAANYVNVSNPEQYIEMVTSPNNPEGLLRHAVIKGCKSIYDMVYYWPHYTPIKYKA
DEDILLFTMSKFTGHSGSRFGWALIKDESVYNNLLNYMTKNTEGTPRETQLRSLKVLKEV
VAMVKTQKGTMRDLNTFGFKKLRERWVNITALLDQSDRFSYQELPQSEYCNYFRRMRPP
SPSYAWVKCEWEEDKDCYQTFQNGRINTQNGVGFEASSRYVRLSLIKTQDDFDQLMYYL
KDMVKAKRK
>non-antioxidant_881
MGSSHHHHHHSSGLVPRGSHMFTTKLAEKVVSAWKAKISQPALKAAQDGVIDTVAAALG
GVTEHSVQVALKYVAATGGSGDSKLWGVNQRSNMFDAAFVNGMAAHAIDFDDSFPVMR
GHPSSSLVPAIFAVGEHVGANGHNCLKSYVLGIEVVATLGRAVGKGHYLAGWHPTSTLGV
FGATTAAALLLGADEEQLRNAWGIAASNSCGIIKNFGTMTKPMHTGSAARNGVLSAWLS
MQSFTGCQTVFDDAEGILAMYGAQPGPELFNAMQKFGTPWAIIAPGLYKKSWPSCYANH
KPLAGLFAIMKEHGLTGQDISHVDVGFLPGVEKPLLYMDPRTTEEAKFSIEANIGAALLDG
EVSLASFEIEHLDRPAMRAAMKKVTRFDMPSETTFSGTTGYTDIVVHTADGKIERRIEATP
GSLEDPMDDAHLERKFKDCTAWMPFGESGLLFDRLRSLTADQGIKTVQP
>non-antioxidant_882
MGSSHHHHHHGSMNHYTRWLELKEQNPGKYARDIAGLMNIREAELAFARVTHDAWRM
HGDIREILAALESVGETKCICRNEYAVHEQVGTFTNQHLNGHAGLILNPRALDLRLFLNQW
ASVFHIKENTARGERQSIQFFDHQGDALLKVYATDNTDMAAWSELLARFITDENTPLELKA
VDAPVVQTRADATVVEQEWRAMTDVHQFFTLLKRHNLTRQQAFNLVADDLACKVSNSA
LAQILESAQQDGNEIMVFVGNRGCVQIFTGVVEKVVPMKGWLNIFNPTFTLHLLEESIAEA
WVTRKPTSDGYVTSLELFAHDGTQIAQLYGQRTEGEQEQAQWRKQIASLIPEGVAA
>non-antioxidant_883
MVRIVIDSGVDSGRPIGVVPFQWAGPGAAPEDIGGIVAADLRNSGKFNPLDRARLPQQPGS
AQEVQPAAWSALGIDAVVVGQVTPNPDGSYNVAYQLVDTGGAPGTVLAQNSYKVNKQW
LRYAGHTASDEVFEKLTGIKGAFRTRIAYVVQTNGGQFPYELRVSDYDGYNQFVVHRSPQ
PLMSPAWSPDGSKLAYVTFESGRSALVIQTLANGAVRQVASFPRHNGAPAFSPDGSKLAFA
LSKTGSLNLYVMDLASGQIRQVTDGRSNNTEPTWFPDSQNLAFTSDQAGRPQVYKVNIN
GGAPQRITWEGSQNQDADVSSDGKFMVMVSSNGGQQHIAKQDLATGGVQVLSSTFLDE

TPSLAPNGTMVIYSSSQGMGSVLNLVSTDGRFKARLPATDGQVKFPAWSPYLHHHHHH
>non-antioxidant_884
PVEEVMPVLEEKERSASYKPVFVTEITDDLHFYVQDVETGTQFQKLMENMRNDIASHPPV
EGSYAPRRGEFCIAKFVDGEWYRARVEKVESPAKIHVFYIDYGNREVLPSTRLGTLSPAFS
TRVLPAQATEYAFAFIQVPQDDDARTDAVDSVVRDIQNTQCLLNVEHLSAGCPHVTLQFA
DSKGDVGLGLVKEGLVMVEVRKEKQFQKVITEYLNAQESAKSARLNLWRYGDFRADDA
DEFGYSR
>non-antioxidant_885
PQITLWKRPLVTIKIGGQLKEALLDTGADDTIIEEMSLPGRWKPKMIGGIGGFIKVRQYDQII
IEIAGHKAIGTVLVGPTPVNIIGRNLLTQIGATLNF
>non-antioxidant_886
RQPEVRGGDTLNVFLAYVPEDAKAMMTTPFEAYLVNDSNYYLYYTYLSAEGKAWNNRS
HGLVEPNTKLLLEEFTKDVLNEMERVAVQLIAFKDGKPAAIKPAVSVELRIDTVKFYKLHT
FSASDFFEEPALIYDIVKDDVPAKQVYV
>non-antioxidant_887
MVANPPKPFEVHESGAYLHGTKAELKVGDRLVPGRESNFEAGRIMNHIYITQTLDAAVWG
AELAAGEGRGRIFIVEPEGAIEDDPNVTDKKLPGNPTRSYRTREPVWIVGELTDWVGHPPE
QLAAMRQGLEELRRKGLAVIYD
>non-antioxidant_888
MSHIQIPPGLTELLQGYTVEVLRQQPPDLVDFAVEYFTRLREARR
>non-antioxidant_889
MAGDPNSMTVSHHNASTARFYALRLLPGQEVFSQLHAFVQQNQLRAAWIAGCTGSLTDV
ALRYAGQEATTSLTGTFEVISLNGTLELTGEHLHLAVSDPYGVMLGGHMMPGCTVRTTLE
LVIGELPALTFSRQPCAISGYDELHISSRLEHHHH
>non-antioxidant_890
GMNPENWLLLRRVVRFGDTDAAGVMHFHQLFRWCHESWEESLESYGLNPADIFPGSRKS
EVTPEVALPIIHCQADFRRPIHTGDALAMELRPERLNPNSFQVHFEFRCEEQIAAHALIRHL
AINAQTRHRCALPEGIDRWLEASGVGKIGSI
>non-antioxidant_891
GHMAGNPPDPNAASTGTNPSPQAAGDISPEMMPETANIKLGYIPIVEAAPLIIAQEKGFFAK
YGMTGVEVSKQANWASARDNVTIGSQGGGIDGGQWQMPMPHLITEGIITNGNKVPMYV
LAQLITQGNGIAVAPMHEGKGVNLDITKAADYIKGFNKTNGRKFKAAHTFPNVNQDFWIR
YWFAAGGVDPDTDIDLLAVPPAETVQGMRNGTMDAFSTGDPWPYRIVTENIGYMAGLTA
QIWPYHPEEYLAIRADWVDKNPKATKALLKGIMEAQQWIDDPKNRPEVVQIVSGRNYFN
VPTTILESPFKGQYTMGDGQPAIDDFQKGPLYWKDGIGNVSYPYKSHDLWFLTESIRWGF
HKNAIPDLDTAQKIIDKVNREDLWREAATEAGFTADIPSSTSRGVETFFDGITFDPANPSAY
LQSLAIKKV
>non-antioxidant_892
GMSLAPWRGAIAHALHRNRSLVYARYLQLATVQPNGRPANRTLVFRGFLEDTNQLRFITD
TRSAKADQIQQQPWAEICWYFPNTREQFRMAGDLTLISSDDSHQDLQPARIAMWQELSDA
ARLQFGWPYPGKPRIKESGAFEPSPPDPIEPVPNFCLLLLDPVQVDHLELRGEPQNRWLYH
RNDQQEWSSEAINP
>non-antioxidant_893
GSVTSANLDHTKPCWYWDKKDLAHTPSQLEGLDPATEARYRREGARFIFDVGTRLGLHY

DTLATGIIYFHRFYMFHSFKQFPRYVTGACCLFLAGKVEETPKKCKDIIKTARSLLNDVQF
GQFGDDPKEEVMVLERILLQTIKFDLQVEHPYQFLLKYAKQLKGDKNKIQKLVQMAWTF
VNDSLCTTLSLQWEPEIIAVAVMYLAGRLCKFEIQEWTSKPMYRRWWEQFVQDVPVDVL
EDICHQILDLYSQGKQQMPH
>non-antioxidant_894
SNAIRSIWENNGFGLMSSKTMTDFDYWISDFEKIGASQKEAEQLIVKAIEIAIDANARNYN
YINAILKDWEQRGFKSVEEREA
>non-antioxidant_895
GSHMKNSVSVDLPGSMKVLVSKSSNADGKYDLIATVDALELSGTSDKNNGSGVLEGVKA
DASKVKLTISDDLGQTTLEVFKSDGSTLVSKKVTSKDKIIIIIKFNEKGEVSEKIITRADGTRL
EYTGIKSDGSGKAKEVLKGYVLEGTLTAEKTTLVVKEGTVTLSKNISKSGEVSVELNDTDS
SAATKKTAAWNSGTSTLTITVNSKKTKDLVFTSSNTITVQQYDSNGTSLEGSAVEITKLDEI
KNALK
>non-antioxidant_896
RSVRVLVDMDGVLADFEAGLLRGFRRRFPEEPHVPLEQRRGFLAREQYRALRPDLADKV
ASVYEAPGFFLDLEPIPGALDAVREMNDLPDTQVFICTSPLLKYHHCVGEKYRWVEQHLG
PQFVERIILTRDKTVVLGDLLIDDKDTVRGQEETPSWEHILFTCCHNRHLVLPPTRRRLLSW
SDNWREILDSKR
>non-antioxidant_897
MMFLRQEDFATVVRSTPLVSLDFIVENSRGEFLLGKRTNRPAQGYWFVPGGRVQKDETLE
AAFERLTMAELGLRLPITAGQFYGVWQHFYDDNFSGTDFTTHYVVLGFRFRVAEEELLLP
DEQHDDYRWLTPDALLASENVHANSRVYFNNDPRAIIGLNKKEVKNV
>non-antioxidant_898
SLEKQIESYYQEIAQLIIDMIPEEWAEVRFYAQEDHDGWKIFFFHYLSASSDEWTKDIDIRD
VIKVPQDEFMEKYNELSFCISDFRKDYAEAFGEPWMSFQMTFYASGKFNIDFYYDKNPFD
TFLTRLAWQYEHFGTIPEDSFYKETLNEYLEEKAQGKRYPFLEPLKEEEGHHHHHH
>non-antioxidant_899
GMTKAREFLGTGWKFPVAAGADGAMVLSSAEEDIAESIRIILGTARGERVMRPDFGCGIH
DRVFSVINTTTLGLIENEVKEALILWEPRIELLSVTASPREAAEGRLLIDIEYRVRSTNTRFNL
VYPFYLKESA
>non-antioxidant_900
GMAYTTTVKLDGDTKTYTLSPTVKKYTLMDLGFVKGRSGAFSFERSLDPTSPYQAAFKL
KMTVNADLTGFKMTTVTGNGVQRANIFKNDAHPEAVEQLRYILANFIERDILTTD
>non-antioxidant_901
MSAVKAARYGKDNVRVYKVHKDEKTGVQTVYEMTVCVLLEGEIETSYTKADNSVIVATD
SIKNTIYITAKQNPVTPPELFGSILGTHFIEKYNHIHAAHVNIVCHRWTRMDIDGKPHPHSFI
RDSEEKRNVQVDVVEGKGIDIKSSLSGLTVLKSTNSQFWGFLRDEYTTLKETWDRILSTD
VDATWQWKNFSGLQEVRSHVPKFDATWATAREVTLKTFAEDNSASVQATMYKMAEQIL
ARQQLIETVEYSLPNKHYFEIDLSWHKGLQNTGKNAEVFAPQSDPNGLIKCTVGRSSLKS
KL
>non-antioxidant_902
MGHHHHHHGSGTDIVLNDLPFVDGPPAEGQSRISWIKNGEEILGADTQYGSEGSMNRPTV
SVLRNVEVLDKNIGILKTSLETANSDIKTIQEAGYIPEAPRDGQAYVRKDGEWVLLSTFLG
SSGSGLVPR

>non-antioxidant_903
SMDRVFTTYKLMHTHQTVDFVRSKHAQFGGFSYKKMTVMEAVDLLDGLVDESDPDVDF
PNSFHAFQTAEGIRKAHPDKDWFHLVGLLHDLGKVLALFGEPQWAVVGDTFPVGCRPQA
SVVFCDSTFQDNPDLQDPRYSTELGMYQPHCGLDRVLMSWGHDEYMYQVMKFNKFSLP
PEAFYMIRFHSFYPWHTGRDYQQLCSQQDLAMLPWVREFNKFDLYTKCPDLPDVDKLRP
YYQGLIDKYCPGILSW
>non-antioxidant_904
GSTYPPTPPNVTRLSDESVMLRWMVPRNDGLPIVIFKVQYRMVGKRKNWQTTNDNIPYG
KPKWNSELGKSFTASVTDLKPQHTYRFRILAVYSNNDNKESNTSAKFYLQPGAALD
>non-antioxidant_905
GSHMSTLKEVQDNITLHEQRLVTTRQKLKDAERAVELDPDDVNKSTLQSRRAAVSALETK
LGELKRELADLIAAQKLA
>non-antioxidant_906
GSSGSSGMCGRFAQSQTREDYLALLAEDIERDIPYDPEPIGRYNVAPGTKVLLLSERDEHL
HLDPVFWGYAPGWWDKPPLINARVETAATSRMFKPLWQHGRAICFADGWFEWKKEGDK
KQPFFIYRADGQPIFMAAIGSTPFERGDEAEGFLIVTAAADQGLVDIHDRRPLVLSPEAARE
WMRQEISGKEASEIAASGCVPANQFSWHPVSRAVGNVKNQGAELIQPV
>non-antioxidant_907
GPLGSYGDAIPEVKAILEAKNEEELVTFTSRWSAEERKELRTQFQDTTGLEFIAFLKKCIKN
GPYEDVMALGWDCNISARVNVIKKAMKNVNDFRAIHDVVLIATPDERLKLAQAYKEKTG
NDLLQDFVDQIPLTSAASYLCHLAIRENRTPRGSVASDAEVLKHNLIDADEPDHEAVVRLII
TSTADEYKEINHRFEVLTGKSVQEAIETRYADKENARGLCIAHYYNLAPARAVAYAFHSAV
ETQNDDMAYEQAARITGLFHDLHKFAWVHYACWGVMRDDILSRFQSKEANKVNFRDAC
LMFWKLAK
>non-antioxidant_908
TIKEMPQPKTFGELKNLPLLNTDKPVQALMKIADELGEIFKFEAPGRVTRYLSSQRLIKEAC
DESRFDKNLSQALKFVRDFAGDGLFTSWTHEKNWKKAHNILLPSFSQQAMKGYHAMMV
DIAVQLVQKWERLNADEHIEVPEDMTRLTLDTIGLCGFNYRFNSFYRDQPHPFITSMVRAL
DEAMNKLQRANPDDPAYDENKRQFQEDIKVMNDLVDKIIADRKASGEQSDDLLTHMLNG
KDPETGEPLDDENIRYQIITFLIAGHETTSGLLSFALYFLVKNPHVLQKAAEEAARVLVDPV
PSYKQVKQLKYVGMVLNEALRLWPTAPAFSLYAKEDTVLGGEYPLEKGDELMVLIPQLH
RDKTIWGDDVEEFRPERFENPSAIPQHAFKPFGNGQRACIGQQFALHEATLVLGMMLKHF
DFEDHTNYELDIKETLTLKPEGFVVKAKSKKIPLGGIPSPSTEQSAKKV
>non-antioxidant_909
SPGQGTQSENSCTHFPGNLPNMLRDLRDAFSRVKTFFQMKDQLDNLLLKESLLEDFKGYL
GCQALSEMIQFYLEEVMPQAENQDPDIKAHVNSLGENLKTLRLRLRRCHRFLPCENKSKA
VEQVKNAFNKLQEKGIYKAMSEFDIFINYIEAYMTMKIRN
>non-antioxidant_910
MIVDFYFDFLSPFSYLANQRLSKLAQDYGLTIRYNAIDLARVKIAIGNVGPSNRDLKVKLD
YLKVDLQRWAQLYGIPLVFPANYNSRRMNIGFYYSGAEAQAAAYVNVVFNAVWGEGIAP
DLESLPALVSEKLGWDRSAFEHFLSSNAATERYDEQTHAAIERKVFGVPTMFLGDEMWW
GNDRLFMLESAMGRLCRQNADLSS
>non-antioxidant_911
MSLTFSILAHDPETGAIGGAAATGSLCVGGWVLRGDLNAGMSASQGAAPSTFWGEEVLQ

HLRDGSHPEDAVNHVTSQDSGRAYRQLAAMDLLGNAAAFTGSENQDIKGSVTFASGIAS
GNMLGDNSVLGAMTEAFVASDLTFERRLLAALIAAEGAGSDFRGLLSAAMLVLHPDRPP
VTLRIDYHPDNPIGALEQLYQKATTGDYADWARQVPVLSDKERILDEGHHHHHH
>non-antioxidant_912
MSSNAQVRPPLPPFTRESAIEKIRLAEDGWNSRDPERVSLAYTLDTQWRNRAEFAHNREE
AKAFLTRKWAKELDYRLIKELWAFTDNRIAVRYAYEWHDDSGNWFRSYGNENWEFDEQ
GLMARRFACINDMPIKAQERKFHWPLGRRPDDHPGLSELGLEHHHHHH
>non-antioxidant_913
GSPPAQLSVHTVSWNSGHERAPTNLEELLGLNSGETPDVIAVAVQGFGFQTDKPQQGPAC
VKNFQSLLTSKGYTKLKNTITETMGLTVYCLEKHLDQNTLKNETIIVTVDDQKKSGGIVTS
FTIYNKRFSFTTSRMSDEDVTSTNTKYAYDTRLDYSKKDDPSDFLFWIGDLNVRVETNATH
AKSLVDQNNIDGLMAFDQLKKAKEQKLFDGWTEPQVTFKPTYKFKPNTDEYDLSATPSW
TDRALYKSGTGKTIQPLSYNSLTNYKQTEHRPVLAKFRVTL
>non-antioxidant_914
EPALPSASEEQVAQDTEEVFRSYVFYRHQQEQEAEGVAAPADPEMVTLPLQPSSTMGQVG
RQLAIIGDDINRRYDSEFQTMLQHLQPTAENAYEYFTKIATSLFESGINWGRVVALLGFGYR
LALHVYQHGLTGFLGQVTRFVVDFMLHHCIARWIAQRGGWVAALNLGNG
>non-antioxidant_915
CLAEGTRIFDPVTGTTHRIEDVVDGRKPIHVVAAAKDGTLHARPVVSWFDQGTRDVIGLR
IAGGAILWATPDHKVLTEYGWRAAGELRKGDRVAVRDVETGELRYSVIREVLPTRRARTF
DLEVEELHTLVAEGVVVHN
>non-antioxidant_916
MSDTLPGTTPPDDNHDRPWWGLPCTVTPCFGARLVQEGNRLHYLADRAGIRGRFSDVDA
YHLDQAFPLLMKQLELMLTGGELNPRHQHTVTLYAKGLTCEADTLGSCGYVVYLAVYPTP
AAPATTVLEHHHHHH
>non-antioxidant_917
MNKTKSEHIKQQALDLFTRLQFLLQKHDTIEPYQYVLDILETGISKTKHNQQTPERQARVV
YNKIASQALVDKLHFTAEENKVLAAINELAHSQKGWGEFNMLDTTNTWPSQ
>non-antioxidant_918
MLLYMRFTENFERAKKEALMSLEIALRKGEVDEDIIPLLKKINSIENYFTTSSCSGRISVME
MPHFGDKVNAKWLGKWHREVSLYEVLEAIKKHRSGQLWFLVRSPILHVGAKTLEDAVKL
VNLAVSCGFKYSNIKSISNKKLIVEIRSTERMDVLLGENGEIFVGEEYLNKIVEIANDQMRR
FKEKLKRLESKINALNR
>non-antioxidant_919
MSAFFLNMEKSIITQKIIAKAFKDLMQSNAYHQISVSDIMQTAKIRRQTFYNYFQNQEELLS
WIFENDFAELINDNSDYYGWQNELLLLLRYLDENQIFYQKIFVIDKNFEHFFLIQWENLLD
KVIFDQEKKSDYHWSDLEKSFICRYNAAAICAITRESIIRGNSLEKLYSQIVNLLLAQIKIFES
LEHHHHHH
>non-antioxidant_920
GAMLYLIFYDITDDNLRNRVAEFLKKKGLDRIQYSVFMGDLNSSRLKDVEAGLKIIGNRK
KLQEDERFFILIVPITENQFRERIVIGYSGSEREEKSNVVW
>non-antioxidant_921
MIVAFCLYKYFPFGGLQRDFMRIASTVAARGHHVRVYTQSWEGDCPKAFELIQVPVKSHT
NHGRNAEYYAWVQNHLKEHPADRVVGFNKMPGLDVYFAADVCYAEKVAQEKGFLYRLT

SRYRHYAAFERATFEQGKSTKLMMLTDKQIADFQKHYQTEPERFQILPPGIYPDRKYSEQI
PNSREIYRQKNGIKEQQNLLLQVGSDFGRKGVDRSIEALASLPESLRHNTLLFVVGQDKPR
KFEALAEKLGVRSNVHFFSGRNDVSELMAAADLLLHPAYQEAAGIVLLEAITAGLPVLTTA
VCGYAHYIADANCGTVIAEPFSQEQLNEVLRKALTQSPLRMAWAENARHYADTQDLYSLP
EKAADIITGGLDG
>non-antioxidant_922
RPSSRVYIVEVLNEFPHDPYAFTQGLVYAENDTLFESTGLYGRSSVRQVALQTGKVENIHK
MDDSYFGEGLTLLNEKLYQVVWLKNIGFIYDRRTLSNIKNFTHQMKDGWGLATDGKILY
GSDGTSILYEIDPHTFKLIKKHNVKYNGHRVIRLNELEYINGEVWANIWQTDCIARISAKDG
TLLGWILLPNLRKKLIDEGFRDIDVLNGIAWDQENKRIFVTGKLWPKLFEIKLHLVRHRIPD
GYIERHCLNLRDNTLSLKTDID
>non-antioxidant_923
TQNFIIPKKEIHTVPDMGKWKRSQAYADYIGFILTLNEGVKGKKLTFEYRVSEAIEKLLALL
NTLDRWIDETPPVDQPSRFGNKAYRTWYAKLDEEAENLVATVVPTHLAAAVPEVAVYLKE
SVGNSTRIDYGTGHEAAFAAFLCCLCKIGVLRVDDQIAIVFKVFNRYLEVMRKLQKTYRM
EPAGSQGVWGLDDFQFLPFIWGSSQLIDHPYLEPRHFVDEKAVNENHKDYMFLECILFITE
MKTGPFAEHSNQLWNISAVPSWSKVNQGLIRMYKAECLEKFPVIQHFKFGSLLPIHPVTS
>non-antioxidant_924
MAPKAVLVGLPGSGKSTIGRRLAKALGVGLLDTDVAIEQRTGRSIADIFATDGEQEFRRIEE
DVVRAALADHDGVLSLGGGAVTSPGVRAALAGHTVVYLEISAAEGVRRTGGNTVRPLLA
GPDRAEKYRALMAKRAPLYRRVATMRVDTNRRNPGAVVRHILSRLQVPSPSEAATLEHHH
HHH
>non-antioxidant_925
HHHHHHGCMSGRKPIIGVMGPGKADTAENQLVMANELGKQIATHGWILLTGGRSLGVM
HEAMKGAKEAGGTTIGVLPGPDTSEISDAVDIPIVTGLGSARDNINALSSNVLVAVGMGPG
TAAEVALALKAKKPVVLLGTQPEAEKFFTSLDAGLVHVAADVAGAIAAVKQLLAKLN
>non-antioxidant_926
VGALAVVVWLWLWLWM
>non-antioxidant_927
GSHMRRRVRAILPYTKVPDTDEISFLKGDMFIVHNELEDGWMWVTNLRTDEQGLIVEDLV
EEVGR
>non-antioxidant_928
GSHMASTPDKFNDGNLNIAYAKPTTQSSVDYNGDPNRAVDGNRNGNFNSGSVTHTRADN
PSWWEVDLKKMDKVGLVKIYNRTDAETQRLSNFDVILYDNNRNEVAKKHVNNLSGESVS
LDFKEKGARYIKVKLLTSGVPLSLAEVEVFRES
>non-antioxidant_929
ASHHLRMHFKTLPAGESLGSLGLWVWGDVDQPSKDWPNGAITMTKAKKDDYGYYLDV
PLAAKHRQQVSYLINNKAGENLSKDQHISLLTPKMNEVWIDENYHAHAYRPLKEGYLRIN
YHNQSGHYDNLAVWTFKDVKTPTTDWPNGLDLSHKGHYGAYVDVPLKEGANEIGFLIL
DKSKTGDAIKVQPKDYLFKELDNHTQVFVKDTDPKVYNNPYYID
>non-antioxidant_930
MGSSHHHHHHMTLNQPEYFTKYENLHFHRDENGILEVRMHTNGSSLVFTGKTHREFPDA
FYDISRDRDNRVVILTGSGDAWMAEIDFPSLGDVTNPREWDKTYWEGKKVLQNLLDIEVP
VISAVNGAALLHSEYILTTDIILASENTVFQDMPHLNAGIVPGDGVHILWPLALGLYRGRYF

LFTQEKLTAQQAYELNVVHEVLPQSKLMERAWEIARTLAKQPTLNLRYTRVALTQRLKRL
VNEGIGYGLALEGITATDLRNT
>non-antioxidant_931
LVPRGSHMTIDQWLLKNAKEDAIAELKKAGITSDFYFNAINKAKTVEEVNALKNEILKAH
A
>non-antioxidant_932
MLYILNSAILPLKPGEEYTVKAKEITIQEAKELVTKEQFTSAIGHQATAELLSSILGVNVPM
NRVQIKVTHGDRILAFMLKQRLPEGVVVKTTEELEKIGYELWLFEIQ
>non-antioxidant_933
MDRSWMTGDFNGSVDIGGSITADDYRQKWEWKVGTGLNGFGNVLNDLTNGGTKLTITV
TGNKPILLGRTKEAFATPVTGGVDGIPHIAFTDYEGASVVLRKPDGETNKNGLAYFVLPMK
NAGGTKVGSVKVNASYAGVLGRGGVTSADGELLSLFADGLSSIFYGGLPRGSELSAGSAA
AARTKLFGSLSRDDILGQIQRVNANVTSLVDVAGSYRENMEYTDGNVVSAAYALGIANGQ
TIEATFNQAVTTSTQWSAPLNVAITYY
>non-antioxidant_934
SMSTLLINQPQYAWLKELGLREENEGVYNGSWGGRGEVITTYCPANNEPIARVRQASVAD
YEETVKKAREAWKIWADIPAPKRGEIVRQIGDALREKIQVLGSLVSLEMGKILVEGVGEVQ
EYVDICDYAVGLSRMIGGPILPSERSGHALIEQWNPVGLVGIITAFNFPVAVYGWNNAIAMI
CGNVCLWKGAPTTSLISVAVTKIIAKVLEDNKLPGAICSLTCGGADIGTAMAKDERVNLLS
FTGSTQVGKQVGLMVQERFGRSLLELGGNNAIIAFEDADLSLVVPSALFAAVGTAGQRCT
TARRLFIHESIHDEVVNRLKKAYAQIRVGNPWDPNVLYGPLHTKQAVSMFLGAVEEAKKE
GGTVVYGGKVMDRPGNYVEPTIVTGLGHDASIAHTETFAPILYVFKFQNEEEVFAWNNEV
KQGLSSSIFTKDLGRIFRWLGPKGSDCGIVNVNIPTSGAEIGGAFGGEKHTGGGRESGSDA
WKQYMRRSTCTINYS
>non-antioxidant_935
MGHHHHHHHHHHSSGHIEGRHMIRLGYPCENLTLGATTNRTLRLAHLTEERVREKAAEN
LRDLERILRFNADHGFALFRIGQHLIPFASHPLFPYDWEGAYEEELARLGALARAFGQRLS
MHPGQYVNPGSPDPEVVERSLAELRYSARLLSLLGAEDGVLVLHLGGAYGEKGKALRRF
VENLRGEEEVLRYLALENDERLWNVEEVLKAAEALGVPVVVDTLHHALNPGRLPLEEAL
RLAFPTWRGRPKVHLASQDPKKRPGAHAFRVTREDWERLLSALPGPADVMVEAKGKEQ
GLATP
>non-antioxidant_936
FTETTIVVHYHRYDGKYDGWNLWIWPVEPVSQEGKAYQFTGEDDFGKVAVVKLPMDLTK
VGIIVRLNEWQAKDVAKDRFIEIKDGKAEVWILQGVEEIFYEKP
>non-antioxidant_937
MLQCYNCPNPTADCKTAVNCSSDFDACLITKAGLQVYNKCWKFEHCNFNDVTTRLRENE
LTYYCCKKDLCNFNEQLENG
>non-antioxidant_938
MGSSHHHHHHSSGLVPRGSHMDVEKLRQLYAAGERDFSIVDLRGAVLENINLSGAILHGA
MLDEANLQQANLSRADLSGATLNGADLRGANLSKADLSDAILDNAILEGAILDEAVLNQA
NLKAANLEQAILSHANIREADLSEANLEAADLSGADLAIADLHQANLHQAALERANLTG
ANLEDANLEGTILEGGNNNLAT
>non-antioxidant_939
QFQKPGDAIEYRQSAFTLIANHFGRVAAMAQGKAPFDAKVAAENIALVSTLSKLPLTAFGP

GTDKGHGTEAKPAVWSDAAGFKAAADKFAAAVDKLDAAGKTGDFAQIKAAVGETGGAC
KGCHDKFKEK
>non-antioxidant_940
GSMKKVEAIIRPEKLEIVKKALSDAGYVGMTVSEVKGRGVQGGIVERYRGREYIVDLIPK
VKIELVVKEEDVDNVIDIICENARTGNPGDGKIFVIPVERVVRVRTKEEGKEALLEHHH
>non-antioxidant_941
HHMGNLNRCIADIVSLFITVMDKLRLEIRAMDEIQPDLRELMETMNRMSHLPPDFEGREK
VSQWLQKLSSMSASDELDDSQVRQMLFDLESAYNAFNRFLHS
>non-antioxidant_942
MGSSHHHHHHSSGLVPRGSMSTSWSDRLQNAADMPANMDKHALKKYRREAYHRVFVN
RSLAMEKIKCFGFDMDYTLAVYKSPEYESLGFELTVERLVSIGYPQELLSFAYDSTFPTRGL
VFDTLYGNLLKVDAYGNLLVCAHGFNFIRGPETREQYPNKFIQRDDTERFYILNTLFNLPE
TYLLACLVDFFTNCPRYTSCETGFKDGDLFMSYRSMFQDVRDAVDWVHYKGSLKEKTVE
NLEKYVVKDGKLPLLLSRMKEVGKVFLATNSDYKYTDKIMTYLFDFPHGPKPGSSHRPW
QSYFDLILVDARKPLFFGEGTVLRQVDTKTGKLKIGTYTGPLQHGIVYSGGSSDTICDLLG
AKGKDILYIGDHIFGDILKSKKRQGWRTFLVIPELAQELHVWTDKSSLFEELQSLDIFLAEL
YKHLDSSSNERPDISSIQRRIKKVTHDMDMCYGMMGSLFRSGSRQTLFASQVMRYADLYA
ASFINLLYYPFSYLFRAAHVLMPHESTVEHTHVDINEMESPLATRNRTSVDFKDTDYKRH
QLTRSISEIKPPNL
>non-antioxidant_943
MAHHHHHHVREEKLRLRKQIIEHMNSLSKERYTTLSEQIVFSLYEQKEWAEAKTIGITLSM
ENEVNTYPIIEKAWKEGKRVVVPKCNKETRTMSFRQISNFDQLETVYMNLREPIPALTEEV
NADEIDLQIVPGVAYTERGERIGYGGGYYDRYLVHYKGKTLSLAYSFQMVEHIPVEPFDK
NVEKIITEKGTMVKNGLV
>non-antioxidant_944
MNQIDLNVTCRYAGVFHVEKNGRYSISRTEAADLCQAFNSTLPTMDQMKLALSKGFETC
RYGFIEGNVVIPRIHPNAICAANHTGVYILVTSNTSHYDTYCFNASAPPEEDCTSVTDLPNS
FDGPVTITIVNRDGTRYSKKGEYRTHQEDIDAS
>non-antioxidant_945
GHMSSTPSNQNIIPIIKKESIVSLFEKGIRQDGRKLTDYRPLSITLDYAKKADGSALVKLGTT
MVLAGTKLEIDKPYEDTPNQGNLIVNVELLPLAYETFEPGPPDENAIELARVVDRSLRDSK
ALDLTKLVIEPGKSVWTVWLDVYVLDYGGNVLDACTLASVAALYNTKVYKVEQHSNGIS
VNKNEVVGKLPLNYPVVTISVAKVDKYLVVDPDLDEESIMDAKISFSYTPDLKIVGIQKSG
KGSMSLQDIDQAENTARSTAVKLLEELKKHLGI
>non-antioxidant_946
GHMREMLQVERPKLILDDGKRTDGRKPDELRSIKIELGVLKNADGSAIFEMGNTKAIAAV
YGPKEMHPRHLSLPDRAVLRVRYHMTPFSTDERKNPAPSRREIELSKVIREALESAVLVELF
PRTAIDVFTEILQADAGSRLVSLMAASLALADAGIPMRDLIAGVAVGKADGVIILDLNETE
AMWGEADMPIAMMPSLNQVTLFQLNGSMTPDEFRQAFDLAVKGINIIYNLEREALKSKY
VEFKEEGV
>non-antioxidant_947
MKSASDPFDLKRFVYAQAPVYRSVVEELRAGRKRGHWMWFVFPQLRGLGSSPLAVRYGI
SSLEEAQAYLQHDLLGPRLHECTGLVNQVQGRSIEEIFGPPDDLKLCSSMTLFARATDANQ
DFVALLAKYYGGGEDRRTVALLAVT

>non-antioxidant_948
GMASVLSAATATDQGPVRENNQDACLADGILYAVADGFGARGHHASATALKTLSAGFAA
APDRDGLLEAVQQANLRVFELLGDEPTVSGTTLTAVAVFEPGQGGPLVVNIGDSPLYRIRDG
HMEQLTDDHSVAGELVRMGEITRHEARWHPQRHLLTRALGIGPHIGPDVFGIDCGPGDRL
LISSDGLFAAADEALIVDAATSPDPQVAVRRLVEVANDAGGSDNTTVVVIDLG
>non-antioxidant_949
EETPVTPQPPDILLGPLFNDVQNAKLFPDQKTFADAVPNSDPLMILADYRMQQNQSGFDL
RHFVNVNFTLPKEGEKYVPPEGQSLREHIDGLWPVLTRSTENTEKWDSLLPLPEPYVVPG
GRFREVYYWDSYFTMLGLAESGHWDKVADMVANFAHEIDTYGHIPNGNRSYYLSRSQPP
FFALMVELLAQHEGDAALKQYLPQMQKEYAYWMDGVENLQAGQQEKRVVKLQDGTLL
NRYWDDRDTPRPESWVEDIATAKSNPNRPATEIYRDLRSAAASGWDFSSRWMDNPQQLN
TLRTTSIVPVDLNSLMFKMEKILARASKAAGDNAMANQYETLANARQKGIEKYLWNDQ
QGWYADYDLKSHKVRNQLTAAALFPLYVNAAAKDRANKMATATKTHLLQPGGLNTTSV
KSGQQWDAPNGWAPLQWVATEGLQNYGQKEVAMDISWHFLTNVQHTYDREKKLVEKY
DVSTTGTGGGGGEYPLQDGFGWTNGVTLKMLDLICPKEQPCDNVPATRPTVKSATTQPST
KEAQPTP
>non-antioxidant_950
STAGKVIKCKAAVLWEEKKPFSIEEVEVAPPKAHEVRIKMVATGICRSDDHVVSGTLVTPLP
VIAGHEAAGIVESIGEGVTTVRPGDKVIPLFTPQCGKCRVCKHPEGNFCLKNDLSMPRGT
MQDGTSRFTCRGKPIHHFLGTSTFSQYTVVDEISVAKIDAASPLEKVCLIGCGFSTGYGSAV
KVAKVTQGSTCAVFGLGGVGLSVIMGCKAAGAARIIGVDINKDKFAKAKEVGATECVNP
QDYKKPIQEVLTEMSNGGVDFSFEVIGRLDTMVTALSCCQEAYGVSVIVGVPPDSQNLSM
NPMLLLSGRTWKGAIFGGFKSKDSVPKLVADFMAKKFALDPLITHVLPFEKINEGFDLLRS
GESIRTILTF
>non-antioxidant_951
IVGGQECKDGECPWQALLINEENEGFCGGTILSEFYILTAAHCLYQAKRFKVRVGDRNTEQ
EEGGEAVHEVEVVIKHNRFTKETYDFDIAVLRLKTPITFRMNVAPACLPERDWAESTLMTQ
KTGIVSGFGRTHEKGEQSTRLKMLEVPYVDRNSCKLSSSFIITQNMFCAGYDTKQEDACQ
GDSGGPHVTRFKDTYFVTGIVSWGEGCARKGKYGIYTKVTAFLKWIDRSMKTRGLPKAK
>non-antioxidant_952
EIKREYKEMEGSPEIKSKRRQFHQEIQSRNMRENVKRSSVVVANPTHIAIGILYKRGETPLP
LVTFKYTDAQVQTVRKIAEEEGVPILQRIPLARALYWDALVDHYIPAEQIEATAEVLRWLE
>non-antioxidant_953
RSWHYVEPKFLNKAFEVALKVQIIAGFDRGLVKWLRVHGRTLSTVQKKALYFVNRRYMQ
THWANYMLWINKKIDALGRTPVVGDYTRLGAEIGRRIDMAYFYDFLKDKNMIPKYLPYM
EEINRMRPADVPVKYMGK
>non-antioxidant_954
APGVTVTPATGLSNGQTVTVSATGLTPGTVYHVGQCAVVEPGVIGCDATTSTDVTADAAG
KITAQLKVHSSFQAVVGADGTPWGTVNCKVVSCSAGLGSDSGEGAAQAITFA
>non-antioxidant_955
GWEIPEPYVWDESFRVFYEQLDEEHKKIFKGIFDCIRDNSAPNLATLVKVTTNHFTHEEAM
MDAAKYSEVVPHKKMHKDFLEKIGGLSAPVDAKNVDYCKEWLVNHIKGTDFKYKGKL
>non-antioxidant_956
EDDLYRQSLEIISRYLREQATGSKDSKPLGEAGAAGRRALETLRRVGDGVQRNHETAFQG

MLRKLDIKNEDDVKSLSRVMIHVFSDGVTNWGRIVTLISFGAFVAKHLKTINQESCIEPLA
ESITDVLVRTKRDWLVKQRGWDGFVEFFHVEDLEGG
>non-antioxidant_957
DTTICEPFGTTTIQGRYVVQNNRWGSTAPQCVTATDTGFRVTQADGSAPTNGAPKSYPSVF
NGCHYTNCSPGTDLPVRLDTVSAAPSSISYGFVDGAVYNASYDIWLDPTARTDGVNQTEI
MIWFNRVGPIQPIGSPVGTASVGGRTWEVWSGGNGSNDVLSFVAPSAISGWSFDVMDFVR
ATVARGLAENDWYLTSVQAGFEPWQNGAGLAVNSFSSTVETGTPGGTDPGDPG
>non-antioxidant_958
DHYNCVSSGGQCLYSACPIFTKIQGTCYRGEAKCCK
>non-antioxidant_959
GMDKLVKYQELVKKLLTNYASDDVSDQDVEVQLILDTERNHYQWMNVGWQGLNRIYR
CVIHFDIKDGKIWLQQNLTDRNPAEELVMMGVPREDIVLGLQAPYKRQYTDYGVA
>non-antioxidant_960
MSHTILLVQPTKRPEGRTYADYESVNECMEGVCKMYEEHLKRMNPNSPSITYDISQLFDFI
DDLADLSCLVYRADTQTYQPYNKDWIKEKIYVLLRRQAQQAGK
>non-antioxidant_961
GSGSMETLCQRLNVCQDKILTHYENDSTDLRDHIDYWKHMRLECAIYYKAREMGFKHIN
HQVVPTLAVSKNKALQAIELQLTLETIYNSQYSNEKWTLQDVSLEVYLTAPTGCIKKHGYT
VEVQFDGDICNTMHYTNWTHIYICEEASVTVVEGQVDYYGLYYVHEGIRTYFVQFKDDA
EKYSKNKVWEVHAGGQVILCPTSVFS
>non-antioxidant_962
GSATIDMNFQSDLLSIFEENLF
>non-antioxidant_963
MQNRQVANATKVAVAGASGYAGGEILRLLLGHPAYADGRLRIGALTAATSAGSTLGEHHP
HLTPLAHRVVEPTEAAVLGGHDAVFLALPHGHSAVLAQQLSPETLIIDCGADFRLTDAAVW
ERFYGSSHAGSWPYGLPELPGARDQLRGTRRIAVPGCYPTAALLALFPALAADLIEPAVTV
VAVSGTSGAGRAATTDLLGAEVIGSARAYNIAGVHRHTPEIAQGLRAVTDRDVSVSFTPVL
IPASRGILATCTARTRSPLSQLRAAYEKAYHAEPFIYLMPEGQLPRTGAVIGSNAAHIAVAVD
EDAQTFVAIAAIDNLVKGTAGAAVQSMNLALGWPETDGLSVVGVAP
>non-antioxidant_964
SNAEEELPFKVLGDGSYLFEGKTSLSDVRHYLDLPENAFGELGDEVDTLSGLFLEIKQELP
HVGDTAVYEPFRFQVTQMDKRRIIEIKIFPFE
>non-antioxidant_965
SNAMANVKLLLPYILKWEGGFVHDPADAGGATNKGVTIATWKRVGYDKDGDGDIDVED
LKLLTDDDVLNRVLKPFYWDRWKADLIESQKVANILVDWVWGSGKYGIVIPQRILGVQA
DGIVGNKTLQAVNSADPDELFESIFDARREFLEDITARSIKKYEDSIGRKATERELLRHTNK
RFLRGWLNRLEDIRKL
>non-antioxidant_966
MEALEELMKLLNMKDFPYRIEGIDISHLQGKYTVASLVVFEDGFPKKGDYRRYKIEQDHP
DDYESIRTVVKRRYSKHPLPNLLFVDGGIGQVNAAIEALKEIGKDCPVVGLAKKEETVVFE
NREIHLPHDHPVLRLLVQIRDETHRFAVSYHRKRREKE
>non-antioxidant_967
QPVNHLVKEIDMLLKEYLLSGDISEAEHCLKELEVPHFHHELVYEAIVMVLESTGESAFK
MILDLLKSLWKSSTITIDQMKRGYERIYNEIPDINLDVPHSYSVLERFVEECFQAGIISKQLR

DLCPSR
>non-antioxidant_968
MASDVYLIFSTCPDLPSAEIISRVLVQERLAACVTQLPGAVSTYRWQGKIETTQEIQLLIKTN
AVHVNAAITRLCALHPYRLPEAIAVQVSVGLPEYLTWINTEIDEEYSLPHHHHHH
>non-antioxidant_969
APVRSLNCTLRDSQQKSLVMSGPYELKALHLQGQDMEQQVVFSMSFVQGEESNDKIPVA
LGLKEKNLYLSCVLKDDKPTLQLESVDPKNYPKKKMEKRFVFNKIEINNKLEFESAQFPN
WYISTSQAENMPVFLGGTKGGQDITDFTMQFVSS
>non-antioxidant_970
MPVDKNLRDLEPGIHTDLEGRLTYGGYLRLDQLLSAQQPLSEPAHHDEMLFIIQHQTSEL
WLKLLAHELRAAIVHLQRDEVWQCRKVLARSKQVLRQLTEQWSVLETLTPSEYMGFRD
VLGPSSGFQSLQYRYIEFLLGNKNPQMLQVFAYDPAGQARLREVLEAPSLYEEFLRYLARF
GHAIPQQYQARDWTAAHVADDTLRPVFERIYENTDRYWREYSLCEDLVDVETQFQLWRF
RHMRTVMRVIGFKRGTGGSSGVGFLQQALALTFFPELFDVRTSVGVDNRPPQGSADAGK
RLEHHHHHH
>non-antioxidant_971
LASIFVDVSSVEPGVQLTVKFLGKPIFIRRRTEADIELGRSVQLGQLVDTNARNANIDAGAE
ATDQNRTLDEAGEWLVMWGVCTHLGCVPIGGVSGDFGGWFCPCHGSHWDSAGRIRKGP
APENLPIPLAKFIDETTIQLG
>non-antioxidant_972
MKPVPTYVQDKDESTLMFSVCSLVRDQAKYDRLLESFERFGFTPDKAEFLAADNREGNQ
FHGFSWHKQMLPRCKGRYVIFCHEDVELVDRGYDDLVAAIEALEEADPKWLVAGVAGSP
WRPLNHSVTAQALHISDVFGNDRRRGNVPCRVESLDECFLLMRRLKPVLNSYDMQGFHY
YGADLCLQAEFLGGRAYAIDFHLHHYGRAIADENFHRLRQEMAQKYRRWFPGRILHCVT
GRVALGGGWYEAR
>non-antioxidant_973
MDKNELVQKAKLAEQAERYDDMAACMKSVTEQGAELSNEERNLLSVAYKNVVGARRSS
WRVVSSIEQKTEGAEKKQQMAREYREKIETELRDICNDVLSLLEKFLIPNASQAESKVFYL
KMKGDYYRYLAEVAAGDDKKGIVDQSQQAYQEAFEISKKEMQPTHPIRLGLALNFSVFY
YEILNSPEKACSLAKTAFDEAIAELDTLSEESYKDSTLIMQLLRDNLTLWTS
>non-antioxidant_974
GASTVEKELLRSRRLENSIIEQKGTMRCYAYVMEQNLPENLLFDYENGVITQGLSEHVYK
FNRVIPHLKVSEDKFFTQEYSVYHDMCLNQKKNFNLISLSTTPHGSLRESLIKFLAEKDTIY
QKQYVITLQFVFLSDDEFSQDMLLDYSHNDKDSIKLKFEKHSISLDSKLVIIENGLEDLPLN
FSCDEHPNLPHSGMGIIKVQFFPRDSKSDGNNDPVPVDFYFIELNNLKSIEQFDKSIFKKES
CETPIALVLKKLISDTKSFFLLNLNDSKNVNKLLTISEEVQTQLCKRKKKLT
>non-antioxidant_975
MKTWPAPTAPTPVRATVTVPGSKSQTNRALVLAALAAAQGRGASTISGALRSRDTELMLD
ALQTLGLRVDGVGSELTVSGRIEPGPGARVDCGLAGTVLRFVPPLAALGSVPVTFDGDQQ
ARGRPIAPLLDALRELGVAVDGTGLPFRVRGNGSLAGGTVAIDASASSQFVSGLLLSAASF
TDGLTVQHTGSSLPSAPHIAMTAAMLRQAGVDIDDSTPNRWQVRPGPVAARRWDIEPDLT
NAVAFLSAAVVSGGTVRITGWPRVSVQPADHILAILRQLNAVVIHADSSLEVRGPTGYDGF
DVDLRAVGELTPSVAALAALASPGSVSRLSGIAHLRGHETDRLAALSTEINRLGGTCRETP
DGLVITATPLRPGIWRAYADHRMAMAGAIIGLRVAGVEVDDIAATTKTLPEFPRLWAEMVG

PGQGWGYPQPRSGQRARRATGQGSGG
>non-antioxidant_976
GMLKSKIKEEYVQMDQVDWKPFPAAFSTGGIRWKLLHVSPEMGSWTAIFDCPAGSSFAA
HVHVGPGEYFLTKGKMDVRGGKAAGGDTAIAPGYGYESANARHDKTEFPVASEFYMSFL
GPLTFVKPDGSPIAVIGWEDAQGAWAA
>non-antioxidant_977
MKERRYLPLSQARKSGFQMDWLSEPHPVKPTFIGTQVFEEYDLQKLVDYIDWKPFFDVW
QLRGKYPNRGFPKIFNDKTVGGEARKVYDDAHNMLNTLISQKKLRARGVVGFWPAQSIQ
DDIHLYAEAAVPQAAEPIATFYGLRQQAENDSASTEPYYCLSDFIAPLHSGIRDYLGLFAVA
CFGVEELSKAYEDDGDDYSSIMVKALGDRLAEAFAEELHERVRRELWAYCGSEQLDVAD
LRRLRYKGIRPAPGYPSQPDHTEKLTMWRLADIEQSTGIRLTESLAMAPASAVSGLYFSNLK
SKYFAVGKISKDQVEDYALRKNISVAEVEKWLGPILGYDTDKLAAALEHHHHHH
>non-antioxidant_978
GMADKTGTPHPLTEPGVWIERIGGRVFPPHLPALFLDRDGTINVDTDYPSDPAEIVLRPQM
LPAIATANRAGIPVVVVTNQSGIARGYFGWSAFAAVNGRVLELLREEGVFVDMVLACAYH
EAGVGPLAIPDHPMRKPNPGMLVEAGKRLALDLQRSLIVGDKLADMQAGKRAGLAQGW
LVDGEAAVQPGFAIRPLRDSSELGDLLAAIETLGRDNRS
>non-antioxidant_979
MAEIEQAKKEIAYLIKKAKEEILEEIKKAKQEIAM
>non-antioxidant_980
GSDSGTLNYEVYKYNTNDTSIANDYFNKPAKYIKKNGKLYVQITVNHSHWITGMSIEGHK
ENIISKNTAKDERTSEFEVSKLNGKIDGKIDVYIDEKVNGKPFKYDHHYNITYKFNGPTDV
AGANAPGKDDKNSASGSDKGSDGTTTGQSESNSSNKDKVE
>non-antioxidant_981
CPSRCSCSGTTVECYSQGRTSVPTGIPAQTTYLDLETNSLKSLPNGVFDELTSLTQLYLGGN
KLQSLPNGVFNKLTSLTYLNLSTNQLQSLPNGVFDKLTQLKELALNTNQLQSLPDGVFDKL
TQLKDLRLYQNQLKSVPDGVFDRLTSLQYIWLHDNPWDCTCPGIRYLSEWINKHSGVVR
NSAGSVAPDSAKCSGSGKPVRSIICP
>non-antioxidant_982
MKDEAEKLFNQDVDAAVRGILRNAKLKPVYDSLDAVRRAALINMVFQMGETGVAGFTN
SLRMLQQKRWDEAAVNLAKSRWYNQTPNRAKRVITTFRTGTWDAYKNLSGGGGAMDIF
EMLRIDEG
>non-antioxidant_983
MDIVFIEQLSVITTIGVYDWEQTIEQKLVFDIEMAWDNRKAAKSDDVADCLSYADIAETVV
SHVEGARFALVERVAEEVAELLLARFNSPWVRIKLSKPGAVARAANVGVIIERGNNLKENN
>non-antioxidant_984
MALSMTGGQQMGRGSMSRDPLPFFPPLYLGGPEITTENCEREPIHIPGSIQPHGALLTADGH
SGEVLQMSLNAATFLGQEPTVLRGQTLAALLPEQWPALQAALPPGCPDALQYRATLDWP
AAGHLSLTVHRVGELLILEFEPTEAWDSTGPHALRNAMFALESAPNLRALAEVATQTVREL
TGFDRVMLYKFAPDATGEVIAEARREGLHAFLGHRFPASDIPAQARALYTRHLLRLTADTR
AAAVPLDPVLNPQTNAPTPLGGAVLRATSPMHMQYLRNMGVGSSLSVSVVVGGQLWGLI
ACHHQTPYVLPPDLRTTLESLGRLLSLQVQVKEAHHHHHH
>non-antioxidant_985
GIDPFTGEAIAKFNFNGDTQVEMSFRKGERITLLRQVDENWYEGRIPGTSRQGIFPITYVD

VIKRPL
>non-antioxidant_986
MGEWEIIDIGPFTQNLGKFAVDEENKIGQYGRLTFNKVIRPCMKKTIYENEGFREIKGYEY
QLYVYASDKLFRADISEDYKTRGRKLLRFNGPVPPP
>non-antioxidant_987
GSLHEEADHRVTDHGPRPFVVNIEDETKRNRAFRRALWTGDHLQVTLMSIQVGEDIGLEI
HPHLDQFLRVEEGRGLVQMGHRQDNLHFQEEVFDDYAILIPAGTWHNVRNTGNRPLKLY
SIYAPPQHPHGTVHETKAIAMAAEEHHHL
>non-antioxidant_988
DRINTVRGPITISEAGFTLTHEHICGSSAGFLRAWPEFFGSRKALAEKAVRGLRRARAAGVR
TIVDVSTFDIGRDVSLLAEVSRAADVHIVAATGLWFDPPLSMRLRSVEELTQFFLREIQYGI
EDTGIRAGIIKVATTGKATPFQELVLKAAARASLATGVPVTTHTAASQRDGEQQAAIFESEG
LSPSRVCIGHSDDTDDLSYLTALAARGYLIGLDHIPYSAIGLEDNASASALLGIRSWQTRAL
LIKALIDQGYMKQILVSNDWTFGFSSYVTNIMDVMDRVNPDGMAFIPLRVIPFLREKGVPQ
ETLAGITVTNPARFLSPTLRA
>non-antioxidant_989
YFQGMLKNIDPALNADVLHALRAMGHGDTLVISDTNFPSDSVARQTTVGKVLHIDNVSA
ARAMKAILSVLPLDTPLQPSVGRMEVMGAPDQLEPVQVEVQQEIDAAEGKSAPMYGIER
FAFYEKAKQAYCVITTGETRFYGCFLLTKGVIPPK
>non-antioxidant_990
MMSGAPSATQPATAETQHIADQVRSQLEEKYNKKFPVFKAVSFKSQVVAGTNYFIKVHVG
DEDFVHLRVFQSLPHENKSLTLSNYQTNKAKHDELTYF
>non-antioxidant_991
MKIWSKEEVVNKLHEIKNKGYLSVPTDMFRTDDGVVGQILERQFGVQENNITLGDLGEFE
LKGMRNRKAKSNLTLFHKKPVAGQTVIQIFNRFGYVKPSSRNPEVMKKKLFTTIKGGRLN
NLGLTLNAKHASEINLYYQDEYLSTWDLNLSKIEKLVLVFAETIGRANSPEEQFHFTKAYM
LTEINDITSLINDGVLVMDLCIDQDLSKSKGPHDRGPHLRIPISKLDKLYRNIERLL
>non-antioxidant_992
GHMHVWPVQDAKARFSEFLDACITEGPQIVSRRGAEEAVLVPIGEWRRLQAAARPSLKQL
LLSDSARTEMLVPERGKARRRQVEPLRGS
>non-antioxidant_993
MTYKITVRVYQTNPNAFFHPVEKTVWKYANGGTWTITDDQHVLTMGGSGTSGTLRFHA
DNGESFTATFGVHNYKRWCDIVTNLAADETGMVINQQYYSQKNREEARERQLSNYEVKN
AKGRNFEIVYTEAEGNDLHANLIIG
>non-antioxidant_994
MQRCDWVSQDPLYIAYHDNEWGVPETDSRKLFEMICLEGQQAGLSWITVLKKRENYRAC
FHQFDPIRIAAMQEEDVERLLQNTGIIRHRGKIQAIISNARAWLAMEQNGESFADFVWSFV
DGQPQITQAASLDKIPTSTPASDALAKALKKRGFKFVGTTICYSFMQACGLVNDHITGCFC
HP
>non-antioxidant_995
MGSDKIHHHHHHNTASWFTALTQHGKEELRFPRGQGVPINTNSGPDDQIGYYRRATRRVR
GGDGKMKELSPRWYFYYLGTGPEASLPYGANKEGIVWVATEGALNTPKDHIGTRNPNNN
AATVLQLPQGTTLPKGFYA
>non-antioxidant_996

SLSEDKMDLYLQQGMYGPLETKPDERHLFLGSLRERVVLALTKGQVLRSKPYKEAEHEL
KNSHNVTLLINGELQYQSYSSYIQMASRYGVPFKIVSDLQFHTPLGIVIAADIAVNRELIYIQ
DDIYNRSVLKS
>non-antioxidant_997
REVLTGGHSVSAPQENRIYVMDSVFMHLTESRVHVYDYTNGKFLGMVPTAFNGHVQVSN
DGKKIYTMTTYHERITRGKRSDVVEVWDADKLTFEKEISLPPKRVQGLNYDGLFRQTTDG
KFIVLQNASPATSIGIVDVAKGDYVEDVTAAAGCWSVIPQPNRPRSFMTICGDGGLLTINLG
EDGKVASQSRSKQMFSVKDDPIFIAPALDKDKAHFVSYYGNVYSADFSGDEVKVDGPWS
LLNDEDKAKNWVPGGYNLVGLHRASGRMYVFMHPDGKEGTHKFPAAEIWVMDTKTKQ
RVARIPGRDALSMTIDQQRNLMLTLDGGNVNVYDISQPEPKLLRTIEGAAEASLQVQFHPV
GGT
>non-antioxidant_998
GMSARDVFHEVVKTALKKDGWQITDDPLTISVGGVNLSIDLAAQKLIAAERQGQKIAVEV
KSFLKQSSAISEFHTALGQFINYRGALRKVEPDRVLYLAVPLTTYKTFFQLDFPKEIIIENQV
KMLVYDVEQEVIFQWIN
>non-antioxidant_999
SLKLTALHFYKYSEPFKSQIVTPKVTLTHRDCLFIELIDDKGNAYFGECNAFQTDWYDHETI
ASVKHVIEQWFEDNRNKSFETYEAALKLVDSLENTPAARATIVMALYQMFHVLPSFSVAY
GATASGLSNKQLESLKATKPTRIKLKWTPQIMHQIRVLRELDFHFQLVIDANESLDRQDFT
QLQLLAREQVLYIEEPFKDISMLDEVADGTIPPIALDEKATSLLDIINLIELYNVKVVVLKPF
RLGGIDKVQTAIDTLKSHGAKVVIGGMYEYGLSRYFTAMLARKGDYPGDVTPAGYYFEQ
DVVAHSGILKEGRLEFRPPLVDITQLQPYEGHHHHHH
>non-antioxidant_1000
GSSAKRKQEEKHLKMLRDMTGLPHNRKCFDCDQRGPTYVNMTVGSFVCTSCSGSLRGL
NPPHRVKSISMTTFTQQEIEFLQKHGNEVCKQIWLGLFDDRSSAIPDFRDPQKVKEFLQEK
YEKKRWYVPPEQAKVVASVHA
>non-antioxidant_1001
MRVNNGLTPQELEAYGISDVHDIVYNPSYDLLYQEELDPSLTGYERGVLTNLGAVAVDTGI
FTGRSPKDKYIVRDDTTRDTFWWADKGKGKNDNKPLSPETWQHLKGLVTRQLSGKRLF
VVDAFCGANPDTRLSVRFITEVAWQAHFVKNMFIRPSDEELAGFKPDFIVMNGAKCTNPQ
WKEQGLNSENFVAFNLTERMQLIGGTWYGGEMKKGMFSMMNYLLPLKGIASMHCSAN
VGEKGDVAVFFGLSGTGKTTLSTDPKRRLIGDDEHGWDDDGVFNFEGGCYAKTIKLSKEA
EPEIYNAIRRDALLENVTVREDGTIDFDDGSKTENTRVSYPIYHIDNIVKPVSKAGHATKVI
FLTADAFGVLPPVSRLTADQTQYHFLSGFTAKLAGTERGITEPTPTFSACFGAAFLSLHPTQ
YAEVLVKRMQAAGAQAYLVNTGWNGTGKRISIKDTRAIIDAILNGSLDNAETFTLPMFNL
AIPTELPGVDTKILDPRNTYASPEQWQEKAETLAKLFIDNFDKYTDTPAGAALVAAGPKL
>non-antioxidant_1002
STRRKPENQPTRVILFNKPYDVLPQFTDEAGRKTLKEFIPVQGVYAAGRLDRDSEGLLVLT
NNGALQARLTQPGKRTGKIYYVQVEGIPTQDALEALRNGVTLNDGPTLPAGAELVDEPAW
LWPRNPPIRERKSIPTSWLKITLYEGRNRQVRRMTAHVGFPTLRLIRYAMGDYSLDNLANG
EWREVTD
>non-antioxidant_1003
PLGSATITQDTPINQIFTDTALAEKMKTVLGKTNVTDTVSQTDLDQVTTLQADRLGIKSID
GVEYLNNLTQINFSNNQLTDITPLKNLTKLVDILMNNNQIADITPLANLTNLTGLTLFNNQIT

DIDPLKNLTNLNRLELSSNTISDISALSGLTSLQQLSFGNQVTDLKPLANLTTLERLDISSNK
VSDISVLAKLTNLESLIATNNQISDITPLGILTNLDELSLNGNQLKDIGTLASLTNLTDLDLA
NNQISNLAPLSGLTKLTELKLGANQISNISPLAGLTALTNLELNENQLEDISPISNLKNLTYLT
LYFNNISDISPVSSLTKLQRLFFANNKVSDVSSLANLTNINWLSAGHNQISDLTPLANLTRIT
QLGLNDQAWTNAPVNYKANVSIPNTVKNVTGALIAPATISDGGSYTEPDITWNLPSYTNE
VSYTFSQPVTIGKGTTTFSGTVTQPLKGG
>non-antioxidant_1004
GSGPEAALENVDAKIAKLMGEGYAFEEVKRALEIAQNNVEVARSILREFAFP
>non-antioxidant_1005
MEHVPAELTRVSEGYTRFYRSPTASVILSGLVKVKWDNEQMTMPLFKWIGGEQAEELHFC
VHIAHSSGPKLNRARSLGTVNSNMDQHWAQAQRNSGATRRTIEGFHLFENDIPNFPDYIKI
KLVPKT
>non-antioxidant_1006
GMSQTTVVNGVNVDQLMATIEQIKAKPEIAQFKFRATNQWMGGTHNQATIKDFYGACAE
DDTRKPMVFDLDEPPVLLGENRGANPVEYLLVALSGCLTTSLVAHAAARGIALRGVKSRY
EGDIDLRGFLGLSEEVPVGYREIRVFFSIDADLTDGQKEELIRMAQKYSPVYNTVAKPVPVA
VLLDRG
>non-antioxidant_1007
AASAAPARPAHPLDPLSTAEIKAATNTVKSYFAGKKISFNTVTLREPARKAYIQWKEQGGP
LPPRLAYYVILEAGKPGVKEGLVDLASLSVIETRALETVQPILTVEDLCSTEEVIRNDPAVIE
QCVLSGIPANEMHKVYCDPWTIGYDERWGTGKRLQQALVYYRSDEDDSQYSHPLDFCPI
VDTEEKKVIFIDIPNRRRKVSKHKHANFYPKHMIEKVGAMRPEAPPINVTQPEGVSFKMT
GNVMEWSNFKFHIGFNYREGIVLSDVSYNDHGNVRPIFHRISLSEMIVPYGSPEFPHQRKH
ALDIGEYGAGYMTNPLSLGCDCKGVIHYLDAHFSDRAGDPITVKNAVCIHEEDDGLLFKH
SDFRDNFATSLVTRATKLVVSQIFTAANYEYCLYWVFMQDGAIRLDIRLTGILNTYILGDDE
EAGPWGTRVYPNVNAHNHQHLFSLRIDPRIDGDGNSAAACDAKSSPYPLGSPENMYGNA
FYSEKTTFKTVKDSLTNYESATGRSWDIFNPNKVNPYSGKPPSYKLVSTQCPPLLAKEGSL
VAKRAPWASHSVNVVPYKDNRLYPSGDHVPQWSGDGVRGMREWIGDGSENIDNTDILFF
HTFGITHFPAPEDFPLMPAEPITLMLRPRHFFTENPGLDIQPSYAMTTSEAKRAV
>non-antioxidant_1008
SNAIFMDYYENRKVMAEAQNIYEKSPMEEQSQDGEVRKQFKALQQINQEIVGWITMDDT
QINYPIVQAKDNDYYLFRNYKGEDMRAGSIFMDYRNDVKSQNRNTILYGHRMKDGSMF
GSLKKMLDEEFFMSHRKLYYDTLFEGYDLEVFSVYTTTTDFYYIETDFSSDTEYTSFLEKI
QEKSLYKTDTTVTAGDQIVTLSTCDYALDPEAGRLVVHAKLVKRQ
>non-antioxidant_1009
MSGKLTVITGPMYSGKTTELLSFVEIYKLGKKKVAVFKPKIDSRYHSTMIVSHSGNGVEAH
VIERPEEMRKYIEEDTRGVFIDEVQFFNPSLFEVVKDLLDRGIDVFCAGLDLTHKQNPFETT
ALLLSLADTVIKKKAVCHRCGEYNATLTLKVAGGEEEIDVGGQEKYIAVCRDCYNTLKKR
V
>non-antioxidant_1010
MITMKDIIREGNPTLRAVAEEVPVPITEEDRQLGEDMLTFLKNSQDPVKAEELQLRGDVGL
AAPQLDISKRIIAVHVPSNDPENETPSLSTVMYNPKILSHSVQDVCLGEGEGCLSVDRDVP
GYVVRHNKITVSYFDMAGEKHKVRLKNYEAIVVQHEIDHINGIMFYDHINKENPFALKEG
VLVIEL

>non-antioxidant_1011
MGSSHHHHHHSSGLVPRGSHMSGSGSGSGTALTPSYLKDDDGRSLILRGFNTASSAKSAP
DGMPQFTEADLAREYADMGTNFVRFLISWRSVEPAPGVYDQQYLDRVEDRVGWYAERG
YKVMLDMHQDVYSGAITPEGNSGNGAGAIGNGAPAWATYMDGLPVEPQPRWELYYIQP
GVMRAFDNFWNTTGKHPELVEHYAKAWRAVADRFADNDAVVAYDLMNEPFGGSLQGPA
FEAGPLAAMYQRTTDAIRQVDQDTWVCVAPQAIGVNQGLPSGLTKIDDPRAGQQRIAYCP
HLYPLPLDIGDGHEGLARTLTDVTIDAWRANTAHTARVLGDVPIILGSFGLDTTLPGARDYI
ERVYGTAREMGAGVSYWSSDPGPWGPYLPDGTQTLLVDTLNKPYPRAVAGTPTEWSSTS
DRLQLTIEPDAAITAPTEIYLPEAGFPGDVHVEGADVVGWDRQSRLLTVRTPADSGNVTVT
VTPAA
>non-antioxidant_1012
DKATIPSESPFAAAEVADGAIVVDIAKMKYETPELHVKVGDTVTWINREAMPHNVHFVAG
VLGEAALKGPMMKKEQAYSLTFTEAGTYDYHCTPHPFMRGKVVVE
>non-antioxidant_1013
MEQRITLKDYAMRFGQTKTAKDLGVYPSSINQAIHAGRKIFLTINADGSVYAEEVKPFPSN
KKTTA
>non-antioxidant_1014
SMEGMLADFVSQTSPMIPSIVVHCVNEIEQRGLTETGLYRISGCDRTVKELKEKFLRVKTV
PLLSKVDDIHAICSLLKDFLRNLKEPLLTFRLNRAFMEAAEITDEDNSIAAMYQAVGELPQ
ANRDTLAFLMIHLQRVAQSPHTKMDVANLAKVFGPTIVAHAVPNPDPVTMLQDIKRQPKV
VERLLSLPLEYWSQFMMVE
>non-antioxidant_1015
ADDAKPRVKVPSSAKAGETVTVKALISHKMESGQRKDADGKLIPRSIINRFTCELNGVNV
VDVAIDPAVSTNPYFEFDAKVDAAGEFKFTWYDDDGSVYEDVKPIAVA
>non-antioxidant_1016
MSLKTTLPISLDWSTEEVIDVVHFFQAIEQAYDQGIAREDLLGKYRRFKEIVPSKSEEKQLF
RAYEQENDVSCYQTIKKAREEMEEHIQMEGHHHHHH
>non-antioxidant_1017
GMTTTQPEAKDRISSLPVPDATQVPEGVRKLWAKAEANIGFVPNVFRAQAVNGEQFLAW
WNYFNLLLNKEGYLTNAERELVAVVVSGVNRCLYCAVSHGAALREFLGDPQKADAVAVN
WRHADLTEREQALAAYAEKLTRHPAEVTAADLEPLRAVGLDDHQIMELVQVIGMFNLTNR
VSSALGFVPNPEYYRQAR
>non-antioxidant_1018
GMPHVHVSTDNSLLDIGLIHRTLSQDTDWAKDIPLALVQRAIDHSLCFGGFVDGRQVAFAR
VISDYATFAYLGDVFVLPEHRGRGYSKALMDAVMAHPDLQGLRRFSLATSDAHGLYARYG
FTPPLFPQSLMERYVPGLYST
>non-antioxidant_1019
GMTVAAKSEIQIDNDEVRVTEWRLPPGSATGHHTHGMDYVVVPMADGEMTIVAPDGTRS
LAQLKTGRSYARKAGVQHDVRNESTAEIVFLEIELKAGS
>non-antioxidant_1020
MGSSHHHHHHSSGLVPRGSHMASKLKENAEVTGSVSLEALEEVQVGENLEVGVGIDELV
NAEAFAYDFTLNYDENAFEYVEAISDDGVFVNAKKIEDGKVRVLVSSLTGEPLPAKEVLA
KVVLRAEAKAEGSNLSVTNSSVGDGEGLVHEIAGTEKTVNIIEGTS
>non-antioxidant_1021

SMNGQLNGFHEAFIEEGTFLFTSESVGEGHPDKICDQISDAVLDAHLQQDPDAKVACETVA
KTGMILLAGEITSRAAVDYQKVVREAVKHIGYDDSSKGFDYKTCNVLVALEQQSPDIAQG
VHLDRNEEDIGAGDQGLMFGYATDETEECMPLTIVLAHKLNAKLAELRRNGTLPWLRPD
SKTQVTVQYMQDRGAVLPIRVHTIVISVQHDEEVCLDEMRDALKEKVIKAVVPAKYLDED
TIYHLQPSGRFVIGGPQGDAGLTGRKIIVDTYGGWGAHGGGAFSGKDYTKVDRSAAYAA
RWVAKSLVKGGLCRRVLVQVSYAIGVSHPLSISIFHYGTSQKSERELLEIVKKNFDLRPGVI
VRDLDLKKPIYQRTAAYGHFGRDSFPWEVPKKLKY
>non-antioxidant_1022
SNAMNPFETKSVTFAEPIEMLYACHGKVRRFCGQVAMLSDYIAENGCNQIVLQTIRQIAQY
FNVAAPLHHEDEEENFFPLLLQYAPQAQESVDELLRQHIGLHDNWAAVSAEFAKLEADNA
YVPDEEAFKRFVAGYDVHLAIEEPLFDMGNTFIPKEKLTEIGEIMAARRRK
>non-antioxidant_1023
SNAQLGGDMKTIAIADRTGEYEQLFKENDEFRFVHAEKTAEEYRKMGADKSGIDAVLEIR
QDLLEDPNAVAIYGYKQLPASVSNHISRILSDYLSDKKIASYNIPDIKQILADSKIELSVHTY
KWSEDGTNERTSGELASGIS
>non-antioxidant_1024
MQDILDFYEEVEKTINPPNYFEWNTYRVFKKLGSYKNLVPNFKLDDSGHPIGNAIPGVEDI
LVEYEHFSILIECSLTIGEKQLDYEGDSVVRHLQEYKKKGIEAYTLFLGKSIDLSFARHIGFN
KESEPVIPLTVDQFKKLVTQLKGDGEHFNPNKLKEILIKLLRSDLGYDQAEEWLTFIEYNL
KHHHHHH
>non-antioxidant_1025
MAIQRRIRRVKTVQMTTNSPIHRSGSVLEPGNWQEYDPFLLLMEDIFERGTFDVHPHRGIE
TVTYVISGELEHFDSKAGHSTLGPGDVQWMTAGRGVVHKEDPASGSTVHSLQLWVNLPS
AYKMTEPRYQNLRSKDMPVRKEEGATIRVFSGSSKGVKAPTKNIVPVTMVEMIVEPGTTV
VQDLPGHYNGFLYILEGSGVFGADNIEGKAGQALFFSRHNRGEETELNVTAREKLRLLLY
AGEPVNEPVVAYGPFVMNTPEQIREAIRDYQEGRFGR
>non-antioxidant_1026
GMKKIRFAAIGLAHNHIYDMCQQLIDAGAELAGVFESDSDNRAKFTSLFPSVPFAASAEQL
ITDASIDLIACAVIPCDRAELALRTLDAGKDFFTAKPPLTTLEQLDAVQRRVAETGRKFAVYF
NERINVDSALFAGELVQRGEIGRVIQTMGVGPHRERGARPDWFYQKRQYGGILCDIGIHQI
EQFLYFTGNTNARVVTSQTANYHHPHHPEFEDFGDAMLLGDNGATGYFRCDWFTPDGLS
VWGDGRLTILGTEGYIEIRKYVDLTRGESNVVYLVNGKGEQRFTPAGSVERAFFPDFLRDC
RERTENAMSQSHIFKATELSILAQQAANKIA
>non-antioxidant_1027
MGSSHHHHHHSSGEDRYKEKLLQKAKAEGVESIEELKKRLADQIEEKKKELNKIDPLREL
EQHLNAGSRIHTNKEHKTTKMSNKSNEKSGNVLPKDKPYKTLDDYLKLDKIKDLSKQEV
EFLWRAKWSNRDDSLVAVVPYVKTFQGMYKYAVKNPLFVLPLPRENAADGNKADKDSV
PVELQYVQWQFAGPNTVHCLITSLAEYKLHQDFAKPHTTIQFHLDLANDKDMVLMNGQ
VESDSNVSLQDAQLLLLNVQRFYGAMGSETSIAKERIQLLEDFNKGSQNFDINKLIQLAQS
MEN
>non-antioxidant_1028
KGRVCVTGGTGFLGSWIIKSLLENGYSVNTTIRADPERKRDVSFLTNLPGASEKLHFFNAD
LSNPDSFAAAIEGCVGIFHTASPIDFAVSEPEEIVTKRTVDGALGILKACVNSKTVKRFIYTS
SGSAVSFNGKDKDVLDESDWSDVDLLRSVKPFGWNYAVSKTLAEKAVLEFGEQNGIDVV

TLILPFIVGRFVCPKLPDSIEKALVLVLGKKEQIGVTRFHMVHVDDVARAHIYLLENSVPGG
RYNCSPFIVPIEEMSQLLSAKYPEYQILTVDELKEIKGARLPDLNTKKLVDAGFDFKYTIED
MFDDAIQCCKEKGYL
>non-antioxidant_1029
GMNSNFSYPALGVDGISSQISPIRDVWSTNLQQEMNLIMSLIERYPVVSMDTEFPGVVARP
LGVFKSSDDYHYQTLRANVDSLKIIQIGLALSDEEGNAPVEACTWQFNFTFNLQDDMYAP
ESIELLTKSGIDFKKHQEVGIEPADFAELLIGSGLVLQEEVTWITFHSGYDFAYLLKAMTQIP
LPAEYEEFYKILCIYFPKNYDIKYIMKSVLNNSKGLQDIADDLQIHRIGPQHQAGSDALLTA
RIFFEIRSRYFDGSIDSRMLNQLYGLGSTGSVLWHNNSSTPQIQFRDLPGAHPSPTPSNAGIP
TTLTNTSSAPNFANSTFRFPPRVV
>non-antioxidant_1030
MNKGQRHIKIREIITSNEIETQDELVDMLKQDGYKVTQATVSRDIKELHLVKVPTNNGSYK
YSL
>non-antioxidant_1031
ASMTTPCITILSGHFPKETIYARKTKELVEEYCSIHGYNFYYEESEPLETEEHALHFRRSWII
QQAAEKFPSTEWFLWLDSDVYVNPKNKNKPITSFIDLSDPNILYHTFHEAPWGSYPINTGV
KFVHKDALEIEKIVWSLRNEAPWNTFPYEQKTVYEYVFPRIPGRYIVHDPYTLNCIVKAYP
EHVKDALFVHMCGTSRAERDEHMEMVAT
>non-antioxidant_1032
GMENFIGSHMIYTYENGWEYEIYIKNDHTIDYRIHSGMVAGRWVRDQEVNIVKLTEGVYK
VSWTEPTGTDVSLNFMPNEKRMHGIIFFPKWVHEHPEITVCYQNDHIDLMKESREKYETY
PKYVVPEFAEITFLKNEGVDNEEVISYAPYEGMTDDIRAGRL
>non-antioxidant_1033
GMTFRDTSAIASWHAHVYFDASSRDAAWTLREQIEAHWSGKLQLGRFHERPVGPHPMW
SYQLAFTQEQFADLVGWLTLNHGALDIFLHPNTGDALRDHRDAAVWIGHSHELVLSALN
>non-antioxidant_1034
ALPDQIDVKVKNLTPEDTIYDRTRQVFYQSNLYKGRIEVYNPKTQSHFNVVIDGASSNGD
GEQQMSGLSLLTHDNSKRLFAVMKNAKSFNFADQSSHGASSFHSFNLPLSENSKPVWSVN
FEKVQDEFEKKAGKRPFGVVQSAQDRDGNSYVAFALGMPAIARVSADGKTVSTFAWESG
NGGQRPGYSGITFDPHSNKLIAFGGPRALTAFDVSKPYAWPEPVKINGDFGTLSGTEKIVTV
PVGNESVLVGARAPYAISFRSWDNWKSANIKKTKRSELQNSGFTAVADYYQGSEQGLYAV
SAFFDNGAHGGRSDYPLYKLDNSIQNFHHHHHH
>non-antioxidant_1035
GHMENKVINFKKIIDSRGSLVAIEENKNIPFSIKRVYYIFDTKGEEPRGFHAHKKLEQVLVC
LNGSCRVILDDGNIIQEITLDSPAVGLYVGPAVWHEMHDFSSDCVMMVLASDYYDETDYIR
QYDNFKKYIAKINLEKEG
>non-antioxidant_1036
LEEVIEQLREANEPVPVPLELPDEDQLVEIEEQLFINIPFVFKEFLLTVSDVVYGSLEPVTVT
DPQSHTYLPEVCATAWDLGVPRELIPICQDGEDYYCVEEDGTVLLWSAEEELVTEESWES
VWHWARDVWLES
>non-antioxidant_1037
MCDEDETTALVCDNGSGLVKAGFAGDDAPRAVFPSIVGRPRHQGVMVGMGQKDSYVGD
EAQSKRGILTLKYPIEHGIITNWDDMEKIWHHTFYNELRVAPEEHPTLLTEAPLNPKANRE
KMTQIMFETFNVPAMYVAIQAVLSLYASGRTTGIVLDSGDGVTHNVPIYEGYALPHAIMRL

DLAGRDLTDYLMKILTERGYSFVTTAEREIVRDIKEKLCYVALDFENEMATAASSSSLEKS
YELPDGQVITIGNERFRCPETLFQPSFIGMESAGIHETTYNSIMKCDIDIRKDLYANNVMSG
GTTMYPGIADRMQKEITALAPSTMKIKIIAPPERKYSVWIGGSILASLSTFQQMWITKQEY
DEAGPSIVHRKCF
>non-antioxidant_1038
AGWNAYIDNLMADGTCQDAAIVGYKDSPSVWAAVPGKTFVNITPAEVGVLVGKDRSSFY
VNGLTLGGQKCSVIRDSLLQDGEFSMDLRTKSTGGAPTFNVTVTKTDKTLVLLMGKEGV
HGGLINKKCYEMASHLRRSQY
>non-antioxidant_1039
GPPPAPPLPAAQGPGGGGAGAPGLAAAIAGAKLRKVSKQEEAS
>non-antioxidant_1040
MGSDKIHHHHHHENLYFQGMQIRLAFPNEIDQIMLLIEEARAEIAKTGSDQWQKEDGYPN
RNDIIDDILNGYAWVGIEDGMLATYAAVIDGHEEVYDAIYEGKWLHDNHRYLTFHRIAISN
QFRGRGLAQTFLQGLIEGHKGPDFRCDTHEKNVTMQHILNKLGYQYCGKVPLDGVRLAY
QKIKEKGETSIYREIDERNPM
>non-antioxidant_1041
PRILGRNIGSHHVRVEHFMNHSITTLAKDTPLEEVVKVVTSTDVTEYPLVESTESQILVGIV
QRAQLVQALQAEPPSRAPGHQQCLQDILARGCPTEPVTLTLFSETTLHQAQNLFKLLNLQS
LFVTSRGRAVGCVSWVEMKKAISNLTNPPAPKEFLEVLFQG
>non-antioxidant_1042
QGHMRVEVFPVEGLPLIKEGDDLAELISSRVRFEDGDVLVVCSTVISKAEGRIRRLEEFNPS
ERAKEIAARIGKPAEFVQAVLEESEEVLLDFPFLLVKAKFGNVCVNAGIDASNVEEGSLLL
PPLDPDGSAEKLRRRILELTGKRVGVIITDTNGRCFRRGVVGFAIGISGVKAMKDWIGRKD
LYGRELEVTVECVADEIAAFANLLMGEGGDGIPAVVVRGLNVAGEGSMEEIYRSEEEDVIR
RCLKRCLGS
>non-antioxidant_1043
GAHMAGGRSWCLRRVGMSAGWLLLEDGCEVTVGRGFGVTYQLVSKICPLMISRNHCVL
KQNPEGQWTIMDNKSLNGVWLNRARLEPLRVYSIHQGDYIQLGVPLENKENAEYEYEVT
EEDWETIYPCLSPKNDQMIEK
>non-antioxidant_1044
GTEDLYFQSHMTIAVTGSIATDHLMRFPGRFSEQLLPEHLHKVSLSFLVDDLVMHRGGVAG
NMAFAIGVLGGEVALVGAAGADFADYRDWLKARGVNCDHVLISETAHTARFTCTTDVD
MAQIASFYPGAMSEARNIKLADVVSAIGKPELVIIGANDPEAMFLHTEECRKLGLAFAADP
SQQLARLSGEEIRRLVNGAAYLFTNDYEWDLLLSKTGWSEADVMAQIDLRVTTLGPKGV
DLVEPDGTTIHVGVVPETSQTDPTGVGDAFRAGFLTGRSAGLGLERSAQLGSLVAVLVLES
TGTQEWQWDYEAAASRLAGAYGEHAAAEIVAVLA
>non-antioxidant_1045
MSLDCRERIEKDLELLEKNLMEMKSIKLSDDEEAVVERALNYRDDSVYYLEKGDHITSFG
CITYAHGLLDSLRMLHRIIEGHHHHHH
>non-antioxidant_1046
MDEIDLRILKILQYNAKYSLDEIAREIRIPKATLSYRIKKLEKDGVIKGYYAYINPASLNLDY
IVITSVKAKYGKNYHVELGNKLAQIPGVWGVYFVLGDNDFIVMARYKTREEFMEKFLER
VMSIPEVERTSTQVVVKIIKESPNIVIF
>non-antioxidant_1047

GHPTLKTPESVTGTWKGDVKIQCIYDPLRGYRQVLVKWLVRHGSDSVTIFLRDSTGDHIQ
QAKYRGRLKVSHKVPGDVSLQINTLQMDDRNHYTCEVTWQTPDGNQVIRDKIIELRVRK
>non-antioxidant_1048
CKGADGAHGVNGCPGTAGAAGSVGGPGCDGGHGGNGGNGNPGCAGGVGGAGGASGG
TGVGGRGGKGGSGTPKGADGAPGAP
>non-antioxidant_1049
SLTGEESDTLRKIVLEECLPNQQQNQNPSPCAEVKPNAGYVVLKDLNGPLQYLLMPTYRI
NGTESPLLTDPSTPNFFWLAWQARDFMSKKYGQPVPDRAVSLAINSRTGRTQNHFHIHISCI
RPDVREQLDNNLANISSRWLPLPGGLRGHEYLARRVTESELVQRSPFMMLAEEVPEAREH
MGSYGLAMVRQSDNSFVLLATQRNLLTLNRASAEEIQDHQCEILR
>non-antioxidant_1050
GVQVETISPGDGRTFPKRGQTCVVHYTGMLEDGKKFDSSRDRNKPFKFMLGKQEVIRGW
EEGVAQMSVGQRAKLTISPDYAYGATGHPGIIPPHATLVFDVELLKLE
>non-antioxidant_1051
GMNLTELMERIPHLREILNIVREAFKDYDDPAHDISHTFRVMENASEIASREKCDLQKAIIA
ALLHDIKRPHEALTGVDHAESGAEYASGLLPTMGFDISFVAEVSKAIRSHRYSGGLTPTSLT
GKILQDADRLDAIGAVAIARVFSYSGKTGTPLHSLQFSPRSSYSGNSRSSINHFHEKILKIRP
ETFWTETARKMAEDRYSFVVEFVQRFLAEWGQI
>non-antioxidant_1052
GSTKVKYVDKIHIGNYEIDAWYFSPFPEDYGKQPKLWLCEYCLKYMKYEKSYRFHLGQC
QWRQPPGKEIYRKSNISVHEVDGKDHKIYCQNLCLLAKLFLDHRTLYFDVEPFVFYILTEV
DRQGAHIVGYFSKEKESPDGNNVACILTLPPYQRRGYGKFLIAFSYELSKLESTVGSPEKPL
SDLGKLSYRSYWSWVLLENLRDFRGTLSIKDLSQMTSITQNDIISTLQSLNMVKYWKGQH
VICVTPKLVEEHLKSAQYKKPPITVDSVCLKWAPPK
>non-antioxidant_1053
GMRGLIVDYAGVLDGTDEDQRRWRNLLAAAKKNGVGTVILSNDPGGLGAAPIRELETNG
VVDKVLLSGELGVEKPEEAAFQAAADAIDLPMRDCVLVDDSILNVRGAVEAGLVGVYYQ
QFDRAVVEIVGLFGLEGEF
>non-antioxidant_1054
GMIYSKVENFINENKQNAIFTEGASHENIGRIEENLQCDLPNSYKWFLEKYGAGGLFGVLV
LGYNFDHASVVNRTNEYKEHYGLTDGLVVIEDVDYFAYCLDTNKMKDGECPVVEWDRVI
GYQDTVADSFIEFFYNKIQEAKDDWDEDEDWDD
>non-antioxidant_1055
GMQGIAFQDAYPDDLSHCYGCGRNNEQGHQLKSYWRGEQTIAHFMPKPFHTAIPGFVYG
GLIASLIDCHGTGSASAAAQRALEQAGEQLDEPPRFVTAALNIDYLAPTPMGVELELVGEI
KEVKPRKVVVEIALSADGKLCARGHMVAVKMPETMAATSA
>non-antioxidant_1056
TIKLIVGLANPGAEYAATRHNAGAWFVDLLAERLRAPLREEAKFFGYTSRVTLGGEDVRL
LVPTTFMNLSGKAVAAMASFFRINPDEILVAHDELDLPPGVAKFKLGGGHGGHNGLKDIIS
KLGNNPNFHRLRIGIGHPGDKNKVVGFVLGKPPVSEQKLIDEAIDEAARCTEMWFTDGLT
KATNRLHAFKAQ
>non-antioxidant_1057
APPSSFSKAKKEAVKIYLDYPTSFYCGCDITWKNKKKGIPELESCGYQVRKQEKRASRIEW
EHVVPAWQFGHQRQCWQKGGRKNCTRNDKQFKSMEADLHNLVPAIGEVNGDRSNFRFS

QWNGSKGAFYGQCAFKVDFKGRVAEPPAQSRGAIARTYLYMNNEYKFNLSKAQRQLME
AWNKQYPVSTWECTRDERIAKIQGNHNQFVYKAC
>non-antioxidant_1058
HMIHEDFCSVCRKSGQLLMCDTCSRVYHLDCLDPPLKTIPKGMWICPRCQDQMLKKEEAI
>non-antioxidant_1059
TELNLSHILIPLPENPTSDQVNEAESQARAIVDQARNGADFGKLAIAHSADQQALNGGQM
GWGRIQELPGIFAQALSTAKKGDIVGPIRSGVGFHILKVNDLR
>non-antioxidant_1060
MSFAGLKDADVAAALAACSAADSFKHKEFFAKVGLASKSLDDVKKAFYVIDQDKSGFIE
EDELKLFLQNFSPSARALTDAETKAFLADGDKDGDGMIGVDEFAAMIKA
>non-antioxidant_1061
MSNKLFPPQIKVAATYMRGGTSKGVFFRLQDLPEAAQVPGPARDALLLRVIGSPDPYAKQI
DGMGGATSSTSKTVILSHSSKANHDVDYLFGQVSIDKPFVDWSGNCGNLTAAVGAFAISN
GLIDAARIPRNGVCTVRIWQANIGKTIIAHVPITDGAVQETGDFELDGVTFPAAEVQIEFMN
PAADDDGEGGCMFPTGNLVDVLEVPGIGRFNATMINAGIPTIFINAEDLGYTGTELQDDIN
SDNAALAKFETIRAHGALRMGLIKHIDEAASRQHTPKIAFVAPPKSYASSSGKTVAAEDVD
LLVRALSMGKLHHAMMGTAAVAIGTAAAIPGTLVNLAAGGGEKEAVRFGHPSGTLRVGA
QAVQENGEWTVIKAIMSRSARVLMEGFVRVPKP
>non-antioxidant_1062
GSGYREVEYWDQRYQGAADSAPYDWFGDFSSFRALLEPELRPEDRILVLGCGNSALSYEL
FLGGFPNVTSVDYSSVVVAAMQACYAHVPQLRWETMDVRKLDFPSASFDVVLEKGTLD
ALLAGERDPWTVSSEGVHTVDQVLSEVSRVLVPGGRFISMTSAAPHFRTRHYAQAYYGW
SLRHATYGSGFHFHLYLMHKGGKLSVAQLALGAQILSP
>non-antioxidant_1063
MKHMPRKMYSCAFETTTKVEDCRVWAYGYMNIEDHSEYKIGNSLDEFMAWVLKVQADL
YFHNLKFAGAFIINWLERNGFKWSADGLPNTYNTIISRMGQWYMIDICLGYKGKRKIHTV
IYDSLKKLPFPVKKIAKDFKLTVLKGDIDYHKERPVGYKITPEEYAYIKNDIQIIAEALLIQF
KQGLDRMTAGSDSLKGFKDIITTKKFKKVFPTLSLGLDKEVRYAYRGGFTWLNDRFKEKE
IGEGMVFDVNSLYPAQMYSRLLPYGEPIVFEGKYVWDEDYPLHIQHIRCEFELKEGYIPTIQ
IKRSRFYKGNEYLKSSGGEIADLWLSNVDLELMKEHYDLYNVEYISGLKFKATTGLFKDFI
DKWTYIKTTSEGAIKQLAKLMLNSLYGKFASNPDVTGKVPYLKENGALGFRLGEEETKDP
VYTPMGVFITAWARYTTITAAQACYDRIIYCDTDSIHLTGTEIPDVIKDIVDPKKLGYWAHE
STFKRAKYLRQKTYIQDIYMKEVDGKLVEGSPDDYTDIKFSVKCAGMTDKIKKEVTFENF
KVGFSRKMKPKPVQVPGGVVLVDDTFTIK
>non-antioxidant_1064
GMGKRDDLIAQYADDLRNKCGMEPDMALLEKVTKGCGPAIYNRDASTVAGSDTAELETI
KKNFLMKKLGLADSESLMGGIQSVIETYGRSERNKYRAVVYYMLTKHFGKESVYG
>non-antioxidant_1065
GMMQKPITEIIIVGGGTAGWITAGLLAAEHNVDKGVLAHSPKLNITLIESPDVATIGVGEGT
WPSMRSTLSKIGIDENDFIRQCDASFKQGSRFINWCKDPQSNVADSYLHPFSLPHGHQELD
LCPYWLPHAEQVSFAEAVCSQQVLTQLGLAPKSIVTAQYHFQNNYGYHLNAAKFSQLLTE
HCTQKLGVTHIRDHVSQIINNQHGDIEKLITKQNGEISGQLFIDCTGAKSLLLGEHLQVPFL
SQKSVLFNDRALAIQVPYSDANSPIASCTHSTAQPNGWIWDIGLPTRKGVGYVYSSSHTN
DIDAQKTLFNYLGVDGAAADKLEPRQLAINPGYRAKCWQNNCIAIGMAAGFIEPLEASAL

ALIEWTASTLAQQLPPNRMVMDTISARVNERYQQHWQQIIDFLKLHYVISQRQEDRYWR
DHRESNSIPDSLQAMLELWRYQTPSQQDISYKEALFPAASFQYVLYGMSFNTQLPTHVKPS
MQQLAQRLFNDNQQRTQALSKNLPTNRELLDKVAQYGFPKL
>non-antioxidant_1066
SELRPSGDSGSSDVDAEISDGFSPLDTSHRDVADEGSLLRRAEMYQDYMKQVPIPTNRGSL
IPFTSWVGLSISMKQLYGQPLHYLTNVLLQRWDQSRFGTDSEEQRLDSIIHPTKAEATIWLV
EEIHRLTPSHLHMALLWRSDPMYHSFIDPIFPEK
>non-antioxidant_1067
SNQLQQLQNPGESPPVHPFVAPLSYLLGTWRGQGEGEYPTIPSFRYGEEIRFSHSGKPVIAY
TQKTWKLESGAPMHAESGYFRPRPDGSIEVVIAQSTGLVEVQKGTYNVDEQSIKLKSDLV
GNASKVKEISREFELVDGKLSYVVRMSTTTNPLQPHLKAILDKL
>non-antioxidant_1068
MSLSLKIALISQNENLLNLFPKLALEKNFIPITKTASLTRASKIAFGLQDEVDAIISRGATSDY
IKKSVSIPSISIKVTRFDTMRAVYNAKRFGNELALIAYKHSIVDKHEIEAMLGVKIKEFLFSS
EDEITTLISKVKTENIKIVVSGKTVTDEAIKQGLYGETINSGEESLRRAIEEALNLIEVRNEG
HHHHHH
>non-antioxidant_1069
MQHNLIAFLSDVGSADEAHALCKGVMYGVAPAATIVDITHDVAPFDVREGALFLADVPHS
FPAHTVICAYVYPETGTATHTIAVRNEKGQLLVGPNNGLLSFALDASPAVECHEVLSPDVM
NQPVTPTWYGKDIVAACAAHLAAGTDLAAVGPRIDPKQIVRLPYASASEVEGGIRGEVVRI
DRAFGNVWTNIPTHLIGSMLQDGERLEVKIEALSDTVLELPFCKTFGEVDEGQPLLYLNSR
GRLALGLNQSNFIEKWPVVPGDSITVSPRVPDSNLGPVLG
>non-antioxidant_1070
MFENITAAPADPILGLADLFRADERPGKINLGIGVYKDETGKTPVLTSVKKAEQYLLENET
TKNYLGIDGIPEFGRCTQELLFGKGSALINDKRARTAQTPGGTGALRVAADFLAKNTSVKR
VWVSNPSWPNHKSVFNSAGLEVREYAYYDAENHTLDFDALINSLNEAQAGDVVLFHGCC
HNPTGIDPTLEQWQTLAQLSVEKGWLPLFDFAYQGFARGLEEDAEGLRAFAAMHKELIVA
SSYSKNFGLYNERVGACTLVAADSETVDRAFSQMKAAIRANYSNPPAHGASVVATILSND
ALRAIWEQELTDMRQRIQRMRQLFVNTLQEKGANRDFSFIIKQNGMFSFSGLTKEQVLRL
REEFGVYAVASGRVNVAGMTPDNMAPLCEAIVAVL
>non-antioxidant_1071
GMANKVEHRLSEQQMKALTDLPLVFLITHDQSKSWPITHAISWVYAKDETTIRFAIEADSL
LVKTLADHPVFTLIFFADQSTYSLTCTDVAAWETTARLPLKVALYEGQIKEVRDILFYGAAV
SDRPRVYKTYDEAAAMQLDQQIQDILKG
>non-antioxidant_1072
EPTCNTPSNRACWSDGFDINTDYEVSTPDTGVTQSYVFNLTEVDNWMGPDGVVKEKVM
LINGNIMGPNIVANWGDTVEVTVINNLVTNGTSIHWHGIHQKDTNLHDGANGVTECPIPP
KGGQRTYRWRARQYGTSWYHSHFSAQYGNGVVGTIQINGPASLPYDIDLGVFPITDYYY
RAADDLVHFTQNNAPPFSDNVLINGTAVNPNTGEGQYANVTLTPGKRHRLRILNTSTENHF
QVSLVNHTMTVIAADMVPVNAMTVDSLFLAVGQRYDVVIDASRAPDNYWFNVTFGGQA
ACGGSLNPHPAAIFHYAGAPGGLPTDEGTPPVDHQCLDTLDVRPVVPRSVPVNSFVKRPD
NTLPVALDLTGTPLFVWKVNGSDINVDWGKPIIDYILTGNTSYPVSDNIVQVDAVDQWTY
WLIENDPEGPFSLPHPMHLHGHDFLVLGRSPDVPAASQQRFVFDPAVDLARLNGDNPPRR
DTTMLPAGGWLLLAFRTDNPGAWLFHCHIAWHVSGGLSVDFLERPADLRQRISQEDEDDF

NRVCDEWRAYWPTNPYPKIDSGL
>non-antioxidant_1073
MEAQPQVISATGVVKGIDLESKKITIHHDPIAAVNWPEMTMRFTITPQTKMSEIKTGDKVA
FNFVQQGNLSLLQDIKVSQ
>non-antioxidant_1074
GMENLGDRLEVVAELSLAPGNITLTPDGRLFLSLHQFYQPEMQVAELTQDGLIPFPPQSGN
AIITFDTVLGIKSDGNGIVWMLDNGNQSKSVPKLVAWDTLNNQLSRVIYLPPPITLSNSFVN
DLAVDLIHNFVYISDPAPDDKAALIRVDLQTGLAARVLQGYPGIAPEDIDLVIDGVPVQIGQ
PDGTVIRPHLGVNGIVLDAENEWLYLSPMHSTSMYRIKSADLSNLQLTDAELGSKIERYSE
KPICDGISIDKDHNIYVGDLAHSAIGVITSADRAYKLLVTDEKLSWTDSFNFGSDGYLYFDC
NQLHHSAPLNAGENISAPPYYIFRLKPLAAGIVGR
>non-antioxidant_1075
SKLQATKTLAADVIMRSPVSWKQELTLDAGRSKGASENMLAIANGGLIGSVSKVEENSTI
VNLLTNTENADKISVKIQHGSTTIYGIIIGYDKENDVLKISQLNSNSDISAGDKVTTGGLGN
FNVADIPVGEVVATTHSTDYLTREVTVKLSADTHNVDVIELVGNSKLVPR
>non-antioxidant_1076
MGAPTLPPAWQPFLKDHRISTFKNWPFLEGCACTPERMAEAGFIHCPTENEPDLAQCFFCF
KELEGWEPDDDPIEEHKKHSSGCAFLSVKKQFEELTLGEFLKLDRERAKNKIAKETNNKK
KEFEETAKKVRRAIEQLAAMD
>non-antioxidant_1077
SLRRRKLASFLKDFDREVEIRIKQIESDRQNLLKEVDNLYNIEILRLPKALREMNWLDYFAL
>non-antioxidant_1078
MGTTAPGPIHLLELCDQKLMEFLCNMDNKDLVWLEEIQEEAERMFTR
>non-antioxidant_1079
MGSSHHHHHHSSGLVPRGSPYTLSKRINGKWSGQSAGGCGNFQETHKNNPIYQFHIEKTG
PLLIELRGPRQYSVGFEVVTVSTLGDPGPHGFLRKSSGDYRCGFCYLELENIPSGIFNIIPST
FLPKQEGPFFLDFNSIIPIKITQLQ
>non-antioxidant_1080
MFKKLLHSLVAGLTFVAAVAAVPAHAQEADAQATVKTAVDDVLATIKGDPDLRGGNLQKV
FQLVDQKIVPRADFKRTTQIAMGRFWSQATPEQQQQIQDGFKSLLIRTYAGALANVRNQT
VAYKPFRAAADDTDVVVRSTVNNNGEPVALDYRVEKSPNGWKVYDINISGLWLSETYKN
QFADVISKRGGVGGLVQFLDERNAQLAKGPAK
>non-antioxidant_1081
MNSLLFVYGTLRKHEKNHHLLAQSACINEQARTKGSLFAAKEGPTVVFNDEDEGYIYGE
VYEADELCIHKLDQFFQGYHKQTVFVETDVGIKIALIYFMNKDGCAGFTKISSGDWKEHQ
MISKSKNPIYYFAYGSCMDNARFQKAGVDHYFQDPVGRAVLKGYTTRFTLKREDGSRAD
MLEDGGTTEGVLYRIPYSALSYLYKREGVESLTYRPAFVDVEAGGRHYKDCLTFLVLQKE
AEIAPPQHYQIEIERGAELYLSPEFTEKLKRHMNSLPKGLEHHHHHH
>non-antioxidant_1082
SNAMQSDHKEKIAILVDVQNVYYTCREAYRSNFDYNQFWYVATQEKEVVSAKAYAIASN
DPKQRQFHHILRGVGFEVMLKPYIQRRDGSAKGDWDVGITLDAIEIAPDVDRVILVSGDG
DFSLLVERIQQRYNKKVTVYGVPRLTSQTLIDCADNFVAIDDDFLL
>non-antioxidant_1083
GMSRGHCILAHGFESGPDALKVTALAEVAERLGWTHERPDFTDLDARRDLGQLGDVRGR

LQRLLEIARAATEKGPVVLAGSSLGSYIAAQVSLQVPTRALFLMVPPTKMGPLPALDAAA
VPISIVHAWHDELIPAADVIAWAQARSARLLLVDDGHRLGAHVQAASRAFAELLQSL
>non-antioxidant_1084
GSDNLSRHDMLAWINESLQLNLTKIEQLCSGAAYCQFMDMLFPGSIALKKVKFQAKLEHE
YIQNFKILQAGFKRMGVDKIIPVDKLVKGKFQDNFEFVQWFKKFFDANYDGKDYDPVAA
RQGQ
>non-antioxidant_1085
GIPRNSLEKFNVDLMKKAGKELGLSLSPNEIGCTIADLIQGQYPEIDSKLQRGDIITKFNGD
ALEGLPFQVSYALFKGANGKVSMEVTRPKPAAAS
>non-antioxidant_1086
MRGSHHHHHHGMASMTGGQQMGRDLYDDDDKDRWGSGDMTLEKHAFKMQLNPGME
AEYRKRHDEIWPELVDLLHQSGASDYSIHLDRETNTLFGVLTRPKDHTMASLPDHPVMKK
WWAHMADIMATNPDNSPVQSDLVTLFHMP
>non-antioxidant_1087
MGSSHHHHHHSSGLVPRGSHMASAASYPPIKNTKVGLALSSHPLASEIGQKVLEEGGNAI
DAAVAIGFALAVVHPAAGNIGGGGFAVIHLANGENVALDFREKAPLKATKNMFLDKQGNV
VPKLSEDGYLAAGVPGTVAGMEAMLKKYGTKKLSQLIDPAIKLAENGYAISQRQAETLKE
ARERFLKYSSSKKYFFKKGHLDYQEGDLFVQKDLAKTLNQIKTLGAKGFYQGQVAELIEK
DMKKNGGIITKEDLASYNVKWRKPVVGSYRGYKIISMSPPSSGGTHLIQILNVMENADLS
ALGYGASKNIHIAAEAMRQAYADRSVYMGDADFVSVPVDKLINKAYAKKIFDTIQPDTVT
PSSQIKPGMGQLHEGSN
>non-antioxidant_1088
GMKCNDKLAPFEVFDHVVNKKLSFRHVTMDDVDMLHSWMHEEHVIPYWKLNIPLVDY
KKHLQTFLNDDHQTLMVGAINGVPMSYWESYWVKEDIIANYYPFEEHDQGIHLLIGPQE
YLGQGLIYPLLLAIMQQKFQEPDTNTIVAEPDRRNKKMIHVFKKCGFQPVKEVELPDKIGL
LMKCEQNVFEKRWSDWKMNKF
>non-antioxidant_1089
MGSSHHHHHHSSGRENLYFQGHMNGLNKGIRKVELAVKWDPSPPGDPATDLDIVAATFLA
GDAYGKPAYVVHFDSRSPDGTIYLNRDSKDGKGFGWDEVMTLELNRLDSRYARVVVGV
VIQQRDAHRTFVGVLNPGLRMREGYTVLAEDDFGGVLGSTAATVGEFVRDDSGEWTFHP
GIHGYDSDPATFARVMGGRQDS
>non-antioxidant_1090
SERRLGVRAWVKENRGSFQPPVCNKLMHQEQLKVMFIGGPNTRKDYHIEEGEEVFYQLE
GDMVLRVLEQGKHRDVVIRQGEIFLLPARVPHSPQRFANTVGLVVERRRLETELDGLRYY
VGDTMDVLFEKWFYCKDLGTQLAPIIQEFFSSEQYRTGKPIPDQLLKEPPFPLSTRSIMEPM
SLDAWLDSHHRELQAGTPLSLFGDTYETQVIAYGQGSSEGLRQNVDVWLWQLEGSSVVT
MGGRRLSLAPDDSLLVLAGTSYAWERTQGSVALSVTQDPACKKPLG
>non-antioxidant_1091
GMHTQEALFVRLALDAWNTQSSRTDKLIQSLSNEALAVETAPGRNSGTYLLGHLTAVHDA
MLPLLELGDTLYPQLAPVFIQNPDKSGLEKPEINDLRLYWSLVQERLANQFNQLQPADWF
NKHAAISREDFLKEPHRNKLSVLINRTNHMAYHLGQLAYLKK
>non-antioxidant_1092
GSDEKRLHFGNGHLKLPGLRTFVDPHTFEDPTQTVHEFAKELDATNISIDKVVGAGEFGEV
CSGRLKLPSKKEISVAIKTLKVGYTEKQRRDFLGEASIMGQFDHPNIIRLEGVVTKSKPVMI

VTEYMENGSLDSFLRKHDAQFTVIQLVGMLRGIASGMKYLSDMGYVHRDLAARNILINS
NLVCKVSDFGLGRVLEDDPEAAYTTRGGKIPIRWTSPEAIAYRKFTSASDVWSYGIVLWEV
MSYGERPYWEMSNQDVIKAVDEGYRLPPPMDCPAALYQLMLDCWQKDRNNRPKFEQIV
SILDKLIRNPGSLKIITSAAARPSNLLLDQSNVDITTFRTTGDWLNGVWTAHCKEIFTGVEY
SSCDTIAKIS
>non-antioxidant_1093
GMDDLSEFVDQVPLLDHHCHFLIDGKVPNRDDRLAQVSTEADKDYPLADTKNRLAYHGF
LALAKEFALDANNPLAAMNDPGYATYNHRIFGHFHFKELLIDTGFVPDDPILDLDQTAELV
GIPVKAIYRLETHAEDFMLEHDNFAAWWQAFSNDVKQAKAHGFVGFKSIAAYRVGLHLE
PVNVIEAAAGFDTWKHSGEKRLTSKPLIDYMLYHVAPFIIAQDMPLQFHVGYGDADTDM
YLGNPLLMRDYLKAFTKKGLKVVLLHCYPYHREAGYLASVFPNLYFDISLLDNLGPSGAS
RVFNEAVELAPYTRILFASDASTYPEMYGLAARQFKQALVAHFNQLPFVDLAQKKAWINA
ICWQTSAKLYHQERELRV
>non-antioxidant_1094
MGSDKIHHHHHHENLYFQGMRDHVEIGIGREARRTYSLDDISVVSSRRTRSSKDVDTTWH
IDAYKFDLPFMNHPSDALASPEFVIEMGKQGGLGVINAEGLWGRHADLDEAIAKVIAAYE
EGDQAAATRTLQELHAAPLDTELLSERIAQVRDSGEIVAVRVSPQNVREIAPIVIKAGADLL
VIQGTLISAEHVNTGGEALNLKEFIGSLDVPVIAGGVNDYTTALHMMRTGAVGIIVGGGEN
TNSLALGMEVSMATAIADVAAARRDYLDETGGRYVHIIADGSIENSGDVVKAIACGADAV
VLGSPLARAEEAAGKGYFWPAVAAHPRFPRGVVTESVDLDEAAPSLEQILHGPSTMPWGV
ENFEGGLKRALAKCGYTDLKSFQKVSLHVN
>non-antioxidant_1095
MGHHHHHHHHHHSSGHIEGRHMAAVTLHLRAETKPLEARAALTPTTVKKLIAKGFKIYV
EDSPQSTFNINEYRQAGAIIVPAGSWKTAPRDRIIIGLKEMPETDTFPLVHEHIQFAHCYKDQ
AGWQNVLMRFIKGHGTLYDLEFLENDQGRRVAAFGFYAGFAGAALGVRDWAFKQTHSD
DEDLPAVSPYPNEKALVKDVTKDYKEALATGARKPTVLIIGALGRCGSGAIDLLHKVGIPD
ANILKWDIKETSRGGPFDEIPQADIFINCIYLSKPIAPFTNMEKLNNPNRRLRTVVDVSADT
TNPHNPIPIYTVATVFNKPTVLVPTTAGPKLSVISIDHLPSLLPREASEFFSHDLLPSLELLPQ
RKTAPVWVRAKKLFDRHCARVKRSSRL
>non-antioxidant_1096
SNAMVRVDQNLFNEVMYLLDELSQDITVPKNVRKVAQDSKAKLSQENESLDLRCATVLS
MLDEMANDPNVPAHGRTDLYTIISKLEALS
>non-antioxidant_1097
GSGPTYCWNEANNPGGPNRCSNNKQCDGARTCSSSGFCQGTSRKPDPGPKGPTYCWDEA
KNPGGPNRCSNSKQCDGARTCSSSGFCQGTAGHAAA
>non-antioxidant_1098
SNAKNIEETELVVEEMLEQYPNGKIVRLLFHGEQAKLPIISHIVQEYQVEVSIIQGNIQQTK
QGAVGSLYIQLLGEEQNILAAIEGLRKLRVETEVIGNE
>non-antioxidant_1099
MGSSHHHHHHSSGLVPRGSKTFIIGISGVTNSGKTTLAKNLQKHLPNCSVISQDDFFKPESE
IETDKNGFLQYDVLEALNMEKMMSAISCWMESARHSVVSTDQESAEEIPILIIEGFLLFNY
KPLDTIWNRSYFLTIPYEECKRRRSTRVYQPPDSPGYFDGHVWPMYLKYRQEMQDITWE
VVYLDGTKSEEDLFLQVYEDLIQEL
>non-antioxidant_1100

AEEYGYIVTDQKPLSLAAGVKLLEILAEHVHMSSGSFINISVVGPALTFRIRHNEQNLSLAD
VTQQAGLVKSELEAQTGLQILQTGVGQRE
>non-antioxidant_1101
GGSMSKTIVLSVGEATRTLTEIQSTADRQIFEEKVGPLVGRLRLTASLRQNGAKTAYRVNLK
LDQADVVDSGLPKVRYTQVWSHDVTIVANSTEASRKSLYDLTKSLVATSQVEDLVVNLVP
LGR
>non-antioxidant_1102
MMNDGKQQSTFLFHDYETFGTHPALDRPAQFAAIRTDSEFNVIGEPEVFYCKPADDYLPQP
GAVLITGITPQEARAKGENEAAFAARIHSLFTVPKTCILGYNNVRFDDEVTRNIFYRNFYDP
YAWSWQHDNSRWDLLDVMRACYALRPEGINWPENDDGLPSFRLEHLTKANGIEHSNAH
DAMADVYATIAMAKLVKTRQPRLFDYLFTHRNKHKLMALIDVPQMKPLVHVSGMFGAW
RGNTSWVAPLAWHPENRNAVIMVDLAGDISPLLELDSDTLRERLYTAKTDLGDNAAVPVK
LVHINKCPVLAQANTLRPEDADRLGINRQHCLDNLKILRENPQVREKVVAIFAEAEPFTPS
DNVDAQLYNGFFSDADRAAMKIVLETEPRNLPALDITFVDKRIEKLLFNYRARNFPGTLDY
AEQQRWLEHRRQVFTPEFLQGYADELQMLVQQYADDKEKVALLKALWQYADEIVEHHH
HHH
>non-antioxidant_1103
GMSIVGNVENLINGVGELWNKYVKHEFILKMRDGSLPLDIFRYYLIQDGKYVEDMLRALL
IASSKGPIDKVTKILNLVFSSRDKGLETHGKLYSKLDISRDVIVKTGYNLINYAYTRHLYYY
ANLDWNKFLVAWTPCMFGYSIVGDYVIDSPNEVYKTWASFYASTEYKKRIEAILYALDEV
SITEDLLNIFINSVRFEIGFWDASLRKDPTVY
>non-antioxidant_1104
GMEGSLSRGVEIMKLRELVARSRSIRRFDEHVAVNDATLRDLVELVCYTPSAANRQLLRFL
PVTGADMSDKVFPCLKWAGYLEDWPGPEPGERPAAALVMLCRNEDLPGAACDSGIAAQT
IMLGAAEKELGGCIVAAIDRERLMASLGIPDAWTVLLVIALGKPAETVVIDQIKPGDDIRY
WRDKHGIHHVPKRQVDELLVTAEQLRERG
>non-antioxidant_1105
GMVEVSQFKDAMAQLASAVHIVTTSGETGQHGFTASAVCSVTDSPPTLLVCINSNARAYE
HFVKNRVLMVNTLTAEQSSLSNIFASPLSQEERFSNASWTTLTTGSPMLQDALINFDCEITEI
KHVGTHDILICKIVDIHQSNAKNALVYRNRVYHSV
>non-antioxidant_1106
GSREATVLSYDGSMFMKIQLPVVMHTEAEDVSLRFRSQRAYGILMATTSRDSADTLRLEL
DAGRVKLTVNLGKGPETLFAGYNLNDNEWHTVRVVRRGKSLKLTVDDQQAMTGQMAG
DHTRLEFHNIETGIITERRYLSSVPSNFIGHLQSLTFNGMAYIDLCKNGDIDYCELNARFGFR
NI
>non-antioxidant_1107
TATTTAAGATATCTTAAATG
>non-antioxidant_1108
SNADEVENLYTQVADNEYLVQGRMLIDEFNEVFETDLHMSDVDTMAGYLITALGTIPDEG
EKPSFEVGNIKLTAEEMEGTRLLVLRVHFYDEE
>non-antioxidant_1109
GSHMSEWKARRFWASVGIHKEEGGWAVLLDERPLRTPGKQPLRLPTEALALAIAEEWQA
VQEVIDPNAMPLTRSANSAIEKVAPQFDAVAAMLGDYGGTDLLSYRADAPEALVRAQAE
GWDPLIDWAATELRAPLRITHGVIPVPQDPVVLLKLRAEVASLDPFGLTALHDLVTLPGSLI

LGLAVIRGRIDAPTAHALSRIDEEFQAERWGRDEEAEAQAASRLAAMRDSERFWHLTRG
>non-antioxidant_1110
SNAKILAVTACPTGIAHTFMAADALKEKAKELGVEIKVETNGSSGIKHKLTAQEIEDAPAII
VAADKQVEMERFKGKRVLQVPVTAGIRRPQELIEKAMNQDAPIY
>non-antioxidant_1111
MHHHHHHHHHHISSASGESQMSLDEIEDVYHTRPGYRPEEYRWGQGGAKIIDYHIQSAG
VDFPPSLTGNQQTDFLMKVVFEYDFDCVVPGILIKTLDGLFLYGTNSFLASEGRENISVSRG
DVRVFKFSLPVDLNSGDYLLSFGISAGNPQTDMTPLDRRYDSIILHVTKSMDFWGVIDLKS
SFTSYQ
>non-antioxidant_1112
MEEFVNPCKIKVIGVGGGGSNAVNRMYEDGIEGVELYAINTDVQHLSTLKVPNKIQIGEKV
TRGLGAGAKPEVGEEAALEDIDKIKEILRDTDMVFISAGLGGGTGTGAAPVIAKTAKEMGI
LTVAVATLPFRFEGPRKMEKALKGLEKLKESSDAYIVIHNDKIKELSNRTLTIKDAFKEVDS
VLSKAVRGITSIVVTPAVINVDFADVRTTLEEGGLSIIGMGEGRGDEKADIAVEKAVTSPLLE
GNTIEGARRLLVTIWTSEDIPYDIVDEVMERIHSKVHPEAEIIFGAVLEPQEQDFIRVAIVATD
FPEEKFQVGEKEVKFKVIKKLHHHHHH
>non-antioxidant_1113
MGLGRHENAIKYLGQDYENLRARCLQNGVLFQDDAFPPVSHSLGFKELGPNSSKTYGIK
WKRPTELLSNPQFIVDGATRTDICQGALGDCWLLAAIASLTLNETILHRVVPYGQSFQEGY
AGIFHFQLWQFGEWVDVVVDDLLPTKDGKLVFVHSAQGNEFWSALLEKAYAKVNGSYE
ALSGGCTSEAFEDFTGGVTEWYDLQKAPSDLYQIILKALERGSLLGCSINISDIRDLEAITFK
NLVRGHAYSVTDAKQVTYQGQRVNLIRMRNPWGEVEWKGPWSDNSYEWNKVDPYERE
QLRVKMEDGEFWMSFRDFIREFTKLEICNLTPDLEHHHHHH
>non-antioxidant_1114
GQHTLKQFAADSALTTTTPLCSEVPLFDINALGDWTYLGTSLPAKFAKLFASILHCIDDEYF
LITPVEKVRVQVEDAPLLIVDFERAQPHSLLNVSTSIGTLHHNVDIKQMKLTDDSVYLPLE
RGLWGKLGRACYYNFVNEFNLSDLNEQ
>non-antioxidant_1115
GMGILSGNPQDEPLHYGEVFSTWTYLSTNNGLINGYRSFINHTGDEDLKNLIDEAIQAMQ
DENHQLEELLRSNGVGLPPAPPDRPAARLDDIPVGARFNDPEISATISMDVAKGLVTCSQII
GQSIREDVALMFSQFHMAKVQFGGKMLKLNKNKGWLIPPPLHSDRPIKE
>non-antioxidant_1116
MTKALFFDIDGTLVSFETHRIPSSTIEALEAAHAKGLKIFIATGRPKAIINNLSELQDRNLIDG
YITMNGAYCFVGEEVIYKSAIPQEEVKAMAAFCEKKGVPCIFVEEHNISVCQPNEMVKKIF
YDFLHVNVIPTVSFEEASNKEVIQMTPFITEEEEKEVLPSIPTCEIGRWYPAFADVTAKGDT
KQKGIDEIIRHFGIKLEETMSFGDGGNDISMLRHAAIGVAMGQAKEDVKAAADYVTAPID
EDGISKAMKHFGII
>non-antioxidant_1117
LGNFSQACYNSAIQGSVLTSTCIRTNGGYNTSSIDLNSVIENVDGSLKWQGSNFIETCRNTQ
LAGSSELAAECKTRAQQFVSTKINLDDHIAAIDGTLKYELEHHHHHH
>non-antioxidant_1118
GSHMTNVTGDYTDCTPLLGDRAALDSFYEEHGYLFLRNVLDRDLVKTVAEQMREGLVAL
GAADPHATLEELTIDSFESVDEVAMHDYVKYDAFWNNPSTIKVFEQVFGEPVFVFLSTTIR
YYPSQAGSEEPSFHYLTPFHQDGFYIGPNQDFRTFWIPLIRTTRESGGVALADGSHRRGKR

DHVLNESFRRFGHPVRGIPPTEVSEDEHLLHSPMEPGDILLFHAHMCHKSIPNLSKDPRLM
RMSMDTRVQPAKSHRGFNAMTPWTESAKDASKGIMAKITGTPTDVE
>non-antioxidant_1119
GMGKGKSAIESQIRMLKLAKEIVEEVASSFPNLEEVYIFGSRARGDYLDTSDIDILFVFKGI
KEMNVFDRMYMVSRFIRGNVDYIVLDEGEKDRVKDKVLFWKREKGFVLL
>non-antioxidant_1120
VFPSNATFGMGDRVRKKSGAAWQGQIVGWYCTNLTPEGYAVESEAHPGSVQIYPVAALER
IN
>non-antioxidant_1121
GSHNADLSEALRELRRELMKETGYSAFVVFTNATLEALAARQPRTLAELAEVPGLGEKRI
EAYGERILDAINTVLDG
>non-antioxidant_1122
GMSAPVTLINPFKVPADKLEAAIEYWEAHRDFMAQQPGYLSTQLHQSIDEGATYQLINVAI
WQSEADFYQAAQKMRQALGHVQVEGLCGNPALYRVIRT
>non-antioxidant_1123
SQPNATLYKMSSINADFAFNLYRRFTVETPDKNIFFSPVSISAALVMLSFGACCSTQTEIVET
LGFNLTDTPMVEIQHGFQHLICSLNFPKKELELQIGNALFIGKHLKPLAKFLNDVKTLYETE
VFSTDFSNISAAKQEINSHVEMQTKGKVVGLIQDLKPNTIMVLVNYIHFKAQWANPFDPS
KTEDSSSFLIDKTTTVQVPMMHQMEQYYHLVDMELNCTVLQMDYSKNALALFVLPKEG
QMESVEAAMSSKTLKKWNRLLQKGWVDLFVPKFSISATYDLGATLLKMGIQHAYSENAD
FSGLTEDNGLKLSNAAHKAVLHIGEKGTEAAGAMFLEAIPR
>non-antioxidant_1124
SENTFLHPIIQIDRSFMLLILERSTRSILFLGKVVNPTEA
>non-antioxidant_1125
MASKRALVILAKGAEEMETVIPVDVMRRAGIKVTVAGLAGKDPVQCSRDVVICPDASLE
DAKKEGPYDVVVLPGGNLGAQNLSESAAVKEILKEQENRKGLIATICAGPTALLAHEIGFG
SKVTTHPLAKDKMMNGGHYTYSENRVEKDGLILTSRGPGTSFEFALAIVEALNGKEVAAQ
VKAPLVLKDLEHHHHHH
>non-antioxidant_1126
GSTKDELTKIMDRASKIEQIQKLAKYAISALNYEDLPTAKDELTKALDLLNSI
>non-antioxidant_1127
AIDLCGMSQDELNECKPAVSKENPTSPSQPCCTALQHADFACLCGYKNSPWLGSFGVDPE
LASALPKQCGLANAPTC
>non-antioxidant_1128
EVPIVTRAEWNAKPPNGAIDSMVTPLPRAVIAHTAGGACADDVTCSQHMRNLQNFQMSK
QKFSDIGYHYLIGGNGKVYEGRSPSQRGAFAGPNNDGSLGIAFIGNFEERAPNKEALDAA
KELLEQAVKQAQLVEGYKLLGHRQVSATKSPGEALYALIQQWPNWSEEML
>non-antioxidant_1129
MAFKIQLDTLGQLPGLLSIYTQISLLYPVSDSSQYPTIVSTFEQGLKRFSEAVPWVAGQVKA
EGISEGNTGTSFIVPFEDVPRVVVKDLRDDPSAPTIEGMRKAGYPMAMFDENIIAPRKTLPI
GPGTGPDDPKPVILLQLNFIKGGLILTVNGQHGAMDMVGQDAVIRLLSKACRNDPFTEEE
MTAMNLDRKTIVPYLENYTIGPEVDHQIVKADVAGGDAVLTPVSASWAFFTFSPKAMSEL
KDAATKTLDASTKFVSTDDALSAFIWKSASRVRLERIDGSAPTEFCRAVDARPAMGVSNN
YPGLLQNMTYHNSTIGEIANESLGATASRLRSELDPASMRQRTRGLATYLHNNPDKSNVSL

TADADPSTSVMLSSWAKVGLWDYDFGLGLGKPETVRRPIFEPVESLMYFMPKKPDGEFC
AALSLRDEDMDRLKADKEWTKYAQYVG
>non-antioxidant_1130
HHGIRMTRISREMMKELLSVYFIMGSNNTKADPVTVVQKALKGGATLYQFREKGGDALT
GEARIKFAEKAQAACREAGVPFIVNDDVELALNLKADGIHIGQEDANAKEVRAAIGDMIL
GVSAHTMSEVKQAEEDGADYVGLGPIYPTETKKDTRAVQGVSLIEAVRRQGISIPIVGIGGI
TIDNAAPVIQAGADGVSMISAISQAEDPESAARKFREEIQTYKTGR
>non-antioxidant_1131
MAHHHHHHEFPKPEFMSKSLEELQIGTYANIAMVRTTTPVYVALGIFVQHRVSALPVVDE
KGRVVDIYSKFDVINLAAEKTYNNLDVSVTKALQHRSHYFEGVLKCYLHETLETIINRLVE
AEVHRLVVVDENDVVKGIVSLSDILQALVLT
>non-antioxidant_1132
SVLPETPVPFKSGTGAIDNDTVYIGLGSAGTAWYKLDTQAKDKKWTALAAFPGGPRDQA
TSAFIDGNLYVFGGIGKNSEGLTQVFNDVHKYNPKTNSWVKLMSHAPMGMAGHVTFVH
NGKAYVTGGVNQNIFNGYFEDLNEAGKDSTAIDKINAHYFDKKAEDYFFNKFLLSFDPST
QQWSYAGESPWYGTAGAAVVNKGDKTWLINGEAKPGLRTDAVFELDFTGNNLKWNKLA
PVSSPDGVAGGFAGISNDSLIFAGGAGFKGSRENYQNGKNYAHEGLKKSYSTDIHLWHNG
KWDKSGELSQGRAYGVSLPWNNSLLIIGGETAGGKAVTDSVLITVKDNKVTVQNLEHHH
HHH
>non-antioxidant_1133
ERCGEQGSNMECPNNLCCSQYGYCGMGGDYCGKGCQNGACWTSKRCGSQAGGATCTN
NQCCSQYGYCGFGAEYCGAGCQGGPCRADIKCGSQAGGKLCPNNLCCSQWGFCGLGSE
FCGGGCQSGACSTDKPCGKDAGGRVCTNNYCCSKWGSCGIGPGYCGAGCQSGGCDG
>non-antioxidant_1134
MRHGDISSSNDTVGVAVVNYKMPRLHTAAEVLDNARKIAEMIVGMKQGLPGMDLVVFPE
YSLQGIMYDPAEMMETAVAIPGEETEIFSRACRKANVWGVFSLTGERHEEHPRKAPYNTL
VLIDNNGEIVQKYRKIIPWCPIEGWYPGGQTYVSEGPKGMKISLIICDDGNYPEIWRDCAM
KGAELIVRCQGYMYPAKDQQVMMAKAMAWANNCYVAVANAAGFDGVYSYFGHSAIIG
FDGRTLGECGEEEMGIQYAQLSLSQIRDARANDQSQNHLFKILHRGYSGLQASGDGDRGL
AECPFEFYRTWVTDAEKARENVERLTRSTTGVAQCPVGRLPYEG
>non-antioxidant_1135
MTFRNCVAVDLGASSGRVMLARYERECRSLTLREIHRFNNGLHSQNGYVTWDVDSLESAI
RLGLNKVCAAGIAIDSIGIDTWGVDFVLLDQQGQRVGLPVAYRDSRTNGLMAQAQQQLG
KRDIYQRSGIQFLPFNTLYQLRALTEQQPELIPHIAHALLMPDYFSYRLTGKMNWEYTNAT
TTQLVNINSDDWDESLLAWSGANKAWFGRPTHPGNVIGHWICPQGNEIPVVAVASHDTAS
AVIASPLNGSRAAYLSSGTWSLMGFESQTPFTNDTALAANITNEGGAEGRYRVLKNIMGL
WLLQRVLQERQINDLPALIAATQALPACRFIINPNDDRFINPDEMCSEIQAACREMAQPIPES
DAELARCIFDSLALLYADVLHELAQLRGEDFSQLHIVGGGCQNTLLNQLCADACGIRVIAG
PVEASTLGNIGIQLMTLDELNNVDDFRQVVSTTANLTTFTPNPDSEIAHYVALIHSTRQTKE
LCA
>non-antioxidant_1136
MSTLEQTIGNTPLVKLQRMGPDNGSEVWLKLEGNNPAGSVKDRAALSMIVEAEKRGEIKP
GDVLIEATSGNTGIALAMIAALKGYRMKLLMPDNMSQERRAAMRAYGAELILVTKEQGM
EGARDLALEMANRGEGKLLDQFNNPDNPYAHYTTTGPEIWQQTGGRITHFVSSMGTTGTI

TGVSRFMREQSKPVTIVGLQPEEGSSIPGIRRWPTEYLPGIFNASLVDEVLDIHQRDAENTM
RELAVREGIFCGVSSGGAVAGALRVAAANPDAVVVAIICDRGDRYLSTGVFGEEHFSQGAG
I
>non-antioxidant_1137
MSSSHKSWNSIYEFTVKDINGVDVSLEKYRGHVCLIVNVACKCGATDKNYRQLQEMHTR
LVGKGLRILAFPCNQFGGQEPWAEAEIKKFVTEKYGVQFDMFSKIKVNGSDADDLYKFLK
SRQHGTLTNNIKWNFSKFLVDRQGQPVKRYSPTTAPYDIEGDIMELLEKK
>non-antioxidant_1138
GMERGIVQYDFMAESQDELTIKSGDKVYILDDKKSKDWWMCQLVDSGKSGLVPAQFIEP
V
>non-antioxidant_1139
SSQIRQNYSTEVEAAVNRLVNLYLRASYTYLSLGFYFDRDDVALEGVCHFFRQLAQQKMQ
GAERLLKMQNQRGGRALFQDLQKPSQDEWGTTPDAMKAAIVLEKSLNQALLDLHALGS
AQADPHLCDFLESHFLDEEVKLIKKMGDHLTNIQRLVGSQAGLGEYLFERLTLKHD
>non-antioxidant_1140
EAPTIIDLTCTVATCTHSSDFGGVLTLTYKTNKNGDCSVHSHSNVATLQEATAKVKTAGKV
TLHFSTASASPSFVVSLCSARATCSASCEP
>non-antioxidant_1141
MASNYNSGLKIGAWVGTQPSESAIKSFQELQGRKLDIVHQFINWSTDFSWVRPYADAVYN
NGSILMITWEPWEYNTVDIKNGKADAYITRMAQDMKAYGKEIWLRPLHEANGDWYPWA
IGYSSRVNTNETYIAAFRHIVDIFRANGATNVKWVFNVNCDNVGNGTSYLGHYPGDNYV
DYTSIDGYNWGTTQSWGSQWQSFDQVFSRAYQALASINKPIIIAEFASAEIGGNKARWITE
AYNSIRTSYNKVIAAVWFHENKETDWRINSSPEALAAYREAIGAG
>non-antioxidant_1142
ESACTLQSETHPPLTWQKCSSGGTCTQQTGSVVIDANWRWTHATNSSTNCYDGNTWSST
LCPDNETCAKNCCLDGAAYASTYGVTTSGNSLSIGFVTQSAQKNVGARLYLMASDTTYQ
EFTLLGNEFSFDVDVSQLPCGLNGALYFVSMDADGGVSKYPTNTAGAKYGTGYCDSQCP
RDLKFINGQANVEGWEPSSNNANTGIGGHGSCCSEMDIWEANSISEALTPHPCTTVGQEIC
EGDGCGGTYSDNRYGGTCDPDGCDWNPYRLGNTSFYGPGSSFTLDTTKKLTVVTQFETS
GAINRYYVQNGVTFQQPNAELGSYSGNELNDDYCTAEEAEFGGSSFSDKGGLTQFKKATS
GGMVLVMSLWDDYYANMLWLDSTYPTNETSSTPGAVRGSCSTSSGVPAQVESQSPNAKV
TFSNIKFGPIGSTGNPSG
>non-antioxidant_1143
PVFENNNQRYYESLPFKQLKELKIACSQYGPTAPFTIAMIENLGTQALPPNDWKQTARACL
SGGDYLLWKSEFFEQCARIADVNRQQGIQTSYEMLIGEGPYQATDTQLNFLPGAYAQISNA
ARQAWKRLPSLEHHHHHH
>non-antioxidant_1144
PLGSQFWVTVQRTEAAERCGLHGSYVLRVEAERLTLLTVGAQSQILEPLLSWPYTLLRRY
GRDKVMFSFEAGRRCPSGPGTFTFQTAQGNDIFQAVETAIHRQKAQG
>non-antioxidant_1145
MATVYDVPGDLLVERVAQRLKEIPEIKPPEWAPFVKTGRHKERLPEQEDWWYYRVASILR
RVYLDGPVGIERLRTYYGGRKNRGHAPERFYKAGGSIIRKALQQLEAAGFVEKVPGKGRV
ITPKGRSFLDKIATELKKELEEIIPELKKY
>non-antioxidant_1146

GPLGSPEFGYWITCCPTCDVDINTWVPFYSTELNKPAMIYCSHGDGHWVHAQCMDLEER
TLIHLSEGSNKYYCNEHVQIARA
>non-antioxidant_1147
DRLTVVKQYVDNVLNKASDTYHGDKPSPLLADGVDPRTGQQLEWIFPDGRRAVLSNFSA
QQNLMRVMSGLSQLSGDPRYQKRAEDIVRYHFQNYQDPSGLLYWGGHRFVDLKTLQPE
GPSEKEMVHELKNAYPYYDLMFSVDSDATARFIRGFWNAHVYDWRILETSRHGEYGKPM
GALWESKFEQQPPFFATKGLSFLNAGNDLIYSASLLYKHQQDQGALTWAKRLADQYVLPR
DAKTGLGVYQFTQALKREEPTDDADTHSKFGDRAQRQFGPEFGPTALEGNMMLKGRTST
LYSENALMQLQLGKDLGPQGQDLLKWTVDGLKAFAKYAYNDQDNTFRPMIANGQDLSN
YTLPRDGYYGKKGTVLKPYKAGNEFLISYARAYAIDNDPLLWKVARGIANDQGLGDIGTA
PGKEVKVNMDTTNSDPYALFALLDLYHASQVADYRKLAEKIGDNIIKIRYIDGFFMASSDR
QYADVDAIEPYALLALEASLRNKPQAVAPFLNGAGFTEGAYRMDDGSARVSTRDNELFLL
NVGEKLQPN
>non-antioxidant_1148
MFLRIDRLQIELPMPKEQDPNAAAAVQALLGGRFGEMSTLMNYMYQSFNFRGKKALKPY
YDLIANIATEELGHIELVAATINSLLAKNPGKDLEEGVDPASTPLGFAKDVRNAAHFIAGGA
NSLVMGAMGEHWNGEYVFTSGNLILDLLHNFFLEVAARTHKLRVYEMTDNPVAREMIGY
LLVRGGVHAAAYGKALESLTGVEMTKMLPIPKIDNSKIPEAKKYMDLGFHRNLYRFSPED
YRDLGLIWKGASPEDGTEVVVVDGPPTGGPVFDAGHDAAEFAPEFHPGELYEIAKKLYEK
AK
>non-antioxidant_1149
MQNITYSWFVQGMIKATTDAWLKGWDERNGGNLTLRLDDADIAPYHDNFHQQPRYIPLS
QPMPLLANTPFIVTGSGKFFRNVQLDPAANLGIVKVDSDGAGYHILWGLTNEAVPTSELPA
HFLSHCERIKATNGKDRVIMHCHATNLIALTYVLENDTAVFTRQLWEGSTECLVVFPDGVG
ILPWMVPGTDAIGQATAQEMQKHSLVLWPFHGVFGSGPTLDETFGLIDTAEKSAQVLVKV
YSMGGMKQTISREELIALGKRFGVTPLASALAL
>non-antioxidant_1150
GSPEKILAQIIQEHREGLDWQEAATRASLSLEETRKLLQSMAAAGQVTLLRVENDLYAIST
ERYQAWWQAVTRALEEFHSRYPLRPGLAREELRSRYFSRLPARVYQALLEEWSREGRLQL
AANTVALAGFTPSF
>non-antioxidant_1151
KVFGRCELAAAMKRHGLDNYRGYSLGNWVCAAKFESNFNTQATNRNTDGSTDYGILQI
NSRWWCNDGRTPGSRNLCNIPCSALLSSDITASVNCAKKIVSDGNGMNAWVAWRNRCKG
TDVQAWIRGCRL
>non-antioxidant_1152
DPDQFGPDLIEQLAQSGKYSQDNTKGDAMIGVKQPLPKAVLRTQHDKNKEAISILDFGVID
DGVTDNYQAIQNAIDAVASLPSGGELFIPASNQAVGYIVGSTLLIPGGVNIRGVGKASQLRA
KSGLTGSVLRLSYDSDTIGRYLRNIRVTGNNTCNGIDTNITAEDSVIRQVYGWVFDNVMV
NEVETAYLMQGLWHSKFIACQAGTCRVGLHFLGQCVSVSVSSCHFSRGNYSADESFGIRI
QPQTYAWSSEAVRSEAIILDSETMCIGFKNAVYVHDCLDLHMEQLDLDYCGSTGVVIENV
NGGFSFSNSWIAADADGTEQFTGIYFRTPTSTQSHKIVSGVHINTANKNTAANNQSIAIEQS
AIFVFVSGCTLTGDEWAVNIVDINECVSFDKCIFNKPLRYLRSGGVSVTDCYLAGITEVQKP
EGRYNTYRGCSGVPSVNGIINVPVAVGATSGSAAIPNPGNLTYRVRSLFGDPASSGDKVSVS
GVTINVTRPSPVGVALPSMVEYLAI

>non-antioxidant_1153
GAMATDWLGSIVSINCGDSLGVYQGRVSAVDQVSQTISLTRPFHNGVKCLVPEVTFRAGDI
TELKILEIPGPGDNQHFGDLHQT
>non-antioxidant_1154
GSGEGSEDDPLYDEAVRFVTESRRASISAVQRKLKIGYNRAARMIEAMEMAGVVTPMNT
NGSREVIAPAPVRD
>non-antioxidant_1155
MSVEKIPGYTYGETENRAPFNLEDLKLLKEAVMFTAEDEEYIQKAGEVLEDQVEEILDTW
YGFVGSHPHLLYYFTSPDGTPNEKYLAAVRKRFSRWILDTSNRSYDQAWLDYQYEIGLRH
HRTKKNQTDNVESVPNIGYRYLVAFIYPITATMKPFLARKGHTPEEVEKMYQAWFKATTL
QVALWSYPYVKYGDF
>non-antioxidant_1156
YQPNYFLSIPITNKKITAGIKVLQNSILRQDNRLTKAMVGDGSFHITLLVMQLLNEDEVNIG
TDALLELKPFVEEILEGKHLTLPFHGIGTFQGQVGFVKLADGDHVSALLEIAETAKRTFQE
KGILAGESRTFKPHLTFMKLSKAPMLWKKGVRKIEPGLYEQFIDHRFGEEILYQIDLCSML
KKKQSNGYYHCESSIVIGEKD
>non-antioxidant_1157
MDPDQYSIEADKKFKYSLKLSDYPTLQDAASAAVDGLLIDRDYNFYGGETVDFGGKVLTI
ECKAKFIGDGNLIFTKLGKGSRIAGVFMESTTTPWVIKPWTDDNQWLTDAAAVVATLKQS
KTDGYQPTVSDYVKFPGIETLLPPNAKGQNITSTLEIRECIGVEVHRASGLMAGFLFRGCH
FCKMVDANNPSGGKDGIITFENLSGDWGKGNYVIGGRTSYGSVSSAQFLRNNGGFERDG
GVIGFTSYRAGESGVKTWQGTVGSTTSRNYNLQFRDSVVIYPVWDGFDLGADTDMNPEL
DRPGDYPITQYPLHQLPLNHLIDNLLVRGALGVGFGMDGKGMYVSNITVEDCAGSGAYL
LTHESVFTNIAIIDTNTKDFQANQIYISGACRVNGLRLIGIRSTDGQSLTIDAPNSTVSGITGM
VDPSRINVANLAEEGLGNIRANSFGYDSAAIKLRIHKLSKTLDSGALYSHINGGAGSGSAY
TQLTAISGSTPDAVSLKVNHKDCRGAEIPFVPDIASDDFIKDSSCFLPYWENNSTSLKALVK
KPNGELVRLTLATL
>non-antioxidant_1158
AFACSFPPSEIPGSKECLAEALQKHQGFKKKSYALICAYLNYKEDAENYERAAEDFDSAVK
CTGCKEGVDLHEGNPELIEEGFEKFLASLKIDRKALGSLCTLFQKLYAIPHN
>non-antioxidant_1159
DAAPAAGSTLDKIAKNGVIVVGHRESSVPFSYYDNQQKVVGYSQDYSNAIVEAVKKKLN
KPDLQVKLIPITSQNRIPLLQNGTFDFECGSTTNNVERQKQAAFSDTIFVVGTRLLTKKGG
DIKDFADLKGKAVVVTSGTTSEVLLNKLNEEQKMNMRIISAKDHGDSFRTLESGRAVAFM
MDDALLAGERAKAKKPDNWDIVGKPQSQEAYGCMLRKDDPQFKKLMDDTIAQVQTSG
EAEKWFDKWFKNPIPPKNLNMNFELSDEMKALFKEPNDKALNLEHHHHHH
>non-antioxidant_1160
ATFEIVNRCSYTVWAAASKGDAALDAGGRQLNSGESWTINVEPGTNGGKIWARTDCYFD
DSGSGICKTGDCGGLLRCKRFGRPPTTLAEFSLNQYGKDYIDISNIKGFNVPMNFSPTTRG
CRGVRCAADIVGQCPAKLKAPGGGCNDACTVFQTSEYCCTTGKCGPTEYSRFFKRLCPD
AFSYVLDKPTTVTCPGSSNYRVTFCPT
>non-antioxidant_1161
DPDQFRAIIESPEGAGHVGYQYRRNTGSTMRMVSDVLDERVSLWDFHCDPSGNVIQPGPN
VDSRQYLQAAIDYVSSNGGGTITIPAGYTWYLGSYGVGGIAGHSGIIQLRSNVNLNIEGRI

HLSPFFDLKPFQVFVGFDNGDPASSGNLENCHIYGHGVVDFGGYEFGASSQLRNGVAFGR
SYNCSVTGITFQNGDVTWAITLGWNGYGSNCYVRKCRFINLVNSSVNADHSTVYVNCPY
SGVESCYFSMSSSFARNIACSVELHQHDTFYRGSTVNGYCRGAYVVMHAAEAAGAGSYA
YNMQVENNIAVIYGQFVILGSDVTATVSGHLNDVIVSGNIVSIGERAAFSAPFGAFIDIGPD
NSGASNVQDIQRVLVTGNSFYAPANITDSAAITLRANLNGCTFIANNFDCRYMVYNAPGTT
SPVVQNLVWDKSNVIGGTHANQRAGQNLFDMQFASVVNSTIEVQLSCEDLSMFSCILFPA
SCQLSYSKITVDSAWTKSMSNTAVFEGNQQAGANVYVSYPATVNLTSYNTQGAVPFFSTD
TNYAWVTSAYSLSINENLDFSPPATYTNKANGQLVGVGYNEIGGVRSVSVRLMLQRQV
>non-antioxidant_1162
MSEITLGKYLFERLKQVNVNTVFGLPGDFNLSLLDKIYEVEGMRWAGNANELNAAYAAD
GYARIKGMSCIITTFGVGELSALNGIAGSYAEHVGVLHVVGVPSISAQAKQLLLHHTLGNG
DFTVFHRMSANISETTAMITDIATAPAEIDRCIRTTYVTQRPVYLGLPANLVDLNVPAKLLQ
TPIDMSLKPNDAESEKEVIDTILVLDKDAKNPVILADACCSRHDVKAETKKLIDLTQFPAFV
TPMGKGSIDEQHPRYGGVYVGTLSKPEVKEAVESADLILSVGALLSDFNTGSFSYSYKTK
NIVEFHSDHMKIRNATFPGVQMKFVLQKLLTTIADAAKGYKPVAVPARTPANAAVPASTPL
KQEWMWNQLGNFLQEGDVVIAETGTSAFGINQTTFPNNTYGISQVLWGSIGFTTGATLGA
AFAAEEIDPKKRVILFIGDGSLQLTVQEISTMIRWGLKPYLFVLNNDGYTIQKLIHGPKAQY
NEIQGWDHLSLLPTFGAKDYETHRVATTGEWDKLTQDKSFNDNSKIRMIEVMLPVFDAPQ
NLVEQAKLTAATNAKQ
>non-antioxidant_1163
MNYNPEEQFRCTIIRGKAKNMLDNLLPAYANIIDDICPCDKASFVKDFNNRLIEILGEETTK
KTLDNHRTEIAGKLFGMFYEDDEVIFPSGRTNKYIEDSDQPAFFKDICFKFQFPNGMDKLD
KVIEKVGAKIQIRQFPYILQVLLTADNNNIQLSKDDIAYYVLNSLQVLQGKIKPIEVIEKIIED
RSNDITKKVRHPGKETSYSMQHIREQLNYLELANLIRIDGNLVKLNYREAENINYIAQFWG
NKPEFNAYKYDFTSEDDKKSFFKDWQQYYSNVNSHKS
>non-antioxidant_1164
MELKHSISDYTEAEFLQLVTTICNADTSSEEELVKLVTHFEEMTEHPSGSDLIYYPKEGDDD
SPSGIVNTVKQWRAANGKSGFKQG
>non-antioxidant_1165
MESKRNKPGKATGKGKPVGDKWLDDAGKDSGAPIPDRIADKLRDKEFKSFDDFRKAVW
EEVSKDPELSKNLNPSNKSSVSKGYSPATPKNQQVGGRKVYELHHDKPISQGGEVYDMD
NIRVTTPKRHIDIHRGK
>non-antioxidant_1166
TVLPKDIPGDSLKVTVGTANGKPGDTVTVPVTFADVAKMKNVGTCNFYLGYDASLLEVV
SVDAGPIVKNAAVNFSSSASNGTISFLFLDNTITDELITADGVFANIKFKLKSVTAKTTTPVT
FKDGGAFGDGTMSKIASVTKTNGSVTIDP
>non-antioxidant_1167
VIVYGDYNNDGNVDSTDFAGLKKYIMAADHAYVKNLDVNLDNEVNAFDLAILKKYLLG
MVSKLE
>non-antioxidant_1168
EVYALEESRDVYLSDLDWLNATHGDDTKSKIVQKNHPFTPGNNNQSTKISLKMEDGSISE
FEKGLGTIAGSPSTITYDISGAGVTKFFSYLGIDRSANPINEQYAKVDKIEVVVDGKVIYSTI
NQFPNGLTYETPAIKVDLNIPENAKRLQLKSYAGEKTWGDEVVYADAKFTAKGDFVN
>non-antioxidant_1169

AHHHHHHMVHITLDRNTANSWLIISKDRRQVRMGDTHQNVSDNKERFSNYPMVLGAQR
FSSGKMYWEVDVTQKEAWDLGVCRDSVQRKGQFSLSPENGFWTIWLWQDSYEAGTSPQ
TTLHIQVPPCQIGIFVDYEAGVVSFYNITDHGSLIYTFSECVFAGPLRPFFNVGFNYSGGNA
APLKLCPLKM
>non-antioxidant_1170
GLDTLDRDGDGSTADADCNDFAPTIHPGAAEATLDGVDSNCDGRDSGVAEVVETFKNPG
TYSSPVINFKIASPPGPGTPIYGPPRDFSGYNKSYSLAIGKTSYYDPTTGTKWNDDTITPVS
DGQDIWRGMTHTGKWSFFNGKAGDKITLSVQRDAQEASLKGAHPGFILFWRPEGGPLFW
AGTQDLDEGQTALPADSDTVIGHVIVQHADWTLQGLPPKADHTAPAGVDTELYPMKPDS
YTMYYVDSGYDADKYVASKKLIMHPTAFKGLALNDGTAGAFTKSITLPKTGYYMLYVAN
VLEVDDWSVDADGKLTTTGEVWEVPAKGCWVNITISKPAEDLDLGFISPDQYTTPDIVCH
KNAAPGAISATAAAGSNIVFQWGPGVWPHPYGPIVTYVVECSGSCTTVNKNNLRWVKIQ
EAGINYNTQVWAQQDLINQGNKWTVKIPSSLRPGNYVFRHELLAAHGASSANGMQNYP
QCVNIAVTGSGTKALPAGTPATQLYKPTDPGILFNPYTTITSYTIPGPALWQG
>non-antioxidant_1171
MSYYHHHHHHLESTSLYKKAGMSDFYDPRERDPSVSRRPQNRQSDEWIRELLLRGTIARV
ATLWQGEDGAAFPFITPLAYAYRPEQGDLVYHTNVVGRLRANAGQGHPATLEVSEIGQFLP
SNSPLELSVQYRSVMVFGTARVLAGEDARAALTTLSERVFPGLKVGETTRPISEDDLKRTS
VYSLSIDRWSGKENWAEQAIQEEDWPALGPEWLG
>non-antioxidant_1172
GHSSSDPVYPCGICTNEVNDDQDAILCEASCQKWFHRICTGMTETAYGLLTAEASAVWGC
DTCMA
>non-antioxidant_1173
AMAAKVVYVFSTEMANKAAEAVLKGQVETIVSFHI
>non-antioxidant_1174
AEKANQVSNIKTQLAMEYMRGQDYRQATASIEDALKSDPKNELAWLVRAEIYQYLKVND
KAQESFRQALSIKPDSAEINNNYGWFLCGRLNRPAESMAYFDKALADPTYPTPYIANLNK
GICSAKQGQFGLAEAYLKRSLAAQPQFPPAFKELARTKMLAGQLGDADYYFKKYQSRVE
VLQADDLLLGWKIAKALGNAQAAYEYEAQLQANFPYSEELQTVLTGQ
>non-antioxidant_1175
ASIPSSASVQLDSYNYDGSTFSGKIYVKNIAYSKKVTVVYADGSDNWNNNGNIIAASFSGP
ISGSNYEYWTFSASVKGIKEFYIKYEVSGKTYYDNNNSANYQVST
>non-antioxidant_1176
EMETYVNKLHEGSTYTAAVQYNVLEKDDDPASLTIWVPMFQSSMPADLLIKELANVNILV
KQISTPKGPSLRVMINSRSAVLAQMPSKFTICANVSLDDRSKLAYDVTTPCEIKACSLTCLK
SKNMLTTVKDLTMKTLNPTHDIIALCEFENIVTSKKVIIPTYLRSISVRNKDLNTLENITTTE
FKNAITNAKIIPYSGLLLVITVTDNKGAFKYIKPQSQFIVDLGAYLEKESIYYVTTNWKHTA
TRFAIKPRED
>non-antioxidant_1177
MAMIKMSPEEIRAKSQSYGQGSDQIRQILSDLTRAQGEIAANWEGQAFSRFEEQFQQLSPK
VEKFAQLLEEIKQQLNSTADAVQEQDQQLSNNFGLQ
>non-antioxidant_1178
MNALLSNPFKERLRKGEVQIGLWLSSTTAYMAEIAATSGYDWLLIDGEHAPNTIQDLYHQ
LQAVAPYASQPVIRPVEGSKPLIKQVLDIGAQTLLIPMVDTAEQARQVVSATRYPPYGERG

VGASVARAARWGRIENYMAQVNDSLCLLVQVESKTALDNLDEILDVEGIDGVFIGPADLS
ASLGYPDNAGHPEVQRIIETSIRRIRAAGKAAGFLAVAPDMAQQCLAWGANFVAVGVDTM
LYSDALDQRLAMFKSGKNGPRIKGSY
>non-antioxidant_1179
MSAKPQPIAAANWKCNGTTASIEKLVQVFNEHTISHDVQCVVAPTFVHIPLVQAKLRNPK
YVISAQNAIAKSGAFTGEVSMPILKDIGVHWVILGHSERRTYYGETDEIVAQKVSEACKQG
FMVIACIGETLQQREANQTAKVVLSQTSAIAAKLTKDAWNQVVLAYEPVWAIGTGKVATP
EQAQEVHLLLRKWVSENIGTDVAAKLRILYGGSVNAANAATLYAKPDINGFLVGGASLKP
EFRDIIDATR
>non-antioxidant_1180
GEINGPESVLVNTYQWIIRNWETVKIQWSQNPTMLYNKMEFEPFQSLVPKAIRGQYSGFVR
TLFQQMRDVLGTFDTTQIIKLLPFAAAPPEQSRMQFSSLTVNVRGSGMRILVRGNSPVFNY
NKTTKRLTILGKDAGTLIEDPDESTSGVESAVLRG
>non-antioxidant_1181
SGRHERDAFDTLFDHAPDKLNVVKKTLITFVNKHLNKLNLEVTELETQFADGVYLVLLM
GLLEGYFVPLHSFFLTPDSFEQKVLNVSFAFELMQDGGLEKPKPRPEDIVNCDLKSTLRVL
YNLFTKYRNVE
>non-antioxidant_1182
SDPVDYQAEDATIVQGAVESNHAGYTGTGFVNYDNVAGSSVEWTVTVPSAGTYDVVVRY
ANGTTTSRPLDFSVNGSISASGVAFGSTGTWPAWTTKTVRVTLAAGVNKIKAVATTANGGP
NVDKITL
>non-antioxidant_1183
ERFSPRYIELAVVADHGIFTKYNSNLNTIRTRVHEMLNTVNGFYRSVDVHAPLANLEVWS
KQDLIKVQKDSSKTLKSFGEWRERDLLPRISHDHAQLLTAVVFDGNTIGRAYTGGMCDPR
HSVGVVRDHSKNNLWVAVTMAHELGHNLGIHHDTGSCSCGAKSCIMASVLSKVLSYEFS
DCSQNQYETYLTNHNPQCILNKP
>non-antioxidant_1184
ESNQQIADFDKEKATLDEADIDERMKLAQAFNDSLNNVVSGDPWSEEMKKKGRAEYAR
MLEIHERMGHVEIPVIDVDLPVYAGTAEEVLQQGAGHLEGTSLPIGGNSTHAVITAHTGLP
TAKMFTDLTKLKVGDKFYVHNIKEVMAYQVDQVKVIEPTNFDDLLIVPGHDYVTLLTCTP
YMINTHRLLVRGHRIPYVAEVEEEFIAANKLSH
>non-antioxidant_1185
GDFAKEYADALYDSLGHSVLICDRDVYIAVSGSSKKDYLNKSISEMLERTMDQRSSVLESD
AKSVQLVNGIDEDMNSYTVGPIVANGDPIGAVVIFSKDQTMGEVEHKAVETAAGFLARQM
EQ
>non-antioxidant_1186
MPDIENQAADGQAEIKPEDAPYITNAYKPAYARWGFGSDSVRNHFIAMSGEFVGTFLFLW
SAFVIAQIANQAPETPDGGSNPAQLIMISFGFGFGVMVGVFITYRVSGGNLNPAVTLALVLA
RAIPPFRGILMAFTQIVAGMAAAGAASAMTPGEIAFANALGGGASRTRGLFLEAFGTAILC
LTVLMLAVEKHRATWFAPFVIGIALLIAHLICIYYTGAGLNPARSFGPAVAARSFPNYHWIY
WLGPILGAFLAYSIWQMWKWLNYQTTNPGQDSDA
>non-antioxidant_1187
ATYHLEDNWVGSAFLSTFTHEAIADPTHGRVNYVDQATALAKNLTYASGDTLILRADHTT
TLSPSGPGRNSVRIRSIKTYTTHVAVFDVRHMPQGCGTWPAAWETDEGDWPNGGEVDIIE

GVNDQSPNAMTLHTGANCAMPASRTMTGHATNNNCDVNTDGNTGCGVQAPTANSYGPS
FNANGGGWYAMERTNSFIKVWFFPRNAGNVPNDIASGPATINTDNWGTPTAFFPNTNCDI
GSHFDANNIIINLTFCGDWAGQASIFNGAGCPGSCVDYVNNNPSAFANAYWDIASVRVYQ
>non-antioxidant_1188
GAMDPDLEATLRAIVHSATSLVDARYGAMEVHDRQHRVLHFVYEGIDEETVRRIGHLPKG
LGVIGLLIEDPKPLRLDDVSAHPASIGFPPYHPPMRTFLGVPVRVRDESFGTLYLTDKTNGQ
PFSDDDEVLVQALAAAAGIAVANARLYQQAK
>non-antioxidant_1189
PLGSMPFHAEPLKPSDEIDMDLGHSVAAQKFKEIREVLEGNRYWARKVTSEEPEFMAEQV
KGQAPNFLWIGCADSRVPEVTIMARKPGDVFVQRNVANQFKPEDDSSQALLNYAIMNVG
VTHVMVVGHTGCGGCIAAFDQPLPTEENPGGTPLVRYLEPIIRLKHSLPEGSDVNDLIKEN
VKMAVKNVVNSPTIQGAWEQARKGEFREVFVHGWLYDLSTGNIVDLNVTQGPHPFVDD
RVPRA
>non-antioxidant_1190
GSMNVILSIDQSTQSTKVFFYDEELNIVHSNNLNHEQKCLKPGWYEHDPIEIMTNLYNLM
NEGIKVLKDKYTSVIIKCIGITNQRETVIIWDRITGKPLYNAIVWLDTRVEELVTEFSAKYN
NNDIQKKTGTYFNTYFSAFKILWLIQNNPEIKQKIDDGTAVIGNINTWLIFNLTKGNCYTDV
TNASRTLLMDINTLQWDEKMCKIFNITNMSVLPEIKSNCSNFGLVKSEHVPDYLNIPITGCI
GDQQSACIGQAIFDEGEAKCTYGTGVFLLINTGEKVVYSTCGLITTICYKFNDNDKPKYAL
EGSIGTAGSGVSWLLKNKLIDDPSEASDIMEKCENTTGVIFVPAFSGLYAPRWRSDARASIY
GMTFNTERSHIVRALLEGIAFQLNEIVDSLTSDMGIEMLHVLRCDGGMTKNKPFMQFNSD
IINTKIEVSKYKEVTSLGAAVLAGLEVKIWDSLDSVKSLLRRSDAVFHSKMDDKKRKKKT
SEWNKAVERTLIQL
>non-antioxidant_1191
RPGADCEVCKEFLNRFYKSLIDRGVNFSLDTIEKELISFCLDTKGKENRLCYYLGATKDAA
TKILSEVTRPMSVHMPAMKICEKLKKLDSQICELKYEKTLD
>non-antioxidant_1192
SEDDLTKVLNYTKQRQTEPNPEYYGVAKKKNIIKIHLESFQTFLINKKVNGKEVTPFLNKL
SSGKEQFTYFPNFFHQTGQGKTSDSEFTMDNSLYGLPQGSAFSLKGDNTYQSLPAILDQKQ
GYKSDVMHGDYKTFWNRDQVYKHFGIDKFYDATYYDMSDKNVVNLGLKDKIFFKDSA
NYQAKMKSPFYSHLITLTNHYPFTLDEKDATIEKSNTGDATVDGYIQTARYLDEALEEYIN
DLKKKGLYDNSVIMIYGDHYGISENHNNAMEKLLGEKITPAKFTDLNRTGFWIKIPGKSG
GINNEYAGQVDVMPTILHLAGIDTKNYLMFGTDLFSKGHNQVVPFRNGDFITKDYKYVN
GKIYSNKNNELITTQPADFEKNKKQVEKDLEMSDNVLNGDLFRFYKNPDFKKVNPSKYK
YETGPK
>non-antioxidant_1193
GPLGSSDGAVTLQEYLELKKALATSEAKVQQLMKVNSSLSDELRKLQREIHKLQAENLQL
RQP
>non-antioxidant_1194
GAMGNLRLIGVPESDVENGTKLENTLQDIIQENFPNLARQANVQIQEIQRTPQRYSSRRATP
RHIIVRFTKVEMKEKMLRAAREKGRVTLKGKPIRLTVD
>non-antioxidant_1195
GSHMKITYPLPPNLPEQLPLLTNCQLEDEAILENHLYQQIDLPNQEVRNLVFRDAVFDHLSL
ANGQFASFDCSNVRFEACDFSNVEWLSGSFHRVTFLRCNLTGTNFADSYLKDCLFEDCKA

DYASFRFANFNLVHFNQTRLVESEFFEVTWKKLLLEACDLTESNWLNTSLKGLDFSQNTFE
RLTFSPNYLSGLKVTPEQAIYLASALGLVIT
>non-antioxidant_1196
FNCNKREGPCSQRSLCECDPNLQLGRHSDQLWHYNLRTNRCERGGYRDNCNSHSSSGAC
VMACERIHHHHHH
>non-antioxidant_1197
GSHMKTLKPKEIKFNSWEELLKWEPGAREDDAINRGSVVLASRRTGHLVNEKASKEAKV
QALSNTNSKAKDHASVGGEEFKAYAFDYWQYLDSMVFWEGLVPTPDVIDAGHRNGVPV
YGTLFFNWSNSIADQERFAEALKQDADGSFPIARKLVDMAKYYGYDGYFINQETTGDLV
KPLGEKMRQFMLYSKEYAAKVNHPIKYSWYDAMTYNYGRYHQDGLGEYNYQFMQPEG
DKVPADNFFANFNWDKAKNDYTIATANWIGRNPYDVFAGLELQQGGSYKTKVKWNDIL
DENGKLRLSLGLFAPDTITSLGKTGEDYHKNEDIFFTGYQGDPTGQKPGDKDWYGIANLV
ADRTPAVGNTFTTSFNTGHGKKWFVDGKVSKDSEWNYRSVSGVLPTWRWWQTSTGEKL
RAEYDFTDAYNGGNSLKFSGDVAGKTDQDVRLYSTKLEVTEKTKLRVAHKGGKGSKVY
MAFSTTPDYKFDDADAWKELTLSDNWTNEEFDLSSLAGKTIYAVKLFFEHEGAVKDYQFN
LGQLTISDNHQEPQSPTSFSVVKQSLKNAQEAEAVVQFKGNKDADFYEVYEKDGDSWKL
LTGSSSTTIYLPKVSRSASAQGTTQELKVVAVGKNGVRSEAATTTFDWGMTVKDTSLPKP
LAENIVP
>non-antioxidant_1198
MGSSHHHHHHMDRYEIKGVDVASYQGDIDWRELEKQNMKFAFIKATEGSAFVDKYFSKN
WTNANKTSMRVGAYHFFSFDSKGETQAEQFIRNVPKYKQALPPVIDVEFYANKKDNPPK
REDVTKELSVMIEMLEKHYGKKVILYATQEAYDLYIKDAYPQCDIWIRSVLTKPSLSDERK
WTFWQYTNRGKLSGYNGKEKYIDLNVFYGNEEEFENYGMKD
>non-antioxidant_1199
GTMGKSKGTRFERDLLVELWKAGFAAIRVAGAGVSPFPCPDIVAGNGRTYLAIEVKMRKE
LPLYLSADEVEQLVTFARGFGAEAYVALKLPRAAWRFFPVQMLERTEKNFKIDESVYPLGL
EIAEVAGKFFQERFGEKV
>non-antioxidant_1200
GAMGIKVQRPRCFFDIAINNQPAGRVVFELFSDVCPKTCENFRCLCTGEKGTGKSTQKPLH
YKSCLFHRVVKDFMVQGGDFSEGNGRGGESIYGGFFEDESFAVKHNKEFLLSMANRGKD
TNGSQFFITTKPTPHLDGHHVVFGQVISGQEVVREIENQKTDAASKPFAEVRILSCGEL
>non-antioxidant_1201
MGSSHHHHHHSSGLVPRGSHNAVNIVMRQPTTPNFSSALNITSANEGGSAMQIRGVEKAL
GTLKITHENPSVDKEYDKNAAALSIDIVKKQKGGKGTAAQGIYINSTSGTTGKLLRIRNLN
DDKFYVKPDGGFYAKETSQIDGNLKLKDPIANDHAATKAYVDGEVEKLKALLAAKQM
>non-antioxidant_1202
MGSKNESTASKASGTASEKKKIEYLDKTYEVTVPTDKIAITGSVESMEDAKLLDVHPQGAI
SFSGKFPDMFKDITDKAEPTGEKMEPNIEKILEMKPDVILASTKFPEKTLQKISTAGTTIPVS
HISSNWKENMMLLAQLTGKEKKAKKIIADYEQDLKETKTKINDKAKDSKALVIRIRQGNI
YIYPEQVYFNSTLYGDLGLKAPNEVKAAKAQELISLEKLSEMNPDHIFVQFSDDENADKP
DALKDLEKNPIWKSLKAVKEDHVYVNSVDPLAQGGTAWSKVRFLKAAAEKLTQNKLAA
ALEHHHHHH
>non-antioxidant_1203
GAMAQVPTDPGYFSVLLDVKHFSPEEISVKVVGDHVEVHARHEERPDEHGFIAREFHRRY

RLPPGVDPAAVTSALSPEGVLSIQATPASAQASLPSPPAAK
>non-antioxidant_1204
MGSSHHHHHHSSGLVPRGSHMAPIVETAKETNSDSSLYLDLMIKVLAGTVYEDPAHRENF
SHRDSTYREEVRNEGRDWPANAHTMIGIKRLENIRQCVEDVIGNNVPGDLVETGVWRGG
ACILMRGILRAHDVRDRTVWVADSFQGIPDVGEDGYAGDRKMALHRRNSVLAVSEEEVR
RNFRNYDLLDEQVRFLPGWFKDTLPTAPIDTLAVLRMDGDLYESTWDTLTNLYPKVSVGG
YVIVDDYMMCPPCKDAVDEYRAKFDIADELITIDRDGVYWQRTR
>non-antioxidant_1205
PVQHVGGTYTHIPLSPRTLNAWVKLVEEKKFGAEVVPGFQALSEGCTPYDINQMLNCVG
DHQAAMQIIREIINEEAAEWDVQHPIPGPLPAGQLREPRGSDIAGTTSTVEEQIQWMFRPQ
NPVPVGNIYRRWIQIGLQKCVRMY
>non-antioxidant_1206
MGSSHHHHHHSSGLVPRGSHMAADNRVQMRTTINNESPLLLSPLYGNDNGNGLWWGNT
LKGAWEAIPEDVKPYAAIELHPAKVCKPTSCIPRDTKELREWYVKMLEEAQSLNIPVFLVI
MSAGERNTVPPEWLDEQFQKYSVLKGVLNIENYWIYNNQLAPHSAKYLEVCAKYGAHFI
WHDHEKWFWETIMNDPTFFEASQKYHKNLVLATKNTPIRDDAGTDSIVSGFWLSGLCDN
WGSSTDTWKWWEKHYTNTFETGRARDMRSYASEPESMIAMEMMNVYTGGGTVYNFEC
AAYTFMTNDVPTPAFTKGIIPFFRHAIQNPAPSKEEVVNRTKAVFWNGEGRISSLNGFYQGL
YSNDETMPLYNNGRYHILPVIHEKIDKEKISSIFPNAKILTKNSEELSSKVNYLNSLYPKLYE
GDGYAQRVGNSWYIYNSNANINKNQQVMLPMYTNNTKSLSLDLTPHTYAVVKENPNNLH
ILLNNYRTDKTAMWALSGNFDASKSWKKEELELANWISKNYSINPVDNDFRTTTLTLSGH
TGHKPQINISGDKNHYTYTENWDENTHVYTITVNHNGMVEMSI
>non-antioxidant_1207
ELSENFKKLMKYPYRPCTCTRCIEEQRVSAWFDERFNRSMQPLLTAKNAHLEEDTYKWW
LRLQREKQPNNLNDTIRELFQVVPGNVDPLLEKRLVSCRRCAVVGNSGNLKESYYGPQID
SHDFVLRMNKAPTEGFEADVGSKTTHHFVYPESFRELAQEVSMILVPFKTTDLEWVISATT
TGRISHTYVPVPAKIKVKKEKILIYHPAFIKYVFDRWLQGHGRYPSTGILSVIFSLHICDEVD
LYGFGADSKGNWHHYWENNPSAGAFRKTGVHDGDFESNVTTILASINKIRIFKGR
>non-antioxidant_1208
SCATGPRNCKDLLDRGYFLSGWHTIYLPDCRPLTVLCDMDTDGGGWTVFQRRMDGSVD
FYRDWAAYKQGFGSQLGEFWLGNDNIHALTAQGSSELRVDLVDFEGNHQFAKYKSFKVA
DEAEKYKLVLGAFVGGSAGNSLTGHNNNFFSTKDQDNDVSSSNCAEKFQGAWWYADCH
ASNLNGLYLMGPHESFANGINWSAAKGYKYSYKVSEMKVRPA
>non-antioxidant_1209
MDPSQTPDANASISVSYKCGVKDGTKNTIRATINIKNTGTTPVNLSDIKVRYWFTSDGNEQ
NNFVCDYAAFGTDKVKGIVKKIENSVPGADTYCEISFTEDAGRLAPGGSTGTIPFRIEGAA
EYDQTDDYSYNSEMSDDFGDNTKITAYIKDKLKYGVEAAALEHHHHHH
>non-antioxidant_1210
RMKQLEDKIEENTSKIYHNTNEIARNTKLVGER
>non-antioxidant_1211
MPTFLLVNDDGYFSPGINALREALKSLGRVVVVAPDRNLSGVGHSLTFTEPLKMRKIDTDF
YTVIDGTPADCVHLGYRVILEEKKPDLVLSGINEGPNLGEDITYSGTVSGAMEGRILGIPSIA
FSAFGRENIMFEEIAKVCVDIVKKVLNEGIPEDTYLNVNIPNLRYEEIKGIKVTRQGKRAYK
ERVFKYIDPYGKPFYWIAAEEFGWHAEEGTDYWAVLNGYVSVTPLHLDLTNYKVMKSIK

YLEDSP
>non-antioxidant_1212
APLVHHHHHHALANPAICRYPLGMSGGQIPDEDITASSQWSESTAAKYGRLDSEEGDGAW
CPEIPVEPDDLKEFLQIDLHTLHFITLVGTQGRHAGGHGIEFAPMYKINYSRDGTRWISWR
NRHGKQVLDGNSNPYDIFLKDLEPPIVARFVRFIPVTDHSMNVCMRVELYGCVWLDG
>non-antioxidant_1213
MSRNPSNSDAAHAFWSTQPVPQTEDETEKIVFAGPMDEPKTVADIPEEPYPIASTFEWWTP
NMEAADDIHAIYELLRDNYVEDDDSMFRFNYSEEFLQWALCPPSYIPDWHVAVRRKADK
KLLAFIAGVPVTLRMGTPKYMKVKAQEKGQEEEAAKYDAPRHICEINFLCVHKQLREKR
LAPILIKEVTRRVNRTNVWQAVYTAGVLLPTPYASGQYFHRSLNPEKLVEIRFSGIPAQYQK
FQNPMAMLKRNYQLPNAPKNSGLREMKPSDVPQVRRILMNYLDNFDVGPVFSDAEISHY
LLPRDGVVFTYVVENDKKVTDFFSFYRIPSTVIGNSNYNILNAAYVHYYAATSMPLHQLIL
DLLIVAHSRGFDVCNMVEILDNRSFVEQLKFGAGDGHLRYYFYNWAYPKIKPSQVALVML
>non-antioxidant_1214
MNPEYDYLFKLLLIGDSGVGKSCLLLRFADDTYTESYISTIGVDFKIRTIELDGKTIKLQIW
DTAGQERFRTITSSYYRGAHGIIVVYDVTDQESFNNVKQWLQEIDRYASENVNKLLVGIKC
DLTTKKVVDYTTAKEFADSLGIPFLETSAKNATNVEQSFMTMAAEIKKRMGP
>non-antioxidant_1215
AMAEAYQIQSNGDPQSKPLLELYVKASGIDARRIGADLFCQEFWMELYALYEIGVARVEV
KTVNVNSEAFKKNFLGAQPPIMIEEEKELTYTDNREIEGRIFHLAKEFNVPLFEKDPSAEKR
IENLYRNFKLFLRAKVEFDKGKKEPSRVEDLPAQIKVHYNRVCEQLSNIDQLLSERKSRYL
LGNSMTEYDCELMPRLHHIRIIGLSLLGFDIPHNFTHLWAYILTAYRTAAFIESCPADQDIIHH
YKEQMNLFTNQRETLQSPTKTHTIPEKVLSDIRVKGLAPDVNVH
>non-antioxidant_1216
MRTSKKEMILRTAIDYIGEYSLETLSYDSLAEATGLSKSGLIYHFPSRHALLLGMHELLADD
WDKELRDITRDPEDPLERLRAVVVTLAENVSRPELLLLIDAPSHPDFLNAWRTVNHQWIP
DTDDLENDAHKRAVYLVQLAADGLFVHDYIHDDVLSKSKRQAMLETILELIPSQTELHHH
HHH
>non-antioxidant_1217
MGIMPRKVLAIKNFKERFDLLPKLKGVIAEKQPDILVVVGNILKNEALEKEYERAHLARR
EPNRKVIHENEHYIIETLDKFFREIGELGVKTFVVPGKNDAPLKIFLRAAYEAETAYPNIRVL
HEGFAGWRGEFEVIGFGGLLTEHEFEEDFVLKYPRWYVEYILKFVNELKPRRLVTIFYTPPI
GEFVDRTPEDPKHHGSAVVNTIIKSLNPEVAIVGHVGKGHELVGNTIVVNPGEFEEGRYAF
LDLTQHKIKLEQFS
>non-antioxidant_1218
SLGLAQSAQEILDRVEKNLSTPWQATVQGRIQGPGGEEELLARVYALPQARLFRVEFLKPG
SLEGNFTVITEKEVWNYLYLTNQLVISPREKAKIQGLGFAPQGLGDLKALSEQVDLRLEGE
VRLPEGMAWKLVGRSKENQGFAAMELYILKADPRPLRFVFLDEKGKVLADLKVVEFKRT
NLTEAQLKRYPKDAQVVRR
>non-antioxidant_1219
MRLRVDVIPGEHLAYPDVVLVVDVIRATTTAAAFLEAGAEALYWTPSLESALAFKDEDVV
LAGETGGLKPPRFDLGNSPREALSAQVAGRVVVMSTTNGTKAAHAAARTAKHVLLASLY
NAHAAARLARELATEEVAILCAGKEGRAGLDDLYTAGVLAEYLGFLGEVEPEDGARVALA
VKRAYPDPLEALSLSAAALALKQVGLEADVPFCAQVAKSAAVPVLRGRVGEALIFKRA

>non-antioxidant_1220
TAPSQVLKIRRPDDWHLHLRDGDMLKTVVPYTSEIYGRAIVMPNLAPPVTTVEAAVAYRQ
RILDAVPAGHDFTPLMTCYLTDSLDPNELERGFNEGVFTAAKLYPANATANSSHGVTSVDA
IMPVLERMEKIGMPLLVHGEVTHADIDIFDREARFIESVMEPLRQRLTALKVVFEHITTKDA
ADYVRDGNERLAATITPQHLMFNRNHMLVGGVRPHLYCLPILKRNIHQQALRELVASGFN
RVFLGTDSAPHARHRKESSCGCAGCFNAPTALGSYATVFEEMNALQHFEAFCSVNGPQFY
GLPVNDTFIELVREEQQVAESIALTDDTLVPFLAGETVRWSVKQ
>non-antioxidant_1221
MVPLTEENVESVLDEIRPYLMSDGGNVALHEIDGNVVRVKLQGACGSCPSSTMTMKMGI
ERRLMEKIPEIVAVEALPDEETGLELNEENIEKVLEEIRPYLIGTADGSLDLVEIEDPIVKIRIT
GPAAGVMTVRVAVTQKLREKIPSIAAVQLI
>non-antioxidant_1222
GLGLPAGLYAFNSGGISLDLGINDPVPFNTVGSKFGTAISQLDADTFVISETGFYKITVIANT
ATASVLGGLTIQVNGVPVPGTGSSLISLGAPIVIQAITQITTTPSLVEVIVTGLGLSLALGTSA
SIIIEKVAL
>non-antioxidant_1223
GPLGSMADEATRRVVSEIPVLKTNAGPRDRELWVQRLKEEYQSLIRYVENNKNADNDWF
RLESNKEGTRWFGKCWYIHDLLKYEFDIEFDIPITYPTTAPEIAVPELDGKTAKMYRGGKIC
LTDHFKPLWARNVPKFGLAHLMALGLGPWLAVEIPDLIQKGVIQHKEKCNQ
>non-antioxidant_1224
MVLYFIGLGLYDERDITVKGLEIAKKCDYVFAEFYTSLMAGTTLGRIQRLIGKEIRVLSRED
VELNFENIVLPLAKENDVAFLTPGDPLVATTHAELRIRAKRAGVESYVIHAPSIYSAVGITGL
HIYKFGKSATVAYPEGNWFPTSYYDVIKENAERGLHTLLFLDIKAEKRMYMTANEAMELL
LKVEDMKKGGVFTDDTLVVVLARAGSLNPTIRAGYVKDLIREDFGDPPHILIVPGKLHIVE
AEYLVEIAGAPREILRVNV
>non-antioxidant_1225
MGNTATEFDGPYVITPISGQSTAYWICDNRLKTTSIEKLQVNRPEHCGDLPETKLSSEIKQI
MPDTYLGIKKVVALSDVHGQYDVLLTLLKKQKIIDSDGNWAFGEGHMVMTGDIFDRGHQ
VNEVLWFMYQLDQQARDAGGMVHLLMGNHEQMVLGGDLRYVHQRYDIATTLINRPYN
KLYSADTEIGQWLRSKNTIIKINDVLYMHGGISSEWISRELTLDKANALYRANVDASKKSL
KADDLLNFLFFGNGPTWYRGYFSETFTEAELDTILQHFNVNHIVVGHTSQERVLGLFHNK
VIAVDSSIKVGKSGELLLLLENNRLIRGLYDGTRETLQENSLNQ
>non-antioxidant_1226
AQVINTFDGVADYLQTYHKLPDNYITKSEAQALGWVASKGNLADVAPGKSIGGDIFSNRE
GKLPGKSGRTWREADINYTSGFRNSDRILYSSDWLIYATTDHYQTFTKIR
>non-antioxidant_1227
MKWFNTLSHNRWLEQETDRIFNFGKNAVVPTGFGWLGNKGQIKEEMGTHLWITARMLH
VYSVAASMGRPGAYDLVDHGIKAMNGALRDKKYGGWYACVNDQGVVDASKQGYQHFF
ALLGAASAVTTGHPEARKLLDYTIEVIEKYFWSEEEQMCLESWDEAFSQTEDYRGGNAN
MHAVEAFLIVYDVTHDKKWLDRALRIASVIIHDVARNGDYRVNEHFDSQWNPIRDYNKD
NPAHRFRAYGGTPGAWIEWGRLMLHLHAALEARFETPPAWLLEDAKGLFHATIRDAWAP
DGADGFVYSVDWDGKPIVRERVRWPIVEAMGTAYALYTLTDDSQYEEWYQKWWDYCIK
YLMDYENGSWWQELDADNKVTTKVWDGKQDIYHLLHCLVIPRLPLAPGLAPAVAAGLL
DINAKLEHHHHHH

>non-antioxidant_1228
MHHHHHHMKDKIIDNAITLFSEKGYDGTTLDDISKSVNIKKASLYYHYDNKEEIYRKSVE
NCFNYFIDFMMRNHDDNYSIDGLYQFLFKFIFDVDERYIKLYVQLSSAPEALNSEIKHHLQ
EINTTLHDELIKYYDPTHIALDKEDFINMILMFLETWYFRASFSQKFGIIEDSKNRFKDQVY
SLLNVFLKK
>non-antioxidant_1229
AAAAAAMTMMDMNFKYCHKIMKKHSKSFSYAFDLLPEDQRKAVWAIYAVCRKIDDSIDV
YGDIQFLNQIKEDIQSIEKYPYEYHHFQSDRRIMMALQHVAQHKNIAFQSFYNLIDTVYKD
QHFTMFETDAELFGYCYGVAGTVGEVLTPILSDHETHQTYDVARRLGESLQLINILRDVGE
DFENERIYFSKQRLKQYEVDIAEVYQNGVNNHYIDLWEYYAAIAEKDFRDVMDQIKVFSI
EAQPIIELAARIYIEILDEVRQANYTLHERVFVEKRKKAKLFHEINSKYHRI
>non-antioxidant_1230
MTQVRETVSFKAGDVILYPGVPGPRDRAYRVLEGLVRLEAVDEEGNALTLRLVRPGGFFG
EEALFGQERIYFAEAATDVRLEPLPENPDPELLKDLAQHLSQGLAEAYRRIERLATQRLKN
RMAAALLELSETPLAHEEEGKVVLKATHDELAAAVGSVRETVTKVIGELAREGYIRSGYG
KIQLLDLKGLKELAESRGQGR
>non-antioxidant_1231
AHMFMAENRLQLQKGSAEETIERFYNRQGIETIEGFQQMFVTKTLNTEDTDEVKILTIWES
EDSFNNWLNSDVFKEAHKNVRLKSDDDGQQSPILSNKVFKYDIGYHYQK
>non-antioxidant_1232
GAHMVNMVSNPGFEDGLDSWQDWQQDMSAVPEAAHNGALGLKIGGGKAAGGGQDIPL
KPNTTYILGAWAKFDSKPAGTFDVVVQYHLKDANNTYVQHILNFNETDWTYKQLLFTTP
DVFGSTPQLALWKGDTSKANLYVDDVYLVEV
>non-antioxidant_1233
MSQCVDGIKHLCTSVLGCFDLDLYKQSGGLGDPELLARDTVFSVSEIEALYELFKKISSAVI
DDGLINKEEFQLALFKTNKKESLFADRVFDLFDTKHNGILGFEEFARALSVFHPNAPIDDKI
HFSFQLYDLKQQGFIERQEVKQMVVATLAESGMNLKDTVIEDIIDKTFEEADTKHDGKIDK
EEWRSLVLRHPSLLKNMTLQYLKDITTTFPSFVFHSQVEDT
>non-antioxidant_1234
AEIYNKDGNKVDLYGKAVGLHYFSKGNGENSYGGNGDMTYARLGFKGETQINSDLTGY
GQWEYNFQGNNSEGADAQTGNKTRLAFAGLKYADVGSFDYGRNYGVVYDALGYTDML
PEFGGDTAYSDDFFVGRVGGVATYRNSNFFGLVDGLNFAVQYLGKNERDTARRSNGDGV
GGSISYEYEGFGIVGAYGAADRTNLQEAQPLGNGKKAEQWATGLKYDANNIYLAANYGE
TRNATPITNKFTNTSGFANKTQDVLLVAQYQFDFGLRPSIAYTKSKAKDVEGIGDVDLVNY
FEVGATYYFNKNMSTYVDYIINQIDSDNKLGVGSDDTVAVGIVYQF
>non-antioxidant_1235
GIPMETKGGTVKAASGFNATEDAQVLRKAMKGLGTDEDAIIGVLACRNTAQRQEIRTAYK
STIGRDLLEDLKSELSSNFEQVILGMMTPTVLYDVQELRRAMKGAGTDEGCLIEILASRNP
EEIRRINQTYQQQYGRSLEEDICSDTSFMFQRVLVSLTAGGRDEGNYLDDALVKQDAQDL
YEAGEKRWGTDEVKFLSILCSRNRNHLLHVFDEYKRISQKDIEQSIKSETSGSFEDALLAIV
KCMRNKPAYFAERLYKSMKGLGTDDSTLIRVMVSRAEIDMLDIRANFKRLYGKSLYSFIKG
DTSGDYRKVLLILCGGDD
>non-antioxidant_1236
MAFSGSQAPYLSPAVPFSGTIQGGLQDGLQITVNGTVLSSSGTRFAVNFQTGFSGNDIAFHF

NPRFEDGGYVVCNTRQNGSWGPEERKTHMPFQKGMPFDLCFLVQSSDFKVMVNGILFVQ
YFHRVPFHRVDTISVNGSVQLSYISFQ
>non-antioxidant_1237
LASVIPDVATLNSLFNQIKNESCGTSTASSPCITFRYPVDGCYARAHKMRQILMNNGYDCE
KQFVYGNLKASTGTCCVAWSYHVAILVSYKNASGVTEKRIIDPSLFSSGPVTDTAWRNAC
VNTSCGSASVSSYANTAGNVYYRSPSNSYLYDNNLINTNCVLTKFSLLSGCSPSPAPDVSSC
GF
>non-antioxidant_1238
EQKTRPFTLPNLPLSSLSNSRAPLPISSMGISPDNVQSVQFQNGRCTLDGRLVGTTPVSLSH
VAKIRGTSNGTVINLTELDGTPFHPFEGPAPIGFPDLGGCDWHINMTQFGHSSQTQYDVDT
TPDTFVPHLGSIQANGIGSGNYVGVLSWISPPSHPSGSQVDLWKIPNYGSSITEATHLAPSV
YPPGFGEVLVFFMSKMPGPGAYNLPCLLPQEYISHLASEQAPTVGEAALLHYVDPDTGRN
LGEFKAYPDGFLTCVPNGASSGPQQLPINGVFVFVSWVSRFYQLKPVGTA
>non-antioxidant_1239
GPGHMEGLRCVVLPEDLCHKFLQLAESNTVRGIETCGILCGKLTHNEFTITHVIVPKQSAG
PDYCDMENVEELFNVQDQHDLLTLGWIHTHPTQTAFLSSVDLHTHCSYQLMLPEAIAIVC
SPKHKDTGIFRLTNAGMLEVSACKKKGFHPHTKEPRLFSICKHVLVKDIKIIVLDLR
>non-antioxidant_1240
GSSEGYCSRILRAQGTRREGYTEFSLRVEGDPDFYKPGTSYRVTLSAAPPSYFRGFTLIALR
ENREGDKEEDHAGTFQIIDEEETQFMSNCPVAVTESTPRRRTRIQVFWIAPPAGTGCVILKA
SIVQKRIIYFQDEGSLTKKLCEQDS
>non-antioxidant_1241
GIPIEPVLENVQPNSAASKAGLQAGDRIVKVDGQPLTQWVTFVMLVRDNPGKSLALEIER
QGSPLSLTLIPESKPGNGKAIGFVGIEPKVI
>non-antioxidant_1242
MDNKLDVFRRELVDVEGIPLFWSIAEHWSQVESFEARPDDILISTYPKSGTTWVSEILDLIY
NNGDAEKCKRDAIYKRVPFMELIIPGITNGVEMLNNMPSPRIVKTHLPVQLLPSSFWKNDC
KIIYVARNAKDVVVSYYYFYQMAKIHPEPGTWEEFLEKFMAGQVSFGPWYDHVKSWWE
KRKEYRILYLFYEDMKENPKCEIQKILKFLEKDIPEEILNKILYHSSFSVMKENPSANYTTM
MKEEMDHSVSPFMRKGISGDWKNQFTVAQYEKFEEDYVKKMEDSTLKFRSEI
>non-antioxidant_1243
MGSSHHHHHHSSGLVPRGSHMQQKLTSPDNNLVMTFQVDSKGAPTYELTYKNKVVIKPS
TLGLELKKEDNTRTDFDWVDRRDLTKLDSKTNLYDGFEVKDTQTATFDETWQPVWGEEK
EIRNHYNELAVTLYQPMNDRSIVIRFRLFNDGLGFRYEFPQQKSLNYFVIKEEHSQFGMNG
DHIAFWIPGDYDTQEYDYTISRLSEIRGLMKEAITPNSSQTPFSQTGVQTALMMKTDDGLY
INLHEAALVDYSCMHLNLDDKNMVFESWLTPDAKGDKGYMQTPCNTPWRTIIVSDDARN
ILASRITLNLNEPCKIADAASWVKPVKYIGVWWDMITGKGSWAYTDELTSVKLGETDYSK
TKPNGKHSANTANVKRYIDFAAAHGFDAVLVEGWNEGWEDWFGNSKDYVFDFVTPYPD
FDVKEIHRYAARKGIKMMMHHETSASVRNYERHMDKAYQFMADNGYNSVKSGYVGNII
PRGEHHYGQWMNNHYLYAVKKAADYKIMVNAHEATRPTGICRTYPNLIGNESARGTEYE
SFGGNKVYHTTILPFTRLVGGPMDYTPGIFETHCNKMNPANNSQVRSTIARQLALYVTMY
SPLQMAADIPENYERFMDAFQFIKDVALDWDETNYLEAEPGEYITIARKAKDTDDWYVG
CTAGENGHTSKLVFDFLTPGKQYIATVYADAKDADWKENPQAYTIKKGILTNKSKLNLHA
ANGGGYAISIKEVKDKSEAKGLKRL

>non-antioxidant_1244
AARQMEALNRGLVAVKTDGGIFVSWRFLGTENASVLFNVYRDGQKLNAAPVKTTNYVD
KNGSAGSTYTVRAVVNGTEQPASEKASVWAQPYHSVPLDKPAGGTTPKGESYTYSANDA
SVGDVDGDGQYELILKWDPSNSKDNSQDGYTGDVLIDAYKLDGTKLWRINLGKNIRAGA
HYTQFMVYDLDGDGKAEVAMKTADGTKDGTGKVIGNANADYRNEQGRVLSGPEYLTVF
QGSTGKELVTANFEPARGNVSDWGDSYGNRVDRFLAGIAYLDGQRPSLIMTRGYYAKTM
LVAYNFRDGKLSKLWTLDSSKSGNEAFAGQGNHNLSIADVDGDGKDEIIFGSMAVDHDGK
GMYSTGLGHGDALHTGDLDPGRPGLEVFQVHEDKNAKYGLSFRDAATGKILWGVYAGK
DVGRGMAADIDPRYPGQEVWANGSLYSAKGVKIGSGVPSSTNFGIWWDGDLLREQLDSN
RIDKWDYQNGVSKNMLTASGAAANNGTKATPTLQADLLGDWREEVVWRTEDSSALRIY
TTTIPTEHRLYTLMHDPVYRLGIAWQNIAYNQPPHTSFFLGDGMAEQPKPNMYTPLEHHH
HHH
>non-antioxidant_1245
MSKMLYRVELIITNKEGVRDPEGETIQRYVVSRFSDKIIETRAGKYLVFRVNSSSQQEATEL
VKKLADEMRLYNPIVHKIEIRANRIEDSSN
>non-antioxidant_1246
DDLPYRFGLGKADITGEAAEVGMMGYSSLEQKTAGIHMRQWARAFVIEEAASGRRLVYV
NTDLGMIFQAVHLKVLARLKAKYPGVYDENNVMLAATHTHSGPGGFSHYAMYNLSVLG
FQEKTFNAIVDGIVRSIERAQARLQPGRLFYGSGELRNASRNRSLLSHLKNPDIAGYEDGI
DPQMSVLSFVDANGELAGAISWFPVHSTSMTNANHLISPDNKGYASYHWEHDVSRKSGF
VAAFAQTNAGNLSPNLNLKPGSGPFDNEFDNTREIGLRQFAKAYEIAGQAQEEVLGELDSR
FRFVDFTRLPIRPEFTDGQPRQLCTAAIGTSLAAGSTEDGPGPLGLEEGNNPFLSALGGLLT
GVPPQELVQCQAEKTILADTGNKKPYPWTPTVLPIQMFRIGQLELLGAPAEFTVMAGVRIR
RAVQAASEAAGIRHVVFNGYANAYASYVTTREEYAAQEYEGGSTLYGPWTQAAYQQLFV
DMAVALRERLPVETSAIAPDLSCCQMNFQTGVVADDPYIGKSFGDVLQQPRESYRIGDKV
TVAFVTGHPKNDLRTEKTFLEVVNIGKDGKQTPVTVATDNDWDTQYRWERVGISASKATI
SWSIPPGTEPGHYYIRHYGNAKNFWTQKISEIGGSTRSFEVLGTTP
>non-antioxidant_1247
ADGKAIFQQKGCGSCHQANVDTVGPSLKKIAQAYAGKEDQLIKFLKGEAPAIVDPAKEAI
MKPQLTMLKGLSDAELKALADFILSHK
>non-antioxidant_1248
MKRVSRRAFLRRLGVGVAATAAFSPLAVAQARRYRWRIQTAWDAGTVGYSLFQKFTERV
KELTDGQLEVQPFPAGAVVGTFDMFDAVKTGVLDGMNPFTLYWAGRMPVTAFLSSYALG
LDRPDQWETWFYSLGGLDIARRAFAEQGLFYVGPVQHDLNIIHSKKPIRRFEDFKGVKLR
VPGGMIAEVFAAAGASTVLLPGGEVYPALERGVIDAADFVGPAVNYNLGFHQVAKYIIMG
PPETPAIHQPVDLMDFTINLNRWRSLPKPLQERFIAAVHEYSWIHYAGIQKANLEAWPKYR
QAGVEVIRLSNEDVRKFRRLAIPIWFKWAKMDKYSREAFASQLEYMKGIGYVTDEELKG
LSL
>non-antioxidant_1249
SAQFASVTIRNAQTGRLLDSNYNGNVYTLPANGGNYQRWTGPGDGTVRNAQTGRCLDS
NYDGAVYTLPCNGGSYQKWLFYSNGYIQNVETGRVLDSNYNGNVYTLPANGGNYQKW
YTG
>non-antioxidant_1250
MEFTEFNLNELIREVYVLFEEKIRKMNIDFCFETDNEDLRVEADRTRIKQVLINLVQNAIEA

TGENGKIKITSEDMYTKVRVSVWNSGPPIPEELKEKIFSPFFTTKTQGTGLGLSICRKIIEDE
HGGKIWTENRENGVVFIFEIPKTPEKR
>non-antioxidant_1251
MFKGIVQGAGIIKKISKNDDTQRHGITFPKDILESVEKGTVMLVNGCSLTVVRISGDVVYF
DIDQAINTTTFRELEVGNKVNLEVRPEFGSLLGKGALTGNIKGVATVDNITEEEDRLKVYIK
IPKDLIENILSEDHIGINGVSHSIEEISDDIIFINYPKNLSITTNLGTLEKGSDVNVETLNVSNE
WD
>non-antioxidant_1252
AAPANAVTADDPTAIALKYNQDATKSERVAAARPGLPPEEQHCANCQFMQANVGEGDWK
GCQLFPGKLINVNGWCASWTLKAG
>non-antioxidant_1253
MQIFVKTLTGKTITLEVEPSDTIENVKAKIQDKEGIPPDQQRLIFAGKQLEDGRTLSDYNIQ
RESTLHLVLRLRGG
>non-antioxidant_1254
MGSSHHHHHHSSGENLYFQGHMTQQPQAKYRHDYRAPDYQITDIDLTFDLDAQKTVVTA
VSQAVRHGASDAPLRLNGEDLKLVSVHINDEPWTAWKEEEGALVISNLPERFTLKIINEISP
AANTALEGLYQSGDALCTQCEAEGFRHITYYLDRPDVLARFTTKIIADKIKYPFLLSNGNR
VAQGELENGRHWVQWQDPFPKPCYLFALVAGDFDVLRDTFTTRSGREVALELYVDRGNL
DRAPWAMTSLKNSMKWDEERFGLEYDLDIYMIVAVDFFNMGAMENKGLNIFNSKYVLA
RTDTATDKDYLDIERVIGHEYFHNWTGNRVTCRDWFQLSLKEGLTVFRDQEFSSDLGSRA
VNRINNVRTMRGLQFAEDASPMAHPIRPDMVIEMNNFYTLTVYEKGAEVIRMIHTLLGEE
NFQKGMQLYFERHDGSAATCDDFVQAMEDASNVDLSHFRRWYSQSGTPIVTVKDDYNP
ETEQYTLTISQRTPATPDQAEKQPLHIPFAIELYDNEGKVIPLQKGGHPVNSVLNVTQAEQT
FVFDNVYFQPVPALLCEFSAPVKLEYKWSDQQLTFLMRHARNDFSRWDAAQSLLATYIKL
NVARHQQGQPLSLPVHVADAFRAVLLDEKIDPALAAEILTLPSVNEMAELFDIIDPIAIAEVR
EALTRTLATELADELLAIYNANYQSEYRVEHEDIAKRTLRNACLRFLAFGETHLADVLVSK
QFHEANNMTDALAALSAAVAAQLPCRDALMQEYDDKWHQNGLVMDKWFILQATSPAA
NVLETVRGLLQHRSFTMSNPNRIRSLIGAFAGSNPAAFHAEDGSGYLFLVEMLTDLNSRNP
QVASRLIEPLIRLKRYDAKRQEKMRAALEQLKGLENLSGDLYEKITKALA
>non-antioxidant_1255
SNAMTEKKIDFKKEEKKFYAPKRKPERIFVPEMNFLMVDGKGDPDGEEYQKAVQSLYAIA
YTIKMSKMGETRLDGYSDFVVPPLEGFWWSEGKFDLKDRDAWLWTSILRQPDFVTEEVL
EWAKEVARKKKPDVDTSRVKLVRFEEGECVQMMHVGPFSEEVHTVAEMHQFMETEGLR
NDTGAIRKHHEIYLSDPRKANPEKMKTILRLPVS
>non-antioxidant_1256
MFKYVIPLCALTLAAPSFAAQTTLMLSQKSDVNYLGWSTDESKVARQEVYRGTTSNPDLR
ERIAVLDAETRTFKDADTNSGLNYWYWVDVVSENQAQVVSNAVTTAPNAGPLRAAKASS
ECKPGATFENRTVDCGGVTIGTSCPNDSDKQKPLIILKNATVKNLRISASGGADGIHCDSG
NCTIENVIWEDICEDAATNNGKTMTIVGGIAHNAKDGYGGKPDKVLQHNSKNSTTVVKG
NFTLTGEHGKLWRSCGDCSNNGGPRFLTVTSATVNGTIDSIAGVNRNYGDVATISGLKIKN
YKEGKPPVCEEFKGVVKGQGSTEKYGEKWDTTNCKVSRSGVSKL
>non-antioxidant_1257
SNALNSAPTPRDVVANAPAPVQAAVAGAQEYAAQAGLNTEELAVDALYNAIKVRLAGTG
LGIPPQIEAFYQANRTNFNGFYMANRGAIDFIFSM

>non-antioxidant_1258
MTQKFGLSAALTTPFKTDGTVDIDAMIAHARRCLSNGCDSVTLFGTTGEGCSVGSRERQA
ILSSFIAAGIAPSRIVTGVLVDSIEDAADQSAEALNAGARNILLAPPSYFKNVSDDGLFAWFS
AVFSKIGKDARDILVYNIPSVTMVTLSVELVGRLKAAFPGIVTGVKDSSGNWSHTERLLKE
HGDLAILIGDERDLARGVRLGGQGAISGVANFLTQEVRAMAVDGKDDPRIVDLVVELLKF
PVTPAVKVLVSHTTGETIWSDVRAPLVAISPEDRRQIEGAFDALFRRQAA
>non-antioxidant_1259
ADYDLKFGMNAGTSSNEYKAAEMFAKEVKEKSQGKIEISLYPSSQLGDDRAMLKQLKDG
SLDFTFAESARFQLFYPEAAVFALPYVISNYNVAQKALFDTEFGKDLIKKMDKDLGVTLLS
QAYNGTRQTTSNRAINSIADMKGLKLRVPNAATNLAYAKYVGASPTPMAFSEVYLALQT
NAVDGQENPLAAVQAQKFYEVQKFLAMTNHILNDQLYLVSNETYKELPEDLQKVVKDAA
ENAAKYHTKLFVDGEKDLVTFFEKQGVKITHPDLVPFKESMKPYYAEFVKQTGQKGESA
LKQIEAINPKGEA
>non-antioxidant_1260
MSLILESLVTTLDEQGRINLAPLGPIVLPPQSPGGLPQFLLRPYEGSTTCDNLLASGNAVIHV
IDDALLIAKTAIGKVDASDLVVPIPGLEDTHVRLKRCHRWFAVRVTQRAGTPPRHELTARC
LASGLVDPFFGFNRAKHAVIEAAVAATRLHLLPPEEIEEELERARIAIEKTGGEPEREALQLI
RRHVRESSISEGHHHHHH
>non-antioxidant_1261
GMEFNHLTKQLNQLLAQDYVAFSITENPVVQMLSQASFAQIAYVMQQYSIFPKELVGFTEL
ARRKALGAGWNGVAQELQENIDEEMGSTTGGISHYTLLADGLEEGLGVAVKNTMPSVAT
SKLLRTVLSLFDRQVDYVLGATYAIEATSIPELTLIVKLVEWLHEGAIPKDLQYFFSKHLDE
WEIEHEAGLRTSVAAYIQPEEFGEFAAGFRAMIDAMQVWWQELAQEAISSEVVLSTAIAQ
HH
>non-antioxidant_1262
PVIQTFVSTPLDHHKRENLAQVYRAVTRDVLGKPEDLVMMTFHDSTPMHFFGSTDPVACV
RVEALGGYGPSEPEKVTSIVTAAITKECGIVADRIFVLYFSPLHCGWNGTNF
>non-antioxidant_1263
SNAMKDPLLNSLIYVSRYYGLANSPEALVNGLPLSDGKLTPFLLPRAAERAGLVAKENRAE
LEKISSLILPAILVLKGGDSCVLNSINMETREAEVTTLESGMVPISIPLEDLLEQYTGRYFLV
KKQFR
>non-antioxidant_1264
GMSDHVCQEGCRHHSHGEDSPEIQQEFQEGRRDFMRDFAVGGVLASAASLGISSSAFGQT
MPKTGLTSGHATHYYIPASDKTVSWGFFSKSLKPVVELESGDFATIETLTHHSNDDASLMV
KGDPGAESVFYWDSKRKNVDRRGMGPMDHKLGAGGGMGVHILTGPVAIKGAEPGDVLE
VRIVDVALRPSANPEFKGKTFGSNVAANWGFHYNELIEEPKKREVVTIYELDATGERNWA
RAFYNYRWTPQKDPFGVVHPIVDYPGVPVDHSTISKNYNVLKNIRVPVRPHFGTMGLAPK
EADLVNSVPPSHFGGNIDNWRIGKGATMYYPVSVAGGLFSVGDPHASQGDSEMCGTAIEC
SLTGTFQFILHKKADLPGTPLADLQYPLLETQDEWVLHGFSYANYLAELGPDAQNSIFSKS
SLDLALKDAFRKMRHFLMQTQNLTEDEAVSLMSIGVDFGITQVVDGNWGVHAVVKKGIF
PGRDV
>non-antioxidant_1265
SAVAPAEINEARLVAQYNYSINILAMLLVGFGFLMVFVRRYGFSATTGTYLVVATGLPLYIL
LRANGIFGHALTPHSVDAVIYAEFAVATGLIAMGAVLGRLRVFQYALLALFIVPVYLLNEWL

VLDNASGLTEGFQDSAGSIAIHAFGAYFGLGVSIALTTAAQRAQPIESDATSDRFSMLGSM
VLWLFWPSFATAIVPFEQMPQTIVNTLLALCGATLATYFLSALFHKGKASIVDMANAALAG
GVAIGSVCNIVGPVGAFVIGLLGGAISVVGFVFIQPMLESKAKTIDTCGVHNLHGLPGLLG
GFSAILIVPGIAVAQLTGIGITLALALIGGVIAGALIKLTGTTKQAYEDSHEFIHLAGPEDEHK
AERLVLEAKTEIQGLKNRIDAAVLSAKSEGHHHHHH
>non-antioxidant_1266
MGSSHHHHHHSSGLVPRGSHMEAIGVLMMCPMSTYLEQELDKRFKLFRYWTQPAQRDFL
ALQAESIRAVVGNSNAGADAELIDALPKLEIVSSFSVGLDKVDLIKCEEKGVRVTNTPDVL
TDDVADLAIGLILAVLRRICECDKYVRRGAWKFGDFKLTTKFSGKRVGIIGLGRIGLAVAER
AEAFDCPISYFSRSKKPNTNYTYYGSVVELASNSDILVVACPLTPETTHIINREVIDALGPKG
VLINIGRGPHVDEPELVSALVEGRLGGAGLDVFEREPEVPEKLFGLENVVLLPHVGSGTVE
TRKVMADLVVGNLEAHFSGKPLLTPVV
>non-antioxidant_1267
GMDISLLKQVVQSTNKIALSTAVNNEADVKIVNFVWYEAQPDTLYFSSVKTSPALKVYDQ
NPDIAFITIPNDGTAGNPYLRAQHVKLQRSTKTMTDLLPQYLETVPNYQQVWDAIGSTLV
VFELKLTDLFVDAGVGGEKQTLTFN
>non-antioxidant_1268
MGSVTPIPLPKIDEPEEYNTNYILFWNHVGLELNRVTHTVGGPLTGPPLSARALGMLHLAI
HDAYFSICPPTDFTTFLSPDTENAAYRLPSPNGANDARQAVAGAALKMLSSLYMKPVEQP
NPNPGANISDNAYAQLGLVLDRSVLEAPGGVDRESASFMFGEDVADVFFALLNDPRGASQ
EGYHPTPGRYKFDDEPTHPVVLIPVDPNNPNGPKMPFRQYHAPFYGKTTKRFATQSEHFL
ADPPGLRSNADETAEYDDAVRVAIAMGGAQALNSTKRSPWQTAQGLYWAYDGSNLIGTP
PRFYNQIVRRIAVTYKKEEDLANSEVNNADFARLFALVDVACTDAGIFSWKEKWEFEFWR
PLSGVRDDGRPDHGDPFWLTLGAPATNTNDIPFKPPFPAYPSGHATFGGAVFQMVRRYYN
GRVGTWKDDEPDNIAIDMMISEELNGVNRDLRQPYDPTAPIEDQPGIVRTRIVRHFDSAWE
LMFENAISRIFLGVHWRFDAAAARDILIPTTTKDVYAVDNNGATVFQNVEDIRYTTRGTRE
DREGLFPIGGVPLGIEIADEIFNNGLKPTPPEIQPMPQETPVQKPVGQQPVKGMWEEEQAP
VVKEAP
>non-antioxidant_1269
ANQLRELKQTHTYTVFGYTDGGFAVISADDLAPELLGVSESNFVETDNPSFKWWLKAIDE
VITNAVKSNKPLNVIKPDPSKYAAEVSTLLTTTWGQQMPYNKLLPKTKKGRLITGAVATAT
AQVLNYFKYPVRGIGSHTVHYPANDPSGVAISADFGNTTYDWANMKDNYSGNYTEAEA
NAVATLMLHCGVASEMQYGGPNEGSGAYMTDCAAGLRTYFGFTDAEYITRANYTDEQW
MDIVFSELTKGHPLIYGGVSPGSMGQDAGHAFVIDGYNKAGLVSVNWGWNGDVDGYYK
IDLLNPGNMYSFTAEQDMVRGVYGKP
>non-antioxidant_1270
ANLERTFIAIKPDGVQRGLVGEIIKRFEQKGFRLVAMKFLRASEEHLKQHYIDLKDRPFFPG
LVKYMNSGPVVAMVWEGLNVVKTGRVMLGETNPADSKPGTIRGDFCIQVGRNIIHGSDS
VKSAEKEISLWFKPEELVDYKSCAHDWVYE
>non-antioxidant_1271
GCSNISEDVNNPNRSLFLIESEPSTGASVSKNLTEIILIFSNDINKVSQLALTDLITDSDIQGID
YNIEGNKVIINNFSLEPTCNYRLSYEVIDIYDNHLQGYIEFLVNQSNYPQIPDQEVNHTILQA
FYWEMNTGEYATEHPEEANLWNLLAERAPELAEAGFTAVWLPPANKGMAGIHDVGYGT
YDLWDLGEFDQKGTVRTKYGTKGELENAIDALHNNDIKVYFDAVLNHRMGADYAETVL

LDENSRDKPGQYIKAWTGFNFPGRNGEYSNFTWNGQCFDGTDWDDYSKESGKYLFDEK
SWDWTYNWDEDYLMGADVDYENEAVQNDVIDWGQWIINNIDFDGFRLDAVKHIDYRFI
DKWMSAVQNSSNRDVFFVGEAWVEDVDDLKGFLDTVGNPDLRVFDFPLRSFFVDMLNG
AYMADLRNAGLVNSPGYENRAVTFVDNHDTDRDEGSYTVSIYSRKYQAYAYILTRAEGVP
TVYWKDYYIWEMKEGLDKLLTARRYYAYGPGYEVDNNDADIYSYVRSGFPDVAGDGLV
LMISDGTSGNVAGKWINSRQPDTEFYDLTGHIKEHVTTDSEGYGNFKVIKSEDKGWSIWV
PVE
>non-antioxidant_1272
GMPQHDKSRLVRIDTGPMINPVAGKPSRPIAGDASFRTVTAFEGGQGKVESGVWESTSGSF
QSNTTGYIEYCHIIEGEARLVDPDGTVHAVKAGDAFIMPEGYTGRWEVDRHVKKIYFVTH
LA
>non-antioxidant_1273
DDLNIKTMIPGVPQIDAESYILIDYNSGKVLAEQNADVRRDPASLTKMMTSYVIGQAMKA
GKFKETDLVTIGNDAWATGNPVFKGSSLMFLKPGMQVPVSQLIRGINLQSGNDACVAMA
DFAAGSQDAFVGLMNSYVNALGLKNTHFQTVHGLDADGQYSSARDMALIGQALIRDVP
NEYSIYKEKEFTFNGIRQLNRNGLLWDNSLNVDGIKTGHTDKAGYNLVASATEGQMRLIS
AVMGGRTFKGREAESKKLLTWGFRFFETVNPLKVGKEFASEPVWFGDSDRASLGVDKDV
YLTIPRGRMKDLKASYVLNSSELHAPLQKNQVVGTINFQLDGKTIEQRPLVVLQEIPEGNF
GDPVID
>non-antioxidant_1274
SNAMKPKLILMSHGRMAEETLASTQMIVGELADAAIVSMTAEDGLSGTQAKLAAILKEA
GNVPTLVLADLKGGTPCNVAMMAMGTYPQLRVVAGLNLAMAIEAAVSPVENVDELAAY
LTQIGQSAVTTIDLPELTDEEEFEE
>non-antioxidant_1275
MDPMYLLVDVGNTHSVFSITEDGKTFRRWRLSTGVFQTEDELFSHLHPLLGDAMREIKGI
GVASVVPTQNTVIERFSQKYFHISPIWVKAKNGCVKWNVKNPSEVGADRVANVVAFVKE
YGKNGIIIDMGTATTVDLVVNGSYEGGAILPGFFMMVHSLFRGTAKLPLVEVKPADFVVG
KDTEENIRLGVVNGSVYALEGIIGRIKEVYGDLPVVLTGGQSKIVKDMIKHEIFDEDLTIKG
VYHFCFGD
>non-antioxidant_1276
GSKLQICVEPTSQKLMPGSTLVLQCVAVGSPIPHYQWFKNELPLTHETKKLYMVPYVDLE
HQGTYWCHVYNDRDSQDSKKVEIIIDELNNL
>non-antioxidant_1277
ADSTITIRGYVRDNR
>non-antioxidant_1278
MGSSHHHHHHSSGLVPRGSMAQGLIEVERKFLPGPGTEERLQELGGTLEYRVTFRDTYYD
TPELSLMQADHWLRRREDSGWELKCPGAAGVLGPHTEYKELTAEPTIVAQLCKVLRADG
LGAGDVAAVLGPLGLQEVASFVTKRSAWKLVLLGADEEEPQLRVDLDTADFGYAVGEVE
ALVHEEAEVPTALEKIHRLSSMLGVPAQETAPAKLIVYLQRFRPQDYQRLLEVNSS
>non-antioxidant_1279
MSLAEPRYKADIGGGSLKLPESRIIAGLLLEGVTEDQWRHAIEVENVLQRRSPGTAKRQSS
LMRNRLETMGPELWQMVRDGSTQVAIQAVFAAAIKHSTLLGDFLDLVVRDQFRMFRPDL
PRKMWDQYLEQCRNRDPLMPVWQDSTANKLADCVYRILVEVGYITDSKTYRLKSVRISG
EVMSYLRENNEQYVIRCIQVSIEGHHHHHH

>non-antioxidant_1280
MAHHHHHHMAASANGSTKGSETDLPIGKDGTTLHLKCKSDELADRIIFVGDPGRVDVISG
YFDKDSIRASRDHREIRFATGTYKGTPVTVISTGMGVDNIEIVLNEIHALKEYDMERGQWR
HRKGDADAPSAGPFFDPSTMKIIRLGTCGSPAESVPPLALAVTRHAIGMDNTSLYYSAGTR
ETSKDQQEIRRIVREQTGLRAIDIYTSMAHPNITKSICAACDAHNAATGSEADKQQYVIGTT
ATASGFYGCQGRRVGRFMKHLTVPNMVEELGSLKFNLSNGVEVVTNIEMETSAICYLSDM
LGYQAGAACVVVSKRVGEKKMFLGDQLDAAMKRCIKIILEALVSA
>non-antioxidant_1281
MVEATAQETDRPRFSFSIAAREGKARTGTIEMKRGVIRTPAFMPVGTAATVKALKPETVRA
TGADIILGNTYHLMLRPGAERIAKLGGLHSFMGWDRPILTDSGGFQVMSLSSLTKQSEEGV
TFKSHLDGSRHMLSPERSIEIQHLLGSDIVMAFDECTPYPATPSRAASSMERSMRWAKRSR
DAFDSRKEQAENAALFGIQQGSVFENLRQQSADALAEIGFDGYAVGGLAGGEGQDEMFR
VLDFSVPMLPDDKPHYLMGVGKPDDIVGAVERGIDMFDCVLPTRSGRNGQAFTWDGPINI
RNARFSEDLKPLDSECHCAVCQKWSRAYIHHLIRAGEILGAMLMTEHNIAFYQQLMQKIR
DSISEGRFSQFAQDFRARYFARNS
>non-antioxidant_1282
MRPVFPLILSAVLFLSCFFGARQTEASASKRAIDANQIVNRMSLDEKLGQMLMPDFRNWQ
KEGESSPQALTKMNDEVASLVKKYQFGGIILFAENVKTTKQTVQLTDDYQKASPKIPLMLS
IDQEGGIVTRLGEGTNFPGNMALGAARSRINAYQTGSIIGKELSALGINTDFSPVVDINNNP
DNPVIGVRSFSSNRELTSRLGLYTMKGLQRQDIASALKHFPGHGDTDVDSHYGLPLVSHG
QERLREVELYPFQKAIDAGADMVMTAHVQFPAFDDTTYKSKLDGSDILVPATLSKKVMTG
LLRQEMGFNGVIVTDALNMKAIADHFGQEEAVVMAVKAGVDIALMPASVTSLKEEQKFA
RVIQALKEAVKNGDIPEQQINNSVERIISLKIKRGMYPARNSDSTKEKIAKAKKIVGSKQHL
KAEKKLAEKAVTVLKNEQHTLPFKPKKGSRILIVAPYEEQTASIEQTIHDLIKRKKIKPVSLS
KMNFASQVFKTEHEKQVKEADYIITGSYVVKNDPVVNDGVIDDTISDSSKWATVFPRAVM
KAALQHNKPFVLMSLRNPYDAANFEEAKALIAVYGFKGYANGRYLQPNIPAGVMAIFGQ
AKPKGTLPVDIPSVTKPGNTLYPLGYGLNIKTGRPL
>non-antioxidant_1283
MENREPPLLPARWSSAYVSYWSPMLPDDQLTSGYCWFDYERDICRIDGLFNPWSERDTGY
RLWMSEVGNAASGRTWKQKVAYGRERTALGEQLCERPLDDETGPFAELFLPRDVLRRLG
ARHIGRRVVLGREADGWRYQRPGKGPSTLYLDAASGTPLRMVTGDEASRASLRDFPNVS
EAEIPDAVFAAKRLEHHHHHH
>non-antioxidant_1284
MFSAGHKIKGTVVLMPKNELEVNPDGSAVDNLNAFLGRSVSLQLISATKADAHGKGKVG
KDTFLEGINTSLPTLGAGESAFNIHFEWDGSMGIPGAFYIKNYMQVEFFLKSLTLEAISNQG
TIRFVCNSWVYNTKLYKSVRIFFANHTYVPSETPAPLVEYREEELKSLRGNGTGERKEYDR
IYDYDVYNDLGNPDKSEKLARPVLGGSSTFPYPRRGRTGRGPTVTDPNTEKQGEVFYVPR
DENLGHLKSKDALEIGTKSLSQIVQPAFESAFDLKSTPIEFHSFQDVHDLYEGGIKLPRDVIS
TIIPLPVIKELYRTDGQHILKFPQPHVVQVSQSAWMTDEEFAREMIAGVNPCVIRGLEEFPP
KSNLDPAIYGDQSSKITADSLDLDGYTMDEALGSRRLFMLDYHDIFMPYVRQINQLNSAK
TYATRTILFLREDGTLKPVAIELSLPHSAGDLSAAVSQVVLPAKEGVESTIWLLAKAYVIVN
DSCYHQLMSHWLNTHAAMEPFVIATHRHLSVLHPIYKLLTPHYRNNMNINALARQSLINA
NGIAETTFLPSKYSVEMSSAVYKNWVFTDQALPADLIKRGVAIKDPSTPHGVRLLIEDYPY
AADGLEIWAAIKTWVQEYVPLYYARDDDVKNDSELQHWWKEAVEKGHGDLKDKPWWP

KLQTLEDLVEVCLIIIWIASALHAAVNFGQYPYGGLIMNRPTASRRLLPEKGTPEYEEMINN
HEKAYLRTITSKLPTLISLSVIEILSTHASDEVYLGQRDNPHWTSDSKALQAFQKFGNKLKE
IEEKLVRRNNDPSLQGNRLGPVQLPYTLLYPSSEEGLTFRGIPNSISI
>non-antioxidant_1285
GPPQMSATNEDLKTNFHSLHNQMRQMPMSHFREALDAPDYSGMRQSGFFAMSQGFQLE
SHGGDVFMHAHRENPQCKGDFAGDKFHISVQREQVPQAFQALSGLLFSVDSPIDKWKVT
DMERVDQQSRVAVGAQFTLYVKPDQENSQYSASSLHNTRQFIECLESRLSESGLMPGQYP
ESDVHPENWKYVSYRNELRSGRDGGEMQSQALREEPFYRLMAE
>non-antioxidant_1286
SISPAQIAEALQGRGWDAEIVTDASMAGQLVDVRPEGILKCVDGRGSDNTRMGGPKMPG
GIYAIAHNRGVTSIEGLKQITKEVASKGHLPSVHGDHSSDMLGCGFFKLWVTGRFDDMGY
PRPQFDADQGANAVKDAGGIIEMHHGSHTEKVVYINLLANKTLEPNENDQRFIVDGWAA
DKFGLDVPKFLIAAAATVEMLGGPKNAKIVVP
>non-antioxidant_1287
MQFVNKQFNYKDPVNGVDIAYIKIPNVGQMQPVKAFKIHNKIWVIPERDTFTNPEEGDLN
PPPEAKQVPVSYYDSTYLSTDNEKDNYLKGVTKLFERIYSTDLGRMLLTSIVRGIPFWGGS
TIDTELKVIDTNCINVIQPDGSYRSEELNLVIIGPSADIIQFECKSFGHEVLNLTRNGYGSTQY
IRFSPDFTFGFEESLEVDTNPLLGAGKFATDPAVTLAHELIHAGHRLYGIAINPNRVFKVNTN
AYYEMSGLEVSFEELRTFGGHDAKFIDSLQENEFRLYYYNKFKDIASTLNKAKSIVGTTAS
LQYMKNVFKEKYLLSEDTSGKFSVDKLKFDKLYKMLTEIYTEDNFVKFFKVLNRKTYLN
FDKAVFKINIVPKVNYTIYDGFNLRNTNLAANFNGQNTEINNMNFTKLKNFTGLFEF
>non-antioxidant_1288
SNAMNRLKNMSKYSDAKELASLTLGKKTEYANQYDPSLLQPVPRSLNRNDLHLSATLPFQ
GCDIWTLYELSWLNQKGLPQVAIGEVSIPATSANLIESKSFKLYLNSYNQTRFASWDEVQT
RLVHDLSACAGETVTVNVKSLNEYTAEPIVTMQGECIDDQDIEIANYEFDDALLQGAAQG
EEVSEVLHSHLLKSNCLITNQPDWGSVEIAYHGAKMNREALLRYLVSFREHNEFHEQCVE
RIFTDIMRYCQPQSLTVYARYTRRGGLDINPFRSSHQSAPNHNQRMARQ
>non-antioxidant_1289
MTDAAEEVLLGKKGCTGVITLNRPKFLNALTLNMIRQIYPQLKKWEQDPETFLIIIKGAGG
KAFCAGGDIRVISEAEKAKQKIAPVFFREEYMLNNAVGSCQKPYVALIHGITMGGGVGLS
VHGQFRVATEKCLFAMPETAIGLFPDVGGGYFLPRLQGKLGYFLALTGFRLKGRDVYRAG
IATHFVDSEKLAMLEEDLLALKSPSKENIASVLENYHTESKIDRDKSFILEEHMDKINSCFS
ANTVEEIIENLQQDGSSFALEQLKVINKMSPTSLKITLRQLMEGSSKTLQEVLTMEYRLSQ
ACMRGHDFHEGVRAVLIDKDQSPKWKPADLKEVTEEDLNNHFKSLGSSDLKFAENLYFQ
>non-antioxidant_1290
DGGFCEVCKKLVGYLDRNLEKNSTKQEILAALEKGCSFLPDPYQKQCDQFVAEYEPVLIEI
LVEVMDPSFVCLKIGACPS
>non-antioxidant_1291
MSLANLSELPNIGKVLEQDLIKAGIKTPVELKDVGSKEAFLRIWENDSSVCMSELYALEGA
VQGIRWHGLDEAKKIELKKFHQSLEGHHHHHH
>non-antioxidant_1292
MLEDLIGKAYLESAEDRRRGDRSEEVEAIRKYIRSARRTVVPNWNAEKVDAINDVLRSFN
LREAEHLQFNTNWADLTRMPAVTKALMALDISGADLVIARGRLGVPGSGSLLVIMDSRGR
LLSAAMSPPHVIHSMEVREAVRSEMTHALERIGFKR

>non-antioxidant_1293
MDNSVETIELKRGSNSVYVQYDDIMFFESSTKSHRLIAHLDNRQIEFYGNLKELSQLDDRF
FRCHNSFVVNRHNIESIDSKERIVYFKNKEHCYASVRNVKKI
>non-antioxidant_1294
MQQQCDTVSAWQSLRGPGTGGYYLFKTTEGGKTDCTYVKGSNFNDAAQTATYTYGNLG
SGNQLTQQTASASISGNAIVVGTDHSEVLYSDGSTCDVVRLNGQIELWIHSSATSNTGNLN
SCCTDKFNQEKGSRPEHVVYRSTCPNLPA
>non-antioxidant_1295
MSLSWEIEELDREIGKIKKHSLILIHEEDASSRGKDILFYILSRKLKSDNLVGMFSISYPLQLI
IRILSRFGVDVIKYLENHRLAIVDTFGSFHGIKATMPGVWYLEGMLSSETLPIKYAKAVEDH
KKVWMDLNLFEGRELYGFAISMSGYLEVFTPEETLRYLETSAEVRYGHPAYKKYPRGTNF
WLWEGVKDKRVLLSVYRRADYVLKTRSSLGENGIKRELLVIKTPKPIEELVRFEYEFKGNE
PKLRREGHHHHHH
>non-antioxidant_1296
SLRPPLPPATPQEVQFVQHMLQHHAQALDLAAPMLERSQQRTVRSLALDIQLSQREQMRQ
MEAMLGRWGQPPGEPISPEHARMMGMASEAEVAGLSTLPVEQAERQFLRLMIRHHQGAV
AMTLPMLDAAARPEVERLARQIVVTQRGEIRTMEGVLGRLDGEVPAAPMRPVEHGHGH
>non-antioxidant_1297
SNAKSAVTAQSILEKADEIRFPQDSFQVNVAIRTAAPDHAEDLYRYQVLSKGNENSIVMITE
PASERGQAILMKGRDLWVFMPSVSQPIRLSLSQRLTGQVANGDIARANFTGDYHPQLLRN
ESIDDEDYYVLELTGIDRSVTYQKVLLWVNQSNFRPYKAEFYSVSGRLLKTSRYENFDNIL
GEMRPTRIIMEDALKSGEVSVLDYSDMKLRDLPDKIFTKDYLKRLE
>non-antioxidant_1298
SNESVDYRATFPE
>non-antioxidant_1299
MGSSHHHHHHSQDPMLSKDIIKLLNEQVNKEMNSSNLYMSMSSWCYTHSLDGAGLFLFD
HAAEEYEHAKKLIIFLNENNVPVQLTSISAPEHKFEGLTQIFQKAYEHEQHISESINNIVDHA
IKSKDHATFNFLQWYVAEQHEEEVLFKDILDKIELIGNENHGLYLADQYVKGIAKSRKS
>non-antioxidant_1300
RSSHHHHHHGEFDDPIRPPLKVARSPRPGQCQDVVQDVPNVDVQMLELYDRMSFKDIDG
GVWKQGWNIKYDPLKYNAHHKLKVFVVPHSHNDPGWIQTFEEYYQHDTKHILSNALRH
LHDNPEMKFIWAEISYFARFYHDLGENKKLQMKSIVKNGQLEFVTGGWVMPDEANSHW
RNVLLQLTEGQTWLKQFMNVTPTASWAIAPFGHSPTMPYILQKSGFKNMLIQRTHYSVKK
ELAQQRQLEFLWRQIWDNKGDTALFTHMMPFYSYDIPHTCGPDPKVCCQFDFKRMGSFG
LSCPWKVPPRTISDQNVAARSDLLVDQWKKKAELYRTNVLLIPLGDDFRFKQNTEWDVQR
VNYERLFEHINSQAHFNVQAQFGTLQEYFDAVHQAERAGQAEFPTLSGDFFTYADRSDNY
WSGYYTSRPYHKRMDRVLMHYVRAAEMLSAWHSWDGMARIEERLEQARRELSLFQHH
DGITGTAKTHVVVDYEQRMQEALKACQMVMQQSVYRLLTKPSIYSPDFSFSYFTLDDSR
WPGSGVEDSRTTIILGEDILPSKHVVMHNTLPHWREQLVDFYVSSPFVSVTDLANNPVEA
QVSPVWSWHHDTLTKTIHPQGSTTKYRIIFKARVPPMGLATYVLTISDSKPEHTSYASNLLL
RKNPTSLPLGQYPEDVKFGDPREISLRVGNGPTLAFSEQGLLKSIQLTQDSPHVPVHFKFLK
YGVRSHGDRSGAYLFLPNGPASPVELGQPVVLVTKGKLESSVSVGLPSVVHQTIMRGGAP
EIRNLVDIGSLDNTEIVMRLETHIDSGDIFYTDLNGLQFIKRRRLDKLPLQANYYPIPSGMFI
EDANTRLTLLTGQPLGGSSLASGELEIMQDRRLASDDERGLGQGVLDNKPVLHIYRLVLE

KVNNCVRPSKLHPAGYLTSAAHKASQSLLDPLDKFIFAENEWIGAQGQFGGDHPSAREDL
DVSVMRRLTKSSAKTQRVGYVLHRTNLMQCGTPEEHTQKLDVCHLLPNVARCERTTLTFL
QNLEHLDGMVAPEVCPMETAAYVSSHSS
>non-antioxidant_1301
NVRFDLSSATSSSYKTFIKNLREALPKDGKVYDIPVLLSTVMDSRRFILIDLVNYDGQSITA
AIDVLNVYIVAYSTGTVSYFFQQVPAQAPKLLFKGTQQRTLPYTGNYENLQTAAKKLRENI
ELGLPALDSAITTLFHYNAEAAASALLVLIQTTSEAARFRYIELQIANNVGTKFKPSQTIISL
ENNWSALSKQIQIAKNKNGQFETPVILIDPQGNRVQITNVTSNVVTQNIQLLLNIGATA
>non-antioxidant_1302
MAPTSSIEIVLDKTTASVGEIVTASINIKNITNFSGCQLNMKYDPAVLQPVTSSGVAYTKST
MPGAGTILNSDFNLRQVADNDLEKGILNFSKAYVSLDDYRTAAAPEQTGTVAVVKFKVLK
EETSSISFEDTTSVPNAIDGTVLFDWNGDRIQSGYSVIQPAVINLDMIKASLEHHHHHH
>non-antioxidant_1303
ADTMTFTAKNGNVTFDHKKHQTIVPDCAVCHGKTPGKIEGFGKEMAHGKSCKGCHEEM
KKGPTKCGECHKK
>non-antioxidant_1304
MKRVLKSTEMSIGGSGENVLSVHPVLDDNSCLFHAIAYGIFKQDSVRDLREMVSKEVLNN
PVKFNDAILDKPNKDYAQWILKMESWGGAIEIGIISDALAVAIYVVDIDAVKIEKFNEDKFD
NYILILFNGIHYDSLTMNEFKTVFNKNQPESDDVLTAALQLASNLKQTGYSFNTHKAQIKC
NTCQMTFVGEREVARHAESTGHVDFGQNR
>non-antioxidant_1305
GSSQISDMTRDGLANKALAVARTLADSPEIRQGLQKKPQESGIQAIAEAVRKRNDLLFIVV
TDMQSLRYSHPEAQRIGQPFKGDDILKALNGEENVAINRGFLAQALRVFTPIYDENHKQIG
VVAIGLELSRVTQQINDSRW
>non-antioxidant_1306
GSPLPWCPHLVAVCPIPAAGLDVTQPCGDCGTIQENWVCLSCYQVYCGRYINGHMLQHH
GNSGHPLVLSYIDLSAWCYYCQAYVHHQALLDVKNIAHQNKFGEDMPHPH
>non-antioxidant_1307
ANADNYKNVINRTGAPQYMKDYDYDDHQRFNPFFDLGAWHGHLLPDGPNTMGGFPGV
ALLTEEYINFMASNFDRLTVWQDGKKVDFTLEAYSIPGALVQKLTAKDVQVEMTLRFATP
RTSLLETKITSNKPLDLVWDGELLEKLEAKEGKPLSDKTIAGEYPDYQRKISATRDGLKVT
FGKVRATWDLLTSGESEYQVHKSLPVQTEINGNRFTSKAHINGSTTLYTTYSHLLTAQEVS
KEQMQIRDILARPAFYLTASQQRWEEYLKKGLTNPDATPEQTRVAVKAIETLNGNWRSPGG
AVKFNTVTPSVTGRWFSGNQTWPWDTWKQAFAMAHFNPDIAKENIRAVFSWQIQPGDSV
RPQDVGFVPDLIAWNLSPERGGDGGNWNERNTKPSLAAWSVMEVYNVTQDKTWVAEM
YPKLVAYHDWWLRNRDHNGNGVPEYGATRDKAHNTESGEMLFTVKKGDKEETQSGLN
NYARVVEKGQYDSLEIPAQVAASWESGRDDAAVFGFIDKEQLDKYVANGGKRSDWTVKF
AENRSQDGTLLGYSLLQESVDQASYMYSDNHYLAEMATILGKPEEAKRYRQLAQQLAD
YINTCMFDPTTQFYYDVRIEDKPLANGCAGKPIVERGKGPEGWSPLFNGAATQANADAV
VKVMLDPKEFNTFVPLGTAALTNPAFGADIYWRGRVWVDQFWFGLKGMERYGYRDDAL
KLADTFFRHAKGLTADGPIQENYNPLTGAQQGAPNFSWSAAHLYMLYNDFFRKQ
>non-antioxidant_1308
MGSSHHHHHHSSGLVPRGSHMLEDPMGTLIRATTLSRLSFIDVVNDNGFYQFEKPKEGRK
YVATLDCSEGRGQDYHALQIIDITEFPYKQVAVYHSNTTSHFILPDIVFKYLMMYNECPVYI

ELNSTGVSIAKSLAMDLEYDNIICDSFIDLGMKQSKRSKAMGCSALKDLIEKDKLIINHKG
TIQELRTFSEKGVSWAAEEGFHDDLVMSLVIFGWLTTQEKFAEYAGKDE
>non-antioxidant_1309
MAFAHFVLIHTICHGAWIWHKLKPLLEALGHKVTALDLAASGVDPRQIEEIGSFDEYSEPL
LTFLEALPPGEKVILVGESCGGLNIAIAADKYCEKIAAAVFHNSVLPDTEHCPSYVVDKLM
EVFPDWKDTTYFTYTKDGKEITGLKLGFTLLRENLYTLCGPEEYELAKMLTRKGSLFQNIL
AKRPFFTKEGYGSIKKIYVWTDQDEIFLPEFQLWQIENYKPDKVYKVEGGDHKLQLTKTK
EIAEILQEVADTYN
>non-antioxidant_1310
SLRSMVSDSVDEIVDGVSKTTAEVINGRKSIAQYATSLIENNPEPDNVRTIISQPLIKNTFLLV
GFGLEKDGSNINNDPSWNPGPTWDPRVRPWYKDAKNAGKLVITAPYADSASGEILVSVAT
PVKDSATGQFLGSIFYDVSLAELAELVNEVKLFDAGYVFIVSEDGTTIAHPKKEFNGKPMS
EFLGESKINVDTHQVIINGKPYAVSFSDVEGEDWYVGVVIDEEIAYAALDELRRS
>non-antioxidant_1311
GMARKRLIIEMGMGIDQHGQEPTIAASRAVRNAIAHNALPGVWEVAGLSHPNEMIIEVQV
AVPYPEQVREEEVLAVLPFGRKTLTVESGGMIVQGRAIPELNDKNDEMLIAIAAVTVLIENE
>non-antioxidant_1312
GMDKYLSANSLEGVIDNEFSMPAPRWLNTYPAGPYRFINREFFIIAYETDPDLLQAILPPDM
ELLEPVVKFEFIRMPDSTGFGDYTESGQVVPVRYKGEEGGFTISMFLDCHAPIAGGREIWG
FPKKLAKPKLFVEEDTLIGILKYGSIDIAIATMGYKHRPLDAEKVLESVKKPVFLLKNIPNV
DGTPLVNQLTKTYLTDITVKGAWTGPGSLELHPHALAPISNLYIKKIVSVSHFITDLTLPYG
KVVADYLA

>non-antioxidant_1313
RSIIGGKHGDRDVRILYQVGDSEEDLPVCAPNAVCSKIDLYETPWIERQCRCPDGRTCPSSL
GVEDGHTIADKTRHYKMCQPVHKLPVCKHFRDYTWTLTTAAELNVTEQIVHCRCPRNSV
TYLTKREPIGNDSPGYRYLFACSPLTRLRCQRKQPCKLFTVRKRQEFLDEVNINSLCQCPK
GHRCPSHHTQSGVIAGESFLEDNIQTYSGYCMANDHHHHHH
>non-antioxidant_1314
SEGNGPAAVHYQPASPPRDACVYSSCYCEENVWKLCEYIKNHDQYPLEECYAVFISNERK
MIPIWKQQARPGDGPVIWDYHVVLLHVSSGGQSFIYDLDTVLPFPCLFDTYVEDAIKSDD
DIHPQFRRKFRVICADSYLKNFASDRSHMKDSSGNWREPPPPYPCIETGDSKMNLNDFISM
DPKVGWGAVYTLSEFTHRFGSKNC
>non-antioxidant_1315
MGSHHHHHHDITSLYKKAGSAAAVLEENLYFQGSFTMRLKELGEFGLIDLIKKTLESKVIG
DDTAPVEYCSKKLLLTTDVLNEGVHFLRSYIPEAVGWKAISVNVSDVIANGGLPKWALISL
NLPEDLEVSYVERFYIGVKRACEFYKCEVVGGNISKSEKIGISVFLVGETERFVGRDGARL
GDSVFVSGTLGDSRAGLELLLMEKEEYEPFELALIQRHLRPTARIDYVKHIQKYANASMDI
SDGLVADANHLAQRSGVKIEILSEKLPLSNELKMYCEKYGKNPIEYALFGGEDYQLLFTHP
KERWNPFLDMTEIGRVEEGEGVFVDGKKVEPKGWKHF
>non-antioxidant_1316
TFPTYKCPETFDAWYCLNDAHCFAVKIADLPVYSCECAIGFMGQRCEYKEID
>non-antioxidant_1317
MAMAEAAEKAAKYAAEAAEKAAKAMAM

>non-antioxidant_1318
MFEQEVTITAPNGLDTRPAAQFVKEAKGFTSEITVTSNGKSASAKSLFKLQTLGLTQGTVV
TISAEGEDEQKAVEHLVKLMAELE
>non-antioxidant_1319
MTPQNITDLCAEYHNTQIHTLNDKIFSYTESLAGKREMAIITFKNGATFQVEVPGSQHIDSQ
KKAIERMKDTLRIAYLTEAKVEKLCVWNNKTPRAIAAISMAN
>non-antioxidant_1320
GDIEQKQAEIIDQLVKRASTCKSEALGPLIIEATSHPSLFAFSEILALPNVAQLEGTTDSVYLD
LLRLFAHGTWGDYKCNATRLPHLSPDQILKLKQLTVLTLAESNKVLPYDTLMVELDVSNV
RELEDFLINECMYAGIVRGKLDQLKRCFEVPFAAGRDLRPGQLGN
>non-antioxidant_1321
GMDTPANKPCIICVAITGSVPTKADNPAVPITVSEQVESTQEAFEAGAAIAHCHVRNDDGT
PSSDPDRFARLTEGLHTHCPGMIVQFSTGGRSGAGQARGGMLPLKPDMASLSVGSNNFPS
RVYENPPDLVDWLAAQMRSYRVTPEIEAFDLSHILRAIDMHGRGLLYGKLYVQFVMGVK
NAMPADREVFDFYVRMMRTRAPQAEWCAAGIGANQLTVNEWAIAAGGHTRTGLEDNIR
LDRQTLAPSNAALVRRSVELCDKYQRPVASWQQAREILGLPAAARN
>non-antioxidant_1322
GASAPMVKIYTKNGDKGQTRIIGKQILYKNDPRVAAYGEVDELNSWVGYTKSLINSHTQV
LSNELEEIQQLLFDCGHDLATPADDERHSFKFKQEQPTVWLEEKIDNYTQVVPAVKKFILP
GGTQLASALHVARTITRRAERQIVQLMREEQINQDVLIFINRLSDYFFAAARYANYLEQQP
DMLYRNSKDVFR
>non-antioxidant_1323
GHMNVKLKVFHAGSLTEPMKAFKRAFEEKHPNVEVQTEAAGSAATIRKVTELGRKADVI
ATADYTLIQKMMYPEFANWTIMFAKNQIVLAYRNDSRYADEINSQNWYEILKRPDVRFGF
SNPNDDPCGYRSLMAIQLAELYYNDPTIFDELVAKNSNLRFSEDNGSYVLRMPSSERIEINK
SKIMIRSMEMELIHLVESGELDYFFIYKSVAKQHGFNFVELPVEIDLSSPDYAELYSKVKVV
LANGKEVTGKPIVYGITIPKNAENRELAVEFVKLVISEEGQEILRELGQEPL
>non-antioxidant_1324
MSIAVGMIETRGFPAVVEAADSMVKAARVTLVGYEKIGSGRVTVIVRGDVSEVQASVSAG
IEAANRVNGGEVLSTHIIARPHENLEYVLPILEHHHHHH
>non-antioxidant_1325
MWVYRLKGTLEALDPILPGLFDGGARGLWEREGEVWAFFPAPVDLPYEGVWEEVGDED
W
>non-antioxidant_1326
MKKVVAVVKLQLPAGKATPAPPVGPALGQHGANIMEFVKAFNAATANMGDAIVPVEITIY
ADRSFTFVTKTP
>non-antioxidant_1327
GINDLDISPIDPQTGGSFDQQGVFVKGYAMLGVTGQKGIDGSPDLDGQDEGESGFYRTTF
NCNELPTDECLWAWQKNQDIPQLTSISWSPSSQRTEWVYVRLGYDITQYNFFLDQTEGMT
DAETLRQRAEIRFLRALHYWYFLDLFGKAPFKEHFSNDLPVEKKGTELYTYIQNELNEIEA
DMYEPRQAPFGRADKAANWLLRARLYLNAGVYTGQTDYAKAEEYASKVIGSAYKLCTN
YSELFMADNDENENAMQEIILPIRQDGVKTRNYGGSTYLVCGTRVAGMPRMGTTNGWSC
IFARAAMVQKFFSNLEDVPMLPADVEIPTKGLDTDEQIDAFDAEHGIRTEDMIKAAGDDR
ALLYSGVGGGRRKIQTDAISGFTDGLSIVKWQNYRSDGKPVSHATYPDTDIPLFRLAEAYL

TRAEAIFRQGGDATGDINELRKRANCTRKVQTVTEQELIDEWAREFYLEGRRRSDLVRFG
MFTTNKYLWDWKGGAMNGTSVASYYNKYPIPVSDINNNRNMSQNEGYK
>non-antioxidant_1328
MVSQIGLLLPLSGDGQILGTTIQSGFNDAKGNSTIPVQVFDTSMNSVQDIIAQAKQAGIKTL
VGPLLKQNLDVILADPAQIQGMDVLALNATPNSRAIPQLCYYGLSPEDEAESAANKMWN
DGVRNPLVAMPQNDLGQRVGNAFNVRWQQLAGTDANIRYYNLPADVTYFVQENNSNTT
ALYAVASPTELAEMKGYLTNIVPNLAIYASSRASASATNTNTDFIAQMNGVQFSDIPFFKDT
NSPQYQKLAKSTGGEYQLMRLYAMGADAWLLINQFNELRQVPGYRLSGLTGILSADTNC
NVERDMTWYQYQDGAIVPVVDHHHHHH
>non-antioxidant_1329
GMTILSDVKALGQQIWLDNLSRSLVQSGELAQMLKQGVCGVTSNPAIFQKAFAGDALYA
DEVAALKRQNLSPKQRYETMAVADVRAACDVCLAEHESTGGKTGFVSLEVSPELAKDAQ
GTVEEARRLHAAIARKNAMIKVPATDAGIDALETLVSDGISVNLTLLFSRAQTLKAYAAYA
RGIAKRLAAGQSVAHIQVVASFFISRVDSALDATLPDRLKGKTAIALAKAAYQDWEQYFTA
PEFAALEAQGANRVQLLWASTGVKNPAYPDTLYVDSLIGVHTVNTVPDATLKAFIDHGTA
KATLTESADEARARLAEIAALGIDVETLAARLQEDGLKQFEEAFEKLLAPLV
>non-antioxidant_1330
GPEIRMDKKSLYKYLLLRSTGDMHKAKSPTIMTRVTNNVYLGNYKNAMDAPSSEVKFKY
VLNLTMDKYTLPNSNINIIHIPLVDDTTTDISKYFDDVTAFLSKCDQRNEPVLVHSAAGVNR
SGAMILAYLMSKNKESLPMLYFLYVYHSMRDLRGAFVENPSFKRQIIEKYVIDKN
>non-antioxidant_1331
AMAIPAFHPGELNVYSAPGDVADVSRALRLTGRRVMLVPTMGALHEGHLALVRAAKRVP
GSVVVVSIFVNPMQFGAGGDLDAYPRTPDDDLAQLRAEGVEIAFTPTTAAMYPDGLRTTV
QPGPLAAELEGGPRPTHFAGVLTVVLKLLQIVRPDRVFFGEKDYQQLVLIRQLVADFNLDV
AVVGVPTVREADGLAMSSRNRYLDPAQRAAAVALSAALTAAAHAATAGAQAALDAARA
VLDAAPGVAVDYLELRDIGLGPMPLNGSGRLLVAARLGTTRLLDNIAIEIGTFAGTDRPDG
YR
>non-antioxidant_1332
QNPADSPHIGKVFFSTNQGDFVCSANIVASANQSTVATAGHCLHDGNGGQFARNFVFAPAY
DYGESEHGVWAAEELVTSAEWANRGDFEHDYAFAVLETKGGTTVQQQVGTASPIAFNQP
RGQYYSAYGYPAAAPFNGQELHSCHGTATNDPMGSSTQGIPCNMTGGSSGGPWFLGNGT
GGAQNSTNSYGYTFLPNVMFGPYFGSGAQQNYNYASTTN
>non-antioxidant_1333
GIEKIISRSMFDQMLKHRNNPACPAKGFYTYDAFIAAAKSFPSFGTTGSTDVRKREIAAFLG
QTSHETTGGWPSAPDGPYAWGYCFLKERNPSSNYCAPSPRYPCAPGKSYYGRGPIQLSWN
YNYGPCGEALRVNLLGNPDLVATDRVISFKTALWFWMTPQAPKPSCHDVITGRWQPSAAD
TAAGRLPGYGVITNIINGGLECGKGPNPQVADRIGFFRRYCGILGVGTGNNLDCYNQRPFG
>non-antioxidant_1334
MVYVSNKYLTMSEMKVNAQYILNYLSSNGWTKQAICGMLGNMQSESTINPGLWQNLDE
GNTSLGFGLVQWTPASNYINWANSQGLPYKNMDSELKRIIWEVNNNAQWINLRDMTFKE
YIKSTKTPRELAMIFLASYERPANPNQPERGDQAEYWYKNLS
>non-antioxidant_1335
MTYGIVIVSHSPEIASGLKKLIREVAKNISLTAIGGLENGEIGTSFDRVMNAIEENEADNLLT
FFDLGSARMNLDLVSEMTDKELTIFNVPLIEGAYTASALLEAGATFEAIKEQLEKMLIEKRS

HHHHHH
>non-antioxidant_1336
SLANIREIAKRAGISIATVSRHLNNTGYVSEDAREKIQKVVDELNYTPNALARAMFTKNSK
TIGLMVPNISNPFFNQMASVIEEYAKNKGYTLFLCNTDDDKEKEKTYLEVLQSHRVAGIIA
SRSQCEDEYANIDIPVVAFENHILDNIITISSDNYNGGRMAFDHLYEKGCRKILHIKGPEVFE
ATELRYKGFLDGARAKDLEIDFIEFQHDFQVKMLEEDINSMKDIVNYDGIFVFNDIAAATV
MRALKKRGVSIPQEVQIIGFDNSFIGELLYPSLTTINQPIEALAYTIIELLIKIINGEGVLIEDYI
MEVKLIERETTISLKDEG
>non-antioxidant_1337
VHFHPFGNVNFYEMDWSLKGDLWAHDPVIAKEGSRWYVFHTGSGIQIKTSEDGVHWEN
MGWVFPSLPDWYKQYVPEKDEDHLWAPDICFYNGIYYLYYSVSTFGKNTSVIGLATNQTL
DPRDPDYEWKDMGPVIHSTASDNYNAIDPNVVFDQEGQPWLSFGSFWSGIQLIQLDTETM
KPAAQAELLTIASRGEEPNAIEAPFIVCRNGYYYLFVSFDFCCRGIESTYKIAVGRSKDITGP
YVDKNGVSMMQGGGTILDEGNDRWIGPGHCAVYFSGVSAILVNHAYDALKNGEPTLQIR
PLYWDDEGWPYLSV
>non-antioxidant_1338
TEQATTTDELAFTRPYGEQEKQILTAEAVEFLTELVTHFTPQRNKLLAARIQQQQDIDNGTL
PDFISETASIRDADWKIRGIPADLEDRRVEITGPVERKMVINALNANVKVFMADFEDSLAP
DWNKVIDGQINLRDAVNGTISYTNEAGKIYQLKPNPAVLICRVRGLHLPEKHVTWRGEAIP
GSLFDFALYFFHNYQALLAKGSGPYFYLPKTQSWQEAAWWSEVFSYAEDRFNLPRGTIKA
TLLIETLPAVFQMDEILHALRDHIVGLNCGRWDYIFSYIKTLKNYPDRVLPDRQAVTMDKP
FLNAYSRLLIKTCHKRGAFAMGGMAAFIPSKDEEHNNQVLNKVKADKSLEANNGHDGT
WIAHPGLADTAMAVFNDILGSRKNQLEVMREQDAPITADQLLAPCDGERTEEGMRANIRV
AVQYIEAWISGNGCVPIYGLMEDAATAEISRTSIWQWIHHQKTLSNGKPVTKALFRQMLG
EEMKVIASELGEERFSQGRFDDAARLMEQITTSDELIDFLTLPGYRLLA
>non-antioxidant_1339
MGSSHHHHHHSSGLVPRGSHMDKGKSVKSTEKSVGMPPKTPKTDNNAHVDNEFLILQVN
DAVFPIGSYTHSFGLETYIQQKKVTNKESALEYLKANLSSQFLYTEMLSLKLTYESALQQD
LKKILGVEEVIMLSTSPMELRLANQKLGNRFIKTLQAMNELDMGEFFNAYAQKTKDPTHA
TSYGVFAASLGIELKKALRHYLYAQTSNMVINCVKSVPLSQNDGQKILLSLQSPFNQLIEK
TLELDESHLCTASVQNDIKAMQHESLYSRLYMS
>non-antioxidant_1340
APDWVPPEVFDLVAEDKARCMSEHGTTQAQIDDVDKGNLVNEPSITCYMYCLLEAFSLVD
DEANVDEDIMLGLLPDQLQERAQSVMGKCLPTSGSDNCNKIYNLAKCVQESAPDVWFVI
>non-antioxidant_1341
AMSKIICLTAGHSNTDPGAVNGSDREADLAQDMRNIVASILRNDYGLTVKTDGTGKGNMP
LRDAVKLIRGSDVAIEFHTNAAANKTATGIEALSTPKNKRWCQVLGKAVAKKTGWKLRGE
DGFKPDNAGQHSRLAYAQAGGIVFEPFFISNDTDLALFKTTKWGICRAIADAIAMELGAA
KV
>non-antioxidant_1342
SSSVPSQKTYQGSYGFRLGFLHSGTAKSVTCTYSPALNKMFCQLAKTCPVQLWVDSTPPP
GTRVRAMAIYKQSQHMTEVVRRCPHHERCSDSDGLAPPQHLIRVEGNLRVEYLDDRNTF
RHSVVVPYEPPEVGSDCTTIHYNYMCNSSCMGGMNRSPILTIITLEDSSGNLLGRNSFEVR
VCACPGRDRRTEEENLRKKG

>non-antioxidant_1343
SNAMKPDIYENNREGILCVYKNEKWLVCIKNWKPDNDIEGIAHLEIHHSTDEQFILSAGKA
ILITAEKENDKFNIELTLMEKGKVYNVPAECWFYSITQKDTKMMYVQDSNCSMDNSDFC
DLSKEEIEYIQTNARKLFEK
>non-antioxidant_1344
MWKAVMNAWNGTESQSKNVSNIQSYSFEDMKRIVGKHDPNVVLVDVREPSEYSIVHIPA
SINVPYRSHPDAFALDPLEFEKQIGIPKPDSAKELIFYCASGKRGGEAQKVASSHGYSNTSL
YPGSMNDWVSHGGDKLDL
>non-antioxidant_1345
SRTDRLEVCREYQRGNCNRGENDCRFAHPADSTMIDTNDNTVTVCMDYIKGRCSREKCK
YFHPPAHLQAK
>non-antioxidant_1346
GSMKFVYKEEHPFEKRRSEGEKIRKKYPDRVPVIVEKAPKARIGDLDKKKYLVPSDLTVG
QFYFLIRKRIHLRAEDALFFFVNNVIPPTSATMGQLYQEHHEEDFFLYIAYSDESVYGL
>non-antioxidant_1347
GAMEPAARRRARECAVQALYSWQLSQNDIADVEYQFLAEQDVKDVDVLYFRELLAGVAT
NTAYLDGLMKPYLSRLLEELGQVEKAVLRIALYELSKRSDVPYKVAINEAIELAKSFGAED
SHKFVNGVLDKAAPVIRPNKK
>non-antioxidant_1348
GPLGSMQNQRIRIRLKAFDHRLIDQATAEIVETAKRTGAQVRGPIPLPTRSRTHLRLVDIVEP
TEKTVDALMRLDLAAGVDVQISLG
>non-antioxidant_1349
GEDKKGGTKPSNEAALTKTENLDFRLSFNKIKVTTDQNHFSGGTSIEQLKQWFGDPNKSE
QRNAGNITLDSYTWVKDGAVINAQLYKNSTVARSISNFSFSREAKIGKEDYDELKIGESYK
KIVEKLGEPDVLSQSMSSDKEEMQTVWSSGIKTKSSSATIELYFENGLLKNKTQKDLE
>non-antioxidant_1350
AAASFGQTKIPRGNGPYSVGCTDLMFDHTNKGTFLRLYYPSQDNDRLDTLWIPNKEYFW
GLSKFLGTHWLMGNILRLLFGSMTTPANWNSPLRPGEKYPLVVFSHGLGAFRTLYSAIGID
LASHGFIVAAVEHRDRSASATYYFKDQSAAEIGDKSWLYLRTLKQEEETHIRNEQVRQRA
KECSQALSLILDIDHGKPVKNALDLKFDMEQLKDSIDREKIAVIGHSFGGATVIQTLSEDQR
FRCGIALDAWMFPLGDEVYSRIPQPLFFINSEYFQYPANIIKMKKCYSPDKERKMITIRGSV
HQNFADFTFATGKIIGHMLKLKGDIDSNVAIDLSNKASLAFLQKHLGLHKDFDQWDCLIEG
DDENLIPGTNINTTNQHI
>non-antioxidant_1351
MGSSHHHHHHSSGLVPRGSHMFSITVRDHIMIAHSFRGDVFGPAQRLHGATFLVDATFRRE
QLDEDNIVVDIGLATQELGAVVGALNYRNLDNEPDFAGVNTSTEFLAKVIADRLAERVHK
GALGEGARGLAGLTVTLHESHVAWASYERAL
>non-antioxidant_1352
MEAVKTFNSELYSLNDYKPPISKAKMTQITKAAIKAIKFYKHVVQSVEKFIQKCKPEYKVP
GLYVIDSIVRQSRHQFGQEKDVFAPRFSNNIISTFQNLYRCPGDDKSKIVRVLNLWQKNNV
FKSEIIQPLLDMAAALEHHHHHH
>non-antioxidant_1353
MKALRGMISEKGGWNLTVNNDNNTVVSSGGALDLSSGSKNLKIVKDGKKNNVTFDVAR
DLTLKSIKLDGVTLNETGLFIANGPQITASGINAGSQKITGVAEGTDANDAVNFGQLKKIET

EVKEQVAASGFVKQDSDTKYLTIGKDTDGDTINIANNKSDKRT
>non-antioxidant_1354
GADDDKPIQVTQMPQLAQQFIKQHFSDSKVALAKMESDFLYKSYEVIFTNGNKVEFDKKG
NWEEVDCKHTSVPVAIIPAAIQKYVTTNYPDAKVLKIERDKKDYEVKLSNRTELKFDLKF
NLIDIDN
>non-antioxidant_1355
MPHDPGATRLLAISPHLDDAVLSFGAGLAQAAQDGANVLVYTVFAGAAQPPYSPAAQRM
HTIWGLAPDDDAVLYRRKEDIAALDHLRVAHRHGRFLDSIYRKLPDGRWLTAHVEGRQKL
AVNDHSPDSDHDLVGEVADDIRSIIDEFDPTLVVTCAAIGEHPDHEATRDAALFATHEKNVP
VRLWEDLPYAVFKSGAVELPQGFRLGSADVSSVKPEMRSQKFQAVERYSSQMVLLNGSEN
NLFDRLDEHARQNAPHGGYGETTWPVVRSDDS
>non-antioxidant_1356
MSEQAPAPKRGRRFKEQTPVQRALGLLVHREHSKKELNRKLQARGIEPEAAQAAVERLA
GEGWQDDVRFAASVVRNRASSGYGPLHIRAELGTHGLDSDAVSAAMATFEGDWTENAL
DLIRRRFGEDGPVDLAQRRKAADLLARRGFDGNSIRLATRFDLED
>non-antioxidant_1357
MQQASTPTIGMIVPPAAGLVPADGARLYPDLPFIASGLGLGSVTPEGYDAVIESVVDHARR
LQKQGAAVVSLMGTSLSFYRGAAFNAALTVAMREATGLPCTTMSTAVLNGLRALGVRRV
ALATAYIDDVNERLAAFLAEESLVPTGCRSLGITGVEAMARVDTATLVDLCVRAFEAAPDS
DGILLSCGGLLTLDAIPEVERRLGVPVVSSSPAGFWDAVRLAGGGAKARPGYGRLFDESLE
HHHHHH
>non-antioxidant_1358
GRISMTVKKLYFIPAGRCMLDHSSVNSALTPGKLLNLPVWCYLLETEEGPILVDTGMPESA
VNNEGLFNGTFVEGQILPKMTEEDRIVNILKRVGYEPDDLLYIISSHLHFDHAGGNGAFTN
TPIIVQRTEYEAALHREEYMKECILPHLNYKIIEGDYEVVPGVQLLYTPGHSPGHQSLFIET
EQSGSVLLTIDASYTKENFEDEVPFAGFDPELALSSIKRLKEVVKKEKPIIFFGHDIEQEKSC
RVFPEYI
>non-antioxidant_1359
GMTDAPADAPLDADARRAVKPVICYPNDSLPRPDLALYRAARASARKTGEVLVPPREGRC
FEVKAGQFFRISSVEGPQVGDLNLHNLHDLTERFFSGKTRALHGTHVTTGERLWSNLPYL
RPMATIIEDTLGWYGIDQYGGSVHDVIGTRCDPYTGNLLAGGHYHHCCHSNLTRALADH
TGLPLHEAEMLVHDVLNVFMCTGFTRDTGQYFMKASPVRPGDYLEFFAEIDLLGNLSACP
GGDCSSEHSSDTASCHPLLVEIFAPAEGMLGDWPSPSVNGYDRSHGR
>non-antioxidant_1360
ACRQEPQPQGPPPAAGAVASYDYLVIGGGSGGLASARRAAELGARAAVVESHKLGGTCV
NVGCVPKKVMWNTAVHSEFMHDHADYGFPSCEGKFNWRVIKEKRDAYVSRLNAIYQNN
LTKSHIEIIRGHAAFTSDPKPTIEVSGKKYTAPHILIATGGMPSTPHESQIPGASLGITSDGFF
QLEELPGRSVIVGAGYIAVEMAGILSALGSKTSLMIRHDKVLRSFDSMISTNCTEELENAG
VEVLKFSQVKEVKKTLSGLEVSMVTAVPGRLPVMTMIPDVDCLLWAIGRVPNTKDLSLNK
LGIQTDDKGHIIVDEFQNTNVKGIYAVGDVCGKALLTPVAIAAGRKLAHRLFEYKEDSKLD
YNNIPTVVFSHPPIGTVGLTEDEAIHKYGIENVKTYSTSFTPMYHAVTKRKTKCVMKMVC
ANKEEKVVGIHMQGLGCDEMLQGFAVAVKMGATKADFDNTVAIHPTSSEELVTLR
>non-antioxidant_1361
GMSENKKKFDKKGAKNMDEISKTLFAPIYPIIAENIINRFGITAGTCIDIGSGPGALSIALAK

QSDFSIRALDFSKHMNEIALKNIADANLNDRIQIVQGDVHNIPIEDNYADLIVSRGSVFFWE
DVATAFREIYRILKSGGKTYIGGGFGNKELRDSISAEMIRKNPDWKEFNRKNISQENVERF
QNVLDEIGISSYEIILGDEGFWIIISKTDQEVI
>non-antioxidant_1362
TQKPSLYRVLILNDDYTPMEFVVYVLERFFNKSREDATRIMLHVHQNGVGVCGVYTYEV
AETKVAQVIDSARRHQHPLQCTMEKD
>non-antioxidant_1363
MPKLVTWMNNQRVGELTKLANGAHTFKYAPEWLASRYARPLSLSLPLQRGNITSDAVFNF
FDNLLPDSPIVRDRIVKRYHAKSRQPFDLLSEIGRDSVGAVTLIPEDETVTHPIMAWEKLTE
ARLEEVLTAYKADIPLGMIREENDFRISVAGAQEKTALLRIGNDWCIPKGITPTTHIIKLPIGE
IRQPNATLDLSQSVDNEYYCLLLAKELGLNVPDAEIIKAGNVRALAVERFDRRWNAERTV
LLRLPQEDMCQTFGLPSSVKYESDGGPGIARIMAFLMGSSEALKDRYDFMKFQVFQWLIG
ATQGHAKNFSVFIQAGGSYRLTPFYDIISAFPVLGGTGIHISDLKLAMGLNASKGKKTAIDK
IYPRHFLATAKVLRFPEVQMHEILSDFARMIPAALDNVKTSLPTDFPENVVTAVESNVLRLH
GRLSREYGSK
>non-antioxidant_1364
ITGTSTVGVGRGVLGDQKNINTTYSTYYYLQDNTRGNGIFTYDAKYRTTLPGSLWADAD
NQFFASYDAPAVDAHYYAGVTYDYYKNVHNRLSYDGNNAAIRSSVHYSQGYNNAFWNG
SQMVYGDGDGQTFIPLSGGIDVVAHELTHAVTDYTAGLIYQNESGAINEAISDIFGTLVEFY
ANKNPDWEIGEDVYTPGISGDSLRSMSDPAKYGDPDHYSKRYTGTQDNGGVHINSGIINK
AAYLISQGGTHYGVSVVGIGRDKLGKIFYRALTQYLTPTSNFSQLRAAAVQSATDLYGSTS
QEVASVKQAFDAVGVK
>non-antioxidant_1365
MSLQLRSRAAFKLEFLLDRYRVVRKGDAVIEIGSSPGGWTQVLNSLARKIISIDLQEMEEIA
GVRFIRCDIFKETIFDDIDRALREEGIEKVDDVVSDAMAKVSGIPSRDHAVSYQIGQRVMEI
AVRYLRNGGNVLLKQFQGDMTNDFIAIWRKNFSSYKISKPPASRGSSSEIYIMFFGFKAEG
HHHHHH
>non-antioxidant_1366
TWTYILRQGDLPPGEMQRYEGGPEPVMVCNVDGEFFAVQDTCTHGDWALSDGYLDGDIV
ECTLHFGKFCVRTGKVKALPACKPIKVFPIKVEGDEVHVDLDNGELK
>non-antioxidant_1367
GFKQDIATIRGDLRTYAQDIFLAFLNKYPDERRYFKNYVGKSDQELKSMAKFGDHTEKVF
NLMMEVADRATDCVPLASDANTLVQMKQHSSLTTGNFEKLFVALVEYMRASGQSFDSQS
WDRFGKNLVSALSSAGMK
>non-antioxidant_1368
SNAEELIEIREARMDDLDTIAKFNYNLAKETEGKELDMDVLTKGVKALLLDERKGKYHV
YTVFDKVVAQIMYTYEWSDWRNGNFLWIQSVYVDKEYRRKGIFNYLFNYIKNICDKDEN
IVGMRLYVEKENINAKATYESLNMYECDYNMYEYEVIHS
>non-antioxidant_1369
VDMSNVVKTYDLQDGSKVHVFKDGKMGMENKFGKSMNMPEGKVMETRDGTKIIMKG
NEIFRLDEALRKGHSEGG
>non-antioxidant_1370
GSMPPQLHNGLDFSAKVIQGSLDSLPQEVRKFVEGNAQLCQPEYIHICDGSEEEYGRLLA
HMQEEGVIRKLKKYDNCWLALTDPRDVARIESKTVIITQEQRDTVPIPKSGQSQLGRWMS

EEDFEKAFNARFPGCMKGRTMYVIPFSMGPLGSPLAKIGIELTDSPYVVASMRIMTRMGTS
VLEALGDGEFIKCLHSVGCPLPLKKPLVNNWACNPELTLIAHLPDRREIISFGSGYGGNSLL
GKKCFALRIASRLAKEEGWLAEHMLILGITNPEGKKKYLAAAFPSACGKTNLAMMNPTL
PGWKVECVGDDIAWMKFDAQGNLRAINPENGFFGVAPGTSVKTNPNAIKTIQKNTIFTNV
AETSDGGVYWEGIDEPLAPGVTITSWKNKEWRPQDEEPCAHPNSRFCTPASQCPIIDPAWE
SPEGVPIEGIIFGGRRPAGVPLVYEALSWQHGVFVGAAMRSEATAAAEHKGKVIMHDPFA
MRPFFGYNFGKYLAHWLSMAHRPAAKLPKIFHVNWFRKDKNGKFLWPGFGENSRVLEW
MFGRIEGEDSAKLTPIGYVPKEDALNLKGLGDVNVEELFGISKEFWEKEVEEIDKYLEDQ
VNADLPYEIERELRALKQRISQM
>non-antioxidant_1371
MGPFDPEEMLFIFTRCMEDNLEDGANRLPMLAKWKEWINEPVDSPATQCFGKCVLVRTGL
YDPVAQKFDASVIQEQFKAYPSLGEKSKVEAYANAVKQLPSTNNDCAAVFKAYDPVHKA
HKDTSKNLFHGNKELTKGLYEKLGKDIRQKKQSYFEFCENKYYPAGSDKRQQLCQIRQYT
VLDDALFKEHTDCVMKGIRYITKDNQLDVEEVKRDFKLVNKDTKALEEVLNDCKSKEPS
NAKEKSWHYYKCLVESSVKDDFKEAFDYREVRSQIYAFNLPKNQAYSKPAVQSQVMEID
GKQCPQ
>non-antioxidant_1372
GATVAPSSRRDFTFDLYRALASAAPSQNIFFSPVSISMSLAMLSLGAGSSTKMQILEGLGLN
LQKSSEKELHRGFQQLLQELNQPRDGFQLSLGNALFTDLVVDLQDTFVSAMKTLYLADTF
PTNFRDSAGAMKQINDYVAKQTKGKIVDLLKNLDSNAVVIMVNYIFFKAKWETSFNHKG
TQEQDFYVTSETVVRVPMMSREDQYHYLLDRNLSCRVVGVPYQGNATALFILPSEGKMQ
QVENGLSEKTLRKWLKMFKKRQLELYLPKFSIEGSYQLEKVLPSLGISNVFTSHADLSGIS
NHSNIQVSEMVHKAVVEVDESGTRAAAATGTIFTF
>non-antioxidant_1373
AEIKNVILMIGDGMGPQQVGLLETYANQAPNSIYKGNKTAIYQLAQEGVIGSSLTHPEDAI
VVDSACSATMLATGIYSSSEVIGIDSQGNHVETVLEKAKKAGKATGLVSDTRLTHATPASF
AAHQPHRSLENQIASDMLATGADVMLSGGLRHWIPKSTNDKGETYKQLEKLTQGDVYLK
SKRKDDRNLLTEAEKDGYQLAFNRNMLDDAKGDKLLGLFAYSGMDDGIAYSNKKKSGE
RTQPSLKEMTQKALNILSKDEDGFFLMVEGGQIDWAGHSNDAGTMLHELLKFDEAIQTV
YEWAKDREDTIVIVTADHETGSFGFSYSSNDLPKPQKRSGEAFADRDYAPNFNFGAFDILD
GLYNQKQSYYGMISEFQKLDKSLQTPEKLAEIVNKNSEFPITAEQAKNVLASKPNPYRLAQ
HKYLSAEEVPAINDFDAFFPYNDRGNLLAREQATGQNIVWGTGTHTHTPVNVFAWGPAE
KILPVSKIMHHSELGEYIKQQVN
>non-antioxidant_1374
MSLTSTATDSPLKYYDIGLNLTDPMFHGIYNGKQYHPADYVKLLERAAQRHVKNALVTGS
SIAESQSAIELVSSVKDLSPLKLYHTIGVHPCCVNEFADASQGDKASASIDNPSMDEAYNES
LYAKVISNPSFAQGKLKELYDLMNQQAKPHDTSFRSIGEIGLDYDRFHYSSKEMQKVFFEE
QLKISCLNDKLSSYPLFLHMRSACDDFVQILERFVVGFTDEKDTFQLQKLGASSSSGFYKF
HPDRKLVVHSFTGSAIDLQKLLNLSPNIFIGVNGCSLRTEENLAVVKQIPTERLLLETDAPW
CEIKRTHASFQYLAKYQEVRDFEYPAFKSVKKNKLADKLNAEELYMVKGRNEPCNMEQV
AIVVSEVKDVDLATLIDTTWKTTCKIFGEGHHHHHH
>non-antioxidant_1375
SFWEWLNAVFNKVDHDRIRDVGPDRAASEWLLRCGAMVRYHGQQRWQKDYNHLPTGP
LDKYKIQAIDATDSCIMSIGFDHMEGLQYVEKIRLCKCHYIEDGCLERLSQLENLQKSMLE

MEIISCGNVTDKGIIALHHFRNLKYLFLSDLPGVKEKEKIVQAFKTSLPSLELKLDLK
>non-antioxidant_1376
GFGCNGPWDEDDMQCHNHCKSIKGYKGGYCAKGGFVCKCY
>non-antioxidant_1377
WKDKEFQVLFVLTILTLISGTIFYSTVEGLRPIDALYFSVVTLTTVGDGNFSPQTDFGKIFTIL
YIFIGIGLVFGFIHKLAVNVQLPSILSNLVPR
>non-antioxidant_1378
GMSPQSMLTSPQHPRRTTMVISHGTLSASAEHAAHLRQLLVHIAQATRQEDGCLLYLVSED
LSQPGHFLITEHWDNLGAMHTHLALPGVTQAIDALKHLNVTDLKITAYEAGEAINIMG
>non-antioxidant_1379
GVLPVEKCNLEDSACMTSAFQQALPTFVAGLPDHGVEVMDVLDLDDFAFDLSGLQFTLK
EGKLKGLKGAVIDNVKWDLKKKNIEVDFHLDATVKGHYTAGGRILILPITGDGQMKLKL
KNIHIHLVVSYEMEKDAEGVDHVIFKKYTVTFDVKDNAQFGLTNLFNGNKELSDTMLTFL
NQNWKQVSEEFGKPVMEAAAKKIFKNIKHFLAKVPIAEIANV
>non-antioxidant_1380
AACYSSDCRVKCVAMGFSSGKCINSKCKCYK
>non-antioxidant_1381
QGDIGIDNLRNFYTKKDFVDLKDVKDNDTPIANQLQFSNESYDLISESKDFNKFSNFKGK
KLDVFGISYNGQCNTKYIYGGVTATNEYLDKSRNIPINIWINGNHKTISTNKVSTNKKFVT
AQEIDVKLRKYLQEEYNIYGHNGTKKGEEYGHKSKFYSGFNIGKVTFHLNNNDTFSYDLF
YTGDDGLPKSFLKIYEDNKTVESEKFHLDVDISYKETI
>non-antioxidant_1382
GMAKKTLILYYSWSGETKKMAEKINSEIKDSELKEVKVSEGTFDADMYKTSDIALDQIQG
NKDFPEIQLDNIDYNNYDLILIGSPVWSGYPATPIKTLLDQMKNYRGEVASFFTSAGTNHK
AYVSHFNEWADGLNVIGVARDDSEVDKWSK
>non-antioxidant_1383
SNAMRNKNMSTIYQTSATASAGRNGVVSTEDKLLELNLSYPKEMGGSGTATNPEQLFAVG
YAACFSNAILHVAREAKVALKEAPVTATVGIGPNGQGGFALSVALAAHIALEDEQARQLVT
VAHQVCPYSNAVRGNIDVQVSVNGLAL
>non-antioxidant_1384
MVDFYYLPGSSPCRSVIMTAKAVGVELNKKLLNLQAGEHLKPEFLKINPQHTIPTLVDNGF
ALWESRAIQVYLVEKYGKTDSLYPKCPKKRAVINQRLYFDMGTLYQSFANYYYPQVFAKA
PADPEAFKKIEAAFEFLNTFLEGQDYAAGDSLTVADIALVATVSTFEVAKFEISKYANVNRW
YENAKKVTPGWEENWAGCLEFKKYFE
>non-antioxidant_1385
MPMISCDMRYGRTDEQKRALSAGLLRVISEATGEPRENIFFVIREGSGINFVQHGEHLPDY
VPGNANDKALIAKLK
>non-antioxidant_1386
GGNLEEMNIDPDNATQTHPKLLLTQICMNAFKRGTDGMYATKKVIQADGESADQYYKWT
RGSFGYYDNLRNVQKMGEEAERVNAPVYTALTKFFRAYYFYELTLRFGDIPYSQALKGEK
EEIYTPEYDAQEDVFAGILQELREADEILANDASVIDGDIIYNGNSTQWRKLINSFRLKVLM
TLSNHTTVGNINIASEFKNIATNSPLMNSLADNGQLVYLDQQGNRYPQFNAQWSGYYMD
DTFIQRMRERRDPRLFIFSAQTNKGKTEGKPIDDFSSYEGGDPAAPYSDAIIKVSEGTISPIN
DRFRTDPIVEPTMLMGYAELQQILAEAVVRGWISGNAQTYYEKGIRASFSFYETHAKDYA

GYLNENAVAQYLKEPLVDFTQASGTEEQIERIIMQKYLVTFYQGNWDSFYEQLRTGYPDF
RRPAGTEIPKRWMYPQGEYDNNGTNVETAITRQFGAGNDKINQATWWQKKS
>non-antioxidant_1387
MLKKLKNFILFSSIFSPIAFAISCSNTGVVKQEDVSVSQGQWDKSITFGVSEAWLNKKKGG
EKVNKEVINTFLENFKKEFNKLKNANDKTKNFDDVDFKVTPIQDFTVLLNNLSTDNPELD
FGINASGKLVEFLKNNPGIITPALETTTNSFVFDKEKDKFYVDGTDSDPLVKIAKEINKIFVE
TPYASWTDENHKWNGNVYQSVYDPTVQANFYRGMIWIKGNDETLAKIKKAWNDKDWN
TFRNFGILHGKDNSSSKFKLEETILKNHFQNKFTTLNEDRSAHPNAYKQKSADTLGTLDDF
HIAFSEEGSFAWTHNKSATKPFETKANEKMEALIVTNPIPYDVGVFRKSVNQLEQNLIVQT
FINLAKNKQDTYGPLLGYNGYKKIDNFQKEIVEVYEKAIK
>non-antioxidant_1388
MPIATPEVYAEMLGQAKQNSYAFPAINCTSSETVNAAIKGFADAGSDGIIQFSTGGAEFGSG
LGVKDMVTGAVALAEFTHVIAAKYPVNVALHTDHCPKDKLDSYVRPLLAISAQRVSKGG
NPLFQSHMWDGSAVPIDENLAIAQELLKAAAAAKIILEIEIGVVGGEEDGVANEINEKLYTS
PEDFEKTIEALGAGEHGKYLLAATFGNVHGVYKPGNVKLRPDILAQGQQVAAAKLGLPA
DAKPFDFVFHGGSGSLKSEIEEALRYGVVKMNVDTDTQYAFTRPIAGHMFTNYDGVLKV
DGEVGVKKVYDPRSYLKKAEASMSQRVVQACNDLHCAGKSLTHHHHHH
>non-antioxidant_1389
GPLGSPEFQSKPTPVKPNYALKFTLAGHTKAVSSVKFSPNGEWLASSSADKLIKIWGAYDG
KFEKTISGHKLGISDVAWSSDSNLLVSASDDKTLKIWDVSSGKCLKTLKGHSNYVFCCNF
NPQSNLIVSGSFDESVRIWDVKTGKCLKTLPAHSDPVSAVHFNRDGSLIVSSSYDGLCRIW
DTASGQCLKTLIDDDNPPVSFVKFSPNGKYILAATLDNTLKLWDYSKGKCLKTYTGHKNE
KYCIFANFSVTGGKWIVSGSEDNLVYIWNLQTKEIVQKLQGHTDVVISTACHPTENIIASA
ALENDKTIKLWKSDC
>non-antioxidant_1390
GMAPDQQVPATALGKSSRISLDGRRSERSVILADGSMHSLTLLHPGVYTLSSEVAETIRVLS
GMAYYHAEGANDVQELHAGDSMVIPANQSYRLEVMEPLDYLLSS
>non-antioxidant_1391
SLSRTVHHQQTAEITQQAADFIRYMNAINDYLYQHPERRAAGGQLTSAQLGLPATKNVSH
LISQQRVFVWAKEKPGLMGALLEQSGDSALLARVENGRLLDTHGRRISITLPAVIPDQVIIW
MN
>non-antioxidant_1392
ALFGTKDTTTAHSDYEIILEGGSSSWGQVKGRAKVNVPAAIPLLPTDCNIRIDAKPLDAQK
GVVRFTTKIESVVDSVKNTLNVEVDIANETKDRRIAVGEGSLSVGDFSHSFSFEGSVVNMY
YYRSDAVRRNIPNPIYMQGRQFHDILMKVPLDNNDLVDTWEGFQQSISGGGANFGDWIRE
FWFIGPAFAAINEGGQRISPIVVNSSNVEGGEKGPVGVTRWKFSHAGSGVVDSISRWTELF
PVEQLNKPASIEGGFRSDSQGIEVKVDGNLPGVSRDAGGGLRRILNHPLIPLVHHGMVGKF
NDFTVDTQLKIVLPKGYKIRYAAPQFRSQNLEEYRWSGGAYARWVEHVCKGGTGQFEVL
YAQ
>non-antioxidant_1393
MRGSHHHHHHGSAKNVVLDHDGNLDDFVAMVLLASNTEKVRLIGALCTDADCFVENGF
NVTGKIMCLMHNNMNLPLFPIGKSAATAVNPFPKEWRCLAKNMDDMPILNIPENVELWD
KIKAENEKYEGQQLLADLVMNSEEKVTICVTGPLSNVAWCIDKYGEKFTSKVEECVIMGG
AVDVRGNVFLPSTDGTAEWNIYWDPASAKTVFGCPGLRRIMFSLDSTNTVPVRSPYVQRF

GEQTNFLLSILVGTMWAMCTHCELLRDGDGYYAWDALTAAYVVDQKVANVDPVPIDVV
VDKQPNEGATVRTDAENYPLTFVARNPEAEFFLDMLLRSARAC
>non-antioxidant_1394
MGSSHHHHHHSSGENLYFQGSHMAVITPQGVTNWTYQELEATHQALTREGYVFVGYHGT
NHVAAQTIVNRIAPVPRGNNTENEEKWGGLYVATHAEVAHGYARIKEGTGEYGLPTRAER
DARGVMLRVYIPRASLERFYRTNTPLENAEEHITQVIGHSLPLRNEAFTGPESAGGEDETVI
GWDMAIHAVAIPSTIPGNAYEELAIDEEAVAKEQSISTKPPYKERKDEL
>non-antioxidant_1395
SNAKNRLGTKALNFTYTLDSGVKGTLYQFPAEYTLLFINNPGCHACAEMIEGLKASPVING
FTAAKKLKVLSIYPDEELDEWKKHRNDFAKEWTNGYDKELVIKNKNLYDLRAIPTLYLLD
KNKTVLLKDATLQKVEQYLAE
>non-antioxidant_1396
GSGMRFYVKDHRNKAMINLHIQKDNPKIVHAFDMEDLGDKAVYCRCWRSKKFPFCDGA
HTKHNEETGDNVGPLIIKKKET
>non-antioxidant_1397
GAMELSFGARAELPRIHPVASKLLRLMQKKETNLCLSADVSLARELLQLADALGPSICML
KTHVDILNDFTLDVMKELITLAKCHEFLIFEDRAFADIGNTVKKQYEGGIFKIASWADLVN
AHVVPGSGVVKGLQEVGLPLHRGCLLIAEMSSTGSLATGDYTRAAVRMAEEHSEFVVGFI
SGSRVSMKPEFLHLTPGVQLEAGGDNLGQQYNSPQEVIGKRGSDIIIVGRGIISAADRLEAA
EMYRKAAWEAYLSRLGV
>non-antioxidant_1398
MSLSSPNILLTRIDNRLVHGQVGVTWTSTIGANLLVVVDDVVANDDIQQKLMGITAETYGF
GIRFFTIEKTINVIGKAAPHQKIFLICRTPQTVRKLVEGGIDLKDVNVGNMHFSEGKKQISS
KVYVDDQDLTDLRFIKQRGVNVFIQDVPGDQKEQIPDEGHHHHHH
>non-antioxidant_1399
MAHHHHHHMGTLEAQTQGPGSMDFRIGQGYDVHQLVPGRPLIIGGVTIPYERGLLGHSD
ADVLLHAITDALFGAAALGDIGRHFSDTDPRFKGADSRALLRECASRVAQAGFAIRNVDS
TIIAQAPKLAPHIDAMRANIAADLDLPLDRVNVKAKTNEKLGYLGRGEGIEAQAAALVVR
EAAA
>non-antioxidant_1400
GMETTHYSIAQHFSSGDFPAVYACFNDIIEWNIIGNQVVKGKADVIDFCNKMLPEMKGAVL
TNDNVIQNENQIVIEGKCRYFDAEGKEAFVSYCDIYRFENDTIKTITSYCI
>non-antioxidant_1401
MHYQPKQDLLNDRIILVTGASDGIGREAAMTYARYGATVILLGRNEEKLRQVASHINEETG
RQPQWFILDLLTCTSENCQQLAQRIAVNYPRLDGVLHNAGLLGDVCPMSEQNPQVWQDV
MQVNVNATFMLTQALLPLLLKSDAGSLVFTSSSVGRQGRANWGAYAASKFATEGMMQV
LADEYQQRLRVNCINPGGTRTAMRASAFPTEDPQKLKTPADIMPLYLWLMGDDSRRKTG
MTFDAQPGRKPGISQ
>non-antioxidant_1402
GEFKGLDSKTFLSEHSMDMKFTYCDDRITELIGYHPEELLGRSAYEFYHALDSENMTKSH
QNLCTKGQVVSGQYRMLAKHGGYVWLETQGTVIYNPRNLQPQCIMCVNYVLSEIEKN
>non-antioxidant_1403
MGDKLSKTDWKIVSFTTEEASGEGSNNGHAKHLIDGNIETFWHSRWQGGSDPLPYEIIID
MNHRVKIAQIELLPRGRGSNNPIKVVRFEASEDGTNWESIGQFGFTNQDAALKYYVKSST

ARYIKLVIPDGVGNGTVAAIRELDVRGTVVNLEHHHHHH
>non-antioxidant_1404
GMKTQITTRDLVLEFIHALNTENFPAAKKRLNENFTFNGPMGHREGSERYMNDMEKMKF
KYVVHKMFEEGNDVCLIYDINMNGKTIAASGLYHLEKGEITSLHVYFDPRPLFEE
>non-antioxidant_1405
MGSDKIHHHHHHMFPYKIVDDVVILMPNKELNIENAHLFKKWVFDEFLNKGYNKIFLVLS
DVESIDSFSLGVIVNILKSISSSGGFFALVSPNEKVERVLSLTNLDRIVKIYDTISEAMEEVRR
K
>non-antioxidant_1406
MGSSHHHHHHSSGRENLYFQGHMNSPTSRPLRRDPLHNALVTTNVLVVLDQLEVAAEPT
RRRLVQLLTSGEQTVNNLAAHFPASRSAISQHLRVLTEAGLVTPRKDGRFRYYRLDPQGLA
QLRALFDSFWIDELDRLVADATEEAASKGDS
>non-antioxidant_1407
KREAEAFWRQTWSGPGTTKRFPETVLARCVKYTEIHPEMRHVDCQSVWDAFKGAFISKH
PCDITEEDYQPLMKLGTQTVPCNKILLWSRIKDLAHQFTQVQRDMFTLEDTLLGYLADDL
TWCGEFDTSKINYQSCPDWRKDCSNNPVSVFWKTVSRRFAEAACDVVHVMLDGSRSKIF
DKDSTFGSVEVHNLQPEKVQTLEAWVIHGGREDSRDLCQDPTIKELESIISKRNIQFSCKNI
YRPDKFLQCVKNPEDSSCTSEI
>non-antioxidant_1408
GMSKVEQNAQHNIQQTSPNAAVQSGLQEWHRIIAEADWERLPDLLAEDVVFSNPSTFDPY
HGKGPLMVILPAVFSVLENFQYARHFSSKSGYVLEFNANMGDELLTGVDLIEFNDAGKITD
LVVMMRPASVVIDLSVEVGKRIAAAQS
>non-antioxidant_1409
MGHHHHHHENLYFQGMKKEKVEQILAEFQLQEEDLKKVMRRMQKEMDRGLRLETHEE
ASVKMLPTYVRSTPEGSEVGDFLSLDLGGTNFRVMLVKVGEGEEGQWSVKTKHQMYSIP
EDAMTGTAEMLFDYISECISDFLDKHQMKHKKLPLGFTFSFPVRHEDIDKGILLNWTKGF
KASGAEGNNVVGLLRDAIKRRGDFEMDVVAMVNDTVATMISCYYEDHQCEVGMIVGTG
CNACYMEEMQNVELVEGDEGRMCVNTEWGAFGDSGELDEFLLEYDRLVDESSANPGQQ
LYEKLIGGKYMGELVRLVLLRLVDENLLFHGEASEQLRTRGAFETRFVSQVESDTGDRKQI
YNILSTLGLRPSTTDCDIVRRACESVSTRAAHMCSAGLAGVINRMRESRSEDVMRITVGV
DGSVYKLHPSFKERFHASVRRLTPSCEITFIESEEGSGRGAALVSAVACKKACMLGQ
>non-antioxidant_1410
GAMGSRKSKAELQSEERKRIDELIESGKEEGMKIDLIDGKGRGVIATKQFSRGDFVVEYH
GDLIEITDAKKREALYAQDPSTGCYMYYFQYLSKTYCVDATRETNRLGRLINHSKCGNCQ
TKLHDIDGVPHLILIASRDIAAGEELLFDYGDRSKASIEAHPWLKH
>non-antioxidant_1411
MEFFGESWKKHLSGEFGKPYFIKLMGFVAEERKHYTVYPPPHQVFTWTQMCDIKDVKVV
ILGQDPYHGPNQAHGLCFSVQRPVPPPPSLENIYKELSTDIEDFVHPGHGDLSGWAKQGVL
LLNAVLTVRAHQANSHKERGWEQFTDAVVSWLNQNSNGLVFLLWGSYAQKKGSAIDRK
RHHVLQTAHPSPLSVYRGFFGCRHFSKTNELLQKSGKKPIDWKEL
>non-antioxidant_1412
GMGMEHKTYEADLFVWCQQQADGLRALSRSRRDLPDDLDLEHIAEEIEDMGRSELREAT
SLVRQICVRVIMAMSAPEAPDRARWRSEVVSWHNLLLDTITPGMIDRIDIGVIWRRAVSEA
KAALIEINVAPQAGLSFQAPLPADHFLDEDFDYDATVARLGPTA

>non-antioxidant_1413
MALKQISSNKCFGGLQKVFEHDSVELNCKMKFAVYLPPKAETGKCPALYWLSGLTCTEQ
NFISKSGYHQSASEHGLVVIAPDTSPRGCNIKGEDESWDFGTGAGFYVDATEDPWKTNYR
MYSYVTEELPQLINANFPVDPQRMSIFGHSMGGHGALICALKNPGKYKSVSAFAPICNPVL
CPWGKKAFSGYLGTDQSKWKAYDATHLVKSYPGSQLDILIDQGKDDQFLLDGQLLPDNFI
AACTEKKIPVVFRLQEDYDHSYYFIATFITDHIRHHAKYLNA
>non-antioxidant_1414
HMPANHFGPIPGVPVGTMWRFRVQVSESGVHRPHVAGIHGRSNDGAYSLVLAGGYEDDV
DNGNYFTYTGSGGRDLSGNKRTAGQSSDQKLTNNNRALALNCHSPINEKGAEAEDWRQG
KPVRVVRNMKGGKHSKYAPAEGNRYDGIYKVVKYWPERGKSGFLVWRYLLRRDDTEPE
PWTREGKDRTRQLGLTMQYPEGYLEALANKEKSRKR
>non-antioxidant_1415
MSTSSSDPFFNFAKSSFRSAAAQKASASSLPPLPGPDKKVPGMDIKYDVVIVGSGPIGCTYA
RELVGAGYKVAMFDIGEIDSGLKIGAHKKNTVEYQKNIDKFVNVIQGQLMSVSVPVNTLV
VDTLSPTSWQASTFFVRNGSNPEQDPLRNLSGQAVTRVVGGMSTHWGCATPRFDREQRP
LLVKDDADADDAEWDRLYTKAESYFQTGTDQFKESIRHNLVLNKLTEEYKGQRDFQQIPL
AATRRSPTFVEWSSANTVFDLQNRPNTDAPEERFNLFPAVACERVVRNALNSEIESLHIHDL
ISGDRFEIKADVYVLTAGAVHNTQLLVNSGFGQLGRPNPANPPELLPSLGSYITEQSLVFCQ
TVMSTELIDSVKSDMTIRGTPGELTYSVTYTPGASTNKHPDWWNEKVKNHMMQHQEDP
LPIPFEDPEPQVTTLFQPSHPWHTQIHRDAFSYGAVQQSIDSRLIVDWRFFGRTEPKEENKL
WFSDKITDAYNMPQPTFDFRFPAGRTSKEAEDMMTDMCVMSAKIGGFLPGSLPQFMKPG
LCLHLGGTHRMGFDEKEDNCCVNTDSRVFGFKNLFLGGCGNIPTAYGANPTLTAMSLAIK
SCEYIKQNFTPSPFTSEAQ
>non-antioxidant_1416
GPDSASRISPGQINQVRPKLPLLKILHAAGAQGEMFTVKEVMHYLGQYIMVKQLYDQQE
QHMVYCGGDLLGELLGRQSFSVKDPSPLYDMLRKNLVTLAT
>non-antioxidant_1417
GSSHHHHHHSSGLVPRGSRRRASSLSRDAERRAYQWCREYLGGAWRRVQPEELRVYPVS
GGLSNLLFRCSLPDHLPSVGEEPREVLLRLYGAILQGVDSLVLESVMFAILAERSLGPQLYG
VFPEGRLEQYIPSRPLKTQELREPVLSAAIATKMAQFHGMEMPFTKEPHWLFGTMERYLK
QIQDLPPTGLPEMNLLEMYSLKDEMGNLRKLLESTPSPVVFCHNDIQEGNILLLSEPENAD
SLMLVDFEYSSYNYRGFDIGNHFCEWVYDYTHEEWPFYKARPTDYPTQEQQLHFIRHYL
AEAKKGETLSQEEQRKLEEDLLVEVSRYALASHFFWGLWSILQASMSTIEFGYLDYAQSRF
QFYFQQKGQLTSVHSSS
>non-antioxidant_1418
SNAMENQKMQEPLVYRRILLTVDEDDNTSSERAFRYATTLAHDYDVPLGICSVLESEDINIF
DSLTPSKIQAKRKHVEDVVAEYVQLAEQRGVNQVEPLVYEGGDVDDVILEQVIPEFKPDL
LVTGADTEFPHSKIAGAIGPRLARKAPISVIVVR
>non-antioxidant_1419
GKPKRPRSAYNVYVAERFQEAKGDSPQEKLKTVKENWKNLSDSEKELYIQHAKEDETRY
HNEMKSWA
>non-antioxidant_1420
GMTESDRDQEASMREVVIVKSTPQRGKFNAFAELVGKLVSETRDFPGCLGAYLMLAPERN
EQVVMHIWETPDALEAYLTWRADRGDFLEINEYLEVEQDFKTYQLA

>non-antioxidant_1421
QAMFERFTDRARRVIVLAQEEARMLNHNYIGTEHILLGLIHEGEGVAAKALESMGISLDAV
RQEVEEIIGQGSQPTTGHIPFTPRAKKVLELSLREGLQMGHKYIGTEFLLLGLIREGEGVAA
QVLVKLGADLPRVRQQVIQLLSG
>non-antioxidant_1422
MGHHHHHHSHVAQFPTPFGGSLDTWAITVEERAKHDQQFHSLKPISGFITGDQARNFFFQS
GLPQPVLAQIWALADMNNDGRMDQVEFSIAMKLIKLKLQGYQLPSALPPVMKQQPVAISS
>non-antioxidant_1423
MQYKLILNGKTLKGVLTIEAVDAATAEKVFKQYANDLGVDGEWTYDDATKTFTVTE
>non-antioxidant_1424
RSTDTFNYATYHTLEEIYDFLDLLVAENPHLVSKIQIGNTYEGRPIYVLKFSTGGSKRPAIWI
DTGIHSREWVTQASGVWFAKKITQDYGQDAAFTAILDTLDIFLEIVTNPDGFAFTHSTNRM
WRKTRSHTAGSLCIGVDPNRNWDAGFGLSGASSNPCSETYHGKFANSEVEVKSIVDFVKD
HGNIKAFISIHSYSQLLMYPYGYKTEPVPDQDELDQLSKAAVTALASLYGTKFNYGSIIKAI
YQASGSTIDWTYSQGIKYSFTFELRDTGRYGFLLPASQIIPTAKETWLALLTIMEHTLNHP
>non-antioxidant_1425
GHMGKPDISAKDLRNIMYDHLPGFGTAFHQLVQVICKLGKDSNSLDIIHAEFQASLAEGDS
PQCALIQITKRVPIFQDAAPPVIHIRSRGDIPRACQKSLRPVPPSPKIDRGWVCVFQLQDGKT
LGLKI
>non-antioxidant_1426
ATCDDGRTTANAACCILFPILDDIQENLFDGAQCGEEVHESLRLTFHDAIGFSPTLGGGGAD
GSIIAFDTIETNFPANAGIDEIVSAQKPFVAKHNISAGDFIQFAGAVGVSNCPGGVRIPFFLGR
PDAVAASPDHLVPEPFDSVDSILARMGDAGFSPVEVVSLLASHSIAAADKVDPSIPGTPFDS
TPGVFDSQFFIETQLKGRLFPGTADNKGEAQSPLQGEIRLQSDHLLARDPQTACEWQSMV
NNQPKIQNRFAATMSKMALLGQDKTKLIDCSDVIPTPPALVGAAHLPAGFSLSDVEQACAA
TPFPALTADPGPVTSVPPVPGS
>non-antioxidant_1427
EPGENLKPVDAMQCFDCHTQIEDMHTVGKHATVNCVHCHDATEHVETASSRRMGERPV
TRMDLEACATCHTAQFNSFVEVRHESHPRLEKATPTSRSPMFDKLIAGHGFAFEHAEPRSH
AFMLVDHFVVDRAYGGRFQFKNWQKVTDGMGAVRGAWTVLTDADPESSDQRRFLSQTA
TAANPVCLNCKTQDHILDWAYMGDEHEAAKWSRTSEVVEFARDLNHPLNCFMCHDPHS
AGPRVVRDGLINAVVDRGLGTYPHDPVKSEQQGMTKVTFQRGREDFRAIGLLDTADSNV
MCAQCHVEYNCNPGYQLSDGSRVGMDDRRANHFFWANVFDYKEAAQEIDFFDFRHATT
GAALPKLQHPEAETFWGSVHERNGVACADCHMPKVQLENGKVYTSHSQRTPRDMMGQ
ACLNCHAEWTEDQALYAIDYIKNYTHGKIVKSEYWLAKMIDLFPVAKRAGVSEDVLNQA
RELHYDAHLYWEWWTAENSVGFHNPDQARESLMTSISKSKEAVSLLNDAIDAQVASR
>non-antioxidant_1428
GAGAMSSRKKPSRRTRVLVGGAALAVLGAGVVGTVAANAADTTEATPAAAPVAARGGEL
TQSTHLTLEAATKAARAAVEAAEKDGRHVSVAVVDRNGNTLVTLRGDGAGPQSYESAER
KAFTAVSWNAPTSELAKRLAQAPTLKDIPGTLFLAGGTPVTAKGAPVAGIGVAGAPSGDL
DEQYARAGAAVLGH
>non-antioxidant_1429
MVAAPPAVGAAMPSLDFDTSVFNKEKVSLAGHEEYIVRGGRNLFPLLPEAFKGIKQIGVIG
WGSQGPAQAQNLRDSLAEAKSDIVVKIGLRKGSKSFDEARAAGFTEESGTLGDIWETVSG

SDLVLLLISDAAQADNYEKIFSHMKPNSILGLSHGFLLGHLQSAGLDFPKNISVIAVCPKGM
GPSVRRLYVQGKEINGAGINSSFAVHQDVDGRATDVALGWSVALGSPFTFATTLEQEYKSD
IFGERGILLGAVHGIVEALFRRYTEQGMDEEMAYKNTVEGITGIISKTISKKGMLEVYNSLT
EEGKKEFNKAYSASFYPCMDILYECYEDVASGSEIRSVVLAGRRFYEKEGLPAFPMGNIDQ
TRMWKVGEKVRSTRPENDLGPLHPFTAGVYYVALMMAQIEVLRKKGHSYSEIINESVIESV
DSLNPFMHARGVAFMVDNCSTTARLGSRKWAPRFDYILTQQAFVTVDKDAPINQDLISNF
MSDPVHGAIEVCAELRPTVDISVPANADFVRPELRQSS
>non-antioxidant_1430
YPMNINDALTSILASKKYRALCPDTVRRILTEEWGRHKSPKQTVEAARTRLHGICGAYVTP
ESLKAAAAALSAGDVKKALSLHASTKERLAELDTLYDFIFSAETPRRVLDIACGLNPLALY
ERGIASVWGCDIHQGLGDVITPFAREKDWDFTFALQDVLCAPPAEAGDLALIFKLLPLLER
EQAGSAMALLQSLNTPRMAVSFPTRSLGGRGKGMEANYAAWFEGGLPAEFEIEDKKTIGT
ELIYLIKKNG
>non-antioxidant_1431
GSHMRDVVSFEQPEFSVSRGDQVARIPVIRRVLDGGKSQVSYRTQDGTAQGNRDYIPVEG
ELLFQPGEAWKELQVKLLELQEVDSLLRGRQVRRFHVQLSNPKFGAHLGQPHSTTIIIRDP
DE
>non-antioxidant_1432
GHMSETNTIFKLEGVSVLSPLRKKLDLVFYLSNVDGSPVITLLKGNDRELSIYQLNKNIKM
ASFLPVPEKPNLIYLFMTYTSCEDNKFSEPVVMTLNKENTLNQFKKLGLLDSNVTDFEKC
VEYIRKQAILTGFKISNPFVNSTLVDTDAEKINSFHLQCHRGTKEGTLYFLPDHIIFGFKKPIL
LLDASDIESITYSSITRLTFNASLVTKDGEKYEFSMIDQTEYAKIDDYVKRK
>non-antioxidant_1433
MQNGYTYEDYQDTAKWLLSHTEQRPQVAVICGSGLGGLVNKLTQAQTFDYSEIPNFPEST
VPGHAGRLVFGILNGRACVMMQGRFHMYEGYPFWKVTFPVRVFRLLGVETLVVTNAAG
GLNPNFEVGDIMLIRDHINLPGFSGENPLRGPNEERFGVRFPAMSDAYDRDMRQKAHSTW
KQMGEQRELQEGTYVMLGGPNFETVAECRLLRNLGADAVGMSTVPEVIVARHCGLRVFG
FSLITNKVIMDYESQGKANHEEVLEAGKQAAQKLEQFVSLLMASIPV
>non-antioxidant_1434
MSKLASPQSVRALLERHGLFADKRFGQNFLVSEAHLRRIVEAARPFTGPVFEVGPGLGALT
RALLEAGAEVTAIEKDLRLRPVLEETLSGLPVRLVFQDALLYPWEEVPQGSLLVANLPYHI
ATPLVTRLLKTGRFARLVFLVQKEVAERMTARPKTPAYGVLTLRVAHHAVAERLFDLPPGA
FFPPPKVWSSLVRLTPTGALDDPGLFRLVEAAFGKRRKTLLNALAAAGYPKARVEEALRA
LGLPPRVRAEELDLEAFRRLREGLEGAV
>non-antioxidant_1435
GAHMDCHLSDMLQQLHSVNASKPSERGLVRQEEAEDPACIPIFWVSKWVDYSDKYGLGY
QLCDNSVGVLFNDSTRLILYNDGDSLQYIERDGTESYLTVSSHPNSLMKKITLLKYFRNYM
SEHLLKAGANITPREGDELARLPYLRTWFRTRSAIILHLSNGSVQINFFQDHTKLILCPLMA
AVTYIDEKRDFRTYRLSLLEEYGCCKELASRLRYARTMVDKLLSSRSASNRLKAS
>non-antioxidant_1436
MAKQLQARRLDGIDYNPWVEFVKLASEHDVVNLGQGFPDFPPPDFAVEAFQHAVSGDFM
LNQYTKTFGYPPLTKILASFFGELLGQEIDPLRNVLVTVGGYGALFTAFQALVDEGDEVIIIE
PFFDCYEPMTMMAGGRPVFVSLKPGPIQNGELGSSSNWQLDPMELAGKFTSRTKALVLN
TPNNPLGKVFSREELELVASLCQQHDVVCITDEVYQWMVYDGHQHISIASLPGMWERTLT

IGSAGKTFSATGWKVGWVLGPDHIMKHLRTVHQNSVFHCPTQSQAAVAESFEREQLLFRQ
PSSYFVQFPQAMQRCRDHMIRSLQSVGLKPIIPQGSYFLITDISDFKRKMPDLPGAVDEPYD
RRFVKWMIKNKGLVAIPVSIFYSVPHQKHFDHYIRFCFVKDEATLQAMDEKLRKWKVEL
>non-antioxidant_1437
GAMVMRLGDAAELCYNLTSSYLQIAAESDSIIAQTQRAINTTKSILINETFPKWSPLNGEIS
FSYNGGKDCQVLLLLYLSCLWEYYIVKLSQSQFDGKFHRFPLTKLPTVFIDHDDTFKTLEN
FIEETSLRYSLSLYESDRDKCETMAEAFETFLQVFPETKAIVIGIRHTDPFGEHLKPIQKTDA
NWPDFYRLQPLLHWNLANIWSFLLYSNEPICELYRYGFTSLGNVEETLPNPHLRKDKNSTP
LKLNFEWEIENRYKHNEVTKAEPIPIADEDLVKIENLHEDYYPGWYLVDDKLERAGRIKK
K
>non-antioxidant_1438
MGHHHHHHHHHHMSKQQIGVVGMAVMGRNLALNIESRGYTVSIFNRSREKTEEVIAENP
GKKLVPYYTVKEFVESLETPRRILLMVKAGAGTDAAIDSLKPYLDKGDIIIDGGNTFFQDTI
RRNRELSAEGFNFIGTGVSGGEEGALKGPSIMPGGQKEAYELVAPILTKIAAVAEDGEPCVT
YIGADGAGHYVKMVHNGIEYGDMQLIAEAYSLLKGGLNLTNEELAQTFTEWNNGELSSY
LIDITKDIFTKKDEDGNYLVDVILDEAANKGTGKWTSQSALDLGEPLSLITESVFARYISSL
KDQRVAASKVLSGPQAQPAGDKAEFIEKVRRALYLGKIVSYAQGFSQLRAASEEYNWDLN
YGEIAKIFRAGCIIRAQFLQKITDAYAENPQIANLLLAPYFKQIADDYQQALRDVVAYAVQI
GIPVPTFSAAVAYYDSYRAAVLPANLIQAQRDYFGAHTYKRIDKEGVFHTEWLD
>non-antioxidant_1439
GSLTTISSILSLKREKPDNLAIILQIDFTKLKEEDSLIVVYNSLKALTIKFARLQFCFVDRNNY
VLDYGSVLHKIDSLDSISNLKSKSSSTQFSPIWLKNTLYPENIHEHLGIVAVSNSNMEAKKSI
LFQDYRCFTSFGRRRPNELKIKVGYLNVDYSDKIDELVEASSWTFVLETLCYSFGLSFDEH
DDDDEEDN
>non-antioxidant_1440
MNHKAFAKFPSSASISPNPFTVSIPDEQLDDLKTLVRLSKIAPPTYESLQADGRFGITSEWLT
TMREKWLSEFDWRPFEARLNSFPQFTTEIEGLTIHFAALFSEREDAVPIALLHGWPGSFVEF
YPILQLFREEYTPETLPFHLVVPSLPGYTFSSGPPLDKDFGLMDNARVVDQLMKDLGFGSG
YIIQGGDIGSFVGRLLGVGFDACKAVHLNFCNMSAPPEGPSIESLSAAEKEGIARMEKFMT
DGYAYAMEHSTRPSTIGHVLSSSPIALLAWIGEKYLQWVDKPLPSETILEMVSLYWLTESFP
RAIHTYREWVPTASAPNGATPYQKELYIHKPFGFSFFPKDLVPVPRSWIATTGNLVFFRDHA
EGGHFAALERPRELKTDLTAFVEQVWQKGRSHHHHHH
>non-antioxidant_1441
GMTAPTLSRAAMEKVIRTYYDGCNEADEAKMIACFVPEAVHYFPAGMYGGAFRGAAQIA
HRWRTAVETLGSYWTIDALVIDAETAEAAIEWTHFKTNQDKVLRGAECVEFDRASGLIREI
RAFYASPQAEGIARLELGDFDYAGRGYRVTSPRKPA
>non-antioxidant_1442
MSLTLTLTGTGGAQGVPAWGCECAACARARRSPQYRRQPCSGVVKFNDAITLIDAGLHDL
ADRWSPGSFQQFLLTHYHMDHVQGLFPLRWGVGDPIPVYGPPDEQGCDDLFKHPGLLDF
SHTVEPFVVFDLQGLQVTPLPLNHSKLTFGYLLETAHSRVAWLSDTAGLPEKTLKFLRNNQ
PQVMVMDCSHPPRADAPRNHCDLNTVLALNQVIRSPRVILTHISHQFDAWLMENALPSGF
EVGFDGMEIGVAHHHHHH
>non-antioxidant_1443
AGPWADIMQGPSESFVDFANRLIKAVEGSDLPPSARAPVIIDCFRQKSQPDIQQLIRTAPSTL

TTPGEIIKYVLDRQ
>non-antioxidant_1444
KVDLQSLPTRAYLDQTVVPILLQGMAVLAKERPPNPIEFLASYLLKNKAQFEDRN
>non-antioxidant_1445
GSNRSLIVTTILEEPYVLFKKSDKPLYGNDRFEGYCIDLLRELSTHLGFTYEIRLVEDGKYG
AQDDVNGQWNGMVRELIDHKADLAVAPLAITYVREEVIDFSKPFMTLGISILYRKGTPIDS
ADDLAKQTKIEYGAVEDGATMTFFKRSKISTYDKMWAFMSSRRQSVLVKSNEEGIQRVLT
SDYAFLMESTTIEFVTQRNCNLTQIGGLIDSKGYGVGTPMGSPYRDKITLAILKLQEQGKL
HMMKEKWWRGNGCPE
>non-antioxidant_1446
PSVYDAAAQLTADVKKDLRDSWKVIGSDKKGNGVALMTTLFADNQETIGYFKRLGDVSQ
GMANDKLRGHSITLMYALQNFIDQLDNPDDLVCVVEKFAVNHITRKISAAEFGKINGPIKK
VLASKNFGDKYANAWAKLVAVVQAAL
>non-antioxidant_1447
SNAMANFEDFLTLDLRIGTVTHAEEFKEARVPAIRLEIDFGELGMKQSSAQITKRYNPEDLI
GQQIVAVVNFPPKRVAGFKSEVLVLGGVPEAGDVVLLQPNMELPNGTKIS
>non-antioxidant_1448
MERVNVVGAGLAGSEAAWTLLRLGVPVRLFEMRPKRMTPAHGTDRFAEIVCSNSLGGEG
ETNAKGLLQAEMRRAGSLVMEAADLARVPAGGALAVDREEFSGYITERLTGHPLLEVVRE
EVREIPPGITVLATGPLTSEALAEALKRRFGDHFLAYYDAASPIVLYESIDLTKCFRAGRYG
QSADYLNCPMTEEEYRRFHQALLEAQRHTPHDWEKLEFFEACVPVEELARRGYQTLLFG
PMKPVGLVDPRTGKEPFAVVQLRQEDKAGRMWSLVGFQTGLKWPEQKRLIQMIPGLENA
EIVRYGVMHRNTYLNAPRLLGETLEFREAEGLYAAGVLAGVEGYLESAATGFLAGLNAA
RKALGLPPVAPPEESMLGGLVRYLATANPEGFQPMYANWGLVPPVEGRMGKKEKRQAM
YRRGLEAFSAWLSGLNPPLPRPEAALV
>non-antioxidant_1449
SSTFSASDFNSERYSSSRPSYPSDFYKMIDEYHDGERKLLVDVGCGPGTATLQMAQELKPF
EQIIGSDLSATMIKTAEVIKEGSPDTYKNVSFKISSSDDFKFLGADSVDKQKIDMITAVECAH
WFDFEKFQRSAYANLRKDGTIAIWGYADPIFPDYPEFDDLMIEVPYGKQGLGPYWEQPGR
SRLRNMLKDSHLDPELFHDIQVSYFCAEDVRDKVKLHQHTKKPLLIRKQVTLVEFADYVR
TWSAYHQWKQDPKNKDKEDVADWFIKESLRRRPELSTNTKIEVVWNTFYKLGKRV
>non-antioxidant_1450
MDINGGGATLPQALYQTSGVLTAGFAQYIGVGSGNGKAAFLNNDYTKFQAGVTNKNVH
WAGSDSKLSATELSTYASAKQPTWGKLIQVPSVGTSVAIPFNKSGSAAVNLSVQELCGVFS
GRINTWDGISGSGRTGPIVVVYRSESSGTTELFTRFLNAKCNAETGNFAVTTTFGTSFSGGL
PAGAVAATGSQGVMTALAAGDGRITYMSPDFAAPTLAGLDDATKVARVGKNVATNTQGV
SPAAANVSAAIGAVPVPAAADRSNPDAWVPVFGPDNTAGVQPYPTSGYPILGFTNLIFSQC
YADATQTTQVRDFFTKHYGASNNNDAAITANAFVPLPTAWKATVRASFLTASNALSIGNT
NVCNGIGRPLLEAAHHHHHH
>non-antioxidant_1451
GSSANDWQCKTCSNVNWARRSECNMCNTPKYAK
>non-antioxidant_1452
AKHYKNNPSLITFLCKNCSVLACSGEDIHVIEKMHHVNMTPEFKELYIVRENKALQKKCA
DYQINGEIICKCGQAWGTMMVHKGLDLPCLKIRNFVVVFKNNSTKKQYKKWVELPITFP

NLDYSELEHHHHHH
>non-antioxidant_1453
MATASHNIDDILQLKDDTGVITVTADNYPLLSRGVPGYFNILYITMRGTNSNGMSCQLCHD
FEKTYHAVADVIRSQAPQSLNLFFTVDVNEVPQLVKDLKLQNVPHLVVYPPAESNKQSQF
EWKTSPFYQYSLVPENAENTLQFGDFLAKILNISITVPQAFNVQEEFHHHHHHHHHH
>non-antioxidant_1454
SNAMKPENKIPVLTRLSDEMTAVVNFQQPGLPPWPADGDIETQRQYYLLERRFWNADAPS
MTTRTCAVPTPYGDVTTRLYSPQPTSQATLYYLHGGGFILGNLDTHDRIMRLLARYTGCTV
IGIDYSLSPQARYPQAIEETVAVCSYFSQHADEYSLNVEKIGFAGDSAGAMLALASALWLR
DKHIRCGNVIAILLWYGLYGLQDSVSRRLFGGAWDGLTREDLDMYEKAYLRNDEDRESP
WYCLFNNDLTRDVPPCFIASAEFDPLIDDSRLLHQTLQAHQQPCEYKMYPGTLHAFLHYS
RMMTIADDALQDGARFFMARMKTPR
>non-antioxidant_1455
GMKVLPVKQYLIMENYNPDTIFNGIVKINSNEKTFDDEILAQIGGALHDIDESWELLLSFA
NTIRSNWEIKTPAYDIVRLIVKKLPYSSDIKDYIEEGLGNKNITLTMLTVRILVNCFNNENW
GVKLLESNQVYKSIFETIDTEFSQASAKQSQNLAIAVSTLIFNYSALVTKGNSDLELLPIVAD
AINTKYGPLEEYQECEEAAYRLTVAYGNLATVEPTLRQFANSVTWLANIKRSYGNVPRFK
DIFDDLS
>non-antioxidant_1456
SLEDYSVVNRFESHGGGWGYSAHSVEAIRFSADTDILLGGLGLFGGRGEYTAKIKLFELGP
DGGDHETDGDLLAETDVLAYDCAAREKYAMMFDEPVLLQAGWWYVAWARVSGPSSDC
GSHGQASITTDDGVIFQFKSSKKSNNGTDVNAGQIPQLLYRLPTSD
>non-antioxidant_1457
MAMHPRKDWYELTRATNWTPSYVTEEQLFPERMSGHMGIPLEKWESYDEPYKTSYPEY
VSIQREKDAGAYSVKAALERAKIYENSDPGWISTLKSHYGAIAVGEYAAVTGEGRMARFS
KAPGNRNMATFGMMDELRHGQLQLFFPHEYCKKDRQFDWAWRAYHSNEWAAIAAKHF
FDDIITGRDAISVAIMLTFSFETGFANMQFLGLAADAAEAGDYTFANLISSIQTDESRHAQQ
GGPALQLLIENGKREEAQKKVDMAIWRAWRLFAVLTGPVMDYYTPLEDRSQSFKEFMYE
WIIGQFERSLIDLGLDKPWYWDLFLKDIDELHHSYHMGVWYWRTTAWWNPAAGVTPEE
RDWLEEKYPGWNKRWGRCWDVITENVLNDRMDLVSPETLPSVCNMSQIPLVGVPGDDW
NIEVFSLEHNGRLYHFGSEVDRWVFQQDPVQYQNHMNIVDRFLAGQIQPMTLEGALKYM
GFQSIEEMGKDAHDFAWADKCKPAMKKSA
>non-antioxidant_1458
MSFESKKPMRTWSHLAEMRKKPSEYDIVSRKLHYSTNNPDSPWELSPDSPMNLWYKQYR
NASPLKHDNWDAFTDPDQLVYRTYNLMQDGQESYVQSLFDQFNEREHDQMVREGWEH
TMARCYSPLRYLFHCLQMSSAYVQQMAPASTISNCCILQTADSLRWLTHTAYRTHELSLTY
PDAGLGEHERELWEKEPGWQGLRELMEKQLTAFDWGEAFVSLNLVVKPMIVESIFKPLQ
QQAWENNDTLLPLLIDSQLKDAERHSRWSKALVKHALENPDNHAVIEGWIEKWRPLADR
AAEAYLSMLSSDILHAQYLERSTSLRASILTV
>non-antioxidant_1459
MSAFPVHAAFEKDFLVQLVVVDLNDSMDQVAEKVAYHCVNRRVAPREGVMRVRKHRST
ELFPRDMTIAESGLNPTEVIDVVFEE
>non-antioxidant_1460
MSTLADQALHNNNVGPIIRAGDLVEPVIETAEIDNPGKEITVEDRRAYVRIAAEGELILTRK

TLEEQLGRPFNMQELEINLASFAGQIQADEDQIRFYFDKTM
>non-antioxidant_1461
MNNKTEQGNDAVSGQPSIKGQPVLGKDDAPVTVVEFGDYKCPSCKVFNSDIFPKIQKDFI
DKGDVKFSFVNVMFHGKGSRLAALASEEVWKEDPDSFWDFHEKLFEKQPDTEQEWVTP
GLLGDLAKSTTKIKPETLKENLDKETFASQVEKDSDLNQKMNIQATPTIYVNDKVIKNFAD
YDEIKETIEKELKGKLEHHHHHH
>non-antioxidant_1462
SNAMHILVTGFAPFDNQNINPSWEAVTQLEDIIGTHTIDKLKLPTSFKKVDNIINKTLASNH
YDVVLAIGQAGGRNAITPERVAINIDDARIPDNDDFQPIDQAIHLDGAPAYFSNLPVKAMT
QSIINQGLPGALSNSAGTFVCNHTLYHLGYLQDKHYPHLRFGFIHVPYIPEQVIGKPDTPSM
PLEKIVAGLTAAIEAISNDEDLHLALGTTE
>non-antioxidant_1463
GMGGAALPDNGWPADRIDTESAHSARIYDYIIGGKDYYPADKEAGDAMSREWPALPVH
MRANRDWMNRAVAHLAKEAGIRQFLDIGTGIPTSPNLHEIAQSVAPESRVVYVDNDPIVLT
LSQGLLASTPEGRTAYVEADMLDPASILDAPELRDTLDLTRPVALTVIAIVHFVLDEDDAVG
IVRRLLEPLPSGSYLAMSIGTAEFAPQEVGRVAREYAARNMPMRLRTHAEAEEFFEGLELV
EPGIVQVHKWHPDAATADGIRDEDIAMYGAVARKP
>non-antioxidant_1464
MSQNLFNVEDYRKLAQKRLPKMVYDYLEGGAEDEYGVKHNRDVFQQWRFKPKRLVDV
SRRSLQAEVLGKRQSMPLLIGPTALNGALWPKGDLALARAATKAGIPFVLSTASNMSIEDL
ARQCDGDLWFQLYVIHREIAQGMVLKALHTGYTTLVLTTDVAVNGYRERDLHNRFKIPPF
LTLKNFEGIDLGKMDKANLEMQAALMSRQMDASFNWEALRWLRDLWPHKLLVKGLLS
AEDADRCIAEGADGVILSNHGGRQLDCAISPMEVLAQSVAKTGKPVLIDSGFRRGSDIVK
ALALGAEAVLLGRATLYGLAARGETGVDEVLTLLKADIDRTLAQIGCPDITSLSPDYLQNE
GVTNTAPVDHLIGKGTHAHHHHHH
>non-antioxidant_1465
SNAMARKKNTSDQSRSQAANTPIAGTYEGEYSVIELEADSYTTDGWLISINGVPSSHIVLG
QPQALEFEYMRWIATGARAFIDAHQDASKLRITHLGGGACTMARYFADVYPQSRNTVVE
LDAELARLSREWFDIPRAPRVKIRVDDARMVAESFTPASRDVIIRDVFAGAITPQNFTTVEF
FEHCHRGLAPGGLYVANCGDHSDLRGAKSELAGMMEVFEHVAVIADPPMLKGRRYGNIIL
MGSDTEFFSSNSTEASAITRELLGGGVPAQYKDESWVRKFASGAQARHDGVSTLQMPSDT
PQHPAETPEHSNTQP
>non-antioxidant_1466
GAQSCVWYGECGIAYGDKRYNCEYSGPPKPLPKDGYDLVQELCPGFFFGQVSLCCDVRQ
LQTLKDNLQLPLQFLSRCPSCFYNLLNLFCELTCSPRQSQFLQVTATEDYVDPVTNQTKTN
VKELQYYVGQSFANAMYNACRDVEAPSSNDKALGLLCGKDADACQATNWIEYMFNKD
NGQAPFTITPVFSDFPVHGMEPMNNATKGCDESVDEVTAPCSCQDCSIVCGPKPQ
>non-antioxidant_1467
MSLQASGNTDQAALESAQARLAALSILVGAVGATGPGVMITIDDPGPGVAPEVMIDVINEL
RAAGAEAIQINDAHRSVRVGVDTWVVGVPGSLTVDTKVLSPPYSILAIGDPPTLAAAMNIP
GGAQDGVKRVGGRMVVQQADRVDVTALRQPKQHQYAQPVKEGHHHHHH
>non-antioxidant_1468
YTGFTPERYNKIQFGMDRTLVWQLAGADQSCSDQVERIICYNNPDHYGPQGHFFFNAAD
KLIHKRQMELFPAPKPTMRLATYNKTQTGMTEAQFWAAVPSDTCSALAEQYPNWPATNG

NLREYVCPSKAERFAPSAYFTFTDGKLTSRSQSQLP
>non-antioxidant_1469
TNVISTLDLNLLTKGGGSWNVDGVNMKKSAVTTFDGKRVVKAVYDKNSGTSANPGVGG
FSFSAVPDGLNKNAITFAWEVFYPKGFDFARGGKHGGTFIGHGAASGYQHSKTGASNRIM
WQEKGGVIDYIYPPSDLKQKIPGLDPEGHGIGFFQDDFKNALKYDVWNRIEIGTKMNTFK
NGIPQLDGESYVIVNGKKEVLKRINWSRSPDLLISRFDWNTFFGGPLPSPKNQVAYFTNFQ
MKKYELEHHHHHH
>non-antioxidant_1470
AVINHDAVPVWPQPEPADATQALAVRFKPQLDVVNGCQPYPAVDPQGNTSGGLKPSGSQA
AACRDMSKAQVYSRSGTYNGYYAIMYSWYMPKDSPSTGIGHRHDWENVVVWLDNAAS
ANIVALSASAHSGYKKSFPADKSYLDGITAKISYKSTWPLDHELGFTTSAGKQQPLIQWEQ
MTQAARDALESTDFGNANVPFKSNFQDKLVKAFFQ
>non-antioxidant_1471
GMNTNLASFIVGLIIDENDRFYFVQKDGQTYALAKEEGQHTVGDTVKGFAYTDMKQKLR
LTTLEVTATQDQFGWGRVTEVRKDLGVFVDTGLPDKEIVVSLDILPELKELWPKKGDQLYI
RLEVDKKDRIWGLLAYQEDFQRLARPAYNNMQNQNWPAIVYRLKLSGTFVYLPENNML
GFIHPSERYAEPRLGQVLDARVIGFREVDRTLNLSLKPRSFEMLENDAQMILTYLESNGGF
MTLNDKSSPDDIKATFGISKGQFKKALGGLMKAGKIKQDQFGTELI
>non-antioxidant_1472
MTTVRIPAGWPATEEEARAVQDELRGRVILDEPGPPPGTGRVTGVDVAYDDERDVVVAAA
VVLDAATLDVVAEATAVGEVSFPYVPGLLAFREIPTVLAALDALPCPPGLIVCDGYGVAHP
RRFGLASHLGVLTGLPTIGVAKNPFTFSYEDPGAPRGSAAPLLAGADEVGRALRTQSGVKP
VFVSVGHRVDLDHACAHTLALTPKYRIPETTRRADSLCRRALKEATALEHHHHHH
>non-antioxidant_1473
MHHHHHHKLITLLLRSSKSEDLRLSIPVDFTVKDLIKRYCTEVKISFHERIRLEFEGEWLDP
NDQVQSTELEDEDQVSVVLD
>non-antioxidant_1474
GMEQHQVWAYQTKTHSVTLNSVDIPALAADDILVQNQAIGINPVDWKFIKANPINWSNGH
VPGVDGAGVIVKVGAKVDSKMLGRRVAYHTSLKRHGSFAEFTVLNTDRVMTLPDNLSFE
RAAALPCPLLTAWQAFEKIPLTKQREVLIVGFGAVNNLLTQMLNNAGYVVDLVSASLSQA
LAAKRGVRHLYREPSQVTQKYFAIFDAVNSQNAAALVPSLKANGHIICIQDRIPAPIDPAFT
RTISYHEIALGALHDFGDRQDWQILMQQGEALLTLIAQGKMEIAAPDIFRFEQMIEALDHS
EQTKLKTVLTLNE
>non-antioxidant_1475
SNAESAAPQVHLSILATTDIHANMMDYDYYSDKETADFGLARTAQLIQKHREQNPNTLLV
DNGDLIQGNPLGEYAVKYQKDDIISGTKTHPIISVMNALKYDAGTLGNHEFNYGLDFLDG
TIKGADFPIVNANVKTTSGENRYTPYVINEKTLIDENGNEQKVKVGYIGFVPPQIMTWDK
KNLEGQVQVQDIVESANETIPKMKAEGADVIIALAHTGIEKQAQSSGAENAVFDLATKTK
GIDAIISGHQHGLFPSAEYAGVAQFNVEKGTINGIPVVMPSSWGKYLGVIDLKLEKADGSW
KVADSKGSIESIAGNVTSRNETVTNTIQQTHQNTLEYVRK
>non-antioxidant_1476
GTGRSRLLEDFRNNRFPNLQLRDLIGHIVEFSQDQHGSRFIQQKLERATPAERQIVFNEILQ
AAYQLMTDVFGNYVIQKFFEFGSLDQKLALATRIRGHVLPLALQMYGCRVIQKALESISSD
QQSEMVKELDGHVLKCVKDQNGNHVVQKCIECVQPQSLQFIIDAFKGQVFVLSTHPYGC

RVIQRILEHCTAEQTLPILEELHQHTEQLVQDQYGNYVIQHVLEHGRPEDKSKIVSEIRGKV
LALSQHKFASNVVEKCVTHASRAERALLIDEVCCQNDGPHSALYTMMKDQYANYVVQK
MIDMAEPAQRKIIMHKIRPHITTLRKYTYGKHILAKLEKYYLKNSPDLG
>non-antioxidant_1477
MIVLENHTDISGKTSERVLHSAWLNSHYQTGLKNLLDTAVLEGTDEESARSLASRWQKID
EIPFDFERRRMSVVVAENTEHHQLVCKGALQEILNVCSQVRHNGEIVPLDDIMLRKIKRVT
DTLNRQGLRVVAVATKYLPAREGDYQRADESDLILEGYIAFLDHHHHHH
>non-antioxidant_1478
SNADDMIILKGVNIFPIQIETILLQFKELGSDYLITLETAESNDEMTVEVELSQLFTDDYGRL
QALTREITRQLKDEILVTPRVKLVPKGALPKSEGKAVRVKDLRKTF
>non-antioxidant_1479
GAQQQELPVPKPHQLKWHEAEMGAVFHYDLHVFDGIRYGQGNNRINPIEDYNIFNPTELN
TDQWVQAAKAAGCKFAVLTATHETGFGLWQSDVNPYCLKAVKWRDGKGDIVRDFVNSC
RKYGLQPGIYIGIRWNSLLGIHNFKAEGEGAFARNRQAWYKRLCEKMVTELCTRYGDLY
MIWFDGGADDPRADGPDVEPIVNKYQPNCLFYHNIDRADFRWGGSETGTVEYPCWSTFP
VPCSHHKRIESSIDQLELLKHGDKNGRYWVPAMADTPLRGANGRHEWFWEPDDENNIYP
LNTLMDKYEKSVGRNATLILGLTPDPTGLIPAGDAQRLKEMGDEINRRFSSPIARISGQKKS
LTLKLGKEQSVNYCIIQENIKNGERIRQYQIEAKVNGKWQTVCKGESVGHKRIEKFEPVEA
TALRLTVSESIALPDIINFSAYSVK
>non-antioxidant_1480
GGEGTDAIQALIQAYFTAWNTNAPERFAEIFWPDGSWVNVVGMHWRGRDQIVFAHTAFL
KTIFKDCKQELVTIEARTIAPGSALAVVTLIQDAYVTPDGRQMPRAHDRLTLLAVEREGVW
RFIHGHNTIVNPDAANNDPVLRMKPA
>non-antioxidant_1481
GMNLDSYERTGLRVSLDLVNIATPGSRRGTPHTGGCVIEDLHDLLKDDPASVAQLGDDHV
EGFVELARLLHTAIDALSNGQVATAATALNHLLRKHPATPELAQDPDGTWRLHHHPLDAE
LVPMWTAICAEGLAREIGHQNVRRFGICNAHRCDRVYFDTSRNGTRQYCSLACQNRVKA
AAFRERRAT
>non-antioxidant_1482
MSLKITEVKAHALSTPIPERMRVESGAGLKLNRQMILVEVRTDEGVTGVGSPSGPYDLAVL
KRAIEDVIGPQLIGEDPANINYLWHKVFHGEVSRNLGHRSVGIAAMSGVDIALWDLKGRA
MNQPIYQLLGGKFHTRGVRAYASSIYWDLTPDQAADELAGWVEQGFTAAKLKVGRAPR
KDAANLRAMRQRVGADVEILVDANQSLGRHDALAMLRILDEAGCYWFEEPLSIDDIEGH
RILRAQGTPVRIATGENLYTRNAFNDYIRNDAIDVLQADASRAGGITEALAISASAASAHL
AWNPHTFNDIITVAANLHLVAASPHPAMFEWDITHNDLMTRLASYDLKLENGLVQPPQGP
GLGFEIDWDFVAAHAWKGEPAIGAGHGMKKEGHHHHHH
>non-antioxidant_1483
GAGTQTAQTQALDSDGIPTGGEWITMFDGKTLNGWRGYCRQDVPLGWVVEDGSITYKG
SDNKADTGFGDLIYDKKFKNFVFEIEWKIDKAGNSGIFYTAQEIEGTPIYYSSPEYQLLDNE
NMPDAWEGCDGNRQAGAVYDMIMPDPQPVKPYGNWNKTRIVVYNQRVIHYMNDVKIL
EFQFGTPVWRALVDHSKFSKFSTSPEKCPEAYDLMLQCGKQPGYIGMQDHGYGVCFRNIR
IKEL
>non-antioxidant_1484
MGVLITGCGSRGDTEPLVALAARLRELGADARMCLPPDYVERCAEVGVPMVPVGRAVRA

GAREPGELPPGAAEVVTEVVAEWFDKVPAAIEGCDAVVTTGLLPAAVAVRSMAEKLGIPY
RYTVLSPDHLPSEQSQAERDMYNQGADRLFGDAVNSHRASIGLPPVEHLYDYGYTDQPW
LAADPVLSPLRPTDLGTVQTGAWILPDQRPLSAELEGFLRAGSPPVYVGFGSGPAPAEAAR
VAIEAVRAQGRRVVLSSGWAGLGRIDEGDDCLVVGEVNHQVLFGRVAAVVHHGGAGTTT
AVTRAGAPQVVVPQKADQPYYAGRVADLGVGVAHDGPTPTVESLSAALATALTPGIRARA
AAVAGTIRTDGTTVAAKLLLEAISRQRSSVPAAKLAAALEHHHHHH
>non-antioxidant_1485
MNTYFDIPHRLVGKALYESYYDHFGQMDILSDGSLYLIYRRATEHVGGSDGRVVFSKLEG
GIWSAPTIVAQAGGQDFRDVAGGTMPSGRIVAASTVYETGEVKVYVSDDSGVTWVHKFT
LARGGADYNFAHGKSFQVGARYVIPLYAATGVNYELKWLESSDGGETWGEGSTIYSGNT
PYNETSYLPVGDGVILAVARVGSGAGGALRQFISLDDGGTWTDQGNVTAQNGDSTDILVA
PSLSYIYSEGGTPHVVLLYTNRTTHFCYYRTILLARAVAGSSGWTERVPAYSAPAASGYTSQ
VVLGGRRILGNLFRETSSTTSGAYQFEVYLGGVPDFESDWFSVSSNSLYTLSHGLQRSPRR
VVVEFARSSSPSTWNIVMPSYFNDGGHKGSGAQVEVGSLNIRLGTGAAVWGTGYFGGID
NSATTRLATGYYRVRAWI
>non-antioxidant_1486
MSLSTMLVAEDLSAVGGISLSSALPVLTAMQYDVAALPTSLLSTHTSGYGTPAVVDLSTWL
PQVFAHWTRAQLHFDQALIGYVGSVALCQQITTYLEQQTLSLLVVDPVLGDLGQLYQGFD
QDYVAAMRQLIQQADVILPNTTEAALLTGAPYQVTPDLEVILPALQAQLKTGAHAVITDV
QRADQIGCAWLDEAGHVQYCGARRLPGHYNGTGDTLAAVIAGLLGRGYPLAPTLARAN
QWLNMAVAETIAQNRTDDRQGVALGDLLQAILALNEGHHHHHH
>non-antioxidant_1487
SEESRESPAEHGYYMPAEWDSHAQTWIGWPERQDNWRHNALPAQRVFAGVAKAISKFEP
VTVCASPAQWENARKQLPEDIRVVEMSMNDSWFRDSGPTFIVRKRPVKLSSLNRNIAGID
WNFNAWGGANDGCYNDWSHDLLVSRKILALERIPRFQHSMILEGGSIHVDGEGTCLVTEE
CLLNKNRNPHMSKEQIEEELKKYLGVQSFIWLPRGLYGDEDTNGHIDNMCCFARPGVVLL
SWTDDETDPQYERSVEALSVLSNSIDARGRKIQVIKLYIPEPLYMTEEESSGITQDGEAIPRL
AGTRLAASYVNFYIANGGIIAPQFGDPIRDKEAIRVLSDTFPHHSVVGIENAREIVLAGGNI
HCITQQQPAEPTSVAENGH
>non-antioxidant_1488
GAMGALETVPKDLRHLRACLLCSLVKTIDQFEYDGCDNCDAYLQMKGNREMVYDCTSS
SFDGIIAMMSPEDSWVSKWQRVSNFKPGVYAVSVTGRLPQGIVRELKSRGVAYKSRDTAIK
T
>non-antioxidant_1489
MAHHHHHHVDDDDKMVTDQRWLIDKSALVRLTDSPDMEIWSNRIERGLVHITGVTRLEV
GFSAECGEIARREFREPPLSAMPVEYLTPRIEDRALEVQTLLADRGHHRGPSIPDLLIAATA
ELSGLTVLHVDKDFDAIAALTGQKTERLTHRPPSA
>non-antioxidant_1490
TQVAKKILVTCALPYANGSIHLGHMLEHIQADVWVRYQRMRGHEVNFICADDAHGTPIM
LKAQQLGITPEQMIGEMSQEHQTDFAGFNISYDNYHSTHSEENRQLSELIYSRLKENGFIK
NRTISQLYDPEKGMFLPDRFVKGTCPKCKSPDQYGDNCEVCGATYSPTELIEPKSVVSGAT
PVMRDSEHFFFDLPSFSEMLQAWTRSGALQEQVANKMQEWFESGLQQWDISRDAPYFGF
EIPNAPGKYFYVWLDAPIGYMGSFKNLCDKRGDSVSFDEYWKKDSTAELYHFIGKDIVYF
HSLFWPAMLEGSNFRKPSNLFVHGYVTVNGAKMSKSRGTFIKASTWLNHFDADSLRYYY

TAKLSSRIDDIDLNLEDFVQRVNADIVNKVVNLASRNAGFINKRFDGVLASELADPQLYKT
FTDAAEVIGEAWESREFGKAVREIMALADLANRYVDEQAPWVVAKQEGRDADLQAICSM
GINLFRVLMTYLKPVLPKLTERAEAFLNTELTWDGIQQPLLGHKVNPFKALYNRIDMRQV
EALVEASK
>non-antioxidant_1491
MSLSFTPLHTTSEAFIEKALPWLEDRYFHIAYLNPNGYTAYPQGAFRHYLAFGSEAAIHVS
DATRVFETWNEIKKGYTNEWIFVFASYDGKNSVEQLHTSKEAGIAFAAATFFIPEHVWEIQ
PDGILIHKGSGSSLVTEIQHAEPSTPVQQSDIFVKQVVSKESYFNAFDELQQIIAQGDAYEIN
YCIPFTAKGNISPAATYQRLNKKTPMPFSVYYKFNTEYILSASPERFIKKTGDTIISQPIKGTS
KRGKSKAEDEMLKQQLGTSEKEQSENTMIVDLVRNDLSRTAVAGSVCVPELSGLYTFPNV
HQLISTVQSTIDPACSSIDVIQQAFPMGSMTGAPKVNVMKFIDRIESMARGPFSGTVGYMD
PHDNFDFNVLIRSIFYNSATQELFMEAGSAITSYAKAETEYEECLLKITPMIHILNNQEGHH
HHHH
>non-antioxidant_1492
GASETQQWKTLEDTRSALMGVYGLTRAALADNNTHWICGDLRKGDFTVYKRSDLQAVS
DNELNKPYDLLKKVSNWRRFYAVINAASVFMEKAPRTVELDRSYSEQNLKYDIAQVRAL
RAFAYFYMVRIWGDVPLVTYSYDNGTFPSMPRTDAQTVLSYAKAELLTAIEDLPYQYGTQ
TNLYYGSYGAQWQGKLFNKLSAYSVLAHICAWQGNYAEAETYSAFIIDHASEINAKYTSI
ADLTSETGLFYSNASVKGSRILGFNFAHNDNEATQSGHLEQLTLAYPLVQKSYPEIYISKDS
LFSIFTNFDDLRFGIIDTIKYSSYYVQNLNEETPVFSKIKIIQDGSAKDNDFGVFGSSIVFTRL
EDITLLRAEALCALNRSTEAVSYLNMIRTNRGLREVSFKKDFGNNRESLIAEIFEERRRELM
GEGWRWYDLVRRQKLMKDNEAFLRLISSGGIYWPVSEDIITANSQIEQNEFWK
>non-antioxidant_1493
PQFEKIEGRMIRILYLLVKPESMSHEQFRKECVVHFQMSAGMPGLHKYEVRLVAGNPTDT
HVPYLDVGRIDAIGECWFASEEQYQVYMESDIRKAWFEHGKYFIGQLKPFVTEELV
>non-antioxidant_1494
MSRFDSGLPSVRQVQLLIKDQTPVEIKLLTGDSLFGTIRWQDTDGLGLVDDSERSTIVRLA
AIAYITPRR
>non-antioxidant_1495
MTIGQKNTNIDPRFPHHHPRPQSFWEARAKALESLLIEKGHLSSDAIERVIKHYEHELGPM
NGAKVVAKAWTDPAFKQRLLEDSETVLRELGYYGLQGEHIRVVENTDTVHNVVVCTLCS
CYPWPLLGLPPSWYKEPAYRARVVKEPRQVLKEFGLDLPDSVEIRVWDSSSEIRFMVLPQR
PEGTEGMTEEELAKLVTRDSMIGVAKIEPPKVTVG
>non-antioxidant_1496
MNGIHDVGGMDGFGKVMYVKEEEDIYFTHDWERLALGLVAGCMAQGLGMKAFDEFRI
GIELMRPVDYLTSSYYGHWIATVAYNLVDTGVLDEKELDERTEVFSKKPDTKIPRREDPAL
VKLVEKALNDGLSPLREISASPRFKVGERIKTKNIHPTGHTRFPRYARDKYGVIDEVYGAH
VFPDDAAHRKGENPQYLYRVRFEAEELWGYKQKDSVYIDLWESYMEPVSH
>non-antioxidant_1497
MGKNVVVLGTQWGDEGKGKVVDLLTERAKYVVRYQGGHNAGHTLVINGEKTVLHLIPS
GILRENVISIIGNGVVLAPDALMKEMTELEARGVPVRERLLLSEACPLILPYHVALDNARE
KARGAKAIGTTGRGIGPAYEDKVARRGLRVSDLFNKETFAIKLKEIVEYHNFQLVHYYKEA
AVDYQKVLDDVLAIADILTAMVVDVSELLDNARKQGELIMFEGAQGTLLDIDHGTYPYV
TSSNTTAGGVATGSGLGPRYVDYVLGIVKAYSTRVGAGPFPTELNDETGEFLRKQGNEYG

ATTGRSRRTGWLDIVAVRRAVQINSLSGFCMTKLDVLDGLKEVKLCVGYRMPDGREVDT
TPLAAEGWEGIEPIYETMPGWSETTFGVKEHSKLPQAALNYIQRVEELTGVPIDIISTGPDR
DETMILRDPFDA
>non-antioxidant_1498
GMQGKKFISPGAWFSMNYPSDWNEFEDGEGSFLFYNPDVWTGNFRISAFKGNASYGKDA
IRQELKENDSASLVKIGTWDCAYSKEMFQEEGTYYTSHLWITGTGNIAFECSFTVPKGGSA
KEAEEVIATLEARKEGEKYPAELIPVRLSEIYQINEGYEWVVSTVKQELKKDFQGVEEDLE
KIQQVIDSGKISPKKKDEWLAIGITVCAILTNEVEGMEWKTLIDGNREVPVLEYQGRTIDP
MKIAWSKVKAGQPCNIAEAYQSAIDHH
>non-antioxidant_1499
GMSSSGIEIAKPFVTATTNVLSTMAGIQPIPGQPYVKKNNVAKGDVSAVVGITGHKNGSIS
VTFTKQCAIAVVKAMLGDDIQDIIQDTKDAVGEVTNMISGQARAALSEMGMTFQGATPSV
IMGDGHTISHVTKSPVIAIPFKTNHGEFTVEFCLE
>non-antioxidant_1500
SEDNGVDFNKNTALDKNYLLNNKIPSNNVEEAGSKNYVHYIIQLQGDDISYEATCNLFSK
NPKNSIIIQRNMNESAKSYFLSDDGESILELNKYRIPERLKNKEKVKVTFIGHGKDEFNTSE
FARLSVDSLSNEISSFLDTIKLDISPKNVEVNLLGCNMFSYDFNVEETYPGKLLLSIMDKITS
TLPDVNKNSITIGANQYEVRINSEGRKELLAHSGKWINKEEAIMSDLSSKEYIFFDSIDNKL
KAKSKNIPGLASISEDIKTL
>non-antioxidant_1501
MDNTILILGDMLSAAYGLQQEEGWVKLLQDKYDAEQSDIVLINASISGETSGGALRRLDA
LLEQYEPTHVLIELGANDGLRGFPVKKMQTNLTALVKKSQAANAMTALMEIYIPPNYGPR
YSKMFTSSFTQISEDTNAHLMNFFMLDIAGKSDLMQNDSLHPNKKAQPLIRDEMYDSIKK
WLNNV
>non-antioxidant_1502
SVSVDLNVDPSLQIDIPDALSERDKVKFTVHTKTTLPTFQSPEFSVTRQHEDFVWLHDTLT
ETTDYAGLIIPPAPTKPDFDGPREKMQKLGEGEGSMTKEEFAKMKQELEAEYLAVFKKTV
SSHEVFLQRLSSHPVLSKDRNFHVFLEYDQDLSVRRKNTK
>non-antioxidant_1503
MQFKVYTYKRESRYRLFVDVQSDIIDTPGRRMVIPLASARLLSDKVSRELYPVVHIGDES
WRMMTTDMASVPVSVIGEEVADLSHRENDIKNAINLMFWGI
>non-antioxidant_1504
GPERTSIAVHALMGLPTGQPANGTKLDSIGLPKVDGMSFTLYRVNEIDLTTQAGWDAASKI
KLEELYTNGHPTDKVTKVATKKTEGGVAKFDNLTPALYLVVQELNGAEAVVRSQPFLVAA
PQTNPTGDGWLQDVHVYPKHQALSEPVKTAVDPDATQPGFSVGENVKYRVATKIPEIASN
TKFEGFTVADKLPAELGKPDTNKITVTLGGKPINSTDVSVQTYQVGDRTVLSVQLAGATL
QSLDQHKDQELVVEFEAPVTKQPENGQLDNQAWVLPSNPTAQWDPEESGDAALRGMPSS
RVSSKFGQITIEKSFDGNTPGADRTATFQLHRCEADGSLVKSDPPISLDGKQEFVTGQDGKA
VLSGIHLGTLQLESNVMKYTDAWAGKGTEFCLVETATASGYELLPKPVIVKLEANESTNVL
VEQKVKIDNKKKN
>non-antioxidant_1505
MSLGELNVATVTRRPEILLVDSQEVILQRLQQLLSPLPYTLHFARDATQALQLLASREVDLV
ISAAHLPQMDGPTLLARIHQQYPSTTRILLTGDPDLKLIAKAINEGEIYRYLSKPWDDQELL
LALRQALEHQHSERERLRLQDEGHHHHHH

>non-antioxidant_1506
GAMGSNPAEELAAAAQRGDVIAILQQALETNSFRLLFQPVISLRGDSHENYEVLLRLLNPQ
GQEVPPAEFLHAAKEAGLAEKIDRWVILNSIKLLAEHRAKGHQTKLFVHLSSASLQDPGL
LPWLGVALKAARLPPESLVFQISEADATSYLKQAKQLTQGLATLHCQAAISQFGCSLNPFN
ALKHLTVQFIKIDGSFVQDLNQVENQEILKGLIAELHEQQKLSIVPFVESASVLATLWQAG
ATYIQGYYLQGPSQAMDYDFSSGDE
>non-antioxidant_1507
MGDTKEQRILRYVQQNAKPGDPQSVLEAIDTYCTQKEWAMNVGDAKGQIMDAVIREYSP
SLVLELGAYCGYSAVRMARLLQPGARLLTMEMNPDYAAITQQMLNFAGLQDKVTILNGA
SQDLIPQLKKKYDVDTLDMVFLDHWKDRYLPDTLLLEKCGLLRKGTVLLADNVIVPGTP
DFLAYVRGSSSFECTHYSSYLEYMKVVDGLEKAIYQGPSSPDKS
>non-antioxidant_1508
GQELKGKYMKTPTGYLMVLRHGDNVLQNLEQLARDEHIPSASFVGIGFMSEATFGFYDF
GRKQFDPKTYRNVEMANMTGSIAWKEGKPSIHAHGTVTDGTFQGAGGHLLGLTVGTGSC
EITVTVYPQRLDRFVDPEIQANVLGLPQQ
>non-antioxidant_1509
GMSYQNANYSAFYVSEPFSESNLGANSTHDFVYYNMLRMWKGEDNSFPFNDAHDKTYN
VRDGSDWEKTLKPRLHTRLDNSKNIILFLSSITANSRALREEMNYGIGTKGLPVIVIYPDYD
KKSDIVDSNGNFKKQIKDLWDKLPAFRDNMSSVATLHIPCTKSVIISALNNEDFMVNTMA
DAEKYYYKP
>non-antioxidant_1510
GMSSKEEILSILEAFASTERMGSFFLDNATADFLFIRPSGNPLDAKGFENMWSSGDLVLESA
EITKVHKFELLGSNAAICVFTLGSKFTYKGTQNDDLPTVTSIFKKIDEKWKVAWMQRSSG
QSDMTLWNE
>non-antioxidant_1511
APAAVDWRARGAVTAVKDQGQCGSCWAFSAIGNVECQWFLAGHPLTNLSEQMLVSCDKT
DSGCSGGLMNNAFEWIVQENNGAVYTEDSYPYASGEGISPPCTTSGHTVGATITGHVELPQ
DEAQIAAWLAVNGPVAVAVDASSWMTYTGGVMTSCVSEQLDHGVLLVGYNDSAAVPYW
IIKNSWTTQWGEEGYIRIAKGSNQCLVKEEASSAVVG
>non-antioxidant_1512
MAFQLHPRLQQDCIVLGNLPLCKVLLIKEDIGPWLILVPRIEELKEIHHMTDEQQIQFIKESS
AVAQLLEDNFSPDKINIGALGNLVPQLHIHHIARFTTDVAWPGPVWGNTTGVIRAQSSQTQ
LVDLLRDKLSNISGFKRLEHHHHHH
>non-antioxidant_1513
SHMSKIKGNVKWFNESKGFGFITPEDGSKDVFVHFSAIQTNGFKTLAEGQRVEFEITNGAK
GPSAANVTAL
>non-antioxidant_1514
MQEPLAAGAVILRRFAFNAAEQLIRDINDVASQSPFRQMVTPGGYTMSVAMTNCGHLGW
TTHRQGYLYSPIDPQTNKPWPAMPQSFHNLCQRAATAAGYPDFQPDACLINRYAPGAKLS
LHQDKDEPDLRAPIVSVSLGLPAIFQFGGLKRNDPLKRLLLEHGDVVVWGGESRLFYHGI
QPLKAGFHPLTIDCRYNLTFRQAGKKENLYFQ
>non-antioxidant_1515
MSLEAIRIGEINSYSQIPAFTLPYRNGWQLAVEQINAAGGLLGGRPLEVISRDDGGDPGKAV
TAAQELLTRHGVHALAGTFLSHVGLAVSDFARQRKVLFMASEPLTDALTWEKGNRYTYR

LRPSTYMQAAMLAAEAAKLPITRWATIAPNYEYGQSAVARFKELLLAARPEVTFVAEQWP
ALYKLDAGPTVQALQQAEPEGLFNVLFGADLPKFVREGRVRGLFAGRQVVSMLTGEPEY
LNPLKDEAPEGWIVTGYPWYDIDTAPHRAFVEAYRARWKEDPFVGSLVGYNTLTAMAVA
FEKAGGTESETLVETLKDMAFSTPMGPLSFRASDHQSTMGAWVGRTALRDGKGVMVDW
RYVDGGSVLPPPEVVSAWRPAGEGHHHHHH
>non-antioxidant_1516
GCDLERYPLTDLSEETFWNSESNAELALTSLYRGSLTDGVEYNPSDWWSYHGMIMMEHL
SDNAFDRRGENNPFFKISSGNLTADNAFIKRYWETSYKRIGYCNRFLVGIQNSSESEKKTR
MIAEARFLRATQYFYLASYFKNVPLVENVLTGEEANNVTKTSQADILKWCVTEFTAAAAD
LPRFSAIPAGEAGRACKQAALAFLGRTCMLQKDWKSGAKAFHDIMELGDNAINANYQEL
FYPSTGTSNKENIFYIQYLENYLGTGLPQHALSAKDGGWSLVNPAADLYESYEFKDGTPFS
YDDPRYDPSNLGKDRDPRLDYTIYYNGAIFMGTEYKMSPDYSAAKKEKLDYTSEASRTG
FMMRKYFEESTPINDVQSANGLTPVIRYAEVLLGYLECLVEDNQTITQGILDETINAVRGRA
SVNMPPVTEVTPAKLREIVRHERRIELAMEGIRYWDIMRWGIAHEVLSQKIWGAPYPGST
QYATTTKEVDPTGNYRWYVGKRAFRNPTDYTWPIPQSEQNINPNLRD
>non-antioxidant_1517
GHMTKLEQIQQWTAQHHASMTYLSNPKTIEYLTGFGSDPIERVLALVVFPDQDPFIFAPALE
VEVIKETGWQFPVIGYLDHENPWAMIADQVKQRHVNPEHVAIEKGQLQVARMEALAAQF
SAPSFDLDITSFIEHMRGS
>non-antioxidant_1518
MAVTDLSLTNSSLMPTLNPMIQQLALAIAASWQSLPLKPYQLPEDLGYVEGRLEGEKLVIE
NRCYQTPQFRKMHLELAKVGKGLDILHCVMFPEPLYGLPLFGCDIVAGPGGVSAAIADLS
PTQSDRQLPAAYQKSLAELGQPEFEQQRELPPWGEIFSEYCLFIRPSNVTEEERFVQRVVDF
LQIHCHQSIVAEPLSEAQTLEHRQGQIHYCQQQQKNDKTRRVLEKAFGEAWAERYMSQVL
FDVIQ
>non-antioxidant_1519
MPAFDKPKVELHVHLDGSIKPETILYYGRRRGIALPANTAEGLLNVIGMDKPLTLPDFLAK
FDYYMPAIAGCREAIKRIAYEFVEMKAKEGVVYVEVRYSPHLLANSKVEPIPWNQAEGDL
TPDEVVALVGQGLQEGERDFGVKARSILCCMRHQPNWSPKVVELCKKYQQQTVVAIDLA
GDETIPGSSLLPGHVQAYQEAVKSGIHRTVHAGEVGSAEVVKEAVDILKTERLGHGYHTL
EDQALYNRLRQENMHFEICPWSSYLTGAWKPDTEHAVIRLKNDQANYSLNTDDPLIFKST
LDTDYQMTKRDMGFTEEEFKRLNINAAKSSFLPEDEKRELLDLLYKAYGMPPSASAGQNL
AENLYFQ
>non-antioxidant_1520
GESSKSGYQTTGENNSSDYQGIIEDGEYKTSKSRGVGISQNSDNLLNLKSFEAGLTTISKDH
FSTKSYIFQEGQYLNKATIQDWLGRKSSSNPEGLNPSDNGKKEANKRNPIYVQQIEEQDY
MKQNNGKLELAGMTIGIGMNQKDYYQKEQYGATYSTTISKEKRIEEGKIAAKKVLARVR
QKVGNNVPIVIAMFAQAPNDSLVGGYFYSYTVSKSGTDIGSWTETNIKSYVLPATEDNKLP
NDNDSTSFDNFQKEVKNFFPNISNVTGQGQYKDKTLQGLHITITTQFYSETEITSFTQYVAQ
AAKSYLPSGIPVDIKINGSDGETQSFVSTTGGNGGYYTHVFGSY
>non-antioxidant_1521
YEVPKAKIDVFYPKGFEVSIPDEEGITLFAFHGKLNEEMEGLEAGTWARDIVKAKNGRWT
FRDRITALKPGDTLYYWTYVIYNGLGYREDDGSFVVNGYSGNNASPH
>non-antioxidant_1522

MSLIYTITLNPAIDRLLFIRGELEKRKTNRVIKTEFDCGGKGLHVSGVLSKFGIKNEALGIAG
SDNLDKLYAILKEKHINHDFLVEAGTSTRECFVVLSDDTNGSTMIPEAGFTVSQTNKDNLL
KQIAKKVKKEDMVVIAGSPPPHYTLSDFKELLRTVKATGAFLGCDNSGEYLNLAVEMGV
DFIKPNEDEVIAILDEKTNSLEENIRTLAEKIPYLVVSLGAKGSICAHNGKLYQVIPPKVQER
NDTGAGDVFVGAFIAGLAMNMPITETLKVATGCSASKVMQQDSSSFDLEAAGKLKNQVS
IIQLEEREGHHHHHH
>non-antioxidant_1523
QVTLKESGPGILKPSQTLSLTCSFSGFSLSTSGMSVGWIRQPSGKGLEWLAHIWWDDDKY
YNPSLKSRLTISKDTSRNQVFLKITSVDTADTATYYCARRTTTADYFAYWGQGTTLTVSSA
KTTPPSVYPLAPGSAAQTNSMVTLGCLVKGYFPEPVTVTWNSGSLSSGVHTFPAVLQSDL
YTLSSSVTVPSSTWPSETVTCNVAHPASSTKVDKKIVPRDC
>non-antioxidant_1524
SNAMSLLEQLDKNIAASGGLIVSCQPVPGSPLDKPEIVAAMALAAEQAGAVAVRIEGIDNL
RMTRSLVSVPIIGIIKRDLDESPVRITPFLDDVDALAQAGAAIIAVDGTARQRPVAVEALLAR
IHHHHLLTMADCSSVDDGLACQRLGADIIGTTMSGYTTPDTPEEPDLPLVKALHDAGCRVI
AEGRYNSPALAAEAIRYGAWAVTVGSAITRLEHICGWYNDALKKAAS
>non-antioxidant_1525
ADEIGDAAKKLGDASYAFAKEVDWNNGIFLQAPGKLQPLEALKAIDKMIVMGAAADPKL
LKAAAEAHHKAIGSVSGPNGVTSRADWDSVNAALGRVIASVPENMVMDVYDSVSKITDP
KVPAYMKSLVNGADAEKAYEGFLAFKDVVKKSQ
>non-antioxidant_1526
MTGREILEKLERREFTREVLKEALSINDRGFNEALFKLADEIRRKYVGDEVHIRAIIEFSNV
CRKNCLYCGLRRDNKNLKRYRMTPEEIVERARLAVQFGAKTIVLQSGEDPYYMPDVISDI
VKEIKKMGVAVTLSLGEWPREYYEKWKEAGADRYLLRHETANPVLHRKLRPDTSFENRL
NCLLTLKELGYETGAGSMVGLPGQTIDDLVDDLLFLKEHDFDMVGIGPFIPHPDTPLANEK
KGDFTLTLKMVALTRILLPDSNIPATTAMGTIVPGGREITLRCGANVIMPNWTPSPYRQLYQ
LYPGKICVFEKDTACIPCVMKMIELLGRKPGRDWGGRKRVFETV
>non-antioxidant_1527
TAQTKNTQTLMPLTERVNVQADSARINQIIDGCWVAVGTNKPHAIQRDFTNLFDGKPSYRF
ELKTEDNTLEGYAKGETKGRAEFSYCYATSDDFRGLPADVYQKAQITKTVYHHGKGACP
QGSSRDYEFSVYIPSSLDSNVSTIFAQWHGMPDRTLVQTPQGEVKKLTVDEFVELEKTTFF
KKNVGHEKVARLDKQGNPVKDKNGKPVYKAGKPNGWLVEQGGYPPLAFGFSGGLFYIK
ANSDRKWLTDKDDRCNANPGKTPVMKPLTSEYKASTIAYKLPFADFPKDCWITFRVHIDW
TVYGKEAETIVKPGMLDVRMDYQEQGKKVSKHIVDNEKILIGRNDEDGYYFKFGIYRVG
DSTVPVCYNLAGYSER
>non-antioxidant_1528
SNASVIVGRALPEVRDGLKPVHRRVLYAMFDSGFRPDRSHAKSARSVAETMGNYHPHGD
ASIYDSLVRMAQPWSLRYPLVDGQGNFGSPGNDPPAAMRYTEARLTPLAMEMLREIDEET
VDFIPNYDGRVQEPTVLPSRFPNLLANGSGGIAVGMATNIPPHNLRELADAVFWALENHDA
DEEETLAAVMGRVKGPDFPTAGLIVGSQGTADAYKTGRGSIRMRGVVEVEEDSRGRTSLV
ITELPYQVNHDNFITSIAEQVRDGKLAGISNIEDQSSDRVGLRIVIEIKRDAVAKVVINNLYK
HTQLQTSFGANMLAIVDGVPRTLRLDQLIRYYVDHQLDVIVRRTTYRLRKANERAHILRG
LVKALDALDEVIALIRASETVDIARAGLIELLDIDEIQAQAILDMQLRRLAALERQRIIDDL
AKIEAEIADLEDILAKPERQRGIVRDELAEIVDRHGDDRRTRIIA

>non-antioxidant_1529
GMDEKPAITKIISGGQTGADRAALDFAIKHHIPYGGWVPKGRLAEGGRVPETYQLQEMPT
SDYSKRTEKNVLDSDGTLIISHGILKGGSALTEFFAEQYKKPCLHIDLDRISIEDAATLINSW
TVSHHIQVLNIAGPRAGKDPEIYQATMDLLEVFLA
>non-antioxidant_1530
TVAYIAIGSNLASPLEQVNAALKALGDIPESHILTVSSFYRTPPLGPQDQPDYLNAAVALETS
LAPEELLNHTQRIELQQGRVRKAERWGPRTLDLDIMLFGNEVINTERLTVPHYDMKNRGF
MLWPLFEIAPELVFPDGEMLRQILHTRAFDKLNKW
>non-antioxidant_1531
SNANKYNKIANELIKIIGEDNIISITHCATRLRVMVKDREIINDKKVEKVDEVKGVFFTSGQ
YQIILGTGIVNKVYAEVEKMGLKTLSKKEQDEL
>non-antioxidant_1532
MGSDKIHHHHHHMKELIRKLTEAFGPSGREEEVRSIILEELEGHIDGHRIDGLGNLIVWKG
SGEKKVILDAHIDEIGVVVTNVDDKGFLTIEPVGGVSPYMLLGKRIRFENGTIGVVGMEGE
TTEERQENVRKLSFDKLFIDIGANSREEAQKMCPIGSFGVYDSGFVEVSGKYVSKAMDDR
IGCAVIVEVFKRIKPAVTLYGVFSVQEEVGLVGASVAGYGVPADEAIAIDVTDSADTPKAIK
RHAMRLSGGPALKVKDRASISSKRILENLIEIAEKFDIKYQMEVLTFGGTNAMGYQRTREG
IPSATVSIPTRYVHSPSEMIAPDDVEATVDLLIRYLGA
>non-antioxidant_1533
MVGYSSKLIFVSMITRHGDRAPFANIENANYSWGTELSELTPIGMNQEYNLGLQLRKRYID
KFGLLPEHYVDQSIYVLSSHTNRTVVSAQSLLMGLYPAGTGPLIGDGDPAIKDRFQPIPIMT
LSADSRLIQFPYEQYLAVLKKYVYNSPEWQNKTKEAAPNFAKWQQILGNRISGLNDVITV
GDVLIVAQAHGKPLPKGLSQEDADQIIALTDWGLAQQFKSQKVSYIMGGKLTNRMIEDLN
NAVNGKSKYKMTYYSGHALTLLEVMGTLGVPLDTAPGYASNLEMELYKDGDIYTVKLRY
NGKYVKLPIMDKNNSCSLDALNKYMQSINEKFQKHHHHHH
>non-antioxidant_1534
MTNNNQIGENKEQTIFDHKGNVIKTEDREIQIISKFEEPLIVVLGNVLSDEECDELIELSKSK
LARSKVGSSRDVNDIRTSSGAFLDDNELTAKIEKRISSIMNVPASHGEGLHILNYEVDQQY
KAHYDYFAEHSRSAANNRISTLVMYLNDVEEGGETFFPKLNLSVHPRKGMAVYFEYFYQ
DQSLNELTLHGGAPVTKGEKWIATQWVRRGTYK
>non-antioxidant_1535
ENEIERPEDMRVRTLANKSKMKVSIVQQIDRKVALDDIAVSHGLDFPELLSEVETIVYSGTR
INIDYFINEVMDEDHLEDIFEYFKESTTDSLEEAMQELGKDYSEEEIRLVRIKFLSEMAN
>non-antioxidant_1536
GMEQQHPTIHTLKIETEFFKAVKERRKTFEIRKNDRNFQVGDILILEEYMNGMYLDDECEA
EVIYITDYAQREGYVVLGIELH
>non-antioxidant_1537
NLYFQGVAIKLSSIDQFEQVIEENKYVFVLKHSETCPISANAYDQFNKFLYERDMDGYYLI
VQQERDLSDYIAKKTNVKHESPQAFYFVNGEMVWNRDHGDINVSSLAQAEE
>non-antioxidant_1538
GSGGSGKVVKFSYMWTINNFSFCREEMGEVIKSSTFSSGANDKLKWCLRVNPKGLDEES
KDYLSLYLLLVSCPKSEVRAKFKFSILNAKGEETKAMESQRAYRFVQGKDWGFKKFIRRD
FLLDEANGLLPDDKLTLFCEVSVVQD
>non-antioxidant_1539

MPNILYKIDNQYPYFTKNEKKIAQFILNYPHKVVNMTSQEIANQLETSSTSIIRLSKKVTPG
GFNELKTRLSKFLPKEVTQYNVELVDNESTISLKNKLHSRSKAAL
>non-antioxidant_1540
MALVDGFLELERSSGKLEWSAILQKMASDLGFSKILFGLLPKDSQDYENAFIVGNYPAAW
REHYDRAGYARVDPTVSHCTQSVLPIFWEPSIYQTRKQHEFFEEASAAGLVYGLTMPLHG
ARGELGALSLSVEAENRAEANRFMESVLPTLWMLKDYALQSGAGLAFEHPVSK
>non-antioxidant_1541
GGTAYWKNPDQFTAFNTGLHALLREKSYNFFLLGEPRADIYGDNPIGGEASQGMERLPFN
TINKENVGISNYGDMYKIINQINQMIAKTTETTILTEATQNYYLGEAYGMRAYLYFHLLRS
WGDVVLYLDYTEGQNLDLSNITKGVSPATEVMEQIKKDIQASENAFGSDYSFKLGRHFWS
AAATQMLKGEAYLWSGRQMNGGNSDYTIAKNAFENVKKADVGLVTSSFKDIFSFENKKN
KEMIFTIHNGKDEYEMWGGYYRMRLIPAQDKMVKIYCDENGNSFVGTPDAQLNGLTQLQ
VRREFYFKGFRNNDTRWTTSLKAVYKKDAQGVVSYFGPITYKFQGTMLEGGSTRSFLDD
FPIYRYADCLLQLAMAKVLLGEDPTEEINAVRERAYGSKYFNEHKAEIAYPNDNDPEFYTD
NKWMKPDNAGALEAILKERLREFMFEGKRWYDIRLLGWDYVHQYSSAEQSRLLWPIDA
GTLTNNSALKQTPGYE
>non-antioxidant_1542
MATKLDYEDAVFYFVDDDKICSRDSIIDLIDEYITWRNHVIVFNKDITSCGRLYKELMKFD
DVAIRYYGIDKINEIVEAMSEGDHYINFTKVHDQESLFATIGICAKITEHWGYKKISESRFQS
LGNITDLMTDDNINILILFLEKKLN
>non-antioxidant_1543
MSLTDAKIRTLKPSDKPFKVSDSHGLYLLVKPGGSRHWYLKYRISGKESRIALGAYPAISLS
DARQQREGIRKMLALNINLEHHHHHH
>non-antioxidant_1544
GAMGSKISEQLKCCSGILKEMFAKKHAAYAWPFYKPVDVEALGLHDYCDIIKHPMDMSTI
KSKLESREYRDAQEFGADVRLMFSNCYKYNPPDHEVVAMARKLQDVFEMRFAKMPDEP
EE
>non-antioxidant_1545
NLYFQGMIPLEQGIEFLSVNVEEDSPVVGKKLKDLPLPRDSIIAAIVRGGVLVVPRGDTEIL
SGDKLYVIVSAEAKETVEETLLGR
>non-antioxidant_1546
GIDPFTVRTRVSEGLVLAEPAKLMISTDGSASTADLTRATTTWNQQSNNLGASSKYVTSVL
MDAGNTGVITITYVADQVGLPTAGNTLILSPYINDGNTRTALATAVAAGTRGTIDWACTSA
SNATATAQGFTGMAAGSVPQEFAPAQCR
>non-antioxidant_1547
MGGGEVKMMSLLEEMKGIYSKKGGKVKPFEKFEGELKEGYRFEYEKKLCEIDVAMFGLI
SGDLNPVHFDEDFASKTRFGGRVVHGMLTTSLVSAAVARLPGTVVLLEQSFRYTSPVRIGD
VVRVEGVVSGVEKNRYTIDVKCYTGDKVVAEGVVKVLIW
>non-antioxidant_1548
MIGKRFFQTTSKKIAFAFDIDGVLFRGKKPIAGASDALKLLNRNKIPYILLTNGGGFSERAR
TEFISSKLDVDVSPLQIIQSHTPYKSLVNKYSRILAVGTPSVRGVAEGYGFQDVVHQTDIVR
YNRDIAPFSGLSDEQVMEYSRDIPDLTTKKFDAVLVFNDPHDWAADIQIISDAINSENGML
NTLRNEKSGKPSIPIYFSNQDLLWANPYKLNRFGQGAFRLLVRRLYLELNGEPLQDYTLGK
PTKLTYDFAHHVLIDWEKRLSGKIGQSVKQKLPLLGTKPSTSPFHAVFMVGDNPASDIIGA

QNYGWNSCLVKTGVYNEGDDLKECKPTLIVNDVFDAVTKTLEKYA
>non-antioxidant_1549
SKRADKKAILELFQTYKEPLGNYIGAEGLQRLFEDIQVDPSDVVTLVLAWKLKASSTCEFS
EKEFVEGLANLQVDSLEKLKRKLSSLRKEIEDPSKFRAFYQFVFQYSKEPSQRSLPAETAM
ALWDVLLRGRFSLLDSWLEFLKNNTHSISRDTWNLLYDFSQLSEKDLSDYDENGAWPVLI
DDFVKWLKHEQPNKHES
>non-antioxidant_1550
MGSDKIHHHHHHENLYFQGMSKVFVNISLSLDGFMAPEGMDMAHFSDPTYKNWGAKW
GALMAWALSQQYLREKLKLGTGGETGPVNDMVRHTFERTGAHIMGKRMFEGGERGWP
EEAPFHTPVYVLTHERRNPWVRPGGTTFYFVNDGPEQALALAREAAGERDIRISGGANVI
QQYLNLGLVDELEIALIPVIFGGGRRLFENLHEPLPQFRIDRVLASPTATHLRYVRL
>non-antioxidant_1551
GMIPFPESRLAAQMSFVVEIDKLKTILRQTLLTDSSRRENDAEHSWHIATMAFLLAEYADE
AVQIGRVARMLLIHDIVEIDAGDTFIHDEAGNEDKEERERKAAARLFGLLPPDQAAEYSAL
WQEYEARETADARFADALDRLQPLLHNFETEGGTWKPHGVTRAKVDKLLPRIEAGSKRL
GAYARALVDEAVRRGYLAP
>non-antioxidant_1552
GMSSSDRYMQRGVSSQKEDVHKAIKSIDKGIYPRAFCKIIPDILGGDPEYCNIMHADGAGT
KSSLAYVYWKETGDISVWKGIAQDAVIMNIDDLICVGAVDNILLSSTIGRNKNLIPGEVLA
AIINGTEEVLQMLRDNGIGIYSTGGETADVGDLVRTIIVDSTVTCRMKRQDVISNENIKAGN
VIVGFASYGQTSYETEYNGGMGSNGLTSARHDVFNNVLASKYPESFDPKVPENLVYSGE
MNLTDPYLNVPLDAGKLVLSPTRTYAPLMKEIIHQYKGKLDGVVHCSGGGQTKVLHFTD
ATTHIIKDNLFDVPPLFQLIQGQSNTPWEEMYKVFNMGHRLEIYTDAAHAEGMIAIAKKF
NIEAKIIGRVEAPVAGKRLTITGPQGTEYTYA
>non-antioxidant_1553
GAENNMVRLSRIIIDPERLEEYNAYLKEEIEVSMRLEPGVLVLYAVAEKERPNHVTILEIYAD
EAAYKSHIATPHFKKYKEGTLDMVQMLELIDATPLIPGLKMK
>non-antioxidant_1554
GQDRSPIETQNVETVLRLFDEGWGAQDGWRDVWRETMTPGFRSIFHSNQAVEGIEQAIAF
NAVLFEGFPRLEVVVENVTVEGDNVVVQARLTGAQDGPFLGVPPSGQMVDVPDVTLFTL
ADGQVIEMRYFTDLLAVMTAISAPPEN
>non-antioxidant_1555
HLEGEVNKIKSALLSTNKAVVSLSNGVSVLTSKVLDLKNYIDKQLLPIVNK
>non-antioxidant_1556
VFPSDEFDASISQVNEKINQSLAFIRKSDELLHNVNAGK
>non-antioxidant_1557
GHMAAPILKDVVAYVEVWSSNGTENYSKTFTTQLVDMGAKVSKTFNKQVTHVIFKDGY
QSTWDKAQKRGVKLVSVLWVEKCRTAGAHIDESLFPAANMNEHLS
>non-antioxidant_1558
GMNHNYVEVKYPAIFRDEGTYWDVRFPDVPAAQTFGASVQVAADNAANALAIALFEQSL
PPASDPQYWRLASTEFVVWITMADVQFGPGADTPEPMN
>non-antioxidant_1559
MGSSHHHHHHSSGLVPRGSHMASMTGGQQMGRGSTKVKREATKVQRDISAFKKVMQNI
SLAVNKFNVDIERYVGGDASHLLADGNVLIKATLDGVQSLQNEPPLSSMEALALVGPVQD

LSNQIMLAIQNLIDKKEPLVQAGFGGKVENNLRQQEEAAQKLSELVSTKVPHELADISRQL
SDGIAAGIKKGIDAF
>non-antioxidant_1560
MSHPDLNKLLELWPHIQEYQDLALKHGINDIFQGNGGKLLQVLLITGLTVLPGREGNDAV
DNAGQEYELKSINIDLTKGFSTHHHMNPVIIAKYRQVPWIFAIYRGIAIEAIYRLEPKDLEFY
YDKWERKWYSDGHKDINNPKIPVKYVMEHGTKIY
>non-antioxidant_1561
MMHLNPAEKEKLQIFLASELLLRRKARGLKLNYPEAVAIITSFIMEGARDGKTVAMLMEE
GKHVLTRDDVMEGVPEMIDDIQAEATFPDGTKLVTVHNPIS
>non-antioxidant_1562
MSNNNYIVPGEYRVAEGEIEINAGREKTTIRVSNTGDRPIQVGSHIHFVEVNKELLFDRAEG
IGRRLNIPSGTAARFEPGEEMEVELTELGGNREVFGISDLTNGSVDNKELILQRAKELGYK
GVE
>non-antioxidant_1563
MKINRQQYAESYGPTVGDEVRLADTDLWIEVEKDYTTYGDEVNFGGGKVLREGMGENG
TYTRTENVLDLLLTNALILDYTGIYKADIGVKDGYIVGIGKGGNPDIMDGVTPNMIVGTAT
EVIAAEGKIVTAGGIDTHVHFINPDQVDVALANGITTLFGGGTGPAEGSKATTVTPGPWNIE
KMLKSTEGLPINVGILGKGHGSSIAPIMEQIDAGAAGLKIHEDWGATPASIDRSLTVADEAD
VQVAIHSDTLNEAGFLEDTLRAINGRVIHSFHVEGAGGGHAPDIMAMAGHPNVLPSSTNP
TRPFTVNTIDEHLDMLMVCHHLKQNIPEDVAFADSRIRPETIAAEDILHDLGIISMMSTDAL
AMGRAGEMVLRTWQTADKMKKQRGPLAEEKNGSDNFRLKRYVSKYTINPAIAQGIAHE
VGSIEEGKFADLVLWEPKFFGVKADRVIKGGIIAYAQIGDPSASIPTPQPVMGRRMYGTVG
DLIHDTNITFMSKSSIQQGVPAKLGLKRRIGTVKNCRNIGKKDMKWNDVTTDIDINPETYE
VKVDGEVLTCEPVKELPMAQRYFLF
>non-antioxidant_1564
ELYSALANKCCHVGCTKRSLARFC
>non-antioxidant_1565
SWMEEVIKLCGRELVRAQIAICGMSTWS
>non-antioxidant_1566
DNDSVVEEHGQLSISNGELVNERGEQVQLKGMSSHGLQWYGQFVNYESMKWLRDDWGI
NVFRAAMYTSSGGYIDDPSVKEKVKEAVEAAIDLDIYVIIDWHILSDNDPNIYKEEAKDFF
DEMSELYGDYPNVIYEIANEPNGSDVTWGNQIKPYAEEVIPIIRNNDPNNIIIVGTGTWSQD
VHHAADNQLADPNVMYAFHFYAGTHGQNLRDQVDYALDQGAAIFVSEWGTSAATGDG
GVFLDEAQVWIDFMDERNLSWANWSLTHKDESSAALMPGANPTGGWTEAELSPSGTFV
REKIRES
>non-antioxidant_1567
MMIVIMDNGGQYVHRIWRTLRYLGVETKIIPNTTPLEEIKAMNPKGIIFSGGPSLENTGNCE
KVLEHYDEFNVPILGICLGHQLIAKFFGGKVGRGEKAEYSLVEIEIIDENEIFKGLPKRLKV
WESHMDEVKELPPKFKILARSETCPIEAMKHEELPIYGVQFHPEVAHTEKGEEILRNFAKL
CGEL

Supporting Information S2. The independent dataset consists of 20 antioxidant proteins which are independent from training set.

>Q148E0
MAAPSEAPVTRQVSGHAAPAPVPSGPASWQPLAAAVAELPVLDASGRPVLFGELFRERRA
IVVFVRHFLCYICKEYVEDLAKIPKSFLQEANVTLIVIGQSSYHHIEPFCKLTGYSHEIYVDP
EREIYKRLGMKRGEEIASSGQSPHVKSNILSGSIRSLWRAVTGPLFDFQGDPAQQGGTLILG
PGNNIHFIHHDRNRLDHKPINSVLQLVGVQHVDFTSRPSVIHV
>Q7RTV5
MAAPAPVTRQVSGAAALVPAPSGPDSGQPLAAAVAELPVLDARGQRVPFGALFRERRAVV
VFVRHFLCYICKEYVEDLAKIPRSFLQEANVTLIVIGQSSYHHIEPFCKLTGYSHEIYVDPER
EIYKRLGMKRGEEIASSGQSPHIKSNLLSGSLQSLWRAVTGPLFDFQGDPAQQGGTLILGPG
NNIHFIHRDRNRLDHKPINSVLQLVGVQHVNFTNRPSVIHV
>Q9D1A0
MAAPVTRQVSGCAGRVPSPAGSVTERGQPLAAAVAELPVLDASGRRVTFGALFRERRAVV
VFVRHFLCYVCKEYVEDLAKIPKSVLREADVTLIVIGQSSYHHIEPFCKLTGYSHEIYVDPE
REIYKRLGMKRGEEISSSGQSPHIKSNLLSGSLQSLWRAVTGPLFDFQGDPAQQGGTLILGP
GNNIHFVHRDRNRLDHKPINSVLQLVGVQPVNFMSRPTVIHV
>P80239
MSLIGKEVLPFEAKAFKNGEFIDVTNEDLKGQWSVFCFYPADFSFVCPTELEDLQEQYAAL
KELGVEVYSVSTDTHFVHKGWHDSSEKISKITYAMIGDPSQTISRNFDVLDEETGLADRGT
FIIDPDGVIQTVEINAGGIGRDASNLVNKVKAAQYVRQNPGEVCPAKWEEGGETLTPSLDL
VGKI
>P0AE08
MSLINTKIKPFKNQAFKNGEFIEITEKDTEGRWSVFFFYPADFTFVCPTELGDVADHYEELQ
KLGVDVYAVSTDTHFTHKAWHSSSETIAKIKYAMIGDPTGALTRNFDNMREDEGLADRAT
FVVDPQGIIQAIEVTAEGIGRDASDLLRKIKAAQYVASHPGEVCPAKWKEGEATLAPSLDL
VGKI
>Q7BHK8
MPLLTIGDQFPAYQLTALIGGDLSKVDAKQPGDYFTTITSDEHPGKWRVVFFWPKDFTFVC
PTEIAAFSKLNDEFEDRDAQILGVSIDSEFAHFQWRAQHNDLKTLPFPMLSDIKRELSQAA
GVLNADGVADRVTFIVDPNNEIQFVSATAGSVGRNVDEVLRVLDALQSDELCACNWRKG
DPTLDAGELLKASA
>P0A251
MSLINTKIKPFKNQAFKNGEFIEVTEKDTEGRWSVFFFYPADFTFVCPTELGDVADHYEEL
QKLGVDVYSVSTDTHFTHKAWHSSSETIAKIKYAMIGDPTGALTRNFDNMREDEGLADRA
TFVVDPQGIIQAIEVTAEGIGRDASDLLRKIKAAQYVAAHPGEVCPAKWKEGEATLAPSLD
LVGKI
>P0A5N4
MSIEKLKAALPEYAKDIKLNLSSITRSSVLDQEQLWGTLLASAAATRNPQVLADIGAEATD
HLSAAARHAALGAAAIMGMNNVFYRGRGFLEGRYDDLRPGLRMNIIANPGIPKANFELW
SFAVSAINGCSHCLVAHEHTLRTVGVDREAIFEALKAAAIVSGVAQALATIEALSPS
>Q8L5E0
MVKAVTVLNSSEGVTGTVYFTQEGDGPTTVTGNLSGLKPGLHGFHVHALGDTTNGCMST

GPHFNPVGKEHGAPGDENRHAGDLGNITVGEDGTAAINIVDKQIPLTGPHSIIGRAVVVHS
DPDDLGRGGHELSKSTGNAGGRVACGIIGLQG
>P06728
MFLKAAVLTLALVAITGTRAEVTSDQVANVVWDYFTQLSNNAKEAVEQFQKTDVTQQLS
TLFQDKLGDASTYADGVHNKLVPFVVQLSGHLAQETERVKEEIKKELEDLRDRMMPHAN
KVTQTFGENMQKLQEHLKPYAVDLQDQINTQTQEMKLQLTPYIQRMQTTIKENVDNLHT
SMMPLATNLKDKFNRNMEELKGHLTPRANELKATIDQNLEDLRRSLAPLTVGVQEKLNH
QMEGLAFQMKKNAEELQTKVSAKIDQLQKNLAPLVEDVQSKVKGNTEGLQKSLEDLNR
QLEQQVEEFRRTVEPMGEMFNKALVQQLEQFRQQLGPNSGEVESHLSFLEKSLREKVNSF
MSTLEKKGSPDQPQALPLPEQAQEQAQEQAQEQVQPKPLES
>Q03247
MKVLWVAVVVALLAGCQADMEGELGPEEPLTTQQPRGKDSQPWEQALGRFWDYLRWV
QTLSDQVQEELLNTQVIQELTALMEETMKEVKAYKEELEGQLGPMAQETQARVSKELQA
AQARLGSDMEDLRNRLAQYRSEVQAMLGQSTEELRARMASHLRKLPKRLLRDADDLKK
RLAVYQAGASEGAERSLSAIRERFGPLVEQGQSRAATLSTLAGQPLLERAEAWRQKLHGR
LEEVGVRAQDRLDKIRQQLEEVHAKVEEQGNQMRLQAEAFQARLRSWFEPLVEDMQRQ
WAGLVEKVQLALRPSPTSPPSENH
>P23529
MKVLWAALVVTLLAGCRADVEPEVEVREPAVWQSGQPWELALSRFWDYLRWVQTLSDQ
VQEELLSNQVTQELTLLIEDTMKEVKAYKAELEKELGPVAEDTKARLAKELQAAQARLG
ADMEEVRNRLSQYRSEVQAMLGQSSEELRARLTSHPRKMKRRLQRDIDELQKRMAVYK
AGAQEGAERGVSAIRERLGSLIEQGRLQALASQPLQERAQAWGEQMRGRLEKVGSQARD
RLEEVREQMEEVRVKVEEQAEAFQARLKSWFEPMMEDMRRQWAELIQKVQVAVGASTS
APSQEP
>P30041
MPGGLLLGDVAPNFEANTTVGRIRFHDFLGDSWGILFSHPRDFTPVCTTELGRAAKLAPEF
AKRNVKLIALSIDSVEDHLAWSKDINAYNCEEPTEKLPFPIIDDRNRELAILLGMLDPAEKD
EKGMPGTARVVFVFGPDKKLKLSILYPATTGRNFDEILRVVISLQLTAEKRVATPVDWKDG
DSVMVLPTIPEEEAKKLFPKGVFTKELPSGRKYLRYTPQP
>O19097
MAVVVRVVYCGAUGYKPKYLQLKKKLEDEFPSRLDICGEGTPQVTGFFEVFVAGKLVHS
KKGGDGYVDTESKFLKLVAAIKAALAQA
>P23345
MVKAVAVLGSSEGVKGTIFFTQEGDGPTTVTGSVSGLKPGLHGFHVHALGDTTNGCMST
GPHYNPASKEHGAPEDENRHAGDLGNVTAGADGVANINVTDSQIPLTGPNSIIGRAVVVH
ADPDDLGKGGHELSKSTGNAGGRVACGIIGLQG
>P23346
MVKAVAVLGSSDGVKGTIFFTQEGDGPTAVTGSVSGLKPGLHGFHVHALGDTTNGCMST
GPHYNPASKEHGAPEDENRHAGDLGNVTAGADGVANINVTDSQIPLTGPNSIIGRAVVVH
ADPDDLGKGGHELSKSTGNAGGRVACGIIGLQG
>O65198
MASHSLMSPSPLTSHSLLRSSFSGVSVKLSPQFSTLSRSKFQPLSVVAAAKKAVAVLKGNST
VEGVVTLTQENESPTTVNVRITGLTPGLHGFHLHEYGDTTNGCISTGPHFNPNQLTHGAPE
DEIRHAGDLGNIIADANGVAEATIVDNQIPLTGPNSVIGRALVVHELEDDLGKGGHELSLST

GNAGGRLACGVVGLTPV
>P93407
MQAILAAAMAAQTLLFSATAPPASLFQSPSSARPFHSLRLAAGPAGAAAARALVVADATK
KAVAVLKGTSQVEGVVTLTQDDQGPTTVNVRVTGLTPGLHGFHLHEFGDTTNGCISTGPH
FNPNNLTHGAPEDEVRHAGDLGNIVANAEGVAEATIVDKQIPLSGPNSVVGRAFVVHELE
DDLGKGGHELSLSTGNAGGRLACGVVGLTPL
>P11964
MASQTLVSPSPLSSHSLLRTSFSGVSVKLAPQFSTLATSNFKPLTVVAAAKKAVSVLKGTSA
VEGVVTLTQDDEGPTTVNVRITGLTPGLHGFHLHEYGDTTNGCISTGPHFNPNKLTHGAPE
DEIRHAGDLGNIVANAEGVAEATIVDNQIPLTGPNSVVGRALVVHELQDDLGKGGHELSLS
TGNAGGRLACGVVGLTPV
>P10792
MAAHTIFTTTSTTNSFLFPIASSNTNSAPSLSSSFHGVSLKVKSKTPQSLTLSSVTSPKPFIVF
AATKKAVAVLKGTSNVEGVVTLTQDDDGPTTVKVRITGLAPGLHGFHLHEFGDTTNGCM
STGPHFNPNGLTHGAPGDEVRHAGDLGNIEANASGVAEATLVDNQIPLSGPNSVVGRALV
VHELEDDLGKGGHELSLTTGNAGGRLACGVVGLTPI
